# Organophotocatalysed synthesis of 2-piperidinones in one step via [1 + 2 + 3] strategy

**DOI:** 10.1038/s41467-023-40197-x

**Published:** 2023-09-02

**Authors:** Yi-Dan Du, Shan Wang, Hai-Wu Du, Xiao-Yong Chang, Xiao-Yi Chen, Yu-Long Li, Wei Shu

**Affiliations:** 1 Shenzhen Grubbs Institute and Department of Chemistry, Southern University of Science and Technology, Shenzhen, 518055 Guangdong P. R. China; 2 State Key Laboratory of Elemento-Organic Chemistry, Nankai University, 300071 Tianjin, P. R. China; 3 College of Chemistry and Environmental Engineering, Sichuan University of Science and Engineering, 643000 Zigong, P. R. China

**Keywords:** Photocatalysis, Synthetic chemistry methodology, Homogeneous catalysis

## Abstract

Six-membered *N*-containing heterocycles, such as 2-piperidinone derivatives, with diverse substitution patterns are widespread in natural products, drug molecules and serve as key precursors for piperidines. Thus, the development of stereoselective synthesis of multi-substituted 2-piperidinones are attractive. However, existing methods heavily rely on modification of pre-synthesized backbones which require tedious multi-step procedure and suffer from limited substitution patterns. Herein, an organophotocatalysed [1 + 2 + 3] strategy was developed to enable the one-step access to diverse substituted 2-piperidinones from easily available inorganic ammonium salts, alkenes, and unsaturated carbonyl compounds. This mild protocol exhibits exclusive chemoselectivity over two alkenes, tolerating both terminal and internal alkenes with a wide range of functional groups.

## Abstract (web summary)

The synthesis of 2-piperidinone derivatives remains challenging. Here, the authors develop an organophotocatalysed [1 + 2 + 3] strategy to enable the one-step access to diverse 2-piperidinones from ammonium salts, alkenes, and unsaturated carbonyl compounds.

## Introduction

**Fig. 1:**
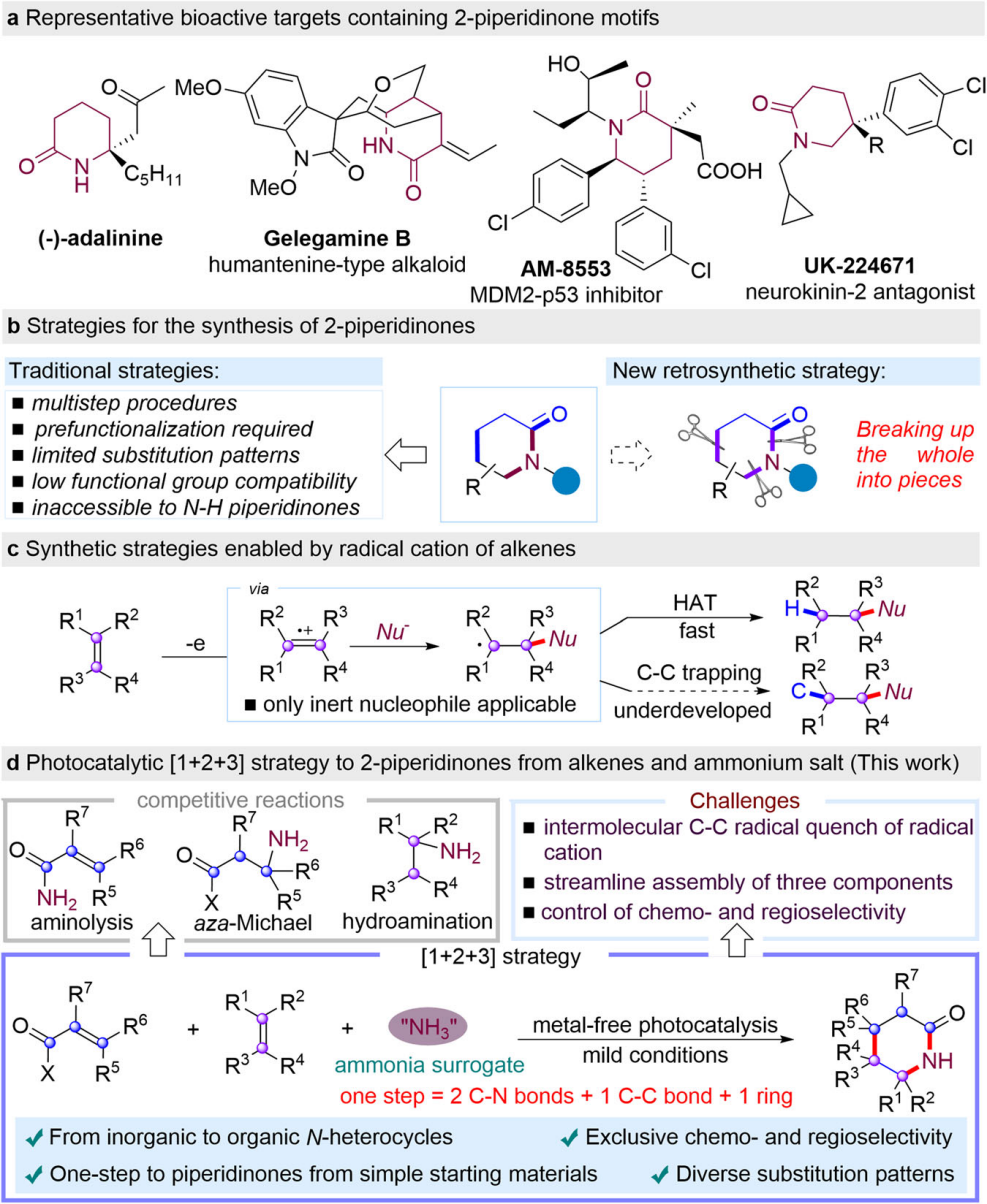
Significance and strategies for the synthesis of 2-piperidinones. **a** Representative bioactive targets containing 2-piperidinone motifs. **b** Strategies for the synthesis of 2-piperidinones. **c** Synthetic strategies enabled by radical cation of alkenes. **d** Photocatalytic [1 + 2 + 3] strategy to 2-piperidinones from alkenes and ammonium salt (This work).

2-Piperidinones are important core substructures in many pharmaceuticals and natural products (Fig. 1a)^1–7^ and serve as key precursors or intermediates for the synthesis of multi-substituted piperidines and medicinally relevant compounds^8,9^. In particular, medicinal chemists found that *N*-containing heterocycles, such as 2-piperidinones and derived piperidines are among the second-most prevalent heterocycles in pharmaceutical core structures^1,2,10^. Classical methods to access this structural motif heavily rely manipulation of cyclic precursors, such as the hydrogenation of unsaturated δ-lactams^11–17^ and oxidation of piperidines (Fig. 1b)^18,19^. However, the tedious multi-step preinstallation of such backbone structures as well as the strong reductive or oxidative conditions severely hampered the application of these methods. Accordingly, stepwise approaches such as annulations from advanced precursors by reduction-cyclization cascade provided an alternative to access multi-substituted 2-pyridinones^20–22^. Although these strategies are useful, they are limited to specific classes of coupling partners, resulting in specific substituted 2-pyridinones with additional manipulation steps required. To this end, Alper developed a dual catalyzed carbonylation of pyrrolidines by ring expansion to form 6-substituted-2-piperidinones using [Co_2_(CO)_8_/Ru_3_(CO)_12_]^23^. In 2007, Landais reported a Et_3_B/O_2_ mediated multi-component process by involving a tandem radical intermolecular additions-lactamization sequence to access 2-piperidinones^24^. In general, existing methods suffer from limited scope and substitution patterns of 2-piperidinones from advanced synthetic intermediates with poor functional group tolerance. Thus, a streamlined protocol to piperidinones with diverse substitution patterns from easily-available and cheap starting materials is highly desirable yet challenging.

Over the past decades, visible-light enabled chemical bond-forming processes have become an attractive platform for organic synthesis^25–28^. Nicewicz reported the seminal work on alkene activation by photo-initiated single electron oxidation to facilitate hydrofunctionalization with inert nucleophiles (Fig. 1c). However, aliphatic amines are not feasible to undergo such hydrofunctionalization of alkenes due to the low oxidative potentials of starting and resultant amines (Fig. 1c)^29–40^. Recently, our group developed the direct synthesis of aliphatic primary amines enabled by hydroamination of alkenes from ammonium carbonate^41,42^. To date, photocatalytic intermolecular functionalizations of alkenes via the cation radical intermediates of alkenes are limited to hydrofunctionalizations^43^, partially due to the fast hydrogen atom transfer process to quench the stable alkyl radical species (Fig. 1c). To the best of our knowledge, intermolecular trapping of such radicals by C-C bond formation remains elusive^44–50^. Thus, we question the feasibility of quenching the carbon-centered radicals by intermolecular C–C bond-forming to substantially expand chemical space of this reaction mode. Herein, we report a metal-free photocatalytic [1 + 2 + 3] strategy for the rapid construction of 2-piperidinones from inorganic ammonium salts and alkenes (Fig. 1d)^51–55^. The use of inorganic salts facilitates the selective C–N bond formation over two different alkenes as well as the radical trap of C-C bond-forming process, providing a rapid access to *N*-unprotected 2-piperidinones from inorganic ammonium salts.

## Results

### Reaction optimization

We commenced our studies with 4-fluoro-β,β-dimethylstyrene **1a** and methyl α-phenylacrylate **2a** to probe the feasibility of this [1 + 2 + 3] strategy. After extensive optimization of reaction parameters, the use of [Mes-3,6-*t*-Bu_2_-Acr-Ph]^+^BF_4_^−^ (2.5 mol%) as catalyst, ammonium acetate (3.0 equiv) as nitrogen source and LiBF_4_ (1.0 equiv) as additive in CH_3_CN/PhCl (10:1) under blue LED irradiation at room temperature was defined as standard conditions (Table 1, entry 1), providing the desired 2-piperidinone **3a** in 88% yield with a 3.7:1 dr. The structure and major isomer of **3a** were confirmed by X-ray diffraction analysis. The use of ammonia surrogate is essential for this reaction. Other ammonium salts could mediate the desired process, albeit leading to the formation of **3a** in inferior yields (Table 1, entries 2–6 and Supplementary Table 3). Other acridinum-based photocatalyst proved to be less effective, delivering **3a** in 54–61% yields (Table 1, entries 7–8). Moreover, additive effect showed a significant impact on the diastereoselectivity. In the absence of LiBF_4_, **3a** was obtained in comparable yield (80% yield) with only 2.6:1 dr (Table 1, entry 9). The use of other Lewis acids or Lewis bases as additive provided **3a** in lower efficiency and/or diastereomeric ratios (Table 1, entries 10–14 and Supplementary Table 8). Control experiments revealed that both photocatalyst and light irradiation are necessary for the [1 + 2 + 3] transformation (Table 1, entry 15).

**Table 1:** Condition evaluation of the reaction^a^


Entry	Variation from “standard condition”	Yield of 3a (dr)^b^
1	None	88% (3.7:1)
2	(NH_4_)_2_CO_3_ instead of NH_4_OAc	41% (1:1.1)
3	NH_4_HCO_3_ instead of NH_4_OAc	41% (1:1.1)
4	NH_2_CO_2_NH_4_ instead of NH_4_OAc	39% (1:1.2)
5	NH_4_Cl instead of NH_4_OAc	trace
6	NH_4_BF_4_ instead of NH_4_OAc	trace
7	Catalyst **A** instead of Catalyst **C**	61% (2.1:1)
8	Catalyst **B** instead of Catalyst **C**	54% (3.6:1)
9	w/o LiBF_4_	80% (2.6:1)
10^c^	Sc(OTf)_3_ instead of LiBF_4_	56% (3.3:1)
11^c^	Zn(OTf)_2_ instead of LiBF_4_	73% (3.7:1)
12	2,6-*t*Bu_2_pyridine instead of LiBF_4_	88% (2.7:1)
13	2,6-lutidine instead of LiBF_4_	82% (2.5:1)
14	collidine instead of LiBF_4_	69% (2.9:1)
15	w/o PC, w/o light	N.D.

^a^The reaction was conducted using **1a** (0.1 mmol), **2a** (0.2 mmol) under indicated conditions.

^b^Yield and diastereomeric ratio (dr) were determined by ^1^H NMR of crude mixture of the reaction using PhTMS as internal standard.

^c^Additive (20 mol%) was used.

### Substrate scope

**Fig. 2:**
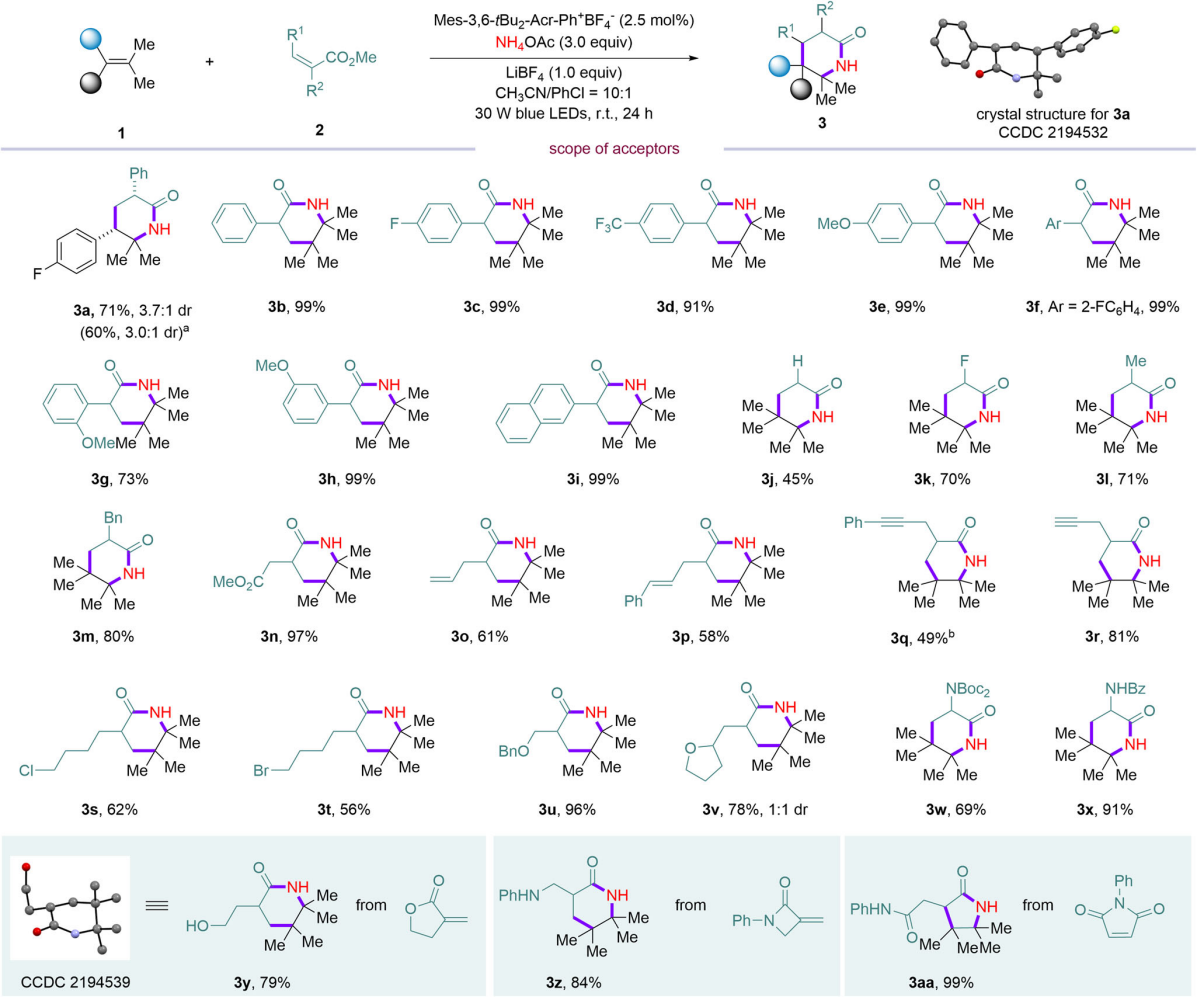
Scope of the acceptors. The reaction was conducted using **1** (0.1 mmol), **2** (0.2 mmol) under standard conditions unless otherwise noted. Diastereomeric ratio (dr) was determined by ^1^H NMR of crude mixture of the reaction. Isolated yield after flash chromatography. The disordered part, solvent molecules and hydrogen atoms of crystal structures have been omitted for clarity, for details, see Supplementary Figs. 15 and 16. ^a^Corresponding Weinreb amide was used. ^b^5 mol% photocatalyst was used.

**Fig. 3:**
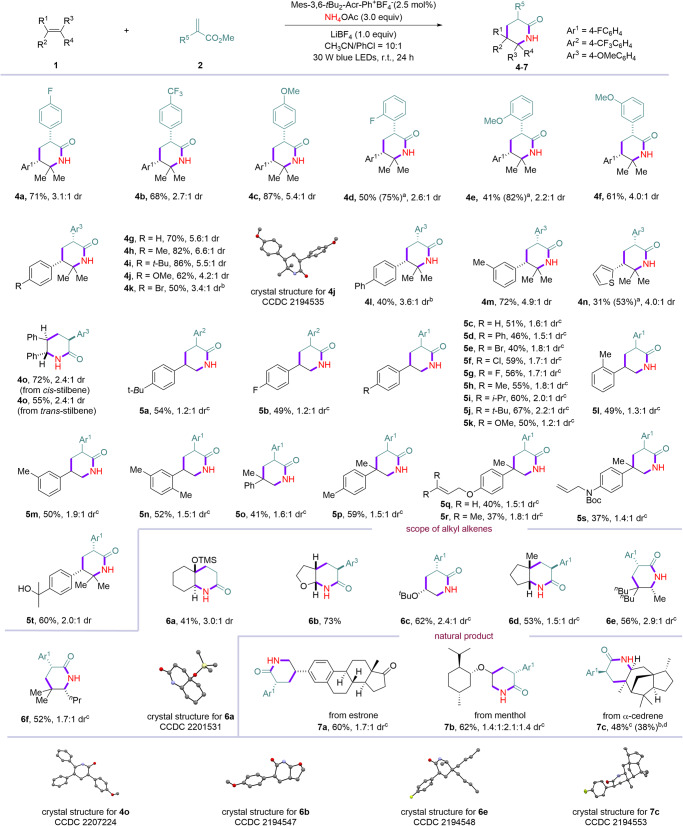
Scope of alkenes. The reaction was conducted using **1** (0.1 mmol), **2** (0.2 mmol) under standard conditions unless otherwise noted. Diastereoisomeric ratio (dr) was determined by ^1^H NMR of crude mixture of the reaction. Isolated yield after flash chromatography. The disordered part, solvent molecules and hydrogen atoms of crystal structures have been omitted for clarity. For more details, see Supplementary Figs. 17–24. ^a^ Yield based on the recovery of alkenes. ^b^ 5 mol% PC was used. ^c^ The reaction was conducted using **1** (0.1 mmol), **2** (0.2 mmol), PC (5 mol%), NH_4_OAc (0.3 mmol), LiBF_4_ (0.1 mmol) in CH_3_CN:PhCl = 100:1 (10.0 mL CH_3_CN). ^d^ The reaction was conducted on 2.0 mmol scale.

After identifying the optimized reaction conditions, we set to explore the scope of this [1 + 2 + 3] strategy enabled 2-piperidinone synthesis. First, the scope of radical acceptor was tested and the results are summarized in Fig. 2. Diverse α-aryl acrylate with *para*- (**3a**–**3e**), *ortho*- (**3f** and **3g**) and *meta*- (**3h**) substituents on the aromatic rings were all well-tolerated, giving the desired 2-piperidinone products in excellent yields (71–99%). In addition, acrylamides could be employed as the acceptor to undergo coupling and cyclization to give **3a** in 60% yield. Moreover, fused aryl, alkyl, fluoro-, and benzyl substituted acrylates were all good substrates, affording diverse substitution patterns at 3-position of 2-piperidinones (**3i–3m**) in good yields. Notably, esters, terminal and internal alkenes, terminal and internal alkynes were tolerated (**3n**–**3r**) under this metal-free conditions. The reaction underwent chemoselective [1 + 2 + 3] reaction to deliver desired products (**3n**–**3r**) in 49–97% yields, leaving chemical space for further elaboration. Halides and ether containing acrylates were successfully involved in the reaction, furnishing corresponding 2-piperidinones (**3s**–**3v**) in 56–96% yields. Amides without or with free protons are both good substrates in the reaction, delivering **3w** and **3x** in 69% and 91% yields, respectively. Interestingly, vinyl γ-lactone like Tulipalin A could be applied to this reaction, giving **3y** in 79% yield. The structure of **3y** was confirmed by X-ray diffraction analysis. Methylene β-lactam underwent the desired reaction to furnish **3z** in 84% yield. Non-polarized electron-deficient alkenes were also compatible under the reaction conditions, delivering corresponding *N*-phenyl-2-(4,4,5,5-tetramethyl-2-oxopyrrolidin-3-yl)acetamide **3aa** in 99% yield. Next, the scope with respect to the other alkenes was examined under the standard conditions. A wide range of functional groups and diverse substitution patterns were amenable in the reaction (Fig. 3). Various 2,2-dimethylstyrenes with electron-donating or electron-withdrawing groups reacted smoothly with different alkene acceptors to furnish desired [1 + 2 + 3] products (**4a**–**4n**) in exclusive regioselectivity and good yields with 2.2:1-6.6:1 dr. Notably, both configurations of stilbene were applicable in the reaction, delivering 3,5,6-trisubstituted 2-piperdinone **4o** in 72% and 55% yields with identical diastereomeric ratio.

1-Substituted styrenes with diverse substitution patterns on arenes are good substrates in the reaction. Electron-donating and electron-withdrawing groups at *para*-, *meta*- and *ortho*-position of arenes are all tolerated, affording 3,5-disubstituted 2-piperidinones (**5a**–**5n**) in synthetic useful yields. 1,1-Disubstituted styrenes could be applied to the reaction to furnish 3,5,5-trisubstituted 2-piperidiones (**5o**–**5s**) in moderate yields. Moreover, the reaction underwent chemoselective functionalization between multiple alkenes (**5q**–**5s**). Furthermore, free alcohol could also be tolerated in the reaction (**5t**). Notably, vinyl silyl ether was successfully involved in the reaction to give bicyclic 2-piperidinone **6a** in 41% yield. Cyclic and acyclic vinyl ethers are both reactive in the reaction, affording bicyclic piperidinone **6b** in 73% yield as single diastereomer and 3,5-disubstituted 2-piperidinone **6c** in 62% yield. It deserves mentioning that aliphatic alkenes were applicable in the reaction. Cyclic aliphatic alkene was converted to octahydro-2*H*-cyclopenta[*b*]pyridin-2-one (**6d**) in 53% yield. Acyclic alkyl alkenes reacted to give 3,5,5,6-tetrasubstituted 2- piperidinones (**6e** and **6f**) in 56% and 52% yields. Furthermore, the [1 + 2 + 3] strategy was applied to late-stage functionalization of complex molecules. Alkenes derived from natural products, such as estrone, menthol, and α-cedrene were all compatible with the reaction conditions, successfully affording corresponding natural product-based 2-piperidinones (**7a**–**7c**) in 48%-62% yields. Moreover, the reaction could be scaled up to 2.0 mmol, affording **7c** in 38% yield. The major isomers of compounds (**4j,** **4o,** **6a,** **6b,** **6e**, and **7c**) were confirmed by X-ray diffraction analysis.

### Control experiments and mechanistic consideration

**Fig. 4:**
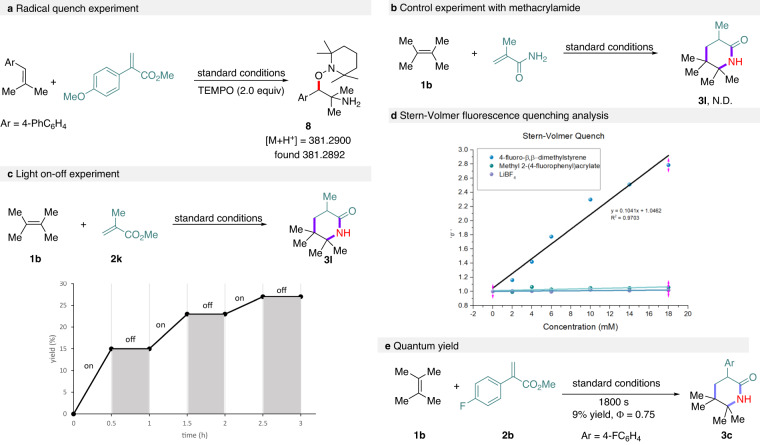
Control experiments and mechanistic investigations. **a** Radical quench experiment. **b** Control experiment with methacrylamide. **c** Light on-off experiment. **d** Stern-Volmer fluorescence-quenching analysis. **e** Quantum yield.

Next, a series of control experiments were carried out to shed light on the reaction mechanism (Fig. 4). First, the reaction of 4-(2-methylprop-1-en-1-yl)-1,1′-biphenyl with methyl 2-(4-methoxyphenyl)acrylate was conducted in the presence of a radical scavenger TEMPO under otherwise identical to standard conditions (Fig. 4a, see more information in Supplementary Information). The desired [1 + 2 + 3] reaction was completely shut down, suggesting the involvement of radical nature in the reaction process. The TEMPO-trapped adduct **8** could be observed by HR-MS analysis. Second, an experiment between **1b** and methacrylamide was conducted under standard conditions (Fig. 4b). However, no desired product **3l** was detected, excluding the possibility of preformation acrylamides during the reaction course. Furthermore, the light on-off experiments of **1b** and methyl methacrylate was conducted under standard conditions (Fig. 4c). The results indicated the reaction undergo a catalytic process instead of a radical chain pathway. In addition, fluorescence-quenching experiments were conducted to further probe the reaction mechanism using 4-fluoro-β,β-dimethylstyrene (**1a**) and methyl 2-(4-fluorophenyl)acrylate (**2b**) (Fig. 4d). Stern-Volmer analysis and time-resolved fluorescence spectroscopy (K_sv_ = 48.07 M^−1^, k_q_ = 4.34 × 10^9 ^M^−1^ s^−1^, see Supplementary Information) indicated that this reaction may proceed through a reductive quenching mechanism of Mes-3,6-*t*Bu_2_-Acr-Ph^+^BF_4_^−^ by 4-fluoro-β,β-dimethylstyrene. The quantum yield (Φ) of the reaction using **1b** and **2b** was determined to be 0.75 (Fig. 4e), indicating the reaction may undergo a catalytic radical process. Yet, a slow chain propagation mechanism cannot be ruled out at this stage ^56^.

**Fig. 5:**
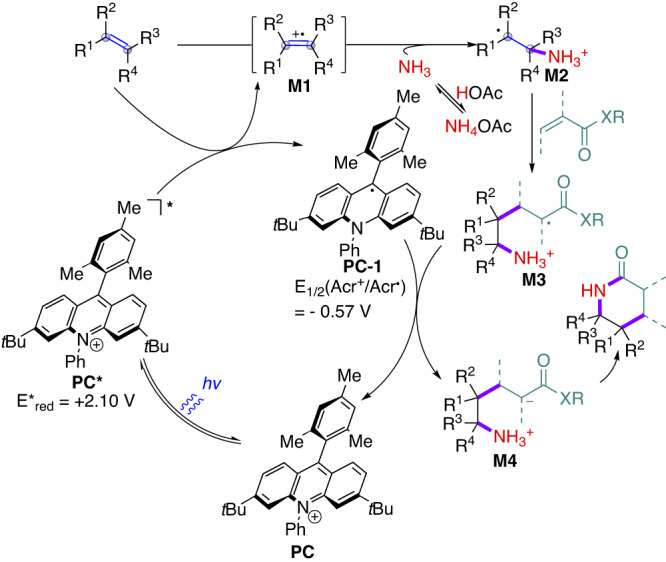
Proposed mechanism. The plausible mechanism for photocatalytic synthesis of 2-piperidinones from alkenes and ammonium salt by [1 + 2 + 3] strategy.

Based on the experimental results and literature precedence^29–44^, a plausible reaction mechanism was proposed and depicted in Fig. 5. First, excited PC* was generated from **PC** by visible light irradiation. PC* interacted with the alkenes via single electron oxidation to give radical cation intermediate **M1** in conjunction with reduced photocatalyst species **PC-1**. **M1** is trapped by ammonia released from NH_4_OAc to deliver intermediate **M2**. **M2** could undergo radical addition with acrylates to generate **M3** by C–C bond-formation, which could be further reduced by **PC-1** to give intermediate **M4** and regenerate **PC**. Finally, the intramolecular lactamization of **M4** generated the desired 2-piperidinone products.

## Discussion

In conclusion, an organophotocatalysed [1 + 2 + 3] strategy for the modular access to unprotected 2-piperidinones has been developed at room temperature. The use of an inorganic ammonium salt as ammonia surrogate enables the construction of *N*-containing heterocycles from two different alkenes with exclusive chemoselectivity. The reaction forges two C–N bonds and one C–C bond sequentially in one step to construct 2-piperidinones with diverse substitution patterns, providing a streamlined access to 2-piperidinones from easily available starting materials. Mechanistic studies revealed the process was initiated by single electron oxidation of alkenes, followed by an intermolecular nucleophilic attack and intermolecular radical trap to form a C–C bond, representing an intermolecular trap of this carbon radical resulting from radical cation of alkenes to form C–C bonds. We anticipate this reaction will pave the way for this reaction mode to discover new reactions and open the avenue for catalytic transforming inorganic ammonium salts into *N*-containing organic frameworks.

## Methods

### General procedure A for the photocatalytic selective [1 + 2 + 3] construction of 2-piperidinones from alkenes and ammonium salt

Under an inert atmosphere, an oven-dried Schlenk-tube equipped with a magnetic stir bar was charged with *N*-Ph-9-mesityl 3,6-di-*tert-*butylacridinium tetrafluoroborate (1.4 mg, 2.5 μmol, 2.5 mol%), NH_4_OAc (23.1 mg, 0.3 mmol), LiBF_4_ (9.4 mg, 0.1 mmol) and alkene (if solid, 0.1 mmol), CH_3_CN (1.0 mL), alkene (if liquid, 0.1 mmol), acceptor (0.2 mmol) and PhCl (0.1 mL) were added consecutively via syringe. The tube was sealed with a Teflon-coated septum cap, and stirred at ambient temperature under irradiation with 30 W blue LEDs for 12 or 24 h. Upon completion, the reaction mixture was quenched with water and extracted with ethyl acetate. The combined organic phase was concentrated in vacuum. The crude mixture was analyzed by ^1^H NMR with PhTMS as internal standard to determine the conversion and was directly purified by column chromatography on silica gel to give the corresponding compound.

### General procedure B for the photocatalytic selective [1 + 2 + 3] construction of 2-piperidinones from alkenes and ammonium salt

Under an inert atmosphere, an oven-dried Schlenk-tube equipped with a magnetic stir bar was charged with *N*-Ph-9-mesityl 3,6-di-*tert-*butylacridinium tetrafluoroborate (2.9 mg, 5 μmol, 5 mol%), NH_4_OAc (23.1 mg, 0.3 mmol), LiBF_4_ (9.4 mg, 0.1 mmol) and alkene (if solid, 0.1 mmol), CH_3_CN (1.0 mL), alkene (if liquid, 0.1 mmol), acceptor (0.2 mmol) and PhCl (0.1 mL) were added consecutively via syringe. The tube was sealed with a Teflon-coated septum cap, and stirred at ambient temperature under irradiation with 30 W blue LEDs for 24 h. Upon completion, the reaction mixture was quenched with water and extracted with ethyl acetate. The combined organic phase was concentrated in vacuum. The crude mixture was analyzed by ^1^H NMR with PhTMS as internal standard to determine the conversion and was directly purified by column chromatography on silica gel to give the corresponding compound.

### General procedure C for the photocatalytic selective [1 + 2 + 3] construction of 2-piperidinones from alkenes and ammonium salt

Under an inert atmosphere, an oven-dried Schlenk-tube equipped with a magnetic stir bar was charged with *N*-Ph-9-mesityl 3,6-di-*tert-*butylacridinium tetrafluoroborate (2.9 mg, 5 μmol, 5 mol%), NH_4_OAc (23.1 mg, 0.3 mmol), LiBF_4_ (9.4 mg, 0.1 mmol) and alkene (if solid, 0.1 mmol), CH_3_CN (10.0 mL), alkene (if liquid, 0.1 mmol), acceptor (0.2 mmol) and PhCl (0.1 mL) were added consecutively via syringe. The tube was sealed with a Teflon-coated septum cap, and stirred at ambient temperature under irradiation with 30 W blue LEDs for 24 h. Upon completion, the reaction mixture was quenched with water and extracted with ethyl acetate. The combined organic phase was concentrated in vacuum. The crude mixture was analyzed by ^1^H NMR with PhTMS as internal standard to determine the conversion and was directly purified by column chromatography on silica gel to give the corresponding compound.

### Supplementary information

Supplementary Information

Peer Review File

## Data Availability

The experimental data and the characterization data for all the compounds generated in this study have been provided in the Supplementary Information. Crystallographic data for the structures reported in this paper have been deposited at the Cambridge Crystallographic Data Centre, under deposition numbers 2194532 (**3a**), 2194539 (**3y**), 2194535 (**4j**), 2207224 (**4o**), 2194544 (**5j**), 2194546 (**5o**), 2201531 (**6a**), 2194547 (**6b**), 2194548 (**6e**) and 2194553 (**7c**). These data can be obtained free of charge from The Cambridge Crystallographic Data Centre via www.ccdc.cam.ac.uk/data_request/cif.

## References

[1] Vitaku E, Smith DT, Njardarson JT (2014). Analysis of the Structural Diversity, Substitution Patterns, and Frequency of Nitrogen Heterocycles among U.S. FDA Approved Pharmaceuticals. J. Med. Chem..

[2] Taylor RD, MacCoss M, Lawson ADG (2014). Rings in Drugs. J. Med. Chem..

[3] O’Hagan D (2000). Pyrrole, Pyrrolidine, Pyridine, Piperidine and Tropane Alkaloids. Nat. Prod. Rep..

[4] Honda T, Kimura M (2000). Concise Enantiospecific Synthesis of a Coccinellied Alkaloid, (-)-Adalinine. Org. Lett..

[5] Liu Y (2013). Regiospecific 6-*endo*-Annulation of in situ Generated 3,4-Dienamides/Acids: Synthesis of δ‑Lactams and δ‑Lactones. Org. Lett..

[6] Lucas BS (2012). An Expeditious Synthesis of the MDM2-p53 Inhibitor AM-8553. J. Am. Chem. Soc..

[7] MacKenzie AR (2002). Structure-Activity Relationships of 1-Alkyl-5-(3,4-dichlorophenyl)−5-{2-[(3-substituted)−1-azetidinyl]ethyl}−2-piperidones. 1. Selective Antagonists of the Neurokinin-2 Receptor. J. Med. Chem..

[8] Escolano C, Amat M, Bosch J (2006). Chiral Oxazolopiperidone Lactams: Versatile Intermediates for the Enantioselective Synthesis of Piperidine-Containing Natural Products. Chem. Eur. J..

[9] Weintraub PM, Sabol JS, Kane JM, Borcherding DR (2003). Recent Advances in the Synthesis of Piperidones and Piperidines. Tetrahedron.

[10] Castro SD (2022). A Versatile Class of 1,4,4-Trisubstituted Piperidines Block Coronavirus Replication In Vitro. Pharmaceuticals.

[11] Chen B (2021). Production of Piperidine and δ-Lactam Chemicals from Biomass-Derived Triacetic Acid Lactone. Angew. Chem. Int. Ed..

[12] Wagener T, Lückemeier L, Daniliuc CG, Glorius F (2021). Interrupted Pyridine Hydrogenation: Asymmetric Synthesis of δ-Lactams. Angew. Chem. Int. Ed..

[13] Wei Y, Rao B, Cong X, Zeng X (2015). Highly Selective Hydrogenation of Aromatic Ketones and Phenols Enabled by Cyclic (Amino)(alkyl)carbene Rhodium Complexes. J. Am. Chem. Soc..

[14] Hassan IS (2019). Asymmetric δ-Lactam Synthesis with a Monomeric Streptavidin Artificial Metalloenzyme. J. Am. Chem. Soc..

[15] Wysocki J, Schlepphorst C, Glorius F (2015). Asymmetric Homogeneous Hydrogenation of 2-Pyridones. Synlett.

[16] Zacharie B, Abbott SD, Baigent CB, Doyle C, Yalagala RS (2018). An Efficient Two-Step Preparation of α-, β-, γ- or δ-Amino Acids from 2-Pyrazinones, 2-Hydroxypyrimidines or 2-Pyridones Respectively. Eur. J. Org. Chem..

[17] Weilbeer C, Sickert M, Naumov S, Schneider C (2017). The Brønsted Acid-Catalyzed, Enantioselective Aza-Diels-Alder Reaction for the Direct Synthesis of Chiral Piperidones. Chem. Eur. J..

[18] Jin X, Kataoka K, Yatabe T, Yamaguchi K, Mizuno N (2016). Supported Gold Nanoparticles for Efficient α-Oxygenation of Secondary and Tertiary Amines into Amides. Angew. Chem. Int. Ed..

[19] Khusnutdinova JR, Ben-David Y, Milstein D (2014). Oxidant-Free Conversion of Cyclic Amines to Lactams and H_2_ Using Water as the Oxygen Atom Source. J. Am. Chem. Soc..

[20] He Y (2013). One-Pot Synthesis of Optically Enriched 2‑Piperidinones from Aliphatic Aldehydes and Cyanoacrylamides. Org. Lett..

[21] Valero G (2009). Highly Enantioselective Organocatalytic Synthesis of Piperidines. Formal Synthesis of (-)-Paroxetine. Tetrahedron Lett..

[22] White NA, DiRocco DA, Rovis T (2013). Asymmetric N-Heterocyclic Carbene Catalyzed Addition of Enals to Nitroalkenes: Controlling Stereochemistry via the Homoenolate Reactivity Pathway to Access δ-Lactams. J. Am. Chem. Soc..

[23] Wang MD, Alper H (1992). Regioselective Synthesis of Piperidinones by Metal-Catalyzed Ring Expansion-Carbonylation Reactions. Remarkable Cobalt and/or Ruthenium Carbonyl Catalyzed Rearrangement and Cyclization Reactions. J. Am. Chem. Soc..

[24] Godineau E, Landais Y (2007). Multicomponent Radical Processes: Synthesis of Substituted Piperidinones. J. Am. Chem. Soc..

[25] Huang H-M, Bellotti P, Glorius F (2022). Merging Carbonyl Addition with Photocatalysis. Acc. Chem. Res..

[26] Holmberg-Douglas N, Nicewicz DA (2022). Photoredox-Catalyzed C-H Functionalization Reactions. Chem. Rev..

[27] Chang L, An Q, Duan L, Feng K, Zuo Z (2022). Alkoxy Radicals See the Light: New Paradigms of Photochemical Synthesis. Chem. Rev..

[28] Capaldo L, Ravelli D, Fagnoni M (2022). Direct Photocatalyzed Hydrogen Atom Transfer (HAT) for Aliphatic C-H Bonds Elaboration. Chem. Rev..

[29] Wu F (2020). Direct Synthesis of Bicyclic Acetals via Visible Light Catalysis. iScience.

[30] Onuska NPR, Schutzbach-Horton ME, Rosario Collazo JL, Nicewicz DA (2020). *anti*-Markovnikov Hydroazidation of Activated Olefins via Organic Photoredox Catalysis. Synlett.

[31] Wu F, Wang L, Chen J, Nicewicz DA, Huang Y (2018). Direct Synthesis of Polysubstituted Aldehydes via Visible-Light Catalysis. Angew. Chem. Int. Ed..

[32] Wang L, Wu F, Chen J, Nicewicz DA, Huang Y (2017). Visible-Light-Mediated [4+2] Cycloaddition of Styrenes: Synthesis of Tetralin Derivatives. Angew. Chem. Int. Ed..

[33] Griffin JD, Cavanaugh CL, Nicewicz DA (2017). Reversing the Regioselectivity of Halofunctionalization Reactions through Cooperative Photoredox and Copper Catalysis. Angew. Chem. Int. Ed..

[34] Margrey KA, Nicewicz DAA (2016). A General Approach to Catalytic Alkene *Anti*-Markovnikov Hydrofunctionalization Reactions via Acridinium Photoredox Catalysis. Acc. Chem. Res..

[35] Qin Y (2021). Mechanistic Investigation and Optimization of Photoredox *Anti*-Markovnikov Hydroamination. J. Am. Chem. Soc..

[36] Miller DC (2019). *Anti*-Markovnikov Hydroamination of Unactivated Alkenes with Primary Alkyl Amines. J. Am. Chem. Soc..

[37] Musacchio AJ (2017). Catalytic Intermolecular Hydroaminations of Unactivated Olefins with Secondary Alkyl Amines. Science.

[38] Wilger DJ, Grandjean J-MM, Lammert TR, Nicewicz DA (2014). The Direct *anti*-Markovnikov Addition of Mineral Acids to Styrenes. Nat. Chem..

[39] Nguyen TM, Manohar N, Nicewicz DA (2014). *anti*-Markovnikov Hydroamination of Alkenes Catalyzed by a Two-Component Organic Photoredox System: Direct Access to Phenethylamine Derivatives. Angew. Chem. Int. Ed..

[40] Nicewicz DA (2013). Direct Catalytic *Anti*-Markovnikov Addition of Carboxylic Acids to Alkenes. J. Am. Chem. Soc..

[41] Du YD, Chen BH, Shu W (2021). Direct Access to Primary Amines from Alkenes by Selective Metal-Free Hydroamination. Angew. Chem. Int. Ed..

[42] Yu T, Li P (2021). One-Step Access to Primary Amines from Alkenes and Ammonium Carbonate by One-Step Metal-Free Catalysis. Chin. J. Org. Chem..

[43] Chen BH, Du YD, Shu W (2022). Organophotocatalytic Regioselective C-H Alkylation of Electron-Rich Arenes Using Activated and Unactivated Alkenes. Angew. Chem. Int. Ed..

[44] Grandjean J, Nicewicz DA (2013). Synthesis of Highly Substituted Tetrahydrofurans via Catalytic Polar-Radical Crossover Cycloadditions of Alkenes and Alkenols. Angew. Chem. Int. Ed..

[45] Belleau B, Au-Young YK (1969). Electrochemical Methoxylation of Vinyl Ethers: A Novel Anodic Dimerization Reaction. Can. J. Chem..

[46] Reed NL, Herman MI, Miltchev VP, Yoon TP (2019). Tandem Copper and Photoredox Catalysis in Photocatalytic Alkene Difunctionalization Reactions. Beilstein J. Org. Chem..

[47] Zeller MA, Riener M, Nicewicz DA (2014). Butyrolactone Synthesis via Polar Radical Crossover Cycloaddition Reactions: Diastereoselective Syntheses of Methylenolactocin and Protolichesterinic Acid. Org. Lett..

[48] Sarabia FJ, Li Q, Ferreira EM (2018). Cyclopentene Annulations of Alkene Radical Cations with Vinyl Diazo Species Using Photocatalysis. Angew. Chem. Int. Ed..

[49] Ganley JM, Murray PRD, Knowles RR (2020). Photocatalytic Generation of Aminium Radical Cations for C-N Bond Formation. ACS Catal..

[50] Masanobu K, Hirochika S, Katsumi T (1981). Electrochemical Oxidation of Aromatic Olefins. Dependence of the Reaction Course on the Structure of the Olefins and on the Nature of the Anode. Chem. Lett..

[51] Jiang H, Studer A (2019). Transition-Metal-Free Three-Component Radical 1,2-Amidoalkynylation of Unactivated Alkenes. Chem. Eur. J..

[52] An X-D, Jiao Y-Y, Zhang H, Gao Y, Yu S (2018). Photoredox-Induced Radical Relay toward Functionalized β-Amino Alcohol Derivatives. Org. Lett..

[53] An X-D, Yu S (2018). Photoredox-Catalyzed Radical Relay Reaction Toward Functionalized Vicinal Diamines. Synthesis.

[54] Jiang H, Seidler G, Studer A (2019). Carboamination of Unactivated Alkenes through Three-Component Radical Conjugate Addition. Angew. Chem. Int. Ed..

[55] Jiang H, Studer A (2020). Intermolecular Radical Carboamination of Alkenes. Chem. Soc. Rev..

[56] McManus JB, Onuska NPR, Nicewicz DA (2018). Generation and Alkylation of α-Carbamyl Radicals via Organic Photoredox Catalysis. J. Am. Chem. Soc..

