# Supplementary material for: Organophotocatalysed synthesis of 2-piperidinones in one step via [1 + 2 + 3] strategy
Source: Nat Commun. 2023 Sep 2;14:5339. doi: 10.1038/s41467-023-40197-x (PMC10475035; doi:10.1038/s41467-023-40197-x)
Supplement: Supplementary file 1 — Supplementary Information [file 41467_2023_40197_MOESM1_ESM.pdf]

Supplementary Information

for

Organophotocatalysed synthesis of 2-piperidinones in one step via [1+2+3] strategy

Yi-Dan Du[1,4], Shan Wang[1,2,4], Hai-Wu Du[1], Xiao-Yong Chang[1], Xiao-Yi Chen[1], Yu-Long Li[3] & Wei Shu[1,2,*]

[1]*Shenzhen Grubbs Institute and Department of Chemistry, Southern University of Science and Technology, Shenzhen 518055, Guangdong, P. R. China.*

[2]*State Key Laboratory of Elemento-Organic Chemistry, Nankai University, Tianjin 300071, P. R. China.*

[3]*College of Chemistry and Environmental Engineering, Sichuan University of Science and Engineering, Zigong, 643000, P. R. China.*

[4]These authors contributed equally: Yi-Dan Du and Shan Wang

*E-mail: shuw@sustech.edu.cn

Table of Contents

Supplementary Note 1 .... **S2**

Supplementary Methods **S3**

Supplementary Tables **S17**

Supplementary Note 2 .... **S23**

- **Characterization of new compounds** **S23**
- **Mechanistic studies** **S55**
- **Scale-up reaction** **S80**
- **X-ray diffraction data** **S81**

Supplementary Note 3: ^{1}H, ^{13}C, and ^{19}F spectra **S90**

Supplementary References **S206**

Supplementary Note 1

General Information

NMR spectra were recorded on 400 MHz or 600 MHz Bruker spectrometers. Chemical shifts are given in ppm. The spectra are calibrated to the residual ^{1}H and ^{13}C signals of the solvents. Multiplicities are abbreviated as follows: singlet (s), doublet (d), triplet (t), quartet (q), doublet-doublet (dd), quintet (quint), septet (sept), multiplet (m), and broad (br). High-resolution electrospray ionization and electronic impact mass spectrometry was performed on a Thermo Scientific Q Exactive mass spectrometer (mass analyzer type: Orbitrap). A mass accuracy $\leq$ 2 ppm was obtained in the peak matching acquisition mode by using a solution containing 2<lPEG200, 2<lPPG450, and 1.5 mg NaOAc (all obtained from Sigma-Aldrich, CH-Buchs) dissolved in 100 mL MeOH (HPLC Supra grade, Scharlau, E-Barcelona) as internal standard.

Materials and Methods: All reactions were carried out under an inert atmosphere of nitrogen in oven dried glassware with magnetic stirring. Unless otherwise noted, commercial reagents were purchased from Energy Chemical Limited, J&K, Adamas-beta®, Aladdin, Macklin Reagent, Bidepharm and used directly without further purification. Chlorobenzene and Benzotrifluoride was purchased in HPLC quality, degassed by purging with nitrogen and dried over activated molecular sieves of appropriate size. 1,4-Dioxane, 1,2-dichloroethane, hexanes, dichloromethane and CH_3CN were distilled over CaH_2 and stored under nitrogen atmosphere. Compounds were visualized by UV light at 254 nm and by dipping the plates in an aqueous potassium permanganate solution followed by heating. Flash column chromatography was performed over silica gel (300-400 mesh).

Supplementary Methods

Supplementary method 1: Typical procedures for the synthesis of starting materials

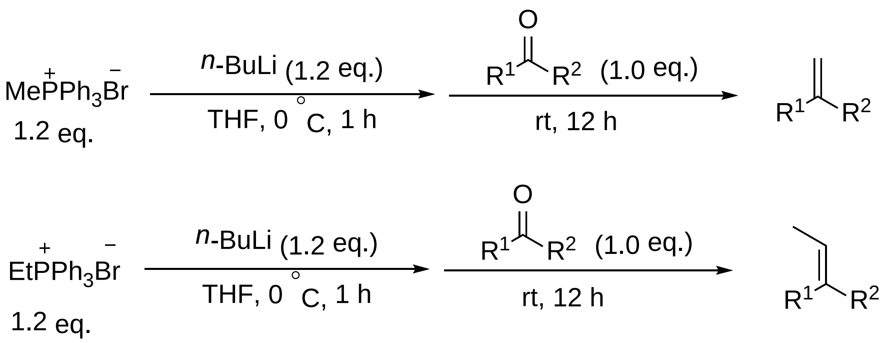

Typical procedure 1: To a suspension of methyltriphenylphosphonium bromide or ethyltriphenylphosphonium bromide (1.2 eq.) in THF (0.2 M) at 0 °C was added *n*-BuLi (1.2 eq.) dropwise. The reaction mixture was stirred at 0 °C for 1 h before corresponding ketone (1.0 eq.) was added dropwise. The resulting mixture was warmed to room temperature and stirred overnight. Upon completion, the reaction mixture was quenched by addition of saturated aqueous ammonium chloride. The organic layer was extracted by diethyl ether (30.0 mL x 3), washed with saturate brine, dried with anhydrous sodium sulfate, and concentrated under vacuum. The final styrenyl derivatives were purified by column chromatography using silica gel with Hexane/ ethyl acetate eluents. The characterization data were in complete agreement with the previously reported values.[1]

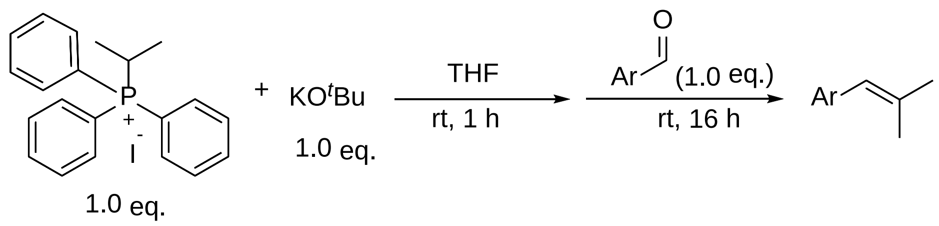

Typical procedure 2: A 100.0 mL round bottom flask was charged with isopropyltriphenylphosphonium iodide (1.0 eq.) and THF (50.0 mL) under a nitrogen atmosphere. Potassium *tert*-butoxide (1.0 eq.) was added under nitrogen pressure, and

the mixture was stirred vigorously at room temperature. After 1 h, aldehyde (1.0 eq.) was added dropwise. The solution continued to stir at room temperature. After 16 h, the reaction was diluted with saturated aqueous ammonium chloride solution (10.0 mL). The layers were separated, and the aqueous phase was extracted with diethyl ether (3 x 20.0 mL). The combined organic extracts were washed with saturated aqueous sodium chloride solution (20.0 mL) and was dried over Na_2SO_4. The solution was filtered and concentrated in vacuo. The crude residue was purified by flash column chromatography to give the products. The characterization data were consistent with the previously reported values.[2]

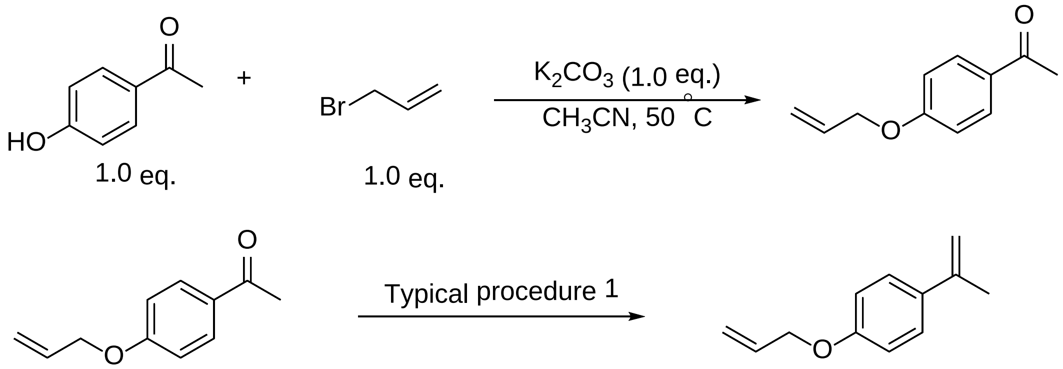

To a solution of 4-hydroxyacetophenone (1.36 g, 10.0 mmol) in CH_3CN (30.0 mL) was added K_2CO_3 (1.38 g, 10.0 mmol) and allyl bromide (0.86 mL, 10.0 mmol). The suspension was stirred at 50 ºC until the starting material was fully consumed, as indicated by TLC (about 3 h). After cooling to r.t., the mixture was filtered through a short pad of Celite. The residue was subsequently washed with ethyl acetate (50.0 mL). After evaporation of the organic solvent, the crude product was obtained and used in the next step without further purification.

To a suspension of *t*BuOK (1.12 g, 10.0 mmol) in anhydrous THF (20.0 mL) was added methyltriphenylphosphonium bromide (3.57 g, 10.0 mmol) under argon atmosphere. The suspension was stirred at room temperature for 1 h, and then the crude product was added. The resulting mixture was warmed to 50 ºC and stirred for 5 h. After cooling to r.t., the mixture was filtered through a short pad of silica gel. The residue was subsequently washed with diethyl ether (200.0 mL). After evaporation of the organic

solvent, the residue was purified by silica gel column chromatography (hexane/ ethyl acetate 50/1 followed by hexane/ ethyl acetate 20/1) to provide the product.[3]

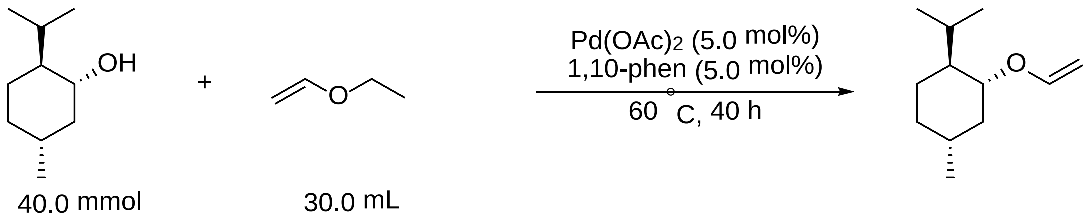

A mixture of $Pd(OAc)_2$ (449.0 mg, 2.0 mmol), 1,10-phenanthroline (360.0 mg, 2.0 mmol) and ethyl vinyl ether (15.0 mL) was stirred at room temperature for 15 min under N_2 atmosphere. To this mixture was added a solution of Menthol (6.25 g, 40.0 mmol) in ethyl vinyl ether (15.0 mL) and the mixture was refluxed at 60 °C for 40 h. The reaction mixture was filtered off and the resulting filtrate was concentrated under reduced pressure. The residue was purified by silica-gel column chromatography using hexane to give the vinyl ether.[4]

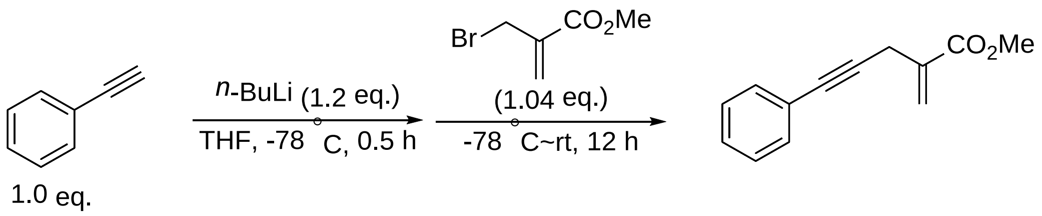

To a suspension of ethynylbenzene (1.02 g, 10.0 mmol) in THF (20.0 mL) at -78 °C was added *n*-BuLi (4.8 mL, 2.5 M in THF, 12.0 mmol) dropwise. The reaction mixture was stirred at -78 °C for 0.5 h before methyl 2-(bromomethyl)acrylate (1.85 g, 10.4 mmol) was added dropwise. The resulting mixture was warmed to room temperature and stirred overnight. Upon completion, the reaction mixture was quenched by addition of saturated aqueous ammonium chloride. The organic layer was extracted by diethyl ether (30.0 mL x 3), washed with saturate brine, dried with anhydrous sodium sulfate, and concentrated under vacuum. The crude residue was purified by flash column chromatography to afford the desired product.[5]

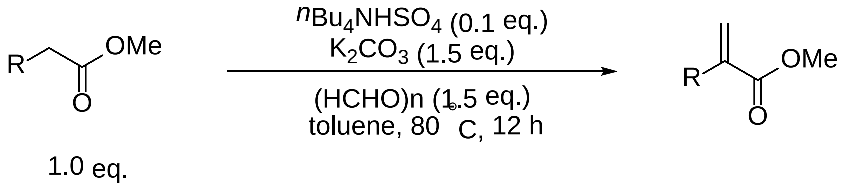

Typical procedure 3: To a solution of ester (1.0 eq.) in anhydrous toluene, K_2CO_3 (1.5 eq.), tetrabutylammonium bisulfate (0.1 eq.), and paraformaldehyde (1.5 eq.) were added. The reaction mixture was heated at 80°C for 12 h. Then the mixture was cooled to room temperature, quenched with H_2O, and extracted with ethyl acetate for three times. The combined organic layer was dried over Na_2SO_4 and concentrated under reduced pressure. The crude residue was purified by flash column chromatography to afford the acrylate.[6]

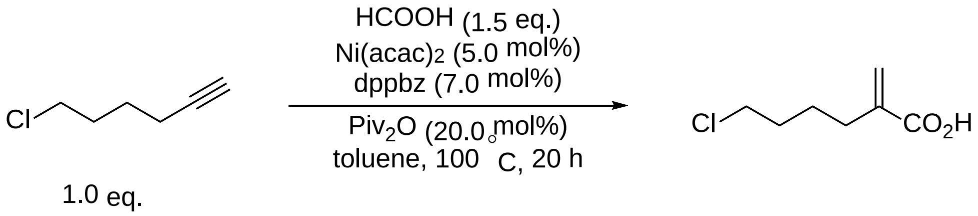

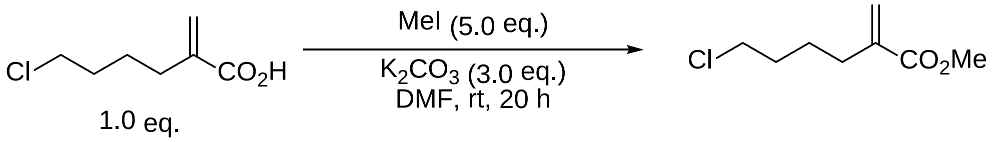

A Schlenk tube containing a magnetic stirring bar was charged with $Ni(acac)_2$ (128.4 mg, 0.5 mmol) and 1,2- bis(diphenylphosphino)benzene (312.2 mg, 0.7 mmol). The tube was evacuated and back-filled with N_2 three times. Toluene (30.0 mL) was added, followed by 6-chlorohex-1-yne (1.17 g, 10.0 mmol), formic acid (0.69 g, 15.0 mmol) and pivalic anhydride (372.5 mg, 2.0 mmol). The reaction mixture was heated to 100 °C for 24 h before cooling to r.t. and concentrating in vacuo. The residue was purified by flash column chromatography (50% ethyl acetate /hexane) to give the corresponding alkenyl carboxylic acid.

To a solution of alkenyl carboxylic acid (1.62g, 10.0 mmol) in DMF (20.0 mL) was added K_2CO_3 (4.2 g, 30.0 mmol) at room temperature. The mixture was cooled to 0 °C before MeI (7.1 g, 50.0 mmol) was added. The resulting mixture was vigorously stirred

at room temperature overnight. H_2O (30.0 mL) was added and the mixture was extracted into CH_2Cl_2 (3 × 30.0 mL). The combined organic layers were washed with H_2O (3 × 50.0 mL), saturated aqueous $NaHCO_3$ (30.0 mL), brine (30.0 mL), dried ($MgSO_4$), filtered, and concentrated in vacuo. The residue was purified by flash column chromatography (10% ethyl acetate /hexane) to give the corresponding methyl ester.[7]

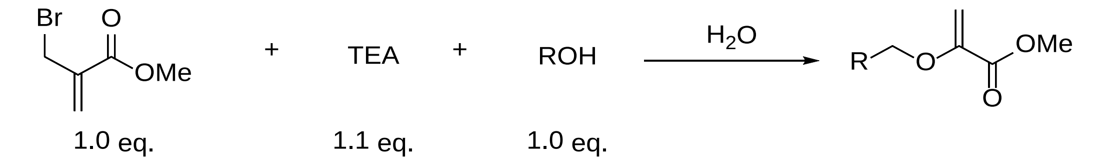

Typical procedure 4: Methyl 2-(bromomethyl)acrylate (0.88 g, 5.0 mmol) was weighed into a glass vial and was added with triethylamine (1.0 g, 5.5 mmol). Alcohol (5.0 mmol) with 2.0 mL of water was added to the mixture. The reaction was vigorously stirred at room temperature for 6 hours. DCM and water were used to extract the reaction mixture. Organic layers were combined and dried over anhydrous sodium sulfate. The solution was dried after filtration and then subjected to flash chromatography to afford the corresponding methyl ester.[8]

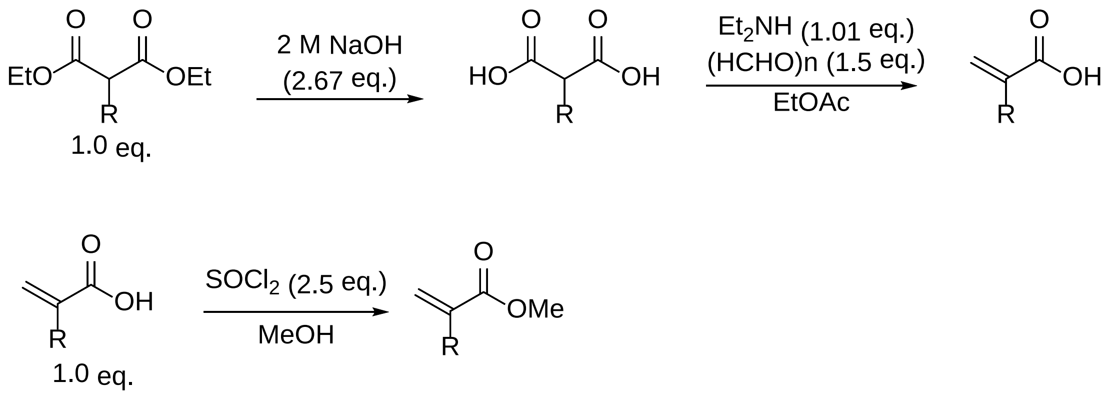

Typical procedure 5: To the substituted malonate (1.0 eq.) was added 2 M NaOH (2.67 eq.), and the resulting mixture was stirred vigorously and refluxed for 2 h. The resulting solution was cooled to rt and extracted with hexane. The aqueous layer was then acidified to PH = 1 with aq HCl. The resulting solution was then extracted with ethyl acetate for 3 times, the combined organic layers were washed with brine, dried ($MgSO_4$) and the volatiles removed in vacuo to afford the corresponding diacid, which could be used directly in the next step. The crude diacid was dissolved in ethyl acetate (0.75 M) and the resulting solution was cooled to 0 °C, followed by the dropwise addition of

diethylamine (1.01 eq.) and subsequent addition of paraformaldehyde (1.5 eq.). The resulting suspension was refluxed for 2 hours and then the reaction mixture was cooled to 0 °C, diluted with H_2O (0.6 mL/mmol diacid) and acidified to pH 1 with concentrated HCl. The aqueous layer was then extracted with ethyl acetate for 3 times and the combined organic layers were washed with brine, dried ($MgSO_4$) and the volatiles removed in vacuo to afford the crude acid, which was purified by flash chromatography (petroleum ether/Et_2O).

To a solution of MeOH (20.0 mL) at 0 °C was added thionyl chloride (2.5 eq.) dropwise followed by acid (1.0 eq.). The reaction mixture was refluxed for 2 h, then cooled to 0 °C, diluted with pentane (40.0 mL), and aq K_2CO_3 was added until pH to 9-10. The aqueous layer was then extracted with pentane (2 x 30.0 mL), the combined organic layers were washed with brine, dried ($MgSO_4$) and the volatiles were removed under a stream of nitrogen to afford the crude product, which was purified by flash chromatography to give the desired product.[9]

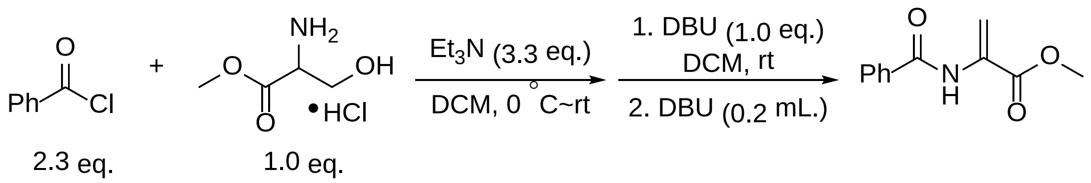

To a solution of serine methyl ester hydrochloride (0.8 g, 5.1 mmol) in CH_2Cl_2 (15.0 mL) was added Et_3N (1.7 g, 17.0 mmol) and benzoyl chloride (1.7 g, 11.8 mmol) in portions at 0 °C. The mixture was stirred for 18 h at room temperature under argon atmosphere. Then the reaction mixture was washed with saturated aqueous $NaHCO_3$, dried over Na_2SO_4, and evaporated to give the residue as a yellow oil. The residue was dissolved in CH_2Cl_2 (15.0 mL), and DBU (0.78 g, 5.1 mmol) was added dropwise at 0 °C. The mixture was stirred for 13 h at room temperature. Another portion of DBU (0.2 mL) was added until the starting material disappeared. Then the reaction mixture was washed with water and saturated aqueous $NaHCO_3$, dried over Na_2SO_4, evaporated and then purified by flash chromatography with n-hexane/ ethyl acetate (5/1 v/v) to give the desired product.[10]

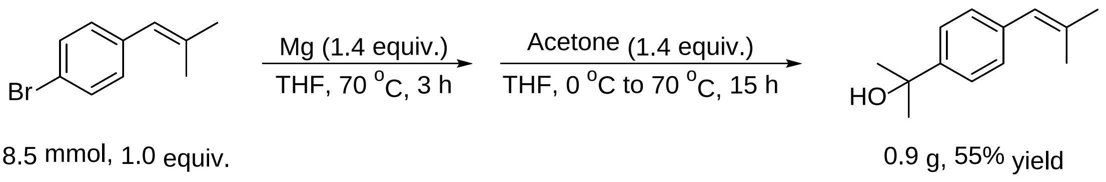

Under nitrogen, the Grignard reagent was prepared by adding slowly 1-bromo-4-(2-methylprop-1-en-1-yl)benzene (1.8 g, 8.5 mmol) to magnesium (289 mg, 11.9 mmol) and a small amount of I_2 in THF(10 ml) at 70 °C for 1h, followed by allowing to react at 70°C for additional 2 h. The Grignard reagent thus prepared was cooled to 0°C and acetone (691 mg, 11.9 mmol) was added dropwise and the reaction mixture was allowed to react at 25°C for additional 15 h. The reaction mixture was then neutralized with H_2O, extracted with ether, washed with brine, and dried over Na_2SO_4. The solvent was removed under reduced pressure. The resultant crude was purified by flash column chromatography (silica gel, hexanes:EtOAc, 10:1) to yield 0.9 g (55% yield) of the title compound.

1-bromo-4-(2-methylprop-1-en-1-yl)benzene (SM1)

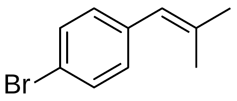

Typical procedure 2 was followed. Colorless oil, 2.0 g, 95% yield. The characterization data were in complete agreement with the previously reported values.[1]

^{1}H NMR (400 MHz, $CDCl_3$) δ 7.42 (d, *J* = 7.6 Hz, 2H), 7.08 (d, *J* = 7.6 Hz, 2H), 6.19 (s, 1H), 1.89 (s, 3H), 1.83 (s, 3H).

1-(allyloxy)-4-(prop-1-en-2-yl)benzene (SM2)

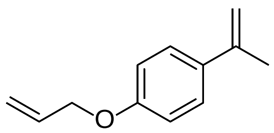

Colorless oil, 1.4 g, 85% yield over 2 steps. The characterization data were in complete agreement with the previously reported values.[3]

^{1}H NMR (400 MHz, $CDCl_3$) δ 7.41 (d, *J* = 8.4 Hz, 2H), 6.88 (d, *J* = 8.4 Hz, 2H), 6.11 – 6.01 (m, 1H), 5.42 (dd, *J* = 17.2, 1.6 Hz, 1H), 5.35 – 5.23 (m, 2H), 4.99 (s, 1H), 4.55 (d, *J* = 5.2 Hz, 2H), 2.13 (s, 3H).

1-((3-methylbut-2-en-1-yl)oxy)-4-(prop-1-en-2-yl)benzene (SM 3)

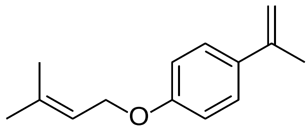

White solid, 1.2 g, 60% yield over 2 steps. The characterization data were in complete agreement with the previously reported values.[3]

^{1}H NMR (400 MHz, $CDCl_3$) δ 7.41 (d, *J* = 8.8 Hz, 2H), 6.88 (d, *J* = 8.8 Hz, 2H), 5.52 – 5.48 (m, 1H), 5.29 – 5.28 (m, 1H), 5.00 – 4.98 (m, 1H), 4.52 (d, *J* = 6.4 Hz, 2H), 2.13 (s, 3H), 1.80 (s, 3H), 1.75 (s, 3H).

(1*S*,4*R*)-1-isopropyl-4-methyl-2-(vinyloxy)cyclohexane (SM4)

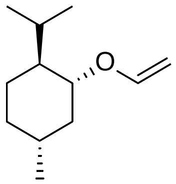

Colorless oil, 4.6 g, 63% yield. The characterization data were in complete agreement with the previously reported values.[4]

^{1}H NMR (600 MHz, $CDCl_3$) δ 6.32 (dd, *J* = 14.0, 6.6 Hz, 1H), 4.28 (dd, *J* = 14.0, 1.4 Hz, 1H), 3.94 (dd, *J* = 6.6, 1.4 Hz, 1H), 3.52 (td, *J* = 10.7, 4.3 Hz, 1H), 2.15 – 2.02 (m, 2H), 1.70 – 1.63 (m, 2H), 1.44 – 1.37 (m, 1H), 1.36 – 1.30 (m, 1H), 1.05 – 0.95 (m, 2H), 0.92 (d, *J* = 6.6 Hz, 3H), 0.90 (d, *J* = 7.0 Hz, 3H), 0.87 – 0.85 (m, 1H), 0.77 (d, *J* = 7.0 Hz, 3H).

methyl 2-methylene-5-phenylpent-4-ynoate (SM5)

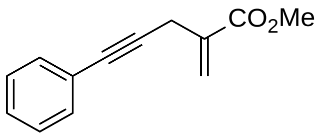

Yellow solid, 0.8 g, 75% yield.

¹H NMR (400 MHz, $CDCl_3$) δ 7.46 – 7.42 (m, 2H), 7.31 – 7.29 (m, 3H), 6.38 (s, 1H), 6.12 (s, 1H), 3.80 (s, 3H), 3.47 (s, 2H).

¹³C NMR (151 MHz, $CDCl_3$) δ 166.8, 135.6, 131.8, 128.4, 128.1, 126.5, 123.5, 85.7, 84.4, 52.2, 22.6.

HR-MS (ESI) calcd for $C_{13}H_{12}O_2^+$ $[M+H]^+$ 201.0910, found 201.0910.

methyl 2-(4-fluorophenyl)acrylate (SM6)

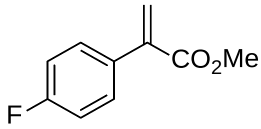

Typical procedure 3 was followed. Colorless oil, 1.6 g, 92% yield. The characterization data were in complete agreement with the previously reported values.[11]

¹H NMR (400 MHz, $CDCl_3$) δ 7.42 – 7.37 (m, 2H), 7.04 (t, *J* = 8.8 Hz, 2H), 6.36 (s, 1H), 5.87 (s, 1H), 3.82 (s, 3H).

methyl 2-(4-methoxyphenyl)acrylate (SM7)

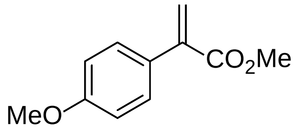

Typical procedure 3 was followed. Colorless oil, 1.7 g, 91% yield. The characterization data were in complete agreement with the previously reported values.[11]

¹H NMR (400 MHz, $CDCl_3$) δ 7.36 (d, *J* = 8.8 Hz, 2H), 6.89 (d, *J* = 8.8 Hz, 2H), 6.27 (s, 1H), 5.84 (s, 1H), 3.82 (s, 6H).

methyl 2-(4-(trifluoromethyl)phenyl)acrylate (SM8)

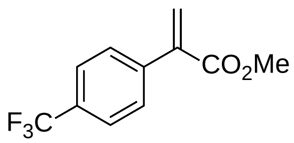

Typical procedure 3 was followed. Colorless oil, 1.9 g, 84% yield. The characterization data were in complete agreement with the previously reported values.[12]

^{1}H NMR (400 MHz, $CDCl_3$) δ 7.69 – 7.59 (m, 3H), 7.48 (t, *J* = 7.8 Hz, 1H), 6.47 (s, 1H), 5.97 (s, 1H), 3.84 (s, 3H).

methyl 2-(3-methoxyphenyl)acrylate (SM9)

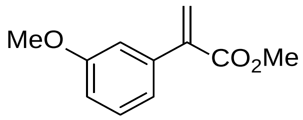

Typical procedure 3 was followed. Colorless oil, 1.6 g, 90% yield. The characterization data were in complete agreement with the previously reported values.[12]

^{1}H NMR (400 MHz, $CDCl_3$) δ 7.29 – 7.25 (m, 1H), 7.02 – 6.94 (m, 2H), 6.90 – 6.87 (m, 1H), 6.36 (d, *J* = 1.2 Hz, 1H), 5.90 (d, *J* = 1.2 Hz, 1H), 3.82 (s, 6H).

methyl 6-chloro-2-methylenehexanoate (SM10)

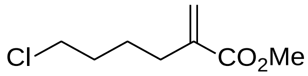

Colorless oil, 1.3 g, 75% yield. The characterization data were in complete agreement with the previously reported values.[7]

^{1}H NMR (400 MHz, $CDCl_3$) δ 6.16 (s, 1H), 5.56 (s, 1H), 3.76 (s, 3H), 3.55 (t, *J* = 6.4 Hz, 2H), 2.39 – 2.30 (m, 2H), 1.84 – 1.77 (m, 2H), 1.67 – 1.59 (m, 2H).

methyl 2-((benzyloxy)methyl)acrylate (SM11)

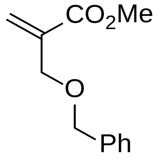

Typical procedure 4 was followed. Colorless oil, 1.1 g, 55% yield. The characterization data were in complete agreement with the previously reported values.[8]

^{1}H NMR (400 MHz, $CDCl_3$) δ 7.38 – 7.27 (m, 5H), 6.33 (q, *J* = 1.2 Hz, 1H), 5.94 (q, *J* = 1.6 Hz, 1H), 4.59 (s, 2H), 4.24 (t, *J* = 1.6 Hz, 2H), 3.77 (s, 3H).

methyl 2-((tetrahydrofuran-2-yl)methyl)acrylate (SM12)

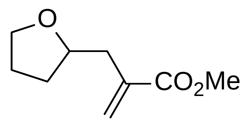

Typical procedure 4 was followed. Colorless oil, 1.0 g, 60% yield. The characterization data were in complete agreement with the previously reported values.[8]

^{1}H NMR (600 MHz, $CDCl_3$) δ 6.22 (s, 1H), 5.67 (s, 1H), 4.05 – 4.00 (m, 1H), 3.88 – 3.85 (m, 1H), 3.75 (s, 3H), 3.74 – 3.68 (m, 1H), 2.51 (d, *J* = 7.2, 2H), 2.00 – 1.96 (m, 1H), 1.91 – 1.84 (m, 2H), 1.54 – 1.48 (m, 1H).

methyl 2-methylenepent-4-enoate (SM13)

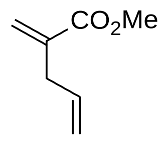

Typical procedure 5 was followed. Colorless oil, 1.0 g, 82% yield. The characterization data were in complete agreement with the previously reported values.[9]

^{1}H NMR (600 MHz, $CDCl_3$) δ 6.20 (s, 1H), 5.89 – 5.78 (m, 1H), 5.58 (q, *J* = 1.2 Hz, 1H), 5.14 – 5.04 (m, 2H), 3.76 (s, 3H), 3.07 – 3.05 (m, 2H).

methyl (*E*)-2-methylene-5-phenylpent-4-enoate (SM14)

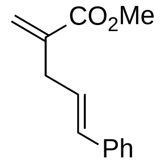

Typical procedure 5 was followed. Colorless oil, 1.2 g, 60% yield. The characterization data were in complete agreement with the previously reported values.[9]

^{1}H NMR (600 MHz, $CDCl_3$) δ 7.36 (d, *J* = 7.8 Hz, 2H), 7.30 (t, *J* = 7.8 Hz, 2H), 7.21 (t, *J* = 7.3 Hz, 1H), 6.45 (d, *J* = 15.8 Hz, 1H), 6.27 – 6.20 (m, 2H), 5.64 (d, *J* = 1.5 Hz, 1H), 3.78 (s, 3H), 3.22 (d, *J* = 6.5 Hz, 2H).

methyl 2-benzamidoacrylate (SM15)

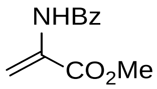

Colorless oil, 1.8 g, 90% yield. The characterization data were in complete agreement with the previously reported values.[10]

^{1}H NMR (600 MHz, $CDCl_3$ δ 8.54 (s, 1H), 7.84 (d, *J* = 7.2 Hz, 2H), 7.55 (t, *J* = 7.2 Hz, 1H), 7.48 (t, *J* = 7.8 Hz, 2H), 6.80 (s, 1H), 6.00 (d, *J* = 1.4 Hz, 1H), 3.90 (s, 3H).

2-(4-(2-methylprop-1-en-1-yl)phenyl)propan-2-ol (SM 16)

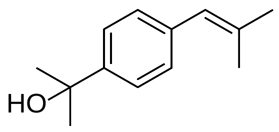

^{1}H NMR (400 MHz, $CDCl_3$) δ 7.44 (d, *J* = 8.4 Hz, 3H), 7.21 (d, *J* = 8.4 Hz, 4H), 6.26 (s, 1H), 1.91 (s, 3H), 1.88 (s, 3H), 1.59 (s, 6H); **^{13}C NMR (101 MHz, $CDCl_3$)** δ 146.6, 137.3, 135.6, 128.7, 124.8, 124.2, 72.6, 31.8, 27.0, 19.6; **HR-MS (ESI)** calcd for $C_{13}H_{19}O^+$ $[M+H]^+$ 191.1430 found 191.1430.

Supplementary method 2: General procedures for the photocatalytic selective [1+2+3] construction of piperidinones from alkenes

General procedure A: Under an inert atmosphere, an oven-dried Schlenk-tube equipped with a magnetic stir bar was charged with *N*-Ph-9-mesityl 3,6-di-*tert*-butylacridinium tetrafluoroborate (1.4 mg, 2.5 μmol, 2.5 mol%), NH_4OAc (23.1 mg, 0.3 mmol), $LiBF_4$ (9.4 mg, 0.1 mmol) and alkene (if solid, 0.1 mmol), CH_3CN (1.0 mL),

alkene (if liquid, 0.1 mmol), acceptor (0.2 mmol) and PhCl (0.1 mL) were added consecutively via syringe. The tube was sealed with a Teflon-coated septum cap, and stirred at ambient temperature under irradiation with 30W blue LEDs for 12 or 24 h. Upon completion, the reaction mixture was quenched with water and extracted with ethyl acetate. The combined organic phase was concentrated in vacumn. The crude mixture was analyzed by ^{1}H NMR with PhTMS as an internal standard to determine the conversion and was directly purified by column chromatography on silica gel with hexane/ ethyl acetate (50/50) to hexane/ ethyl acetate /MeOH (50/50/5) as eluent to give the corresponding piperidinone in pure form.

General procedure B: Under an inert atmosphere, an oven-dried Schlenk-tube equipped with a magnetic stir bar was charged with *N*-Ph-9-mesityl 3,6-di-*tert*-butylacridinium tetrafluoroborate (2.9 mg, 5 μmol, 5 mol%), NH_4OAc (23.1 mg, 0.3 mmol), $LiBF_4$ (9.4 mg, 0.1 mmol) and alkene (if solid, 0.1 mmol), CH_3CN (1.0 mL), alkene (if liquid, 0.1 mmol), acceptor (0.2 mmol) and PhCl (0.1 mL) were added consecutively via syringe. The tube was sealed with a Teflon-coated septum cap, and stirred at ambient temperature under irradiation with 30W blue LEDs for 24 h. Upon completion, the reaction mixture was quenched with water and extracted with ethyl acetate. The combined organic phase was concentrated in vacumn. The crude mixture was analyzed by ^{1}H NMR with PhTMS as an internal standard to determine the conversion and was directly purified by column chromatography on silica gel with hexane/ ethyl acetate (50/50) to hexane/ ethyl acetate /MeOH (50/50/5) as eluent to give the corresponding piperidinone in pure form.

General procedure C: Under an inert atmosphere, an oven-dried Schlenk-tube equipped with a magnetic stir bar was charged with *N*-Ph-9-mesityl 3,6-di-*tert*-butylacridinium tetrafluoroborate (2.9 mg, 5 μmol, 5 mol%), NH_4OAc (23.1 mg, 0.3 mmol), $LiBF_4$ (9.4 mg, 0.1 mmol) and alkene (if solid, 0.1 mmol), CH_3CN (10.0 mL), alkene (if liquid, 0.1 mmol), acceptor (0.2 mmol) and PhCl (0.1 mL) were added consecutively via syringe. The tube was sealed with a Teflon-coated septum cap, and

stirred at ambient temperature under irradiation with 30W blue LEDs for 24 h. Upon completion, the reaction mixture was quenched with water and extracted with ethyl acetate. The combined organic phase was concentrated in vacumn. The crude mixture was analyzed by ^{1}H NMR with PhTMS as an internal standard to determine the conversion and was directly purified by column chromatography on silica gel with hexane/ ethyl acetate (50/50) to hexane/ ethyl acetate /MeOH (50/50/5) as eluent to give the corresponding piperidinone in pure form.

Supplementary method 3: General procedure for conditions evaluation

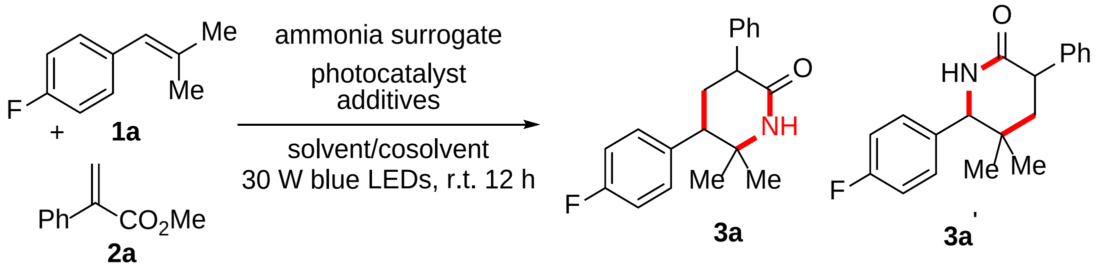

Procedure: Under an inert atmosphere, an oven-dried Schlenk-tube equipped with a magnetic stir bar was charged with photocatalyst, ammonia surrogate and additives. Then solvent, **1a** (0.1 mmol), **2a** and co-solvent was added consecutively via syringe. The tube was sealed with a Teflon-coated septum cap, and stirred at ambient temperature under irradiation with 30W blue LEDs for 12 h. Upon completion, the reaction mixture was diluted with H_2O and extracted with ethyl acetate. The combined organic phase was concentrated in vacumn. The crude mixture was analyzed by ^{1}H NMR with PhTMS as an internal standard to determine the conversion and yield.

Supplementary Tables

Supplementary Table 1. Screening of the ratio of substrates

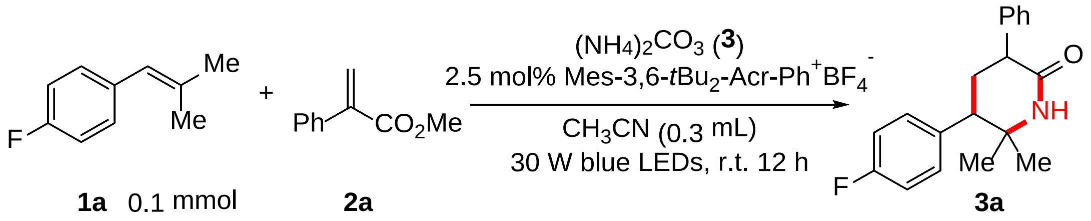

Entry	**1a:2a:3**	**1a** conv.[a]	**2a** conv.[a]	yield of **3a** (dr)[a]	**3a'**[a]
1	1:3:5	>99%	76%	25% (1:1.5)	6%
2	1:2:5	93%	81%	22% (1:1.4)	6%
3	1:2:2	85%	80%	22 % (1:1.4)	5%
**4**	**1:2:3**	**88%**	**80%**	**23% (1:1.4)**	**4%**
5	1:2:4	81%	65%	21% (1:1.4)	6%
6[b]	1:2:5	>99%	83%	28% (1:1.2)	6%

[a]Conversion, yield and diastereoisomeric ratio (dr) were determined by ^{1}H NMR analysis of the crude mixture using PhTMS as internal standard. [b]CH_3CN/PhCl (2:1) was used as solvent.

Supplementary Table 2. Screening of cosolvent

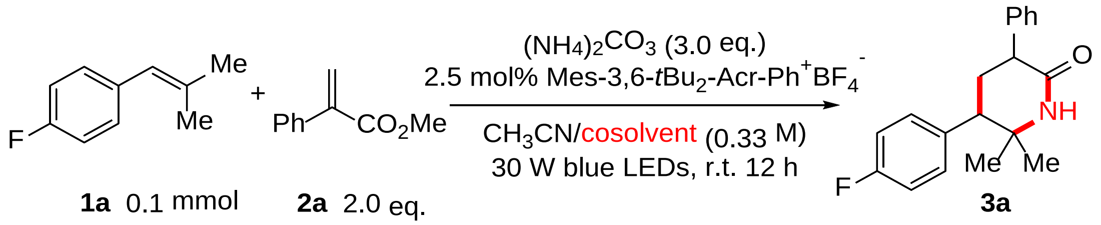

Entry	cosolvent	**1a** conv.[a]	**2a** conv.[a]	yield of **3a** (dr)[a]	**3a'**[a]
1	DCE	90%	76%	25% (1:1.4)	6%
2	DCM	95%	85%	25% (1:1.2)	6%
3	Dioxane	70%	76%	28% (1:1.2)	6%
4	Hexane	83%	67%	19% (1:1.2)	6%

**5**	**PhCl**	**>99%**	**85%**	**34% (1:1.1)**	**6%**
6	$PhCF_3$	83%	70%	22% (1:1.3)	5%

[a]Conversion, yield and diastereoisomeric ratio (dr) were determined by ^{1}H NMR analysis of the crude mixture using PhTMS as internal standard.

Supplementary Table 3. Screening of ammonia surrogate

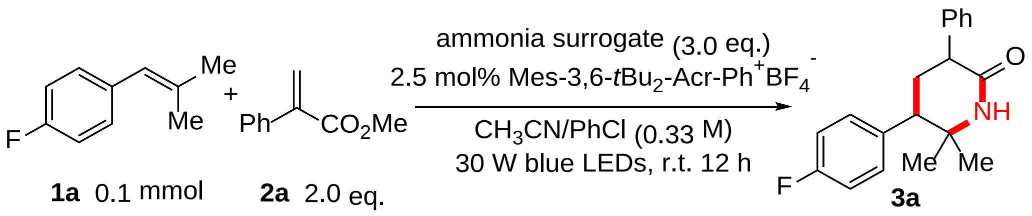

Entry	NH_4X	**1a** conv.[a]	**2a** conv.[a]	yield of **3a** (dr)[a]	**3a'**[a]
1	$(NH_4)_2CO_3$	>99%	85%	34% (1:1.1)	6%
2	NH_4HCO_3	92%	76%	29% (1:13)	5%
3	$NH_2CO_2NH_4$	93%	71%	28% (1:1.4)	4%
**4**	**NH_4OAc**	**>99%**	**82%**	**56% (1.8:1)**	**4%**
5	HCO_2NH_4	85%	50%	28% (1.2:1)	3%
6	NH_4Cl	66%	52%	trace	
7	NH_4BF_4	63%	43%	trace	
8	NH_4PF_6	70%	45%	trace	
9	$(NH_4)_2HPO_4$	40%	37%	trace	

[a]Conversion, yield and diastereoisomeric ratio (dr) were determined by ^{1}H NMR analysis of the crude mixture using PhTMS as internal standard.

Supplementary Table 4. Screening of photocatalysts

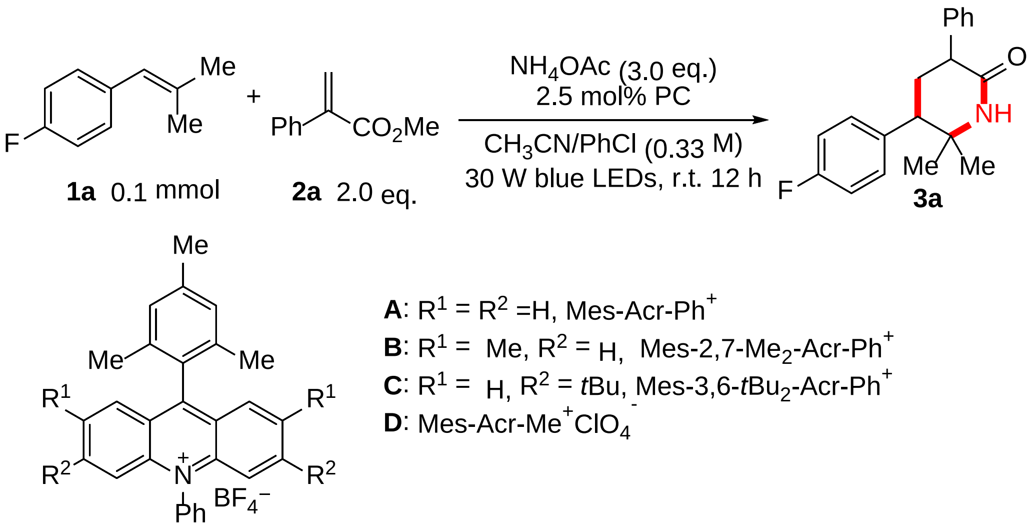

Entry	PC	**1a** conv.[a]	**2a** conv.[a]	yield of **3a** (dr)[a]	**3a'**[a]
**1**	**C**	**>99%**	**82%**	**56% (1.8:1)**	**4%**
2	**A**	40%	21%	35%	2%
3	**B**	95%	50%	50%	4%
4	**D**	50%	26%	40%	2%
**5[b]**	**C**	**>99%**	**80%**	**62% (1.8:1)**	**4%**

[a]Conversion, yield and diastereoisomeric ratio (dr) were determined by ^{1}H NMR analysis of the crude mixture using PhTMS as internal standard. [b]5 mol% **C** was used.

Supplementary Table 5. Screening of the loading of photocatalyst and the concentration of solvents

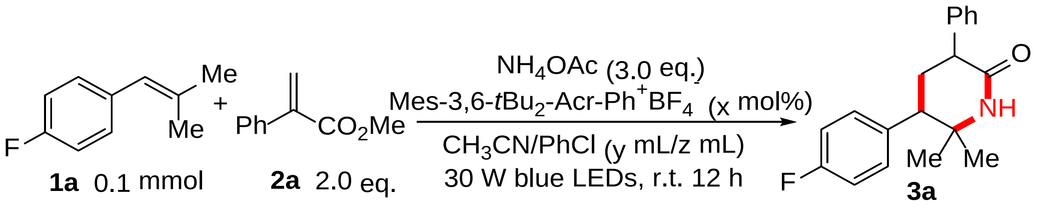

Entry	x	y : z	**1a** conv.[a]	**2a** conv.[a]	yield of **3a** (dr)[a]	**3a'**[a]
1	1.0	0.2 : 0.1	>99%	83%	46% (1.5:1)	4%
2	1.0	0.25 : 0.05	>99%	78%	50% (1.6:1)	4%
3	1.0	0.5 : 0.1	>99%	78%	70% (2.6;1)	4%
**4**	**1.0**	**1.0 : 0.1**	**>99%**	**77%**	**75% (1.9:1)**	**5%**
5	1.0	2.0 : 0.1	>99%	82%	67% (1.9:1)	4%
6	2.5	0.2 : 0.1	>99%	82%	55% (1.8:1)	4%
7	2.5	0.2 : 0.1	>99%	84%	46% (2.3:1)	4%
8	2.5	0.15 : 0.15	>99%	72%	69% (2.9;1)	4%
9	2.5	0.3 : 0.1	>99%	87%	74% (2.4:1)	4%
10	2.5	0.4 : 0.1	>99%	85%	75% (2.7;1)	4%
**11**	**2.5**	**0.5 : 0.1**	**>99%**	**82%**	**87% (2.4:1)**	**4%**
12	2.5	0.25 : 0.05	>99%	89%	67% (2.3:1)	4%
**13**	**2.5**	**1.0 : 0.1**	**>99%**	**76%**	**84% (1.8:1)**	**4%**
14	2.5	2.0 : 0.1	>99%	74%	71% (1.7:1)	5%
15	5.0	0.2 : 0.1	>99%	80%	62% (1.8:1)	4%

[a]Conversion, yield and diastereoisomeric ratio (dr) were determined by ^{1}H NMR analysis of the crude mixture using PhTMS as internal standard.

Supplementary Table 6. Confirmation the optimal concentration of solvents

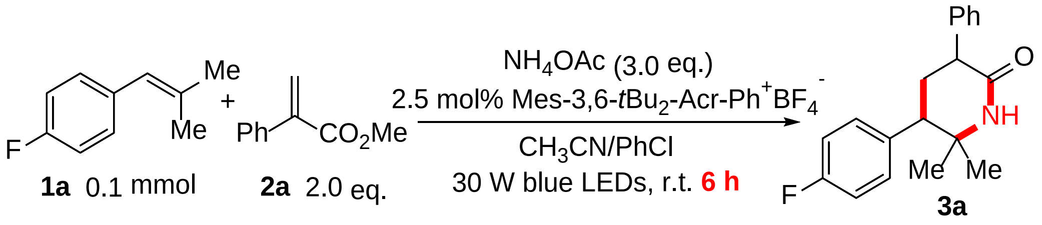

Entry	CH3CN/PhCl (mL:mL)	**1a** conv.[a]	**2a** conv.[a]	yield of **3a** (dr)[a]	**3a'**[a]
1	0.25:0.05	58%	28%	38% (2.4:1)	3%
2	0.5:0.1	39%	23%	36% (1.7:1)	2%
3	0.3:0.03	95%	54%	44% (1.4:1)	4%
**4**	**1.0:0.1**	**62%**	**38%**	**62% (1.9:1)**	**3%**

[a]Conversion, yield and diastereoisomeric ratio (dr) were determined by ^{1}H NMR analysis of the crude mixture using PhTMS as internal standard.

Supplementary Table 7. Further optimization of concentration of solvents

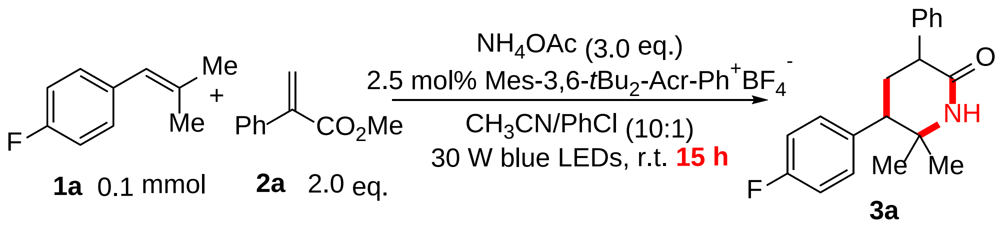

Entry	*c* (M) (mL:mL)	**1a** conv.[a]	**2a** conv.[a]	yield of **3a** (dr)[a]	**3a'**[a]
1	0.3 (0.3:0.03)	>99%	73%	54% (2.6:1)	4%
2	0.23 (0.4:0.04)	>99%	85%	74% (2.9:1)	4%
3	0.18 (0.5:0.05)	>99%	69%	67% (2.7:1)	4%
**4**	**0.09 (1.0:0.1)**	**>99%**	**75%**	**80% (2.6:1)**	**4%**
5	0.045 (2.0:0.2)	>99%	85%	73% (2.2:1)	5%
6[b]	0.1	>99%	76%	54% (2.4:1)	4%

[a]Conversion, yield and diastereoisomeric ratio (dr) were determined by ^{1}H NMR analysis of the crude mixture using PhTMS as internal standard. [b]CH3CN as solvent.

Supplementary Table 8. Screening of additives

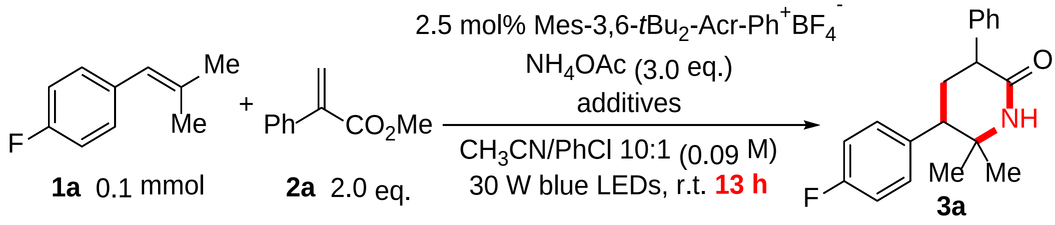

Entry	additives (eq.)	**1a** conv.[a]	**2a** conv.[a]	yield of **3a** (dr)[a]	**3a'**[a]
1	$NaBF_4$ (0.2)	>99%	77%	70% (3.4:1)	4%
2	$LiBF_4$ (0.2)	>99%	73%	73% (3.6:1)	5%
3	$Sc(OTf)_3$ (0.2)	81%	41%	56% (3.3:1)	3%
4	$Zn(OTf)_2$ (0.2)	97%	55%	73% (3.7:1)	3%
5	K_2CO_3 (0.2)	>99%	78%	56% (1.9:1)	4%
6	2,6-*t*Bu_2pyridine (0.2)	86%	55%	80% (2.7:1)	4%
7	$LiBF_4$ (0.5)	65%	33%	58% (3.3:1)	3%
**8**	**$LiBF_4$ (1.0)**	**>99%**	**87%**	**88% (3.7:1)**	**4%**
9	LiBF4 (2.0)	56%	27%	45% (3.0:1)	3%
10	2,6-tBu2pyridine (0.5)	>99%	78%	81% (2.7:1)	5%
**11**	**2,6-^{t}Bu_2pyridine (1.0)**	**>99%**	**87%**	**88% (2.7:1)**	**5%**
12	2,6-^{t}Bu_2pyridine (2.0)	>99%	73%	75% (2.4:1)	5%
13	2,6-lutidine (1.0)	>99%	70%	82% (2.5:1)	4%
14	2,4,6-collidine (1.0)	>99%	86%	69% (2.9:1)	4%

[a]Conversion, yield and diastereoisomeric ratio (dr) were determined by ^{1}H NMR analysis of the crude mixture using PhTMS as internal standard.

Supplementary Note 2

Characterization of new compounds

***cis*-5-(4-fluorophenyl)-6,6-dimethyl-3-phenylpiperidin-2-one (3a)**

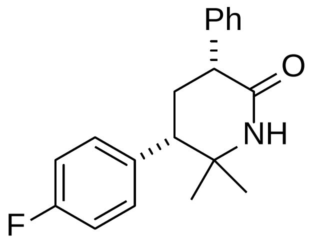

Prepared from 4-fluoro-β,β-dimethylstyrene and methyl 2-phenylacrylate following General procedure A with 24 h irradiation to give **3a** as a white solid in 21.1 mg (71% yield) with 3.7:1 dr. **^{1}H NMR** (600 MHz, $CDCl_3$) δ 7.28 (t, *J* = 7.6 Hz, 2H), 7.20 – 7.18 (m, 3H), 7.12 – 7.09 (m, 2H), 6.92 (t, *J* = 8.6 Hz, 2H), 6.55 (s, 1H), 3.63 (dd, *J* = 12.1, 6.6 Hz, 1H), 2.98 (dd, *J* = 13.4, 2.5 Hz, 1H), 2.48 – 2.41 (m, 1H), 2.06 (ddd, *J* = 13.7, 6.6, 2.5 Hz, 1H), 1.12 (s, 3H), 1.07 (s, 3H); **^{19}F NMR** (564 MHz, $CDCl_3$) -115.36 – -115.41 (m, 1F); **^{13}C NMR** (151 MHz, $CDCl_3$) δ 172.1, 162.5 (d, *J* = 245.8 Hz), 140.7, 135.6 (d, *J* = 3.3 Hz), 130.6 (d, *J* = 7.9 Hz), 128.8, 128.4, 127.0, 115.0 (d, *J* = 21.2 Hz), 56.3, 49.48, 49.46, 34.6, 30.4, 25.3; **HR-MS (ESI)** calcd for $C_{19}H_{21}FNO^+$ $[M+H]^+$ 298.1607 found 298.1599.

***trans*-6-(4-fluorophenyl)-5,5-dimethyl-3-phenylpiperidin-2-one (3a')**

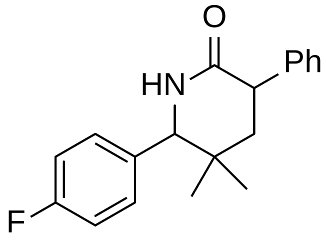

Prepared from 4-fluoro-β,β-dimethylstyrene and methyl 2-phenylacrylate following General procedure A with 24 h irradiation to give **3a'** as a white solid. **^{1}H NMR** (600 MHz, $CDCl_3$) δ 7.38 (t, *J* = 7.6 Hz, 2H), 7.35 – 7.31 (m, 2H), 7.30 – 7.26 (m, 3H), 7.09 (t, *J* = 8.6 Hz, 2H), 6.12 (s, 1H), 4.28 (d, *J* = 4.3 Hz, 1H), 3.76 (dd, *J* = 12.2, 6.8 Hz, 1H), 2.01 – 1.97 (m, 1H), 1.69 – 1.65 (m, 1H), 1.38 (s, 3H), 0.69 (s, 3H); **^{19}F NMR** (564 MHz, $CDCl_3$) δ -114.34 – -114.33 (m, 1F); **^{13}C NMR** (151 MHz, $CDCl_3$) δ 172.7, 162.5 (d, *J* = 247.3 Hz), 140.3, 135.8 (d, *J* = 3.3 Hz), 129.5 (d, *J* = 8.1 Hz), 128.9, 128.6, 127.1, 115.3 (d, *J* = 21.7 Hz), 66.4, 45.6, 39.4, 33.6, 27.7, 27.5; **HR-MS (ESI)** calcd for $C_{19}H_{21}FNO^+$ $[M+H]^+$ 298.1607 found 298.1599.

5,5,6,6-tetramethyl-3-phenylpiperidin-2-one (3b)

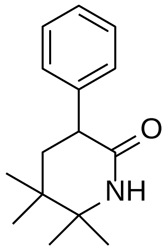

Prepared from 2,3-dimethyl-2-butene and methyl 2-phenylacrylate following General procedure A with 12 h irradiation to give **3b** as a white solid in 23.1 mg (99% yield). **^{1}H NMR** (600 MHz, $CDCl_3$) δ 7.32 (t, *J* = 7.4 Hz, 2H), 7.23 (t, *J* = 7.4 Hz, 1H), 7.21 – 7.18 (m, 2H), 6.07 (s, 1H), 3.62 (dd, *J* = 12.3, 7.3 Hz, 1H), 2.10 (dd, *J* = 14.1, 12.3 Hz, 1H), 1.72 (dd, *J* = 14.1, 7.3 Hz, 1H), 1.35 (s, 3H), 1.17 (s, 3H), 1.16 (s, 3H), 0.98 (s, 3H); **^{13}C NMR** (151 MHz, $CDCl_3$) δ 172.6, 141.5, 128.8, 128.4, 126.8, 58.5, 45.9, 43.1, 35.1, 28.2, 24.89, 24.85, 23.0; **HR-MS (ESI)** calcd for $C_{15}H_{22}NO^+$ $[M+H]^+$ 232.1701 found 232.1692.

3-(4-fluorophenyl)-5,5,6,6-tetramethylpiperidin-2-one (3c)

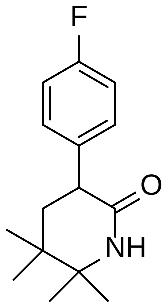

Prepared from 2,3-dimethyl-2-butene and methyl 2-(4-fluorophenyl)acrylate following General procedure A with 12 h irradiation to give **3c** as a white solid in 24.9 mg (99% yield). **^{1}H NMR** (600 MHz, $CDCl_3$) δ 7.18 – 7.14 (m, 2H), 7.01 (t, *J* = 8.6 Hz, 2H), 5.92 (s, 1H), 3.62 (dd, *J* = 12.4, 7.1 Hz, 1H), 2.08 (dd, *J* = 14.1, 12.4 Hz, 1H), 1.72 (dd, *J* = 14.1, 7.1 Hz, 1H), 1.36 (s, 3H), 1.20 (s, 3H), 1.16 (s, 3H), 1.00 (s, 3H); **^{19}F NMR** (564 MHz, $CDCl_3$) δ -116.18 – -116.23 (m, 1F); **^{13}C NMR** (151 MHz, $CDCl_3$) δ 172.4, 161.9 (d, *J* = 245.1 Hz), 136.9 (d, *J* = 3.3 Hz), 130.0 (d, *J* = 8.1 Hz), 115.7 (d, *J* = 21.4 Hz), 58.8, 45.1, 42.9, 35.2, 28.3, 25.0, 24.9, 23.1; **HR-MS (ESI)** calcd for $C_{15}H_{21}FNO^+$ $[M+H]^+$ 250.1607 found 250.1598.

5,5,6,6-tetramethyl-3-(4-(trifluoromethyl)phenyl)piperidin-2-one (3d)

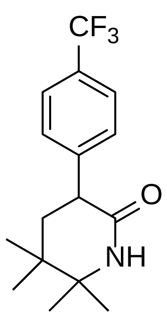

Prepared from 2,3-dimethyl-2-butene and methyl 2-(4-trifluoromethylphenyl)acrylate following General procedure A with 12 h irradiation to give **3d** as a white solid in 27.2 mg (91% yield). **^{1}H NMR** (400 MHz, $CDCl_3$) δ 7.57 (d, *J* = 8.0 Hz, 2H), 7.31 (d, *J* = 8.0 Hz, 2H), 6.17 (s, 1H), 3.70 (dd, *J* = 12.2, 7.2 Hz, 1H), 2.07 (dd, *J* = 14.0, 12.2 Hz, 1H), 1.72 (dd, *J* = 14.0, 7.2 Hz, 1H), 1.34 (s, 3H), 1.18

(s, 3H), 1.16 (s, 3H), 0.99 (s, 3H); **^{19}F NMR** (376 MHz, $CDCl_3$) δ -62.50 (s, 3F); **^{13}C NMR** (101 MHz, $CDCl_3$) δ 171.7, 145.5 (q, *J* = 1.0 Hz), 129.1 (q, *J* = 32.0 Hz) , 128.9, 125.8 (q, *J* = 3.7 Hz), 124.3 (q, *J* = 270.0 Hz), 58.6, 45.8, 42.7, 35.1, 28.2, 24.8, 23.0; **HR-MS (ESI)** calcd for $C_{16}H_{21}F_3NO^+$ $[M+H]^+$ 300.1575 found 300.1565.

3-(4-methoxyphenyl)-5,5,6,6-tetramethylpiperidin-2-one (3e)

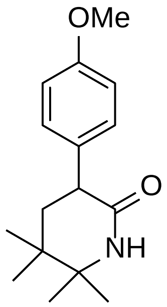

Prepared from 2,3-dimethyl-2-butene and methyl 2-(4-methoxyphenyl)acrylate following General procedure A with 12 h irradiation to give **3e** as a white solid in 26.1 mg (99% yield). **^{1}H NMR** (600 MHz, $CDCl_3$) δ 7.13 – 7.09 (m, 2H), 6.88 – 6.84 (m, 2H), 5.98 (s, 1H), 3.77 (s, 3H), 3.57 (dd, *J* = 12.3, 7.2 Hz, 1H), 2.08 (dd, *J* = 14.1, 12.3 Hz, 1H), 1.70 (dd, *J* = 14.1, 7.2 Hz, 1H), 1.34 (s, 3H), 1.16 (s, 3H), 1.14 (s, 3H), 0.97 (s, 3H); **^{13}C NMR** (151 MHz, $CDCl_3$) δ 172.8, 158.4, 133.5, 129.4, 114.3, 58.5, 55.4, 45.0, 43.0, 35.1, 28.2, 24.88, 24.87, 23.0; **HR-MS (ESI)** calcd for $C_{16}H_{24}NO_2^+$ $[M+H]^+$ 262.1807 found 262.1797.

3-(2-fluorophenyl)-5,5,6,6-tetramethylpiperidin-2-one (3f)

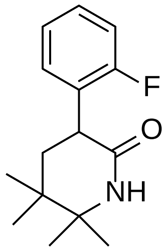

Prepared from 2,3-dimethyl-2-butene and methyl 2-(2-fluorophenyl)acrylate following General procedure A with 12 h irradiation to give **3f** as a white solid in 24.9 mg (99% yield). **^{1}H NMR** (600 MHz, $CDCl_3$) δ 7.24 – 7.17 (m, 2H), 7.09 (t, *J* = 7.5 Hz, 1H), 7.06 – 7.01 (m, 1H), 5.87 (s, 1H), 3.77 (dd, *J* = 12.4, 7.1 Hz, 1H), 2.18 – 2.14 (m, 1H), 1.68 (dd, *J* = 13.9, 7.1 Hz, 1H), 1.35 (s, 3H), 1.18 (s, 3H), 1.17 (s, 3H), 0.98 (s, 3H); **^{19}F NMR** (564 MHz, $CDCl_3$) δ -116.80 (s, 1F); **^{13}C NMR** (151 MHz, $CDCl_3$) δ 171.7, 160.9 (d, *J* = 245.8 Hz), 130.9 (d, *J* = 4.7 Hz), 128.7 (d, *J* = 8.3 Hz), 128.4 (d, *J* = 14.6 Hz), 124.5 (d, *J* = 3.5 Hz), 115.8 (d, *J* = 21.8 Hz), 58.7, 41.2, 41.0, 35.1, 27.6, 25.0, 24.9, 23.0; **HR-MS (ESI)** calcd for $C_{15}H_{21}FNO^+$ $[M+H]^+$ 250.1607 found 250.1597.

3-(2-methoxyphenyl)-5,5,6,6-tetramethylpiperidin-2-one (3g)

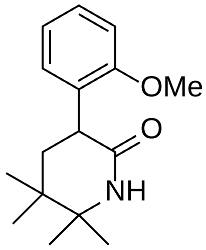

Prepared from 2,3-dimethyl-2-butene and methyl 2-(2-methoxyphenyl)acrylate following General procedure A with 12 h irradiation to give **3g** as a white solid in 19.0 mg (73% yield). **^{1}H NMR** (600 MHz, $CDCl_3$) δ 7.22 (td, *J* = 7.8, 1.7 Hz, 1H), 7.13 (dd, *J* = 7.5, 1.7 Hz, 1H), 6.94 – 6.89 (m, 1H), 6.87 (d, *J* = 8.2 Hz, 1H), 5.69 (s, 1H), 3.80 (s, 3H), 3.72 – 3.60 (m, 1H), 2.25 – 2.21 (m, 1H), 1.59 – 1.55 (m, 1H), 1.37 (s, 3H), 1.17 (s, 3H), 1.16 (s, 3H), 0.96 (s, 3H); **^{13}C NMR** (151 MHz, $CDCl_3$) δ 172.9, 156.9, 129.8, 128.3, 121.1, 111.3, 58.5, 55.3, 40.4, 35.1, 27.2, 25.2, 24.9, 23.1; **HR-MS (ESI)** calcd for $C_{16}H_{24}NO_2^+$ $[M+H]^+$ 262.1807 found 262.1796.

3-(3-methoxyphenyl)-5,5,6,6-tetramethylpiperidin-2-one (3h)

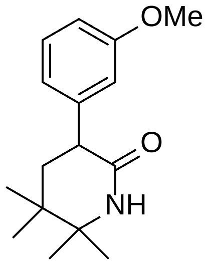

Prepared from 2,3-dimethyl-2-butene and methyl 2-(3-methoxyphenyl)acrylate following General procedure A with 12 h irradiation to give **3h** as a white solid in 26.1 mg (99% yield). **^{1}H NMR** (600 MHz, $CDCl_3$) δ 7.23 (t, *J* = 7.9 Hz, 1H), 6.80 – 6.76 (m, 2H), 6.76 – 6.72 (m, 1H), 6.01 (s, 1H), 3.78 (s, 3H), 3.59 (dd, *J* = 12.3, 7.3 Hz, 1H), 2.10 (dd, *J* = 14.1, 12.3 Hz, 1H), 1.72 (dd, *J* = 14.1, 7.3 Hz, 1H), 1.34 (s, 3H), 1.17 (s, 3H), 1.14 (s, 3H), 0.97 (s, 3H); **^{13}C NMR** (151 MHz, $CDCl_3$) δ 172.4, 159.8, 143.0, 129.8, 120.8, 114.6, 112.1, 58.5, 55.3, 46.0, 43.0, 35.1, 28.2, 24.9, 24.8, 23.0; **HR-MS (ESI)** calcd for for $C_{16}H_{24}NO_2^+$ $[M+H]^+$ 262.1807 found 262.1797.

5,5,6,6-tetramethyl-3-(naphthalen-2-yl)piperidin-2-one (3i)

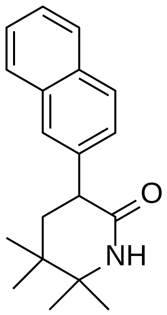

Prepared from 2,3-dimethyl-2-butene and methyl 2-(naphthalen-2-yl)acrylate following General procedure A with 12 h irradiation to give **3i** as a white solid in 28.1 mg (99% yield). **^{1}H NMR** (600 MHz, $CDCl_3$) δ 7.84 – 7.77 (m, 3H), 7.69 (s, 1H), 7.47 – 7.41 (m, 2H), 7.31 (dd, *J* = 8.4, 1.8 Hz, 1H), 6.07 (s, 1H), 3.82 (dd, *J* = 12.3, 7.2 Hz, 1H), 2.20 (dd, *J* = 14.1, 12.3 Hz, 1H), 1.79 (dd, *J* = 14.1, 7.2 Hz, 1H), 1.40 (s, 3H), 1.20 (s, 6H), 1.00 (s, 3H); **^{13}C NMR** (151 MHz, $CDCl_3$) δ 172.5, 138.8, 133.7, 132.5,

128.6, 127.8, 127.7, 127.4, 126.3, 126.1, 125.7, 58.6, 46.1, 42.9, 35.2, 28.3, 24.94, 24.89, 23.1; **HR-MS (ESI)** calcd for $C_{19}H_{24}NO^+$ $[M+H]^+$ 282.1858 found 282.1848.

5,5,6,6-tetramethylpiperidin-2-one (3j)

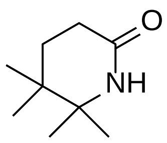

Prepared from 2,3-dimethyl-2-butene and methyl acrylate following General procedure A with 12 h irradiation to give **3j** as a white solid in 7.0 mg (45% yield). **^{1}H NMR** (400 MHz, $CDCl_3$) δ 5.89 (s, 1H), 2.36 (t, *J* = 7.0 Hz, 2H), 1.66 (t, *J* = 7.0 Hz, 2H), 1.17 (s, 6H), 0.99 (s, 6H); **^{13}C NMR** (101 MHz, $CDCl_3$) δ 171.9, 58.2, 34.2, 31.9, 28.2, 26.3, 23.8; **HR-MS (ESI)** calcd for $C_9H_{18}NO^+$ $[M+H]^+$ 156.1388 found 156.1382.

3-fluoro-5,5,6,6-tetramethylpiperidin-2-one (3k)

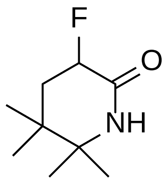

Prepared from 2,3-dimethyl-2-butene and methyl 2-fluoroacrylate following General procedure A with 12 h irradiation to give **3k** as a white solid in 12.1 mg (70% yield). **^{1}H NMR** (400 MHz, $CDCl_3$) δ 6.38 (s, 1H), 4.87 (ddd, *J* = 47.3, 8.8, 7.4 Hz, 1H), 2.10 – 2.03 (m, 2H), 1.27 (s, 3H), 1.17 (s, 3H), 1.06 (s, 3H), 1.04 (d, *J* = 1.5 Hz, 3H); **^{19}F NMR** (376 MHz, $CDCl_3$) δ -189.98 – -190.19 (m, 1F); **^{13}C NMR** (101 MHz, $CDCl_3$) δ 168.0 (d, *J* = 20.0 Hz), 84.8 (d, *J* = 176.4 Hz), 59.0, 38.9 (d, *J* = 17.9 Hz), 35.7 (d, *J* = 7.3 Hz), 27.1, 25.0, 24.9, 23.7; **HR-MS (ESI)** calcd for $C_9H_{17}FNO^+$ $[M+H]^+$ 174.1294 found 174.1287.

3,5,5,6,6-pentamethylpiperidin-2-one (3l)

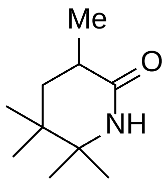

Prepared from 2,3-dimethyl-2-butene and methyl 2-methylacrylate following General procedure A with 12 h irradiation to give **3l** as a white solid in 12.0 mg (71% yield). **^{1}H NMR** (600 MHz, $CDCl_3$) δ 5.61 (s, 1H), 2.47 – 2.38 (m, 1H), 1.67 (dd, *J* = 13.8, 11.6 Hz, 1H), 1.52 (dd, *J* = 13.8, 7.0 Hz, 1H), 1.20 (d, *J* = 7.0 Hz, 3H), 1.20 (s, 3H), 1.11 (s, 3H), 1.03 (s, 3H), 0.95 (s, 3H); **^{13}C NMR** (151 MHz, $CDCl_3$) δ 174.9, 58.4, 41.4, 34.9, 32.8, 28.0, 25.04, 24.98, 23.2, 17.4; **HR-MS (ESI)** calcd for $C_{10}H_{20}NO^+$ $[M+H]^+$ 170.1545 found 170.1537.

3-benzyl-5,5,6,6-tetramethylpiperidin-2-one (3m)

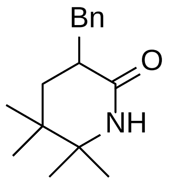

Prepared from 2,3-dimethyl-2-butene and methyl 2-benzylacrylate following General procedure A with 12 h irradiation to give **3m** as a white solid in 19.6 mg (80% yield). **^{1}H NMR** (600 MHz, $CDCl_3$) δ 7.23 – 7.18 (m, 2H), 7.16 – 7.11 (m, 3H), 5.60 (s, 1H), 3.22 (dd, *J* = 13.6, 4.0 Hz, 1H), 2.72 (dd, *J* = 13.6, 9.1 Hz, 1H), 2.57 (tdt, *J* = 11.0, 6.7, 4.0 Hz, 1H), 1.57 (dd, *J* = 14.0, 11.7 Hz, 1H), 1.20 (dd, *J* = 14.0, 7.2 Hz, 1H), 1.03 (s, 3H), 0.96 (s, 3H), 0.90 (s, 3H), 0.81 (s, 3H); **^{13}C NMR** (151 MHz, $CDCl_3$) δ 173.4, 139.7, 129.5, 128.4, 126.3, 58.1, 39.6, 37.6, 37.5, 34.9, 27.8, 25.0, 24.9, 23.0; **HR-MS (ESI)** calcd for $C_{16}H_{24}NO^+$ $[M+H]^+$ 246.1858 found 246.1849.

methyl 2-(5,5,6,6-tetramethyl-2-oxopiperidin-3-yl)acetate (3n)

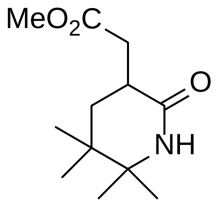

Prepared from 2,3-dimethyl-2-butene and dimethyl itaconate following General procedure A with 12 h irradiation to give **3n** as a white solid in 22.1 mg (97% yield). **^{1}H NMR** (600 MHz, $CDCl_3$) δ 5.82 (s, 1H), 3.66 (s, 3H), 2.75 (ddt, *J* = 12.7, 12.0, 3.5 Hz, 2H), 2.65 – 2.58 (m, 1H), 1.84 (dd, *J* = 13.6, 12.0 Hz, 1H), 1.50 (dd, *J* = 13.6, 6.4 Hz, 1H), 1.23 (s, 3H), 1.11 (s, 3H), 1.05 (s, 3H), 0.94 (s, 3H); **^{13}C NMR** (151 MHz, $CDCl_3$) δ 172.9, 172.6, 58.5, 51.7, 38.4, 35.7, 34.9, 27.7, 25.0, 24.8, 23.1; **HR-MS (ESI)** calcd for $C_{12}H_{22}NO_3^+$ $[M+H]^+$ 228.1600 found 228.1592.

3-allyl-5,5,6,6-tetramethylpiperidin-2-one (3o)

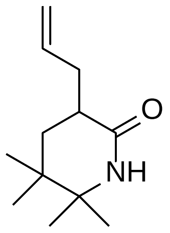

Prepared from 2,3-dimethyl-2-butene and methyl 2-methylenepent-4-enoate following General procedure A with 12 h irradiation to give **3o** as a white solid in 11.9 mg (61% yield). **^{1}H NMR** (600 MHz, $CDCl_3$) δ 5.78 – 5.71 (m, 1H), 5.64 (s, 1H), 5.11 – 5.03 (m, 2H), 2.61 – 2.54 (m, 1H), 2.48 – 2.40 (m, 1H), 2.34 (dddt, *J* = 13.7, 8.4, 7.0, 1.2 Hz, 1H), 1.71 (dd, *J* = 14.0, 11.7 Hz, 1H), 1.44 (dd, *J* = 14.0, 7.1 Hz, 1H), 1.19 (s, 3H), 1.11 (s, 3H), 1.02 (s, 3H), 0.96 (s, 3H); **^{13}C NMR** (151 MHz, $CDCl_3$) δ 173.5, 136.0, 117.3, 58.2, 37.8, 37.4, 36.0, 34.8, 28.0, 25.1, 25.0, 23.2; **HR-MS (ESI)** calcd for $C_{12}H_{22}NO^+$ $[M+H]^+$ 196.1701 found 196.1695.

3-cinnamyl-5,5,6,6-tetramethylpiperidin-2-one (3p)

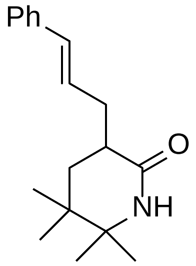

Prepared from 2,3-dimethyl-2-butene and methyl (*E*)-2-methylene-5-phenylpent-4-enoate following General procedure A with 12 h irradiation to give **3p** as a white solid in 15.8 mg (58% yield). **^{1}H NMR** (600 MHz, $CDCl_3$) δ 7.35 (d, *J* = 7.2 Hz, 2H), 7.29 (t, *J* = 7.7 Hz, 2H), 7.20 (t, *J* = 7.2 Hz, 1H), 6.45 (d, *J* = 15.6 Hz, 1H), 6.17 (dt, *J* = 15.6, 7.0 Hz, 1H), 5.69 (s, 1H), 2.72 (ddd, *J* = 14.0, 7.8, 4.1 Hz, 1H), 2.53 (dq, *J* = 10.4, 7.8 Hz, 2H), 1.77 (dd, *J* = 14.0, 10.8 Hz, 1H), 1.48 (dd, *J* = 14.0, 6.5 Hz, 1H), 1.18 (s, 3H), 1.12 (s, 3H), 1.03 (s, 3H), 0.95 (s, 3H); **^{13}C NMR** (151 MHz, $CDCl_3$) δ 173.4, 137.6, 132.5, 128.6, 127.8, 127.2, 126.2, 58.2, 37.91, 37.86, 35.2, 34.9, 28.0, 25.1, 24.9, 23.2; **HR-MS (ESI)** calcd for $C_{18}H_{26}NO^+$ $[M+H]^+$ 272.2014 found 272.2003.

5,5,6,6-tetramethyl-3-(3-phenylprop-2-yn-1-yl)piperidin-2-one (3q)

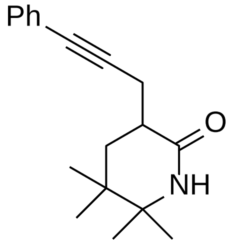

Prepared from 2,3-dimethyl-2-butene and methyl 2-methylene-5-phenylpent-4-ynoate following General procedure B with 12 h irradiation to give **3q** as a white solid in 13.1 mg (49% yield). **^{1}H NMR** (400 MHz, $CDCl_3$) δ 7.32 – 7.30 (m, 2H), 7.23 – 7.18 (m, 3H), 5.67 (s, 1H), 2.85 (dd, *J* = 16.8, 7.2 Hz, 1H), 2.72 (dd, *J* = 16.8, 4.0 Hz, 1H), 2.55 (dtd, *J* = 11.6, 7.2, 4.0 Hz, 1H), 2.09 (dd, *J* = 13.9, 11.6 Hz, 1H), 1.60 (dd, *J* = 13.9, 7.1 Hz, 1H), 1.21 (s, 3H), 1.07 (s, 3H), 1.00 (s, 3H), 0.94 (s, 3H); **^{13}C NMR** (101 MHz, $CDCl_3$) δ 172.3, 131.7, 128.3, 127.8, 123.8, 87.7, 82.4, 58.4, 37.9, 37.4, 34.9, 28.0, 25.1, 24.8, 23.2, 22.0; **HR-MS (ESI)** calcd for $C_{18}H_{24}NO^+$ $[M+H]^+$ 270.1858 found 270.1846.

5,5,6,6-tetramethyl-3-(prop-2-yn-1-yl)piperidin-2-one (3r)

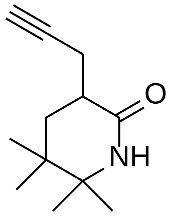

Prepared from 2,3-dimethyl-2-butene and methyl 2-methylenepent-4-ynoate following General procedure A with 12 h irradiation to give **3r** as a white solid in 15.6 mg (81% yield). **^{1}H NMR** (400 MHz, $CDCl_3$) δ 5.82 (s, 1H), 2.69 – 2.57 (m, 2H), 2.56 – 2.48 (m, 1H), 2.05 – 1.93 (m, 2H), 1.61 (dd, *J* = 13.8, 7.0 Hz, 1H), 1.24 (s, 3H), 1.12 (s, 3H), 1.04 (s, 3H), 0.99

(s, 3H); **^{13}C NMR** (101 MHz, $CDCl_3$) δ 172.2, 82.2, 70.1, 58.4, 37.7, 37.1, 34.9, 28.1, 25.1, 24.9, 23.1, 21.0; **HR-MS (ESI)** calcd for $C_{12}H_{20}NO^+$ $[M+H]^+$ 194.1545 found 194.1537.

3-(4-chlorobutyl)-5,5,6,6-tetramethylpiperidin-2-one (3s)

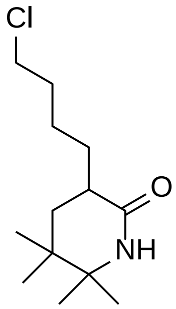

Prepared from 2,3-dimethyl-2-butene and methyl 6-chloro-2-methylenehexanoate following General procedure A with 12 h irradiation to give **3s** as a white solid in 15.2 mg (62% yield). **^{1}H NMR** (400 MHz, $CDCl_3$) δ 5.57 (s, 1H), 3.57 – 3.53 (m, 2H), 2.36 (dtd, *J* = 11.6, 7.6, 3.8 Hz, 1H), 1.93 (td, *J* = 9.8, 8.7, 4.8 Hz, 1H), 1.84 – 1.75 (m, 2H), 1.69 (dd, *J* = 13.8, 11.6 Hz, 1H), 1.53 – 1.47 (m, 4H), 1.20 (s, 3H), 1.12 (s, 3H), 1.02 (s, 3H), 0.97 (s, 3H); **^{13}C NMR** (151 MHz, $CDCl_3$) 173.9, 58.0, 45.1, 38.3, 37.6, 34.8, 32.7, 30.9, 28.0, 25.1, 25.0, 24.1, 23.1; **HR-MS (ESI)** calcd for $C_{13}H_{25}ClNO^+$ $[M+H]^+$ 246.1625 found 246.1615.

3-(4-bromobutyl)-5,5,6,6-tetramethylpiperidin-2-one (3t)

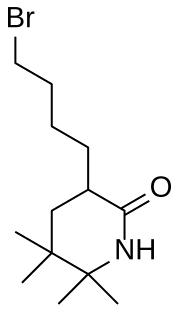

Prepared from 2,3-dimethyl-2-butene and methyl 6-bromo-2-methylenehexanoate following General procedure A with 12 h irradiation to give **3t** as a white solid in 16.2 mg (56% yield). **^{1}H NMR** (600 MHz, $CDCl_3$) δ 5.66 (s, 1H), 3.42 (td, *J* = 6.8, 4.4 Hz, 2H), 2.34 (dtd, *J* = 11.6, 7.6, 3.8 Hz, 1H), 1.93 (ddd, *J* = 8.6, 6.8, 4.4 Hz, 1H), 1.87 (tdd, *J* = 8.6, 6.8, 3.8 Hz, 2H), 1.68 (dd, *J* = 13.8, 11.6 Hz, 1H), 1.54 – 1.42 (m, 4H), 1.20 (s, 3H), 1.11 (s, 3H), 1.02 (s, 3H), 0.97 (s, 3H); **^{13}C NMR** (151 MHz, $CDCl_3$) 173.9, 58.0, 38.3, 37.6, 34.8, 34.0, 32.9, 30.8, 28.0, 25.4, 25.1, 25.0, 23.1; **HR-MS (ESI)** calcd for $C_{13}H_{25}BrNO^+$ $[M+H]^+$ 290.1120 found 290.1108.

3-((benzyloxy)methyl)-5,5,6,6-tetramethylpiperidin-2-one (3u)

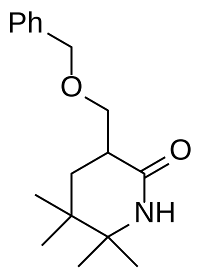

Prepared from 2,3-dimethyl-2-butene and methyl 2-((benzyloxy)methyl)acrylate following General procedure A with 12 h irradiation to give **3u** as a white solid in 26.5 mg (96% yield). **^{1}H NMR** (600 MHz, $CDCl_3$) δ 7.28 – 7.23 (m, 4H), 7.22 – 7.17 (m, 1H), 5.68 (s, 1H), 4.50 (d, *J* = 12.0 Hz, 1H), 4.43 (d, *J* = 12.0 Hz, 1H), 3.80 (dd, *J* = 9.0, 6.0 Hz, 1H), 3.65 (dd, *J* = 9.0, 3.4 Hz, 1H), 2.52 (dddd, *J* = 11.0, 7.2, 6.0, 3.4 Hz, 1H), 2.01 (dd, *J* = 13.9, 11.7 Hz, 1H), 1.46 (dd, *J* = 13.9, 7.3 Hz, 1H), 1.16 (s, 3H), 1.05 (s, 3H), 0.96 (s, 3H), 0.91 (s, 3H); **^{13}C NMR** (151 MHz, $CDCl_3$) δ 172.0, 138.7, 128.4, 127.6, 127.5, 73.4, 70.8, 58.1, 39.3, 36.1, 34.7, 27.7, 25.0, 24.9, 23.0; **HR-MS (ESI)** calcd for $C_{17}H_{26}NO_2^+$ $[M+H]^+$ 276.1964 found 276.1953.

5,5,6,6-tetramethyl-3-((tetrahydrofuran-2-yl)methyl)piperidin-2-one (3v)

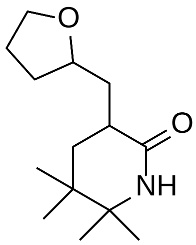

Prepared from 2,3-dimethyl-2-butene and methyl 2-((tetrahydrofuran-2-yl)methyl)acrylate following General procedure A with 12 h irradiation to give **3v** as a white solid in 18.6 mg (78% yield) with 1:1 dr. **^{1}H NMR** (400 MHz, $CDCl_3$) δ 5.62 (s, 0.5H) 5.61 (s, 0.5H), 4.03 – 3.97 (m, 0.5H), 3.97 – 3.91 (m, 0.5H), 3.86 – 3.81 (m, 1H), 3.66 – 3.56 (m, 1H), 2.62 – 2.55 (m, 0.5H), 2.43 – 2.36 (m, 0.5H), 2.22 – 2.13 (m, 1H), 2.03 – 1.94 (m, 1H), 1.93 – 1.79 (m, 2H), 1.75 (dd, *J* = 13.9, 11.6 Hz, 1H), 1.63 (ddd, *J* = 13.7, 7.1, 4.6 Hz, 1H), 1.57 – 1.43 (m, 2H), 1.19 (s, 3H), 1.10 (s, 1.5H), 1.09 (s, 1.5H), 1.02 (s, 1.5H), 1.01 (s, 1.5H), 0.95 (s, 1.5H), 0.94 (s, 1.5H); **^{13}C NMR** (101 MHz, $CDCl_3$) δ 174.3, 174.1, 78.7, 75.9, 67.8, 67.7, 58.04, 57.97, 39.4, 38.3, 37.9, 37.6, 36.6, 35.3, 34.81, 34.77, 32.0, 31.8, 27.9, 27.8, 25.7, 25.6, 25.12, 25.09, 24.97, 24.95, 23.1, 23.0; **HR-MS (ESI)** calcd for $C_{14}H_{26}NO_2^+$ $[M+H]^+$ 240.1964 found 240.1955.

3-di(*tert*-butoxycarbonyl)amino -5,5,6,6-tetramethylpiperidin-2-one (3w)

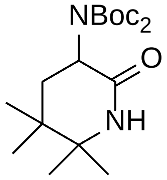

Prepared from 2,3-dimethyl-2-butene and methyl 2-[di(*tert*-butoxycarbonyl)amino]prop-2-enoate following General procedure A with 12 h irradiation to give **3w** as a white solid in 25.7 mg (69% yield).

^{1}H NMR (400 MHz, $CDCl_3$) δ 5.44 (s, 1H), 4.79 (dd, *J* = 12.1, 6.6 Hz, 1H), 2.37 (dd, *J* = 12.7, 12.1 Hz, 1H), 1.60 (dd, *J* = 12.7, 6.6 Hz, 1H), 1.50 (s, 18H), 1.35 (s, 3H), 1.11 (s, 6H), 0.98 (s, 3H); **^{13}C NMR** (101 MHz, $CDCl_3$) δ 169.4, 152.6, 82.9, 58.7, 54.1, 37.4, 35.8, 28.4, 28.2, 28.1, 27.3, 25.3, 24.9, 23.2; **HR-MS (ESI)** calcd for $C_{19}H_{34}N_2O_5Na^+$ $[M+Na]^+$ 393.2365 found 393.2355.

***N*-(5,5,6,6-tetramethyl-2-oxopiperidin-3-yl)benzamide (3x)**

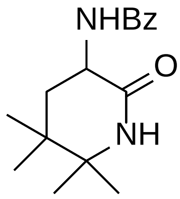

Prepared from 2,3-dimethyl-2-butene and methyl 2-benzamidoacrylate following General procedure A with 12 h irradiation to give **3x** as a white solid in 24.9 mg (91% yield). **^{1}H NMR** (600 MHz, $CDCl_3$) δ 7.79 – 7.78 (m, 2H), 7.47 – 7.25 (m, 3H), 6.02 (s, 1H), 4.46 (dt, *J* = 12.0, 6.5 Hz, 1H), 2.14 (dd, *J* = 13.4, 6.5 Hz, 1H), 1.99 (dd, *J* = 13.4, 12.0 Hz, 1H), 1.27 (s, 3H), 1.14 (s, 3H), 1.11 (s, 3H), 0.96 (s, 3H); **^{13}C NMR** (151 MHz, $CDCl_3$) δ 171.1, 167.7, 134.1, 131.7, 128.5, 127.3, 59.3, 48.8, 38.7, 35.4, 27.7, 25.1, 24.5, 23.4; **HR-MS (ESI)** calcd for $C_{16}H_{23}N_2O_2^+$ $[M+H]^+$ 275.1760 found 275.1750.

3-(2-hydroxyethyl)-5,5,6,6-tetramethylpiperidin-2-one (3y)

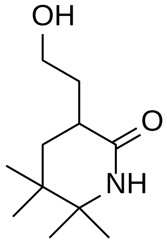

Prepared from 2,3-dimethyl-2-butene and 3-methylenedihydrofuran-2(3*H*)-one following General procedure A with 12 h irradiation to give **3y** as a white solid in 15.7 mg (79% yield) by recrystallization instead of silica gel chromatography. **^{1}H NMR** (400 MHz, $CDCl_3$) δ 5.60 (s, 1H), 3.84 – 3.76 (m, 1H), 3.69 (td, *J* = 11.0, 10.5, 3.0 Hz, 1H), 3.25 (s, 1H), 2.55 – 2.45 (m, 1H), 1.99 – 1.88 (m, 1H), 1.77 (dd, *J* = 13.8, 12.0 Hz, 1H), 1.62 (dq, *J* = 14.7, 3.8 Hz, 1H), 1.55 – 1.47 (m, 1H), 1.21 (s, 3H), 1.14 (s, 3H), 1.03 (s, 3H), 0.96 (s, 3H); **^{13}C NMR** (101 MHz, $CDCl_3$) δ 175.3, 62.1, 58.6, 40.4, 38.0, 35.3, 34.7, 27.9, 25.0, 24.7, 23.1; **HR-MS (ESI)** calcd for $C_{11}H_{22}NO_2^+$ $[M+H]^+$ 200.1651 found 200.1643.

5,5,6,6-tetramethyl-3-((phenylamino)methyl)piperidin-2-one (3z)

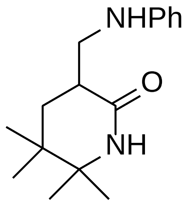

Prepared from 2,3-dimethyl-2-butene and 3-methylene-1-phenylazetidin-2-one following General procedure A with 12 h irradiation to give **3z** as a white solid in 21.9 mg (84% yield). **^{1}H NMR** (400 MHz, $CDCl_3$) δ 7.21 – 7.14 (m, 2H), 6.75 – 6.67 (m, 3H), 5.44 (s, 1H), 3.34 (d, *J* = 6.0 Hz, 2H), 2.70 – 2.62 (m, 1H), 1.86 – 1.80 (m, 1H), 1.56 – 1.51 (m, 1H), 1.21 (s, 3H), 1.14 (s, 3H), 1.04 (s, 3H), 0.98 (s, 3H); **^{13}C NMR** (151 MHz, $CDCl_3$) δ 173.6, 147.9, 129.4, 118.2, 113.9, 58.12, 46.9, 37.4, 37.2, 34.8, 27.9, 25.0, 24.8, 23.2; **HR-MS (ESI)** calcd for $C_{16}H_{25}N_2O^+$ $[M+H]^+$ 261.1967 found 261.1958.

***N*-phenyl-2-(4,4,5,5-tetramethyl-2-oxopyrrolidin-3-yl)acetamide (3aa)**

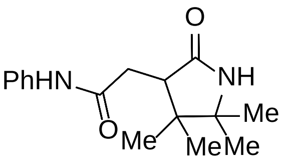

Prepared from 2,3-dimethyl-2-butene and *N*-Phenylmaleimide following General procedure A with 12 h irradiation to give **3aa** as a white solid in 27.4 mg (99% yield). **^{1}H NMR** (600 MHz, $CDCl_3$) δ 10.32 (s, 1H), 7.57 – 7.52 (m, 2H), 7.29 – 7.23 (m, 2H), 7.06 – 7.01 (m, 1H), 6.51 (s, 1H), 2.84 (dd, *J* = 10.6, 2.2 Hz, 1H), 2.66 (dd, *J* = 14.5, 10.6 Hz, 1H), 2.21 (dd, *J* = 14.5, 2.2 Hz, 1H), 1.18 (s, 3H), 1.14 (s, 3H), 1.02 (s, 3H), 0.85 (s, 3H); **^{13}C NMR** (151 MHz, $CDCl_3$) δ 178.1, 170.6, 138.8, 128.9, 123.7, 119.6, 60.5, 48.1, 45.3, 34.5, 25.1, 22.7, 20.6, 19.2; **HR-MS (ESI)** calcd for $C_{16}H_{23}N_2O_2^+$ $[M+H]^+$ 275.1760 found 275.1750.

***cis*-3,5-bis(4-fluorophenyl)-6,6-dimethylpiperidin-2-one (4a)**

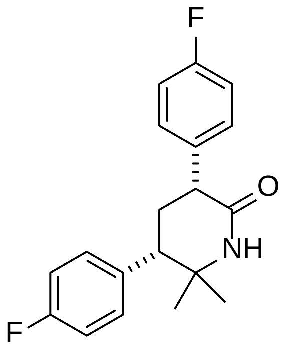

Prepared from 4-fluoro-β,β-dimethylstyrene and methyl 2-(4-fluorophenyl)acrylate following General procedure A with 24 h irradiation to give **4a** as a white solid in 22.5 mg (71% yield) with 3.1:1 dr. Major diasteroisomer: **^{1}H NMR** (400 MHz, $CDCl_3$) δ 7.25 – 7.14 (m, 4H), 7.07 – 6.94 (m, 4H), 6.14 (s, 1H), 3.72 (dd, *J* = 12.2, 6.5 Hz, 1H), 3.10 (dd, *J* = 13.4, 2.5 Hz, 1H), 2.56 – 2.46 (m, 1H), 2.16 – 2.11 (m, 1H), 1.26 (s, 3H), 1.16 (s, 3H); **^{19}F NMR** (376 MHz, $CDCl_3$) δ -115.17 – -115.25 (m, 1F), -115.87 – -115.94 (m, 1F); **^{13}C NMR** (101 MHz, $CDCl_3$) δ 172.2, 163.2 (d, *J* = 244.6 Hz),160.8 (d, *J* = 243.8 Hz), 136.4 (d, *J* = 3.3 Hz), 135.4 (d, *J* = 3.4 Hz), 130.6 (d, *J* = 8.0 Hz),

130.0 (d, J = 8.0 Hz), 115.7 (d, J = 21.5 Hz), 115.2 (d, J = 21.2 Hz), 56.5, 49.5, 48.8, 34.5, 30.5, 25.5; **HR-MS (ESI)** calcd for $C_{19}H_{20}F_2NO^+$ $[M+H]^+$ 316.1513 found 316.1506.

***cis*-5-(4-fluorophenyl)-6,6-dimethyl-3-(4-(trifluoromethyl)phenyl)piperidin-2-one (4b)**

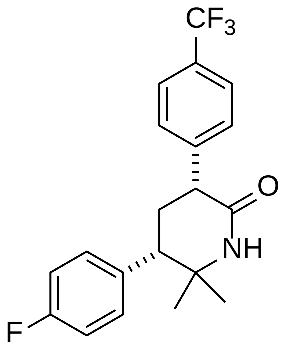

Prepared from 4-fluoro-β,β-dimethylstyrene and methyl 2-(4-trifluoromethylphenyl)acrylate following General procedure A with 24 h irradiation to give **4b** as a white solid in 24.9 mg (68% yield) with 2.7:1 dr. Major diasteroisomer: **^{1}H NMR** (400 MHz, $CDCl_3$) δ 7.63 – 7.56 (m 2H), 7.42 – 7.34 (m, 2H), 7.21 – 7.14 (m, 2H), 7.04 – 6.94 (m, 2H), 6.08 (s, 1H), 3.79 (dd, J = 12.2, 6.5 Hz, 1H), 3.09 (dd, J = 13.4, 2.5 Hz, 1H), 2.56 – 2.46 (m, 1H), 2.20 – 2.14 (m, 1H), 1.25 (s, 3H), 1.19 (s, 3H); **^{19}F NMR** (376 MHz, $CDCl_3$) δ -62.54 (s, 3F), -115.02 – -115.11 (m, 1F); **^{13}C NMR** (151 MHz, $CDCl_3$) 171.5, 162.2 (d, J = 247.6 Hz), 141.6 (s), 135.3 (d, J = 4.5 Hz), 132.1 (s), 131.1 (q, J = 48.3 Hz), 130.6 (d, J = 7.5 Hz), 125.3 (q, J = 4.0 Hz), 124.3 (q, J = 271.8 Hz), 115.2 (d, J = 21.1 Hz), 56.5, 49.5, 49.4, 34.4, 30.5, 25.5; **HR-MS (ESI)** calcd for $C_{20}H_{20}F_4NO^+$ $[M+H]^+$ 366.1481 found 366.1472.

***cis*-5-(4-fluorophenyl)-3-(4-methoxyphenyl)-6,6-dimethylpiperidin-2-one (4c)**

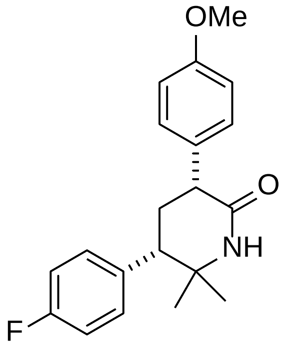

Prepared from 4-fluoro-β,β-dimethylstyrene and methyl 2-(4-methoxyphenyl)acrylate following General procedure A with 24 h irradiation to give **4c** as a white solid in 28.5 mg (87% yield) with 5.4:1 dr. Major diasteroisomer: **^{1}H NMR** (400 MHz, $CDCl_3$) δ 7.20 – 7.15 (m, 4H), 7.00 (t, J = 8.0 Hz, 2H), 7.02 – 6.88 (m, 2H), 6.14 (s, 1H), 3.79 (s, 3H), 3.66 (dd, J = 12.6, 6.5 Hz, 1H), 3.08 (dd, J = 13.3, 2.4 Hz, 1H), 2.55 – 2.45 (m, 1H), 2.15 (ddd, J = 13.7, 6.5, 2.4 Hz, 1H), 1.24 (s, 3H), 1.17 (s, 3H); **^{19}F NMR** (376 MHz, $CDCl_3$) δ -115.32 – -115.40 (m, 1F); **^{13}C NMR** (101 MHz, $CDCl_3$) δ 172.5, 162.1 (d, J = 247.4 Hz), 158.7, 135.6 (d, J = 3.0 Hz), 132.8, 130.5 (d, J = 8.0 Hz), 129.4,

115.1 (d, J = 21.2 Hz), 114.4, 56.4, 55.4, 49.6, 48.7, 34.6, 30.7, 25.5; **HR-MS (ESI)** calcd for $C_{20}H_{23}FNO_2^+$ $[M+H]^+$ 328.1713 found 328.1703.

***cis*-3-(2-fluorophenyl)-5-(4-fluorophenyl)-6,6-dimethylpiperidin-2-one (4d)**

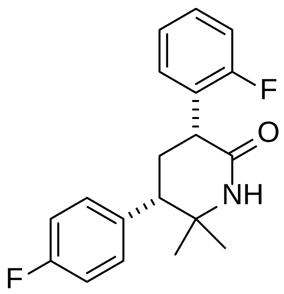

Prepared from 4-fluoro-β,β-dimethylstyrene and methyl 2-(2-fluorophenyl)acrylate following General procedure A with 24 h irradiation to give **4d** as a white solid in 15.8 mg (50% yield) with 2.6:1 dr. Major diasteroisomer: **^{1}H NMR** (400 MHz, $CDCl_3$) δ 7.26 – 7.12 (m, 5H), 7.11 – 7.05 (m, 1H), 7.02 – 6.98 (m, 2H), 5.93 (s, 1H), 3.84 (dd, J = 12.1, 6.5 Hz, 1H), 3.08 (dd, J = 13.3, 2.6 Hz, 1H), 2.66 – 2.56 (m, 1H), 2.13 (ddd, J = 13.3, 6.5, 2.6 Hz, 1H), 1.23 (s, 3H), 1.19 (s, 3H); **^{19}F NMR** (376 MHz, $CDCl_3$) δ -115.20 – -115.28 (m, 1F), -116.42 – -116.52 (m, 1F); **^{13}C NMR** (101 MHz, $CDCl_3$) δ 171.4, 162.7 (d, J = 245.0 Hz), 160.3 (d, J = 245.0 Hz), 135.6 (d, J = 3.0 Hz), 130.9 (d, J = 5.0 Hz), 130.7 (d, J = 8.0 Hz), 129.0 (d, J = 9.0 Hz), 127.9 (d, J = 15.0 Hz), 124.5 (d, J = 4.0 Hz), 116.0 (d, J = 21.0 Hz), 115.1 (d, J = 21.0 Hz), 56.3, 49.5, 44.8, 32.5, 30.5, 24.8; **HR-MS (ESI)** calcd for $C_{19}H_{20}F_2NO^+$ $[M+H]^+$ 316.1513 found 316.1505.

***cis*-5-(4-fluorophenyl)-3-(2-methoxyphenyl)-6,6-dimethylpiperidin-2-one (4e)**

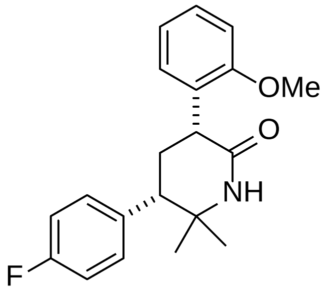

Prepared from 4-fluoro-β,β-dimethylstyrene and methyl 2-(2-methoxyphenyl)acrylate following General procedure A with 24 h irradiation to give **4e** as a white solid in 13.4 mg (41% yield) with 2.2:1 dr with 50% conversion of 4-fluoro-β,β-dimethylstyrene. Major diasteroisomer: **^{1}H NMR** (400 MHz, $CDCl_3$) δ 7.28 – 7.24 (m, 1H), 7.21 – 7.11 (m, 2H), 6.99 (t, J = 8.8 Hz, 2H), 7.02 – 6.96 (m, 2H), 6.15 (s, 1H), 3.80 (s, 3H), 3.70 – 3.66 (m, 1H), 3.07 (dd, J = 13.2, 2.4 Hz, 1H), 2.54 – 2.47 (m, 1H), 2.20 – 2.14 (m, 1H), 1.23 (s, 3H), 1.17 (s, 3H); **^{19}F NMR** (376 MHz, $CDCl_3$) δ -115.28 – -115.35 (m, 1F); **^{13}C NMR** (101 MHz, $CDCl_3$) δ 172.1, 162.1 (d, J = 246.4 Hz), 159.9, 142.3, 135.5 (d, J = 4.0 Hz), 130.6 (d, J = 8.1 Hz), 129.9, 120.8, 115.1 (d, J = 21.2 Hz),

114.7, 112.2, 56.4, 55.3, 49.5, 43.9, 34.5, 30.6, 25.5; **HR-MS (ESI)** calcd for calcd for $C_{20}H_{23}FNO_2^+$ $[M+H]^+$ 328.1713 found 328.1703.

***cis*-5-(4-fluorophenyl)-3-(3-methoxyphenyl)-6,6-dimethylpiperidin-2-one (4f)**

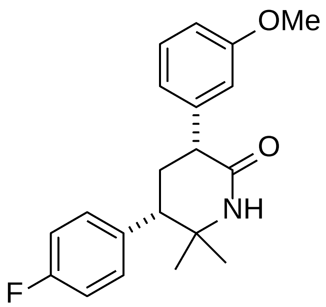

Prepared from 4-fluoro-β,β-dimethylstyrene and methyl 2-(3-methoxyphenyl)acrylate following General procedure A with 24 h irradiation to give **4f** as a white solid in 19.9 mg (61% yield) with 4.0:1 dr. Major diasteroisomer: **^{1}H NMR** (600 MHz, $CDCl_3$) 7.33 – 7.24 (m, 1H), 7.19 (dd, *J* = 8.4, 5.3 Hz, 2H), 7.02 (t, *J* = 8.4 Hz, 2H), 6.92 – 6.78 (m, 3H), 6.49 (s, 1H), 3.82 (s, 3H), 3.70 (dd, *J* = 12.0, 6.7 Hz, 1H), 3.08 (dd, *J* = 13.4, 2.5 Hz, 1H), 2.57 – 2.50 (m, 1H), 2.21 – 2.16 (m, 1H), 1.25 (s, 3H), 1.20 (s, 3H); **^{19}F NMR** (564 MHz, $CDCl_3$) δ -115.31 – -115.36 (m, 1F); **^{13}C NMR** (151 MHz, $CDCl_3$) δ 172.4, 162.1 (d, *J* = 246.0 Hz), 159.9, 142.2, 135.5 (d, *J* = 3.4 Hz), 130.6 (d, *J* = 7.8 Hz), 129.9, 120.8, 115.1 (d, *J* = 21.2 Hz), 114.7, 112.2, 56.4, 55.3, 49.52, 49.48, 34.5, 30.5, 25.4; **HR-MS (ESI)** calcd for $C_{20}H_{23}FNO_2^+$ $[M+H]^+$ 328.1713 found 328.1703.

***cis*-3-(4-methoxyphenyl)-6,6-dimethyl-5-phenylpiperidin-2-one (4g)**

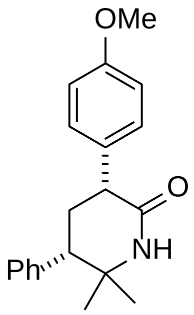

Prepared from β,β-dimethylstyrene and methyl 2-(4-methoxyphenyl)acrylate following General procedure A with 24 h irradiation to give **4g** as a white solid in 21.7 mg (70% yield) with 5.6:1 dr. Major diasteroisomer: **^{1}H NMR** (400 MHz, $CDCl_3$) δ 7.34 – 7.26 (m, 3H), 7.24 – 7.15 (m, 4H), 6.89 (d, *J* = 8.6 Hz, 2H), 5.97 (s, 1H), 3.79 (s, 3H), 3.66 (dd, *J* = 12.1, 6.4 Hz, 1H), 3.08 (dd, *J* = 13.2, 2.5 Hz, 1H), 2.60 – 2.50 (m, 1H), 2.17 (ddd, *J* = 13.7, 6.5, 2.5 Hz, 1H), 1.24 (s, 3H), 1.19 (s, 3H); **^{13}C NMR** (101 MHz, $CDCl_3$) δ 172.6, 158.6, 139.9, 133.0, 129.5, 129.3, 128.2, 127.3, 114.3, 56.5, 55.4, 50.3, 48.8, 34.4, 30.8, 25.7; **HR-MS (ESI)** calcd for $C_{20}H_{24}NO_2^+$ $[M+H]^+$ 310.1807 found 310.1798.

***cis*-3-(4-methoxyphenyl)-6,6-dimethyl-5-(*p*-tolyl)piperidin-2-one (4h)**

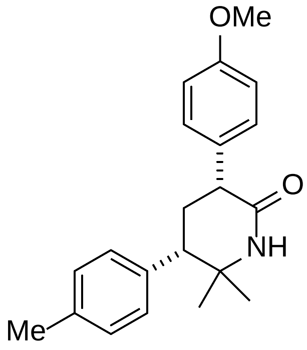

Prepared from 4,β,β-trimethylstyrene and methyl 2-(4-methoxyphenyl)acrylate following General procedure A with 24 h irradiation to give **4h** as a white solid in 26.4 mg (82% yield) with 6.6:1 dr. Major diasteroisomer: **^{1}H NMR** (400 MHz, $CDCl_3$) δ 7.22 – 7.15 (m, 2H), 7.14 – 7.03 (m, 4H), 6.93 – 6.86 (m, 2H), 5.84 (s, 1H), 3.80 (s, 3H), 3.65 (dd, *J* = 12.1, 6.5 Hz, 1H), 3.05 (dd, *J* = 13.4, 2.5 Hz, 1H), 2.60 – 2.50 (m, 1H), 2.33 (s, 3H), 2.14 (ddd, *J* = 13.7, 6.5, 2.5 Hz, 1H), 1.24 (s, 3H), 1.18 (s, 3H); **^{13}C NMR** (101 MHz, $CDCl_3$) δ 173.0, 158.6, 136.9, 136.8, 133.0, 129.4, 129.1, 128.9, 114.3, 56.5, 55.4, 49.8, 48.8, 34.5, 30.6, 25.6, 21.1; **HR-MS (ESI)** calcd for $C_{21}H_{26}NO_2^+$ [M+H]$^+$ 324.1964 found 324.1955.

***cis*-5-(4-(*tert*-butyl)phenyl)-3-(4-methoxyphenyl)-6,6-dimethylpiperidin-2-one (4i)**

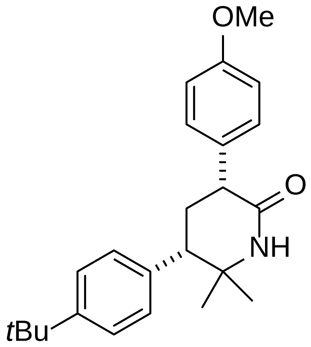

Prepared from 4-*tert*-butyl-β,β-dimethylstyrene and methyl 2-(4-methoxyphenyl)acrylate following General procedure A with 24 h irradiation to give **4i** as a white solid in 31.4 mg (86% yield) with 5.5:1 dr. Major diasteroisomer: **^{1}H NMR** (400 MHz, $CDCl_3$) δ 7.33 – 7.28 (m, 2H), 7.21 – 7.15 (m, 2H), 7.15 – 7.10 (m, 2H), 6.92 – 6.86 (m, 2H), 6.21 (s, 1H), 3.79 (s, 3H), 3.65 (dd, *J* = 12.1, 6.5 Hz, 1H), 3.05 (dd, *J* = 13.3, 2.5 Hz, 1H), 2.55 – 2.51 (m, 1H), 2.15 (ddd, *J* = 13.7, 6.5, 2.5 Hz, 1H), 1.31 (s, 9H), 1.25 (s, 3H), 1.19 (s, 3H); **^{13}C NMR** (101 MHz, $CDCl_3$) δ 172.8, 158.6, 150.2, 136.8, 133.1, 129.4, 128.9, 125.0, 114.3, 56.6, 55.4, 49.8, 48.8, 34.53, 34.48, 31.4, 30.7, 25.6; **HR-MS (ESI)** calcd for $C_{24}H_{32}NO_2^+$ [M+H]$^+$ 366.2433 found 366.2424.

***cis*-3,5-bis(4-methoxyphenyl)-6,6-dimethylpiperidin-2-one (4j)**

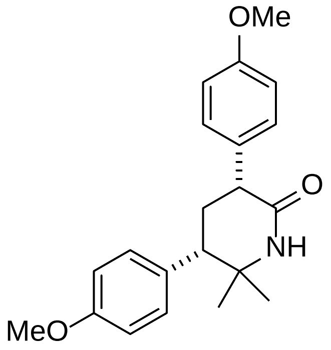

Prepared from 4-methoxy-β,β-dimethylstyrene and methyl 2-(4-methoxyphenyl)acrylate following General procedure A with 24 h irradiation to give **4j** as a white solid in 21.0 mg (62% yield) with 4.2:1 dr. **^{1}H NMR** (400 MHz, $CDCl_3$) δ 7.18 (d, *J* = 8.0 Hz, 1H), 7.12 (d, *J* = 8.4 Hz, 1H), 6.90 (d, *J* = 8.4 Hz, 1H), 6.84 (d, *J* = 8.0 Hz, 1H), 6.16 (s, 1H), 3.80 (s, 6H), 3.66 (dd, *J* = 12.1, 6.5 Hz, 1H), 3.03 (dd, *J* = 13.3, 2.5 Hz, 1H), 2.53 – 2.47 (m, 1H), 2.15 (ddd, *J* = 13.7, 6.5, 2.5 Hz, 1H), 1.24 (s, 3H), 1.18 (s, 3H); **^{13}C NMR** (101 MHz, $CDCl_3$) δ 172.9, 158.9, 158.7, 132.8, 131.8, 130.2, 129.5, 114.4, 113.6, 56.8, 55.5, 55.4, 49.4, 48.8, 34.6, 30.7, 25.6; **HR-MS (ESI)** calcd for $C_{21}H_{26}NO_3^+$ $[M+H]^+$ 340.1913 found 340.1906.

***cis*-5-(4-bromophenyl)-3-(4-methoxyphenyl)-6,6-dimethylpiperidin-2-one (4k)**

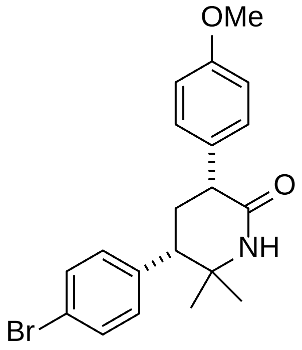

Prepared from 4-bromide-β,β-dimethylstyrene and methyl 2-(4-methoxyphenyl)acrylate following General procedure B to give **4k** as a white solid in 19.5 mg (50% yield) with 3.4:1 dr. Major diasteroisomer: **^{1}H NMR** (600 MHz, $CDCl_3$) δ 7.43 (d, *J* = 8.4 Hz, 2H), 7.17 (d, *J* = 8.6 Hz, 2H), 7.09 (d, *J* = 8.4 Hz, 2H), 6.89 (d, *J* = 8.6 Hz, 2H), 6.00 (s, 1H), 3.79 (s, 3H), 3.65 (dd, *J* = 12.2, 6.5 Hz, 1H), 3.05 (dd, *J* = 13.4, 2.5 Hz, 1H), 2.50 (td, *J* = 13.5, 12.2 Hz, 1H), 2.14 (ddd, *J* = 13.7, 6.5, 2.5 Hz, 1H), 1.22 (s, 3H), 1.17 (s, 3H); **^{13}C NMR** (151 MHz, $CDCl_3$) δ 172.4, 158.7, 138.9, 132.7, 131.4, 130.9, 129.4, 121.4, 114.4, 56.3, 55.4, 49.8, 48.7, 34.3, 30.7, 25.6; **HR-MS (ESI)** calcd for $C_{20}H_{23}BrNO_2^+$ $[M+H]^+$ 388.0912 found 388.0903.

***cis*-5-([1,1'-biphenyl]-4-yl)-3-(4-methoxyphenyl)-6,6-dimethylpiperidin-2-one (4l)**

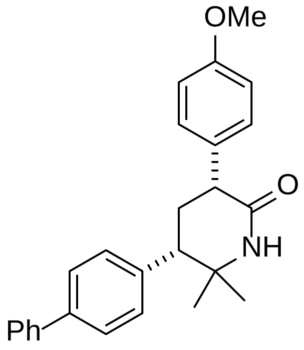

Prepared from 4-phenyl-β,β-dimethylstyrene and methyl 2-(4-methoxyphenyl)acrylate following General procedure B to give **4l** as a white solid in 15.4 mg (40% yield) with 3.6:1 dr. Major diasteroisomer: **^{1}H NMR** (400 MHz, $CDCl_3$) δ 7.64 – 7.52 (m, 4H), 7.44 (t, *J* = 7.6 Hz, 2H), 7.39 – 7.32 (m, 2H), 7.28 (d, *J* = 7.9 Hz, 1H), 7.20 (d, *J* = 8.4 Hz, 2H), 6.90 (d, *J* = 8.6 Hz, 2H), 6.13 (s, 1H), 3.80 (s, 3H), 3.68 (dd, *J* = 12.0, 6.4 Hz, 1H), 3.13 (dd, *J* = 13.3, 2.5 Hz, 1H), 2.55 – 2.46 (m, 1H), 2.21 (ddd, *J* = 13.7, 6.4, 2.5 Hz, 1H), 1.30 (s, 3H), 1.24 (s, 3H); **^{13}C NMR** (151 MHz, $CDCl_3$) δ 172.6, 158.7, 140.6, 140.2, 139.0, 133.0, 129.7, 129.5, 128.9, 127.5, 127.1, 126.9, 114.4, 56.6, 55.4, 50.0, 48.8, 34.5, 30.8, 25.7; **HR-MS (ESI)** calcd for $C_{26}H_{28}NO_2^+$ $[M+H]^+$ 386.2120 found 386.2110.

***cis*-3-(4-methoxyphenyl)-6,6-dimethyl-5-(*m*-tolyl)piperidin-2-one (4m)**

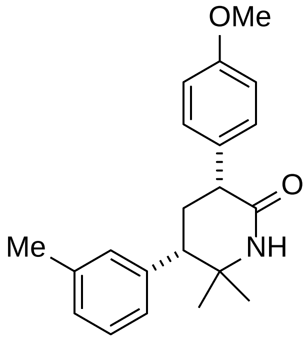

Prepared from 3,β,β-trimethylstyrene and methyl 2-(4-methoxyphenyl)acrylate following General procedure A with 24 h irradiation to give **4m** as a white solid in 23.3 mg (72% yield) with 4.9:1 dr. Major diasteroisomer: **^{1}H NMR** (400 MHz, $CDCl_3$) δ 7.23 – 7.15 (m, 3H), 7.07 (d, *J* = 7.6 Hz, 1H), 7.04 – 6.98 (m, 2H), 6.92 – 6.86 (m, 2H), 6.39 (s, 1H), 3.79 (s, 3H), 3.65 (dd, *J* = 12.1, 6.5 Hz, 1H), 3.04 (dd, *J* = 13.4, 2.5 Hz, 1H), 2.57 – 2.51 (m, 1H), 2.33 (s, 3H), 2.15 (ddd, *J* = 13.7, 6.5, 2.5 Hz, 1H), 1.25 (s, 3H), 1.19 (s, 3H); **^{13}C NMR** (101 MHz, $CDCl_3$) δ 172.9, 158.6, 139.9, 137.7, 133.0, 130.0, 129.4, 128.04, 128.01, 126.4, 114.3, 56.5, 55.4, 50.2, 48.8, 34.5, 30.7, 25.6, 21.6; **HR-MS (ESI)** calcd for $C_{21}H_{26}NO_2^+$ $[M+H]^+$ 324.1964 found 324.1954.

***cis*-3-(4-methoxyphenyl)-6,6-dimethyl-5-(thiophen-2-yl)piperidin-2-one (4n)**

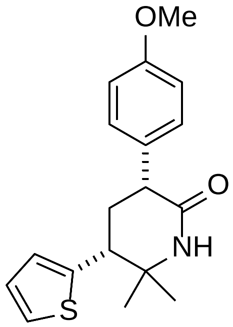

Prepared from 2-(2-methylprop-1-en-1-yl)thiophene and methyl 2-(4-methoxyphenyl)acrylate following General procedure A with 24 h irradiation to give **4n** as a white solid in 9.8 mg (31% yield) with 4.0:1 dr. Major diasteroisomer: **^{1}H NMR** (600 MHz, $CDCl_3$) δ 7.20 (d, *J* = 5.1 Hz, 1H), 7.19 – 7.15 (m, 2H), 6.97 (dd, *J* = 5.1, 3.5 Hz, 1H), 6.91 – 6.88 (m, 2H), 6.88 – 6.82 (m, 1H), 6.11 (s, 1H), 3.79 (s, 3H), 3.65 (dd, *J* = 12.1, 6.8 Hz, 1H), 3.38 (dd, *J* = 13.2, 2.8 Hz, 1H), 2.48 – 2.41 (m, 1H), 2.31 (ddd, *J* = 13.9, 6.8, 2.8 Hz, 1H), 1.35 (s, 3H), 1.25 (s, 3H); **^{13}C NMR** (151 MHz, $CDCl_3$) δ 172.5, 158.7, 142.6, 132.6, 129.5, 126.6, 126.2, 124.2, 114.4, 56.4, 55.4, 48.5, 45.8, 35.8, 30.8, 26.0; **HR-MS (ESI)** calcd for $C_{18}H_{22}NO_2S^+$ [M+H]$^+$ 316.1371 found 316.1364.

***(3S,5S,6R)*-3-(4-methoxyphenyl)-5,6-diphenylpiperidin-2-one (4o)**

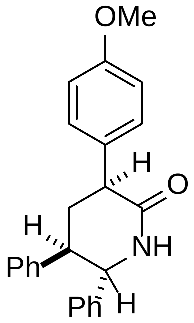

Prepared from *cis*-stilbene and methyl 2-(4-methoxyphenyl)acrylate following General procedure A with 24 h irradiation to give **4o** as a white solid in 25.7 mg (72% yield) with 2.4:1 dr; from *trans*-stilbene 19.5 mg (55% yield) was isolated with 2.4:1 dr. Major diasteroisomer: **^{1}H NMR** (400 MHz, $CDCl_3$) δ 7.19 – 7.16 (m, 2H), 7.16 – 7.11 (m, 3H), 7.11 – 7.05 (m, 3H), 7.01 – 6.93 (m, 2H), 6.90 – 6.81 (m, 4H), 5.93 (s, 1H), 4.58 (d, *J* = 10.8 Hz, 1H), 3.78 (dd, *J* = 12.4, 6.0 Hz, 1H), 3.73 (s, 3H), 3.02 (ddd, *J* = 12.8, 10.4, 2.4 Hz, 1H), 2.52 – 2.41 (m, 1H), 2.27 (ddd, *J* = 13.6, 6.0, 2.8 Hz, 1H); **^{13}C NMR** (151 MHz, $CDCl_3$) 173.1, 158.7, 140.4, 140.2, 132.5, 129.6, 129.1, 128.6, 128.5, 128.3, 127.9, 127.2, 114.3, 65.4, 55.4, 49.7, 48.7, 38.7; **HR-MS (ESI)** calcd for $C_{24}H_{24}NO_2^+$ [M+H]$^+$ 358.1807 found 358.1799.

5-(4-(*tert*-butyl)phenyl)-3-(4-(trifluoromethyl)phenyl)piperidin-2-one (5a)

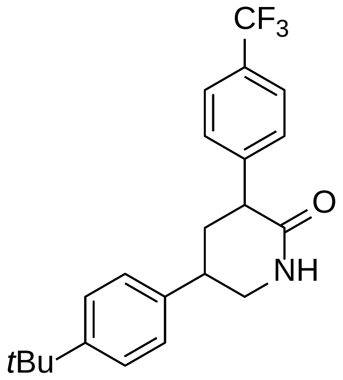

Prepared from 4-*tert*-butylstyrene and methyl 2-(4-trifluoromethylphenyl)acrylate following General procedure C to give **5a** as a white solid in 20.3 mg (54% yield) with 1.2:1 dr. Major diasteroisomer: **^{1}H NMR** (600 MHz, $CDCl_3$) δ 7.62 – 7.55 (m, 2H), 7.41 – 7.31 (m, 4H), 7.19 – 7.11 (m, 2H), 6.96 (s, 1H), 3.81 (dd, *J* = 12.4, 6.1 Hz, 1H), 3.60 – 3.55 (m, 1H), 3.52 – 3.46 (m, 1H), 3.25 (tdd, *J* = 11.9, 5.5, 2.8 Hz, 1H), 2.36 (ddt, *J* = 13.3, 5.7, 2.5 Hz, 1H), 2.27 – 2.20 (m, 1H), 1.30 (s, 9H); **^{19}F NMR** (564 MHz, $CDCl_3$) δ -62.50 (s, 3F); **^{13}C NMR** (151 MHz, $CDCl_3$) δ 172.1, 150.5, 144.8, 137.9, 129.3 (q, *J* = 30.8 Hz), 129.0, 126.7, 125.8, 125.7 (q, *J* = 3.6 Hz), 124.3 (q, *J* = 270.0 Hz), 49.5, 49.1, 39.8, 38.1, 34.6, 31.4; **HR-MS (ESI)** calcd for $C_{22}H_{25}F_3NO^+$ $[M+H]^+$ 376.1888 found 376.1879.

5-(4-fluorophenyl)-3-(4-(trifluoromethyl)phenyl)piperidin-2-one (5b)

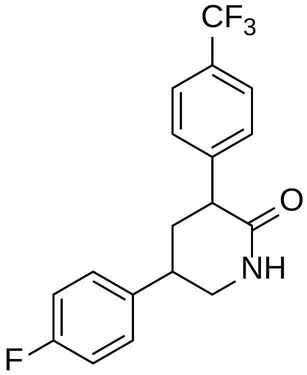

Prepared from 4-fluorostyrene and methyl 2-(4-trifluoromethylphenyl)acrylate following General procedure C to give **5b** as a white solid in 16.5 mg (49% yield) with 1.2:1 dr. Major diasteroisomer: **^{1}H NMR** (400 MHz, $CDCl_3$) δ 7.60 (d, *J* = 8.0 Hz, 2H), 7.39 (t, *J* = 7.6 Hz, 2H), 7.23 – 7.19 (m, 2H), 7.05–6.97 (m, 2H), 6.52 (s, 1H), 3.83 (dd, *J* = 12.4, 6.0 Hz, 1H), 3.58 – 3.53 (m, 1H), 3.50 – 3.43 (m, 1H), 3.32 – 3.23 (m, 1H), 2.39 – 2.32 (m, 1H), 2.26 – 2.19 (m, 1H); **^{19}F NMR** (564 MHz, $CDCl_3$) δ -62.54 (s, 3F), -114.94 – -114.99 (m, 1F); **^{13}C NMR** (151 MHz, $CDCl_3$) δ 172.1, 162.1 (d, *J* = 245.9 Hz), 144.5, 136.7 (d, *J* = 3.3 Hz), 129.5 (q, *J* = 32.4 Hz), 129.0, 128.5 (d, *J* = 7.9 Hz), 125.8 (q, *J* = 3.8 Hz), 124.3 (q, *J* = 270.3 Hz), 115.9 (d, *J* = 21.3 Hz), 49.6, 49.1, 39.6, 38.1; **HR-MS (ESI)** calcd for $C_{18}H_{16}F_4NO^+$ $[M+H]^+$ 338.1168 found 338.1158.

3-(4-fluorophenyl)-5-phenylpiperidin-2-one (5c)

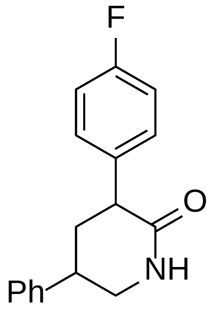

Prepared from styrene and methyl 2-(4-fluorophenyl)acrylate following General procedure C to give **5c** as a white solid in 13.7 mg (51% yield) with 1.6:1 dr. Major diasteroisomer: **^{1}H NMR** (400 MHz, $CDCl_3$) δ 7.36 – 7.29 (m, 2H), 7.29 – 7.18 (m, 5H), 7.05– 6.98 (m, 2H), 6.84 (s, 1H), 3.74 (dd, *J* = 12.4, 6.0 Hz, 1H), 3.59 – 3.53 (m, 1H), 3.47 (t, *J* = 11.6 Hz, 1H), 3.26 (tdd, *J* = 11.6, 5.5, 2.8 Hz, 1H), 2.36 (ddt, *J* = 13.3, 6.2, 2.6 Hz, 1H), 2.29 – 2.17 (m, 1H); **^{19}F NMR** (376 MHz, $CDCl_3$) δ -115.89 – -115.98 (m, 1F); **^{13}C NMR** (101 MHz, $CDCl_3$) δ 172.8, 161.9 (d, *J* = 245.0 Hz), 141.2, 136.4 (d, *J* = 3.2 Hz), 130.1 (d, *J* = 8.0 Hz), 129.0, 127.5, 127.0, 115.7 (d, *J* = 21.4 Hz), 49.6, 48.6, 40.4, 38.3; **HR-MS (ESI)** calcd for $C_{17}H_{17}FNO^+$ $[M+H]^+$ 270.1294 found 270.1285.

5-([1,1'-biphenyl]-4-yl)-3-(4-fluorophenyl)piperidin-2-one (5d)

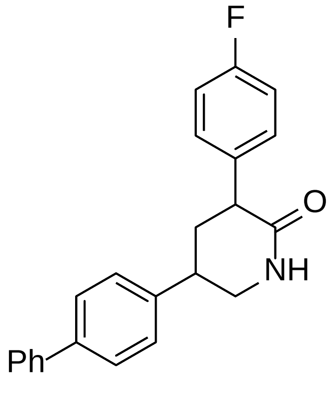

Prepared from 4-vinyl-1,1'-biphenyl and methyl 2-(4-fluorophenyl)acrylate following General procedure C to give **5d** as a white solid in 15.8 mg (46% yield) with 1.5:1 dr. Major diasteroisomer: **^{1}H NMR** (400 MHz, $CDCl_3$) δ 7.60 – 7.52 (m, 3H), 7.46 – 7.41 (m, 2H), 7.38 – 7.30 (m, 2H), 7.29 – 7.21 (m, 3H), 7.03 (t, *J* = 8.6 Hz, 2H), 6.56 (s, 1H), 3.76 (dd, *J* = 12.4, 6.0 Hz, 1H), 3.68 – 3.62 (m, 1H), 3.53 (t, *J* = 11.2 Hz, 1H), 3.36 – 3.29 (m, 1H), 2.40 – 2.37 (m, 1H), 2.26 (dd, *J* = 13.1, 6.5 Hz, 1H); **^{19}F NMR** (564 MHz, $CDCl_3$) δ -115.84 – -115.90 (m, 1F); **^{13}C NMR** (101 MHz, $CDCl_3$) δ 172.8, 162.0 (d, *J* = 246.2 Hz), 140.6, 140.4, 140.3, 136.5 (d, *J* = 3.4 Hz), 130.1 (d, *J* = 8.0 Hz), 128.9, 127.59, 127.56, 127.4, 127.1, 115.6 (d, *J* = 21.7 Hz), 49.4, 48.6, 40.0, 38.3; **HR-MS (ESI)** calcd for $C_{23}H_{21}FNO^+$ $[M+H]^+$ 346.1607 found 346.1598.

5-(4-bromophenyl)-3-(4-fluorophenyl)piperidin-2-one (5e)

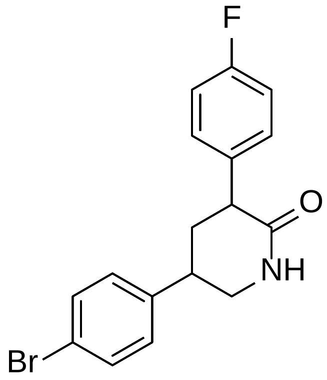

Prepared from 4-bromostyrene and methyl 2-(4-fluorophenyl)acrylate following General procedure C to give **5e** as a white solid in 13.9 mg (40% yield) with 1.8:1 dr. Major diasteroisomer: **^{1}H NMR** (400 MHz, $CDCl_3$) δ 7.48 – 7.41 (m, 2H), 7.25 – 7.19 (m, 2H), 7.15 – 7.10 (m, 2H), 7.05 – 7.00 (m, 2H), 6.88 (s, 1H), 3.73 (dd, *J* = 12.4, 6.0 Hz, 1H), 3.55 – 3.50 (m, 1H), 3.47 – 3.41 (m, 1H), 3.22 (tdd, *J* = 12.4, 5.3, 2.8 Hz, 1H), 2.33 (ddd, *J* = 10.9, 6.2, 2.8 Hz, 1H), 2.22 – 2.13 (m, 1H); **^{19}F NMR** (564 MHz, $CDCl_3$) δ -115.73 – -115.81 (m, 1F); **^{13}C NMR** (151 MHz, $CDCl_3$) δ 172.8, 162.0 (d, *J* = 245.5 Hz), 140.1, 136.2 (d, *J* = 3.3 Hz), 132.1, 130.1 (d, *J* = 8.0 Hz), 128.7, 121.3, 115.7 (d, *J* = 21.4 Hz), 49.3, 39.9, 38.1, 34.4; **HR-MS (ESI)** calcd for $C_{17}H_{16}BrFNO^+$ $[M+H]^+$ 348.0399 found 348.0391.

5-(4-chlorophenyl)-3-(4-fluorophenyl)piperidin-2-one (5f)

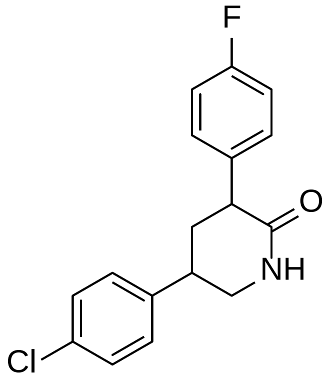

Prepared from 4-chlorostyrene and methyl 2-(4-fluorophenyl)acrylate following General procedure C to give **5f** as a white solid in 17.8 mg (59% yield) with 1.7:1 dr. Major diasteroisomer: **^{1}H NMR** (400 MHz, $CDCl_3$) δ 7.26 – 7.18 (m, 2H), 7.18– 7.12 (m, 2H), 7.12 – 7.09 (m, 2H), 6.98– 6.92 (m, 2H), 6.88 (s, 1H), 3.66 (dd, *J* = 12.4, 6.0 Hz, 1H), 3.49 – 3.43 (m, 1H), 3.39 – 3.33 (m, 1H), 3.16 (tdd, *J* = 12.0, 5.3, 2.8 Hz, 1H), 2.25 (ddt, *J* = 13.3, 5.7, 2.5 Hz, 1H), 2.16 – 2.07 (m, 1H); **^{19}F NMR** (376 MHz, $CDCl_3$) δ -115.75 – -115.82 (m, 1F); **^{13}C NMR** (101 MHz, $CDCl_3$) δ 173.0, 161.9 (d, *J* = 246.0 Hz), 139.7, 136.4 (d, *J* = 3.3 Hz), 133.1, 130.0 (d, *J* = 8.1 Hz), 129.0, 128.3, 115.6 (d, *J* = 21.5 Hz), 49.2, 48.4, 39.7, 38.2; **HR-MS (ESI)** calcd for $C_{17}H_{16}ClFNO^+$ $[M+H]^+$ 304.0904 found 304.0897.

3,5-bis(4-fluorophenyl)piperidin-2-one (5g)

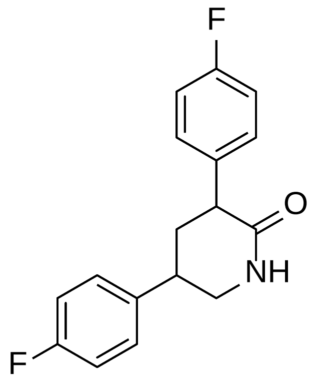

Prepared from 4-fluorostyrene and methyl 2-(4-fluorophenyl)acrylate following General procedure C to give **5g** as a white solid in 16.2 mg (56% yield) with 1.7:1 dr. Major diasteroisomer: **^{1}H NMR** (400 MHz, $CDCl_3$) δ 7.25– 7.19 (m, 4H), 7.06 – 6.97 (m, 4H), 6.66 (s, 1H), 3.73 (dd, *J* = 12.4, 6.0 Hz, 1H), 3.53 (dtd, *J* = 11.8, 4.8, 2.2 Hz, 1H), 3.46 – 3.39 (m, 1H), 3.24 (tdd, *J* = 12.1, 5.2, 2.7 Hz, 1H), 2.33 (ddt, *J* = 13.3, 5.7, 2.5 Hz, 1H), 2.23 – 2.14 (m, 1H); **^{19}F NMR** (376 MHz, $CDCl_3$) δ -115.11 – -115.19 (m, 1F), -115.81 – -115.88 (m, 1F); **^{13}C NMR** (101 MHz, $CDCl_3$) δ 172.9, 162.1 (d, *J* = 10.3 Hz), 161.2 (d, *J* = 9.8 Hz), 136.9 (d, *J* = 3.3 Hz), 136.3 (d, *J* = 3.2 Hz), 130.1 (d, *J* = 8.0 Hz), 128.4 (d, *J* = 8.0 Hz), 115.8 (d, *J* = 21.4 Hz), 115.7 (d, *J* = 21.5 Hz), 49.5, 48.4, 39.5, 38.4; **HR-MS (ESI)** calcd for $C_{17}H_{16}F_2NO^+$ $[M+H]^+$ 288.1200 found 288.1192.

3-(4-fluorophenyl)-5-(*p*-tolyl)piperidin-2-one (5h)

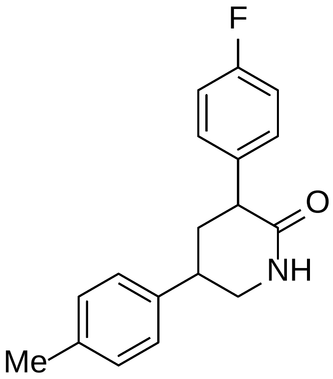

Prepared from 4-methylstyrene and methyl 2-(4-fluorophenyl)acrylate following General procedure C to give **5h** as a white solid in 15.7 mg (55% yield) with 1.8:1 dr. Major diasteroisomer: **^{1}H NMR** (600 MHz, $CDCl_3$) 7.25 – 7.19 (m, 2H), 7.16– 7.10 (m, 4H), 7.04 – 6.99 (m 2H), 6.69 (s, 1H), 3.72 (dd, *J* = 12.4, 6.0 Hz, 1H), 3.53 (dtd, *J* = 11.8, 4.7, 2.1 Hz, 1H), 3.48 – 3.42 (m, 1H), 3.21 (tdd, *J* = 11.8, 5.3, 2.8 Hz, 1H), 2.35 – 2.29 (m, 1H), 2.33 (s, 3H), 2.24 – 2.16 (m, 1H); **^{19}F NMR** (564 MHz, $CDCl_3$) δ -115.96 – -116.02 (m, 1F); **^{13}C NMR** (151 MHz, $CDCl_3$) δ 172.9, 161.9 (d, *J* = 245.1 Hz), 138.2, 137.1, 136.5 (d, *J* = 3.3 Hz), 130.1 (d, *J* = 8.1 Hz), 129.6, 126.9, 115.6 (d, *J* = 21.4 Hz), 49.6, 48.6, 40.0, 38.3, 21.1; **HR-MS (ESI)** calcd for $C_{18}H_{19}FNO^+$ $[M+H]^+$ 284.1451 found 284.1441.

3-(4-fluorophenyl)-5-(4-isopropylphenyl)piperidin-2-one (5i)

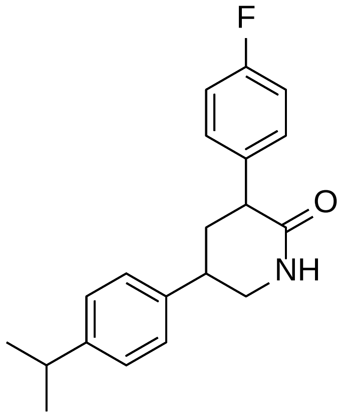

Prepared from 4-isopropylstyrene and methyl 2-(4-fluorophenyl)acrylate following General procedure C to give **5i** as a white solid in 18.6 mg (60% yield) with 2.0:1 dr. Major diasteroisomer: **^{1}H NMR** (600 MHz, $CDCl_3$) 7.25 – 7.19 (m, 2H), 7.19 – 7.15 (m, 4H), 7.04 – 6.98 (m, 2H), 6.81 (s, 1H), 3.72 (dd, *J* = 12.4, 6.1 Hz, 1H), 3.54 (dtd, *J* = 11.8, 4.6, 2.2 Hz, 1H), 3.50 – 3.42 (m, 1H), 3.22 (tdd, *J* = 11.8, 5.3, 2.8 Hz, 1H), 2.89 (hept, *J* = 6.8 Hz, 1H), 2.34 (ddt, *J* = 13.3, 5.6, 2.5 Hz, 1H), 2.24– 2.16 (m, 1H), 1.23 (d, *J* = 6.8 Hz, 6H); **^{19}F NMR** (564 MHz, $CDCl_3$) δ -115.98 – -116.06 (m, 1F); **^{13}C NMR** (151 MHz, $CDCl_3$) δ 172.9, 161.9 (d, *J* = 245.2 Hz), 148.1, 138.5, 136.5 (d, *J* = 3.3 Hz), 130.1 (d, *J* = 8.0 Hz), 126.93, 126.91, 115.6 (d, *J* = 21.4 Hz), 49.5, 48.5, 40.0, 38.4, 33.8, 24.1; **HR-MS (ESI)** calcd for $C_{20}H_{23}FNO^+$ $[M+H]^+$ 312.1764 found 312.1754.

5-(4-(*tert*-butyl)phenyl)-3-(4-fluorophenyl)piperidin-2-one (5j)

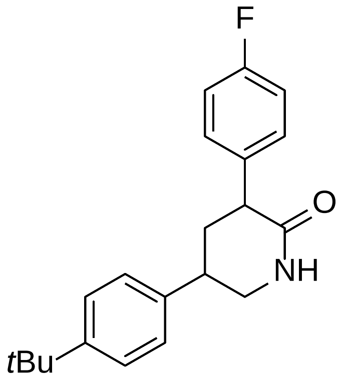

Prepared from 4-*tert*-butylstyrene and methyl 2-(4-fluorophenyl)acrylate following General procedure C to give **5j** as a white solid in 21.8 mg (67% yield) with 2.2:1 dr. Major diasteroisomer: **^{1}H NMR** (600 MHz, $CDCl_3$) δ 7.37 – 7.32 (m, 2H), 7.27 – 7.20 (m, 2H), 7.20 – 7.15 (m, 2H), 7.06 – 6.99 (m, 2H), 6.84 (s, 1H), 3.73 (dd, *J* = 12.4, 6.1 Hz, 1H), 3.55 (dtd, *J* = 9.4, 4.5, 2.4 Hz, 1H), 3.52 – 3.43 (m, 1H), 3.23 (tdd, *J* = 11.8, 5.3, 2.6 Hz, 1H), 2.34 (ddt, *J* = 13.3, 6.1, 2.6 Hz, 1H), 2.25 – 2.16 (m, 1H), 1.31 (s, 9H); **^{19}F NMR** (564 MHz, $CDCl_3$) δ -115.98 – -116.07 (m, 1F); **^{13}C NMR** (151 MHz, $CDCl_3$) δ 173.0, 161.9 (d, *J* = 245.0 Hz), 150.4, 138.2, 136.5 (d, *J* = 3.4 Hz), 130.1 (d, *J* = 8.1 Hz), 126.7, 125.8, 115.6 (d, *J* = 21.3 Hz), 49.5, 48.5, 39.9, 38.4, 34.6, 31.4; **HR-MS (ESI)** calcd for $C_{21}H_{25}FNO^+$ $[M+H]^+$ 326.1920 found 326.1911.

3-(4-fluorophenyl)-5-(4-methoxyphenyl)piperidin-2-one (5k)

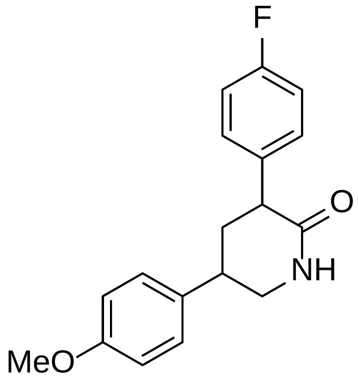

Prepared from 4-methoxystyrene and methyl 2-(4-fluorophenyl)acrylate following General procedure C to give **5k** as a white solid in 14.9 mg (50% yield) with 1.2:1 dr. Major diasteroisomer: **^{1}H NMR** (400 MHz, $CDCl_3$) δ 7.25 – 7.20 (m, 4H), 7.04 – 7.00 (m, 2H), 6.88 – 6.82 (m, 2H), 6.72 (s, 1H), 3.79 (s, 3H), 3.72 (dd, *J* = 12.4, 6.0 Hz, 1H), 3.67 – 3.62 (m, 1H), 3.46 – 3.41 (m, 1H), 3.13 – 3.06 (m, 1H), 2.35 – 2.27 (m, 1H), 2.22 – 2.14 (m, 1H); **^{19}F NMR** (376 MHz, $CDCl_3$) δ -115.98 – -116.00 (m, 1F); **^{13}C NMR** (101 MHz, $CDCl_3$) δ 172.9, 161.9 (d, *J* = 245.8 Hz), 158.8, 136.5(d, *J* = 3.3 Hz), 133.3, 130.1 (d, *J* = 8.0 Hz), 128.0, 115.6 (d, *J* = 3.1 Hz), 114.3, 55.4, 49.7, 48.8, 39.5, 33.9; **HR-MS (ESI)** calcd for $C_{18}H_{19}FNO_2^+$ $[M+H]^+$ 300.1400 found 300.1391.

3-(4-fluorophenyl)-5-(*o*-tolyl)piperidin-2-one (5l)

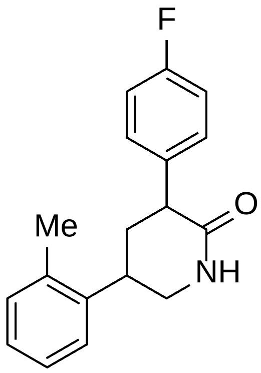

Prepared from *o*-methylstyrene and methyl 2-(4-fluorophenyl)acrylate following General procedure C to give **5l** as a white solid in 13.8 mg (49% yield) with 1.3:1 dr. Major diasteroisomer: **^{1}H NMR** (400 MHz, $CDCl_3$) δ 7.29 – 7.21 (m, 2H), 7.21 – 7.10 (m, 4H), 7.06– 7.00 (m, 2H), 3.77 – 3.72 (m, 1H), 3.53 – 3.41 (m, 3H), 2.43 – 2.40 (m, 1H), 2.42 (s, 3H), 2.31 – 2.25 (m, 1H); **^{19}F NMR** (564 MHz, $CDCl_3$) δ -115.90 – -115.97 (m, 1F); **^{13}C NMR** (151 MHz, $CDCl_3$) δ 172.8, 161.9 (d, *J* = 245.2 Hz), 139.3, 136.5 (d, *J* = 3.2 Hz), 135.8, 130.9, 130.1 (d, *J* = 7.8 Hz), 127.2, 126.7, 125.4, 115.7 (d, *J* = 21.3 Hz), 48.9, 48.6, 38.0, 36.3, 19.5; **HR-MS (ESI)** calcd for $C_{18}H_{19}FNO^+$ $[M+H]^+$ 284.1451 found 284.1441.

3-(4-fluorophenyl)-5-(*m*-tolyl)piperidin-2-one (5m)

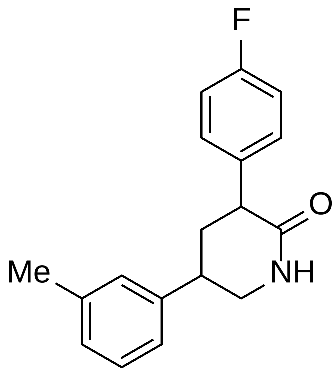

Prepared from *m*-methylstyrene and methyl 2-(4-fluorophenyl)acrylate following General procedure C to give **5m** as a white solid in 14.2 mg (50% yield) with 1.9:1 dr. Major diasteroisomer: **^{1}H NMR** (600 MHz, $CDCl_3$) δ 7.26 – 7.17 (m, 3H), 7.11 – 6.96 (m, 5H), 6.70 (s, 1H), 3.72 (dd, *J* = 12.5, 6.1 Hz, 1H), 3. 57 – 3.51 (m, 1H), 3.51 – 3.43 (m, 1H), 3.21 (tdd, *J* = 11.7, 5.3, 2.8 Hz, 1H), 2.37 – 2.30 (m, 1H), 2.34 (s, 3H), 2.26 – 2.16 (m, 1H); **^{19}F NMR** (564 MHz, $CDCl_3$) δ -115.96 – -116.02 (m, 1F); **^{13}C NMR** (101 MHz, $CDCl_3$) δ 173.0, 161.9 (d, *J* = 245.8 Hz), 141.2, 138.6, 136.5 (d, *J* = 3.3 Hz), 130.1 (d, *J* = 8.1 Hz), 128.8, 128.2, 127.8, 124.1, 115.6 (d, *J* = 21.5 Hz), 49.5, 48.5, 40.3, 38.3, 21.5; **HR-MS (ESI)** calcd for $C_{18}H_{19}FNO^+$ $[M+H]^+$ 284.1451 found 284.1442.

5-(2,5-dimethylphenyl)-3-(4-fluorophenyl)piperidin-2-one (5n)

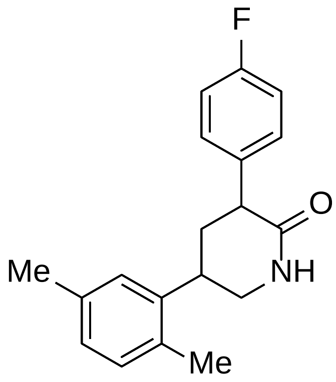

Prepared from 2,5-dimethylstyrene and methyl 2-(4-fluorophenyl)acrylate following General procedure C to give **5n** as a white solid in 15.5 mg (52% yield) with 1.5:1 dr. Major diasteroisomer: **^{1}H NMR** (600 MHz, $CDCl_3$) δ 7.30 – 7.21 (m, 2H), 7.09 – 6.92 (m, 5H), 6.68 (s, 1H), 3.75 (dd, *J* = 10.8, 7.8 Hz, 1H), 3.51 – 3.39 (m, 3H), 2.43 (ddd, *J* = 13.5, 11.4, 5.9 Hz, 1H), 2.36 (s, 3H), 2.28 (s, 3H), 2.28 – 2.25 (m, 1H); **^{19}F NMR** (564 MHz, $CDCl_3$) δ -115.96 – -116.02 (m, 1F); **^{13}C NMR** (101 MHz, $CDCl_3$) δ 173.0, 161.9 (d, *J* = 245.8 Hz), 139.0, 136.5 (d, *J* = 3.2 Hz), 136.1, 132.6, 130.7, 130.1 (d, *J* = 8.1 Hz), 127.8, 126.1, 115.6 (d, *J* = 21.5 Hz), 48.9, 48.6, 38.0, 36.2, 21.2, 19.0; **HR-MS (ESI)** calcd for $C_{19}H_{21}FNO^+$ $[M+H]^+$ 298.1607 found 298.1599.

3-(4-fluorophenyl)-5-methyl-5-phenylpiperidin-2-one (5o)

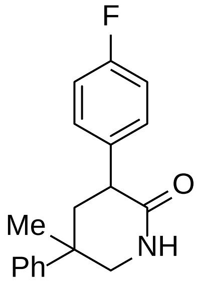

Prepared from α-methylstyrene and methyl 2-(4-fluorophenyl)acrylate following General procedure C to give **5o** as a white solid in 11.6 mg (41% yield) with 1.6:1 dr. Major diasteroisomer: **^{1}H NMR** (400 MHz, $CDCl_3$) δ 7.44 – 7.32 (m, 2H), 7.36 – 7.24 (m, 3H), 7.22 – 7.18 (m, 1H), 7.10 – 7.07 (m, 1H), 7.05 – 7.00 (m, 2H), 6.40 (s, 1H), 3.79 (dd, *J* = 12.0, 6.8 Hz, 1H), 3.71 – 3.66 (m, 1H), 3.46 (ddd, *J* = 12.0, 4.5, 2.8 Hz, 1H), 2.34 (ddd, *J* = 13.5, 6.8, 2.8 Hz, 1H), 2.25 (t, *J* = 12.9 Hz, 1H), 1.55 (s, 3H); **^{19}F NMR** (564 MHz, $CDCl_3$) δ -115.89 – -115.97 (m, 1F); **^{13}C NMR** (151 MHz, $CDCl_3$) δ 173.0, 161.9 (d, *J* = 245.4 Hz), 146.4, 136.8 (d, *J* = 3.3 Hz), 130.2 (d, *J* = 7.7 Hz), 128.8, 127.0, 125.0, 115.7 (d, *J* = 21.3 Hz); 53.1, 45.3, 43.0, 36.7, 24.0; **HR-MS (ESI)** calcd for $C_{18}H_{19}FNO^+$ $[M+H]^+$ 284.1451 found 284.1442.

3-(4-fluorophenyl)-5-methyl-5-(*p*-tolyl)piperidin-2-one (5p)

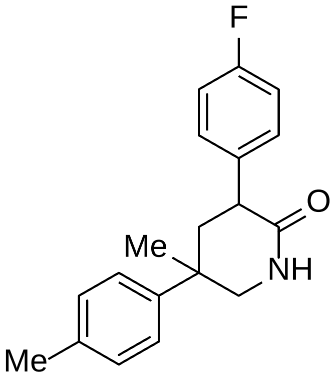

Prepared from 4,α-dimethylstyrene and methyl 2-(4-fluorophenyl)acrylate following General procedure C to give **5p** as a white solid in 17.4 mg (59% yield) with 1.5:1 dr. Major diasteroisomer: **^{1}H NMR** (400 MHz, $CDCl_3$) δ 7.32 – 7.27 (m, 2H), 7.22 – 7.17 (m, 2H), 7.13 – 7.07 (m, 2H), 7.00 – 6.94 (m, 2H), 6.44 (s, 1H), 3.86 (dt, *J* = 12.8, 3.6 Hz, 1H), 3.49 (d, *J* = 12.8 Hz, 1H), 3.12 (dd, *J* = 13.2, 6.0 Hz, 1H), 2.52 – 2.46 (m, 1H), 2.36 (s, 3H), 2.13 (t, *J* = 13.2 Hz, 1H), 1.31 (s, 3H); **^{19}F NMR** (376 MHz, $CDCl_3$) δ -116.15 – -116.23 (m, 1F); **^{13}C NMR** (101 MHz, $CDCl_3$) δ 172.8, 161.9 (d, *J* = 245.8 Hz), 140.6, 136.5 (d, *J* = 3.3 Hz), 136.46, 130.1 (d, *J* = 8.0 Hz), 129.8, 125.8, 115.7 (d, *J* = 21.4 Hz), 51.6, 45.4, 44.6, 37.7, 30.0, 21.0; **HR-MS (ESI)** calcd for $C_{19}H_{21}FNO^+$ $[M+H]^+$ 298.1607 found 298.1599.

5-(4-(allyloxy)phenyl)-3-(4-fluorophenyl)-5-methylpiperidin-2-one (5q)

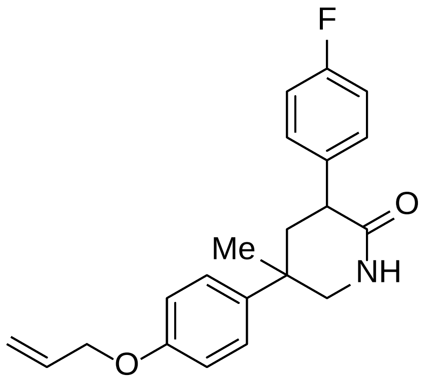

Prepared from α-methyl-4-allyloxystyrene and methyl 2-(4-fluorophenyl)acrylate following General procedure C to give **5q** as a white solid in 13.3 mg (40% yield) with 1.5:1 dr. Major diasteroisomer: **^{1}H NMR** (400 MHz, $CDCl_3$) δ 7.24 – 7.17 (m, 3H), 7.04 – 6.99 (m, 4H), 6.90 – 6.85 (m, 1H), 6.11 – 6.00 (m, 1H), 5.45 – 5.38 (m, 1H), 5.32 – 5.26 (m, 1H), 4.52 – 4.50 (m, 1H), 3.79 – 3.72 (m, 1H), 3.62 (d, *J* = 12.0 Hz, 1H), 3.42 – 3.71 (m, 1H), 2.34 – 2.28 (m, 1H), 2.25 – 2.19 (m, 1H), 1.52 (s, 3H); **^{19}F NMR** (376 MHz, $CDCl_3$) δ -115.98 – -116.06 (m, 1F); **^{13}C NMR** (101 MHz, $CDCl_3$) δ 172.7, 161.8 (d, *J* = 245.9 Hz), 157.3, 138.7, 136.9 (d, *J* = 3.3 Hz), 133.3, 130.1 (d, *J* = 8.0 Hz), 126.1, 117.8, 115.6 (d, *J* = 21.5 Hz), 114.8, 68.9, 53.4, 45.3, 43.2, 36.1, 24.0; **HR-MS (ESI)** calcd for $C_{21}H_{23}FNO_2^+$ $[M+H]^+$ 340.1713 found 340.1704.

3-(4-fluorophenyl)-5-methyl-5-(4-((3-methylbut-2-en-1-yl)oxy)phenyl) piperidin-2-one (5r)

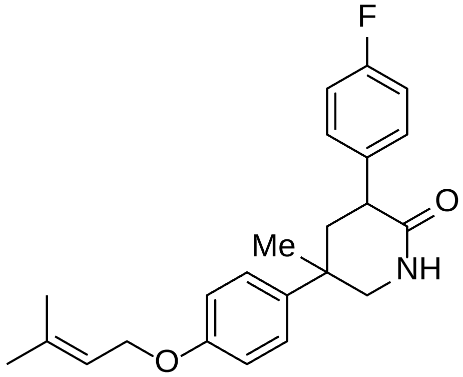

Prepared from α-methyl-4-((3-methylbut-2-en-1-yl)oxy styrene and methyl 2-(4-fluorophenyl)acrylate following General procedure C to give **5r** as a white solid in 13.6 mg (37% yield) with 1.8:1 dr. Major diasteroisomer: **^{1}H NMR** (400 MHz, $CDCl_3$) δ 7.24 – 7.18 (m, 4H), 7.03 – 6.99 (m, 2H), 6.89 – 6.85 (m, 2H), 6.75 (s, 1H), 5.49 – 5.45 (m, 1H), 4.48 (d, *J* = 6.8 Hz, 2H), 3.79 – 3.73 (m, 1H), 3.64 (d, *J* = 12.0 Hz, 1H), 3.41 (ddd, *J* = 12.4, 4.8, 2.8 Hz, 1H), 2.31 (ddd, *J* = 13.6, 6.8, 2.8 Hz, 1H), 2.25 – 2.20 (m, 1H), 1.79 (d, *J* = 1.3 Hz, 3H), 1.74 (d, *J* = 1.3 Hz, 3H), 1.52 (s, 3H); **^{19}F NMR** (376 MHz, $CDCl_3$) δ -116.03 – -116.10 (m, 1F); **^{13}C NMR** (101 MHz, $CDCl_3$) δ 172.7, 161.9 (d, *J* = 246.0 Hz), 157.6, 138.4, 136.9 (d, *J* = 3.3 Hz), 135.4, 130.1 (d, *J* = 8.0 Hz), 126.1, 119.7, 115.7 (d, *J* = 21.5 Hz), 114.7, 64.9,

53.5, 45.4, 43.3, 36.2, 25.9, 24.0, 18.3; **HR-MS (ESI)** calcd for $C_{23}H_{27}FNO_2^+$ $[M+H]^+$ 368.2026 found 368.2017.

***tert*-butyl allyl(4-(5-(4-fluorophenyl)-3-methyl-6-oxopiperidin-3-yl)phenyl) carbamate (5s)**

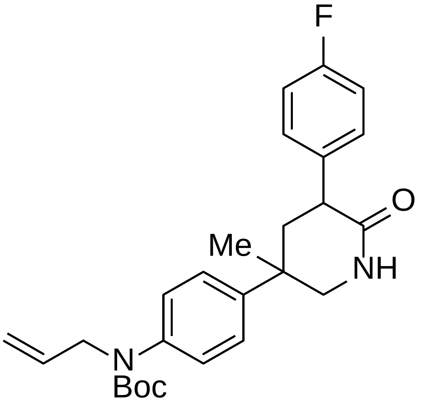

Prepared from *tert*-butyl allyl(4-(prop-1-en-2-yl)phenyl)carbamate and methyl 2-(4-fluorophenyl)acrylate following General procedure C to give **5s** as a white solid in 16.3 mg (37% yield) with 1.4:1 dr. Major diasteroisomer: **^{1}H NMR** (400 MHz, $CDCl_3$) δ 7.31 – 7.17 (m, 4H), 7.05 – 6.94 (m, 4H), 6.85 (s, 1H), 6.00 – 5.83 (m, 1H), 5.24 – 5.07 (m, 2H), 4.19 (dt, *J* = 5.5, 1.6 Hz, 2H), 3.80 – 3.72 (m, 1H), 3.65 (d, *J* = 12.2 Hz, 1H), 3.42 (ddd, *J* = 12.1, 4.6, 2.7 Hz, 1H), 2.33 (ddd, *J* = 13.5, 6.8, 2.7 Hz, 1H), 2.27 – 2.20 (m, 1H), 1.53 (s, 3H), 1.44 (s, 9H); **^{19}F NMR** (376 MHz, $CDCl_3$) δ -115.94 – -116.02 (m, 1F); **^{13}C NMR** (101 MHz, $CDCl_3$) δ 172.6, 161.9 (d, *J* = 246.3 Hz), 154.5, 143.6, 141.6, 136.8 (d, *J* = 3.4 Hz), 134.4, 130.1 (d, *J* = 8.0 Hz), 126.2, 125.3, 116.4, 115.7 (d, *J* = 21.5 Hz), 80.7, 53.2, 52.9, 45.3, 43.1, 36.5, 28.4, 23.9; **HR-MS (ESI)** calcd for $C_{26}H_{32}FN_2O_3^+$ $[M+H]^+$ 439.2397 found 439.2388.

***cis*-3-(4-fluorophenyl)-5-(4-(2-hydroxypropan-2-yl)phenyl)piperidin-2-one (5t)**

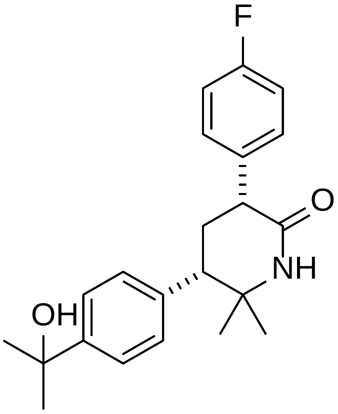

Prepared from 2-(4-(2-methylprop-1-en-1-yl)phenyl)propan-2-ol and methyl 2-(4-fluorophenyl)acrylatemethyl following General procedure A with 24 h irradiation to give **5t** as a white solid in 21.3 mg (60% yield) with 2.0:1 dr. Major diasteroisomer: **^{1}H NMR** (400 MHz, $CDCl_3$) δ 7.41 (d, *J* = 8.4 Hz, 2H), 7.24 – 7.10 (m, 4H), 7.06 – 6.95 (m, 2H), 6.16 (s, 1H), 3.68 (dd, *J* = 12.0, 6.4 Hz, 1H), 3.07 (dd, *J* = 13.6, 2.8 Hz, 1H), 2.56 – 2.46 (m, 1H), 2.17 – 2.12 (m, 1H), 1.56 (s, 6H), 1.24 (s, 3H), 1.18 (s, 3H). **^{19}F NMR** (376 MHz, $CDCl_3$) δ -115.98 – -116.05 (m, 1F); **^{13}C NMR** (101 MHz, $CDCl_3$) δ 172.1, δ 161.79 (d, *J* = 245.7 Hz),

148.2, 137.9, 136.4 (d, J = 3.2.0 Hz), 129.9 (d, J = 8.1 Hz), 128.9, 124.2, 115.6 (d, J = 21.5 Hz), 72.3, 56.5, 49.8, 48.8, 34.3, 31.8, 30.6, 25.5**;** **HR-MS (ESI)** calcd for $C_{22}H_{27}FNO_2^+$ $[M+H]^+$ 356.2020 found 356.2020.

4a-((trimethylsilyl)oxy)octahydroquinolin-2(1*H*)-one (6a)

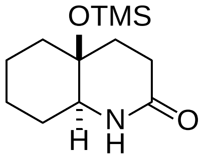

Prepared from (cyclohex-1-en-1-yloxy)trimethylsilane and methyl acrylate following General procedure A with 24 h irradiation to give **6a** as a white solid in 10.0 mg (41% yield) with 3.0:1 dr.. Major diasteroisomer: **^{1}H NMR** (400 MHz, $CDCl_3$) δ 6.01 (s, 1H), 3.04 (dd, J = 12.0, 4.0 Hz, 1H), 2.51 – 2.47 (m, 1H), 2.36 – 2.31 (m, 1H), 1.89 – 1.79 (m, 3H), 1.71 – 1.59 (m, 3H), 1.54 – 1.48 (m, 1H), 1.44 – 1.39 (m, 1H), 1.31 – 1.23 (m, 2H), 0.14 (s, 9H); **^{13}C NMR** (101 MHz, $CDCl_3$) δ 172.0, 71.1, 60.0, 36.4, 34.0, 28.3, 27.4, 24.1, 21.2, 2.4; **HR-MS (ESI)** calcd for $C_{12}H_{24}NO_2Si^+$ $[M+H]^+$ 242.1576 found 242.1567.

(3*aS*,5*S*,7*aR*)-5-(4-methoxyphenyl)hexahydrofuro[2,3-*b*]pyridin-6(2*H*)-one (6b)

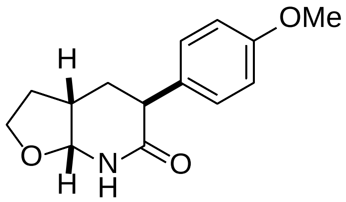

Prepared from 2,3-dihydrofuran and methyl 2-(4-methoxyphenyl)acrylate following General procedure A with 24 h irradiation to give **6b** as a white solid in 18.0 mg (73% yield). **^{1}H NMR** (600 MHz, $CDCl_3$) δ 7.15 – 7.10 (m, 2H), 6.87 (d, J = 8.6 Hz, 2H), 6.41 (s, 1H), 5.21 (dd, J = 6.5, 2.6 Hz, 1H), 3.87 – 3.82 (m, 1H), 3.78 (s, 3H), 3.66 – 3.63 (m, 2H), 2.67 – 2.60 (m, 1H), 2.20 – 2.14 (m, 1H), 2.13 – 2.06 (m, 2H), 1.92 – 1.86 (m, 1H); **^{13}C NMR** (151 MHz, $CDCl_3$) δ 173.5, 158.7, 131.3, 129.3, 114.2, 85.5, 66.1, 55.4, 43.6, 34.0, 31.9, 29.9; **HR-MS (ESI)** calcd for $C_{14}H_{18}NO_3^+$ $[M+H]^+$ 248.1287 found 248.1277.

***cis*-5-(*tert*-butoxy)-3-(4-fluorophenyl)piperidin-2-one (6c)**

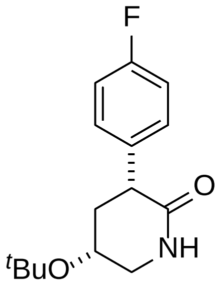

Prepared from *tert*-butyl vinyl ether and methyl 2-(4-fluorophenyl)acrylate following General procedure C to give **6c** as a white solid in 16.4 mg (62% yield) with 2.4:1 dr. Major diasteroisomer: **^{1}H NMR** (600 MHz, $CDCl_3$) δ 7.21 (dd, *J* = 8.4, 5.3 Hz, 2H), 7.04– 6.98 (m, 2H), 6.46 (s, 1H), 4.01 – 3.94 (m, 1H), 3.90 (dd, *J* = 10.0, 5.9 Hz, 1H), 3.51 (ddd, *J* = 12.5, 4.4, 1.8 Hz, 1H), 3.30 – 3.25 (m, 1H), 2.15 – 2.09 (m, 1H), 2.03 (ddd, *J* = 13.2, 10.0, 3.0 Hz, 1H), 1.17 (s, 9H); **^{19}F NMR** (564 MHz, $CDCl_3$) δ -116.25 – -116.33 (m, 1F); **^{13}C NMR** (151 MHz, $CDCl_3$) δ 173.1, 161.8 (d, *J* = 244.7 Hz), 136.5 (d, *J* = 3.3 Hz), 130.0 (d, *J* = 8.1 Hz), 115.5 (d, *J* = 21.3 Hz), 74.5, 62.1, 49.4, 43.7, 37.7, 28.4; **HR-MS (ESI)** calcd for $C_{15}H_{21}FNO_2^+$ $[M+H]^+$ 266.1556 found 266.1547.

(4*aS*,7a*S*)-3-(4-fluorophenyl)-4*a*-methyloctahydro-2*H*-cyclopenta[*b*]pyridin-2-one (6d)

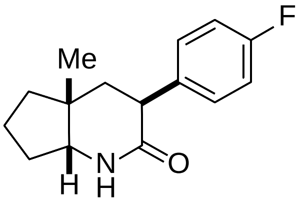

Prepared from 1-methylcyclopentene and methyl 2-(4-fluorophenyl)acrylate following General procedure C to give **6d** as a white solid in 13.0 mg (53% yield) with 1.5:1 dr. Major diasteroisomer: **^{1}H NMR** (400 MHz, $CDCl_3$) δ 7. 21 – 7.14 (m, 2H), 7.04 – 6.96 (m, 2H), 6.07 (s, 1H), 3.51 (dd, *J* = 12.9, 5.4 Hz, 1H), 3.36 (td, *J* = 7.3, 2.8 Hz, 1H), 2.15 – 2.09 (m, 1H), 2.03 – 1.97 (m, 1H), 1.87 – 1.73 (m, 3H), 1.69 – 1.64 (m, 1H), 1.64 – 1.59 (m, 1H), 1.59 – 1.53 (m, 1H), 1.25 (s, 3H); **^{19}F NMR** (376 MHz, $CDCl_3$) δ -116.28 – -116.38 (m, 1F); **^{13}C NMR** (101 MHz, $CDCl_3$) 173.6, 161.9 (d, *J* = 245.7 Hz), 136.2 (d, *J* = 3.3 Hz), 130.1 (d, *J* = 7.9 Hz), 115.4 (d, *J* = 21.5 Hz), 62.4, 44.7, 41.3, 40.8, 40.1, 35.1, 25.7, 21.7; **HR-MS (ESI)** calcd for $C_{15}H_{19}FNO^+$ $[M+H]^+$ 248.1451 found 248.1441.

5,5-dibutyl-3-(4-fluorophenyl)-6-methylpiperidin-2-one (6e)

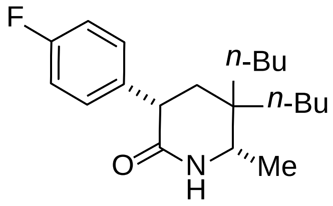

Prepared from 5-ethylidenenonane and methyl 2-(4-fluorophenyl)acrylate following General procedure C to give **6e** as a white solid in 18.0 mg (56% yield) with 2.9:1 dr. Major diasteroisomer: **^{1}H NMR** (600 MHz, $CDCl_3$) δ 7.19 – 7.12 (m, 2H), 7. 03 – 6.97 (m, 2H), 6.37 (s, 1H), 3.48 (dd, *J* = 12.4, 7.2 Hz, 1H), 3.34 – 3.28 (m, 1H), 1.80 (ddd, *J* = 14.0, 7.2, 1.4 Hz, 1H), 1.73 (dd, *J* = 14.2, 12.4 Hz, 1H), 1.64 (ddd, *J* = 14.2, 11.5, 5.6 Hz, 1H), 1.47 (ddd, *J* = 13.9, 11.4, 5.3 Hz, 1H), 1.39 – 1.32 (m, 3H), 1.30 – 1.25 (m, 5H), 1.24 (d, *J* = 6.6 Hz, 3H), 1.22 – 1.13 (m, 2H), 0.95 (t, *J* = 7.3 Hz, 3H), 0.89 (t, *J* = 7.1 Hz, 3H); **^{19}F NMR** (564 MHz, $CDCl_3$) δ -116.31 – -116.37 (m, 1F); **^{13}C NMR** (151 MHz, $CDCl_3$) 172.9, 161.8 (d, *J* = 244.8 Hz), 137.1 (d, *J* = 3.3 Hz), 130.0 (d, *J* = 8.1 Hz), 115.6 (d, *J* = 21.3 Hz), 55.4, 44.6, 37.3, 36.9, 34.6, 32.5, 25.4, 24.9, 23.53, 23.49, 19.6, 14.3, 14.1; **HR-MS (ESI)** calcd for $C_{20}H_{31}FNO^+$ $[M+H]^+$ 320.2390 found 320.2380.

3-(4-fluorophenyl)-5,5-dimethyl-6-propylpiperidin-2-one (6f)

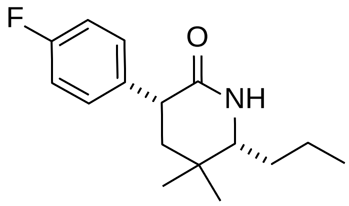

Prepared from 2-methylhex-2-ene and methyl 2-(4-fluorophenyl)acrylate following General procedure C to give **6f** as a white solid in 13.7 mg (52% yield) with 1.7:1 dr. Major diasteroisomer: **^{1}H NMR** (600 MHz, $CDCl_3$) δ 7.21 – 7.13 (m, 2H), 7.03 – 6.97 (m, 2H), 5.91 (s, 1H), 3.62 – 3.55 (m, 1H), 3.21 (dd, *J* = 10.4, 1.7 Hz, 1H), 1.79 – 1.73 (m, 1H), 1.63 – 1.59 (m, 1H), 1.54 – 1.48 (m, 1H), 1.45 – 1.40 (m, 1H), 1.35 – 1.30 (m, 1H), 1.27 – 1.24 (m, 1H), 1.19 (s, 3H), 1.01 (s, 3H), 0.97 (t, *J* = 7.3 Hz, 3H); **^{19}F NMR** (564 MHz, $CDCl_3$) δ -116.25 – -116.32 (m, 1F); **^{13}C NMR** (151 MHz, $CDCl_3$) δ 172.8, 161.8 (d, *J* = 243.3 Hz), 136.8 (d, *J* = 3.3 Hz), 130.0 (d, *J* = 7.9 Hz), 115.6 (d, *J* = 21.3 Hz), 62.1, 47.0, 45.3, 33.0, 32.5, 27.2, 19.8, 18.7, 14.0; **HR-MS (ESI)** calcd for $C_{16}H_{23}FNO^+$ $[M+H]^+$ 264.1764 found 264.1753.

3-(4-fluorophenyl)-5-((8*R*,9*S*,13*S*,14*S*)-13-methyl-17-oxo-7,8,9,11,12,13,14,15,16,17-decahydro-6*H*-cyclopenta[*a*]phenanthren-3-yl)piperidin-2-one (7a)

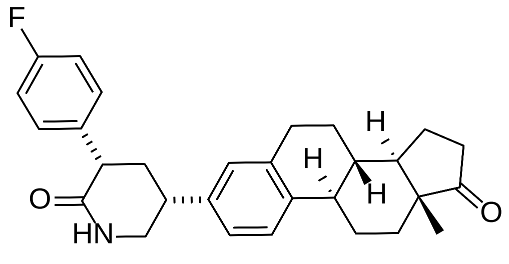

Prepared from 3-deoxy-3-vinylestrone and methyl 2-(4-fluorophenyl)acrylate following General procedure C to give **7a** as a white solid in 26.8 mg (60% yield) with 1.7:1 dr. Major diasteroisomer: **^{1}H NMR** (400 MHz, $CDCl_3$) δ 7.26 – 7.19 (m, 3H), 7.07 – 6.95 (m, 4H), 6.22 (s, 1H), 3.74 – 3.70 (m, 1H), 3.57 – 3.51 (m, 1H), 3.51 – 3.45 (m, 1H), 3.24 – 3.17 (m, 1H), 2.91 – 2.88 (m, 1H), 2.54 – 2.47 (m, 1H), 2.45 – 2.37 (m, 1H), 2.36 – 2.25 (m, 2H), 2.22 – 2.11 (m, 2H), 2.08– 1.99 (m, 2H), 1.99 – 1.92 (m, 2H), 1.67– 1.56 (m, 2H), 1.56 – 1.40 (m, 4H), 0.90 (s, 3H); **^{19}F NMR** (376 MHz, $CDCl_3$) δ -115.93 – -116.01 (m, 1F); **^{13}C NMR** (151 MHz, $CDCl_3$) δ 220.9, 172.7, 161.9 (d, *J* = 245.3 Hz), 139.0, 138.7, 137.1, 136.54 – 136.46 (m), 130.1 (d, *J* = 7.9 Hz), 127.6 (d, *J* = 4.3 Hz), 115.6 (d, *J* = 21.5 Hz), 125.9, 124.5 (d, *J* = 3.5 Hz), 66.0, 50.6, 49.5, 48.5, 48.1, 44.4, 39.9, 38.2, 36.0, 31.7, 29.6, 26.6, 25.8, 21.7, 14.0; **HR-MS (ESI)** calcd for $C_{29}H_{33}FNO_2^+$ $[M+H]^+$ 446.2495 found 446.2486.

3-(4-fluorophenyl)-5-(((1*R*,2*S*,5*R*)-2-isopropyl-5-methylcyclohexyl)oxy)piperidin-2-one (7b)

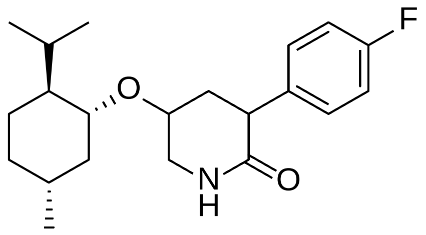

Prepared from (-)menthyl vinyl ether and methyl 2-(4-fluorophenyl)acrylate following General procedure C to give **7b** as a white solid in 21.4 mg (62% yield) with 1.4:1:2.1:1.4 dr. Major diasteroisomer: **^{1}H NMR** (400 MHz, $CDCl_3$) δ 7.23 – 7.16 (m, 2H), 7.05 – 6.98 (m, 2H), 6.53 (s, 1H), 3.94 – 3.87 (m, 2H), 3.85 (dd, *J* = 11.1, 5.8 Hz, 1H), 3.62 – 3.56 (m, 1H), 3.48 – 3.45 (m, 1H), 3.16 (ddd, *J* = 14.3, 8.5, 3.5 Hz, 1H), 2.39 – 2.32 (m, 1H), 2.26 (dddd, *J* = 17.4, 12.9, 8.9, 5.1 Hz, 1H), 2.14 (dtd, *J* = 12.2, 7.5, 7.0, 2.2 Hz, 1H), 2.08 – 1.98 (m, 1H), 1.98 – 1.88 (m, 1H), 1.69 – 1.59 (m, 2H), 1.33 (tdq, *J* = 12.9, 6.6, 3.7, 3.2 Hz, 1H), 1.30 – 1.21 (m, 2H), 1.16 (ddq, *J* = 13.2, 10.0, 3.0 Hz, 1H), 0.91 (d, *J* = 6.6 Hz, 6H), 0.78 (d, *J* = 7.0 Hz, 3H); **^{19}F NMR** (564 MHz, $CDCl_3$) δ -116.15 – -116.22 (m, 1F); **^{13}C NMR** (151 MHz, $CDCl_3$) δ 172.9,

161.9 (d, J = 244.8 Hz), 136.0 (d, J = 3.0 Hz), 130.1 (d, J = 7.9 Hz), 115.6 (d, J = 21.4 Hz), 77.6, 67.2, 48.63, 48.60, 43.3, 41.5, 35.0, 34.5, 31.7, 25.5, 23.1, 22.4, 21.4, 16.1; **HR-MS (ESI)** calcd for $C_{21}H_{31}FNO_2^+$ $[M+H]^+$ 348.2339 found 348.2328.

(4*aR*,5*R*,6*aS*,9*R*,9*aR*)-3-(4-fluorophenyl)-4a,6,6,9-tetramethyldecahydro-1*H*-5,9*a*-methanoazuleno[5,6-*b*]pyridin-2(3*H*)-one (7c)

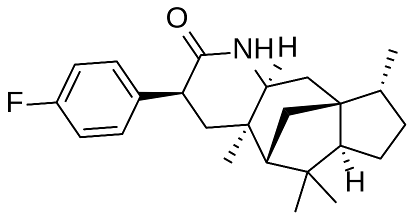

Prepared from α-cedrene and methyl 2-(4-fluorophenyl)acrylate following General procedure C to give **7c** as a white solid in 17.7 mg (48% yield). **^{1}H NMR** (400 MHz, $CDCl_3$) 7.20 – 7.12 (m, 2H), 7.07 – 6.98 (m, 2H), 6.51 (s, 1H), 3.71 (dd, J = 12.3, 6.9 Hz, 1H), 3.55 – 3.45 (m, 1H), 2.38 (t, J = 13.0 Hz, 1H), 1.94 – 1.86 (m, 2H), 1.80 – 1.74 (m, 1H), 1.74 – 1.66 (m, 2H), 1.62 – 1.47 (m, 5H), 1.47 – 1.40 (m, 2H), 1.37 (s, 3H), 1.30 (s, 3H), 1.04 (s, 3H), 0.88 (d, J = 7.1 Hz, 3H); **^{19}F NMR** (564 MHz, $CDCl_3$) δ -116.24 – -116.31 (m, 1F); **^{13}C NMR** (151 MHz, $CDCl_3$) δ 173.0, 161.8 (d, J = 245.1 Hz), 137.5 (d, J = 3.3 Hz), 130.1 (d, J = 8.2 Hz), 115.7 (d, J = 21.5 Hz), 58.9, 57.0, 56.4, 54.2, 45.0, 44.9, 42.2, 41.3, 40.3, 38.4, 36.6, 36.5, 30.02, 30.00, 25.4, 23.0, 15.8; **HR-MS (ESI)** calcd for $C_{24}H_{33}FNO^+$ $[M+H]^+$ 370.2546 found 370.2536.

Mechanistic studies

Control experiments

Supplementary Table 9. Control experiments

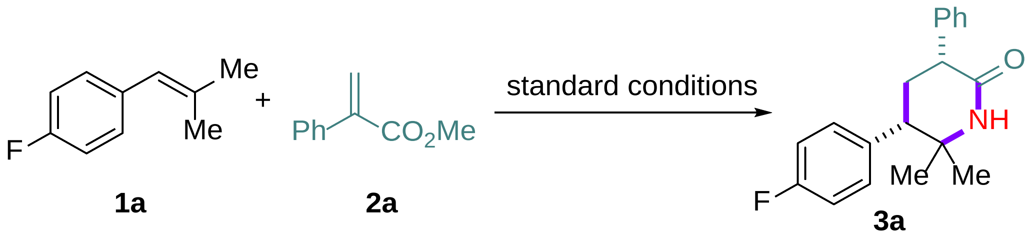

1a **2a** **3a**

Entry	variations	**1a** conv.	**2a** conv.	yield of **3a** (dr)
1	standard conditions	99%	87%	88% (3.7:1)

2	no PC	17%	60%	15% (3.6:1)
3	no light	Trace	57%	N.D.
4	no PC, no light	trace	47%	N.D.

The reaction was performed on 0.1 mmol of 4-fluoro-β,β-dimethylstyrene (**1a**), 0.2 mmol of methyl 2-phenylacrylate (**2a**) with 0.3 mmol of NH_4OAc and 0.1 mmol of $LiBF_4$ in a mixture of CH_3CN (1.0 mL) and chlorobenzene (0.1 mL) under irradiation with 30 W blue LED for 24 h at ambient temperature. Yield was determined by ^{1}H NMR analysis of the crude mixture using PhTMS as internal standard. N.D. stands for not detected.

Radical quench experiment

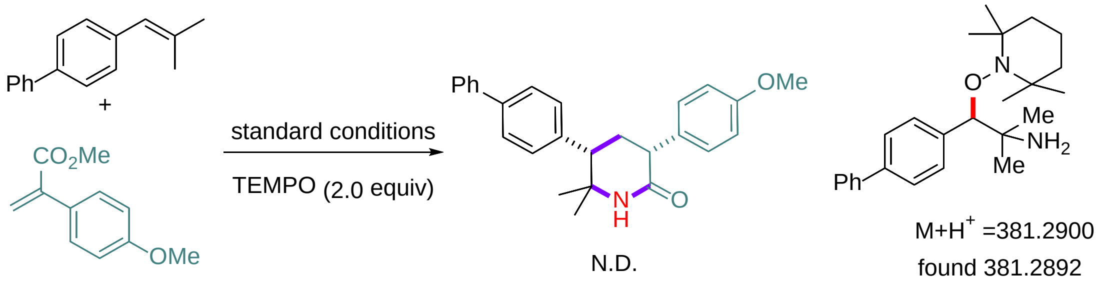

An oven-dried Schlenk-tube equipped with a magnetic stir bar was charged with *N*-Ph-9-mesityl acridinium tetrafluoroborate (2.9 mg, 5.0 μmol, 5.0 mol%), NH_4OAc (23.1 mg, 0.3 mmol), $LiBF_4$ (9.4 mg, 0.1 mmol) and 2,2,6,6-tetramethyl-1-piperidinyloxy (TEMPO, 31.2 mg, 0.2 mmol, 2.0 eq.). CH_3CN (1.0 mL), 4-(2-methylprop-1-en-1-yl)-1,1'-biphenyl (12.2 mg, 0.1 mmol), methyl 2-(4-methoxyphenyl)acrylate (36.0 mg, 0.2 mmol, 2.0 eq.) and PhCl (0.1 mL) were added consecutively via syringe. The tube was sealed with a Teflon-coated septum cap, and stirred at ambient temperature under irradiation with 30W blue LEDs for 24 h. Upon completion, the reaction mixture was quenched with water and extracted with ethyl acetate. The combined organic phase was concentrated in vacumn. No desired “1+2+3” product was obtained and the TEMPO-trapped product was detected by HR-MS analysis (found 381.2892)

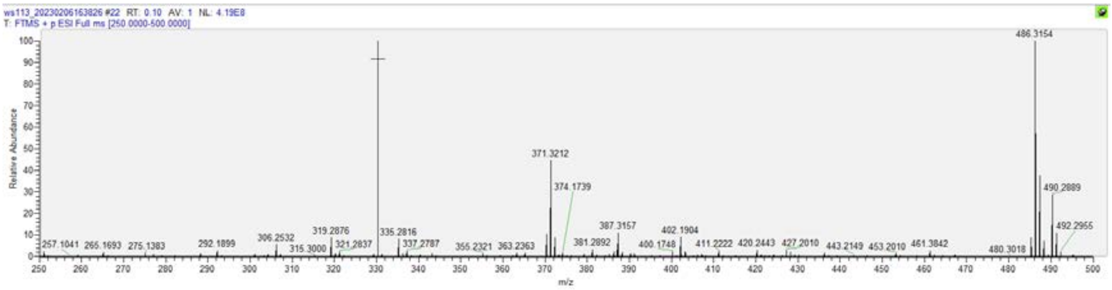

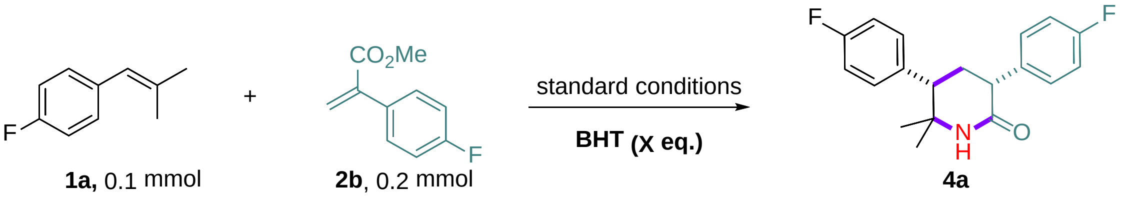

condition 1
X = 5.0, 27%, 2.0 :1 dr
condition 2
X = 10.0, N.D.

An oven-dried Schlenk-tube equipped with a magnetic stir bar was charged with *N*-Ph-9-mesityl acridinium tetrafluoroborate (2.9 mg, 5.0 µmol, 5.0 mol%), NH_4OAc (23.1 mg, 0.3 mmol), $LiBF_4$ (9.4 mg, 0.1 mmol) and 2,6-di-*tert*-butyl-4-methylphenol (BHT, X eq.). CH_3CN (1.0 mL), **1a** (15.0 mg, 0.1 mmol, 1.0 eq.), methyl 2-(4-fluorophenyl)acrylate **2b** (36.0 mg, 0.2 mmol, 2.0 eq.) and PhCl (0.1 mL) were added consecutively via syringe. The tube was sealed with a Teflon-coated septum cap, and stirred at ambient temperature under irradiation with 30W blue LEDs for 24 h. The reaction mixture was quenched with water and extracted with ethyl acetate. The combined organic phase was concentrated in vacumn. The residue was analyzed by ^1H NMR with PhTMS as internal standard to determine 46% conversion of **1a** with 27% yield of product **4a** obtained with 2.0:1 dr when 5.0 eq. BHT was used. No desired product **4a** was obtained when 10.0 eq. BHT was used.

Control experiment with methacrylamide

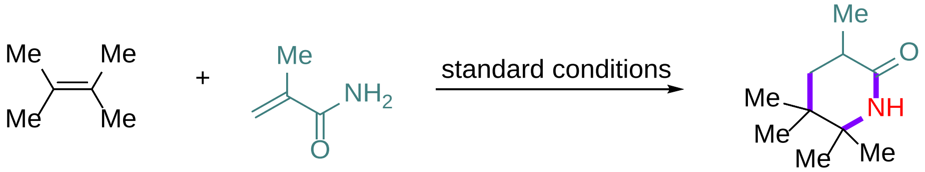

1b **3l**

N.D.

An oven-dried Schlenk-tube equipped with a magnetic stir bar was charged with *N*-Ph-9-mesityl 3,6-di-*tert*-butylacridinium tetrafluoroborate (1.4 mg, 2.5 μmol, 2.5 mol%), NH_4OAc (23.1 mg, 0.3 mmol), $LiBF_4$ (9.4 mg, 0.1 mmol), CH_3CN (1.0 mL), 2,3-dimethylbut-2-ene **1b** (8.4 mg, 0.1 mmol), methacrylamide (17.0 mg, 0.2 mmol) and PhCl (0.1 mL) were added consecutively via syringe. The tube was sealed with a Teflon-coated septum cap, and stirred at ambient temperature under irradiation with 30W blue LEDs for 12 h. Upon completion, the reaction mixture was quenched with water and extracted with ethyl acetate. The combined organic phase was concentrated in vacumn. The crude mixture was analyzed by ^1H NMR with PhTMS as an internal standard to determine the conversion of methacrylamide as 47% while no desired product **3l** obtained.

Light on-off experiment

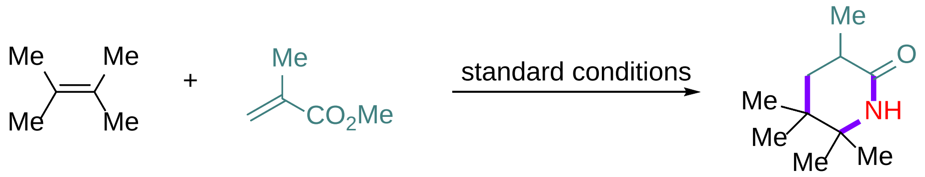

1b **2k** **3l**

Parellel reactions were conducted. An oven-dried Schlenk-tube equipped with a magnetic stir bar was charged with *N*-Ph-9-mesityl 3,6-di-*tert*-butylacridinium tetrafluoroborate (1.4 mg, 2.5 μmol, 2.5 mol%), NH_4OAc (23.1 mg, 0.3 mmol), $LiBF_4$ (9.4 mg, 0.1 mmol), CH_3CN (1.0 mL), 2,3-dimethylbut-2-ene **1b** (8.4 mg, 0.1 mmol), methyl methacrylate **2k** (20.0 mg, 0.2 mmol) and PhCl (0.1 mL) were added consecutively via syringe. The tube was sealed with a Teflon-coated septum cap, and

stirred at ambient temperature under irradiation with 30W blue LEDs for indicated time. The light was turned on and off every half an hour. Upon the indicated time, the reaction mixture was quenched with water and extracted with ethyl acetate. The combined organic phase was concentrated in vacumn. The crude mixture was analyzed by ^{1}H NMR with PhTMS as an internal standard to determine the yield of **3l**.

Supplementary Table 10. Data of light on-off experiment

time (h)	yield of **3l**	light
0	0	/
0.5	15%	on
1.0	15%	off
1.5	23%	on
2.0	23%	off
2.5	27%	on
3.0	27%	off

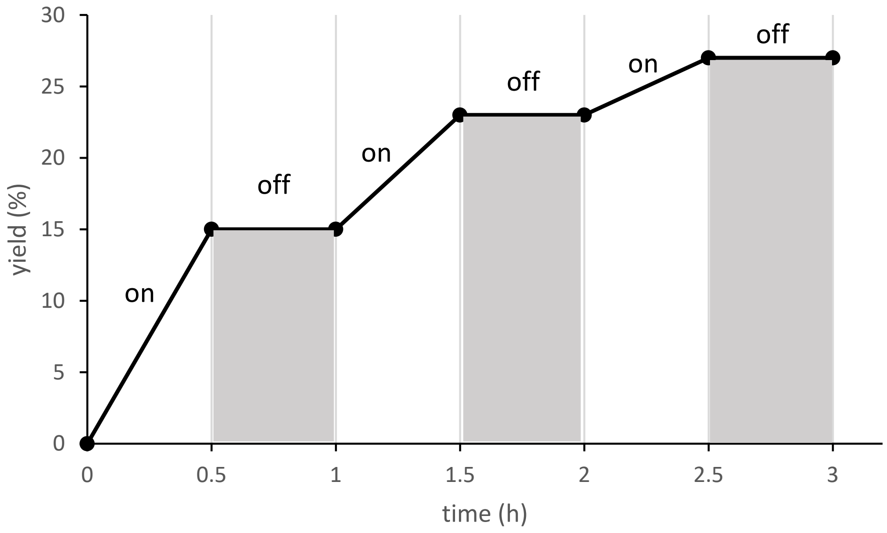

Supplementary Figure 1 Light on-off experiment with **1b** and **2k**

Time course study

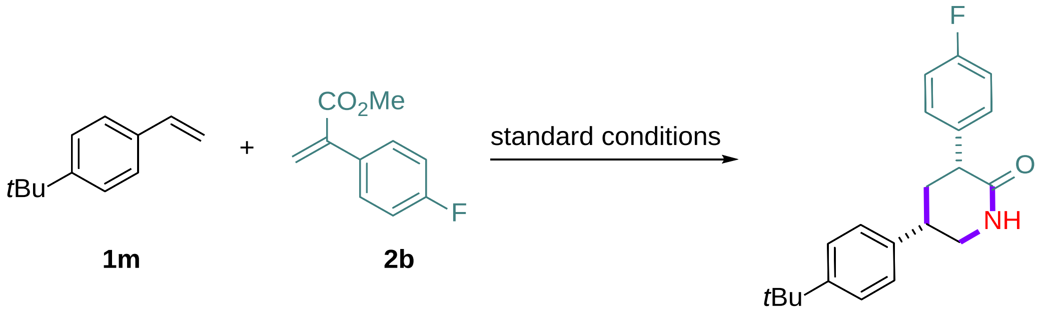

1m 2b 5j

An oven-dried Schlenk-tube equipped with a magnetic stir bar was charged with *N*-Ph-9-mesityl acridinium tetrafluoroborate (2.9 mg, 5.0 μmol, 5.0 mol%), NH_4OAc (23.1 mg, 0.3 mmol) and $LiBF_4$ (9.4 mg, 0.1 mmol). CH_3CN (10.0 mL), 4-*tert*-butylstyrene (16.0 mg, 0.1 mmol), methyl 2-(4-fluorophenyl)acrylate **2b** (36.0 mg, 0.2 mmol) and PhCl (0.1 mL) were added consecutively via syringe. The tube was sealed with a Teflon-coated septum cap, and stirred at ambient temperature under irradiation with 30W blue LEDs for indicated time. Upon completion, the reaction mixture was quenched with water and extracted with ethyl acetate. The combined organic phase was concentrated in vacumn. The residue was analyzed by ^{1}H NMR with PhTMS as internal standard to determine the conversion and yield of **5j**.

Supplementary Table 11. Time course study of **1m** and **2b** to **5j**

entry	time (h)	rsm of **1m**	rsm of **2b**	yield of **5j**
1	0	100%	100%	0
2	1.0	26%	74%	20%
3	3.0	8%	63%	48%
4	5.0	3%	61%	77%
5	7.0	1%	59%	79%
6	9.0	1%	54%	86%
7	12.0	1%	51%	89%

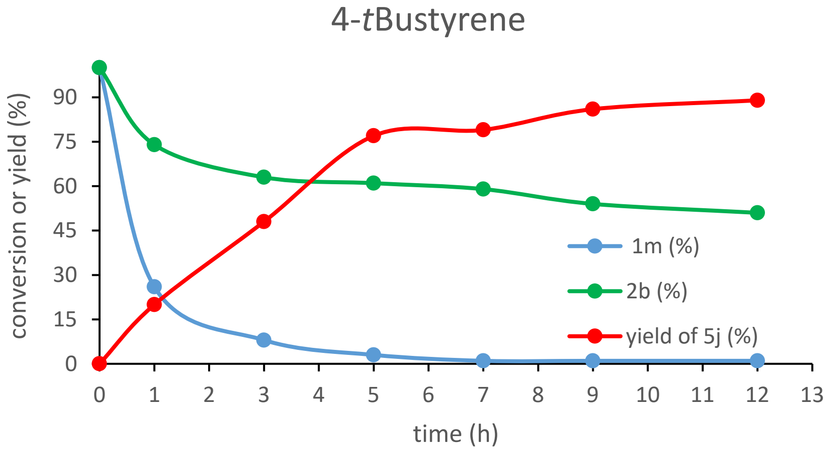

Supplementary Figure 2 Time course study of **1m** and **2b** to **5j**

Stern-Volmer quench analysis

The photocatalyst, potential quenchers and CH_3CN/PhCl (10:1) were weighed into vials inside a glovebox under nitrogen. The emission spectra were recorded using an Edinburgh FLS980 spectrometer. Mes-3,6-*t*Bu_2-Acr-$Ph^+BF_4^-$ was excited at 430 nm and the emission intensity was collected at 519 nm. In a typical experiment, to a $2.5*10^{-3}$ M solution of Mes-3,6-*t*Bu_2-Acr-$Ph^+BF_4^-$ in CH_3CN/PhCl (10:1) was added the appropriate amount of alkene **1a** or **2b** or $LiBF_4$ in a screw-top quartz cuvette, the emission of the sample was collected.

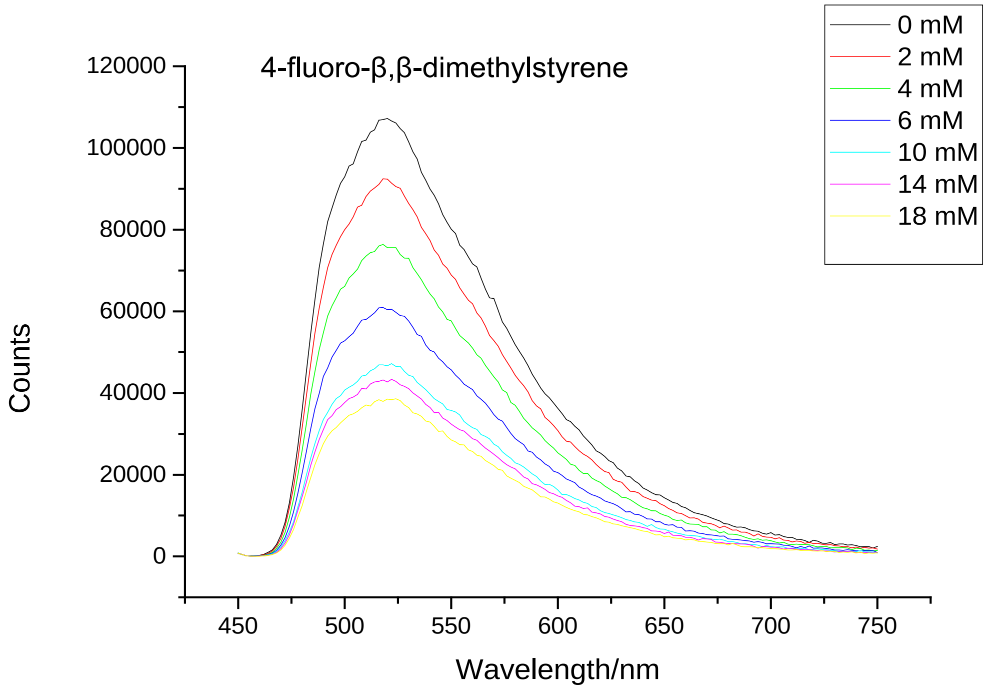

Supplementary Figure 3 Dynamic Quenching of **1a**

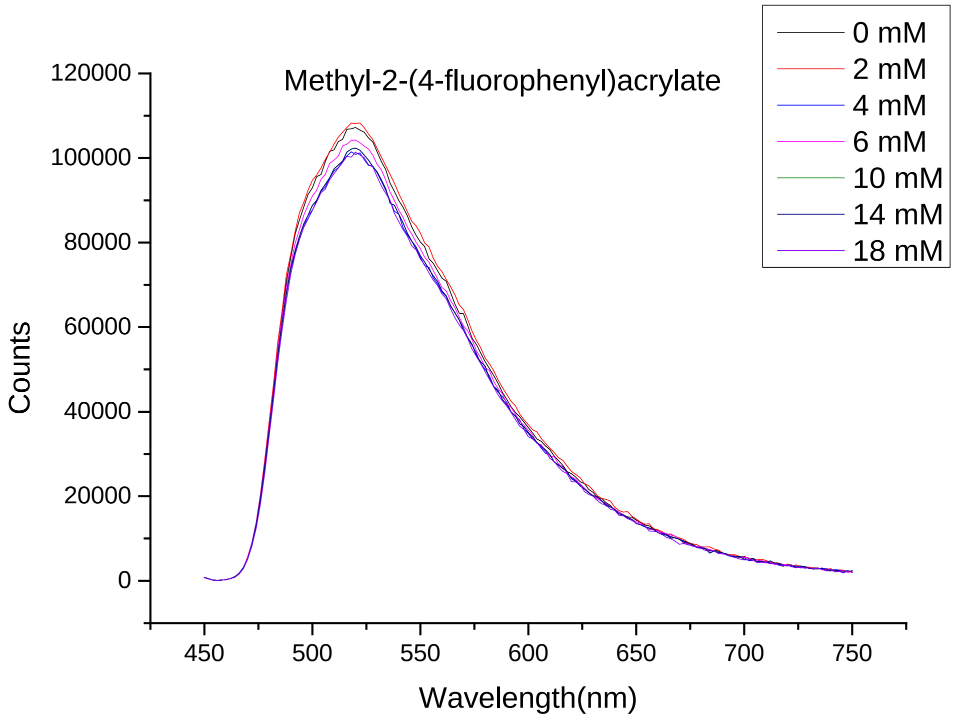

Supplementary Figure 4 Dynamic Quenching of **2b**

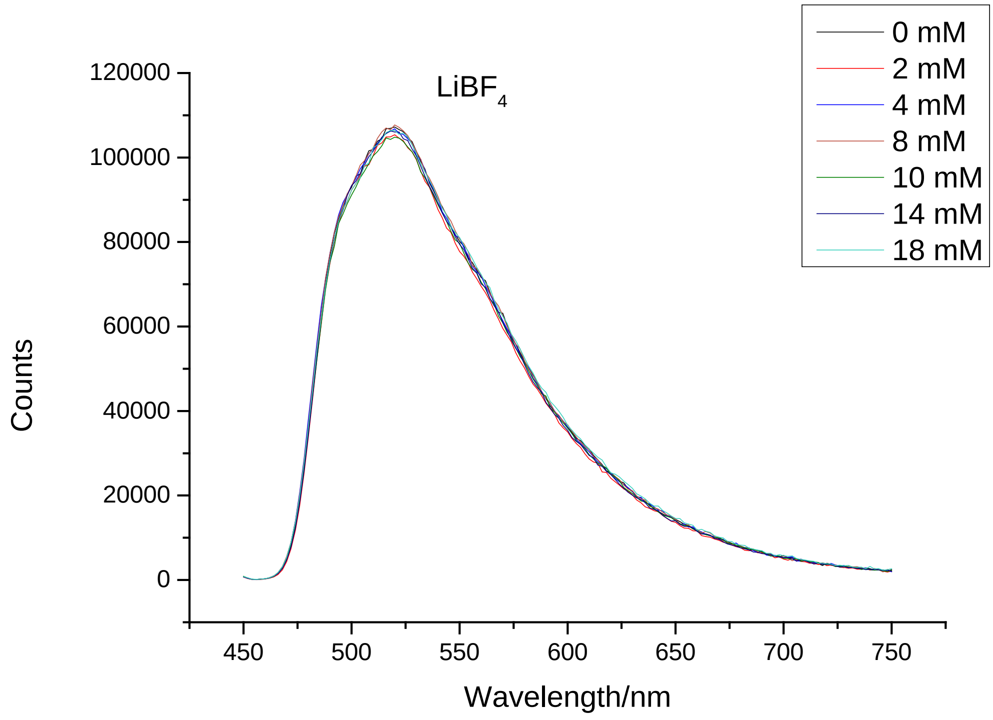

Supplementary Figure 5 Dynamic Quenching of $LiBF_4$

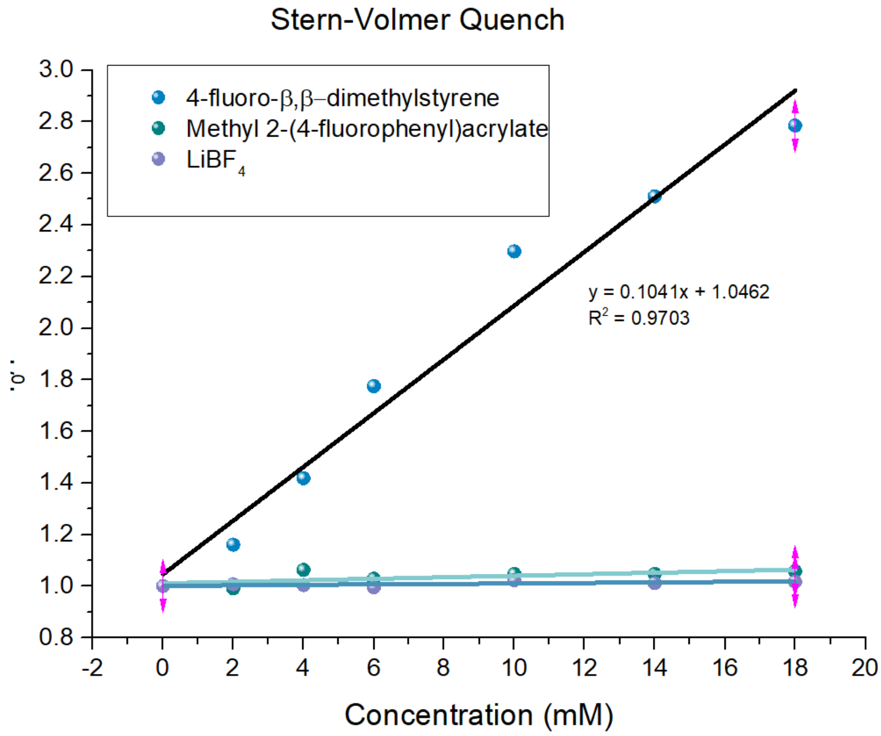

Supplementary Figure 6 Stern-Volmer plot for quenching of **1a**, **2b** and $LiBF_4$

Stern-Volmer analysis indicated that this reaction proceeds through a reductive quenching mechanism of Mes-3,6-*t*-Bu_2-Acr-$Ph^+BF_4^-$ by alkene 4-fluoro-β,β-dimethylstyrene.

Time-resolved fluorescence measurements

All photophysical measurements were taken in 4 mL quartz cells sealed with a silicone rubber-lined screw cap purchased from Starna Cells, Inc. Solutions were made by dilution and thorough mixing of freshly prepared stock solutions of each component. Emission lifetime measurements were obtained at ambient temperature using an Edinburgh PLS1000 fluorescence spectrometer. Measurements were made using the time-correlated single photon counting (TCSPC) capability of the instrument with pulsed excitation light (510 nm, pulse width = 95 ps) generated by an Edinburgh EPL-445 pulsed laser diode. Each data set was collected for 6 minutes of counting. Raw data was fit to a single term exponential decay using the Curve Fitting tool on MatLabR2019a.

$$I_t = I_0 e^{-\frac{t}{\tau}} \qquad (1)$$

where I is counts, t is time, and τ is the mean lifetime of fluorescence. Excited state lifetimes were determined by integrating the exponential fit obtained.

$$\ln(I_t) = -\frac{t}{\tau} + \ln(I_0) \qquad (2)$$

The traditional Stern-Volmer relationship is:[13]

$$\frac{k}{k_0} = 1 + K_{SV}[Q] = 1 + \tau_0 k_q[Q] \qquad (3)$$

where k and k_0 is the rate of fluorescence decay in the presence and absence of quencher, K_{SV} is the SternVolmer constant, τ_0 is the fluorescence lifetime in the absence of quencher, k_q is the rate of bimolecular quenching, and [Q] is the concentration of quencher. Since rates of fluorescence decay are related to fluorescence lifetimes, a modified form of this equation was employed to determine bimolecular quenching constants (k_q) as previously seen in our laboratory and others.[14,15]

$$\frac{\tau_0}{\tau} = 1 + K_{SV}[Q] = 1 + \tau_0 K_q[Q] \qquad (4)$$

General Procedure for the Determination of Bimolecular Quenching Constants

A 25 μM solution of catalyst was prepared in the specified solvent. 2 mL were transferred to a quartz cuvette, and sealed with a silicone rubber-lined cap. The system was sparged with argon for 10 minutes. The fluorescence lifetime was measured without quencher. Separately, a 0.5 M quencher solution containing 25 μM catalyst was prepared in an analogous manner using a 2-dram vial equipped with a PTFE-lined cap. This ensures the concentration of photoredox catalyst does not change upon addition of quencher solution. Aliquots were added in equal increments, collecting fluorescence decay measurements after each addition. Bimolecular quenching constants were obtained as the average of 2 independent trials where dynamic quenching was observed.

Supplementary Table 12. Quenching of **Catalyst C** using **1a** in CH_3CN/PhCl (10/1)

Trial 1:

Addition	[**1a**] (M)	τ(ns)	τ_0/τ
A	0.000	11.03	1.00
B	0.002	9.12	1.21
C	0.004	8.41	1.31
D	0.006	7.82	1.41
E	0.010	6.87	1.61
F	0.014	6.27	1.76
G	0.018	5.85	1.88

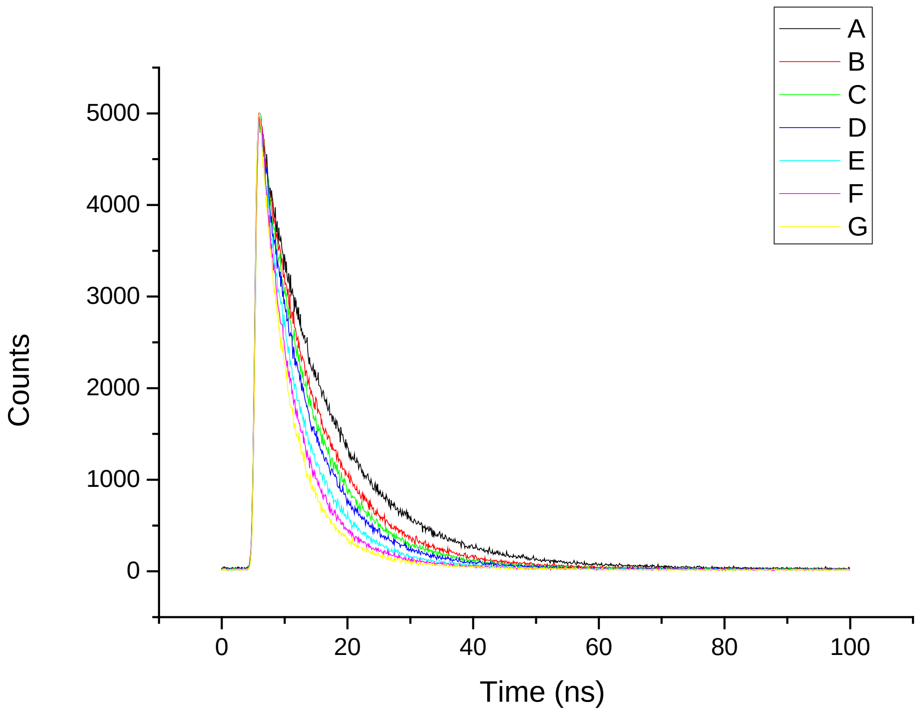

Counts
Time (ns)
A
B
C
D
E
F
G

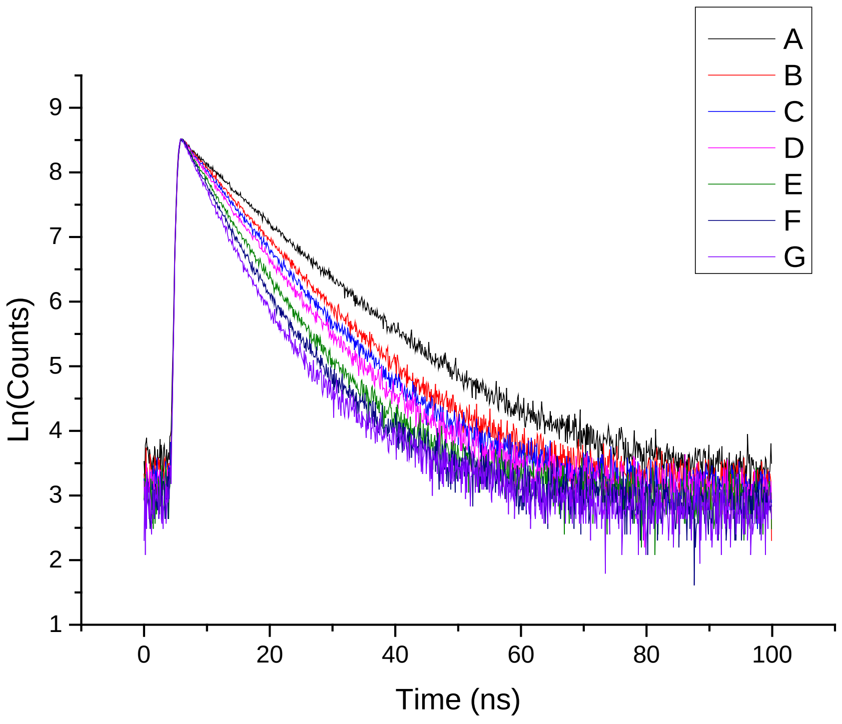

Ln(Counts)
Time (ns)
A
B
C
D
E
F
G

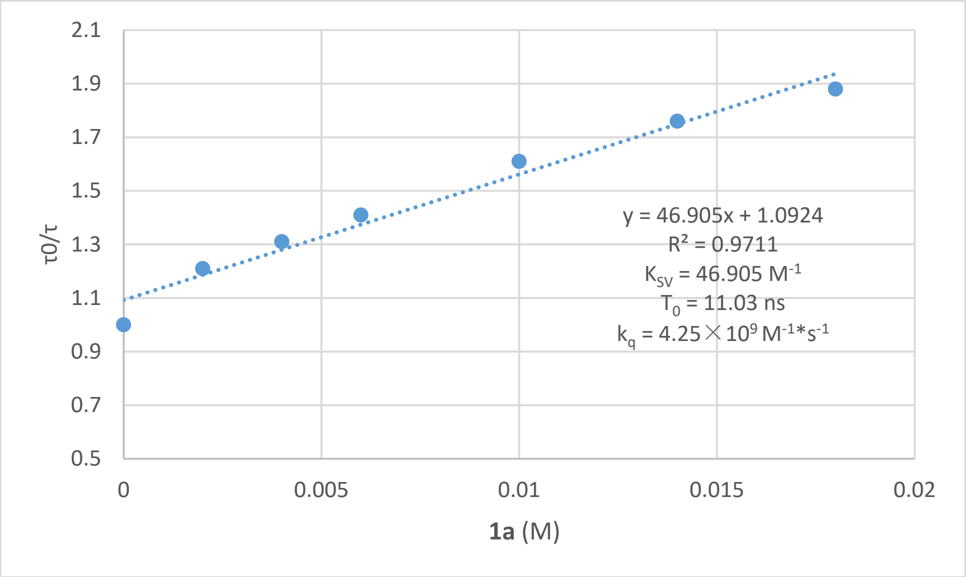

Supplementary Figure 7 Stern-Volmer plot for quenching of **Catalyst C** using **1a** in CH_3CN/PhCl (10/1) (Trial 1)

Supplementary Table 13. Quenching of **Catalyst C** using **1a** in CH_3CN/PhCl (10/1) Trial 2:

Addition	[**1a**] (M)	τ(ns)	τ_0/τ
A	0.000	11.09	1.00
B	0.002	9.12	1.22
C	0.004	8.18	1.36
D	0.006	7.69	1.44
E	0.010	6.78	1.64
F	0.014	6.14	1.81
G	0.018	5.74	1.93

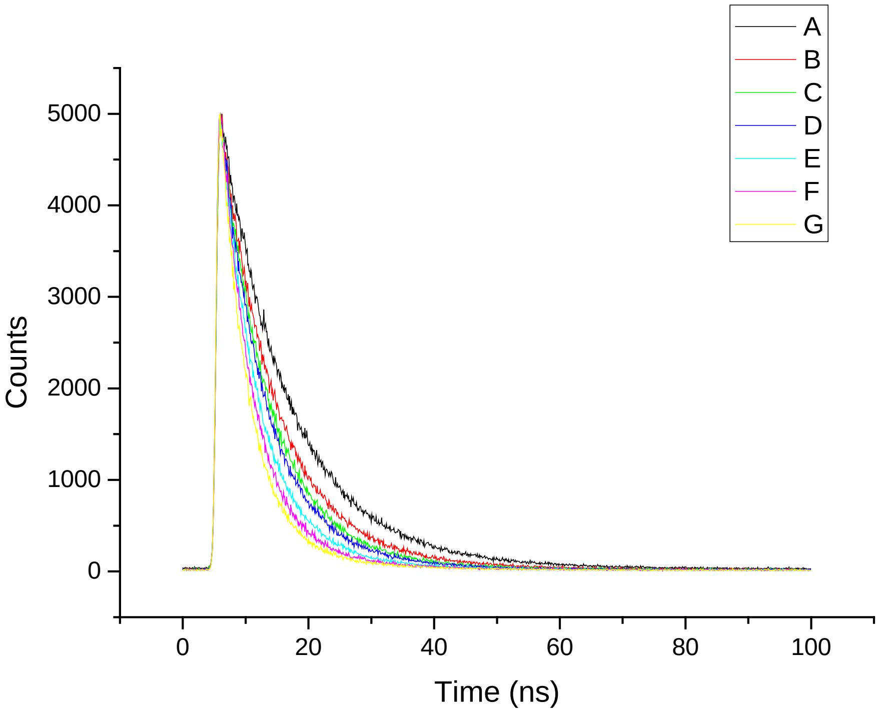

Counts
5000
4000
3000
2000
1000
0
0
20
40
60
80
100
Time (ns)
A
B
C
D
E
F
G

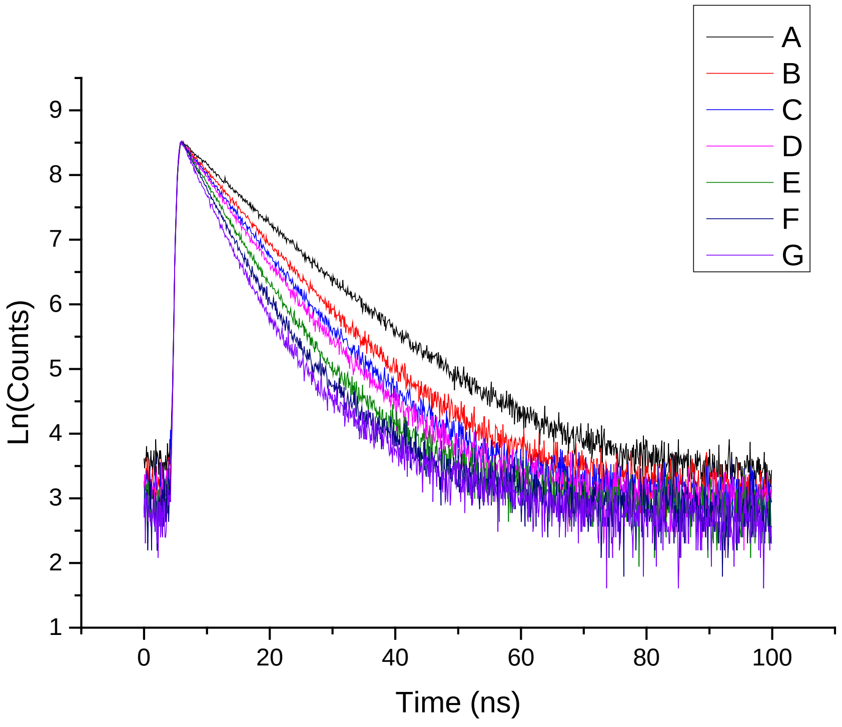

Ln(Counts)
9
8
7
6
5
4
3
2
1
0
20
40
60
80
100
Time (ns)
A
B
C
D
E
F
G

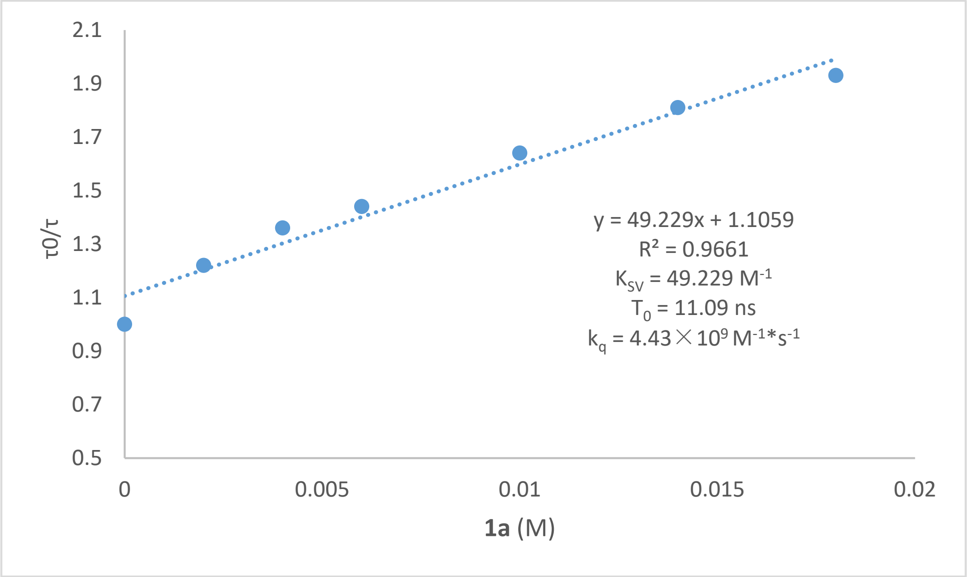

Supplementary Figure 8 Stern-Volmer plot for quenching of **Catalyst C** using **1a** in CH_3CN/PhCl (10/1) (Trial 2)

Average $k_q = 4.34\times10^{9}\ M^{-1}s^{-1}$

Supplementary Table 14. Quenching of **Catalyst C** using **2a** in CH_3CN/PhCl (10/1)

Trial 1:

Addition	[**2a**] (M)	τ(ns)	τ_0/τ
A	0.000	11.03	1.00
B	0.004	11.02	1.00
C	0.010	10.95	1.01
D	0.014	10.98	1.00
E	0.018	10.93	1.01

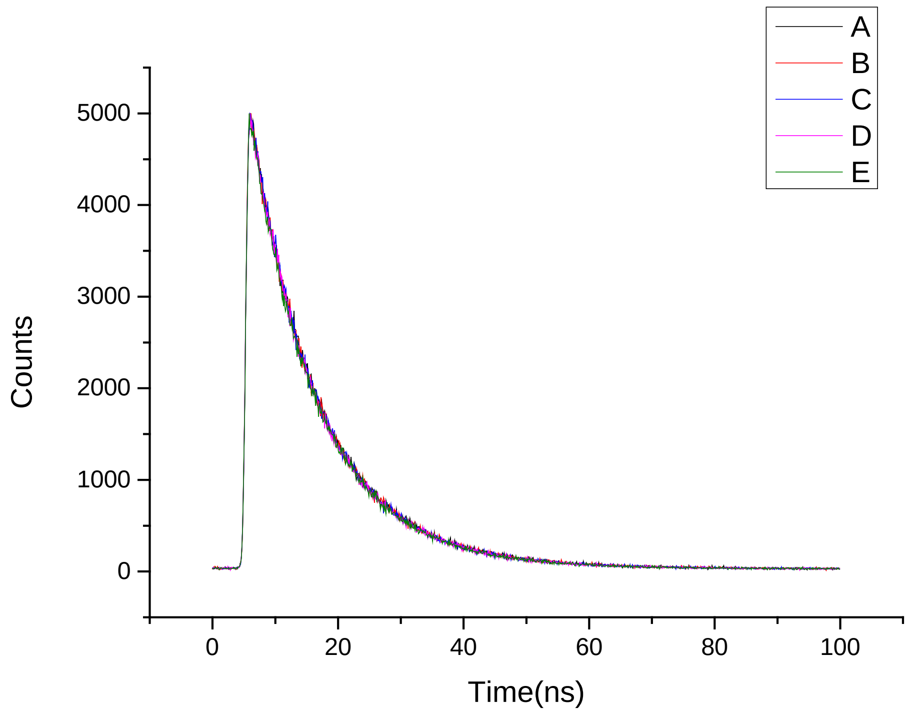

A
B
C
D
E
Counts
5000
4000
3000
2000
1000
0
0
20
40
60
80
100
Time(ns)

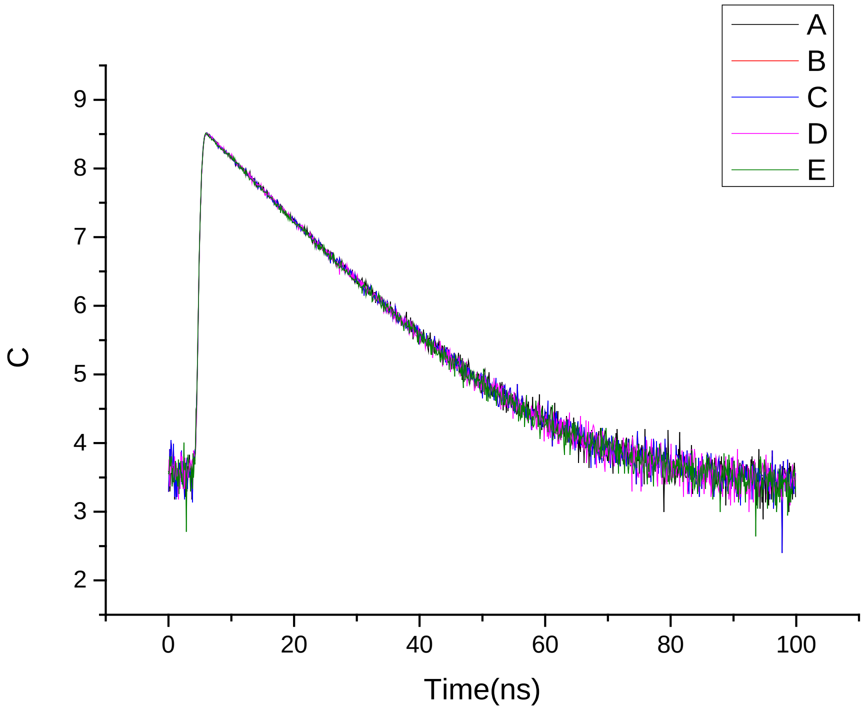

A
B
C
D
E
C
9
8
7
6
5
4
3
2
0
20
40
60
80
100
Time(ns)

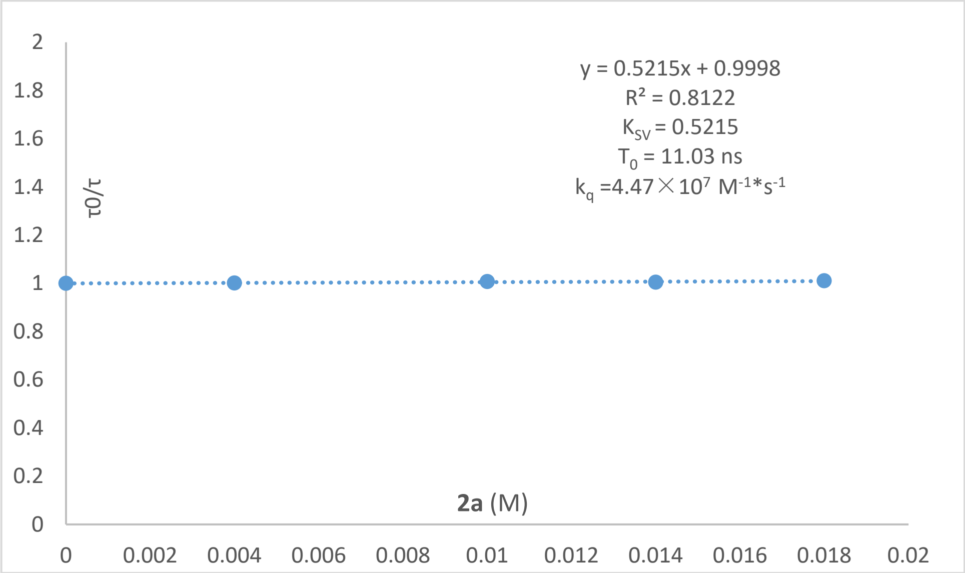

Supplementary Figure 9 Stern-Volmer plot for quenching of **Catalyst C** using **2a** in CH_3CN/PhCl (10/1) (Trial 1)

Dynamic quenching not observed using 2a in CH_3CN/PhCl (10/1).

Supplementary Table 15. Quenching of **Catalyst C** using **2a** in CH_3CN/PhCl (10/1)

Trial 2:

Addition	[**2a**](M)	τ(ns)	τ_0/τ
A	0.000	11.07	1.00
B	0.002	11.03	1.00
C	0.010	10.86	1.02
D	0.014	10.84	1.02
E	0.018	10.82	1.02

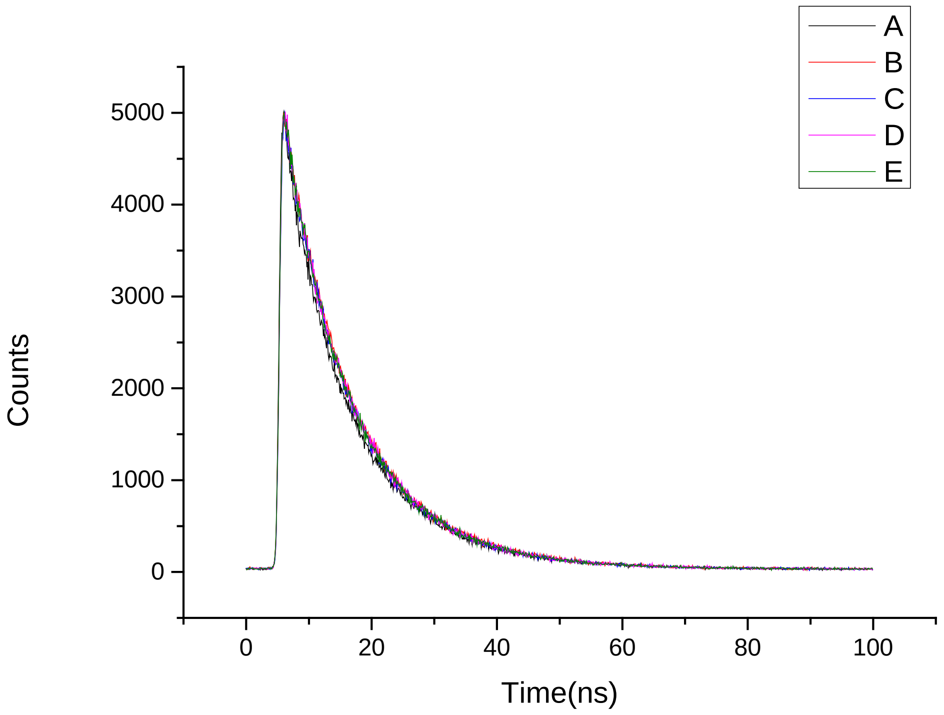

A
B
C
D
E
Counts
5000
4000
3000
2000
1000
0
0
20
40
60
80
100
Time(ns)

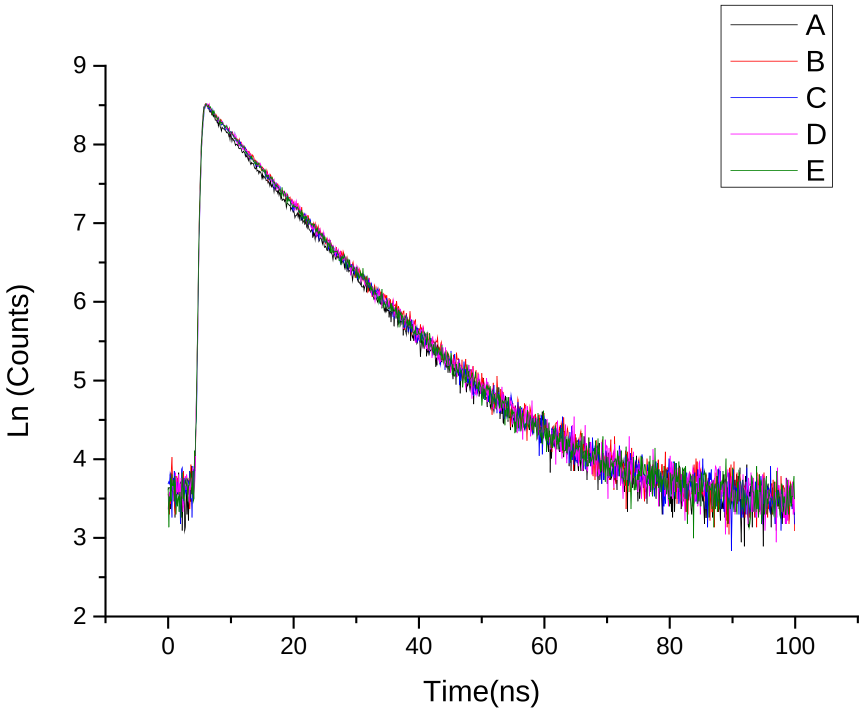

A
B
C
D
E
Ln (Counts)
9
8
7
6
5
4
3
2
0
20
40
60
80
100
Time(ns)

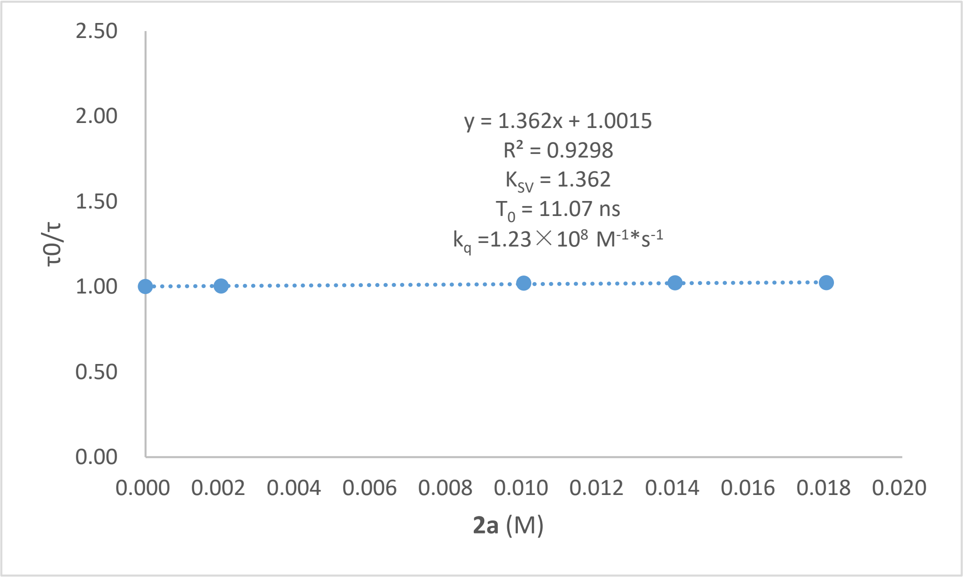

Supplementary Figure 10 Stern-Volmer plot for quenching of **Catalyst C** using **2a** in CH_3CN/PhCl (10/1) (Trial 2)

Dynamic quenching not observed using 2a in CH_3CN/PhCl (10/1).

Supplementary Table 16. Quenching of **Catalyst C** using $LiBF_4$ in CH_3CN/PhCl (10/1)

Trial 1:

Addition	[**$LiBF_4$**](M)	τ(ns)	τ_0/τ
A	0.000	11.12	1.00
B	0.002	11.08	1.00
C	0.004	11.01	1.01
D	0.006	10.88	1.02
E	0.010	10.81	1.03
F	0.014	10.79	1.03

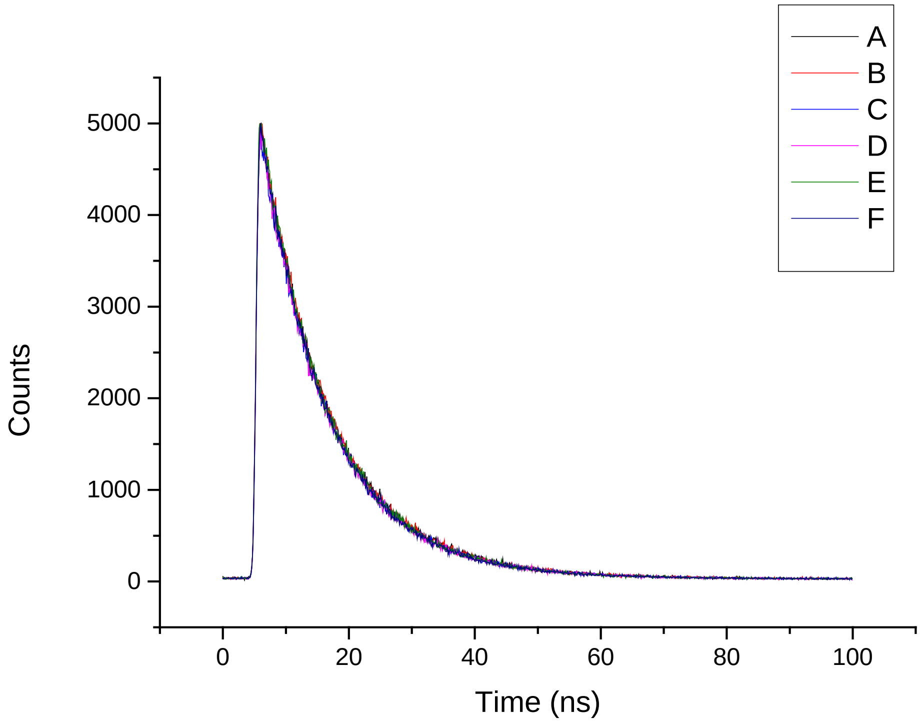

A
B
C
D
E
F
5000
4000
3000
2000
1000
0
Counts
0
20
40
60
80
100
Time (ns)

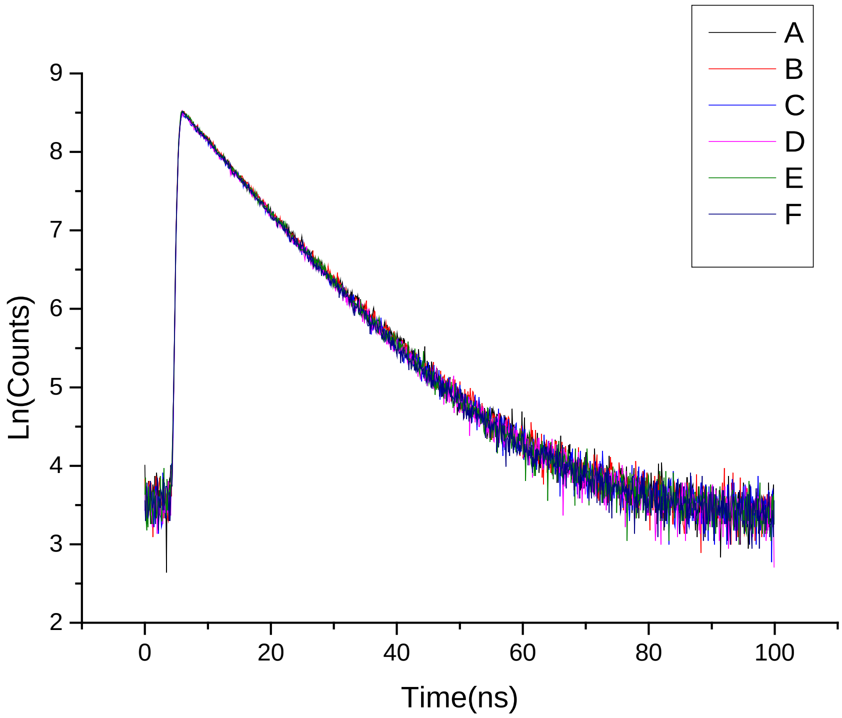

A
B
C
D
E
F
9
8
7
6
5
4
3
2
Ln(Counts)
0
20
40
60
80
100
Time(ns)

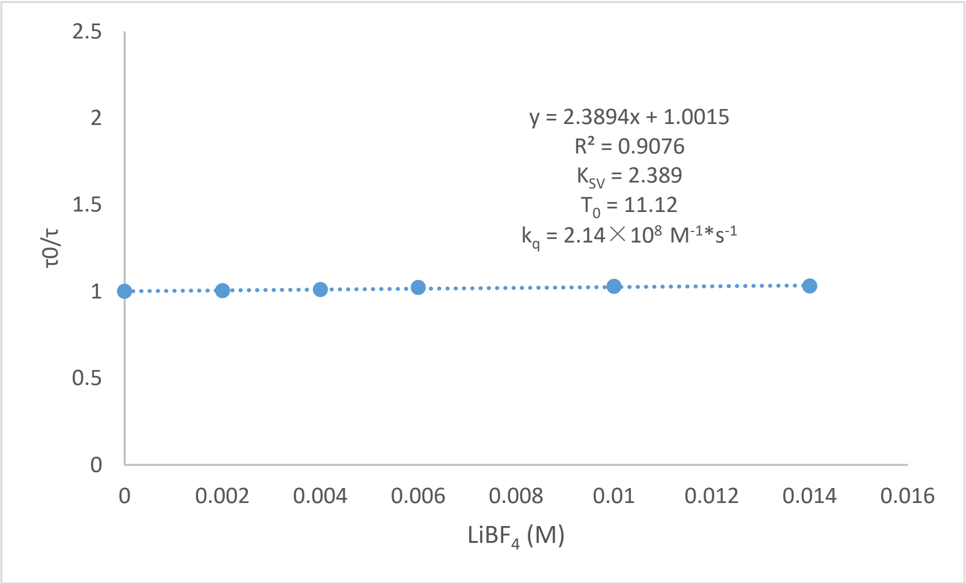

Supplementary Figure 11 Stern-Volmer plot for quenching of **Catalyst C** using $LiBF_4$ in CH_3CN/PhCl (10/1) (Trial 1)

Dynamic quenching not observed using $LiBF_4$ in CH_3CN/PhCl (10/1).

Supplementary Table 17. Quenching of **Catalyst C** using $LiBF_4$ in CH_3CN/PhCl (10/1)

Trial 2:

Addition	[$LiBF_4$](M)	τ(ns)	τ_0/τ
A	0.000	11.17	1.00
B	0.002	11.02	1.01
C	0.006	10.96	1.02
D	0.010	10.98	1.02
E	0.018	10.85	1.03

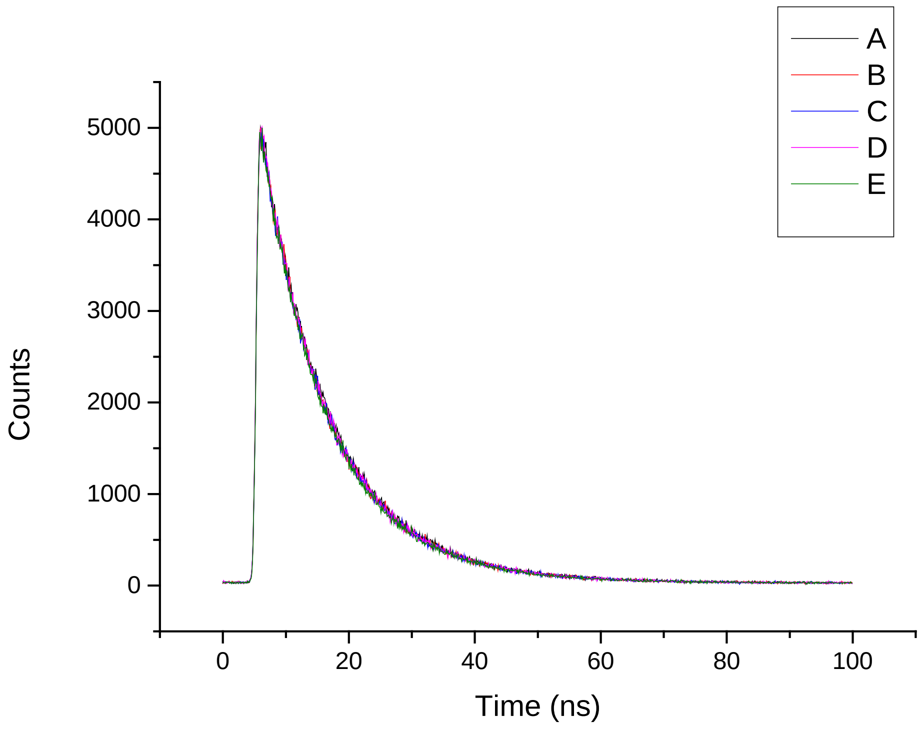
A
B
C
D
E
5000
4000
3000
2000
1000
0
Counts
0
20
40
60
80
100
Time (ns)

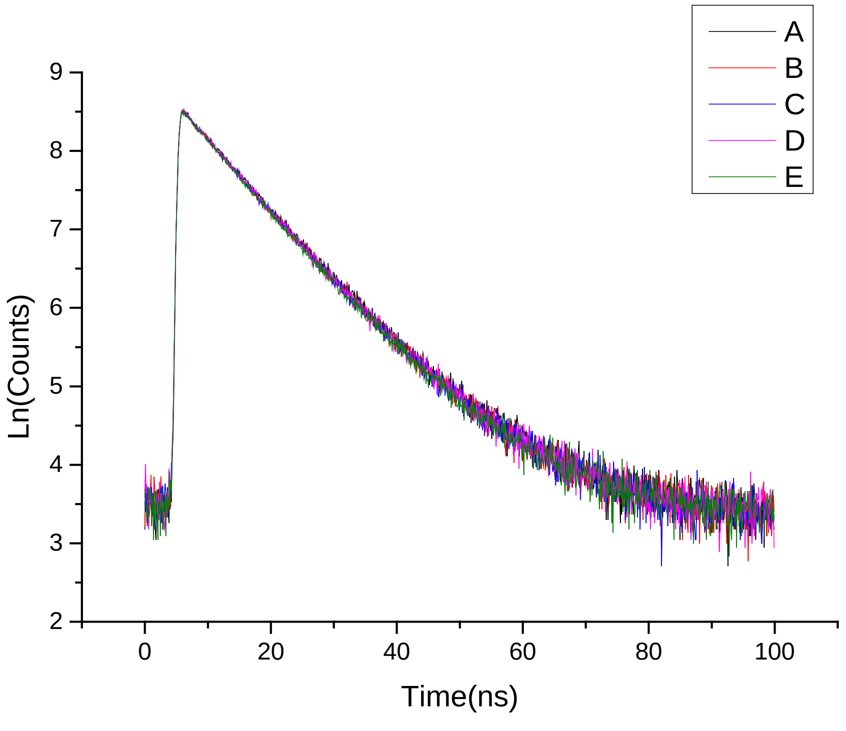
A
B
C
D
E
9
8
7
6
5
4
3
2
Ln(Counts)
0
20
40
60
80
100
Time(ns)

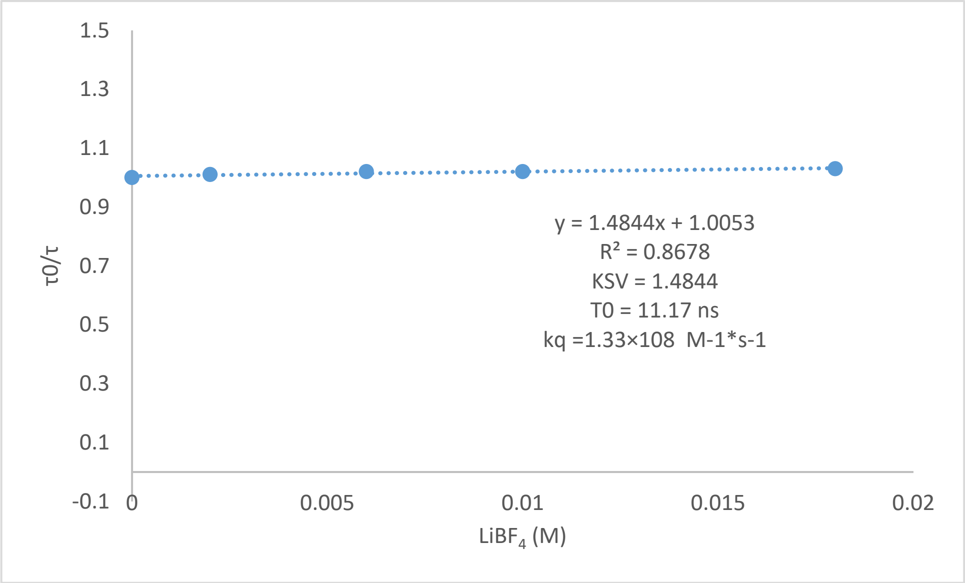

Supplementary Figure 12 Stern-Volmer plot for quenching of **Catalyst C** using $LiBF_4$ in CH_3CN/PhCl (10/1) (Trial 2)

Dynamic quenching not observed using $LiBF_4$ in CH_3CN/PhCl (10/1).

Quantum yield measurements

Blue LED (λmax = 440 nm) was used for measurement of quantum yield.

Determination of the light intensity at 440 nm

The photon flux of the blue LED light was determined by standard ferrioxalate actinometry,[16-18] the photon flux of the LED (λmax = 440 nm) was determined by standard ferrioxalate actinometry. A 0.15 M solution of ferrioxalate was prepared by dissolving potassium ferrioxalate hydrate (2.21 g) in H_2SO_4 (30 mL of a 0.05 M solution). A buffered solution of 1,10-phenanthroline was prepared by dissolving 1,10-phenanthroline (50 mg) and sodium acetate (11.25g) in H_2SO_4 (50 mL of a 0.5 M solution). Both solutions were stored in the dark. To determine the photon flux of the LED, the ferrioxalate solution (2.0 mL) was placed in a cuvette and irradiated for 45 seconds at λmax = 440 nm. After irradiation, the phenanthroline solution (0.35 mL) was added to the cuvette and the mixture was allowed to stand in the dark for 1 h to allow the ferrous ions to completely coordinate to the phenanthroline. The absorbance

of the solution was measured at 510 nm. A nonirradiated sample was also prepared and the absorbance at 510 nm was measured. Conversion was calculated using eq 5.

$$\text{Mol of } Fe^{2+} = \frac{V \cdot \Delta A_{510\,nm}}{l \cdot \varepsilon} = \frac{(0.00235\ L)\cdot(1.425)}{(1.0\ cm)\cdot 11100\frac{L}{mol}\cdot cm)} = 3.02\times10^{-7}\ \text{mol} \qquad (5)$$

V is the total volume (0.00235 L) of the solution after addition of phenanthroline, ΔA (1.425) is the difference in absorbance at 510 nm between the irradiated and non-irradiated solutions (**Supplementary Figure 13**), l is the path length (1.00 cm), and ε is the molar absorptivity of the ferrioxalate actinometer at 510 nm (11,100 $Lmol^{-1}cm^{-1}$). The photon flux can be calculated using eq 6.

$$\text{Photon flux} = \frac{mol\ of\ Fe^{2+}}{\emptyset \cdot t \cdot f} = \frac{3.02\times10^{-7}}{(1.01)\cdot(45\ s)\cdot(0.994)} = 6.68\times10^{-9}\ \text{einstein/s} \qquad (6)$$

Where Φ is the quantum yield for the ferrioxalate actinometer (1.01 at λ = 440 nm), t is the irradiation time (45 s), and f is the fraction of light absorbed at 440 nm by the ferrioxalate actinometer. This value is calculated using eq 7 where A_{440} nm is the absorbance of the ferrioxalate solution at 440 nm.

$$f = 1-10^{-A_{440\,nm}} = 1-10^{-2.256} = 0.994 \qquad (7)$$

The photon flux was thus calculated (average of three experiments) to be 6.68×10^{-9} einstein/s.

Determination of the reaction quantum yield.

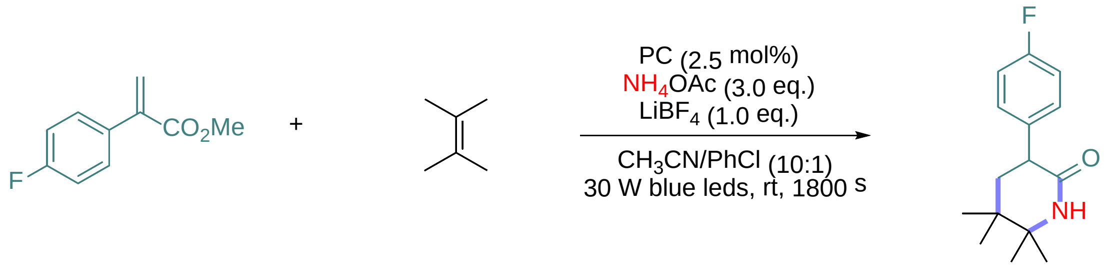

2b, 0.2 mmol **1b**, 0.1 mmol **3c**

A cuvette sealed with a rubber stopper was charged with *N*-Ph-9-mesityl 3,6-di-*tert*-butylacridinium tetrafluoroborate (1.4 mg, 2.5 μmol, 2.5 mol%), NH_4OAc (23.1 mg, 0.3 mmol), $LiBF_4$ (9.4 mg, 0.1 mmol) and CH_3CN (1.0 mL), **1b** (8.4 mg, 0.1 mmol),

2b (36.0 mg, 0.2 mmol) and PhCl (0.1 mL) were added consecutively via syringe. The sample was stirred and irradiated (λ = 440 nm) for 1800 s (0.5 h) at room temperature. After irradiation, the solvent was removed. The yield of product formed was determined as 9% yield (9×10^{-6} mol of **3c**) by crude ^{1}H NMR based on a mesitylene standard. The reaction quantum yield (Φ) was determined using eq 9 where the photon flux is 6.68×10^{-9} *einstein/s* (determined by actinometry as described above), t is the reaction time (1800 s) and f is the fraction of incident light absorbed by the photosensitizer, Mes-3,6-tBu_2-Acr-$Ph^+BF_4^-$ (**Catalyst C**), determined using eq 8. An absorption spectrum of the catalyst ($2.5*10^{-5}$ M) gave an absorbance value of 0.0979 at 440 nm (**Supplementary Figure 14**), indicating that the fraction of light absorbed by the photocatalyst (f) is 0.9979.

$$f = 1-10^{-A_{440\,nm}} = 1-10^{-0.0979} = 0.9979 \qquad (8)$$

$$\emptyset = \frac{mol\ of\ product}{flux \cdot t \cdot f} = \frac{9\times10^{-6}\ mol}{6.68\times10^{-9}\ einstein/s \times 1800s \times 0.9979} = 0.75 \qquad (9)$$

The reaction quantum yield (Φ) was calculated to be 0.75.

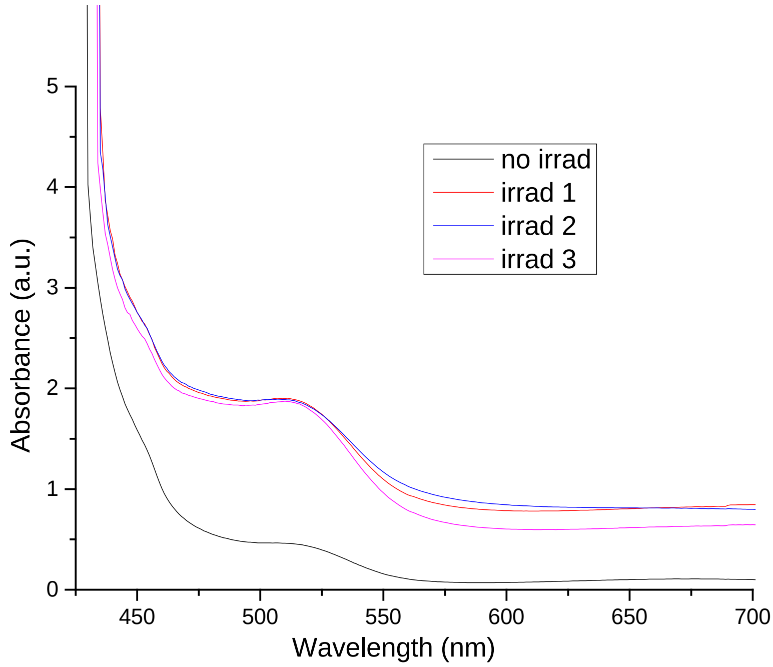

Supplementary Figure 13 Three irradiation experiments and non-irradiation experiment absorption spectra

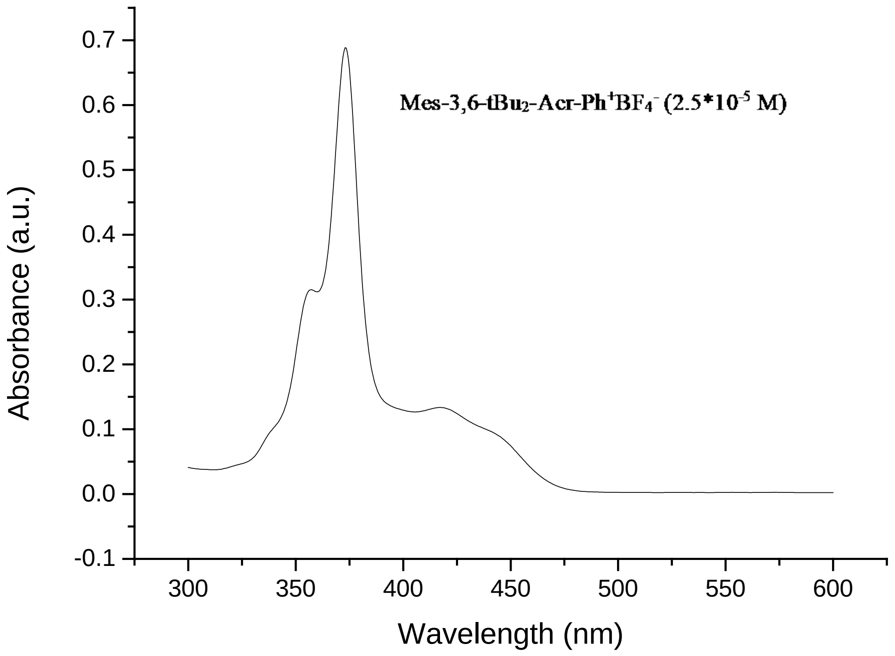

Supplementary Figure 14 Absorption spectra of **Catalyst C**

Scale-up reaction

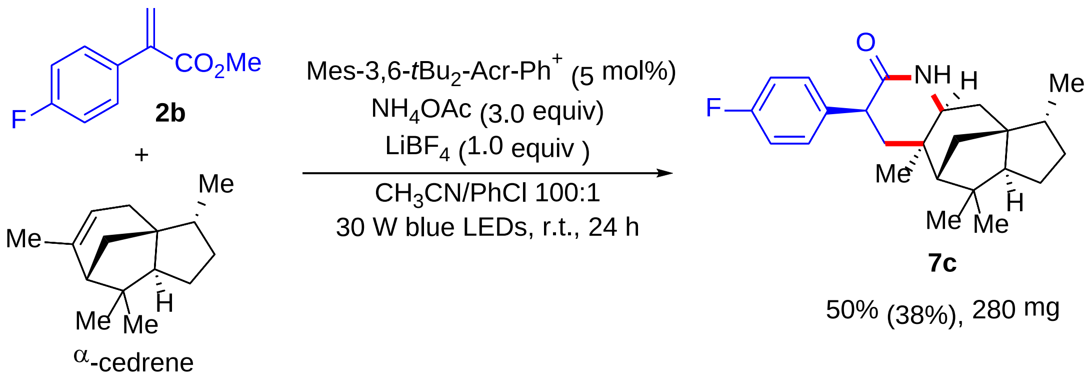

An oven-dried 500 ml Schlenk-tube equipped with a magnetic stir bar was charged with *N*-Ph-9-mesityl 3,6-di-*tert*-butylacridinium tetrafluoroborate (57.3 mg, 0.1 mmol, 5.0 mol%), NH_4OAc (462.5 mg, 6.0 mmol, 3.0 eq.) and $LiBF_4$ (187.5 mg, 2.0 mmol). CH_3CN (200 mL), *α*-cedrene (408.7 mg, 2.0 mmol), methyl 2-(4-fluorophenyl)acrylate (720.7 mg, 4.0 mmol, 2.0 eq.) and PhCl (2.0 mL) were added consecutively via syringe. The tube was sealed with a Teflon-coated septum cap, and stirred at ambient

temperature under irradiation with 30W blue LEDs for 24 h. The reaction mixture was quenched with water and extracted with Ethyl acetate. The combined organic phase was concentrated in vacumn. The residue was analyzed by ^{1}H NMR with PhTMS (75.1 mg, 0.5 mmol) as internal standard to determine the yield as 50%, **7c** was isolated (280 mg) as pale yellow solid in 38% yield.

X-ray diffraction data

The suitable crystal of **3a**, **4j**, **4o**, **5j**, **5o**, **6a**, **6b**, **6e** and **7c** were selected and mounted on an Bruker D8 venture microsource diffractometer. Data were measured using monochromatic Cu-Kαradiation at T = 100 K. Data reduction, scaling and absorption corrections were performed using SAINT (Bruker, V8.38A, 2013).

The structure was solved with the ShelXT (Sheldrick, 2015) structure solution program using the intrinsic Phasing solution method and by using Olex 2 (Dolomanov et al., 2009) as the graphical interface. The model was refined with version 2016/6 of ShelXL (Sheldrick, 2008) using Least Squares minimisation.

A suitable crystal of **3y** was selected and mounted on an Bruker D8 venture microsource diffractometer. Data were measured using monochromatic Mo-Kαradiation at T = 100 K. Data reduction, scaling and absorption corrections were performed using SAINT (Bruker, V8.38A, 2013).

The structure was solved with the ShelXT (Sheldrick, 2015) structure solution program using the intrinsic Phasing solution method and by using Olex 2 (Dolomanov et al., 2009) as the graphical interface. The model was refined with version 2016/6 of ShelXL (Sheldrick, 2008) using Least Squares minimisation.

The crystal structure of **3y** is slightly disordered due to atom vibration, the configuration determination of **3y** is not affected by the disorder. For crystal structures of **4j**, **5j** and **5o**, the molecules in the asymmetric unit is disordered due to the presence of isomer in the sample. Two disordered parts have been solved by the "disorder tools" plugin in

Olex2. All 1,2-distances and 1,3-distances involved in the disorder have been restricted by SADI command. In addition, EADP and/or ISOR commands have been used to make the anisotropic displacement parameters of disordered atoms reasonable. For more details, please see the CIF files.

Supplementary Table 18. Crystal data and structure refinement for **3a** (CCDC 2194532), **3y** (CCDC 2194539), **4j** (CCDC 2194535), **4o** (CCDC 2207224) and **5j** (CCDC 2194544)

	**3a**	**3y**	**4j**	**4o**	**5j**
Empirical formula	$C_{27}H_{29}FN_2O_2$	$C_{11}H_{21}NO_2$	$C_{45}H_{58}N_2O_{6.5}$	$C_{24}H_{23}NO_2$	$C_{21}H_{25}FNO_{1.5}$
Formula weight	432.52	199.29	730.93	357.43	334.42
Temperature/K	100.0 (2)	100.0 (2)	100.0 (2)	100.0 (2)	100.0 (2)
Crystal system	monoclinic	triclinic	triclinic	monoclinic	monoclinic
Space group	$P2_1/c$	$P\bar{1}$	$P\bar{1}$	$C2/c$	$P2_1/n$
a/Å	11.3775(7)	6.8268(8)	11.4194(6)	57.1343(16)	16.1441(4)
b/Å	8.8660(5)	7.3446(9)	12.6683(7)	5.10260(10)	6.0392(2)
c/Å	23.5378(14)	11.7611(14)	15.1413(8)	27.7917(7)	36.9913(10)
α/°	90	74.572(5)	73.690(3)	90	90
β/°	102.140(3)	88.101(4)	89.310(3)	113.794(2)	94.926(2)
γ/°	90	84.897(4)	81.879(3)	90	90
Volume/Å^3	2321.2(2)	566.17(12)	2080.3(2)	7413.5(3)	3593.24(18)
Z	4	2	2	16	8
ρ_{calc}g/cm^3	1.238	1.169	1.167	1.281	1.236
μ/mm^{-1}	0.672	0.079	0.616	0.637	0.675
F(000)	920.0	220.0	788.0	3040.0	1432.0
Crystal size/mm^3	0.31 × 0.24 × 0.18	0.32 × 0.31 × 0.24	0.35 × 0.24 × 0.22	0.02 × 0.02 × 0.01	0.36 × 0.24 × 0.22

Radiation	CuKα (λ = 1.54178)	MoKα (λ = 0.71073)	CuKα (λ = 1.54178)	CuKα (λ = 1.54184)	CuKα (λ = 1.54178)
2Θ range for data collection/°	7.684 to 136.664	5.774 to 55.216	6.084 to 136.68	6.386 to 130.326	5.804 to 137.23
Index ranges	-13 ≤ h ≤ 13<br>-10 ≤ k ≤ 10<br>-25 ≤ l ≤ 28	-8 ≤ h ≤ 8<br>-9 ≤ k ≤ 9<br>-15 ≤ l ≤ 15	-11 ≤ h ≤ 13, -15 ≤ k ≤ 15, -18 ≤ l ≤ 18	-66 ≤ h ≤ 66, -6 ≤ k ≤ 5, -32 ≤ l ≤ 32	-19 ≤ h ≤ 19, -7 ≤ k ≤ 7, -44 ≤ l ≤ 41
Reflections collected	21631	2622	33744	49452	34774
Independent reflections	4253 [R_{int}= 0.0416, R_{sigma}= 0.0282]	2622 [R_{int}= 0.0925, R_{sigma}= 0.0606]	7593 [R_{int}= 0.0609, R_{sigma}= 0.0460]	6278 [R_{int} = 0.0843, R_{sigma} = 0.0460]	6619 [R_{int} = 0.0589, R_{sigma} = 0.0410]
Data/restraints/parameters	4253/0/291	2622/13/157	7593/138/625	6278/0/514	6619/80/506
Goodness-of-fit on F^2	1.057	1.091	1.046	1.065	1.216
Final R indexes [I>=2σ (I)]	R_1= 0.0516<br>wR_2= 0.1259	R_1= 0.0773, wR_2= 0.1809	R_1= 0.0617, wR_2= 0.1776	R_1 = 0.0453, wR_2 = 0.0983	R_1= 0.0759, wR_2= 0.1583
Final R indexes [all data]	R_1= 0.0562<br>wR_2= 0.1288	R_1= 0.1124, wR_2= 0.1996	R_1= 0.0688, wR_2= 0.1849	R_1 = 0.0631, wR_2 = 0.1063	R_1= 0.0896, wR_2= 0.1641
Largest diff. peak/hole / e $Å^{-3}$	0.58/-0.25	0.37/-0.46	0.94/-0.24	0.20/-0.21	0.19/-0.23

Supplementary Table 19. Crystal data and structure refinement for **5o** (CCDC 2194546), **6a** (CCDC 2201531), **6b** (CCDC 2194547), **6e** (CCDC 2194548) and **7c** (CCDC 2194553)

	**5o**	**6a**	**6b**	**6e**	**7c**
Empirical formula	$C_{18}H_{18}FNO$	$C_{12}H_{23}NO_2Si$	$C_{14}H_{17}NO_3$	$C_{20}H_{30}FNO$	$C_{24.03}H_{32.06}Cl_{0.06}FNO$

Formula weight	283.33	241.40	247.28	319.45	372.02
Temperature/K	100.0 (2)	200.0 (2)	100.0 (2)	100.0 (2)	100.0 (2)
Crystal system	monoclinic	triclinic	triclinic	triclinic	monoclinic
Space group	$P2_1/n$	$P\bar{1}$	$P\bar{1}$	$P\bar{1}$	C2
a/Å	5.9189(6)	6.3053(2)	6.0626(5)	9.1221(8)	26.4486(9)
b/Å	11.3802(9)	8.8033(3)	9.7194(8)	9.3424(12)	9.4288(4)
c/Å	21.6088(16)	12.5995(4)	11.2117(9)	12.0659(12)	35.3706(13)
α/°	90	79.0620(10)	69.071(3)	76.677(5)	90
β/°	90.747(4)	79.7960(10)	75.172(4)	81.283(4)	103.613(2)
γ/°	90	83.4760(10)	80.221(4)	66.552(5)	90
Volume/Å^3	1455.4(2)	673.48(4)	594.26(9)	915.91(17)	8572.9(6)
Z	4	2	2	2	16
ρ_{calc}g/cm^3	1.293	1.190	1.382	1.158	1.153
μ/mm^{-1}	0.716	1.439	0.792	0.610	0.658
F(000)	600.0	264.0	264.0	348.0	3220.0
Crystal size/mm^3	0.31 × 0.25 × 0.19	0.3 × 0.22 × 0.2	0.29 × 0.15 × 0.15	0.32 × 0.28 × 0.04	0.31 × 0.28 × 0.06
Radiation	CuKα (λ = 1.54178)	CuKα (λ = 1.54178)	CuKα (λ = 1.54178)	CuKα (λ = 1.54178)	CuKα (λ = 1.54178)
2Θ range for data collection/°	8.184 to 136.51	7.238 to 144.066	8.64 to 136.572	7.546 to 136.952	5.142 to 136.902
Index ranges	-7 ≤ h ≤ 6, -10 ≤ k ≤ 13, -24 ≤ l ≤ 26	-7 ≤ h ≤ 7, -10 ≤ k ≤ 10, -15 ≤ l ≤ 15	-7 ≤ h ≤ 7, -11 ≤ k ≤ 11, -13 ≤ l ≤ 13	-10 ≤ h ≤ 10, -10 ≤ k ≤ 11, -14 ≤ l ≤ 14	-31 ≤ h ≤ 31, -10 ≤ k ≤ 11, -42 ≤ l ≤ 42
Reflections collected	12073	13495	7784	12352	91721

Independent reflections	2654 [R_{int} = 0.0276, R_{sigma} = 0.0212]	2620 [R_{int} = 0.0359, R_{sigma} = 0.0263]	2172 [R_{int}= 0.0306, R_{sigma}= 0.0289]	3337 [R_{int} = 0.0267, R_{sigma} = 0.0217]	15594 [R_{int} = 0.0622, R_{sigma} = 0.0386]
Data/restraints/parameters	2654/27/219	2620/0/148	2172/0/165	3337/1/215	15594/35/1075
Goodness-of-fit on F^2	1.113	1.062	1.049	1.029	1.073
Final R indexes [I>=2σ (I)]	R_1 = 0.0531, wR_2 = 0.1294	R_1 = 0.0346, wR_2 = 0.0926	R_1= 0.0338, wR_2= 0.0832	R_1 = 0.0353, wR_2 = 0.0893	R_1 = 0.0764, wR_2 = 0.2096
Final R indexes [all data]	R_1 = 0.0561, wR_2 = 0.1312	R_1 = 0.0359, wR_2 = 0.0936	R_1= 0.0356, wR_2= 0.0843	R_1 = 0.0381, wR_2 = 0.0911	R_1 = 0.0791, wR_2 = 0.2115
Largest diff. peak/hole / e Å^{-3}	0.36/-0.28	0.31/-0.27	0.26/-0.17	0.31/-0.20	0.51/-0.38

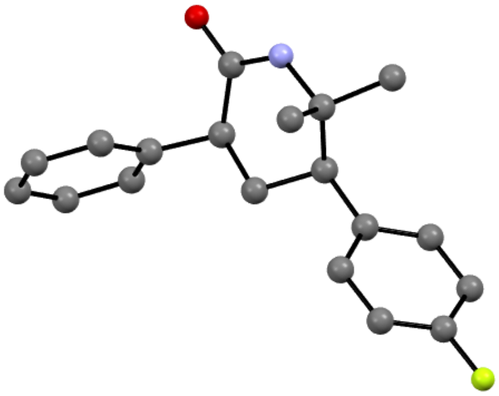

Supplementary Figure 15 Crystal structure of compound **3a** (CCDC 2194532) with thermal ellipsoids drawn at the 50% probability level. The 2-phenylacetamide molecule and hydrogen atoms have been omitted for clarity.

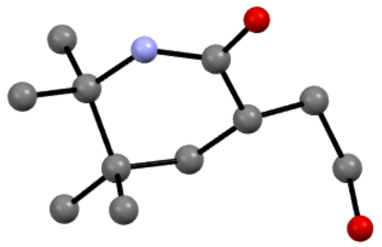

Supplementary Figure 16 Crystal structure of compound **3y** (CCDC 2194539) with thermal ellipsoids drawn at the 50% probability level. The disordered part and hydrogen atoms have been omitted for clarity.

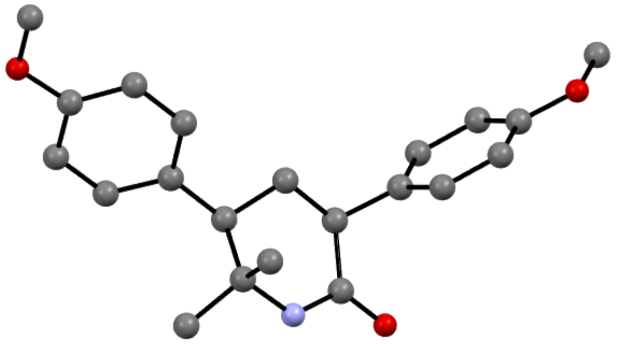

Supplementary Figure 17 Crystal structure of compound **4j** (CCDC 2194535) with thermal ellipsoids drawn at the 50% probability level. The disordered part, water molecules and hydrogen atoms have been omitted for clarity.

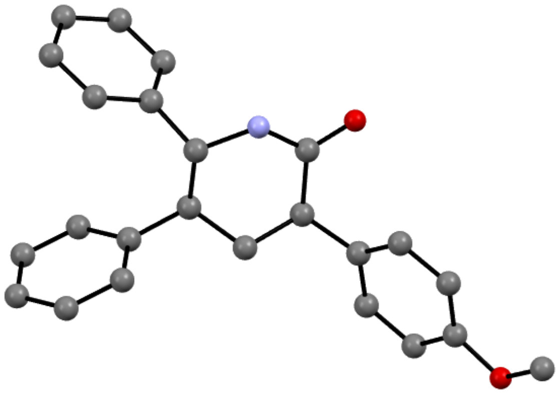

Supplementary Figure 18 Crystal structure of compound **4o** (CCDC 2207224) with thermal ellipsoids drawn at the 50% probability level. The disordered part and hydrogen atoms have been omitted for clarity.

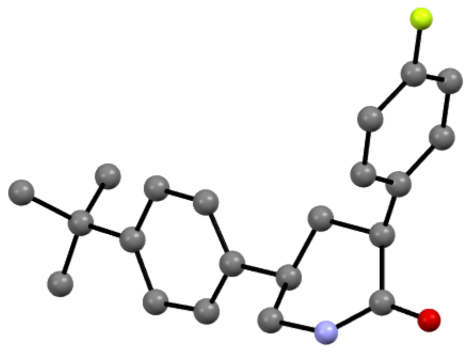

Supplementary Figure 19 Crystal structure of compound **5j** (CCDC 2194544) with thermal ellipsoids drawn at the 50% probability level. The disordered part, water molecules and hydrogen atoms have been omitted for clarity.

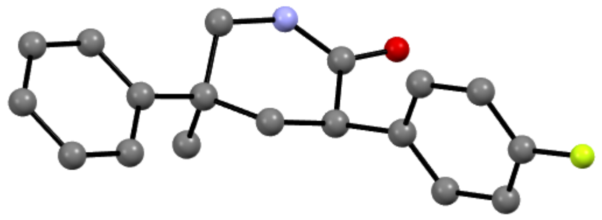

Supplementary Figure 20 Crystal structure of compound **5o** (CCDC 2194546) with thermal ellipsoids drawn at the 50% probability level. The disordered part and hydrogen atoms have been omitted for clarity.

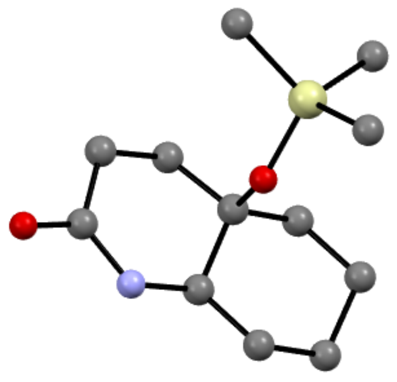

Supplementary Figure 21 Crystal structure of compound **6a** (CCDC 2201531) with thermal ellipsoids drawn at the 50% probability level. The hydrogen atoms have been omitted for clarity.

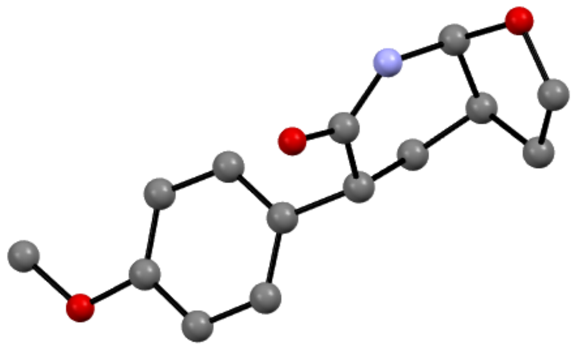

Supplementary Figure 22 Crystal structure of compound **6b** (CCDC 2194547) with thermal ellipsoids drawn at the 50% probability level. The hydrogen atoms have been omitted for clarity.

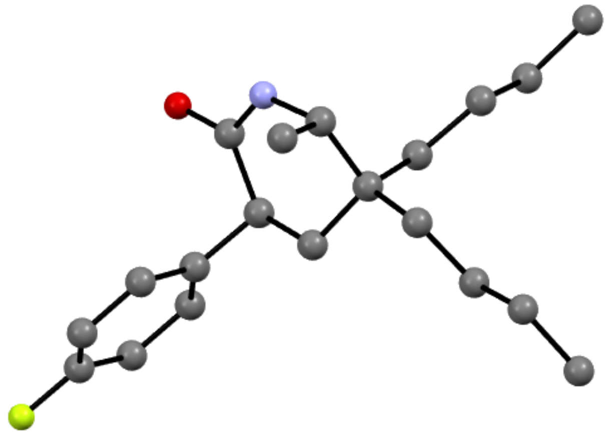

Supplementary Figure 23 Crystal structure of compound **6e** (CCDC 2194548) with thermal ellipsoids drawn at the 50% probability level. The hydrogen atoms have been omitted for clarity.

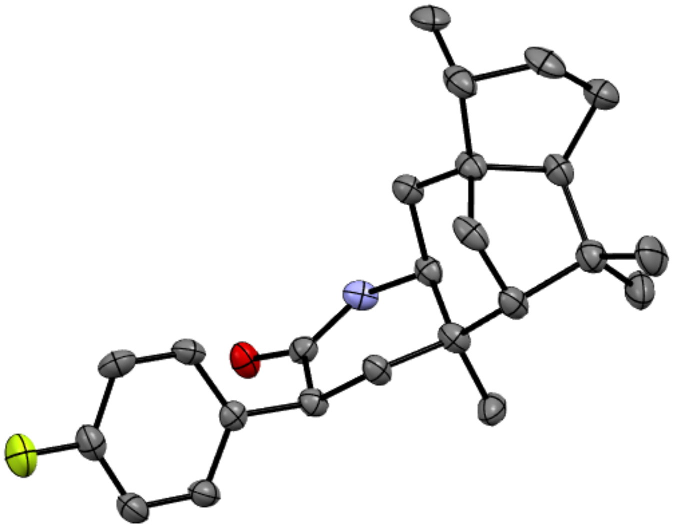

Supplementary Figure 24 Crystal structure of compound **7c** (CCDC 2194553) with thermal ellipsoids drawn at the 50% probability level. The disordered part, dichloromethane molecule and hydrogen atoms have been omitted for clarity.

Supplementary Note 3: ^{1}H, ^{13}C, and ^{19}F spectra

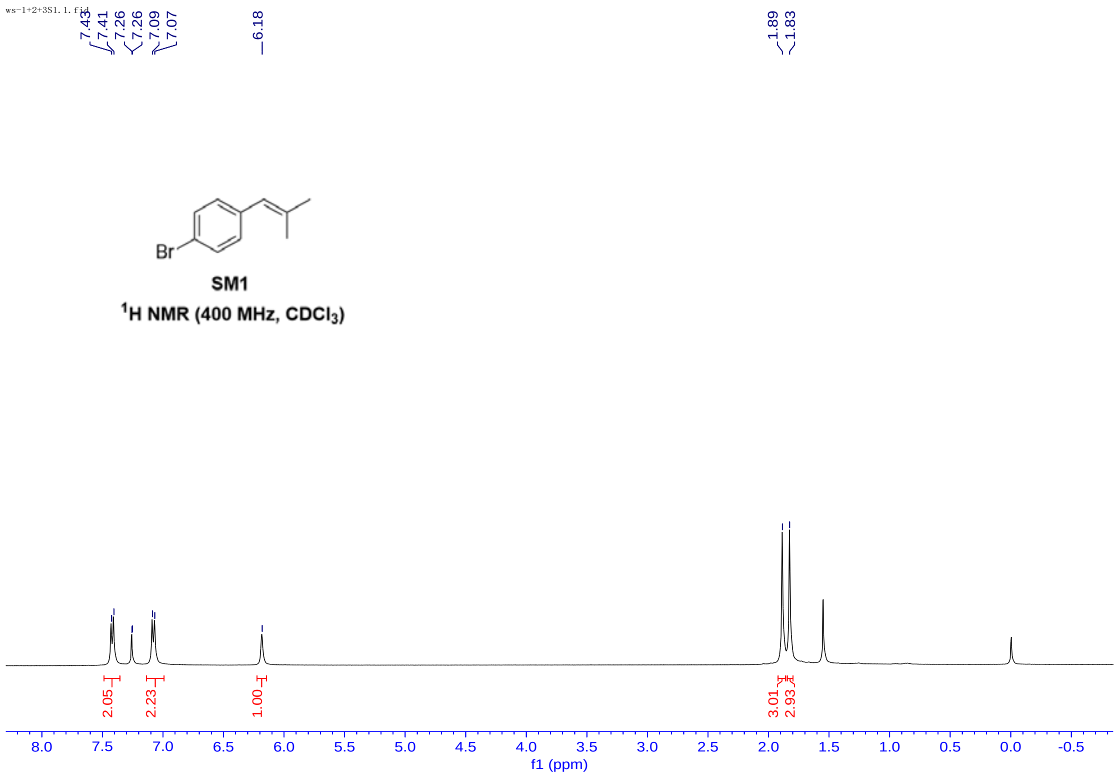

Supplementary Figure 25 ^{1}H NMR (400 MHz, 298K, $CDCl_3$) of **SM1**

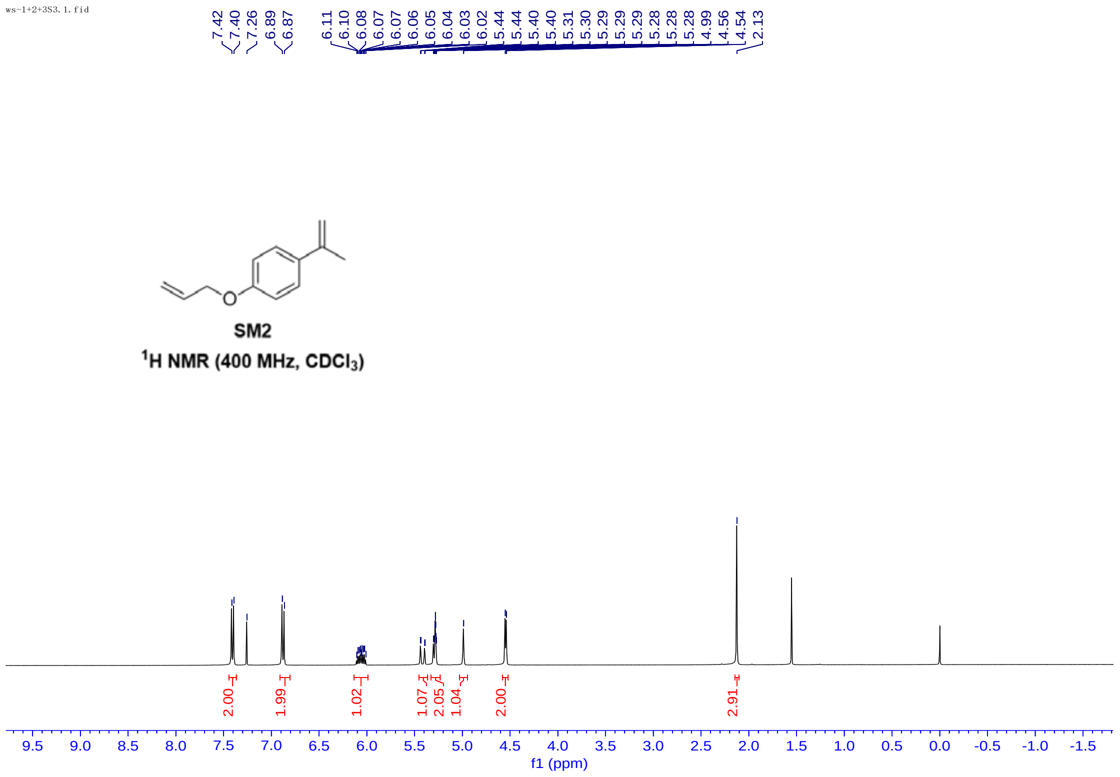

Supplementary Figure 26 ^{1}H NMR (400 MHz, 298K, $CDCl_3$) of **SM2**

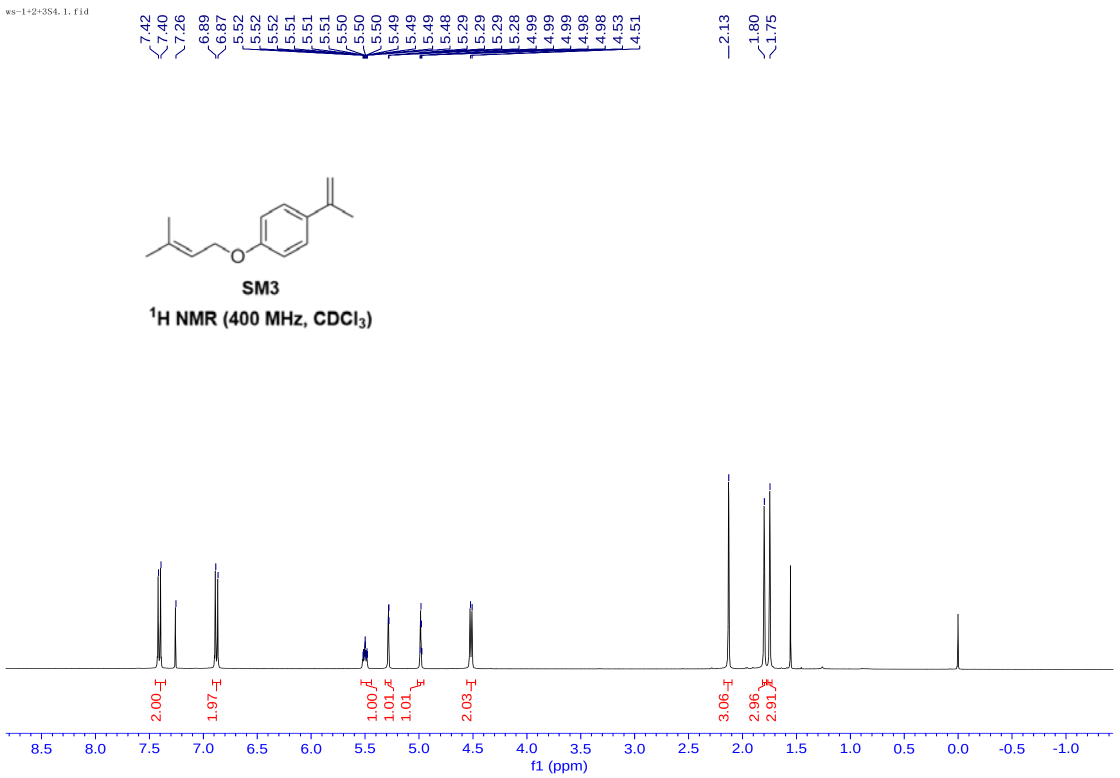

Supplementary Figure 27 ^{1}H NMR (400 MHz, 298K, $CDCl_3$) of **SM3**

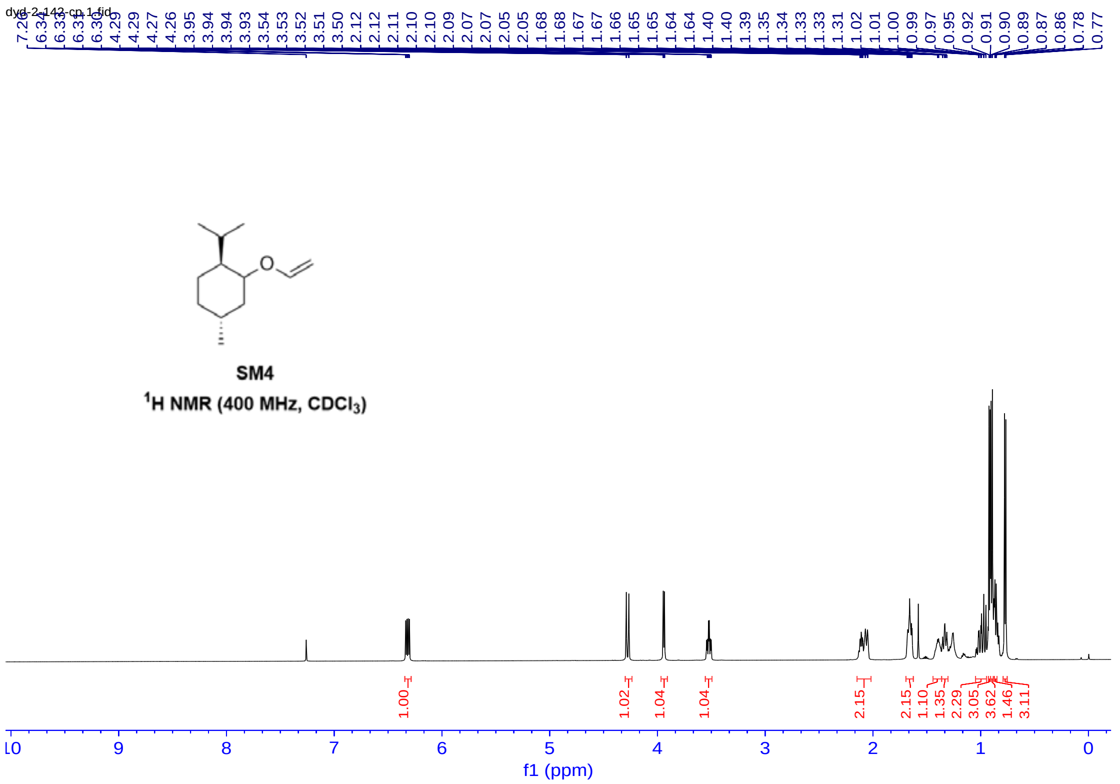

Supplementary Figure 28 ^{1}H NMR (400 MHz, 298K, $CDCl_3$) of **SM4**

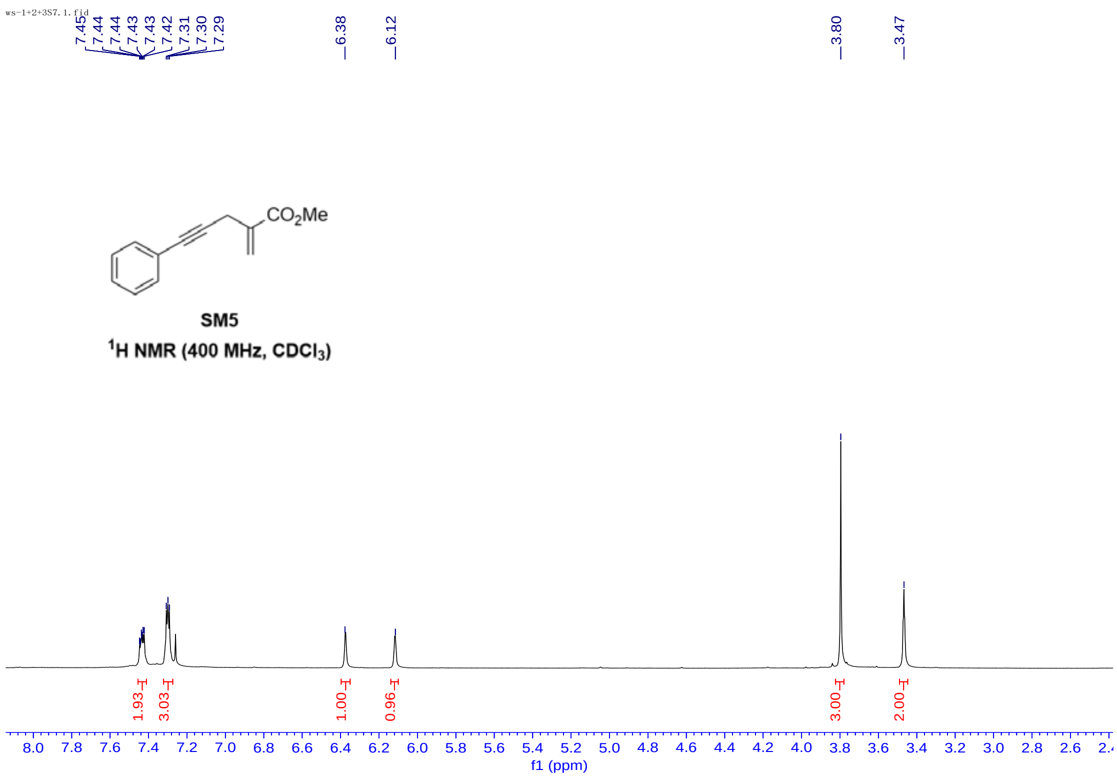

Supplementary Figure 29 ^{1}H NMR (400 MHz, 298K, $CDCl_3$) of **SM5**

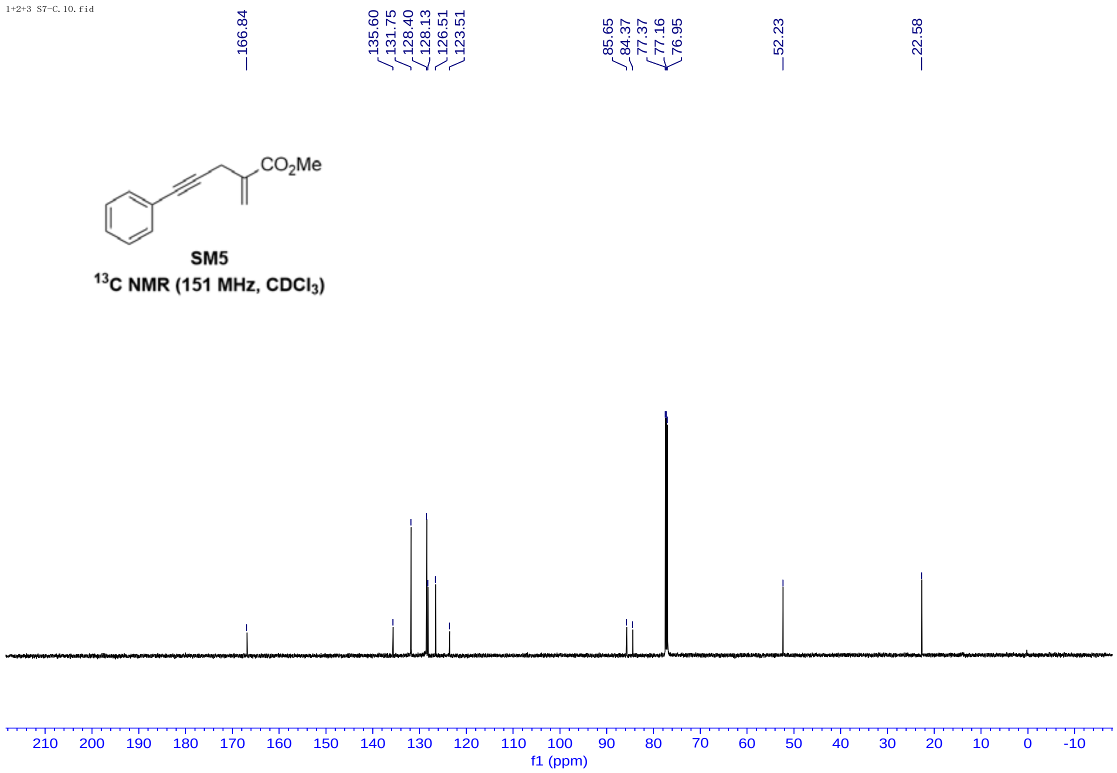

Supplementary Figure 30 ^{13}C NMR (151 MHz, 298K, $CDCl_3$) of **SM5**

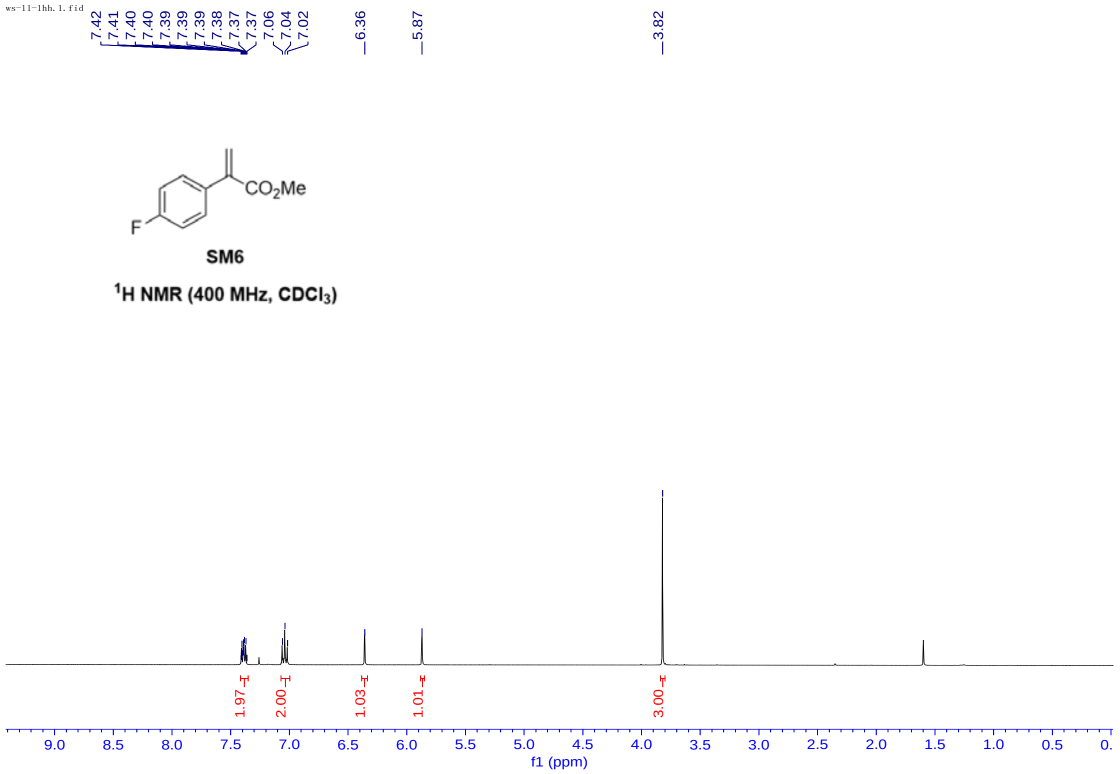

Supplementary Figure 31 ^{1}H NMR (400 MHz, 298K, $CDCl_3$) of **SM6**

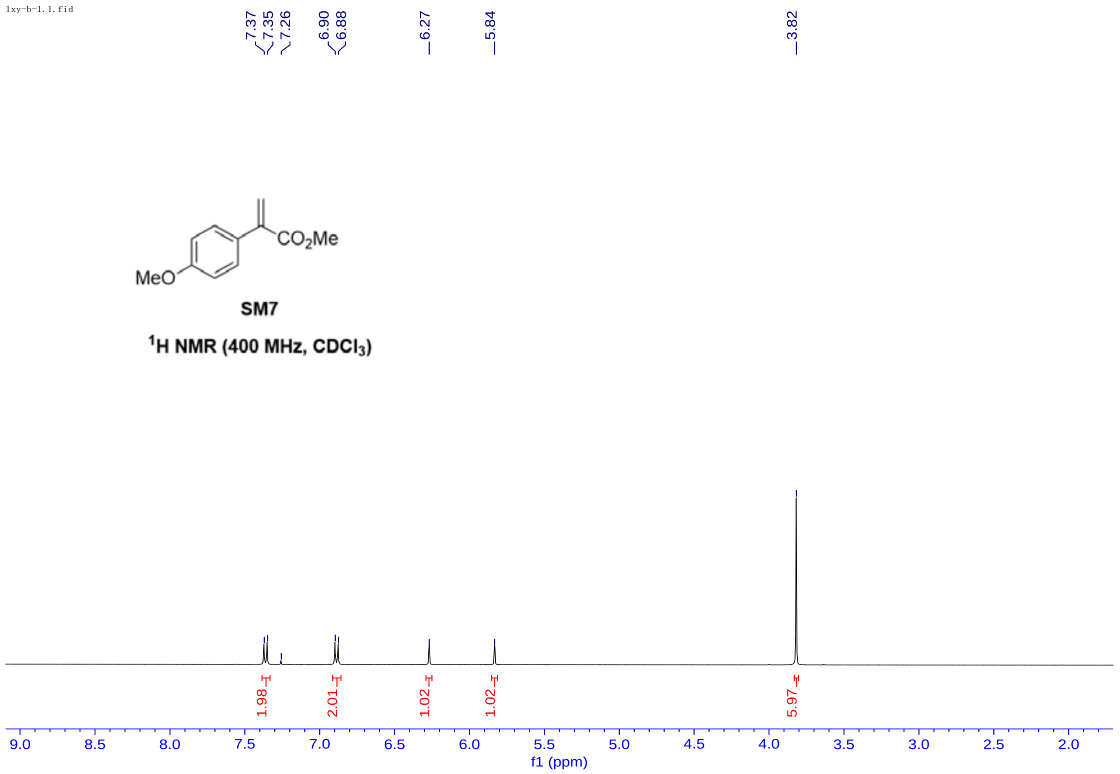

Supplementary Figure 32 ^{1}H NMR (400 MHz, 298K, $CDCl_3$) of **SM7**

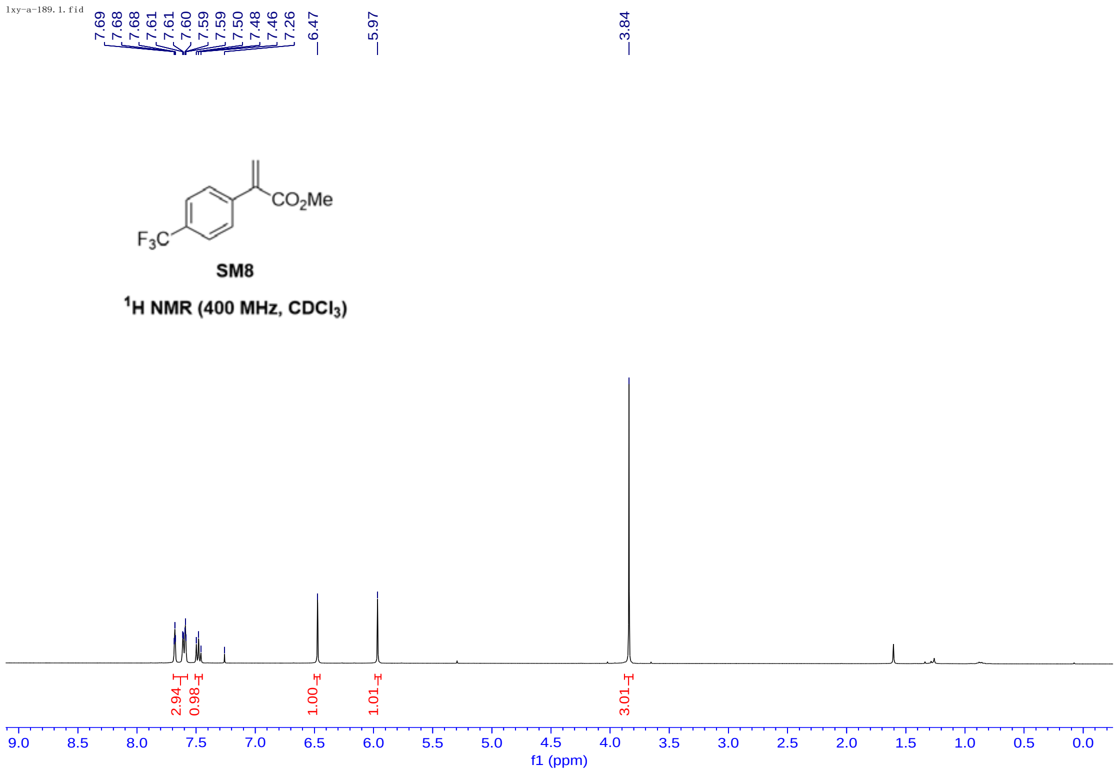

Supplementary Figure 33 ^{1}H NMR (400 MHz, 298K, $CDCl_3$) of **SM8**

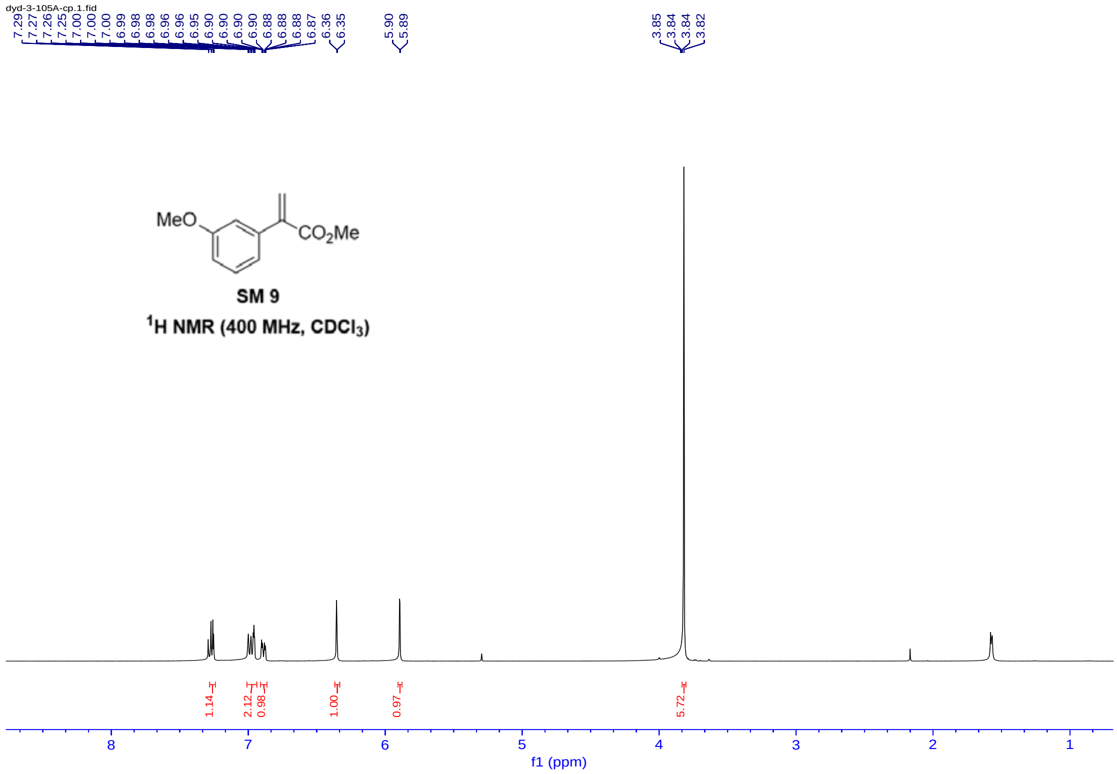

Supplementary Figure 34 ^{1}H NMR (400 MHz, 298K, $CDCl_3$) of **SM9**

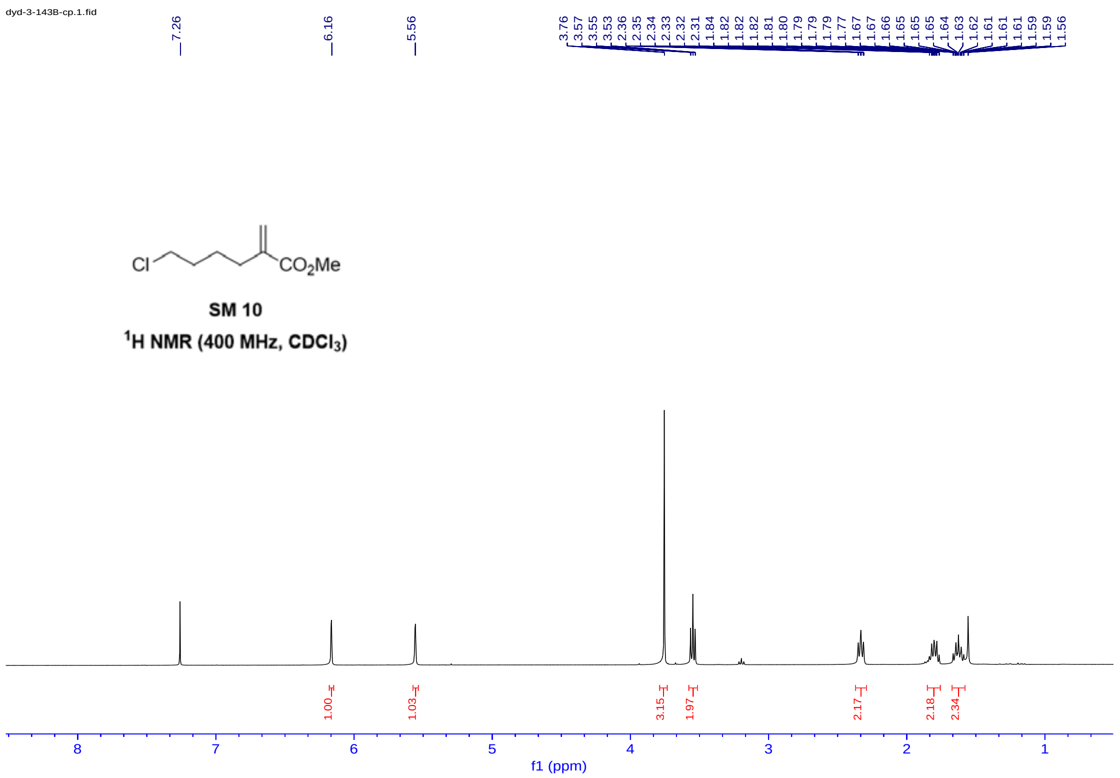

Supplementary Figure 35 ^{1}H NMR (400 MHz, 298K, $CDCl_3$) of **SM10**

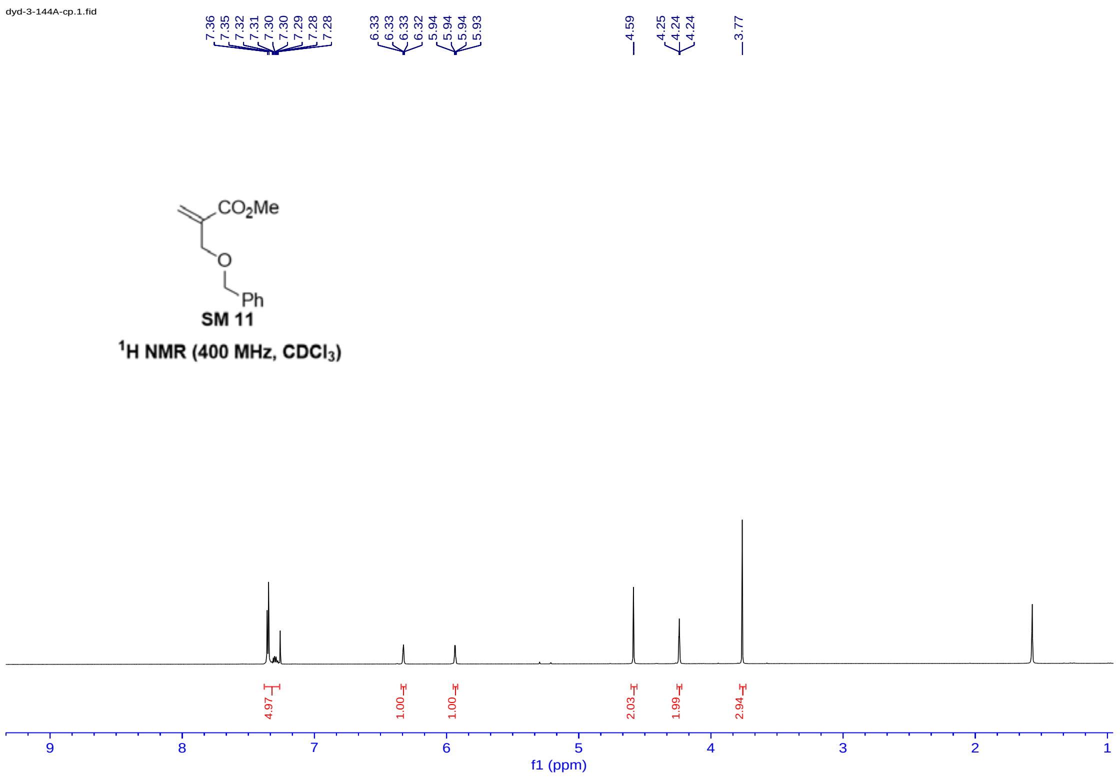

Supplementary Figure 36 ^{1}H NMR (400 MHz, 298K, $CDCl_3$) of **SM11**

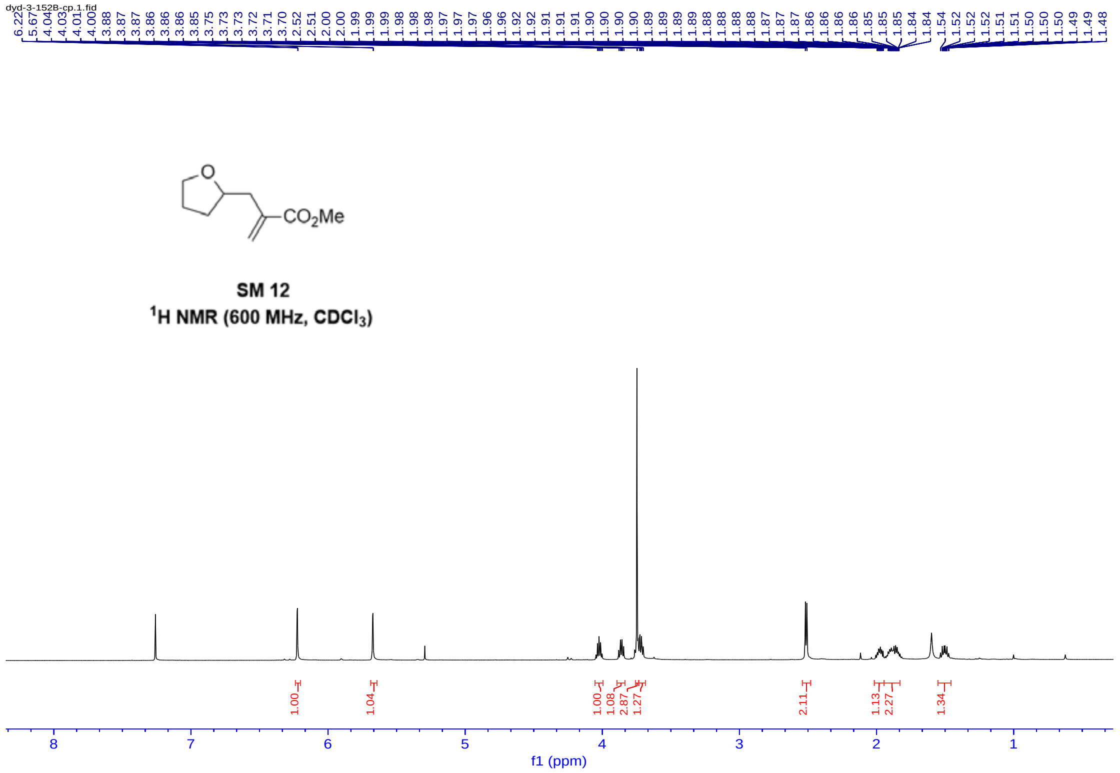

Supplementary Figure 37 ^{1}H NMR (600 MHz, 298K, $CDCl_3$) of **SM12**

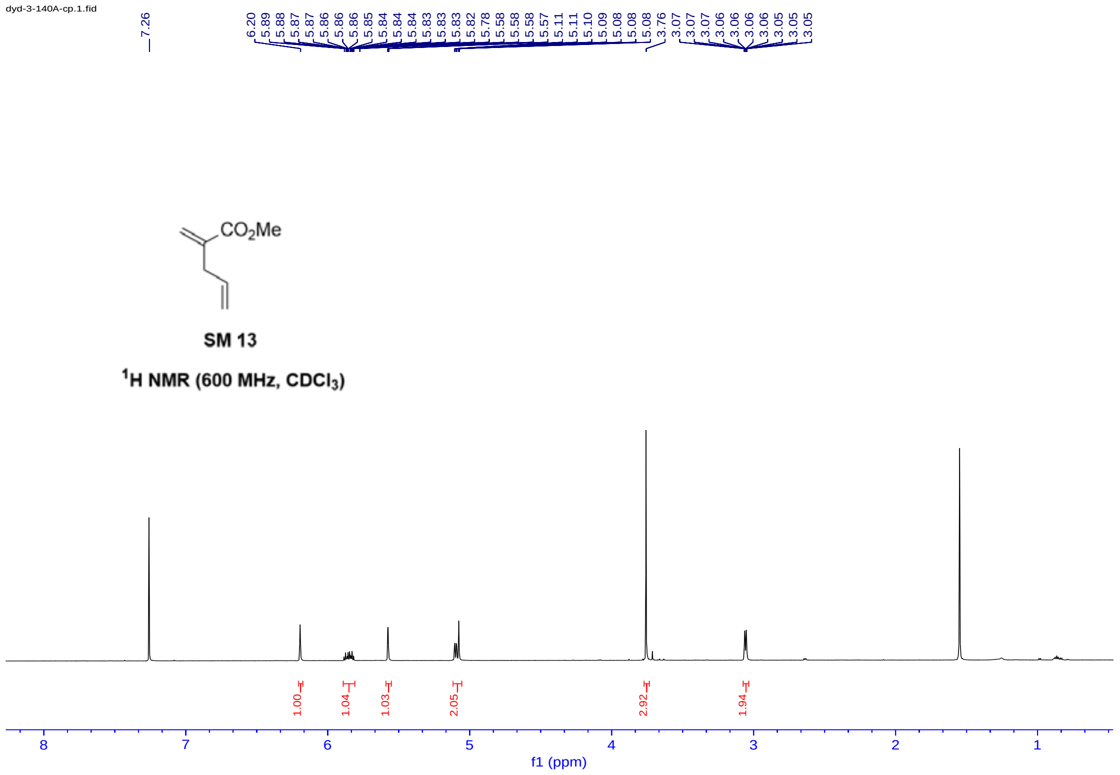

Supplementary Figure 38 ^{1}H NMR (600 MHz, 298K, $CDCl_3$) of **SM13**

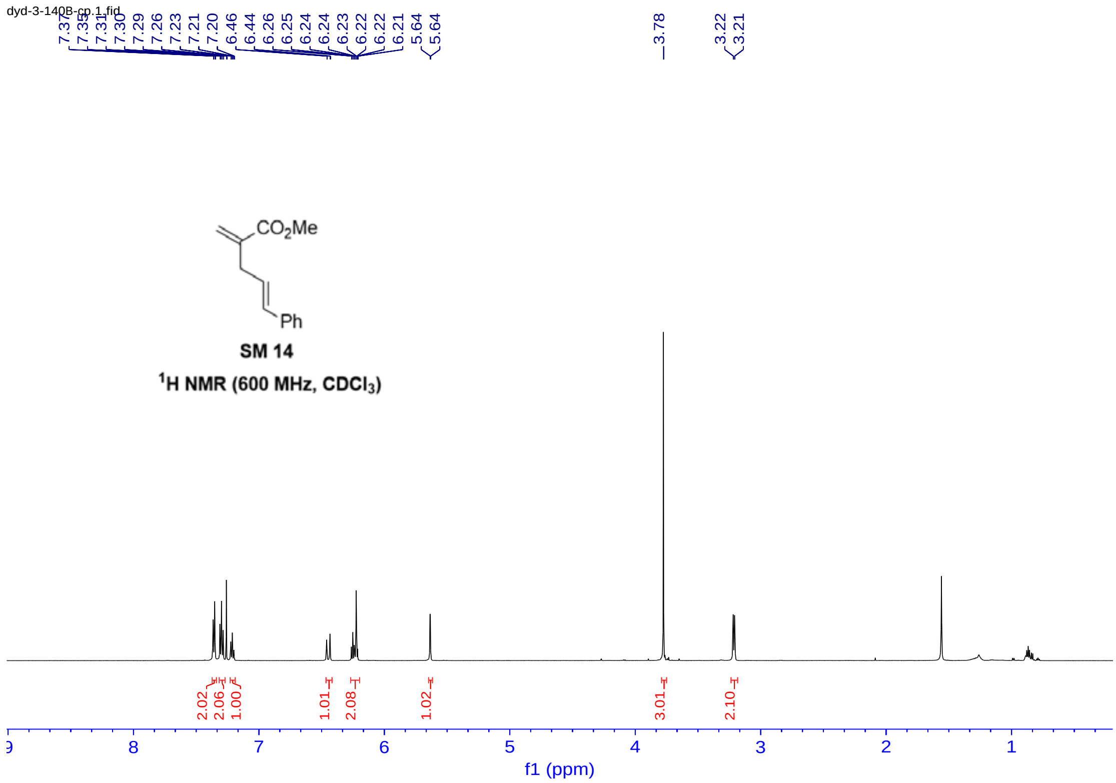

Supplementary Figure 39 ^{1}H NMR (600 MHz, 298K, $CDCl_3$) of **SM14**

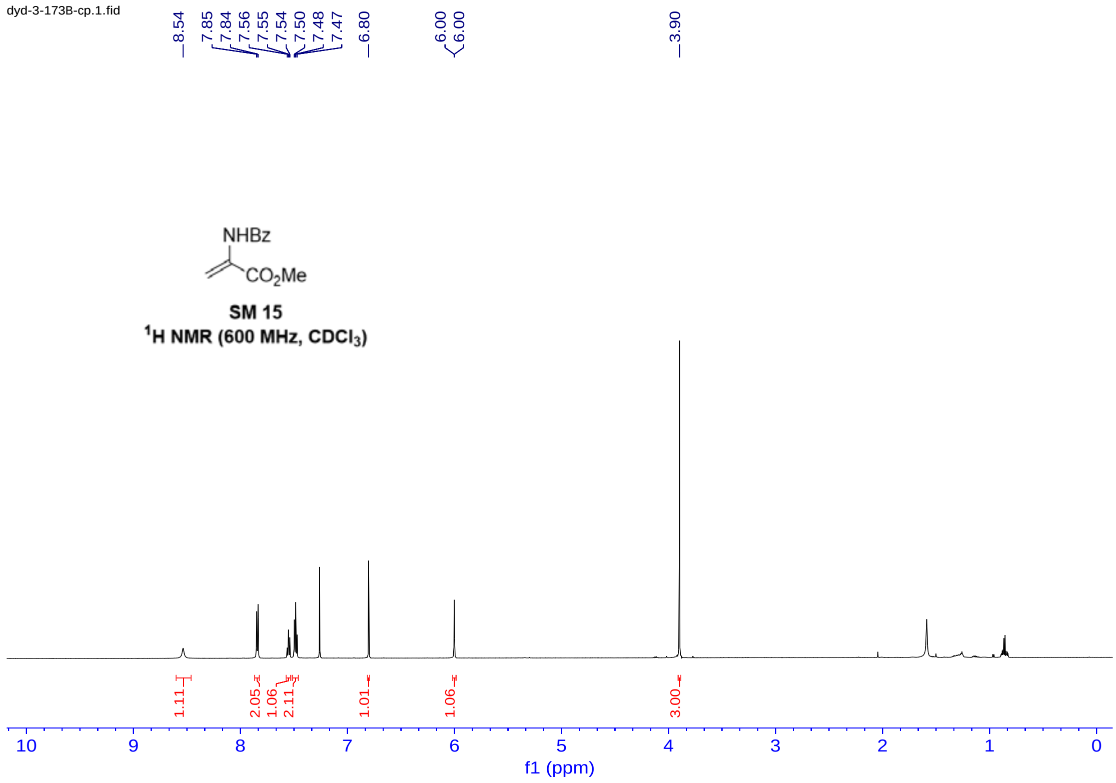

Supplementary Figure 40 ^{1}H NMR (600 MHz, 298K, $CDCl_3$) of **SM15**

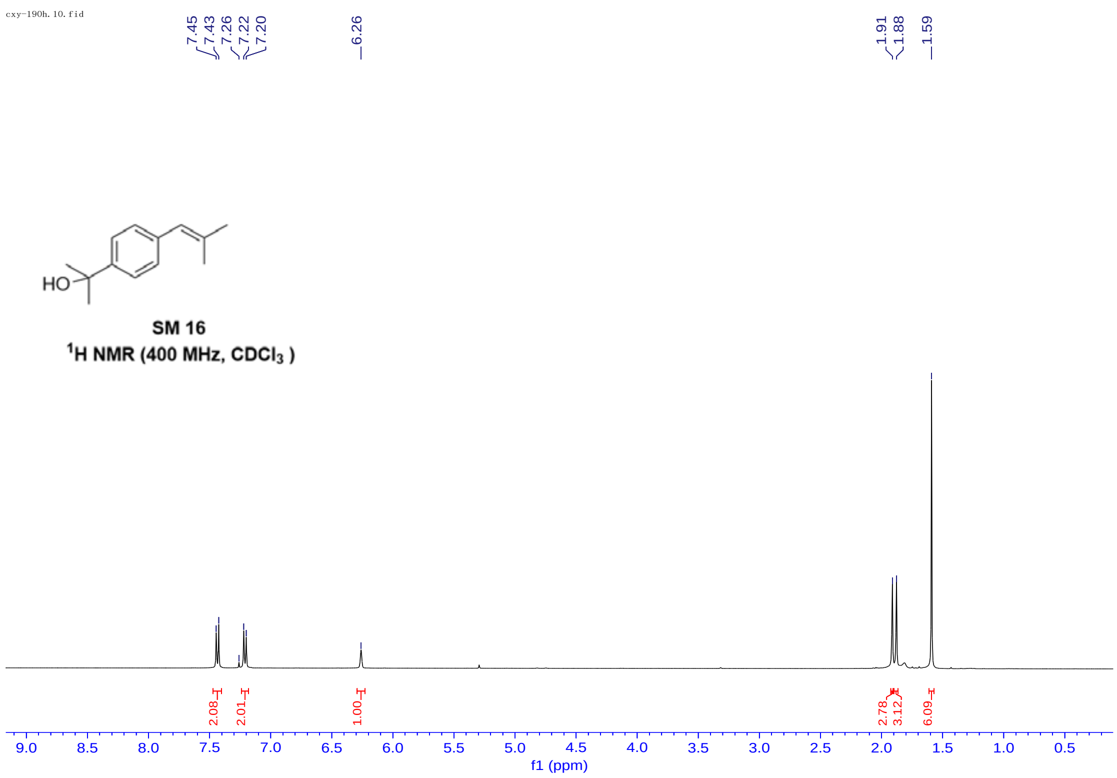

Supplementary Figure 41 ^{1}H NMR (400 MHz, 298K, $CDCl_3$) of **SM16**

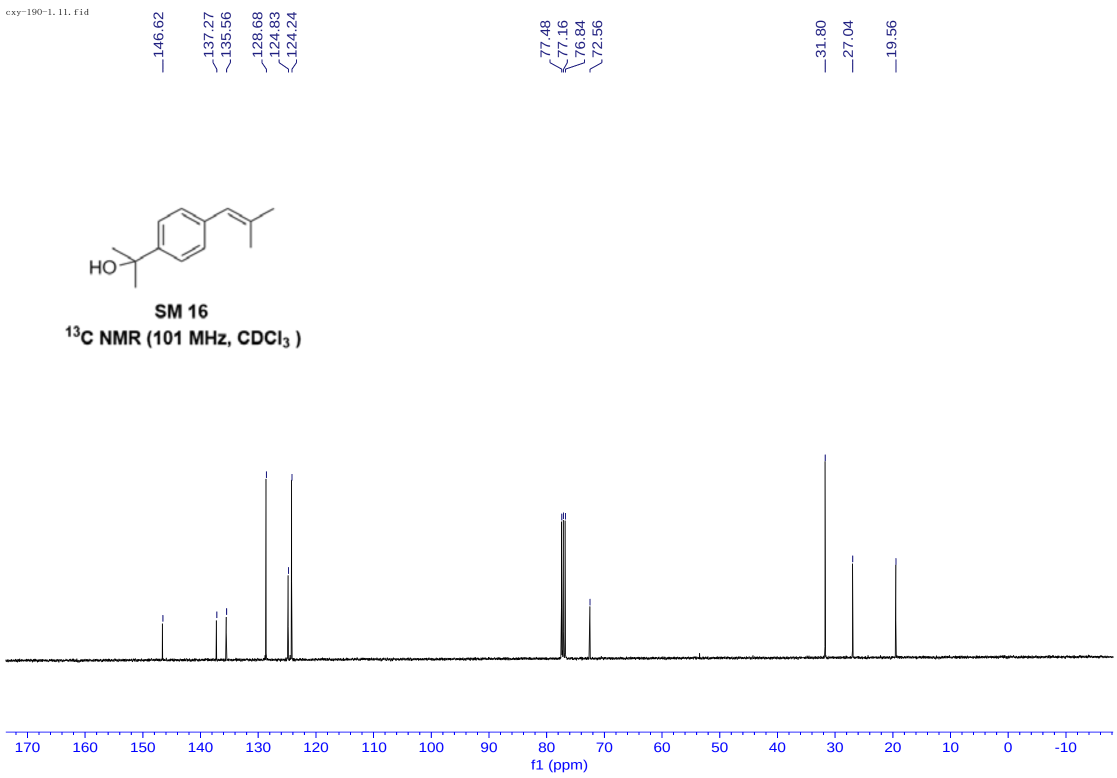

Supplementary Figure 42 ^{13}C NMR (101 MHz, 298K, $CDCl_3$) of **SM16**

cis-5-(4-fluorophenyl)-6,6-dimethyl-3-phenylpiperidin-2-one

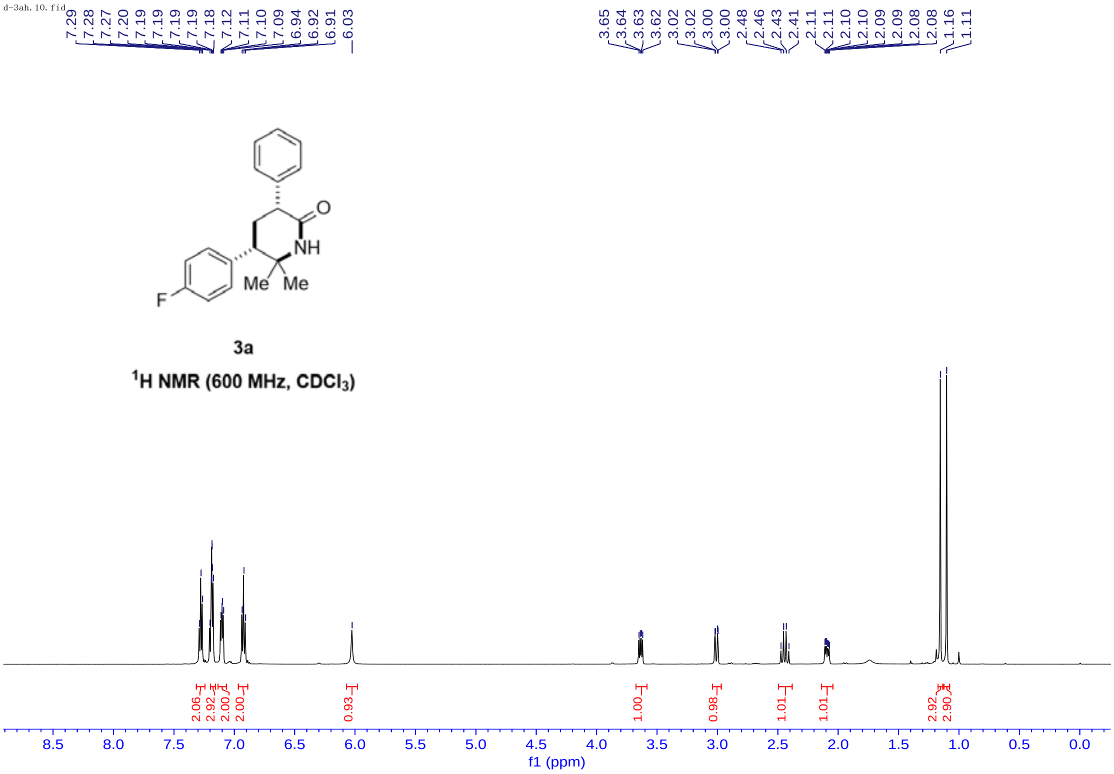

Supplementary Figure 43 ^{1}H NMR (600 MHz, 298K, $CDCl_3$) of **3a**

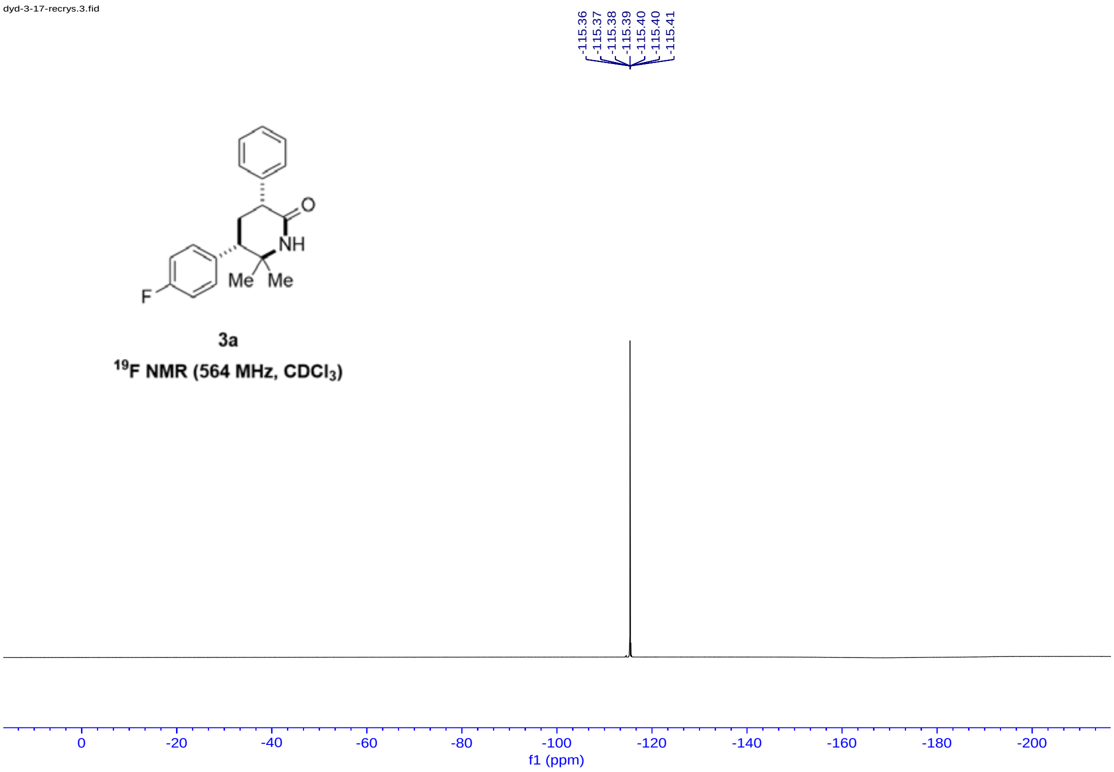

Supplementary Figure 44 ^{19}F NMR (564 MHz, 298K, $CDCl_3$) of **3a**

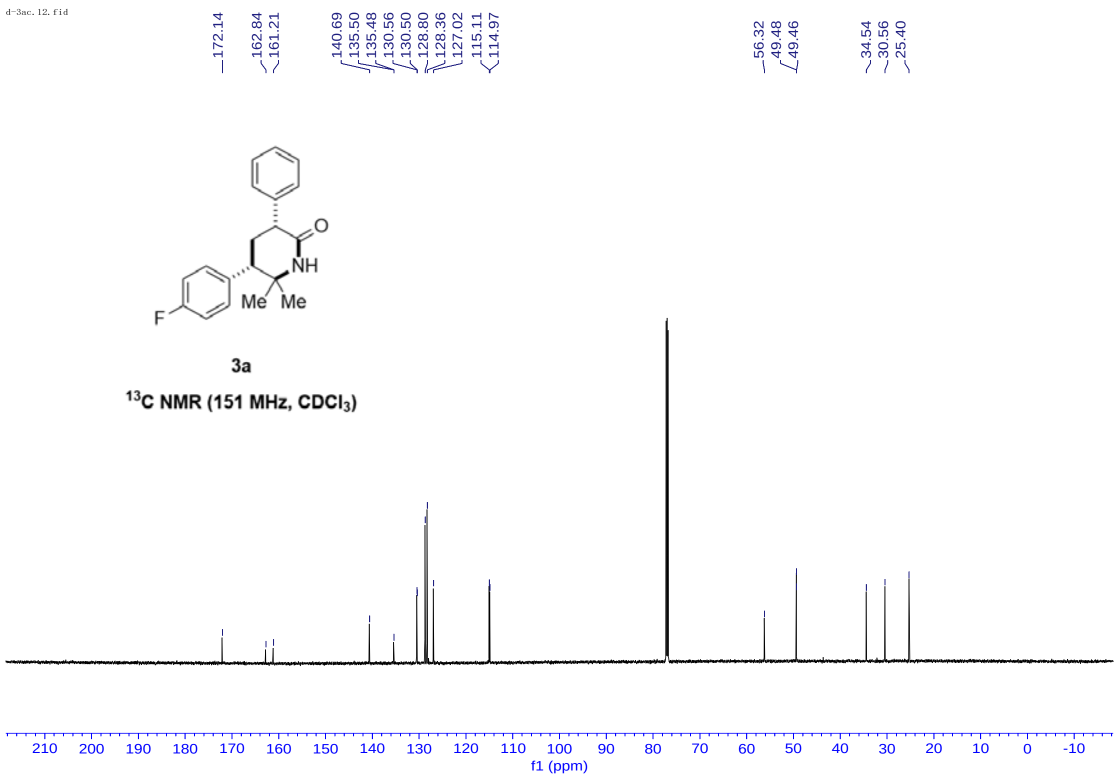

Supplementary Figure 45 ^{13}C NMR (151 MHz, 298K, $CDCl_3$) of **3a**

***trans*-6-(4-fluorophenyl)-5,5-dimethyl-3-phenylpiperidin-2-one**

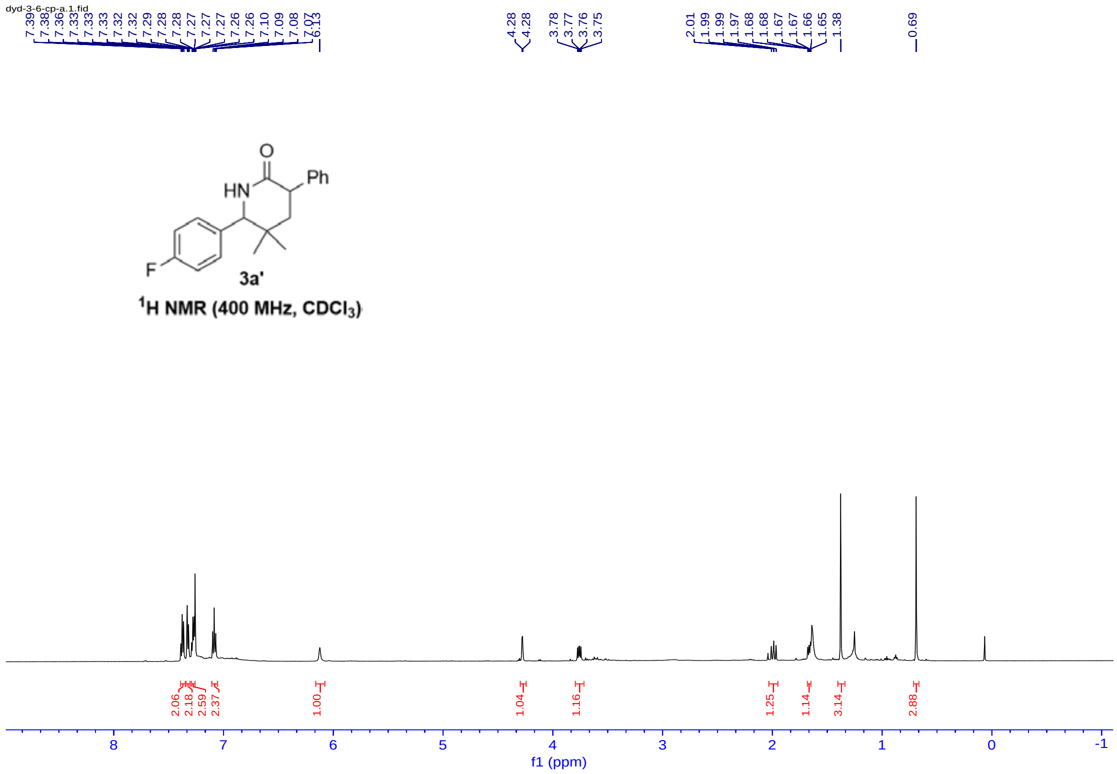

Supplementary Figure 46 ^{1}H NMR (400 MHz, 298K, $CDCl_3$) of **3a’**

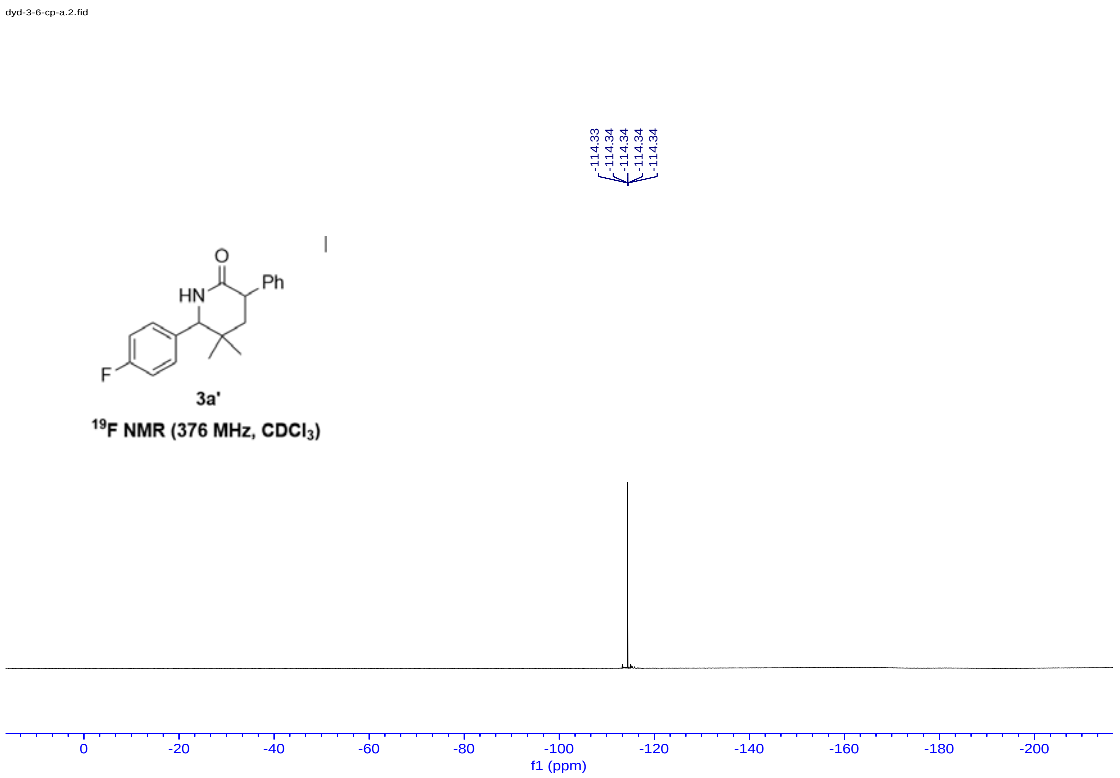

Supplementary Figure 47 ^{19}F NMR (376 MHz, 298K, $CDCl_3$) of **3a’**

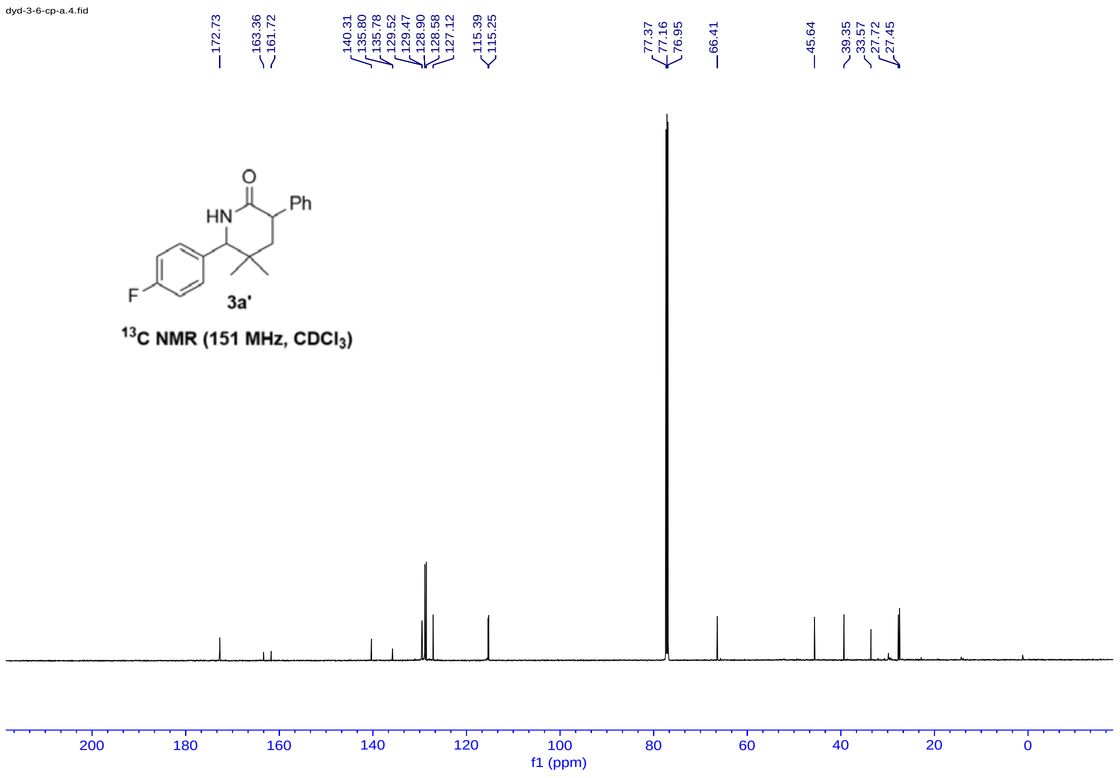

Supplementary Figure 48 ^{13}C NMR (151 MHz, 298K, $CDCl_3$) of **3a’**

5,5,6,6-tetramethyl-3-phenylpiperidin-2-one

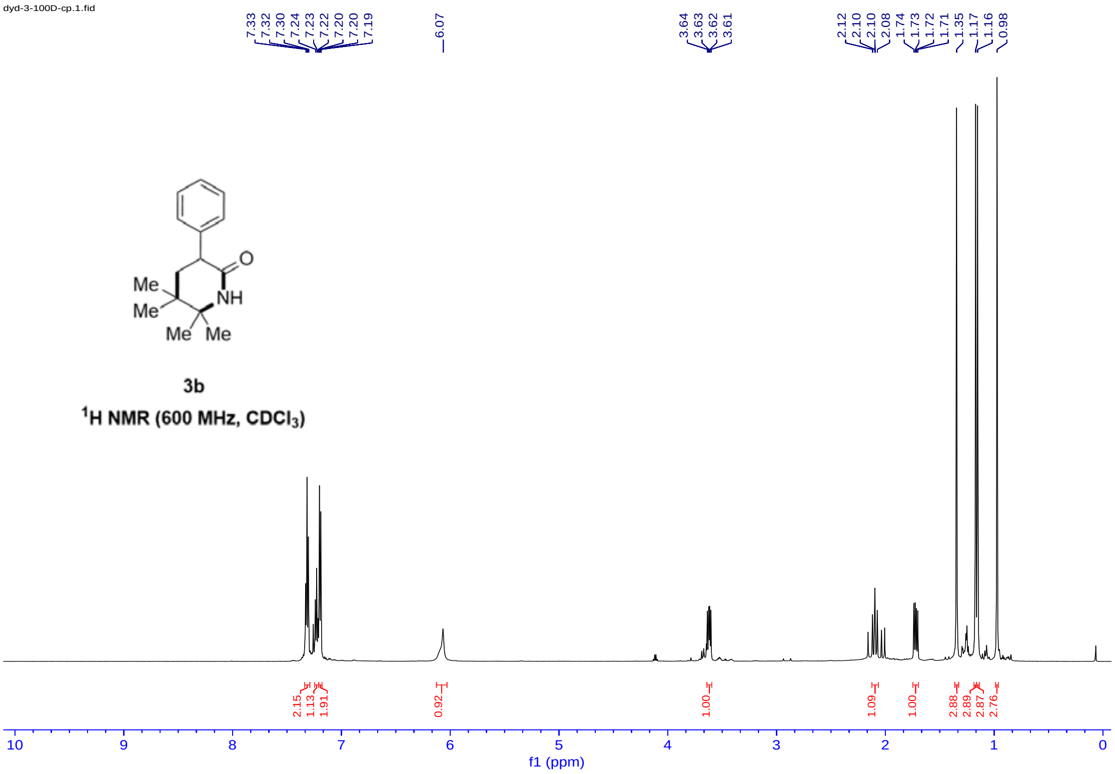

Supplementary Figure 49 ^{1}H NMR (600 MHz, 298K, $CDCl_3$) of **3b**

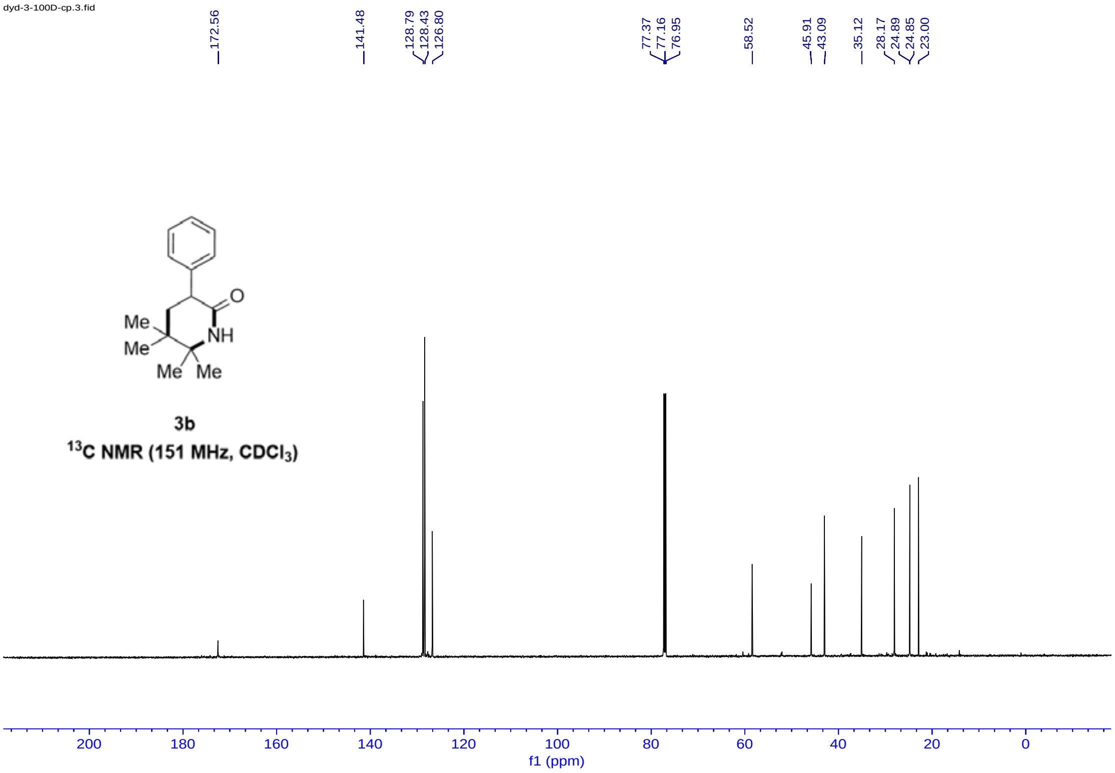

Supplementary Figure 50 ^{13}C NMR (151 MHz, 298K, $CDCl_3$) of **3b**

3-(4-fluorophenyl)-5,5,6,6-tetramethylpiperidin-2-one

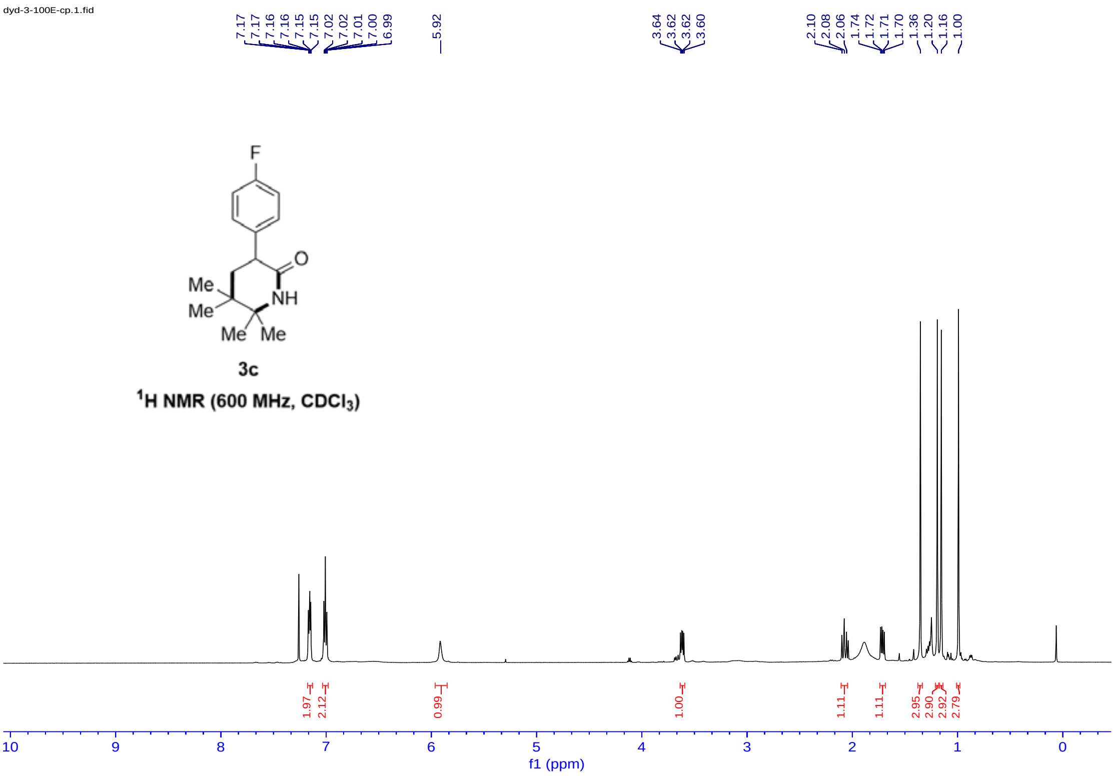

Supplementary Figure 51 ^{1}H NMR (600 MHz, 298K, $CDCl_3$) of **3c**

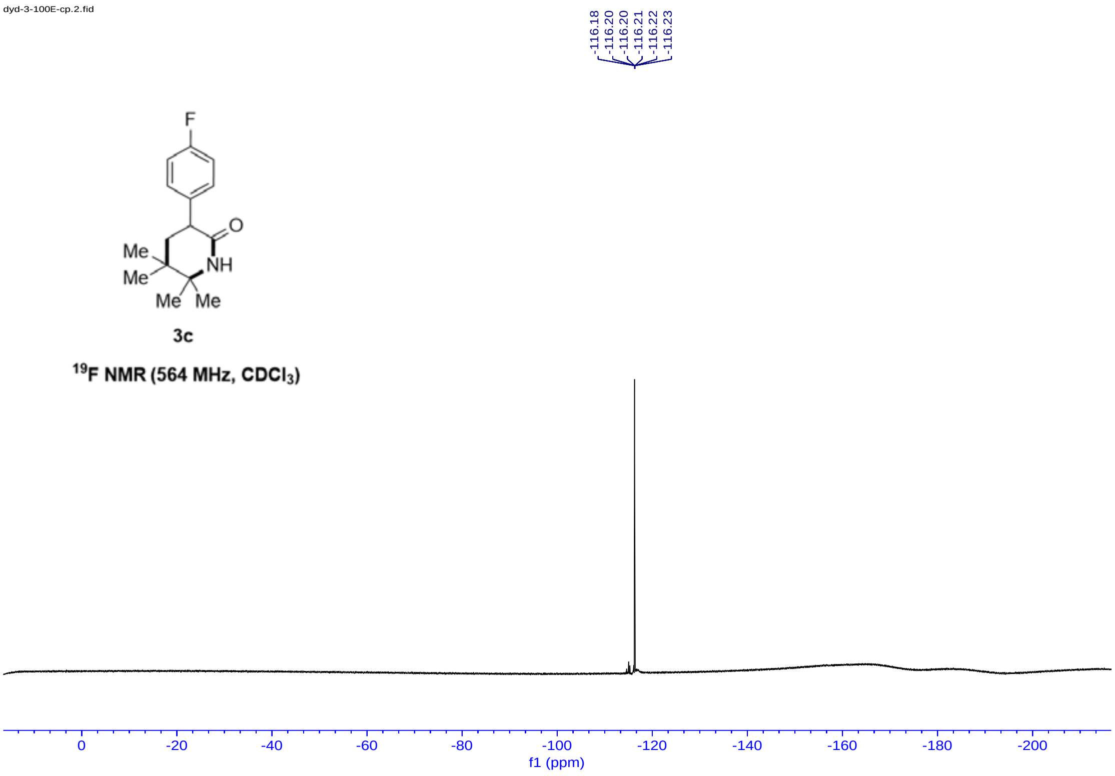

Supplementary Figure 52 ^{19}F NMR (564 MHz, 298K, $CDCl_3$) of **3c**

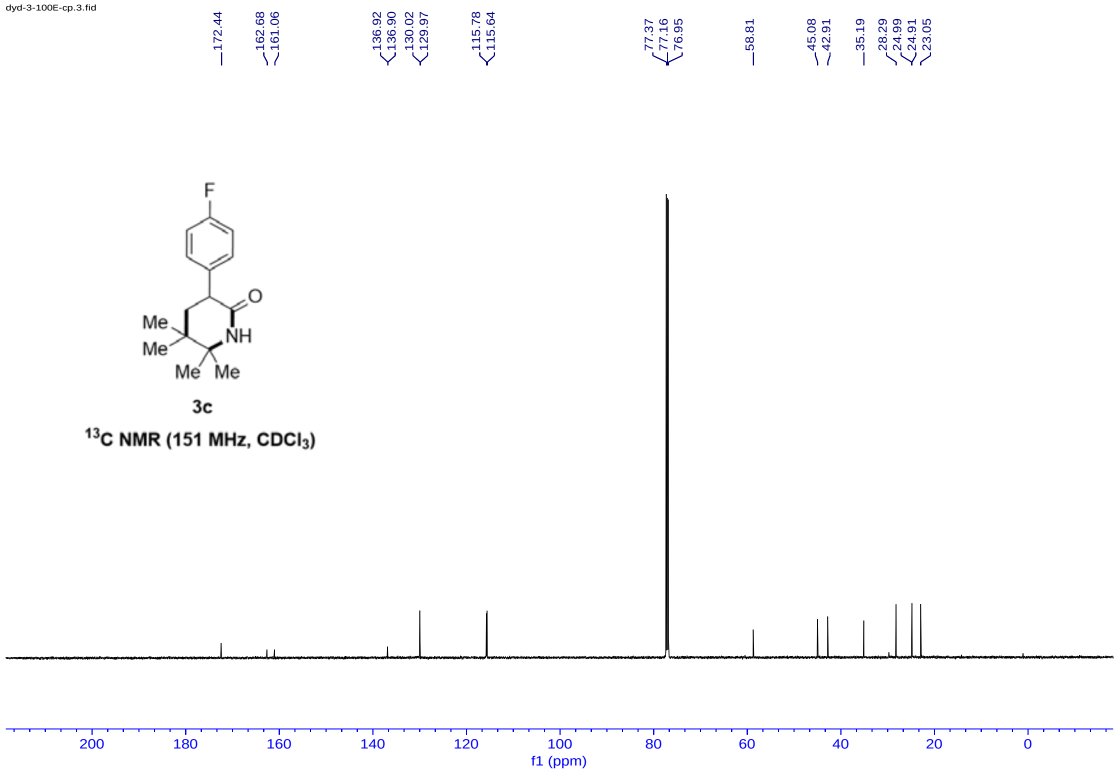

Supplementary Figure 53 ^{13}C NMR (151 MHz, 298K, $CDCl_3$) of **3c**

5,5,6,6-tetramethyl-3-(4-(trifluoromethyl)phenyl)piperidin-2-one

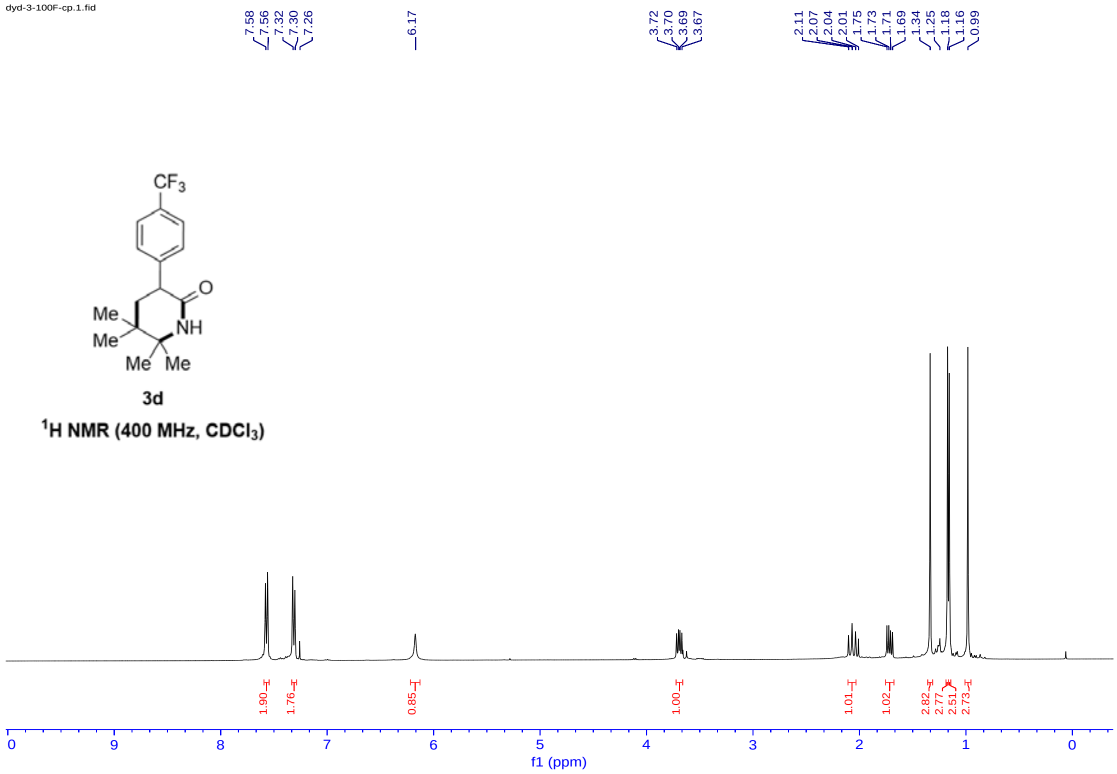

Supplementary Figure 54 ^{1}H NMR (400 MHz, 298K, $CDCl_3$) of **3d**

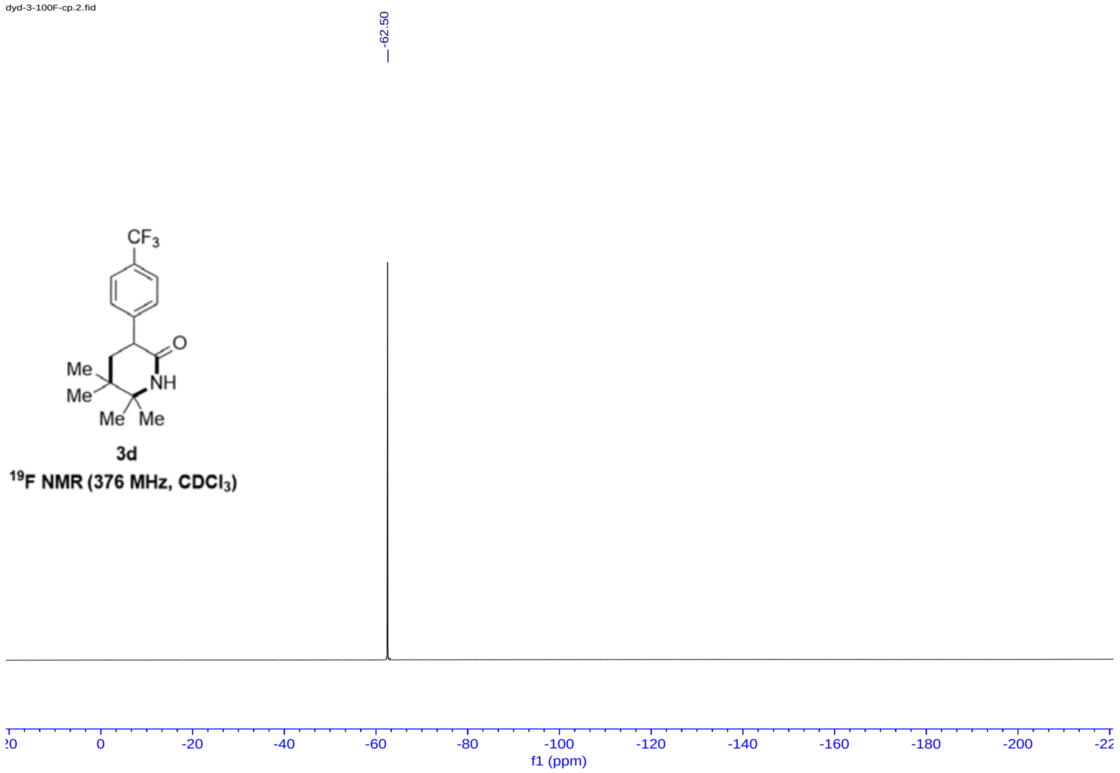

Supplementary Figure 55 ^{19}F NMR (376 MHz, 298K, $CDCl_3$) of **3d**

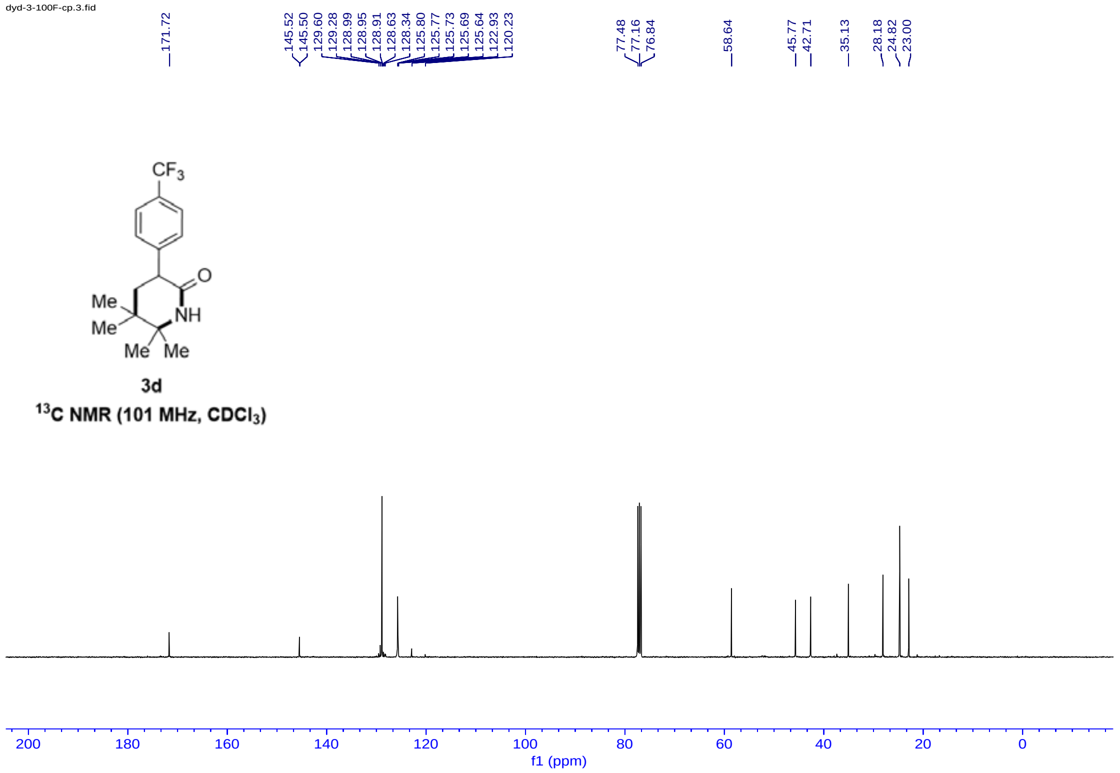

Supplementary Figure 56 ^{13}C NMR (101 MHz, 298K, $CDCl_3$) of **3d**

3-(4-methoxyphenyl)-5,5,6,6-tetramethylpiperidin-2-one

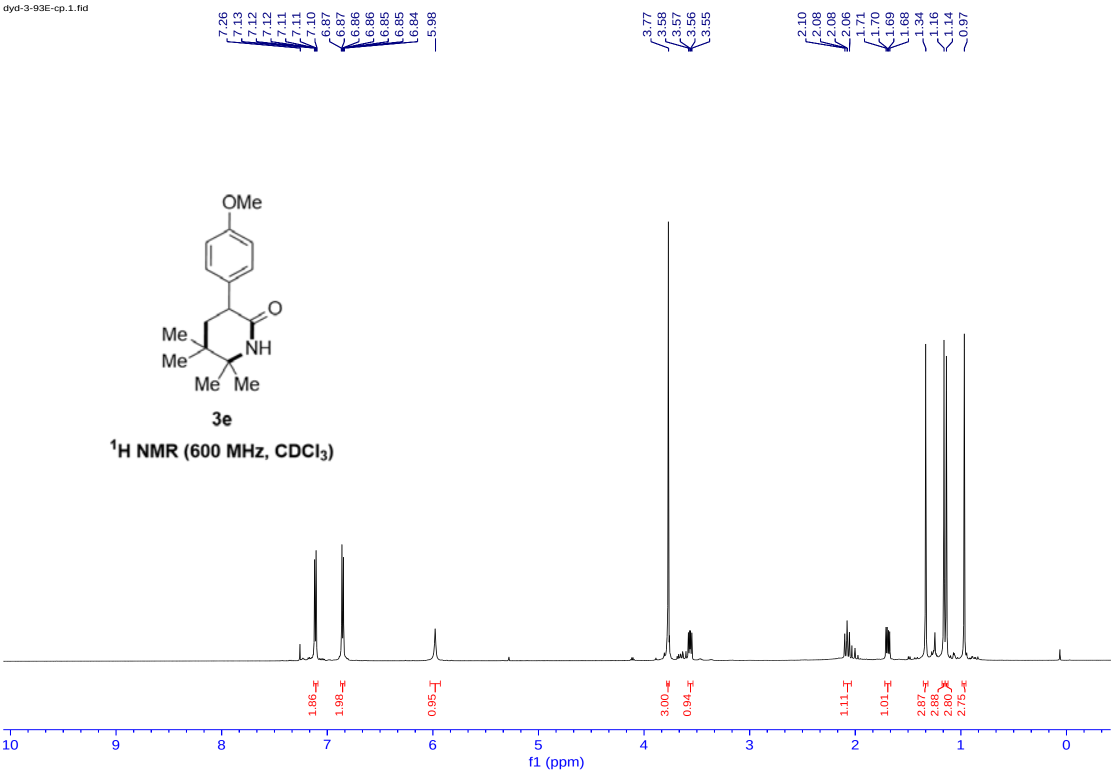

Supplementary Figure 57 ^{1}H NMR (600 MHz, 298K, $CDCl_3$) of **3e**

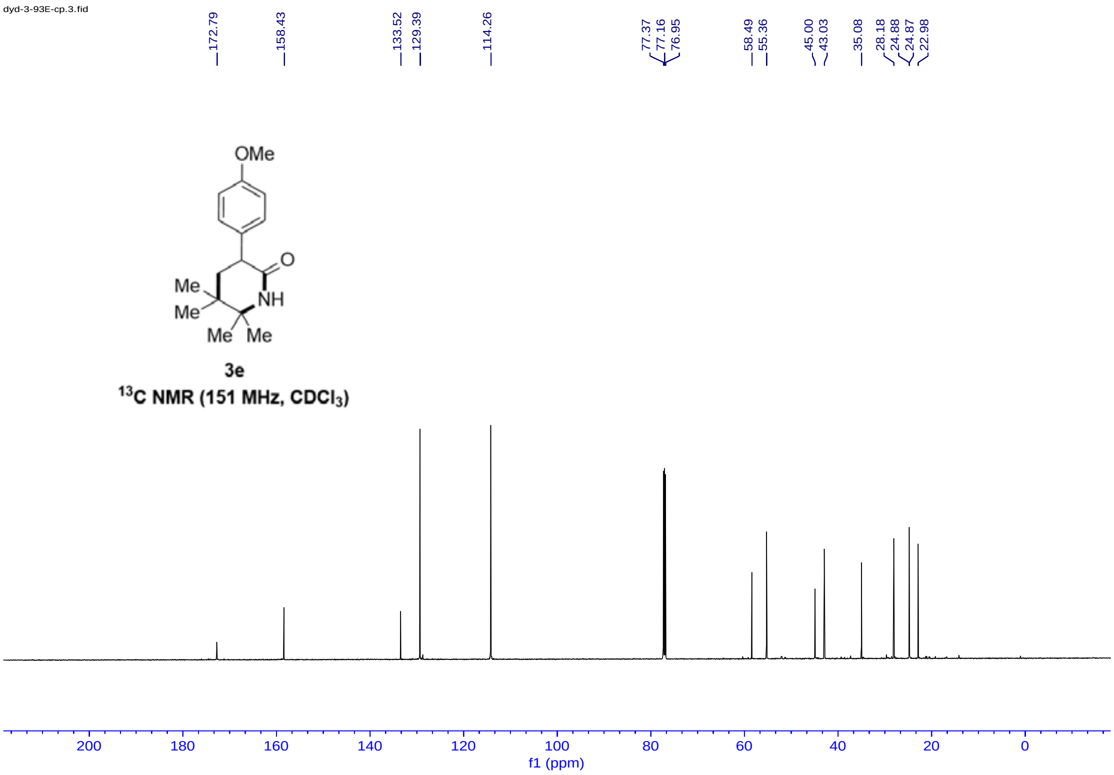

Supplementary Figure 58 ^{13}C NMR (151 MHz, 298K, $CDCl_3$) of **3e**

3-(2-fluorophenyl)-5,5,6,6-tetramethylpiperidin-2-one

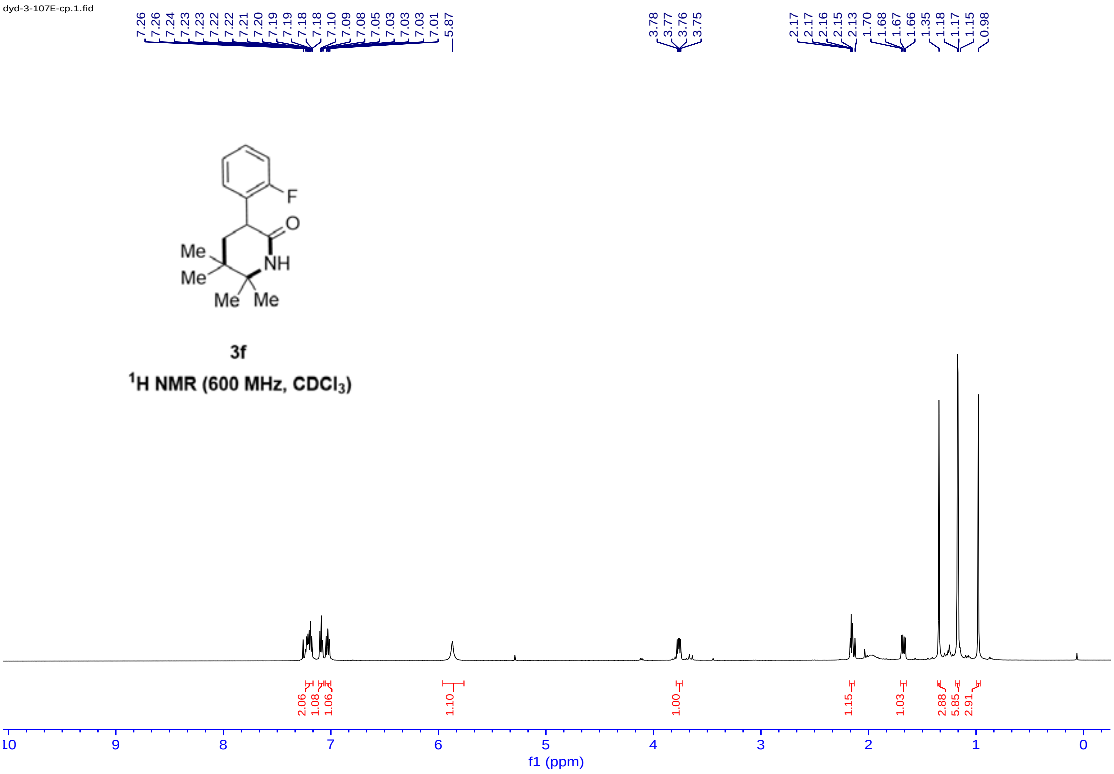

Supplementary Figure 59 ^{1}H NMR (600 MHz, 298K, CDCl$_3$) of **3f**

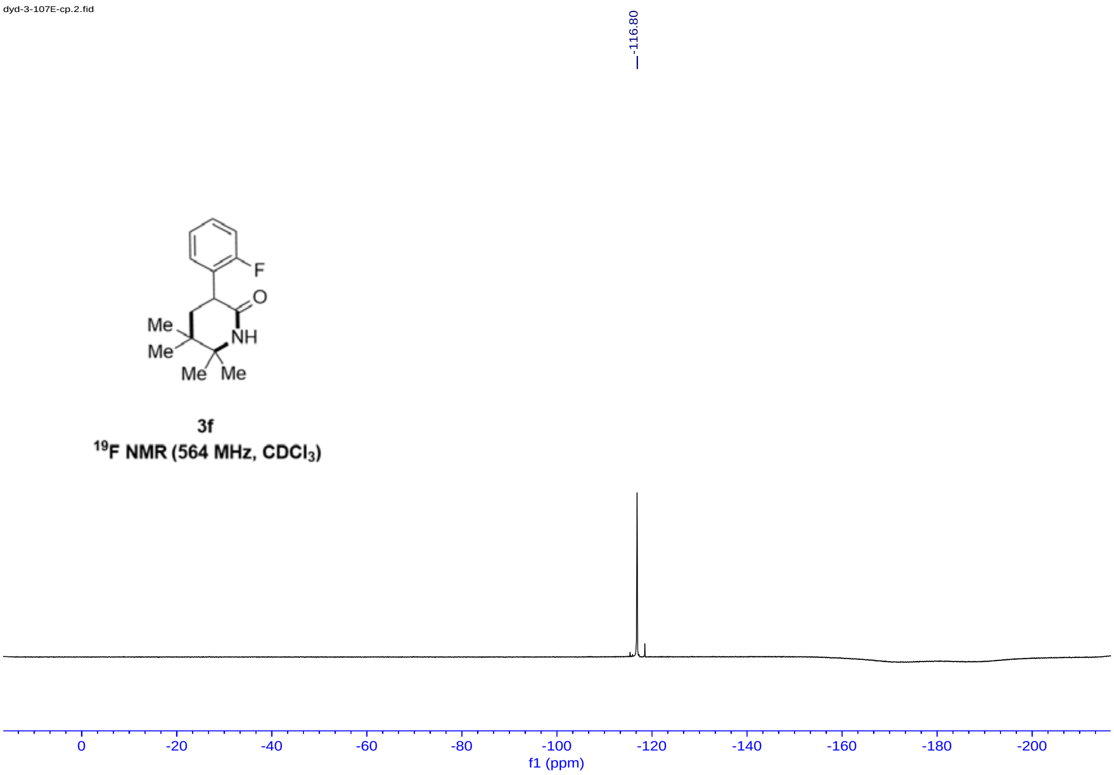

Supplementary Figure 60 ^{19}F NMR (564 MHz, 298K, CDCl$_3$) of **3f**

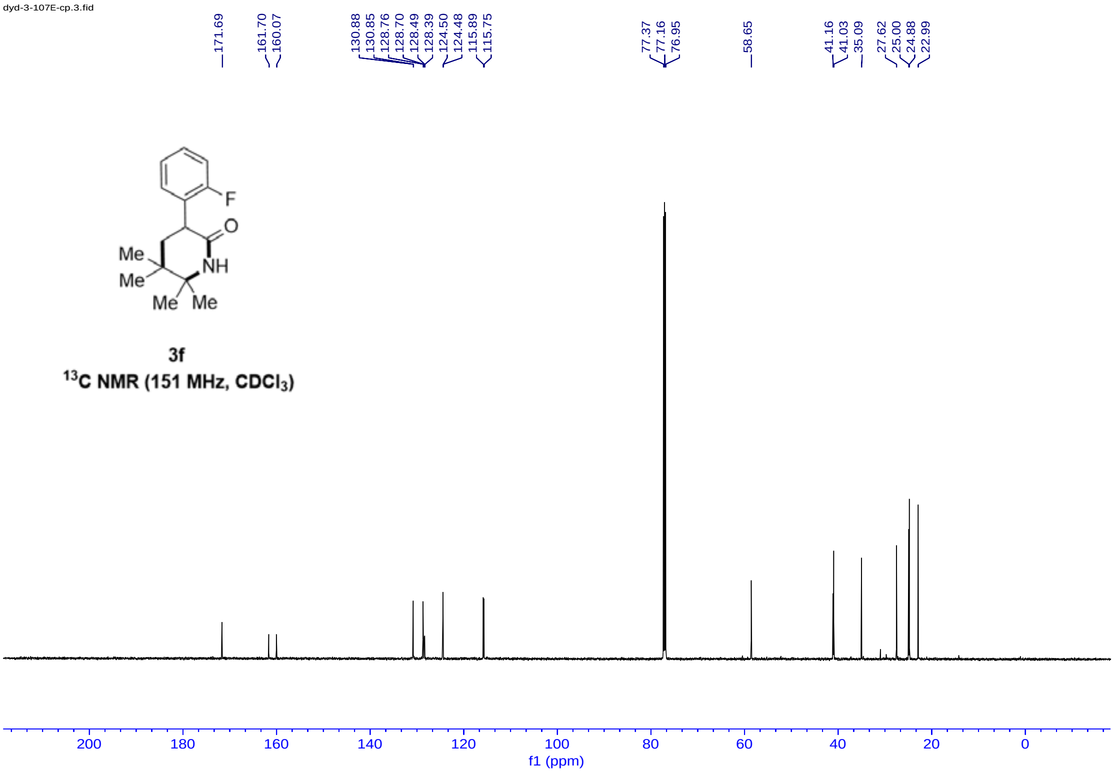

Supplementary Figure 61 ^{13}C NMR (151 MHz, 298K, $CDCl_3$) of **3f**

3-(2-methoxyphenyl)-5,5,6,6-tetramethylpiperidin-2-one

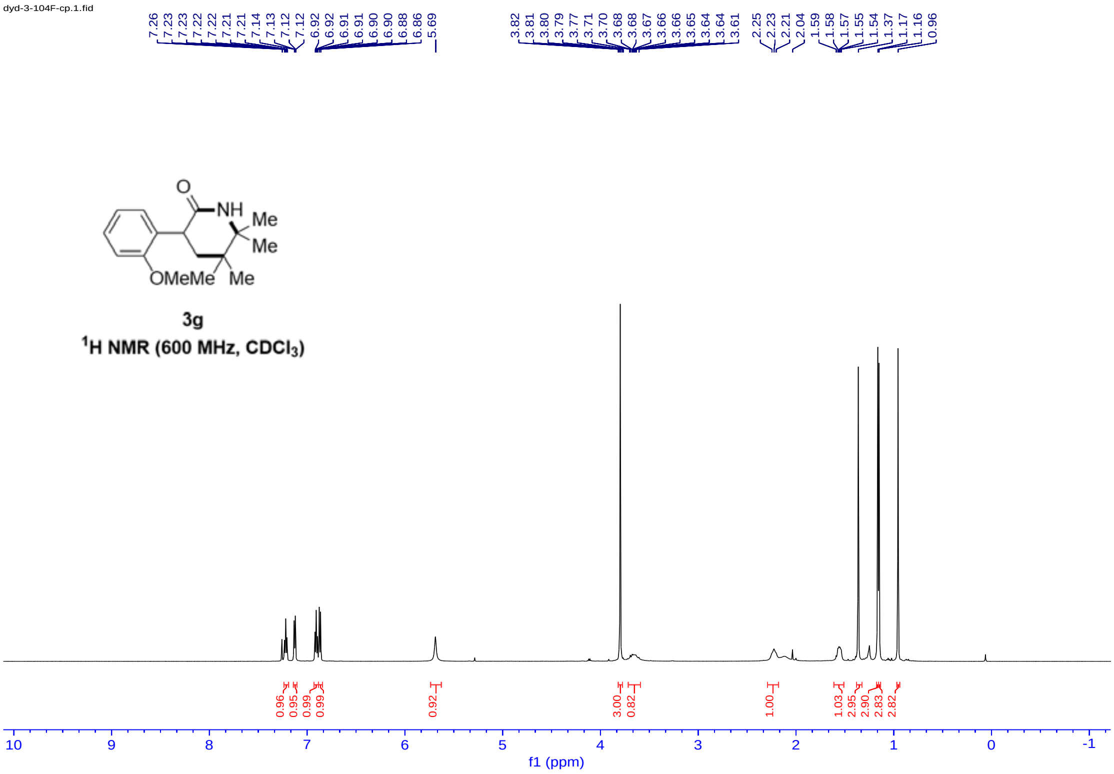

Supplementary Figure 62 ^{1}H NMR (600 MHz, 298K, $CDCl_3$) of **3g**

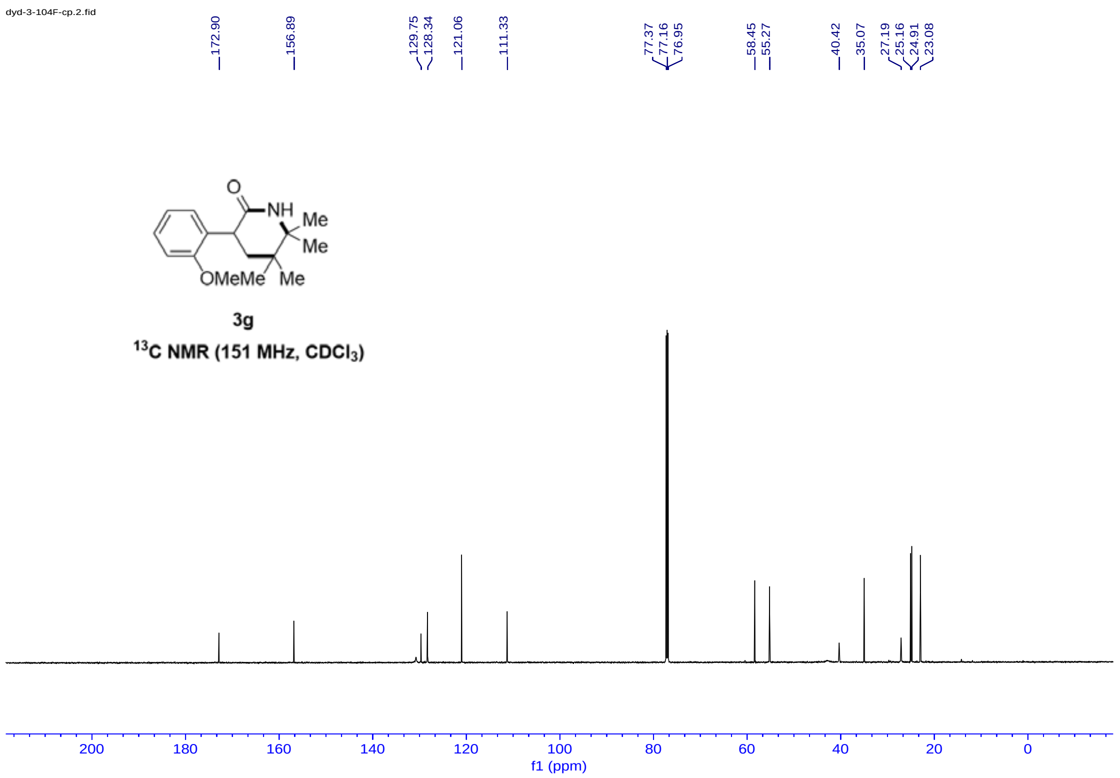

Supplementary Figure 63 ^{13}C NMR (151 MHz, 298K, $CDCl_3$) of **3g**

3-(3-methoxyphenyl)-5,5,6,6-tetramethylpiperidin-2-one

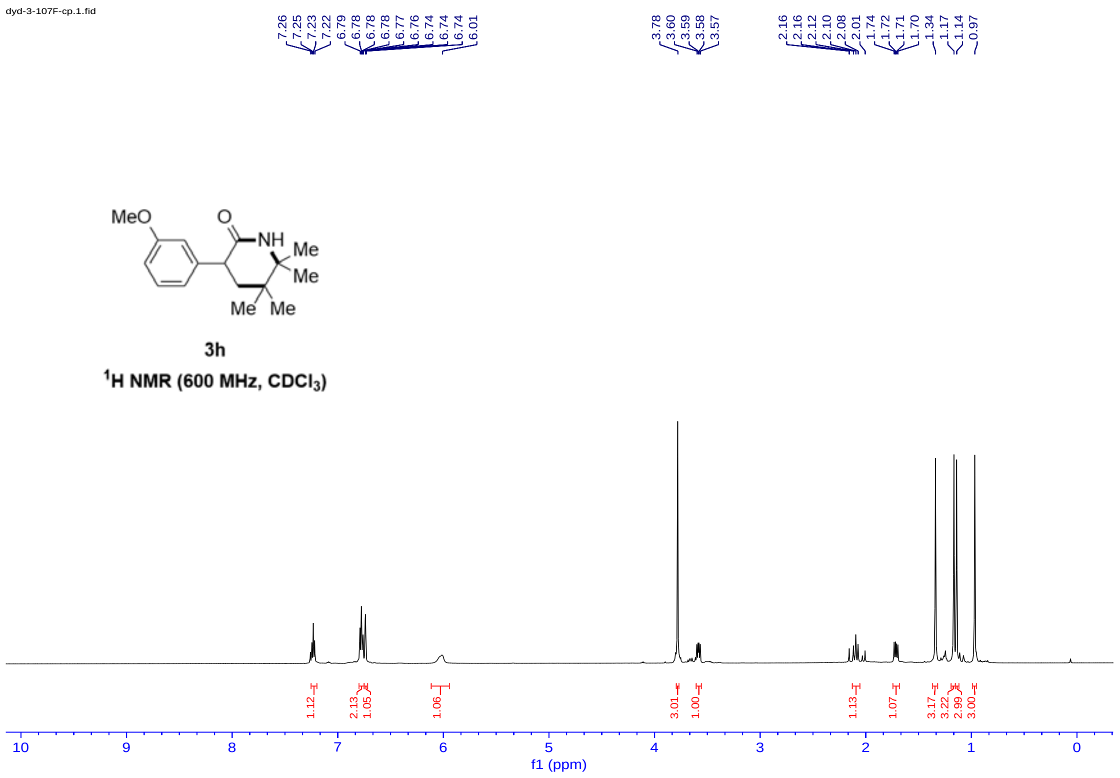

Supplementary Figure 64 ^{1}H NMR (600 MHz, 298K, $CDCl_3$) of **3h**

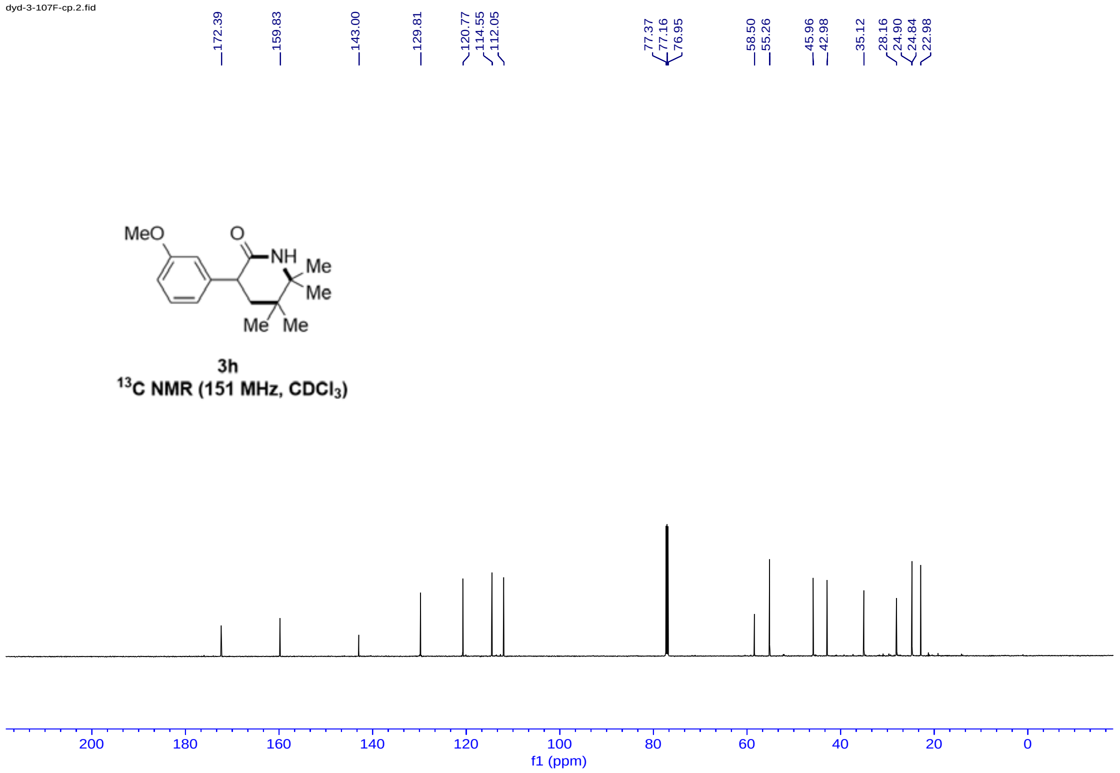

Supplementary Figure 65 ^{13}C NMR (151 MHz, 298K, $CDCl_3$) of **3h**

5,5,6,6-tetramethyl-3-(naphthalen-2-yl)piperidin-2-one

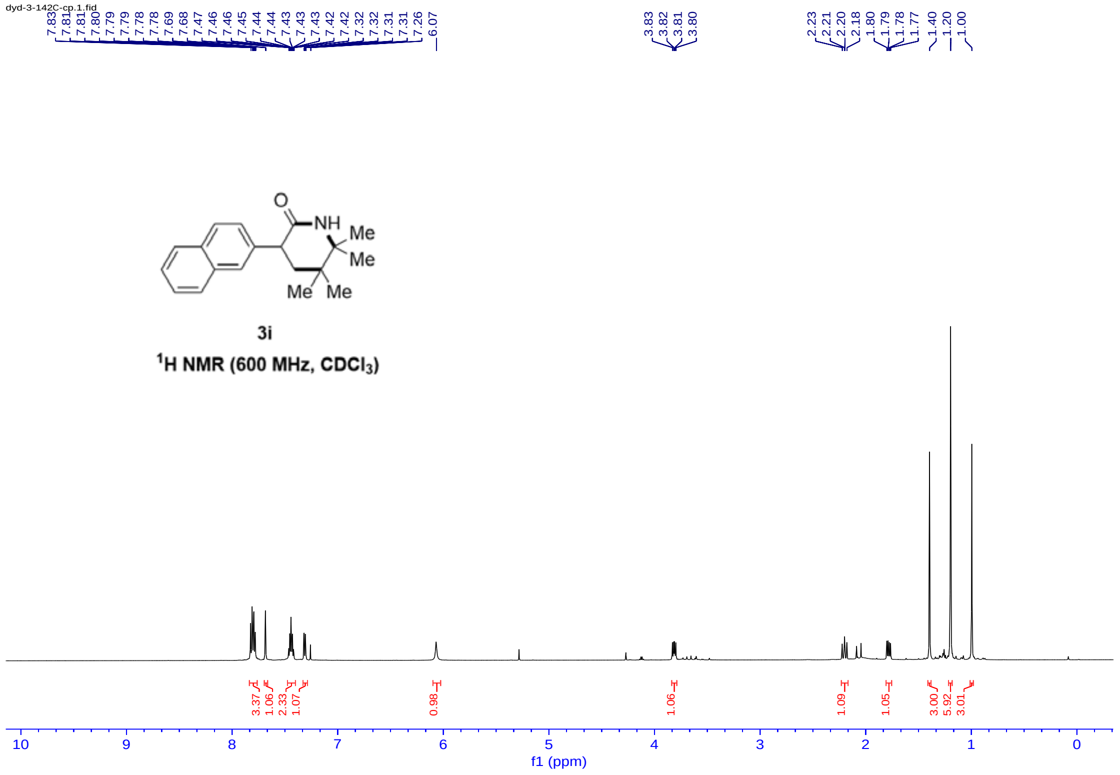

Supplementary Figure 66 ^{1}H NMR (600 MHz, 298K, $CDCl_3$) of **3i**

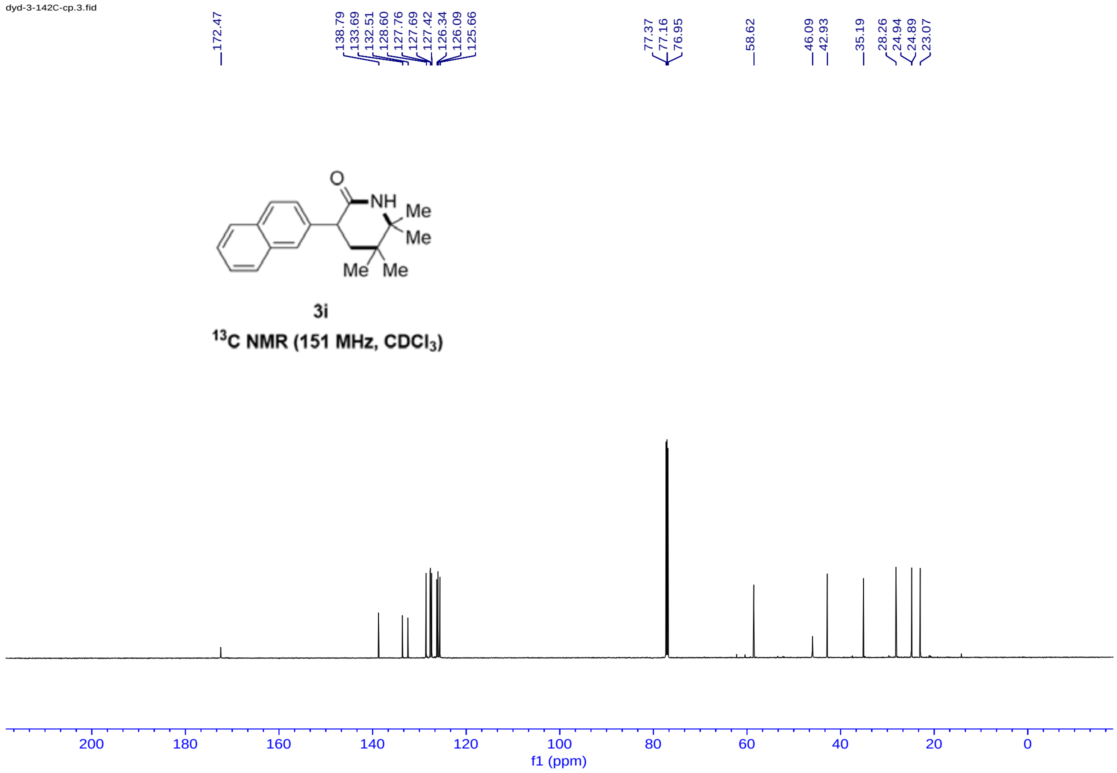

Supplementary Figure 67 ^{13}C NMR (151 MHz, 298K, $CDCl_3$) of **3i**

5,5,6,6-tetramethylpiperidin-2-one

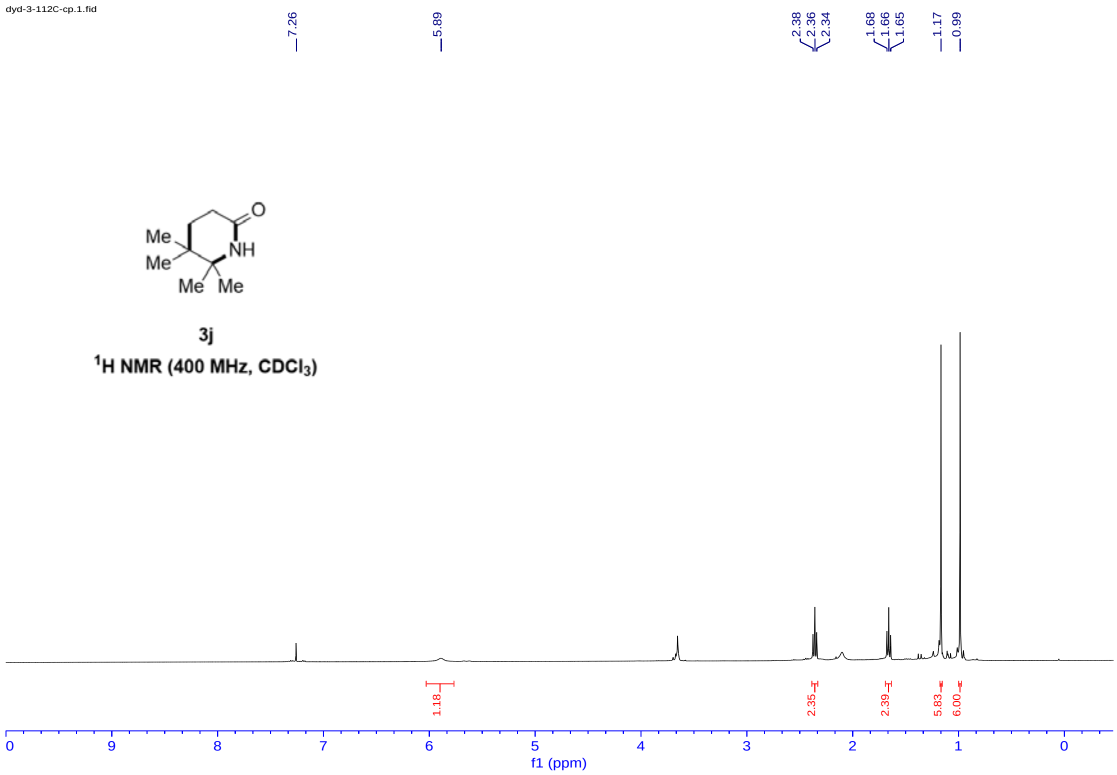

Supplementary Figure 68 ^{1}H NMR (400 MHz, 298K, $CDCl_3$) of **3j**

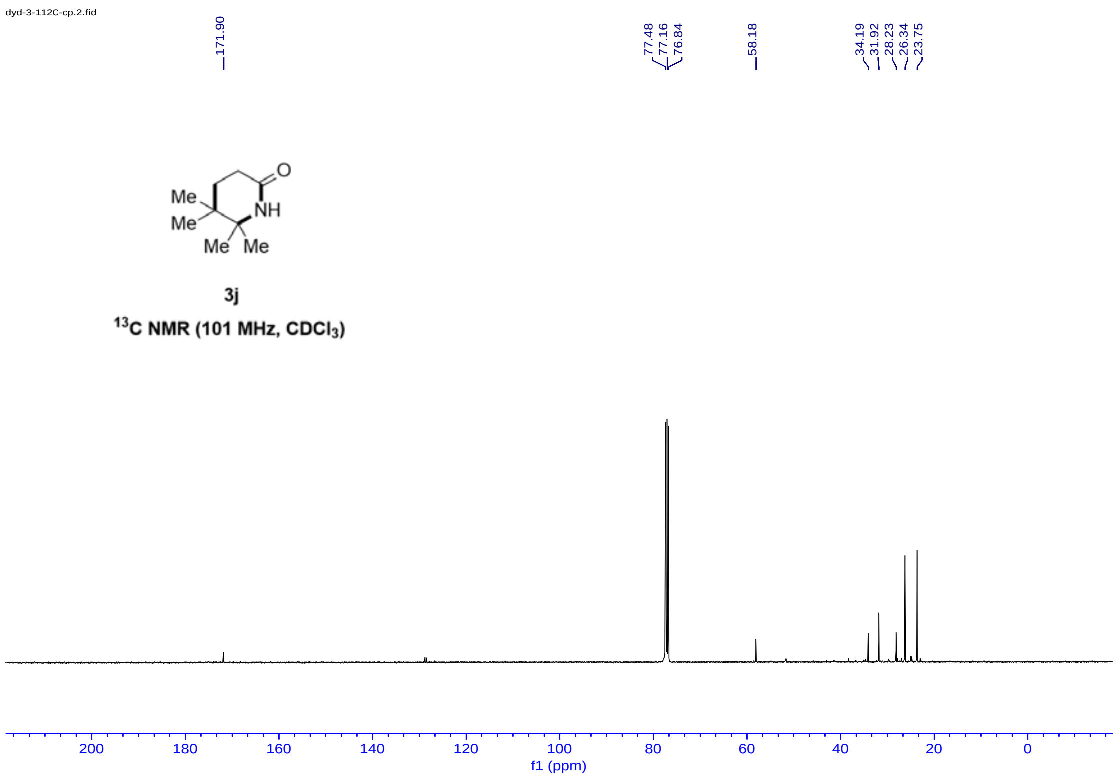

Supplementary Figure 69 ^{13}C NMR (101 MHz, 298K, $CDCl_3$) of **3j**

3-fluoro-5,5,6,6-tetramethylpiperidin-2-one

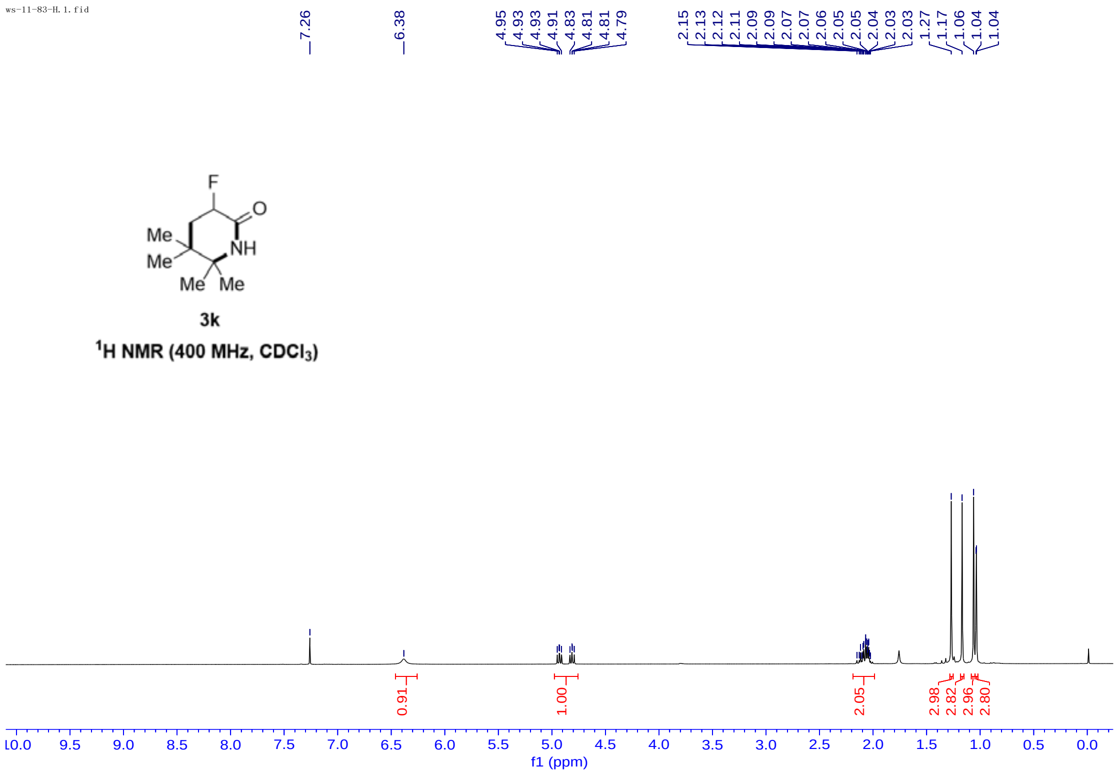

Supplementary Figure 70 ^{1}H NMR (400 MHz, 298K, $CDCl_3$) of **3k**

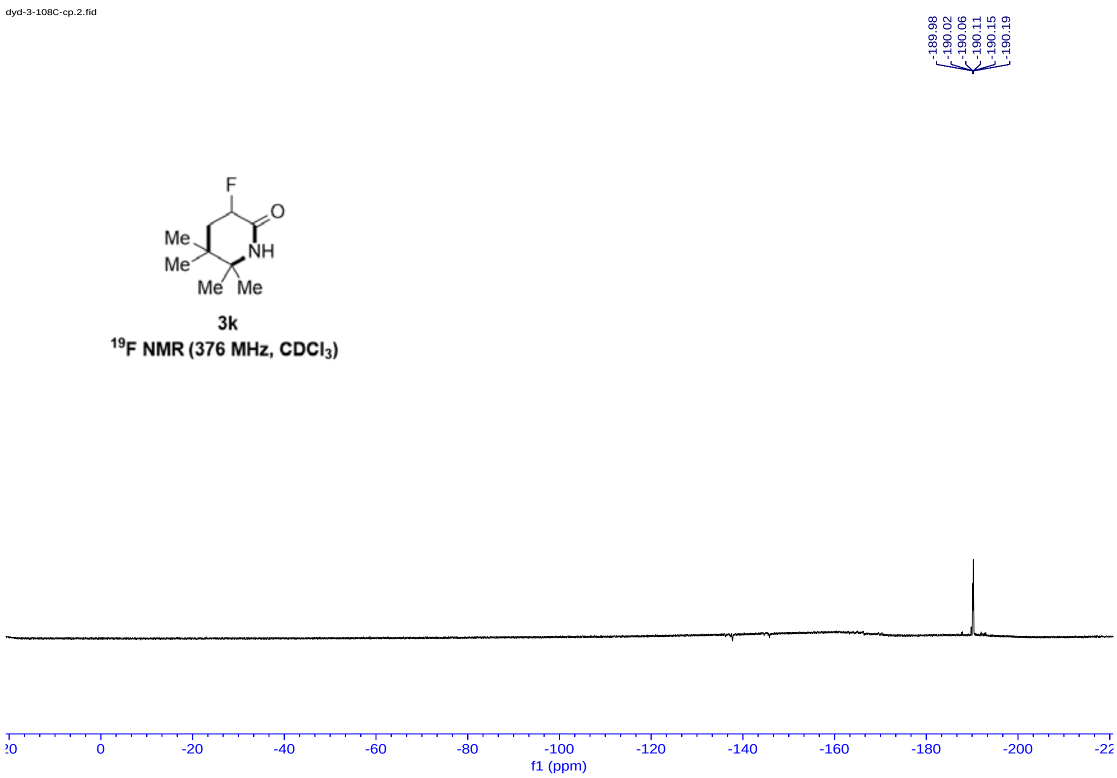

Supplementary Figure 71 ^{19}F NMR (376 MHz, 298K, $CDCl_3$) of **3k**

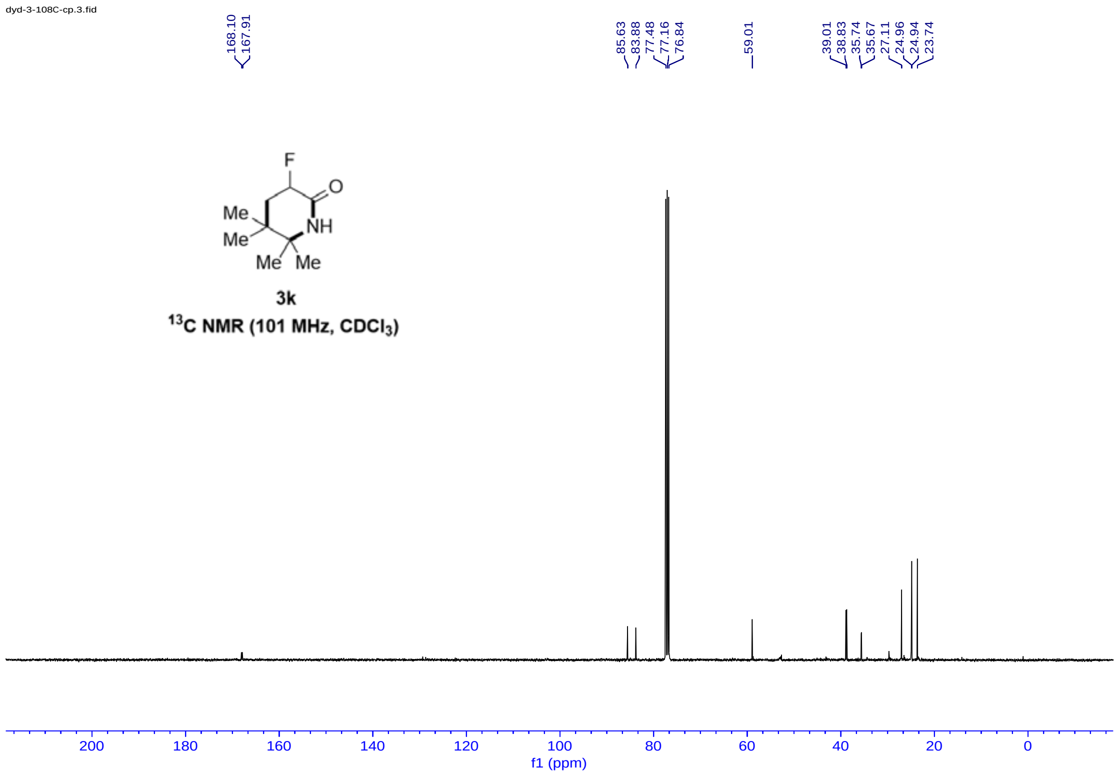

Supplementary Figure 72 ^{13}C NMR (101 MHz, 298K, $CDCl_3$) of **3k**

3,5,5,6,6-pentamethylpiperidin-2-one

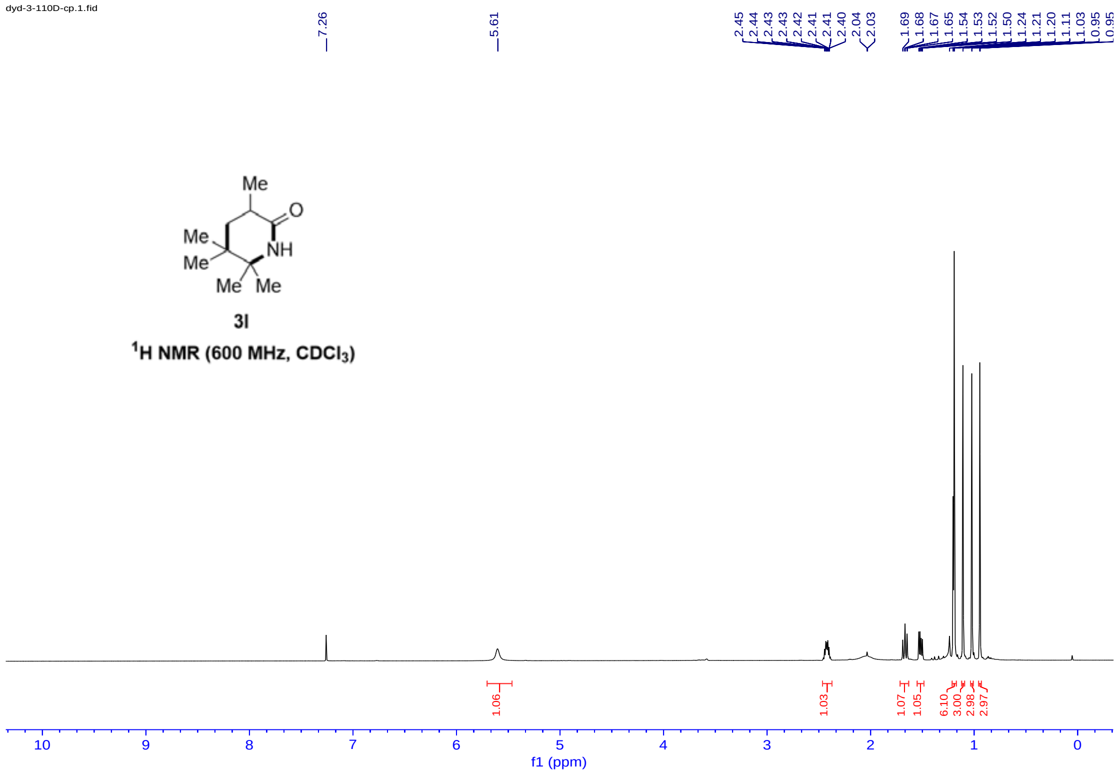

Supplementary Figure 73 ^{1}H NMR (600 MHz, 298K, $CDCl_3$) of **3l**

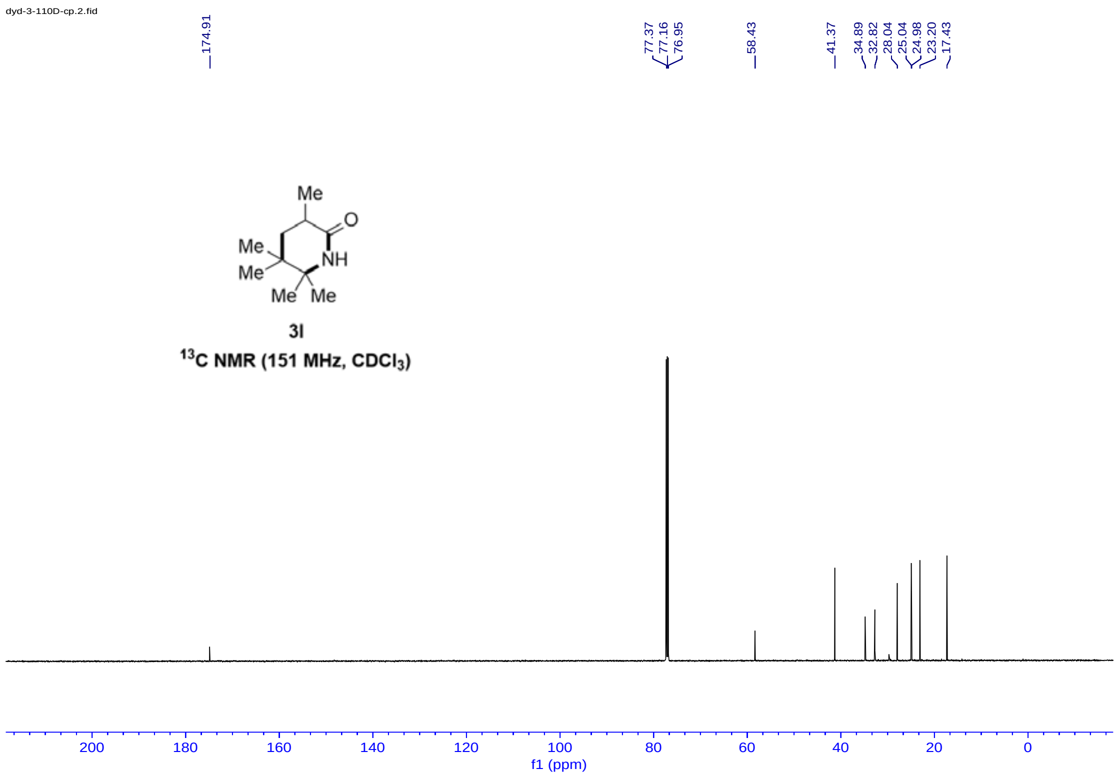

Supplementary Figure 74 ^{13}C NMR (151 MHz, 298K, $CDCl_3$) of **3l**

3-benzyl-5,5,6,6-tetramethylpiperidin-2-one

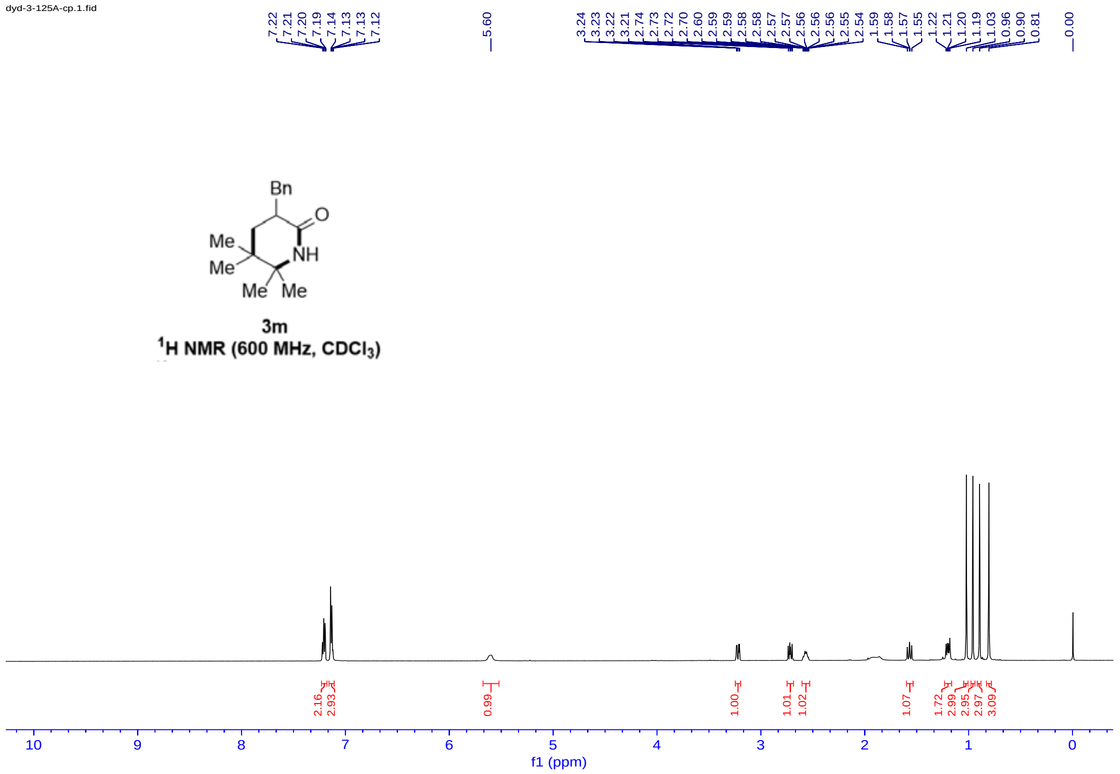

Supplementary Figure 75 ^{1}H NMR (600 MHz, 298K, $CDCl_3$) of **3m**

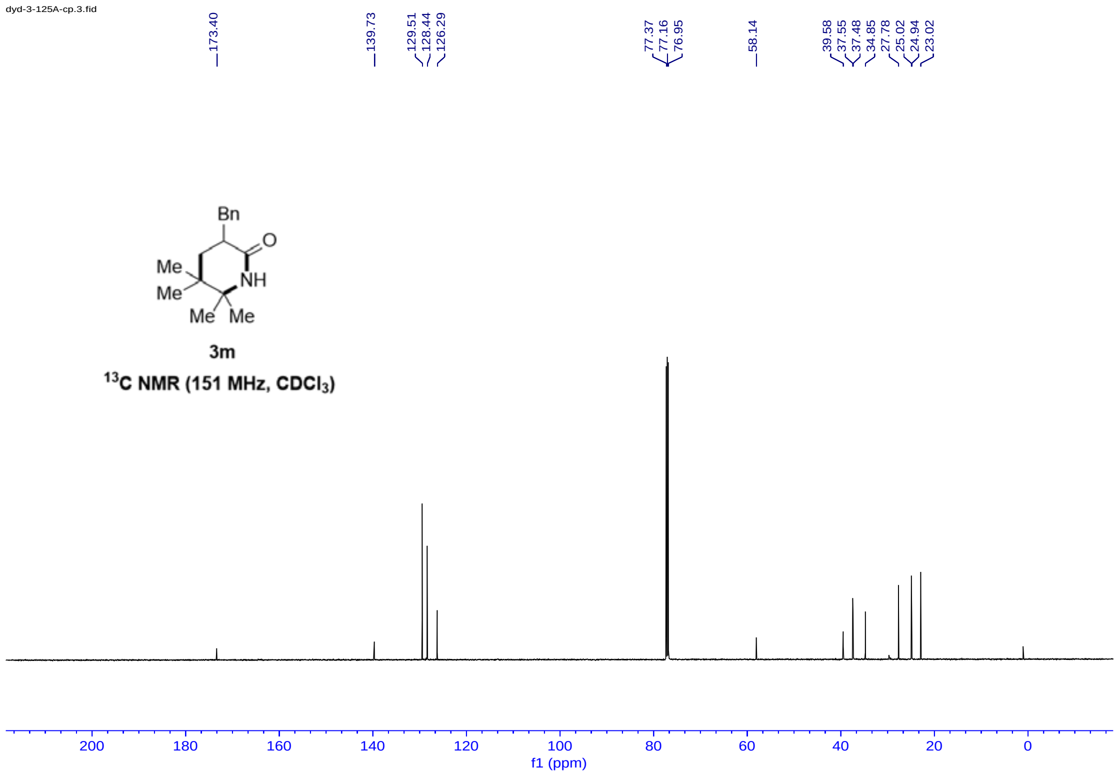

Supplementary Figure 76 ^{13}C NMR (151 MHz, 298K, $CDCl_3$) of **3m**

methyl 2-(5,5,6,6-tetramethyl-2-oxopiperidin-3-yl)acetate

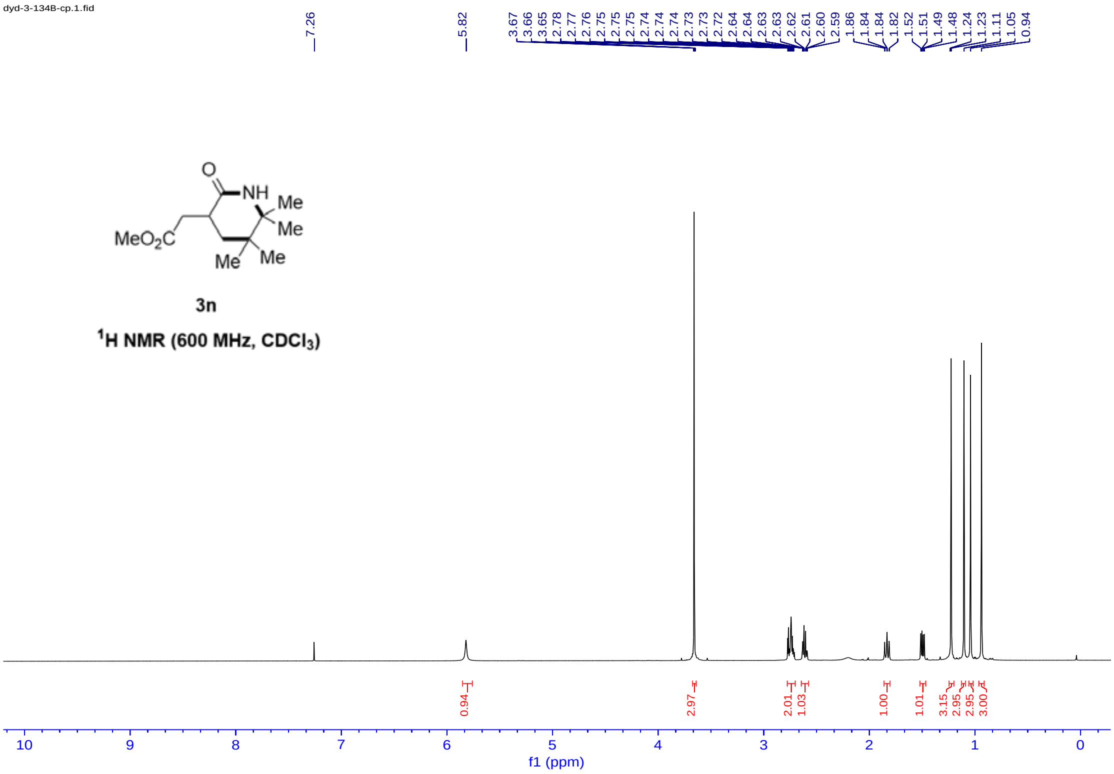

Supplementary Figure 77 ^{1}H NMR (600 MHz, 298K, $CDCl_3$) of **3n**

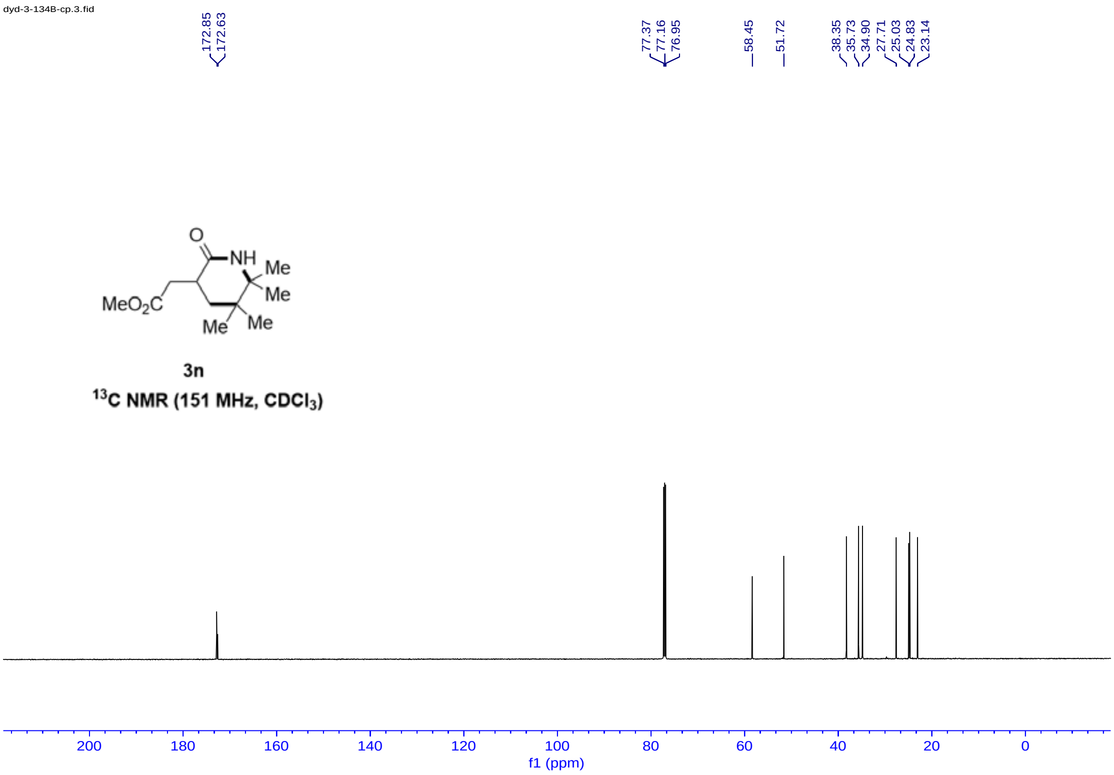

Supplementary Figure 78 ^{13}C NMR (151 MHz, 298K, $CDCl_3$) of **3n**

3-allyl-5,5,6,6-tetramethylpiperidin-2-one

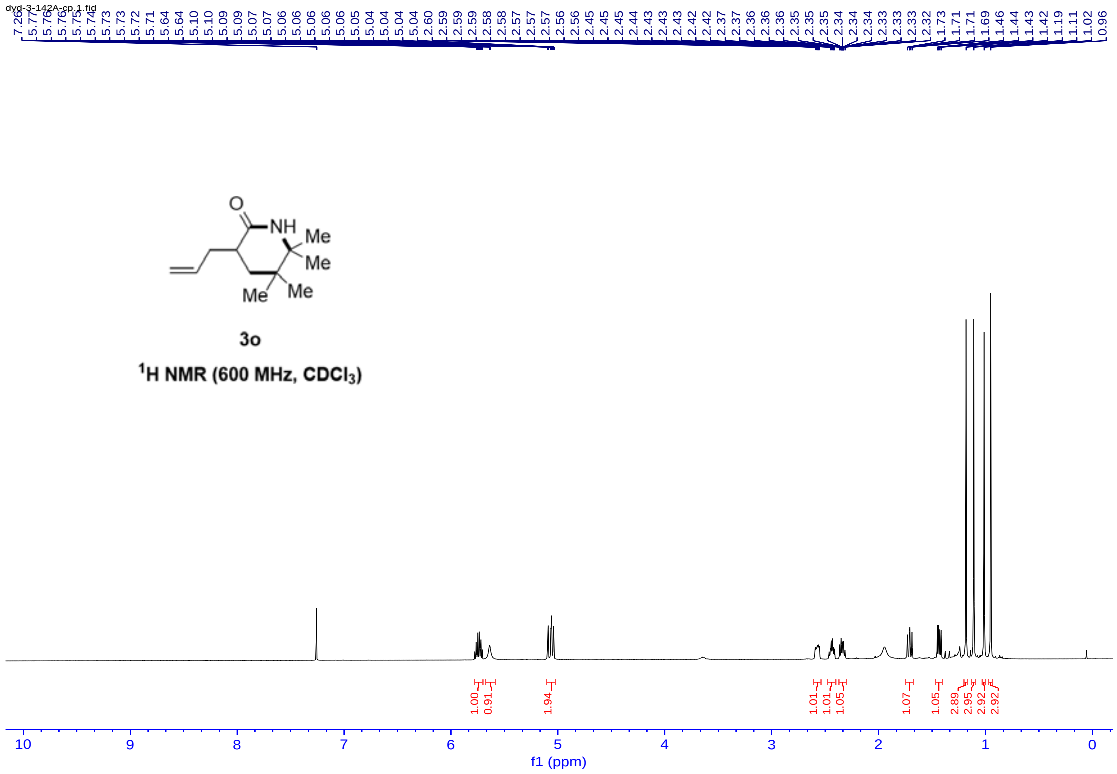

Supplementary Figure 79 ^{1}H NMR (600 MHz, 298K, $CDCl_3$) of **3o**

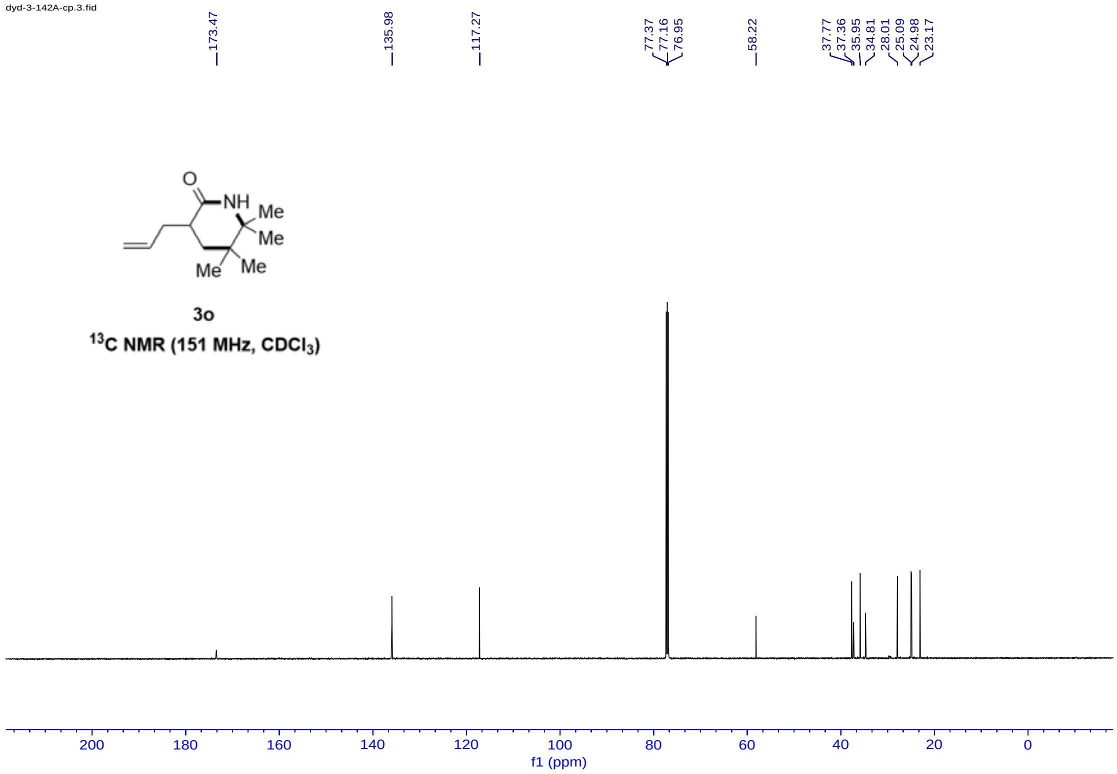

Supplementary Figure 80 ^{13}C NMR (151 MHz, 298K, $CDCl_3$) of **3o**

3-cinnamyl-5,5,6,6-tetramethylpiperidin-2-one

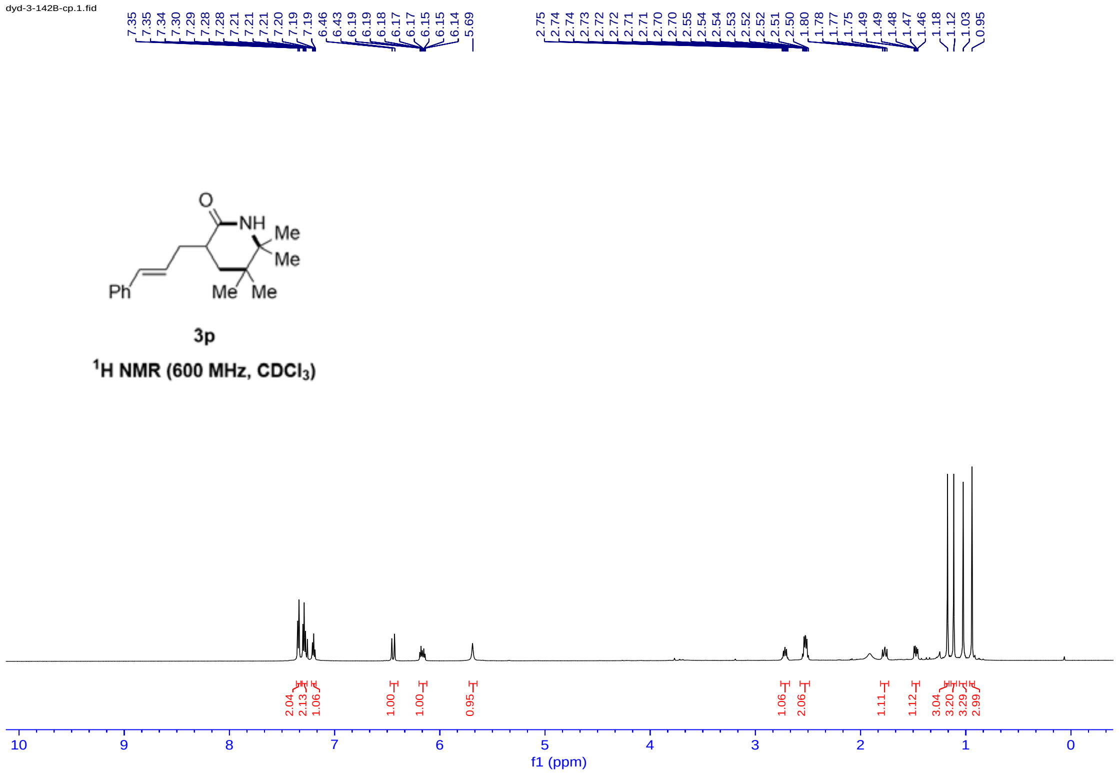

Supplementary Figure 81 ^{1}H NMR (600 MHz, 298K, $CDCl_3$) of **3p**

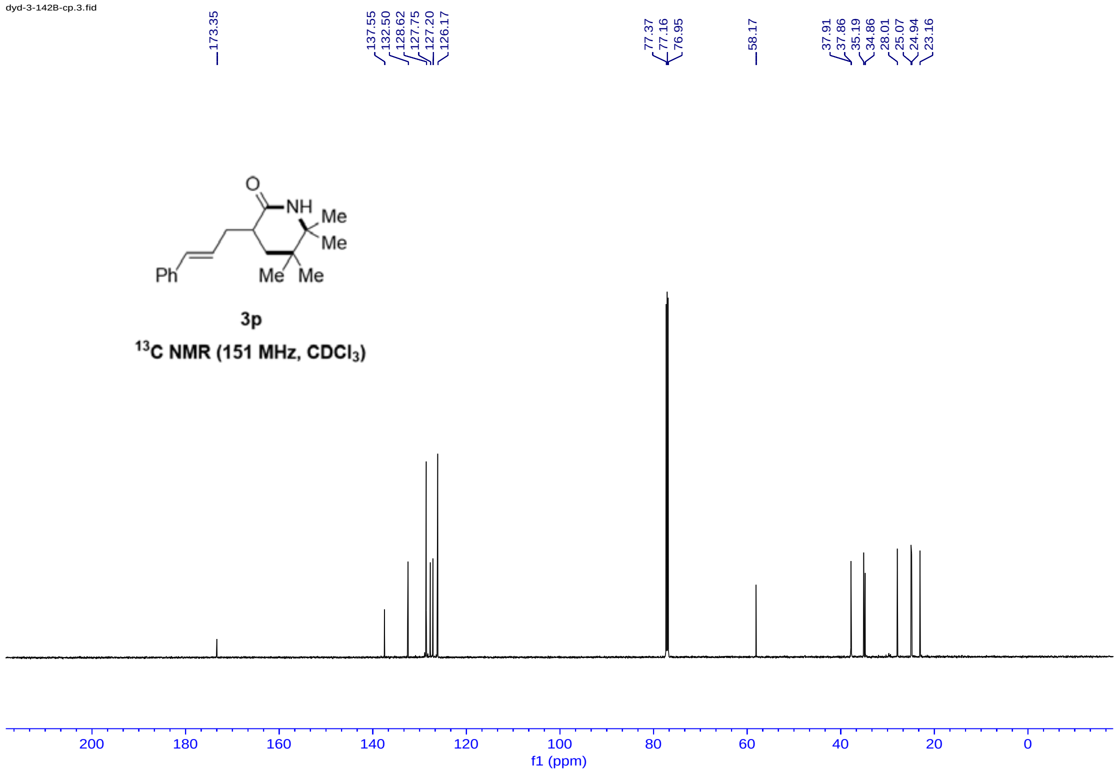

Supplementary Figure 82 ^{13}C NMR (151 MHz, 298K, $CDCl_3$) of **3p**

5,5,6,6-tetramethyl-3-(3-phenylprop-2-yn-1-yl)piperidin-2-one

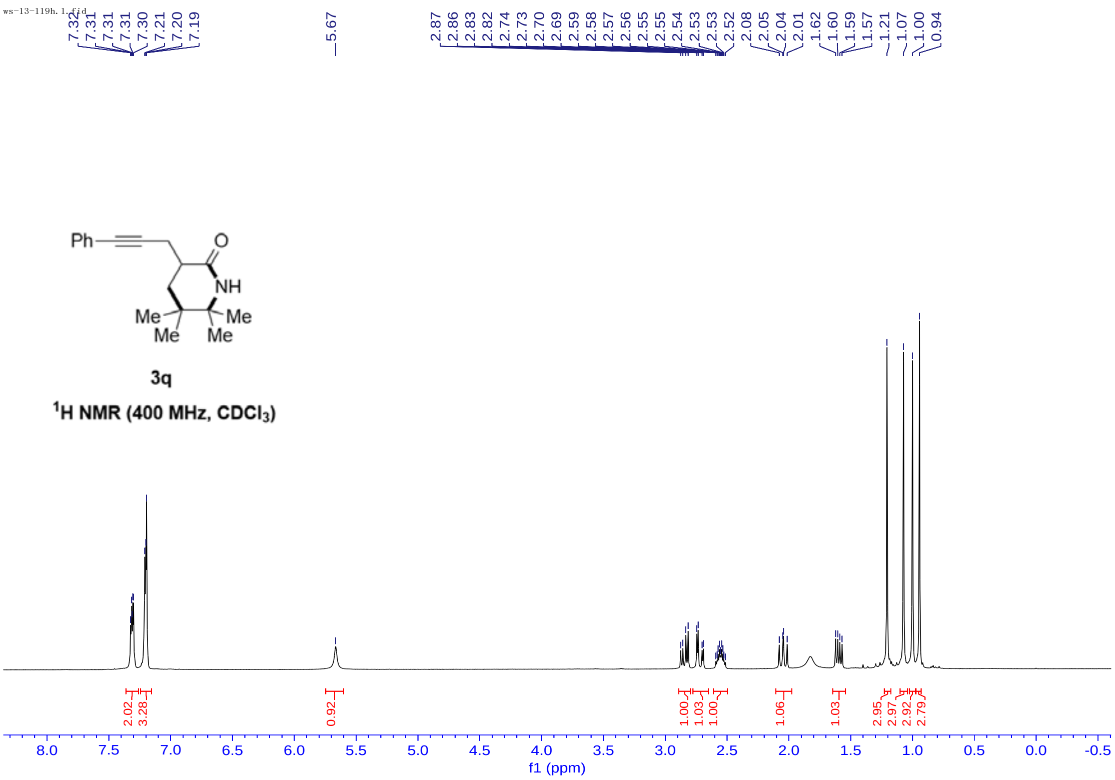

Supplementary Figure 83 ^{1}H NMR (400 MHz, 298K, $CDCl_3$) of **3q**

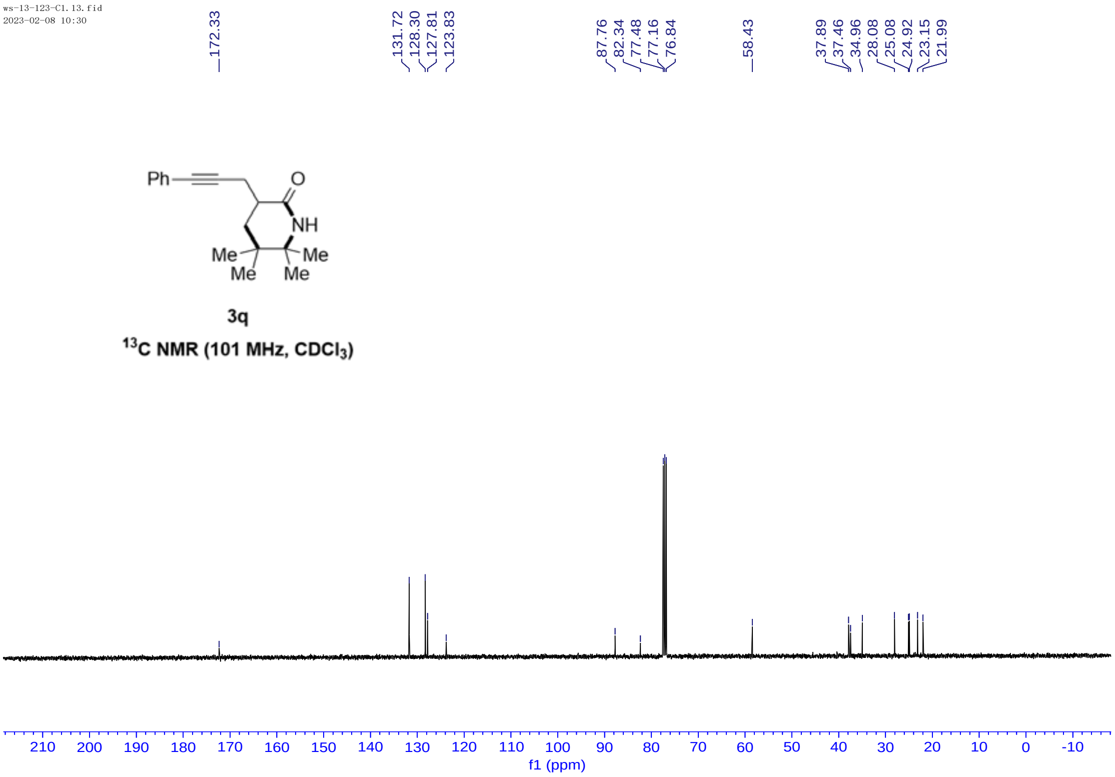

Supplementary Figure 84 ^{13}C NMR (101 MHz, 298K, $CDCl_3$) of **3q**

5,5,6,6-tetramethyl-3-(prop-2-yn-1-yl)piperidin-2-one

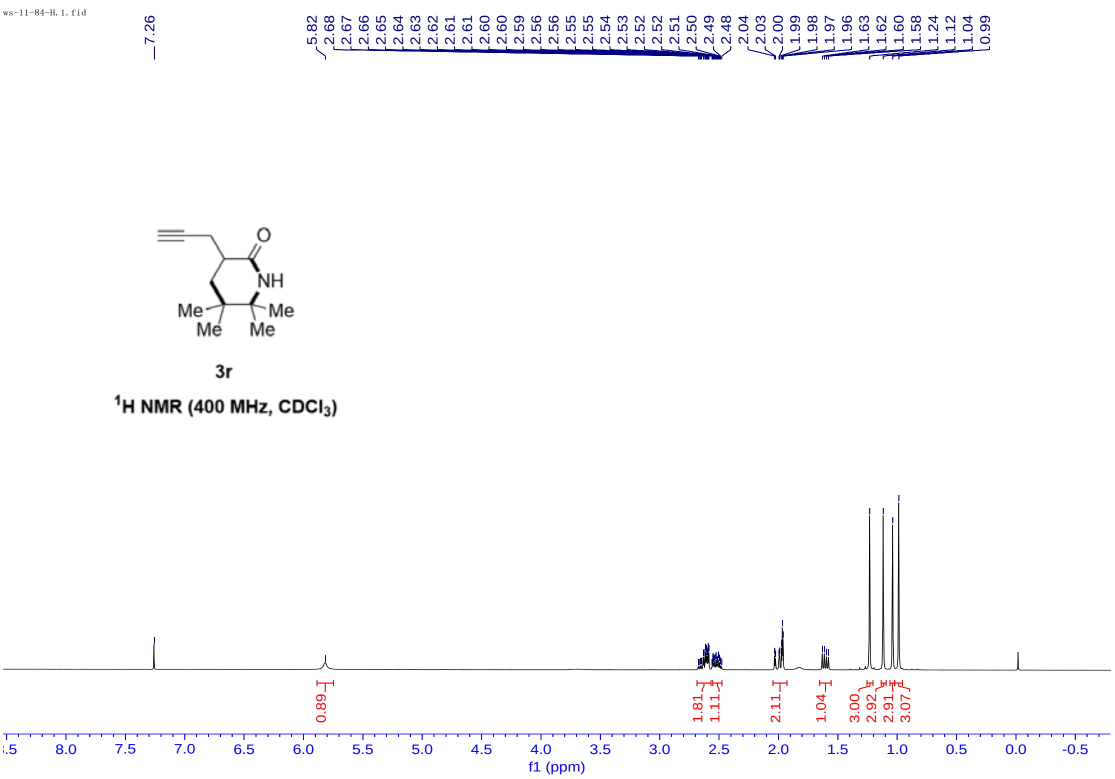

Supplementary Figure 85 ^{1}H NMR (400 MHz, 298K, $CDCl_3$) of **3r**

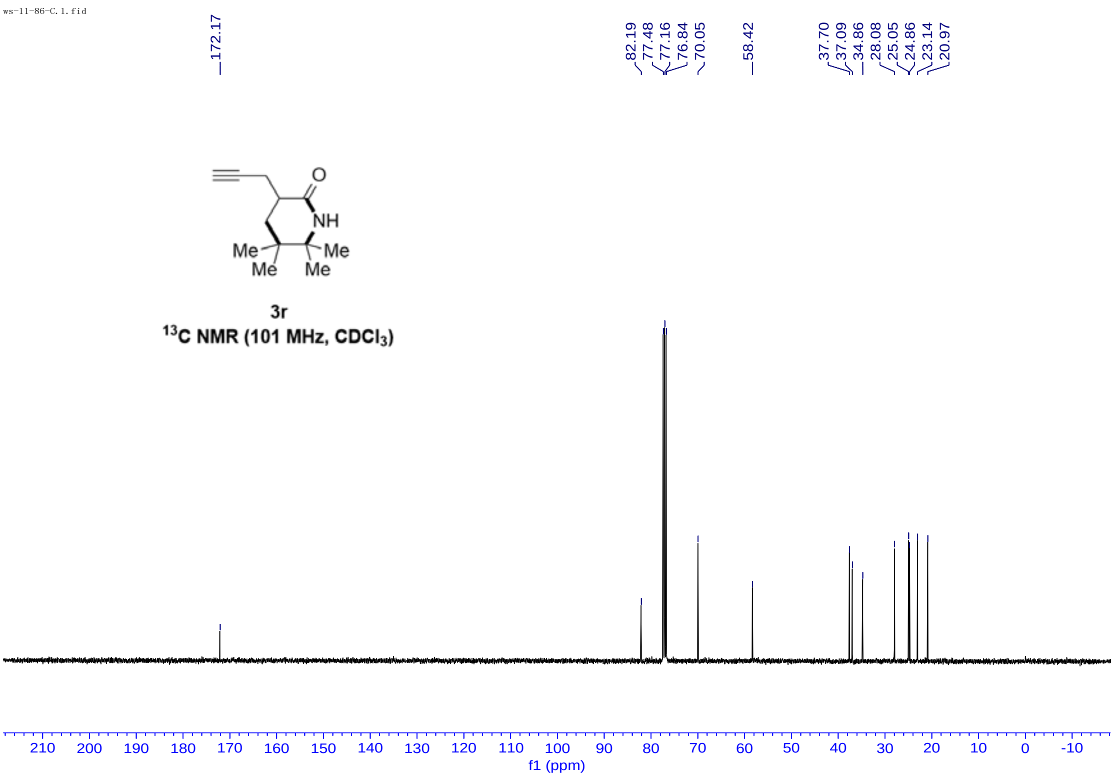

Supplementary Figure 86 ^{13}C NMR (101 MHz, 298K, $CDCl_3$) of **3r**

3-(4-chlorobutyl)-5,5,6,6-tetramethylpiperidin-2-one

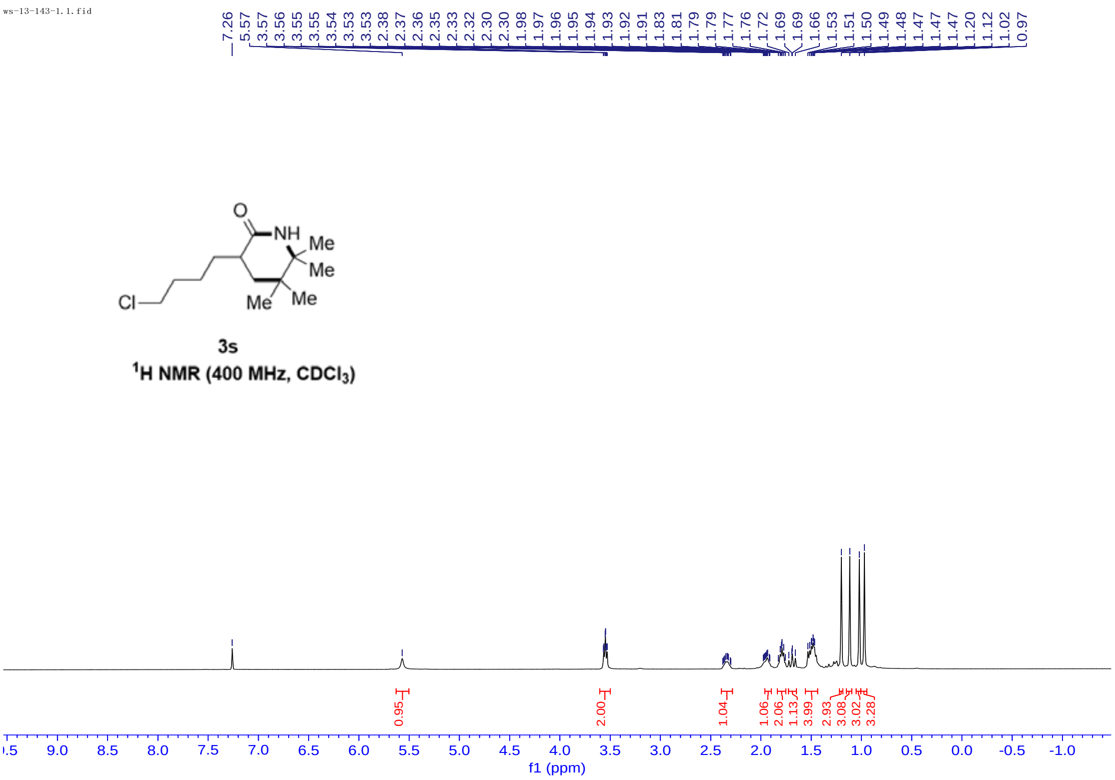

Supplementary Figure 87 ^{1}H NMR (400 MHz, 298K, $CDCl_3$) of **3s**

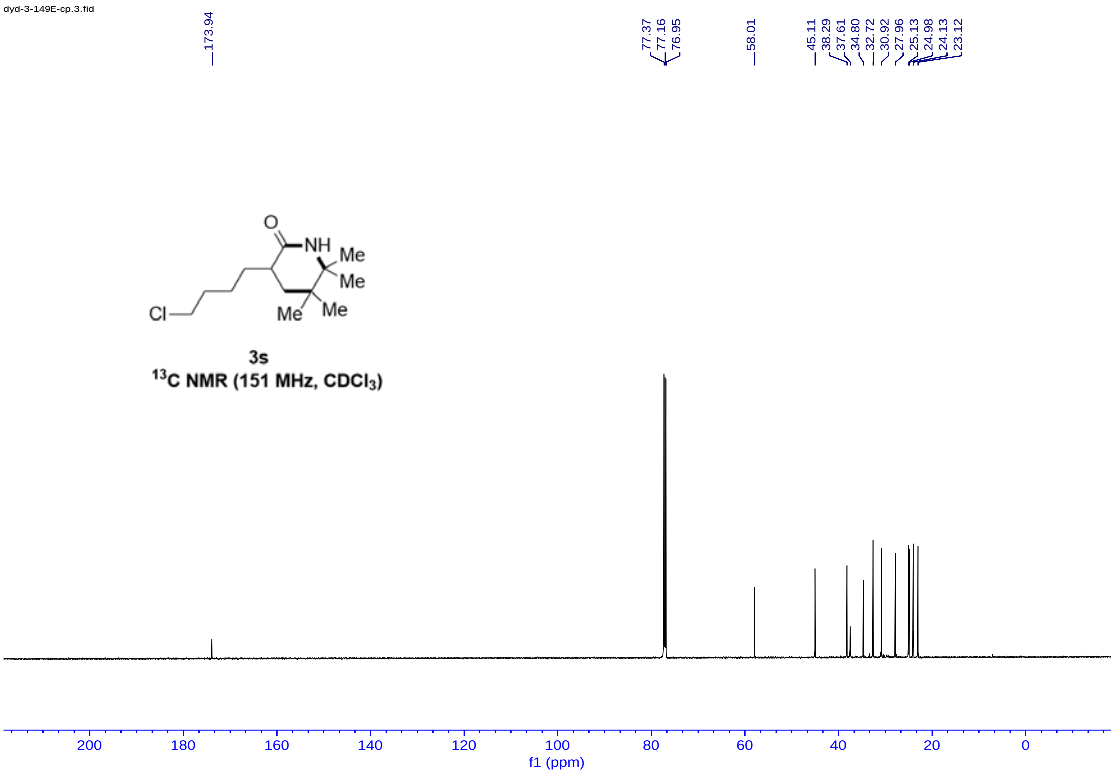

Supplementary Figure 88 ^{13}C NMR (151 MHz, 298K, $CDCl_3$) of **3s**

3-(4-bromobutyl)-5,5,6,6-tetramethylpiperidin-2-one

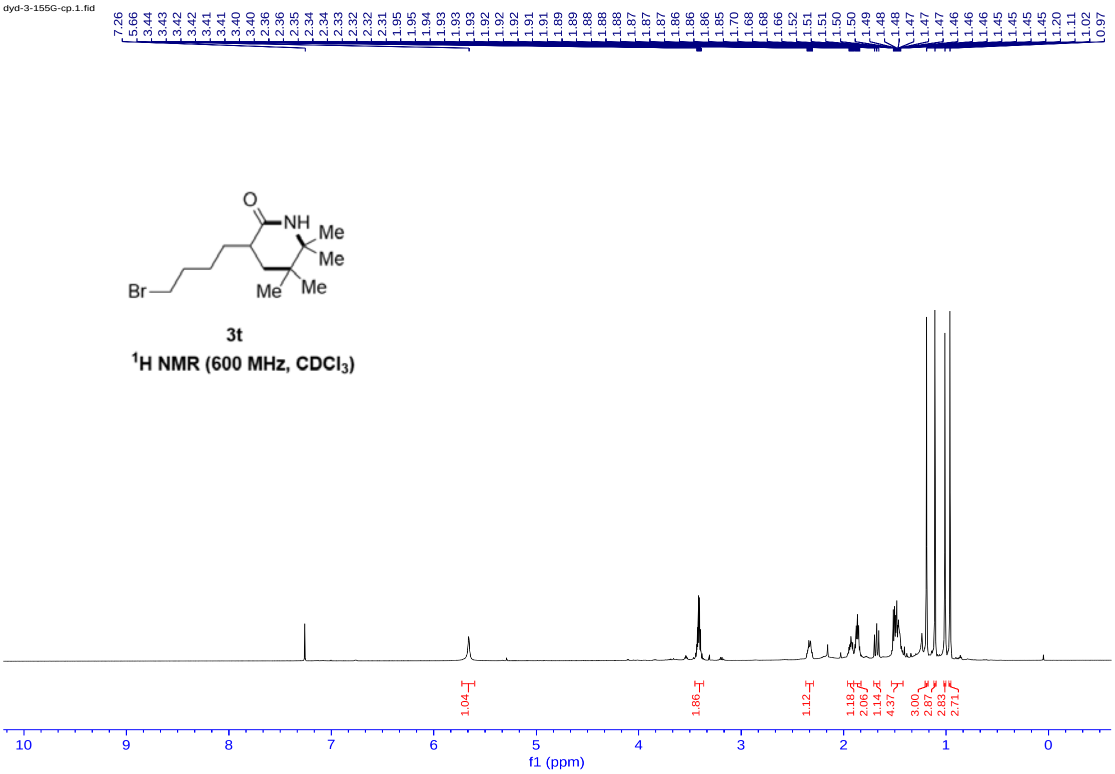

Supplementary Figure 89 ^{1}H NMR (600 MHz, 298K, $CDCl_3$) of **3t**

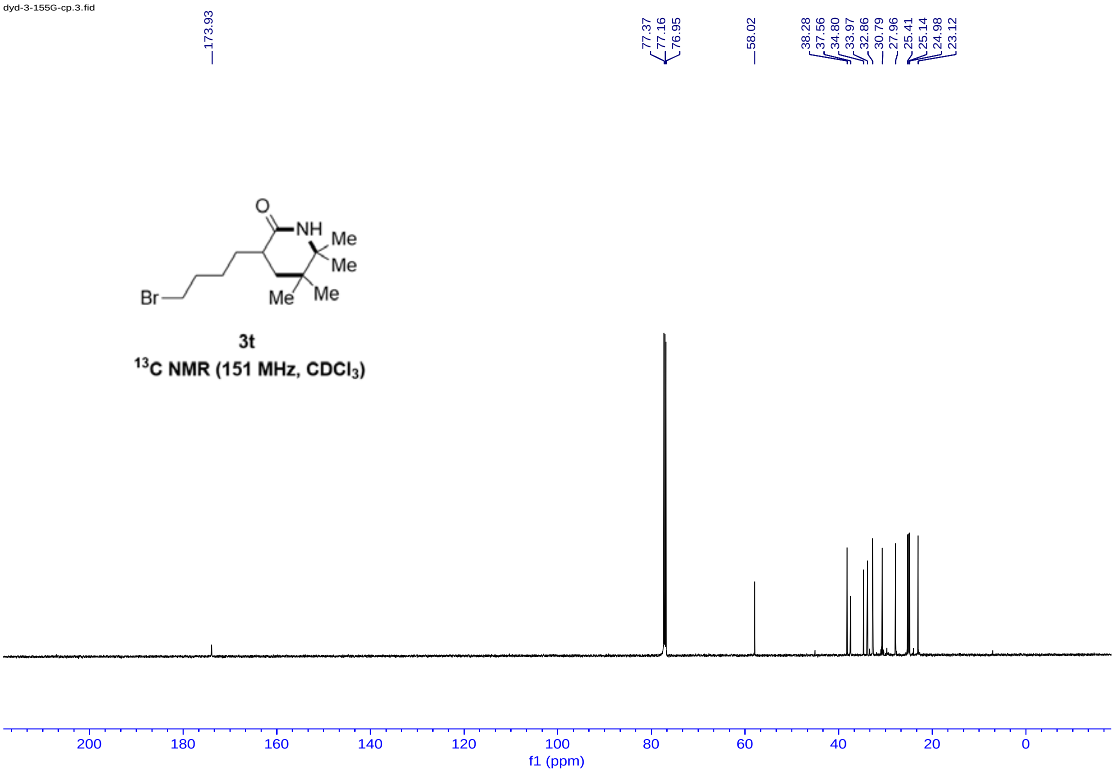

Supplementary Figure 90 ^{13}C NMR (151 MHz, 298K, $CDCl_3$) of **3t**

3-((benzyloxy)methyl)-5,5,6,6-tetramethylpiperidin-2-one

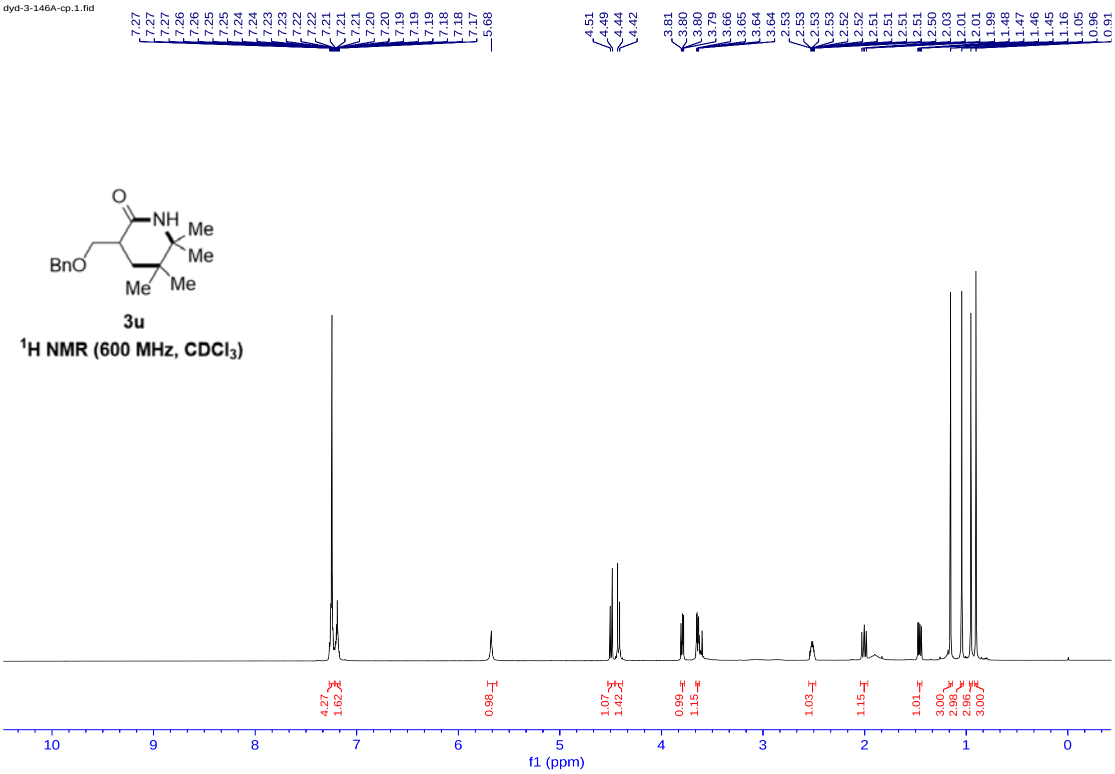

Supplementary Figure 91 ^{1}H NMR (600 MHz, 298K, $CDCl_3$) of **3u**

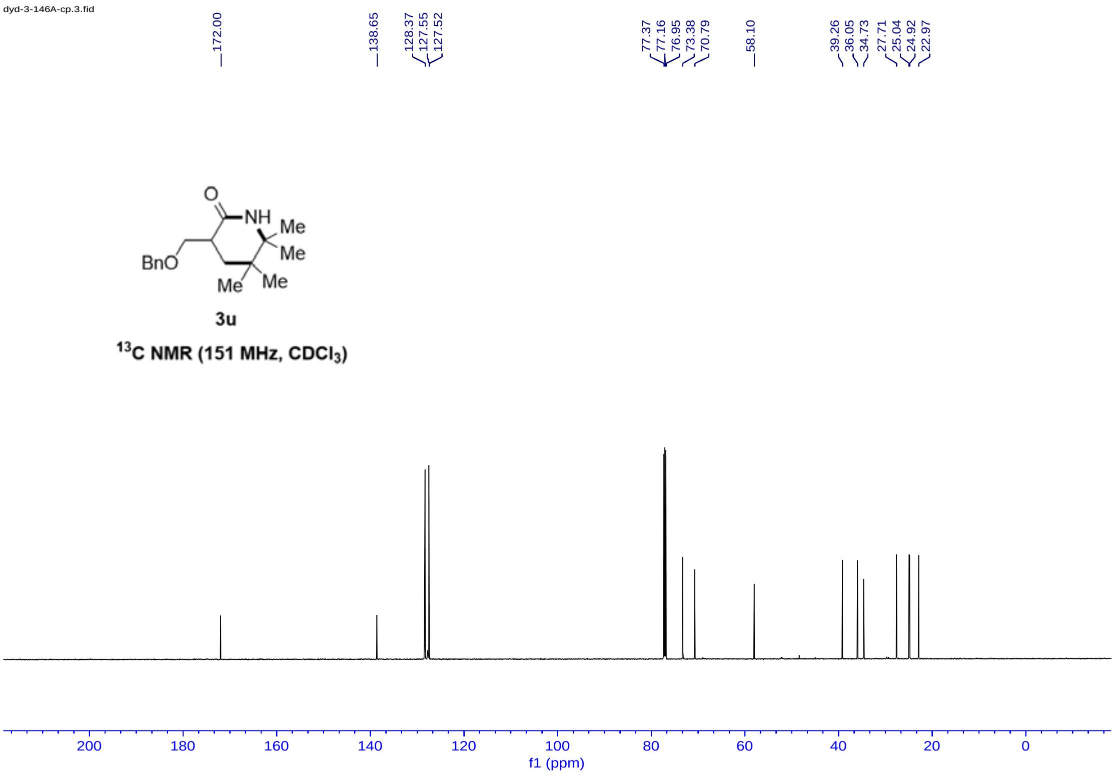

Supplementary Figure 92 ^{13}C NMR (151 MHz, 298K, $CDCl_3$) of **3u**

5,5,6,6-tetramethyl-3-((tetrahydrofuran-2-yl)methyl)piperidin-2-one

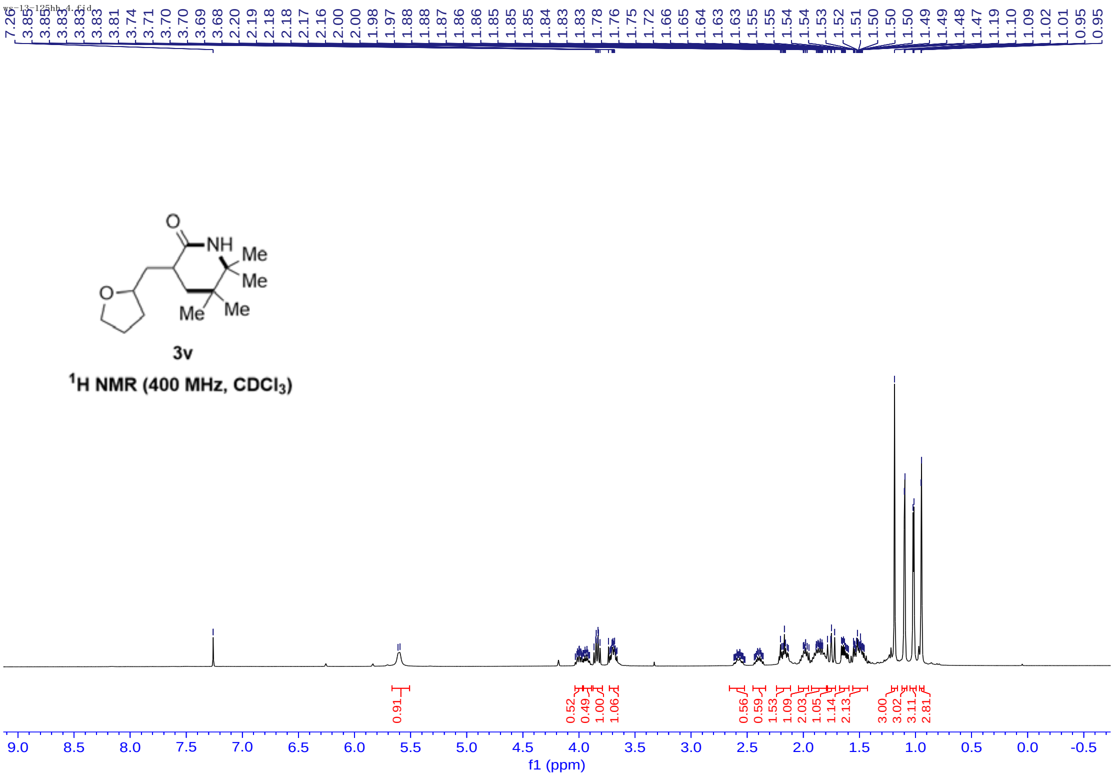

Supplementary Figure 93 ^{1}H NMR (400 MHz, 298K, $CDCl_3$) of **3v**

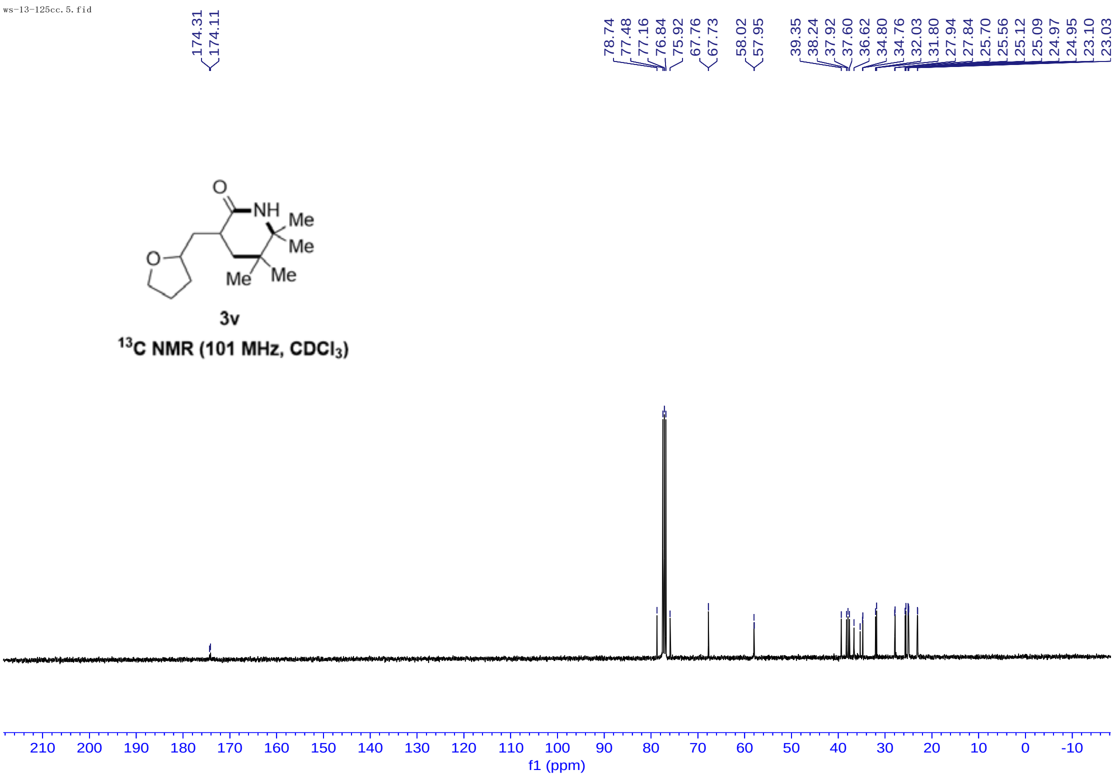

Supplementary Figure 94 ^{13}C NMR (101 MHz, 298K, $CDCl_3$) of **3v**

3-di(*tert*-butoxycarbonyl)amino -5,5,6,6-tetramethylpiperidin-2-one

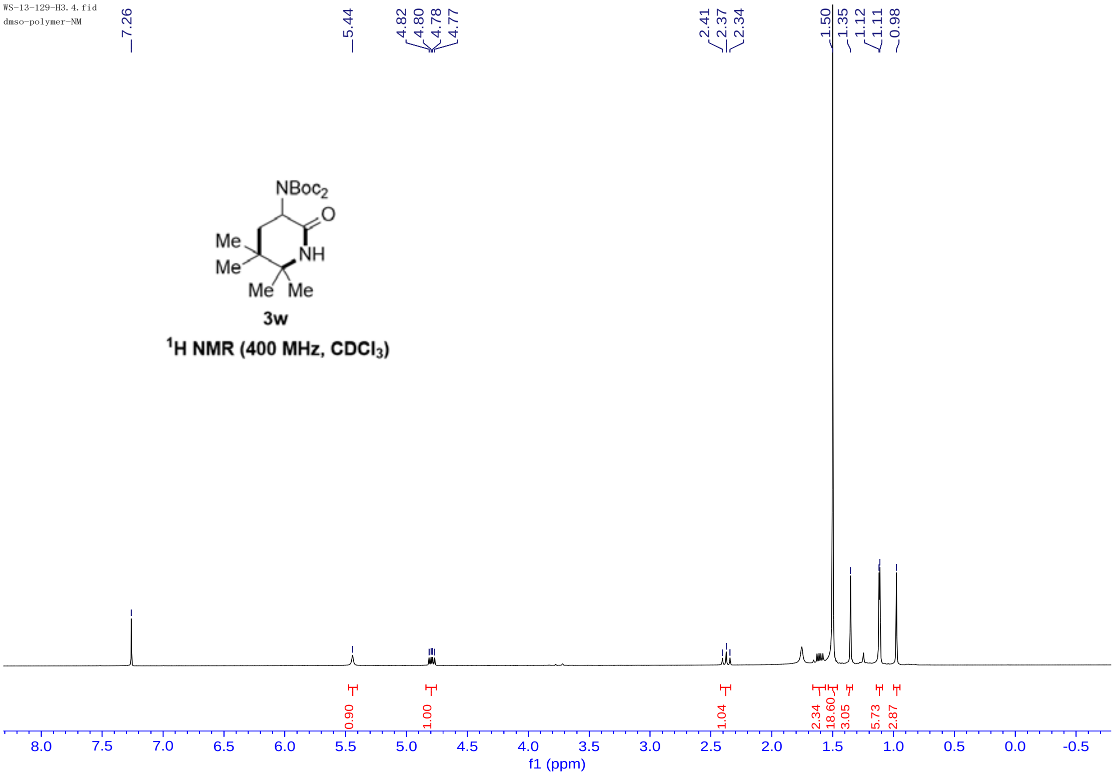

Supplementary Figure 95 ^{1}H NMR (400 MHz, 298K, $CDCl_3$) of **3w**

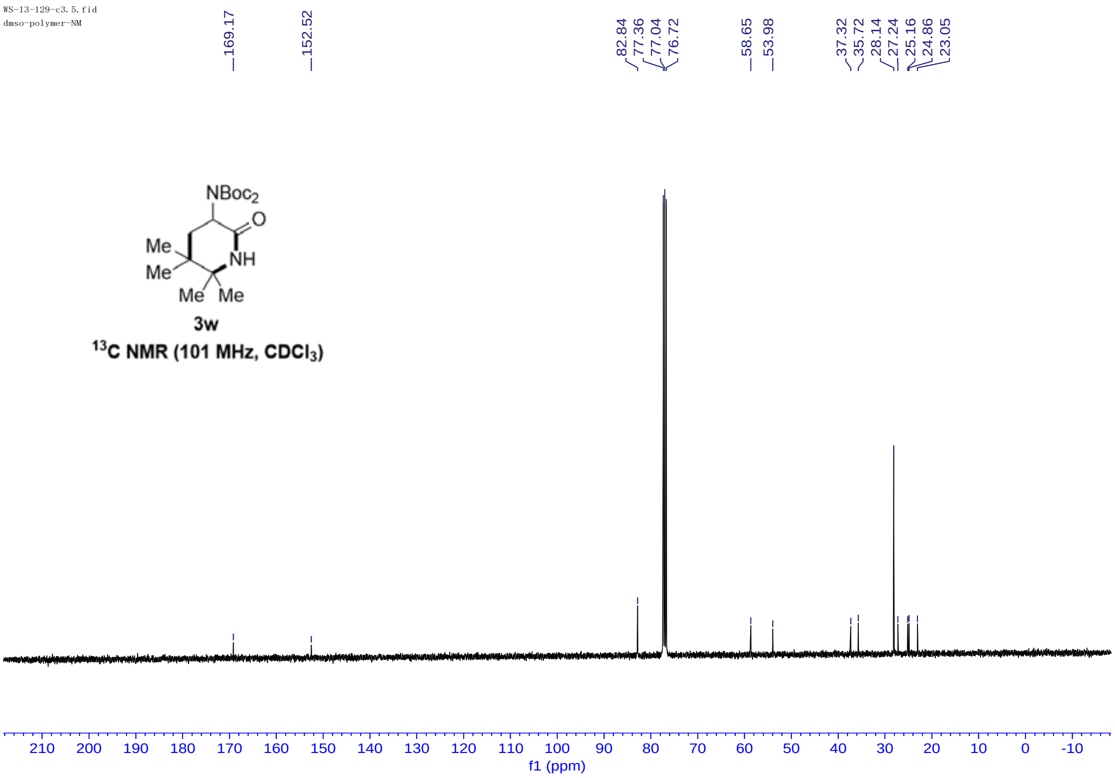

Supplementary Figure 96 ^{13}C NMR (101 MHz, 298K, $CDCl_3$) of **3w**

N-(5,5,6,6-tetramethyl-2-oxopiperidin-3-yl)benzamide

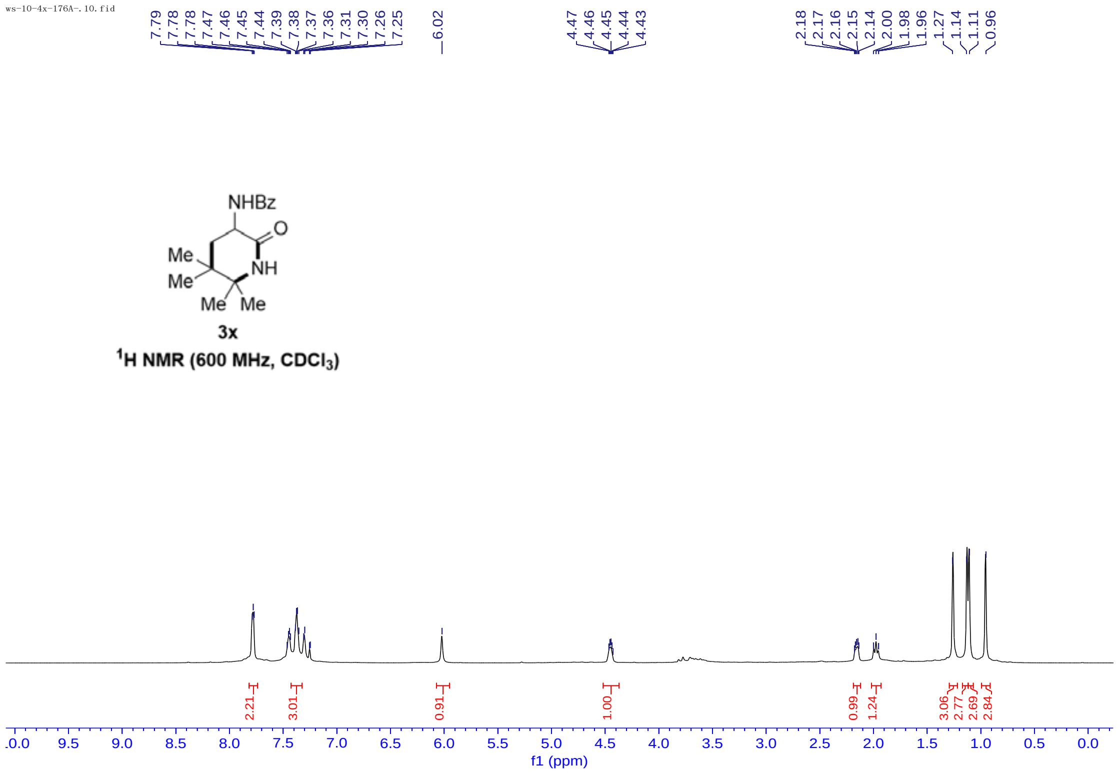

Supplementary Figure 97 ^{1}H NMR (600 MHz, 298K, $CDCl_3$) of **3x**

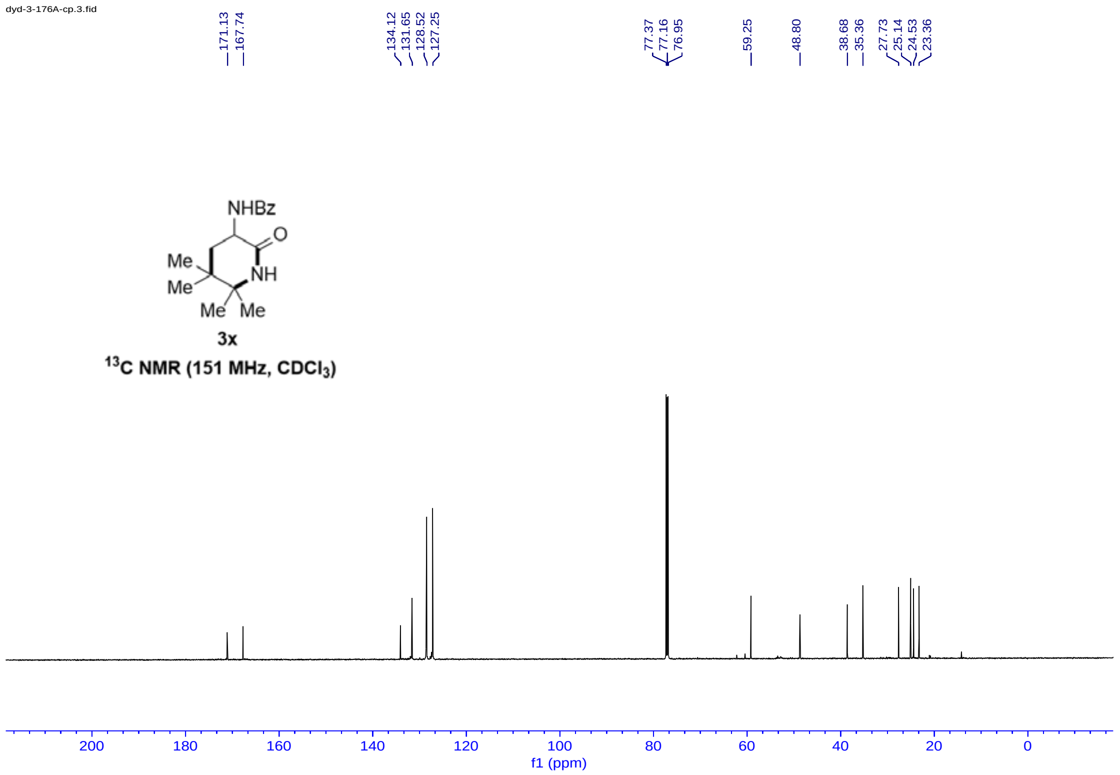

Supplementary Figure 98 ^{13}C NMR (151 MHz, 298K, $CDCl_3$) of **3x**

3-(2-hydroxyethyl)-5,5,6,6-tetramethylpiperidin-2-one

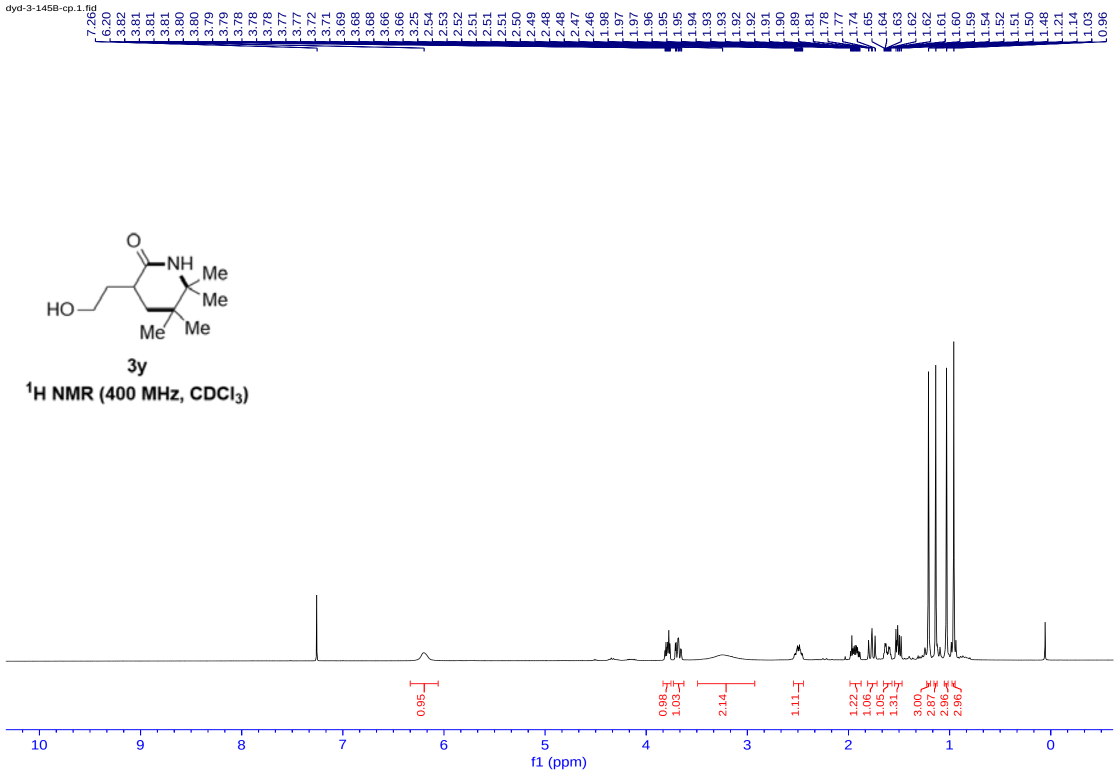

Supplementary Figure 99 ^{1}H NMR (400 MHz, 298K, $CDCl_3$) of **3y**

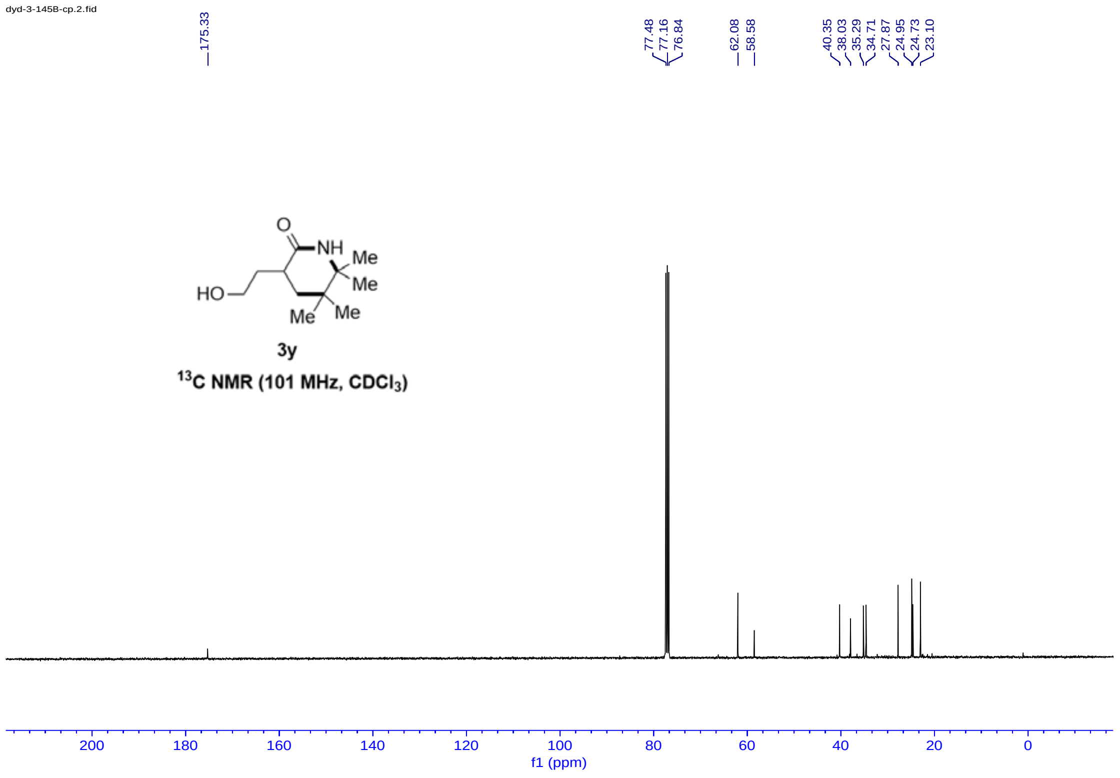

Supplementary Figure 100 ^{13}C NMR (101 MHz, 298K, $CDCl_3$) of **3y**

5,5,6,6-tetramethyl-3-((phenylamino)methyl)piperidin-2-one

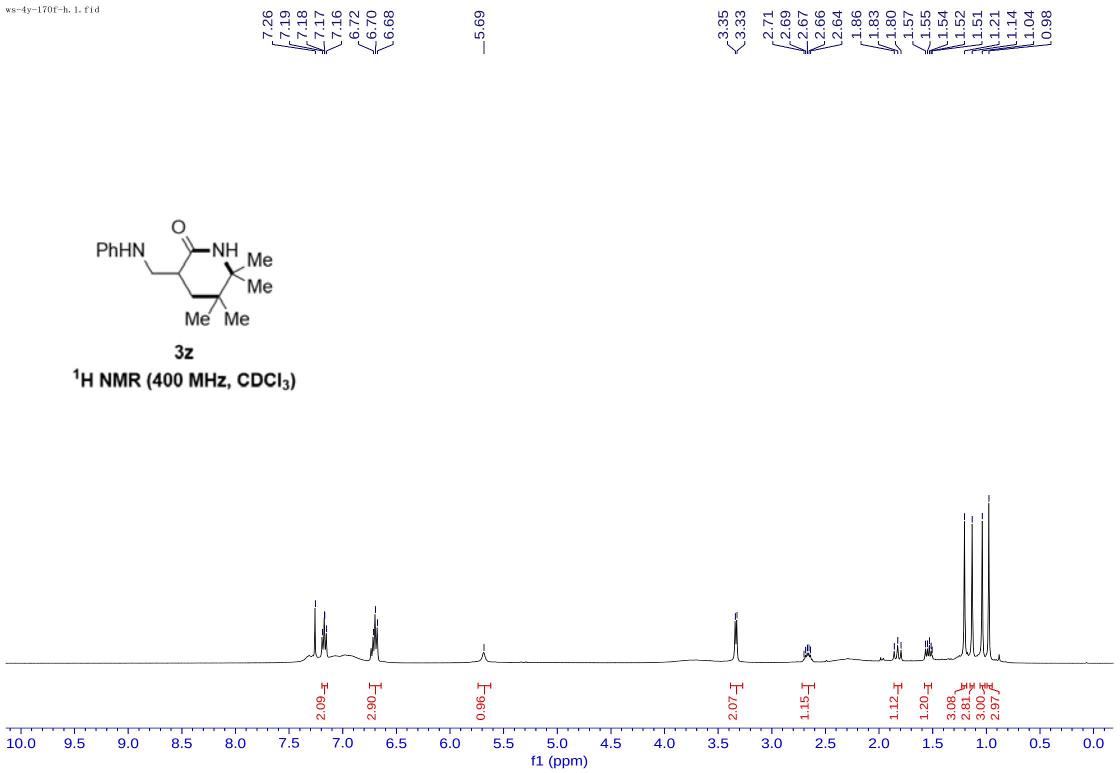

Supplementary Figure 101 ^{1}H NMR (400 MHz, 298K, $CDCl_3$) of **3z**

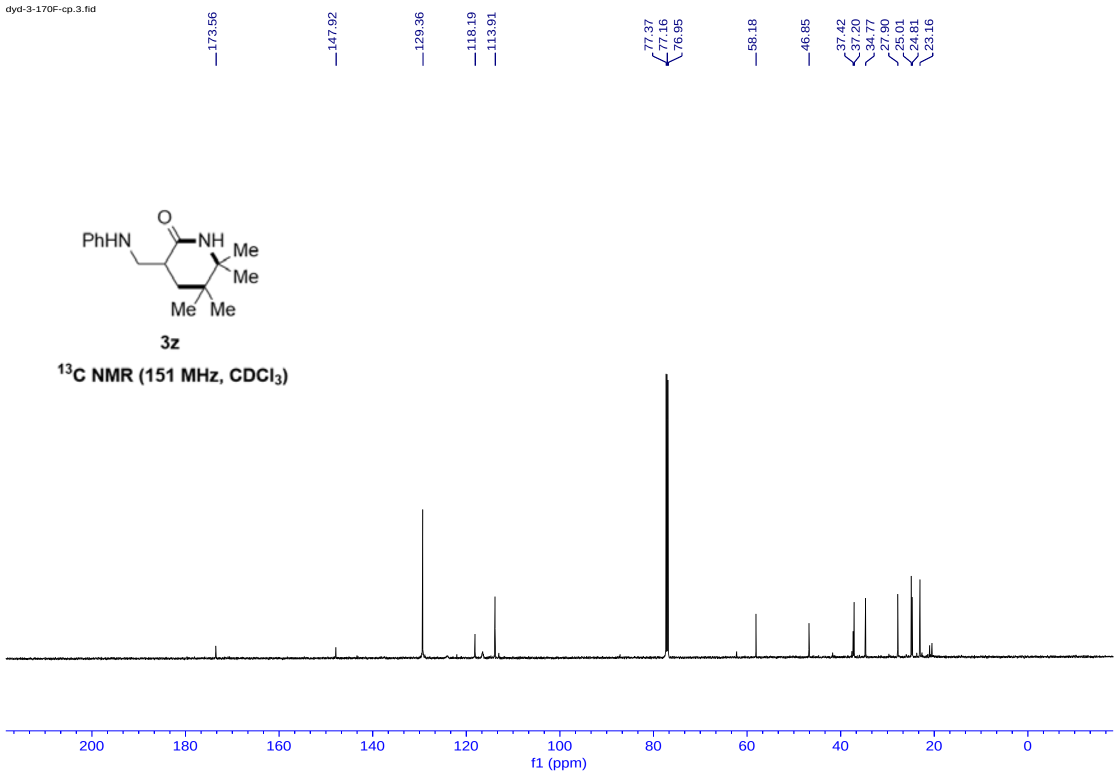

Supplementary Figure 102 ^{13}C NMR (151 MHz, 298K, $CDCl_3$) of **3z**

2,2,3,3-tetramethyl-6-oxo-*N*-phenylpiperidine-4-carboxamide

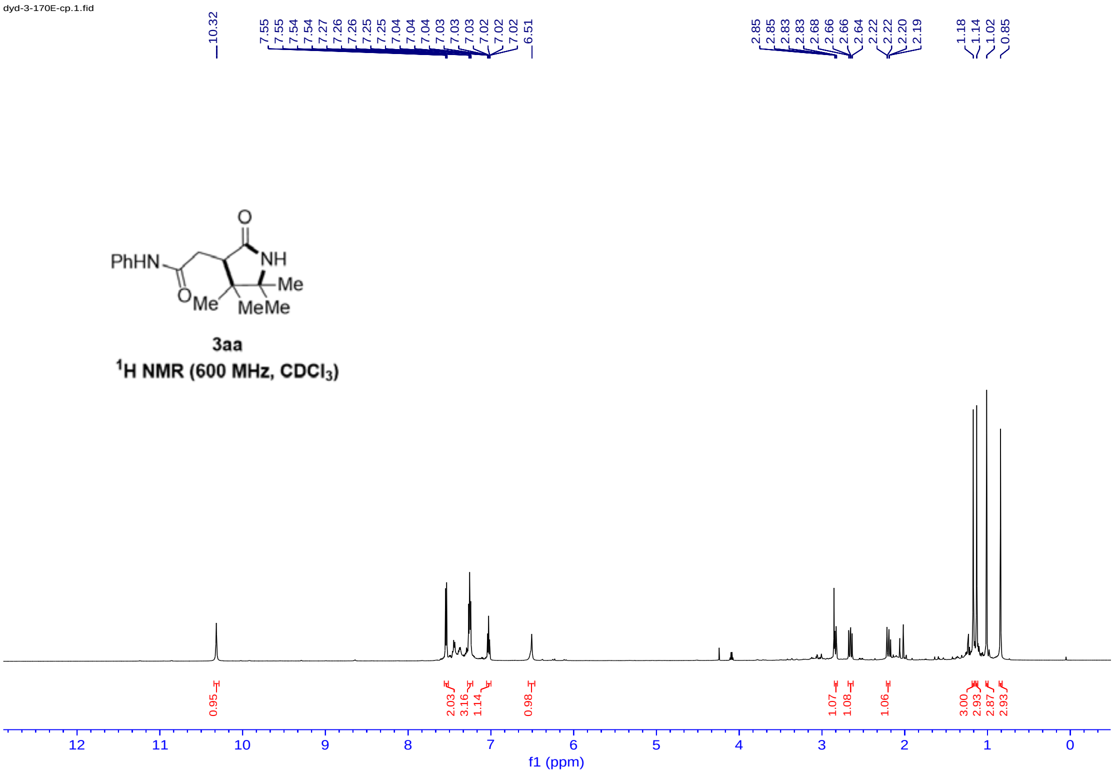

Supplementary Figure 103 ^{1}H NMR (600 MHz, 298K, $CDCl_3$) of **3aa**

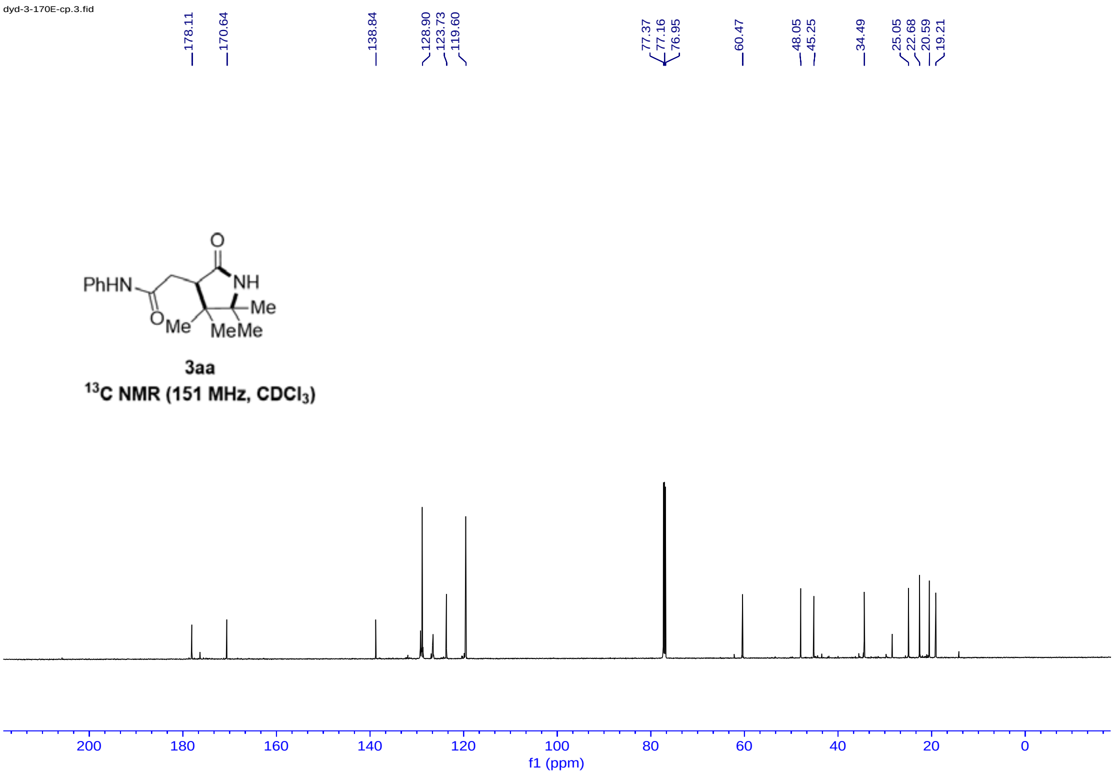

Supplementary Figure 104 ^{13}C NMR (151 MHz, 298K, $CDCl_3$) of **3aa**

cis-3,5-bis(4-fluorophenyl)-6,6-dimethylpiperidin-2-one

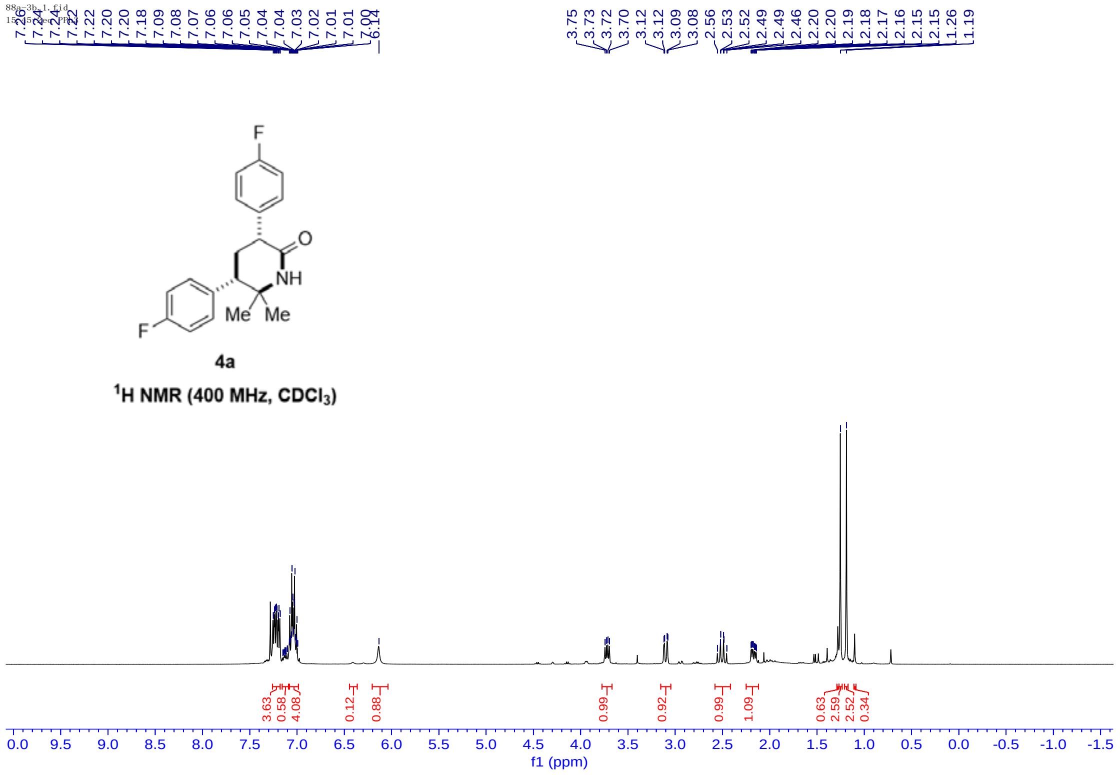

Supplementary Figure 105 ^{1}H NMR (400 MHz, 298K, $CDCl_3$) of **4a**

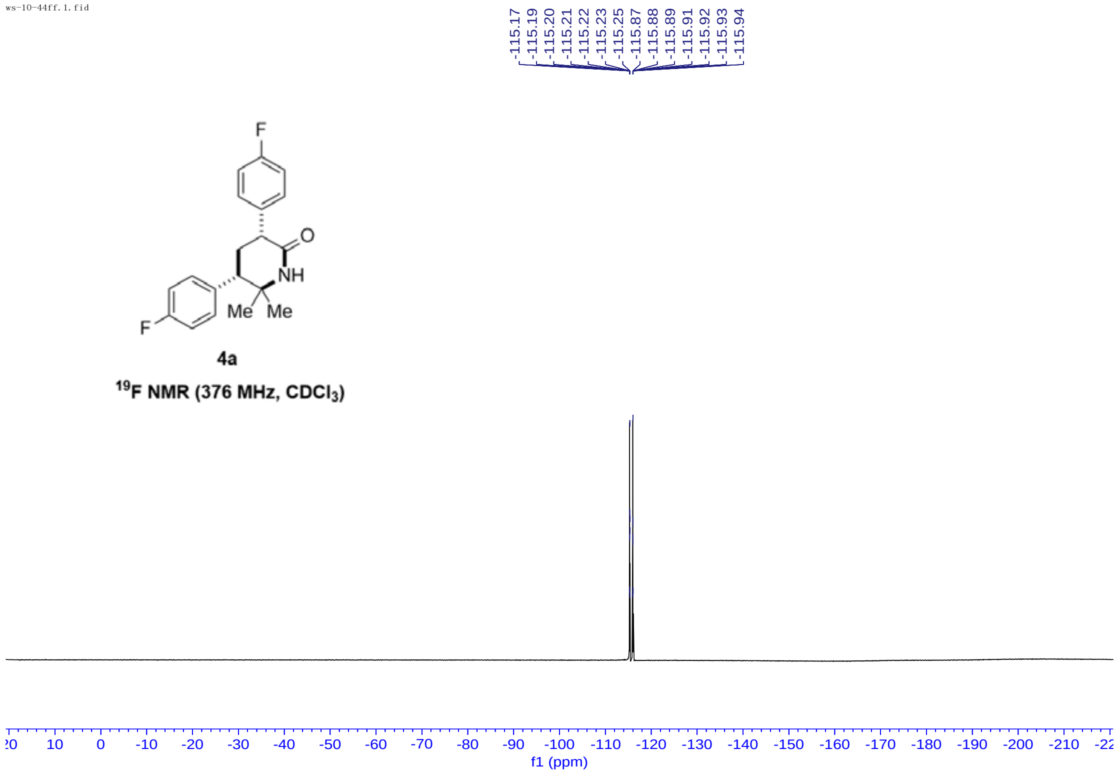

Supplementary Figure 106 ^{19}F NMR (376 MHz, 298K, $CDCl_3$) of **4a**

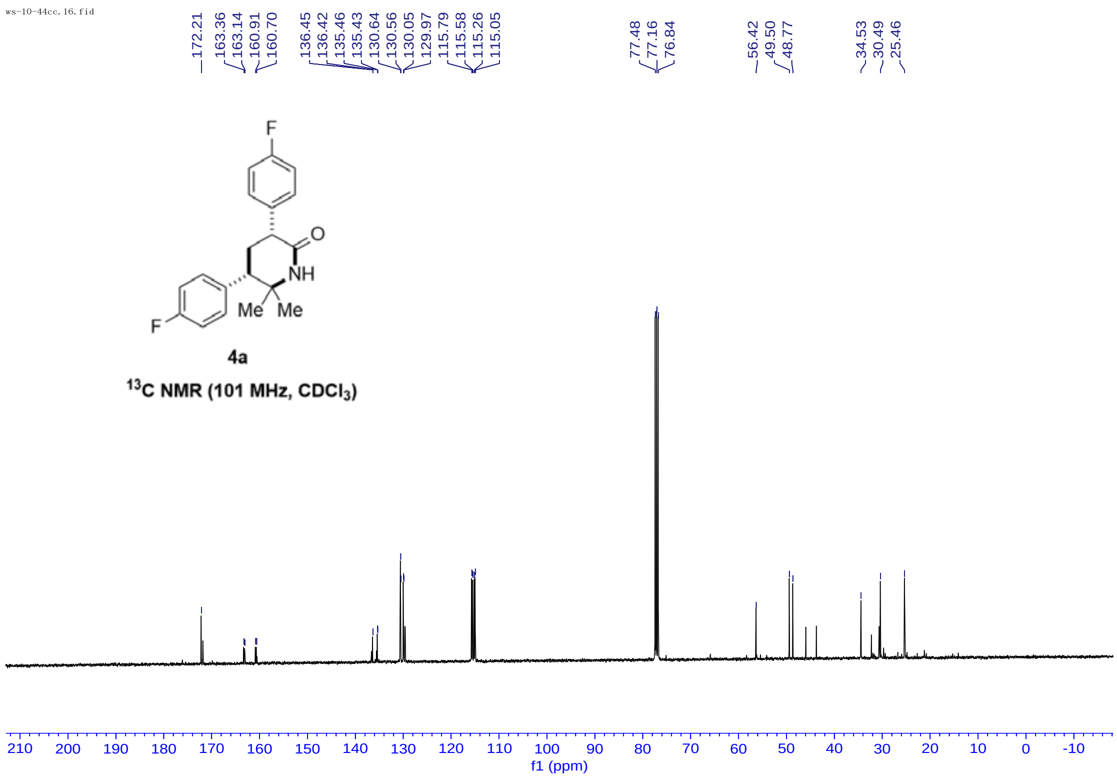

Supplementary Figure 107 ^{13}C NMR (101 MHz, 298K, $CDCl_3$) of **4a**

***cis*-5-(4-fluorophenyl)-6,6-dimethyl-3-(4-(trifluoromethyl)phenyl)piperidin-2-one**

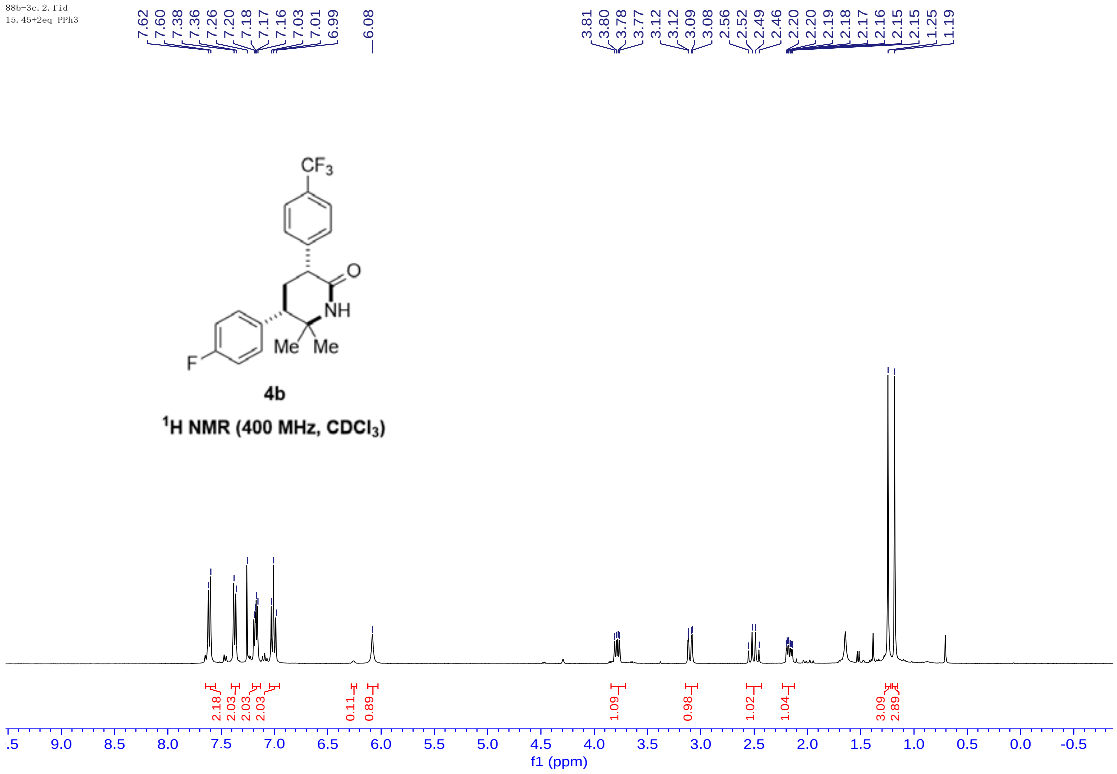

Supplementary Figure 108 ^{1}H NMR (400 MHz, 298K, $CDCl_3$) of **4b**

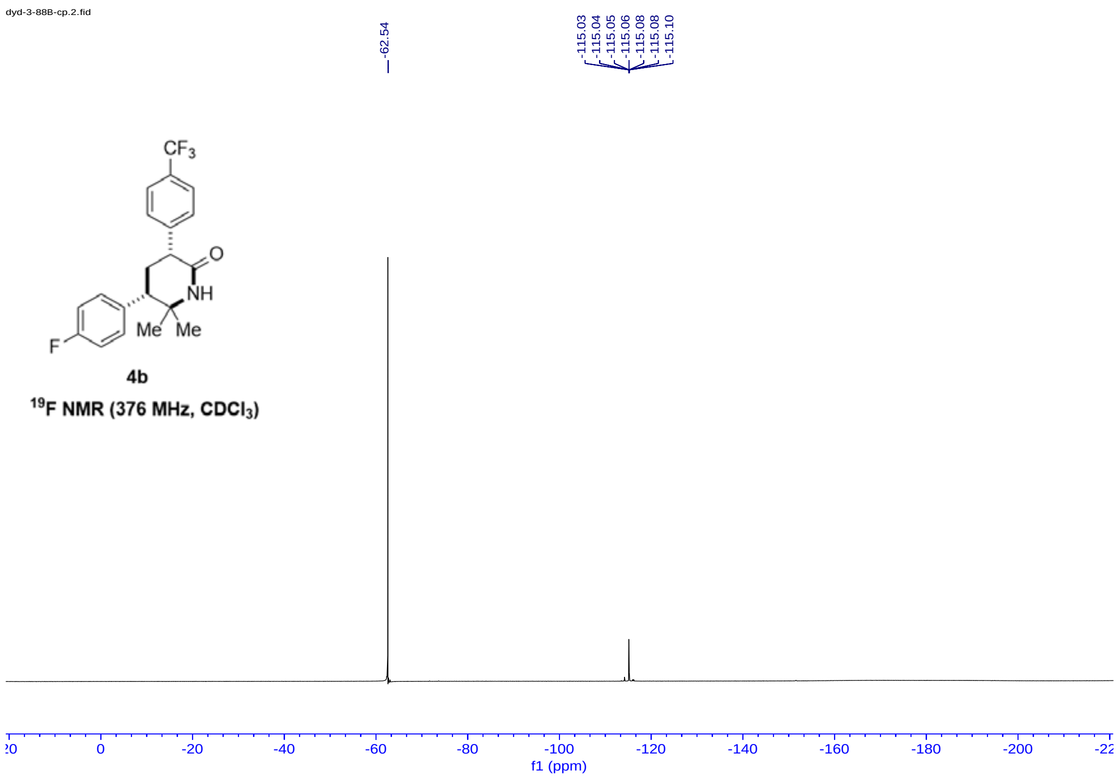

Supplementary Figure 109 ^{19}F NMR (376 MHz, 298K, $CDCl_3$) of **4b**

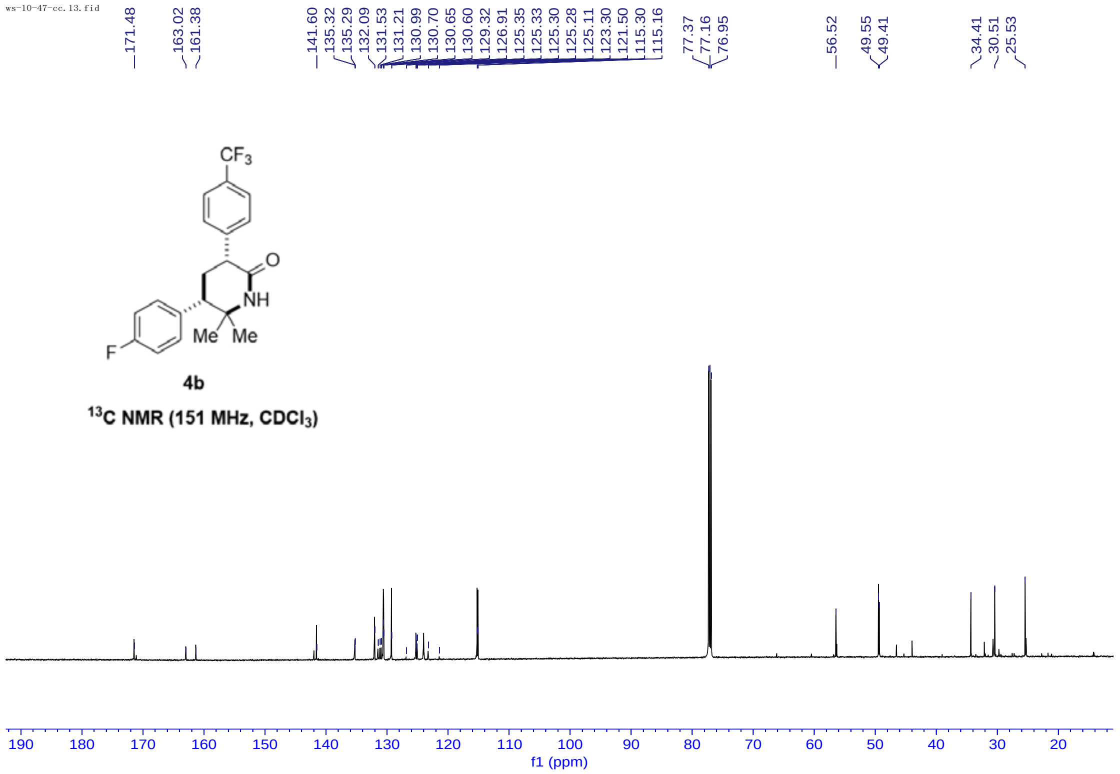

Supplementary Figure 110 ^{13}C NMR (151 MHz, 298K, $CDCl_3$) of **4b**

***cis*-5-(4-fluorophenyl)-3-(4-methoxyphenyl)-6,6-dimethylpiperidin-2-one**

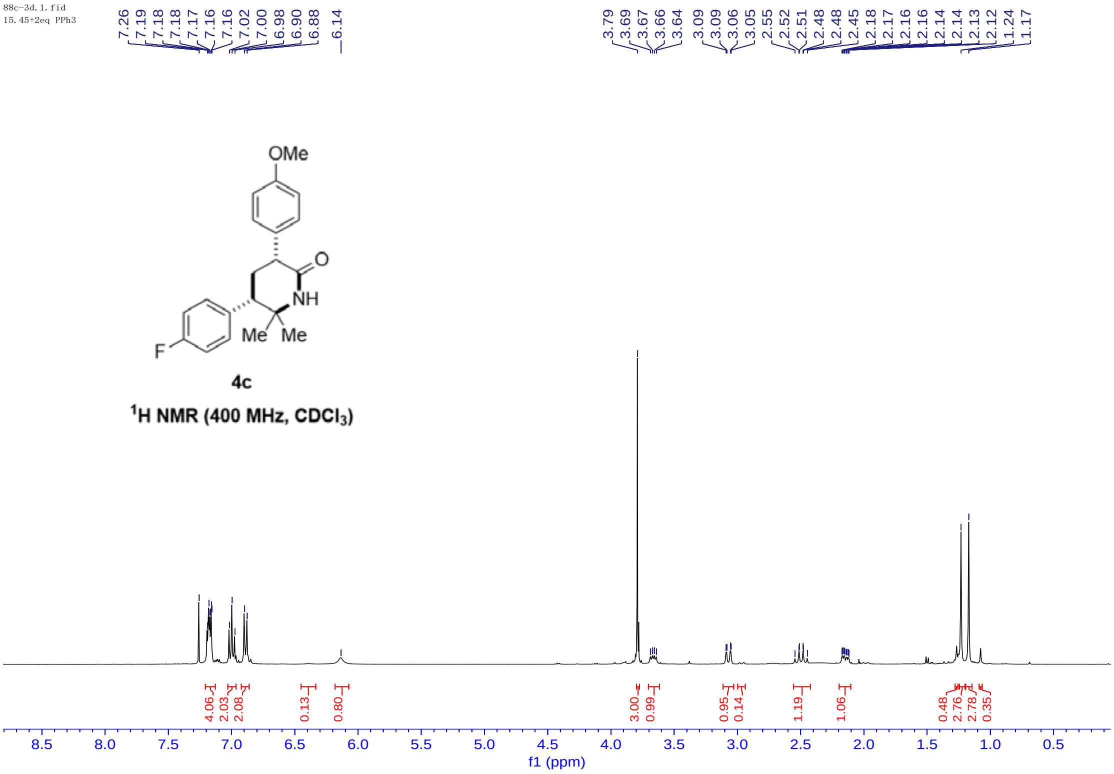

Supplementary Figure 111 ^{1}H NMR (400 MHz, 298K, $CDCl_3$) of **4c**

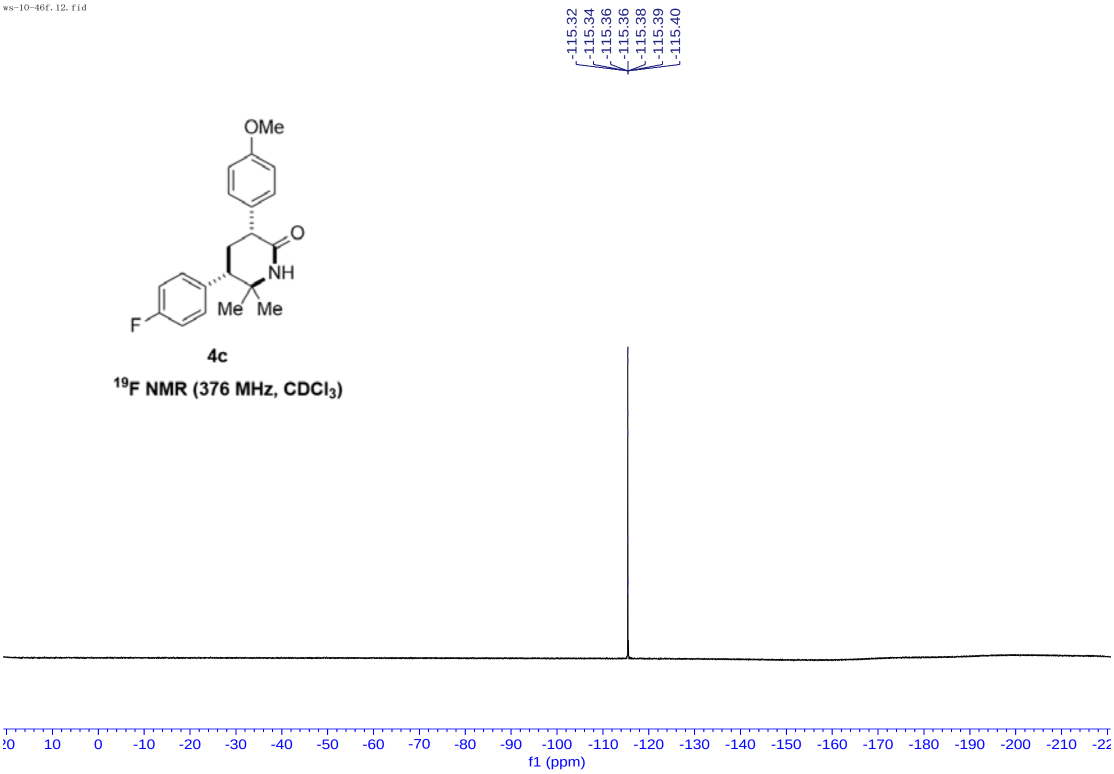

Supplementary Figure 112 ^{19}F NMR (376 MHz, 298K, $CDCl_3$) of **4c**

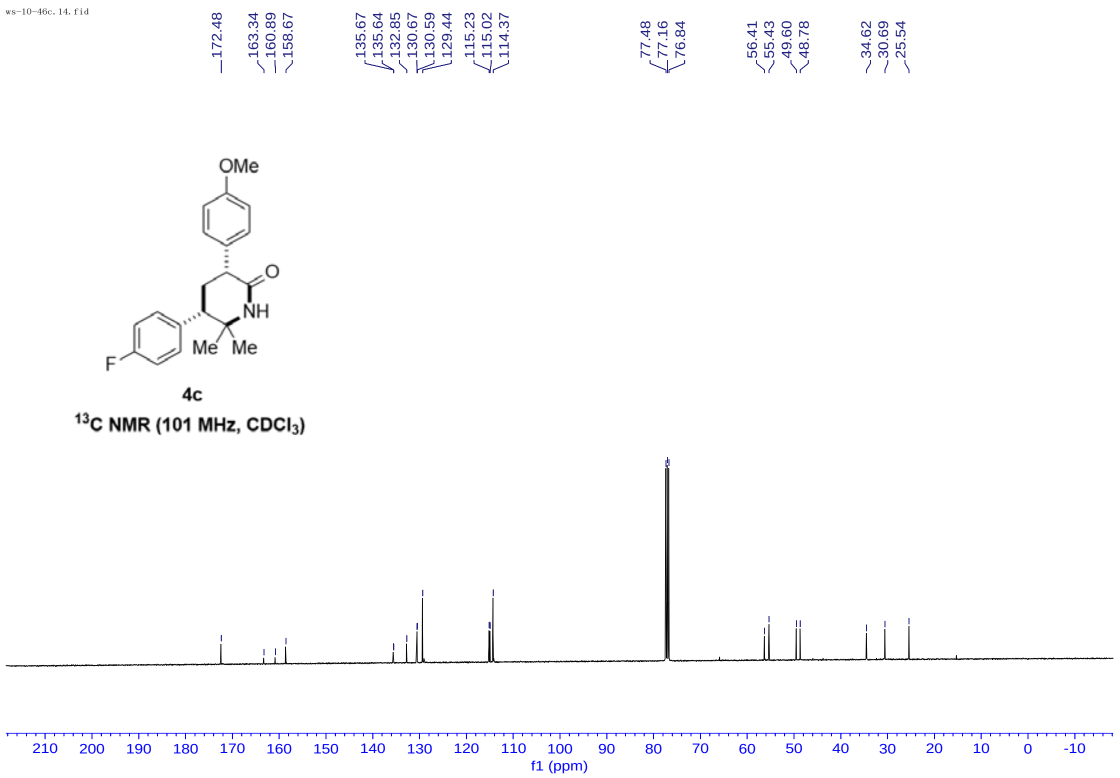

Supplementary Figure 113 ^{13}C NMR (101 MHz, 298K, $CDCl_3$) of **4c**

***cis*-3-(2-fluorophenyl)-5-(4-fluorophenyl)-6,6-dimethylpiperidin-2-one**

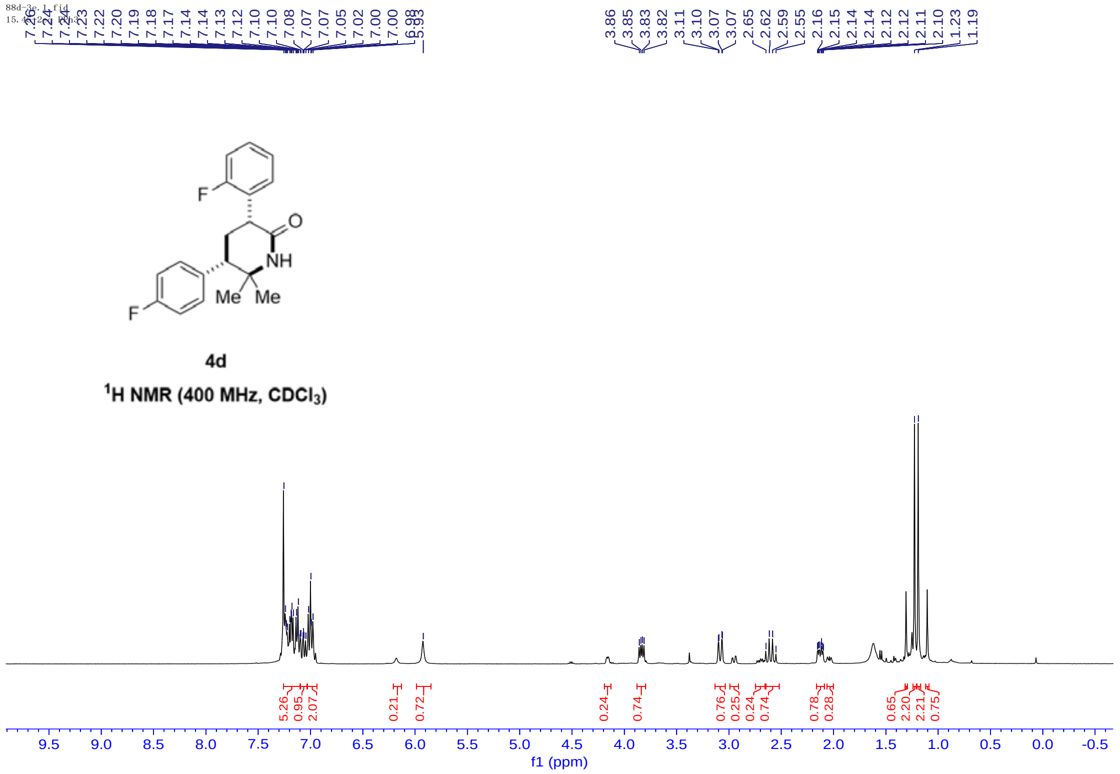

Supplementary Figure 114 ^{1}H NMR (400 MHz, 298K, $CDCl_3$) of **4d**

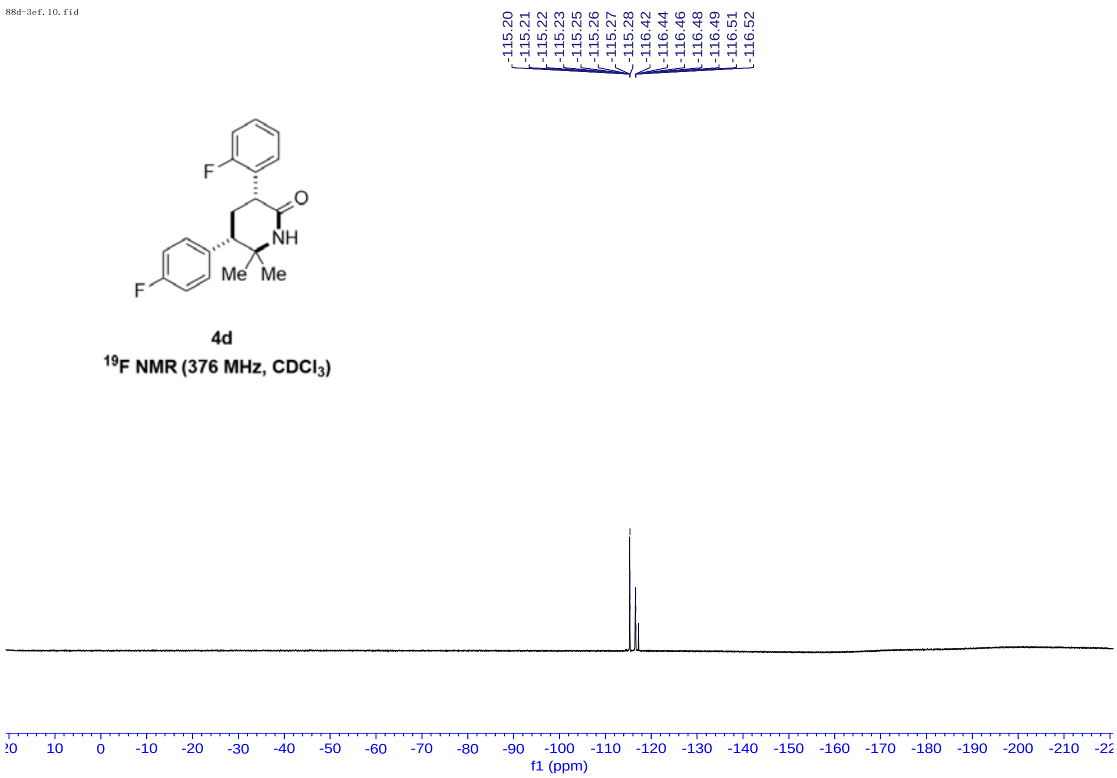

Supplementary Figure 115 ^{19}F NMR (376 MHz, 298K, $CDCl_3$) of **4d**

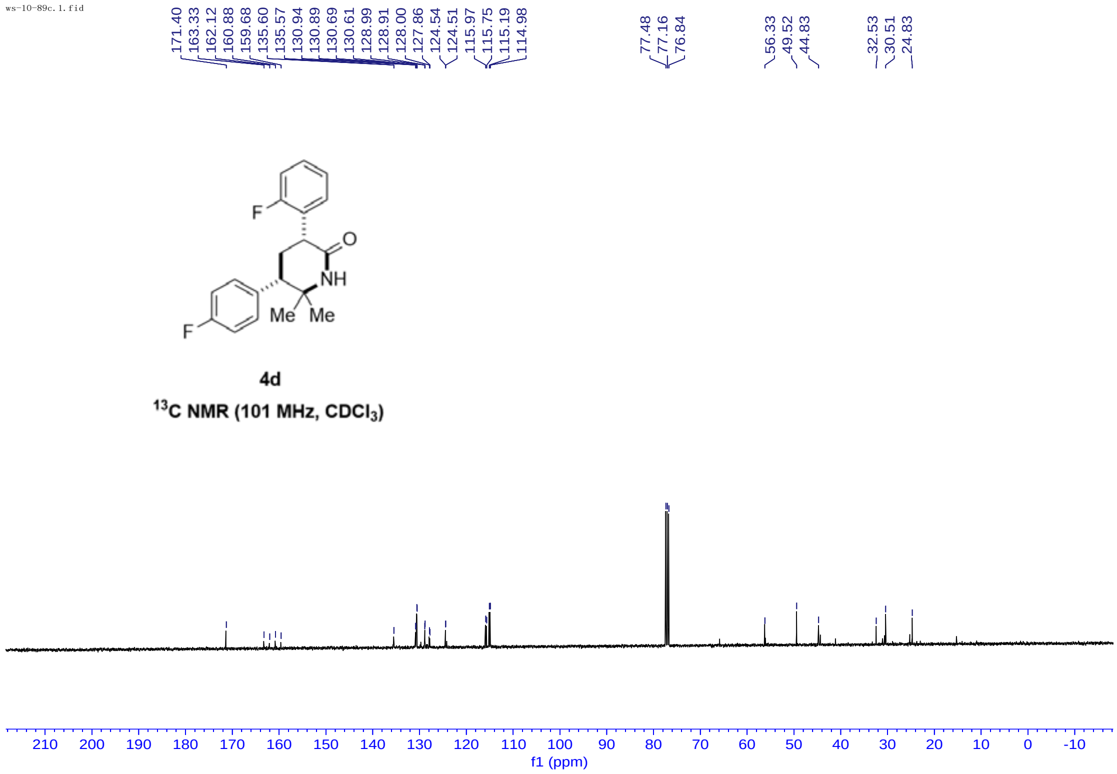

Supplementary Figure 116 ^{13}C NMR (101 MHz, 298K, $CDCl_3$) of **4d**

cis-5-(4-fluorophenyl)-3-(2-methoxyphenyl)-6,6-dimethylpiperidin-2-one

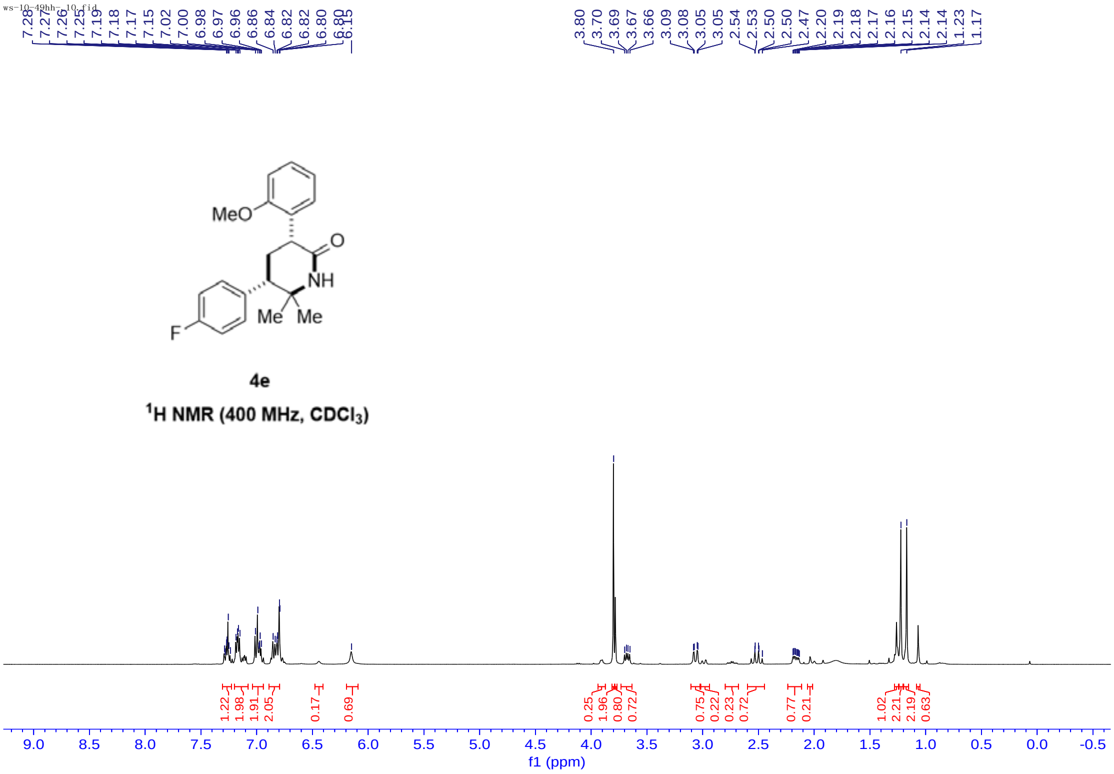

Supplementary Figure 117 ^{1}H NMR (400 MHz, 298K, $CDCl_3$) of **4e**

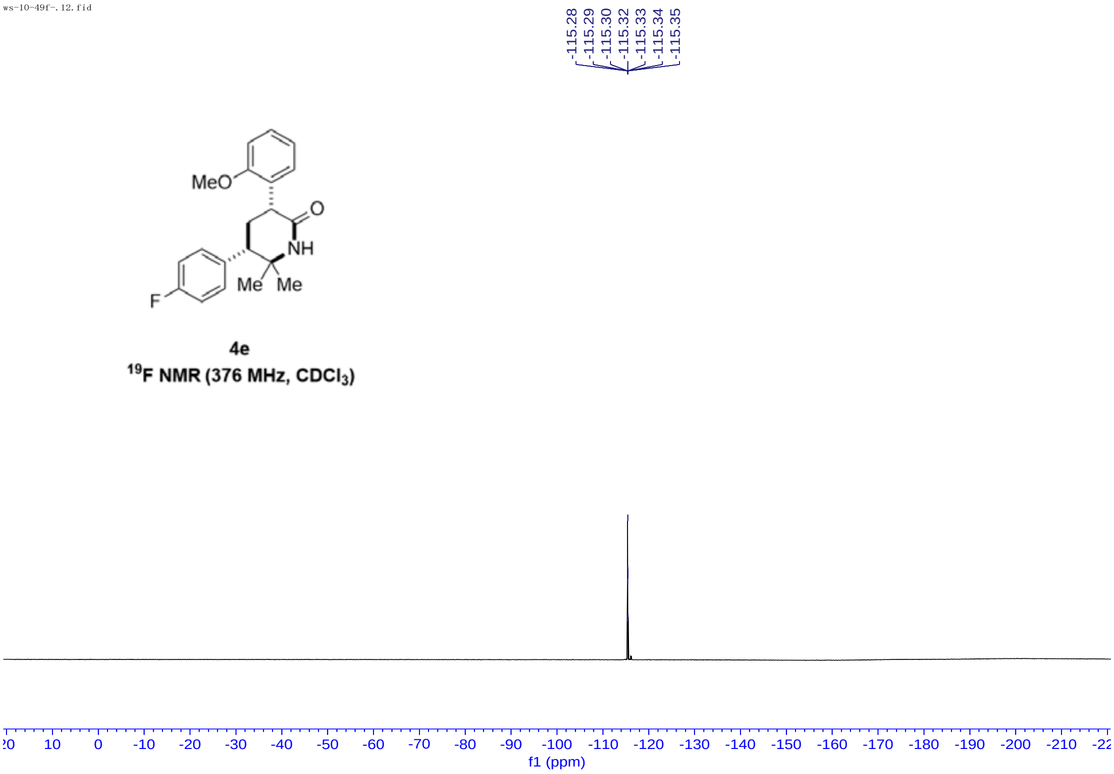

Supplementary Figure 118 ^{19}F NMR (376 MHz, 298K, $CDCl_3$) of **4e**

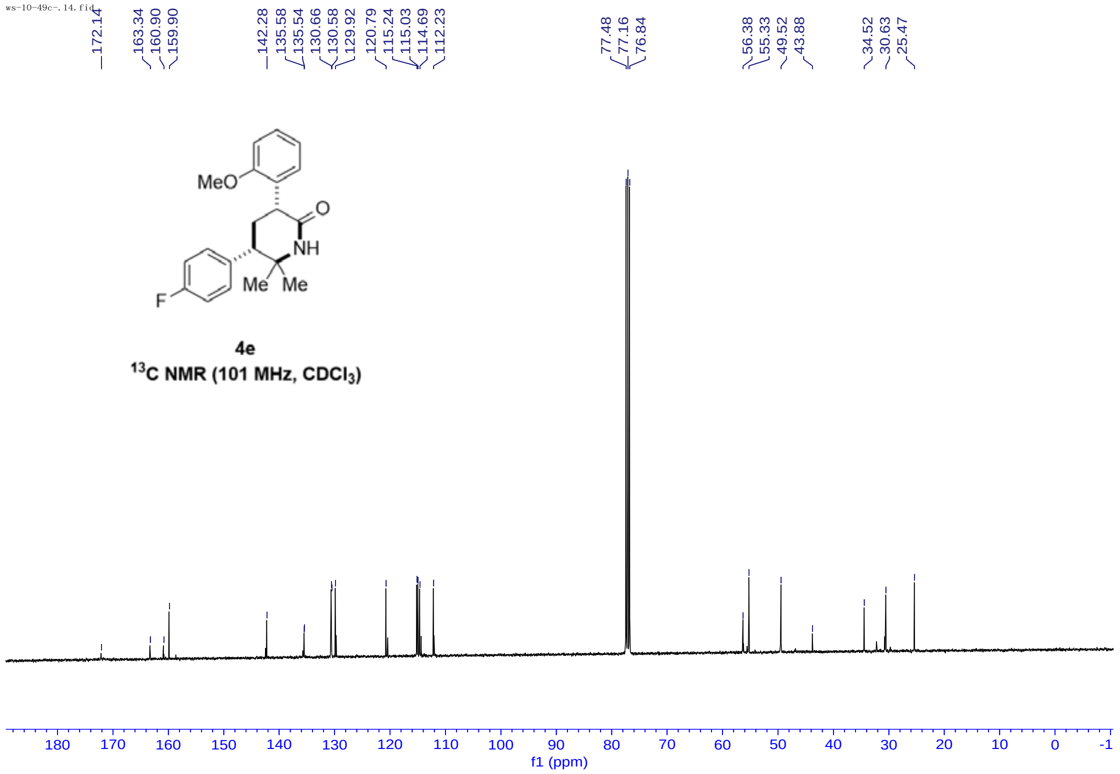

Supplementary Figure 119 ^{13}C NMR (101 MHz, 298K, $CDCl_3$) of **4e**

***cis*-5-(4-fluorophenyl)-3-(3-methoxyphenyl)-6,6-dimethylpiperidin-2-one**

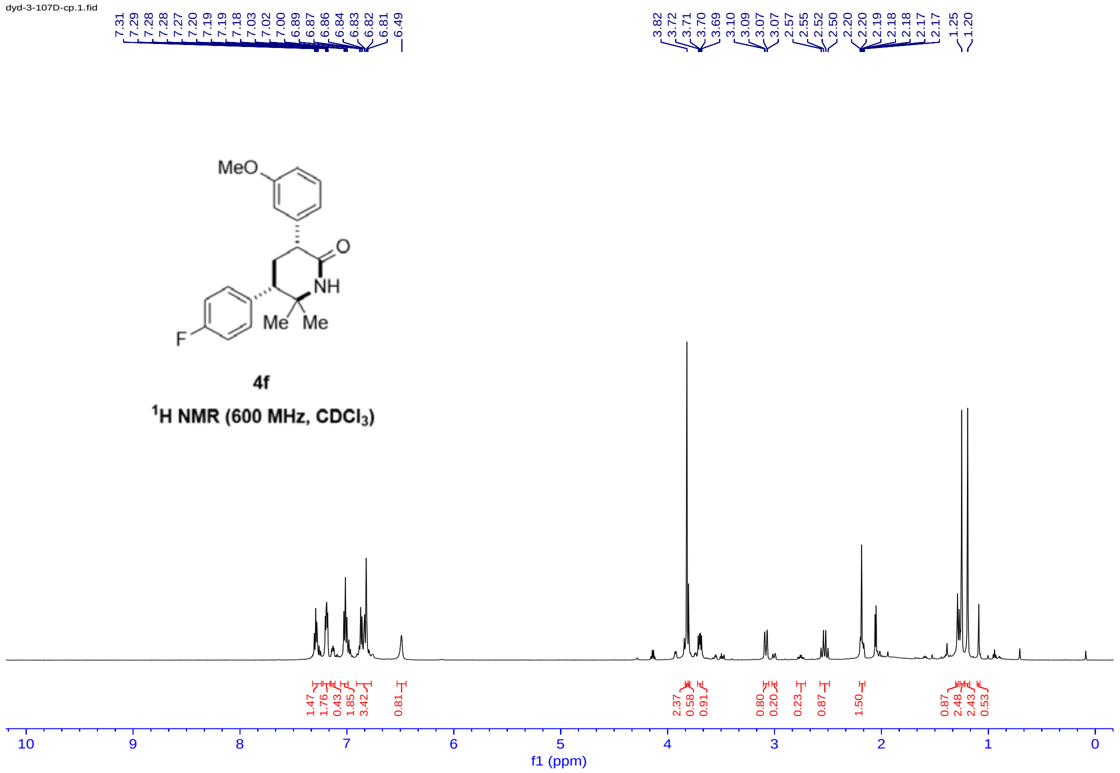

Supplementary Figure 120 ^{1}H NMR (600 MHz, 298K, $CDCl_3$) of **4f**

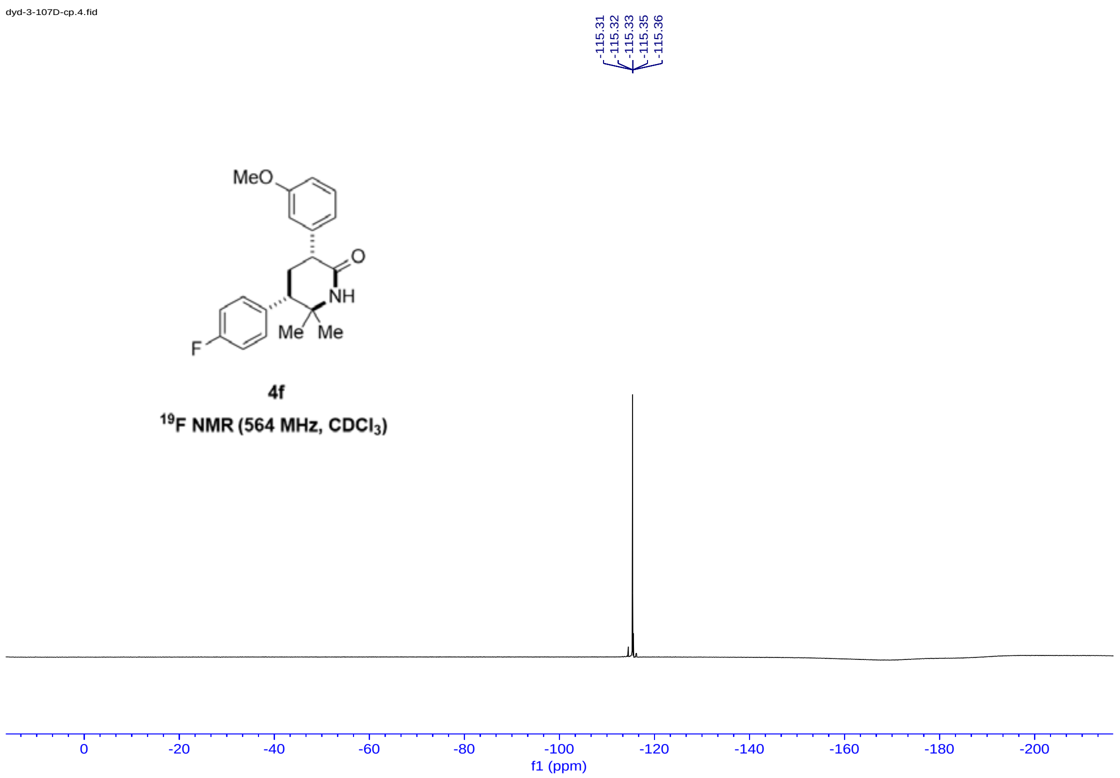

Supplementary Figure 121 ^{19}F NMR (564 MHz, 298K, $CDCl_3$) of **4f**

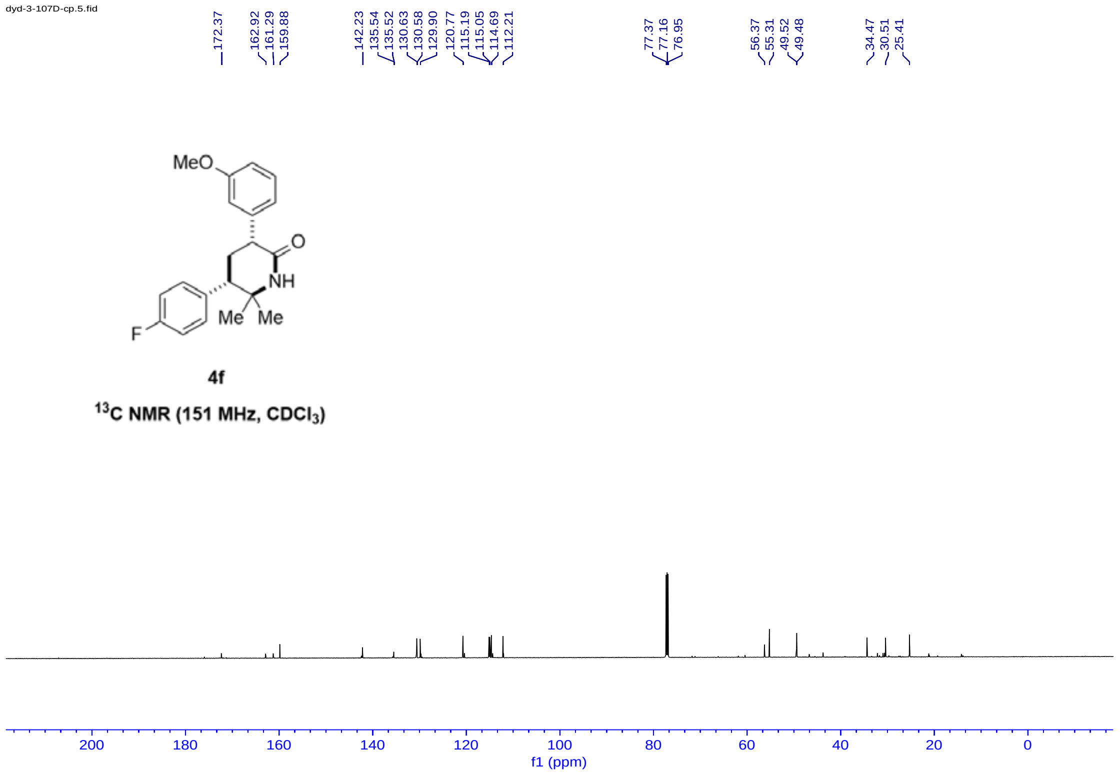

Supplementary Figure 122 ^{13}C NMR (151 MHz, 298K, $CDCl_3$) of **4f**

cis-3-(4-methoxyphenyl)-6,6-dimethyl-5-phenylpiperidin-2-one

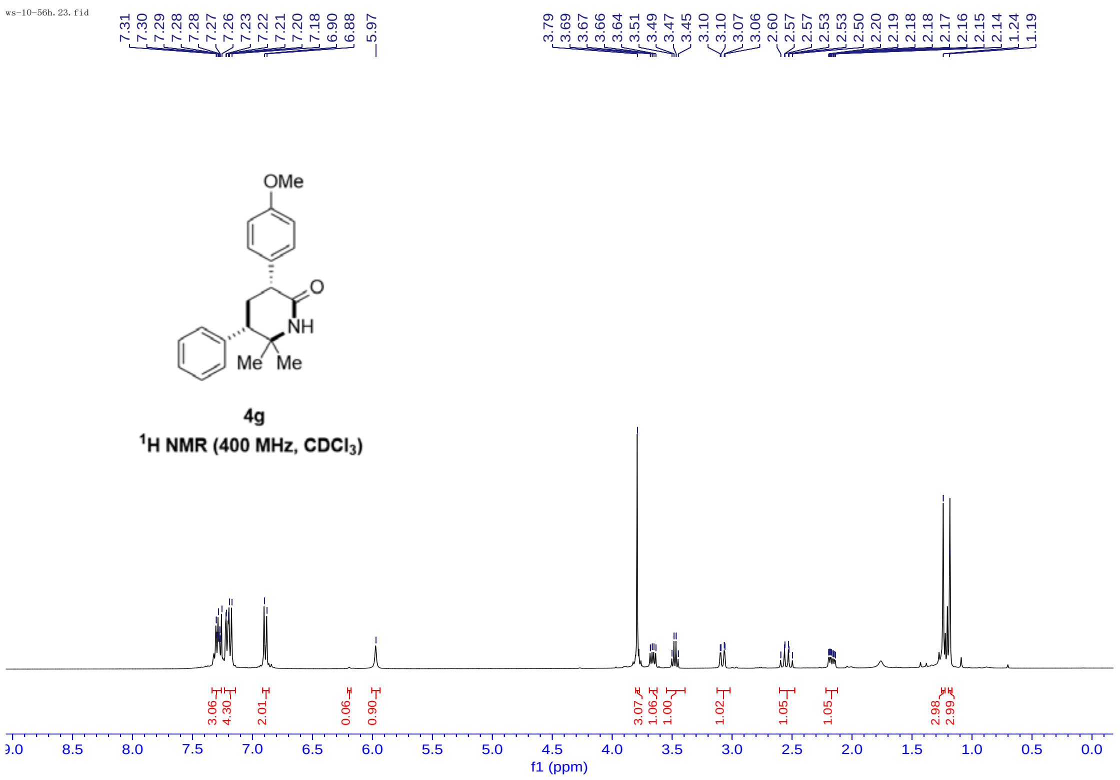

Supplementary Figure 123 ^{1}H NMR (400 MHz, 298K, $CDCl_3$) of **4g**

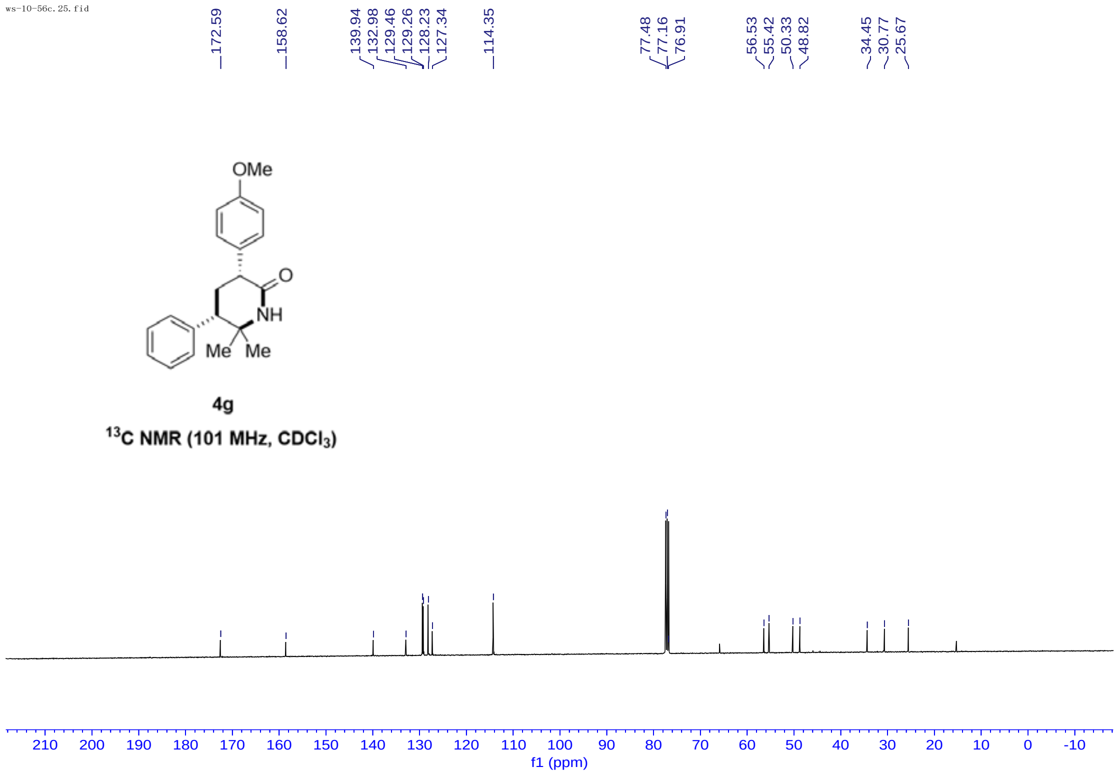

Supplementary Figure 124 ^{13}C NMR (101 MHz, 298K, $CDCl_3$) of **4g**

cis-3-(4-methoxyphenyl)-6,6-dimethyl-5-(*p*-tolyl)piperidin-2-one

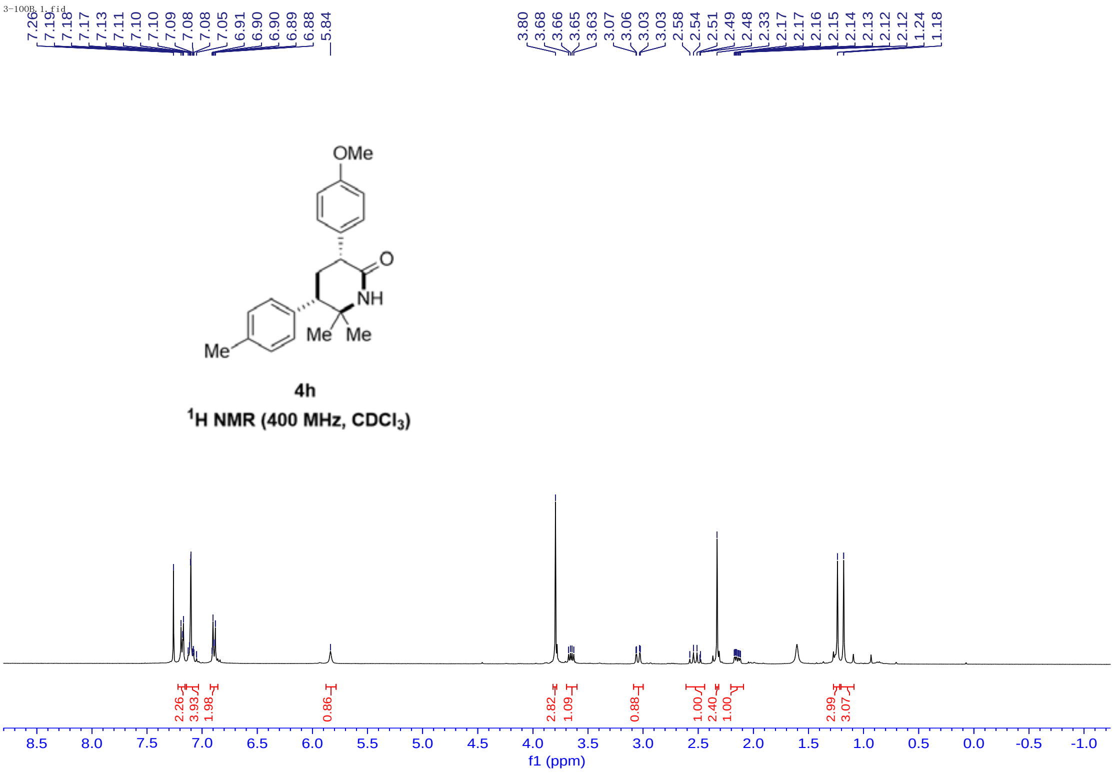

Supplementary Figure 125 ^{1}H NMR (400 MHz, 298K, $CDCl_3$) of **4h**

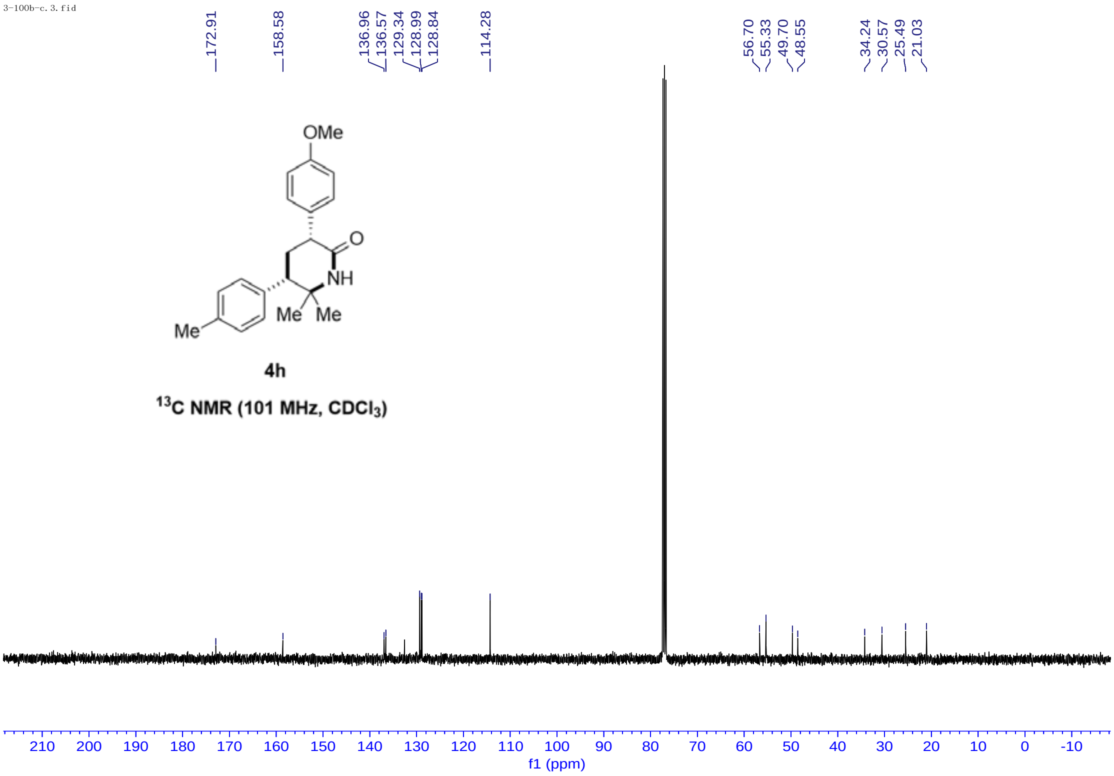

Supplementary Figure 126 ^{13}C NMR (101 MHz, 298K, $CDCl_3$) of **4h**

cis-5-(4-(*tert*-butyl)phenyl)-3-(4-methoxyphenyl)-6,6-dimethylpiperidin-2-one

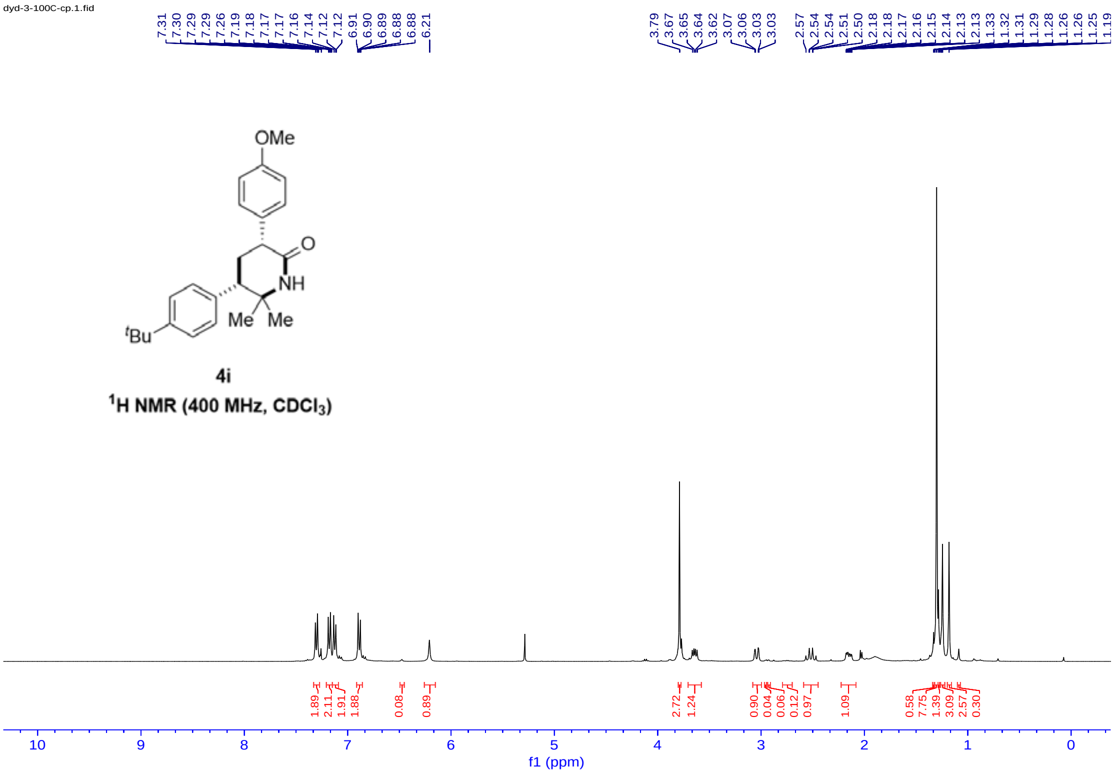

Supplementary Figure 127 ^{1}H NMR (400 MHz, 298K, $CDCl_3$) of **4i**

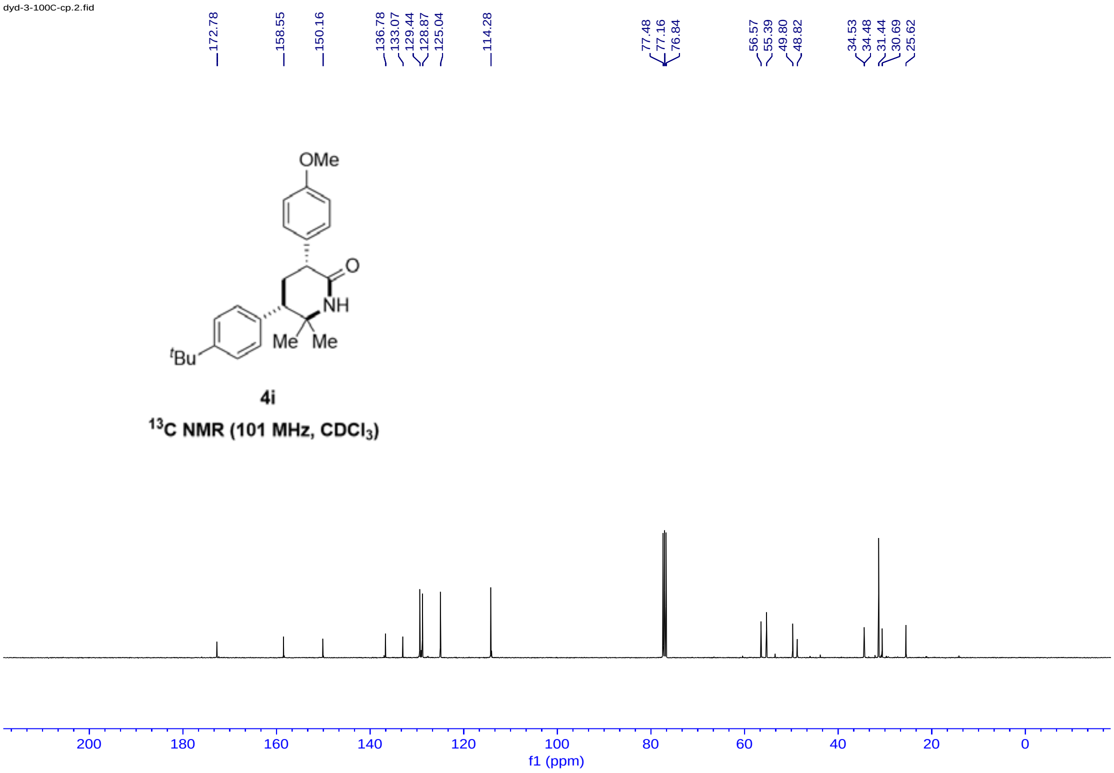

Supplementary Figure 128 ^{13}C NMR (101 MHz, 298K, $CDCl_3$) of **4i**

cis-3,5-bis(4-methoxyphenyl)-6,6-dimethylpiperidin-2-one

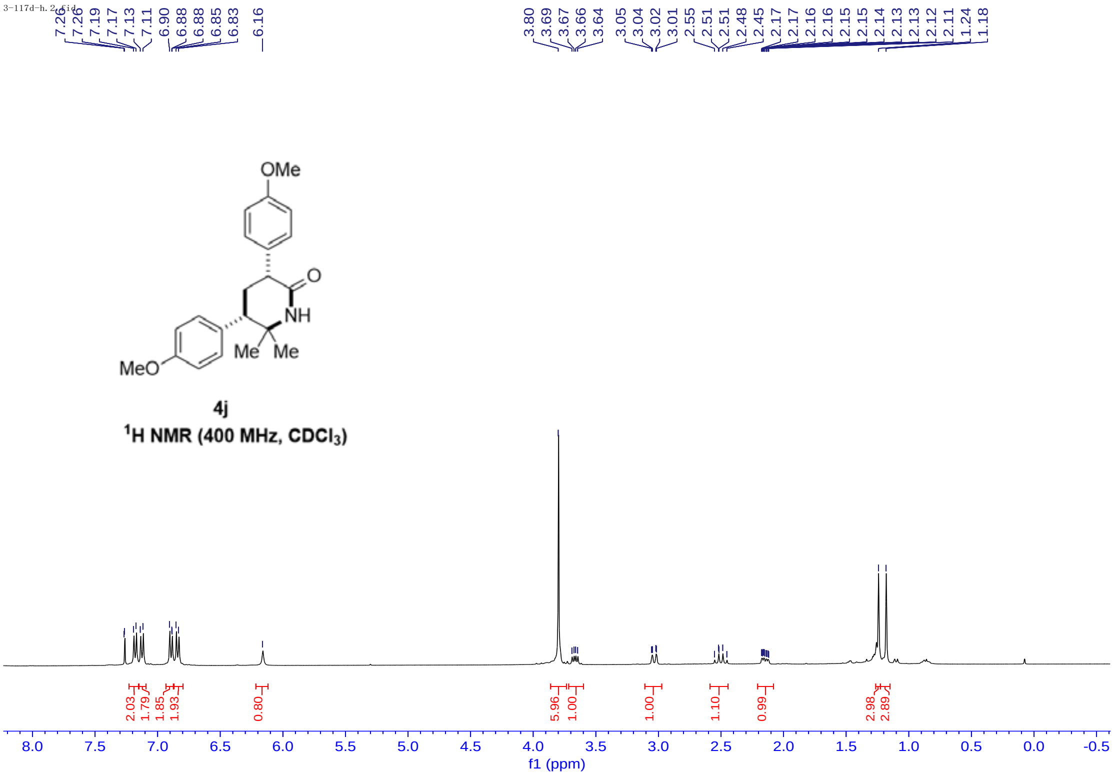

Supplementary Figure 129 ^{1}H NMR (400 MHz, 298K, $CDCl_3$) of **4j**

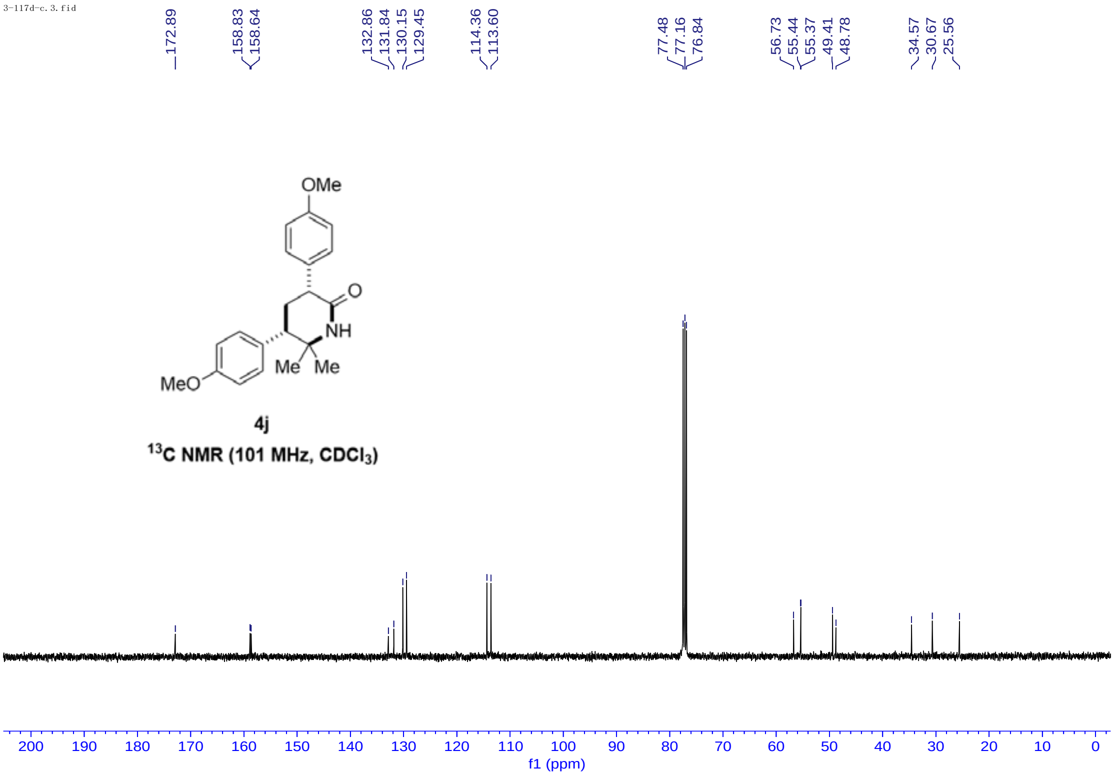

Supplementary Figure 130 ^{13}C NMR (101 MHz, 298K, $CDCl_3$) of **4j**

cis-5-(4-bromophenyl)-3-(4-methoxyphenyl)-6,6-dimethylpiperidin-2-one

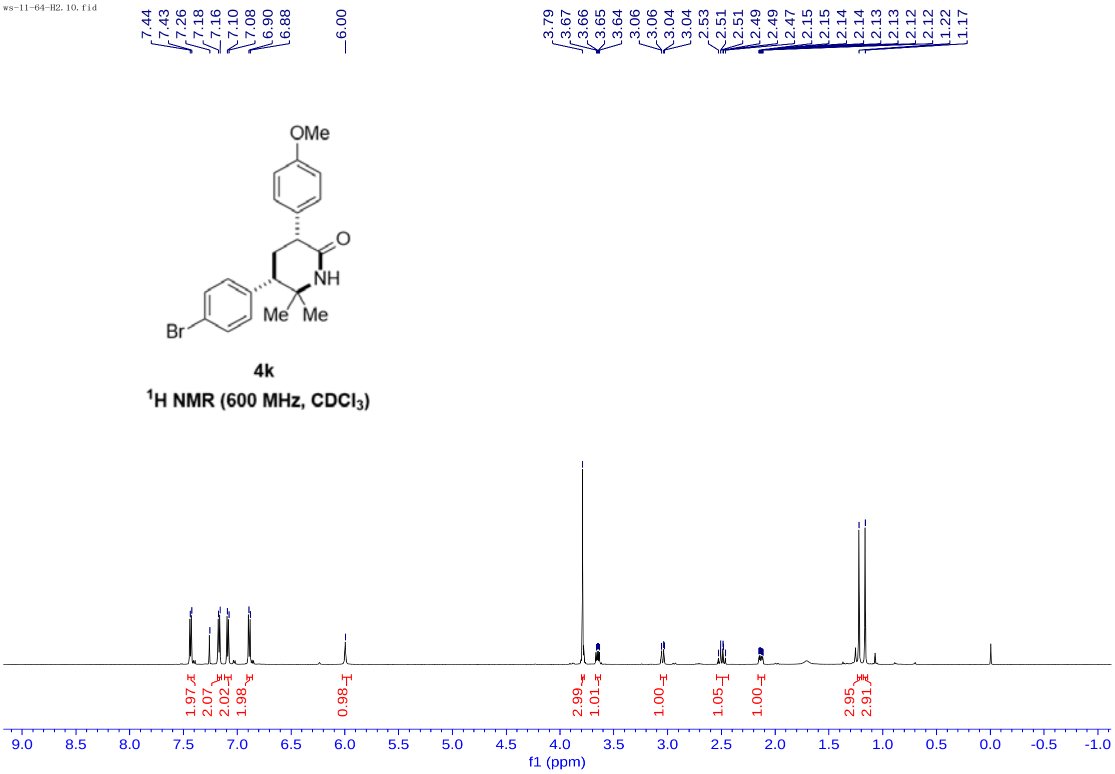

Supplementary Figure 131 ^{1}H NMR (600 MHz, 298K, $CDCl_3$) of **4k**

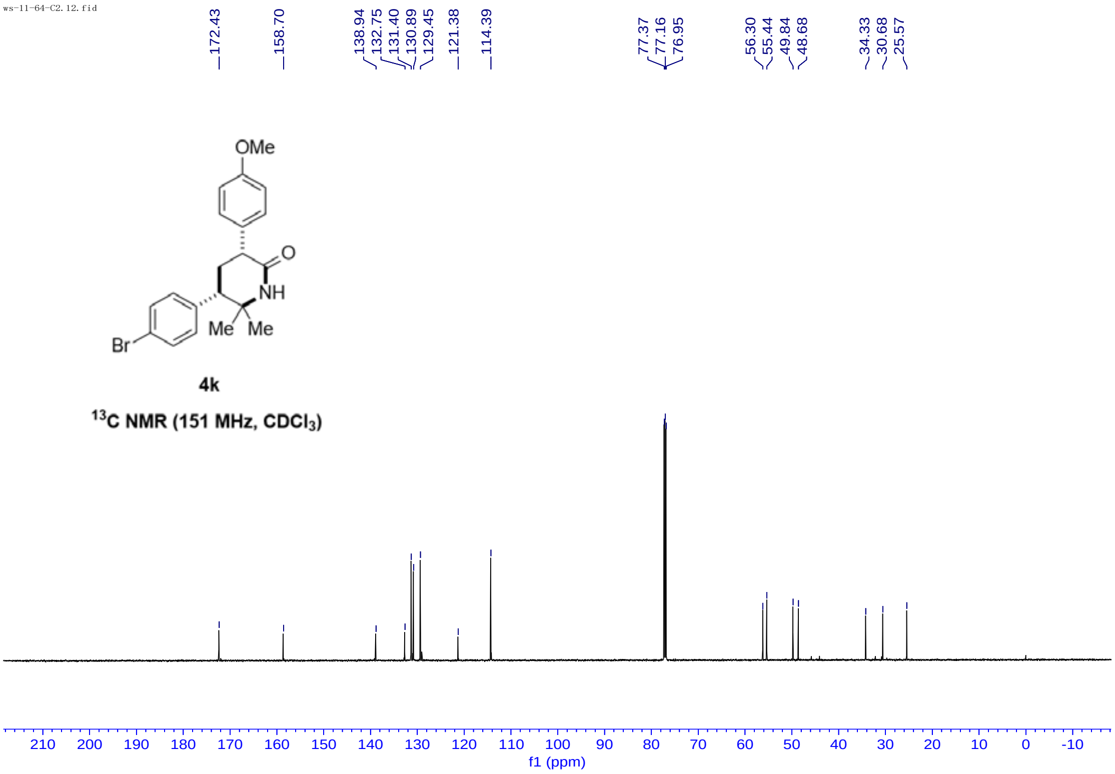

Supplementary Figure 132 ^{13}C NMR (151 MHz, 298K, $CDCl_3$) of **4k**

cis-5-([1,1'-biphenyl]-4-yl)-3-(4-methoxyphenyl)-6,6-dimethylpiperidin-2-one

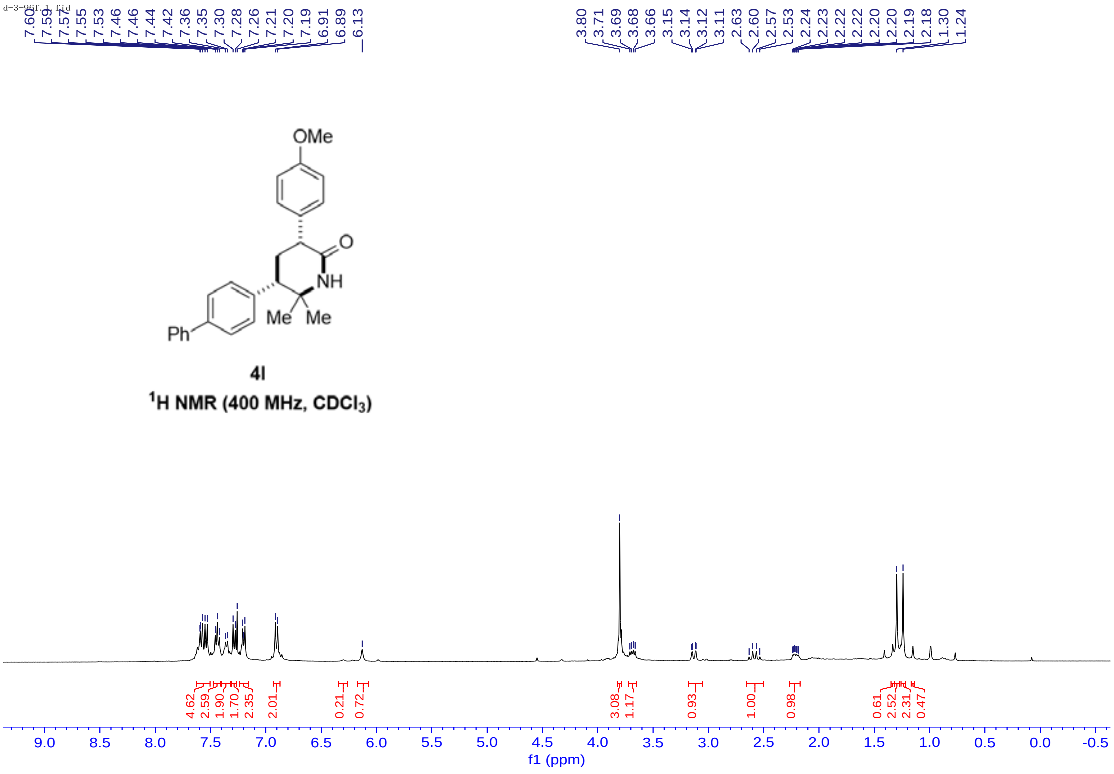

Supplementary Figure 133 ^{1}H NMR (400 MHz, 298K, $CDCl_3$) of **4l**

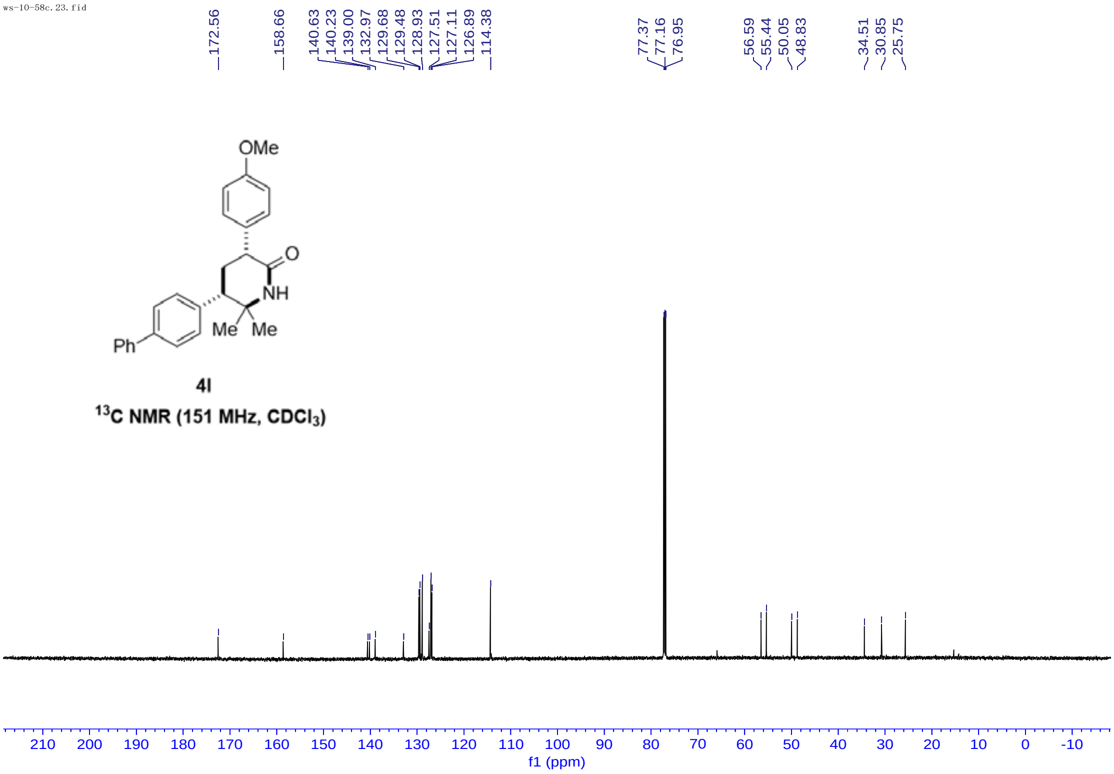

Supplementary Figure 134 ^{13}C NMR (151 MHz, 298K, $CDCl_3$) of **4l**

cis-3-(4-methoxyphenyl)-6,6-dimethyl-5-(m-tolyl)piperidin-2-one

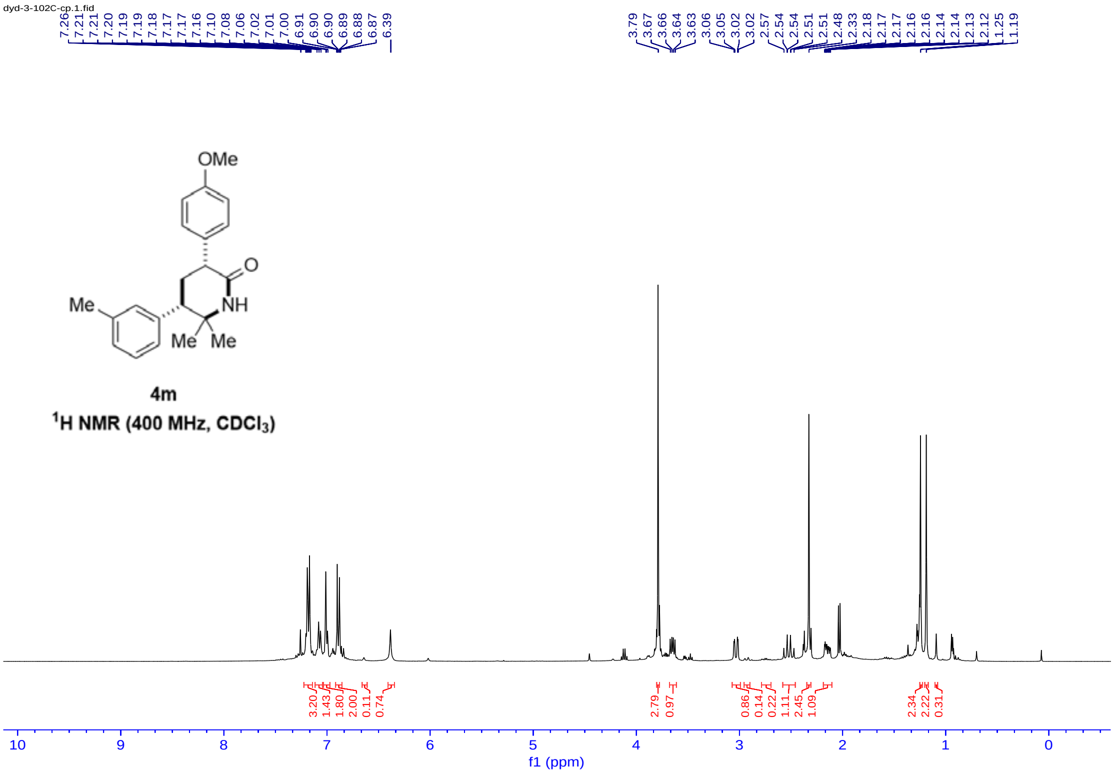

Supplementary Figure 135 ^{1}H NMR (400 MHz, 298K, $CDCl_3$) of **4m**

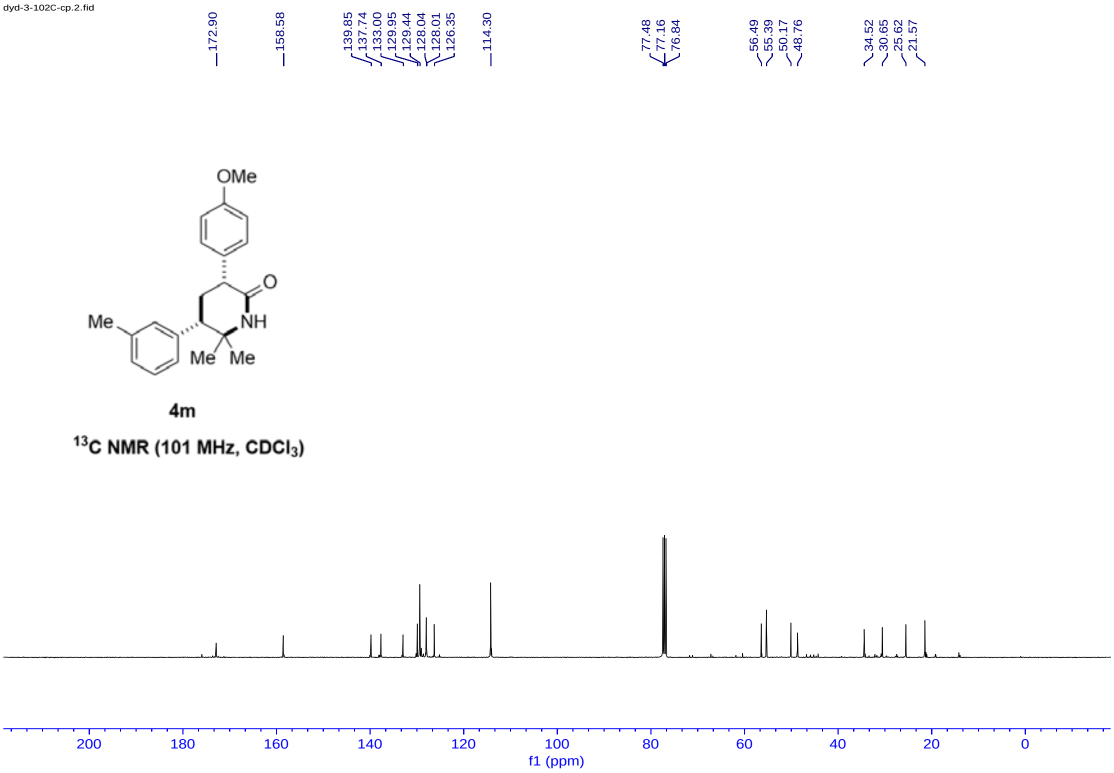

Supplementary Figure 136 ^{13}C NMR (101 MHz, 298K, $CDCl_3$) of **4m**

cis-3-(4-methoxyphenyl)-6,6-dimethyl-5-(thiophen-2-yl)piperidin-2-one

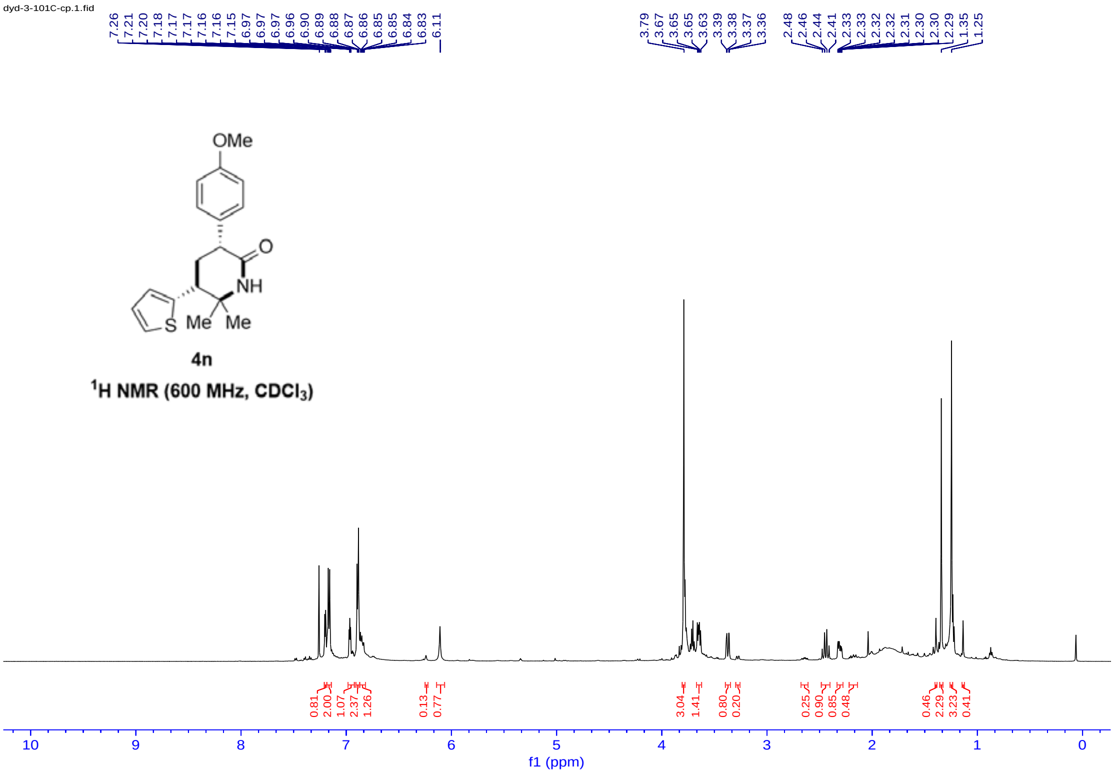

Supplementary Figure 137 ^{1}H NMR (600 MHz, 298K, $CDCl_3$) of **4n**

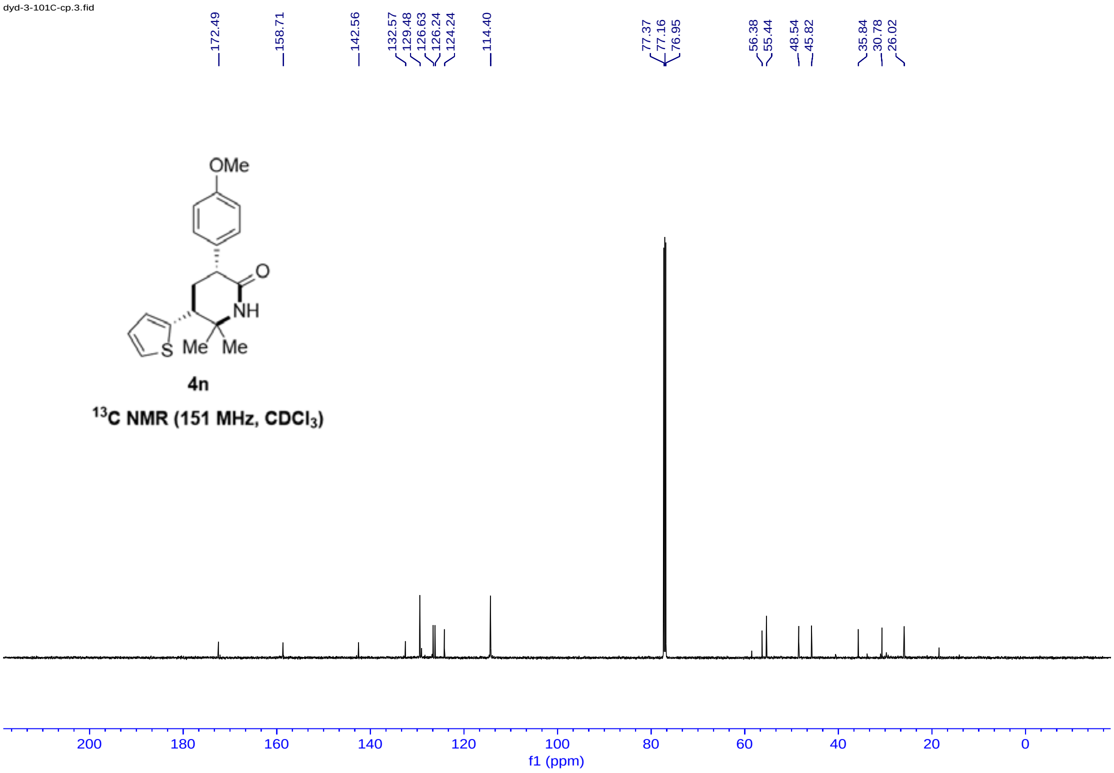

Supplementary Figure 138 ^{13}C NMR (151 MHz, 298K, $CDCl_3$) of **4n**

(3S,5S,6R)-3-(4-methoxyphenyl)-5,6-diphenylpiperidin-2-one

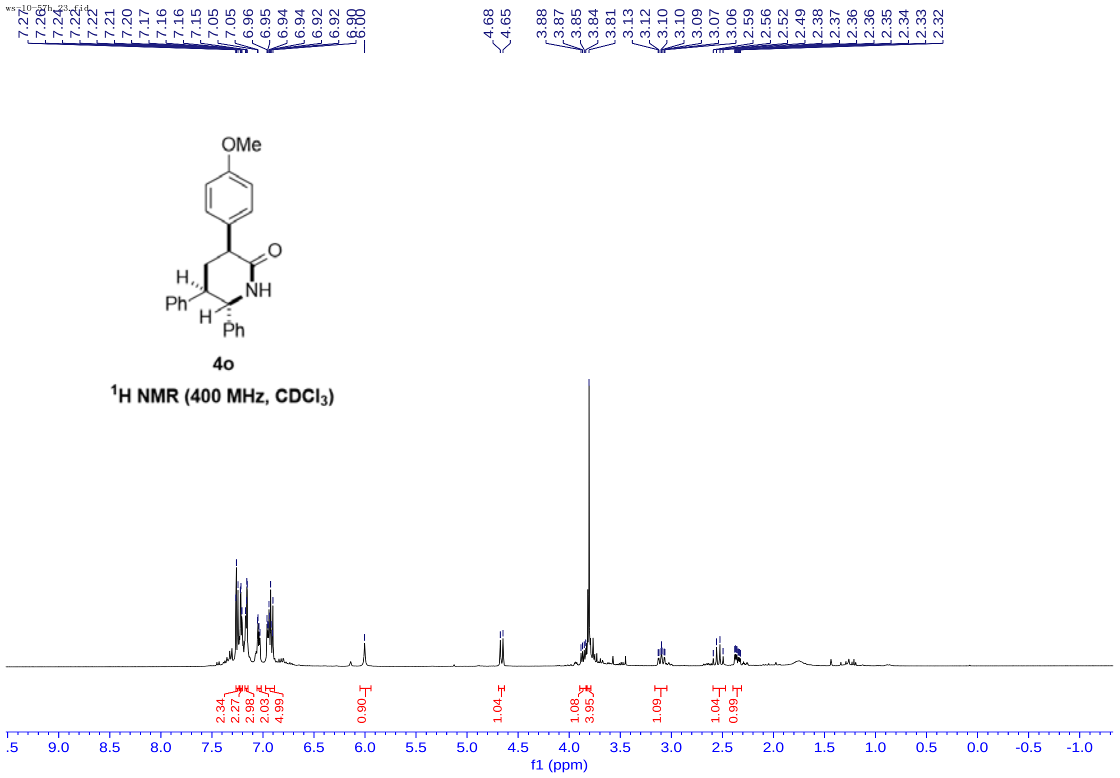

Supplementary Figure 139 ^{1}H NMR (400 MHz, 298K, $CDCl_3$) of **4o**

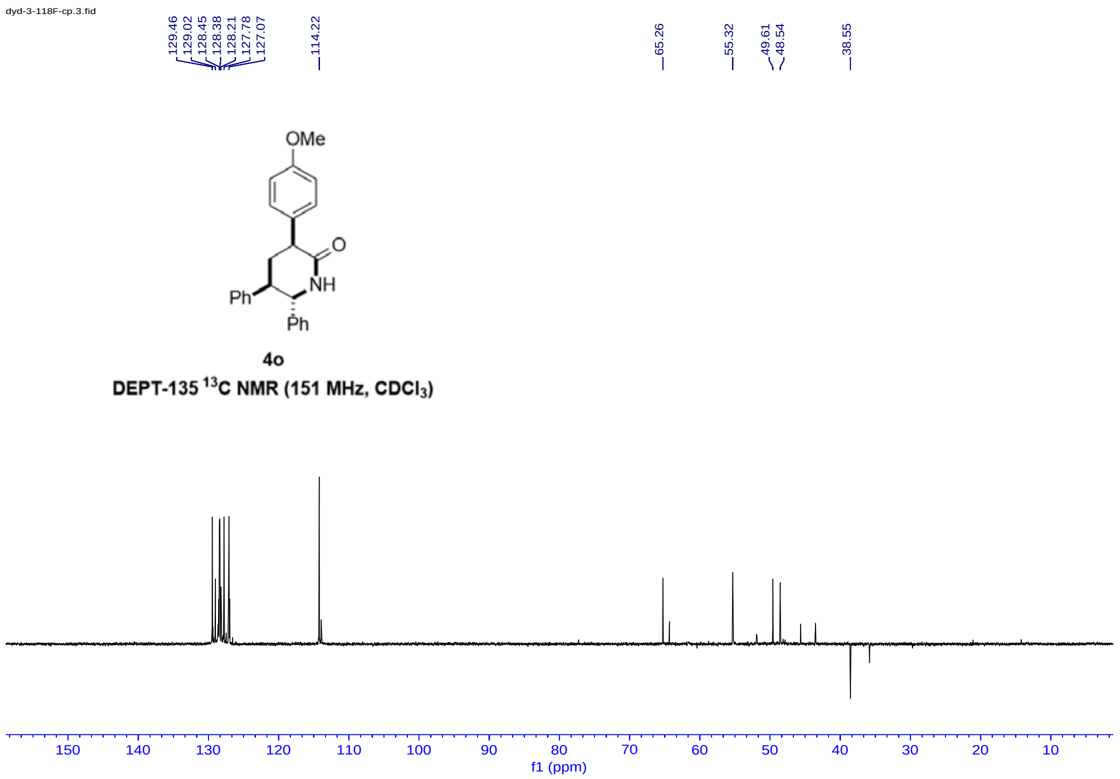

Supplementary Figure 140 DEPT-135 ^{13}C NMR (151 MHz, 298K, $CDCl_3$) of **4o**

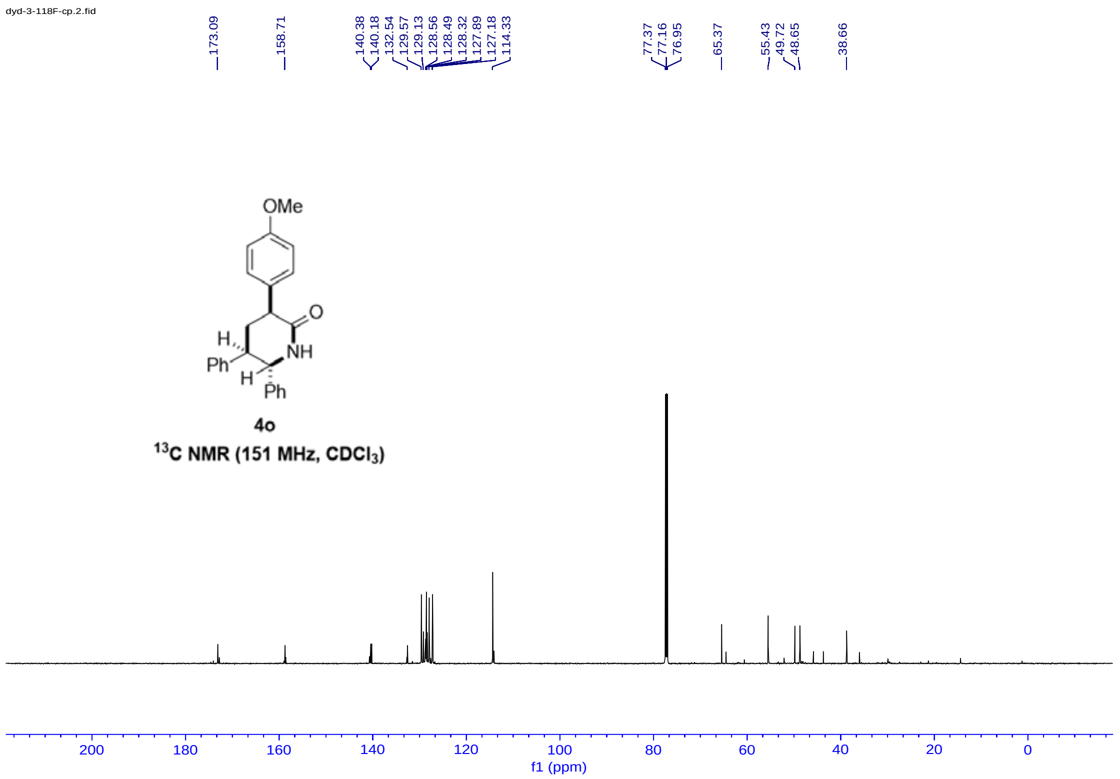

Supplementary Figure 141 ^{13}C NMR (151 MHz, 298K, $CDCl_3$) of **4o**

5-(4-(*tert*-butyl)phenyl)-3-(4-(trifluoromethyl)phenyl)piperidin-2-one

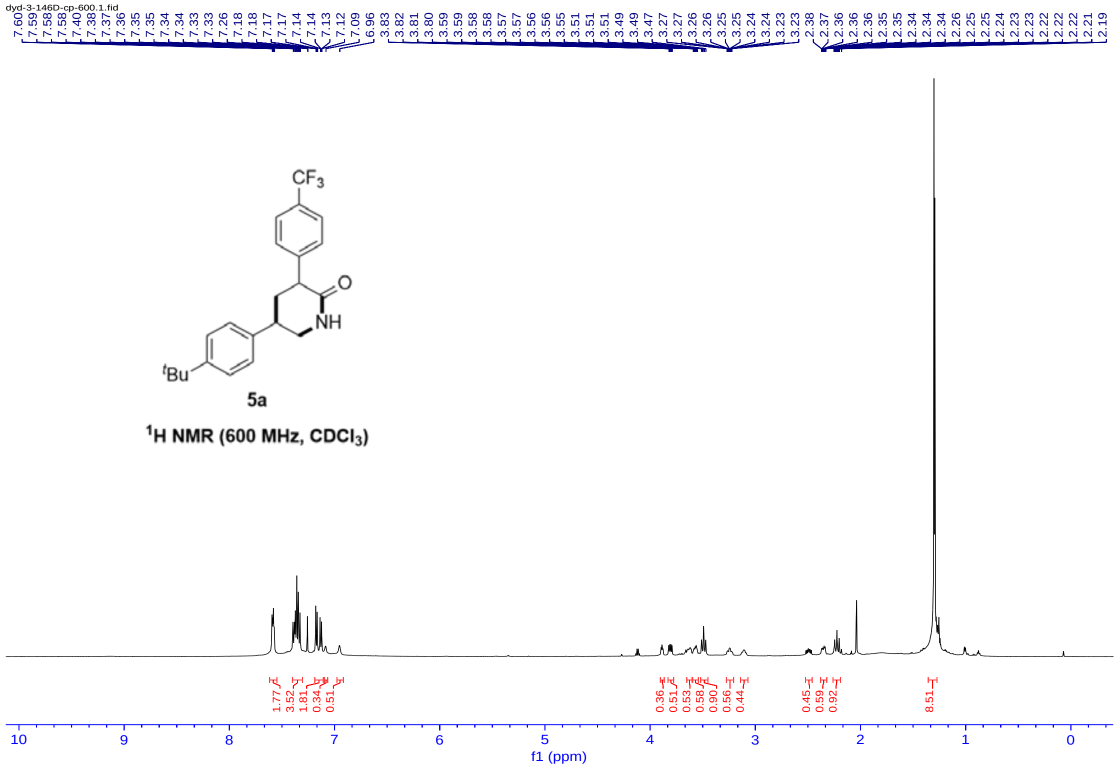

Supplementary Figure 142 ^{1}H NMR (600 MHz, 298K, $CDCl_3$) of **5a**

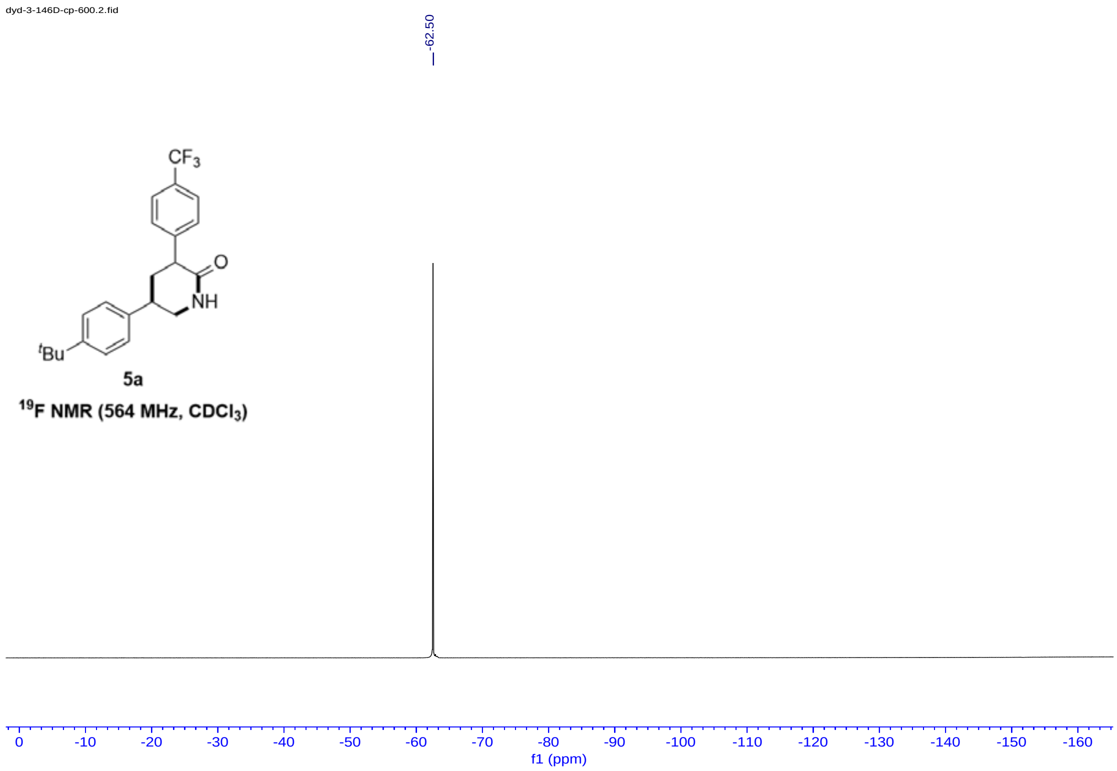

Supplementary Figure 143 ^{19}F NMR (564 MHz, 298K, $CDCl_3$) of **5a**

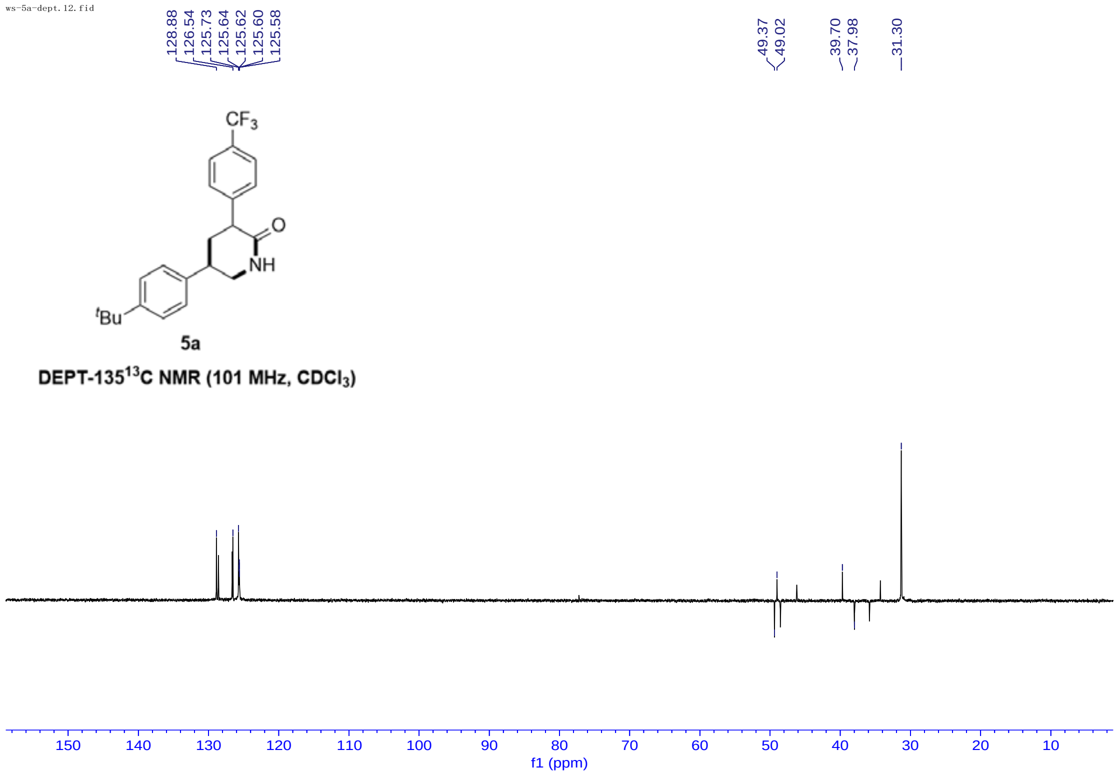

Supplementary Figure 144 DEPT-135 ^{13}C NMR (101 MHz, 298K, $CDCl_3$) of **5a**

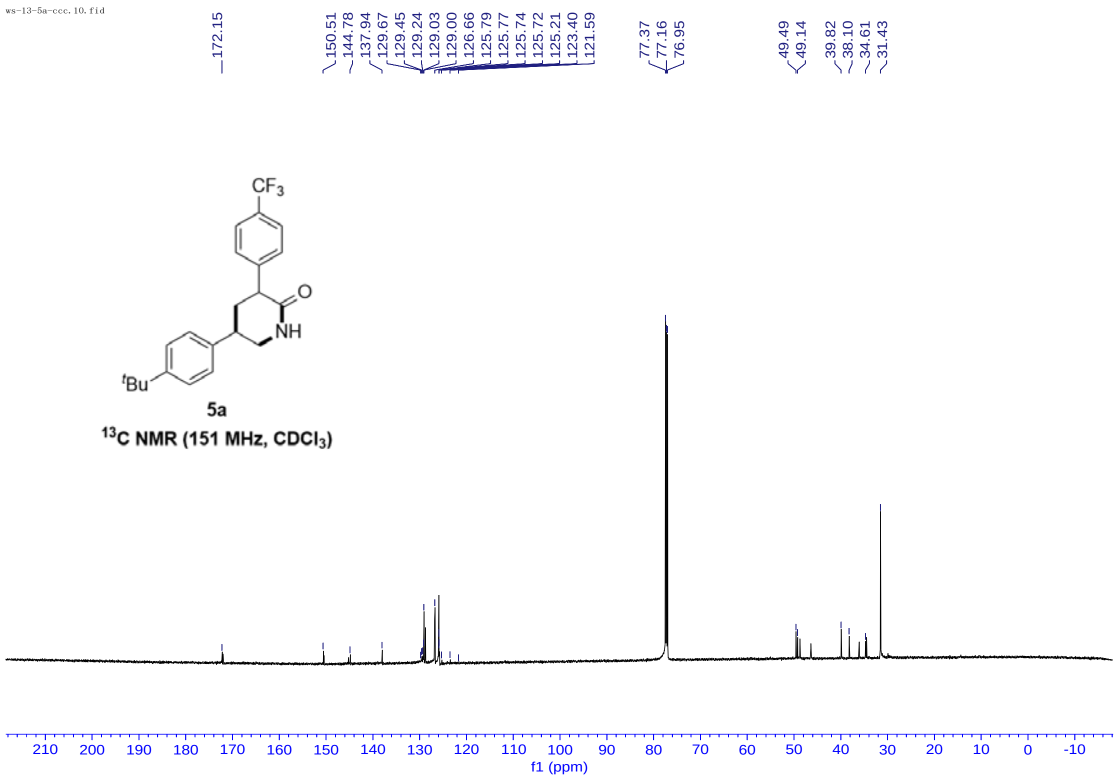

Supplementary Figure 145 ^{13}C NMR (151 MHz, 298K, $CDCl_3$) of **5a**

5-(4-fluorophenyl)-3-(4-(trifluoromethyl)phenyl)piperidin-2-one

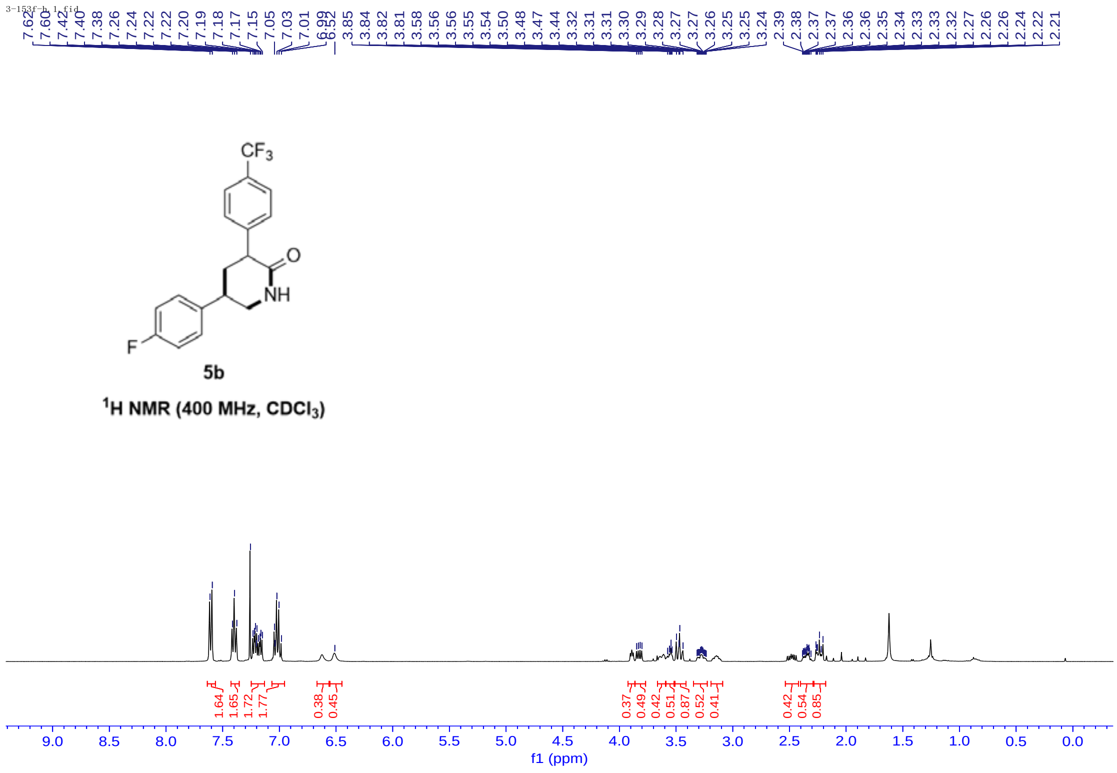

Supplementary Figure 146 ^{1}H NMR (400 MHz, 298K, $CDCl_3$) of **5b**

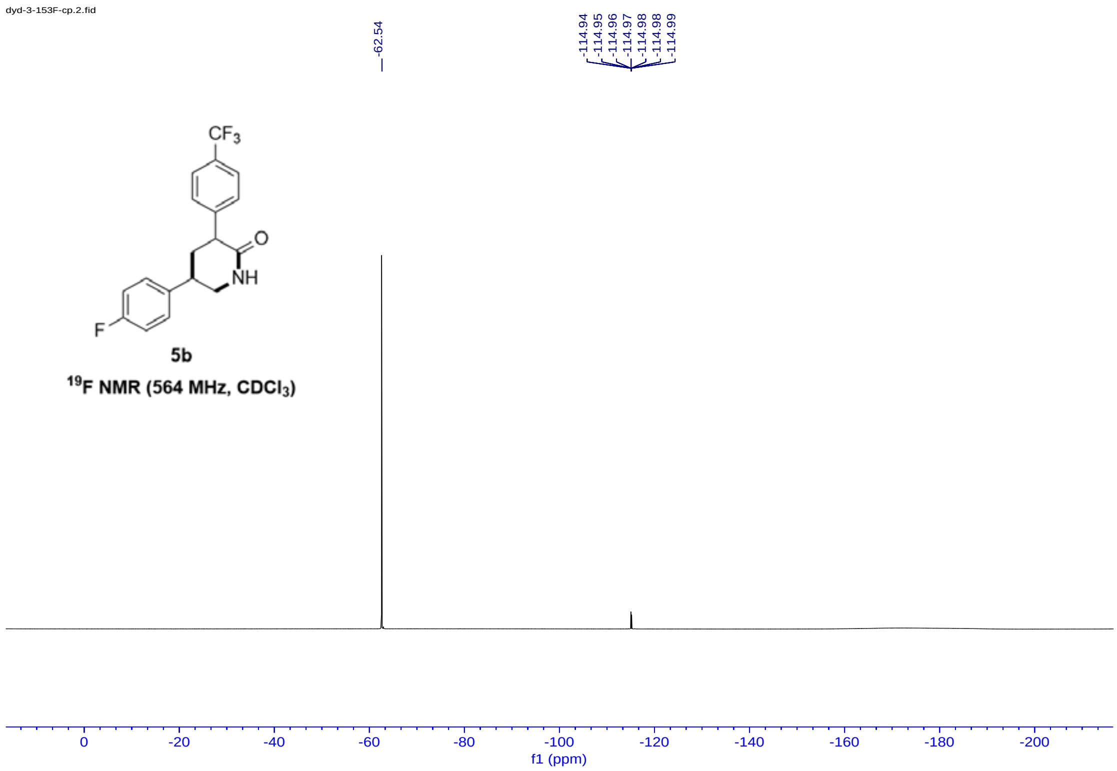

Supplementary Figure 147 ^{19}F NMR (564 MHz, 298K, $CDCl_3$) of **5b**

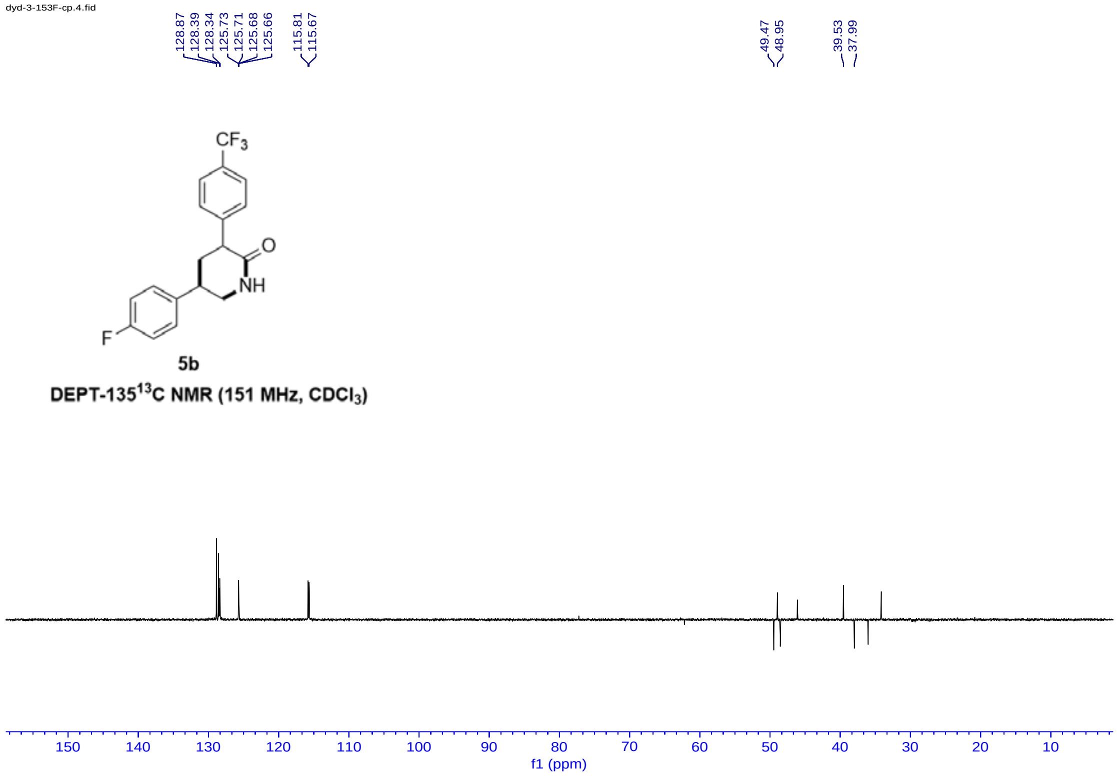

Supplementary Figure 148 DEPT-135 ^{13}C NMR (151 MHz, 298K, $CDCl_3$) of **5b**

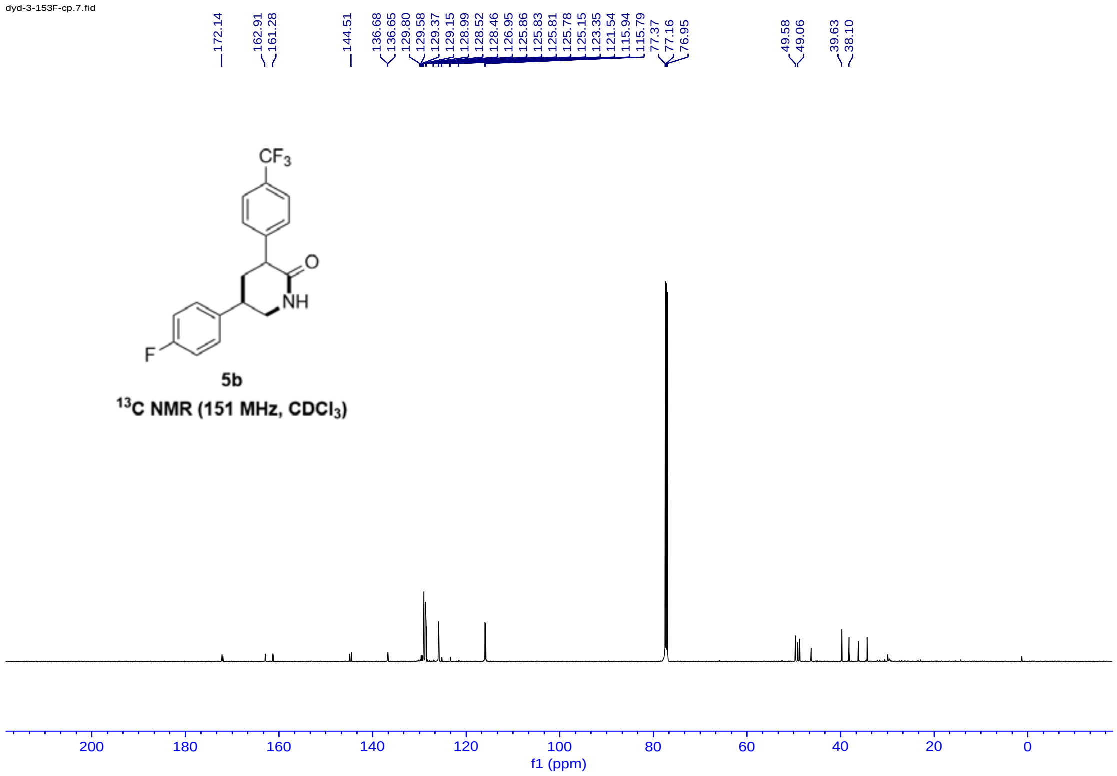

Supplementary Figure 149 ^{13}C NMR (151 MHz, 298K, $CDCl_3$) of **5b**

3-(4-fluorophenyl)-5-phenylpiperidin-2-one

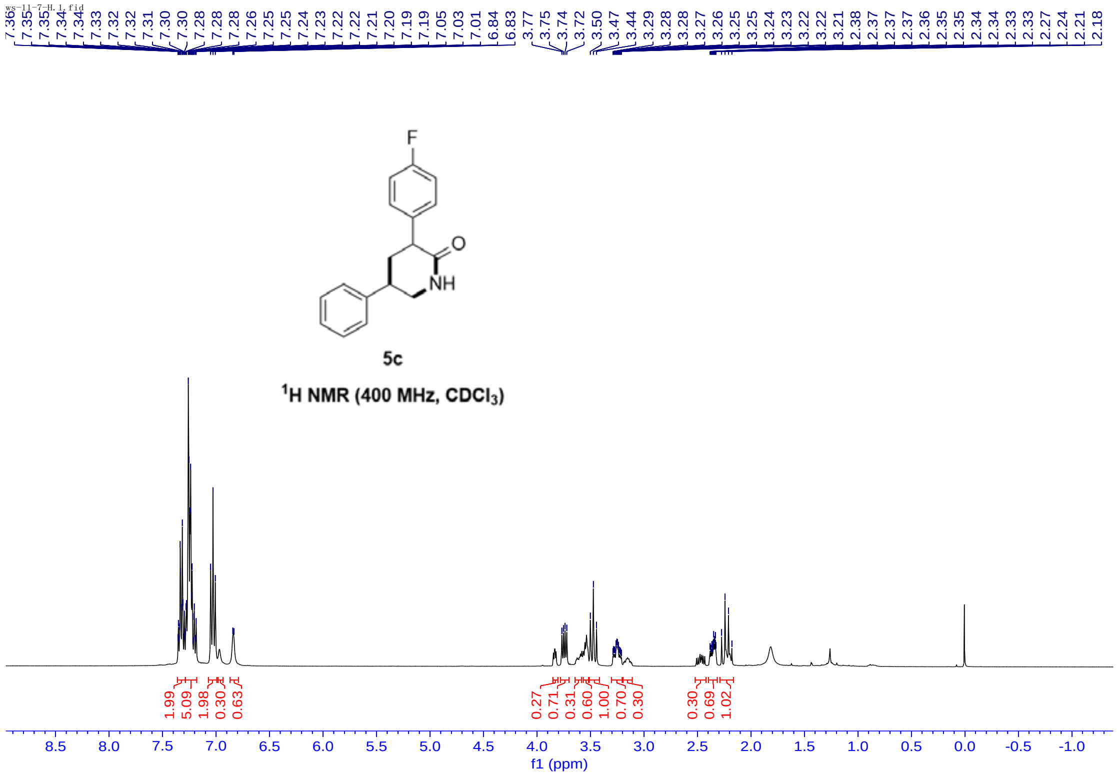

Supplementary Figure 150 ^{1}H NMR (400 MHz, 298K, $CDCl_3$) of **5c**

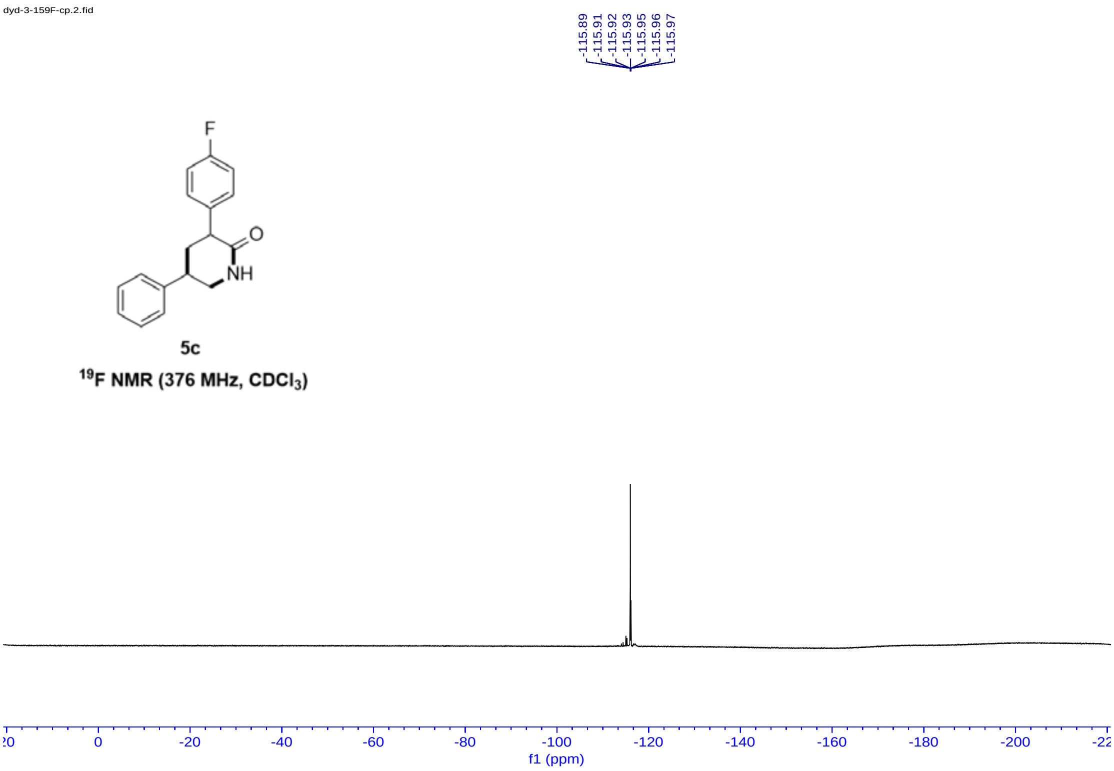

Supplementary Figure 151 ^{19}F NMR (376 MHz, 298K, $CDCl_3$) of **5c**

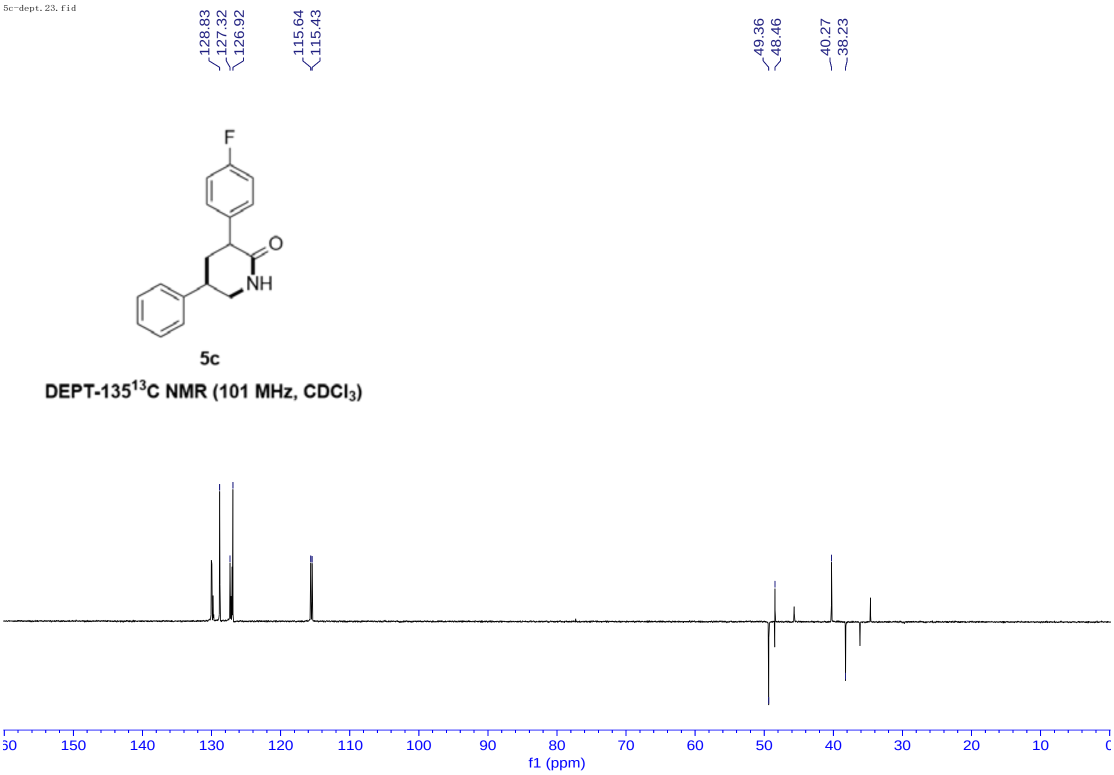

Supplementary Figure 152 DEPT-135 ^{13}C NMR (101 MHz, 298K, $CDCl_3$) of **5c**

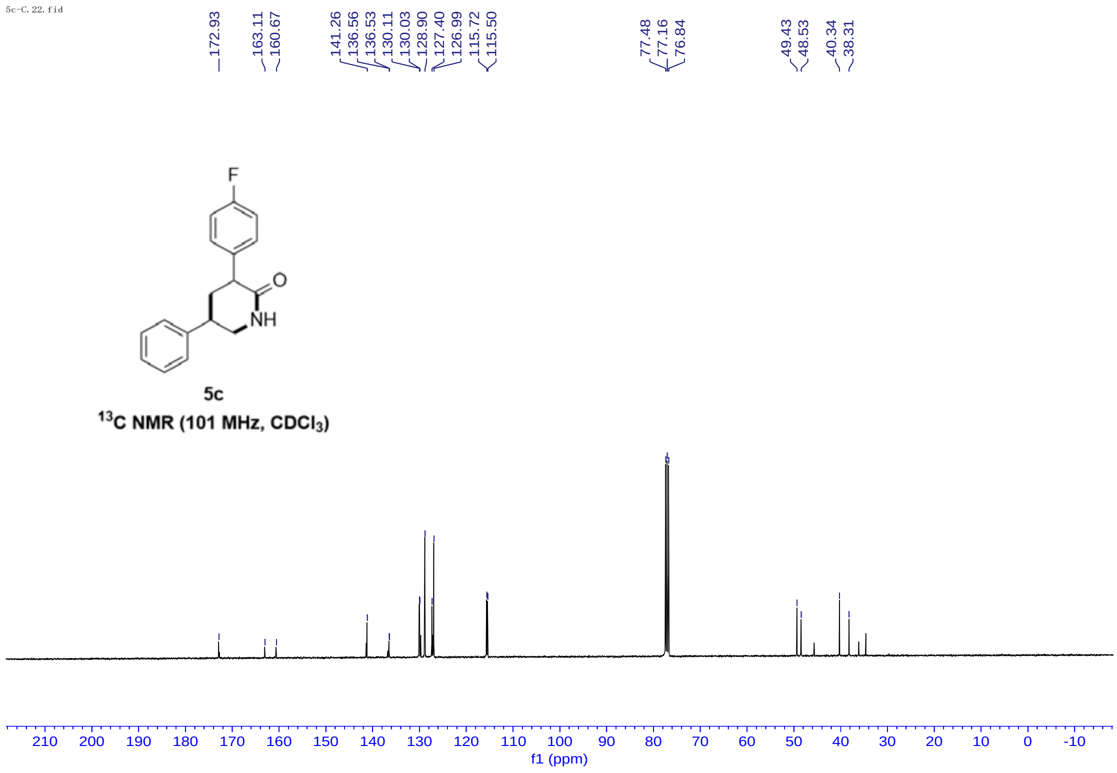

Supplementary Figure 153 ^{13}C NMR (101 MHz, 298K, $CDCl_3$) of **5c**

5-([1,1'-biphenyl]-4-yl)-3-(4-fluorophenyl)piperidin-2-one

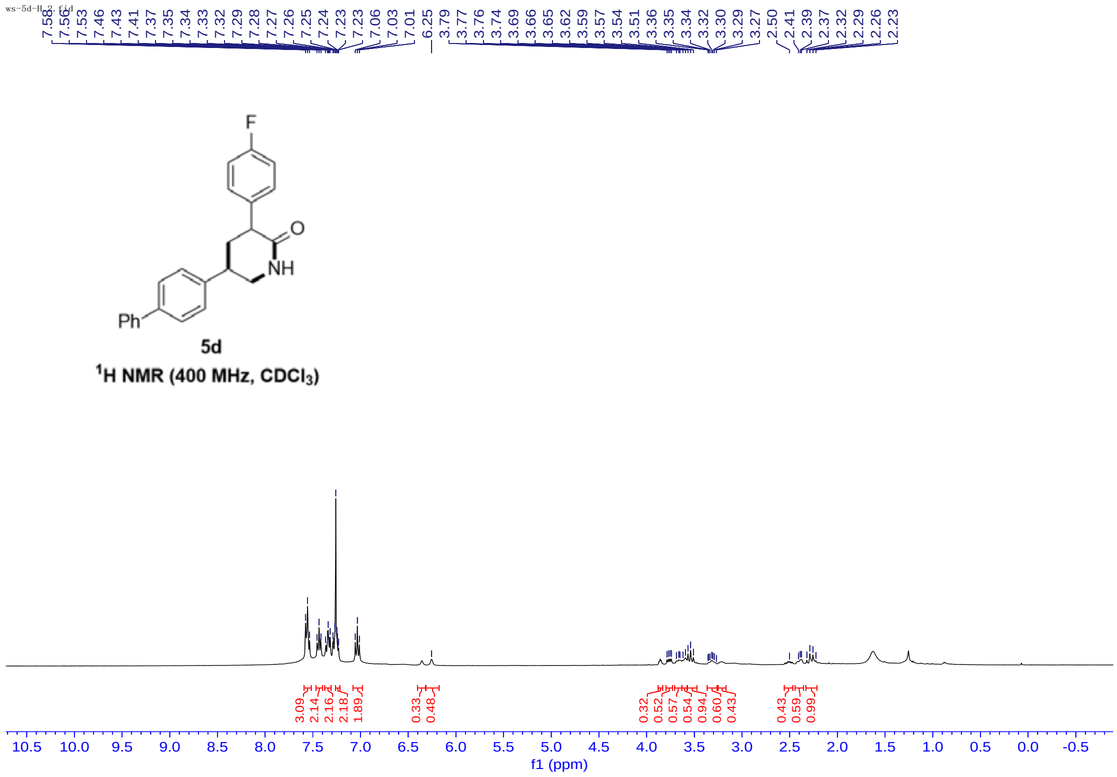

Supplementary Figure 154 ^{1}H NMR (400 MHz, 298K, $CDCl_3$) of **5d**

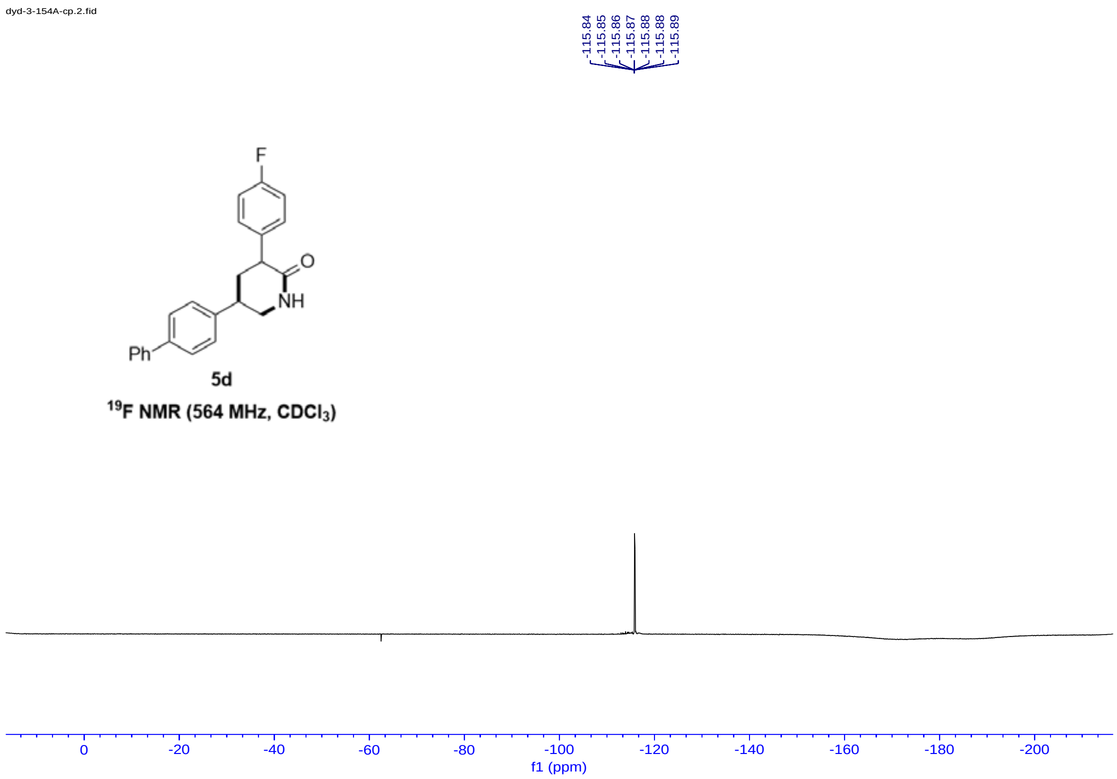

Supplementary Figure 155 ^{19}F NMR (564 MHz, 298K, $CDCl_3$) of **5d**

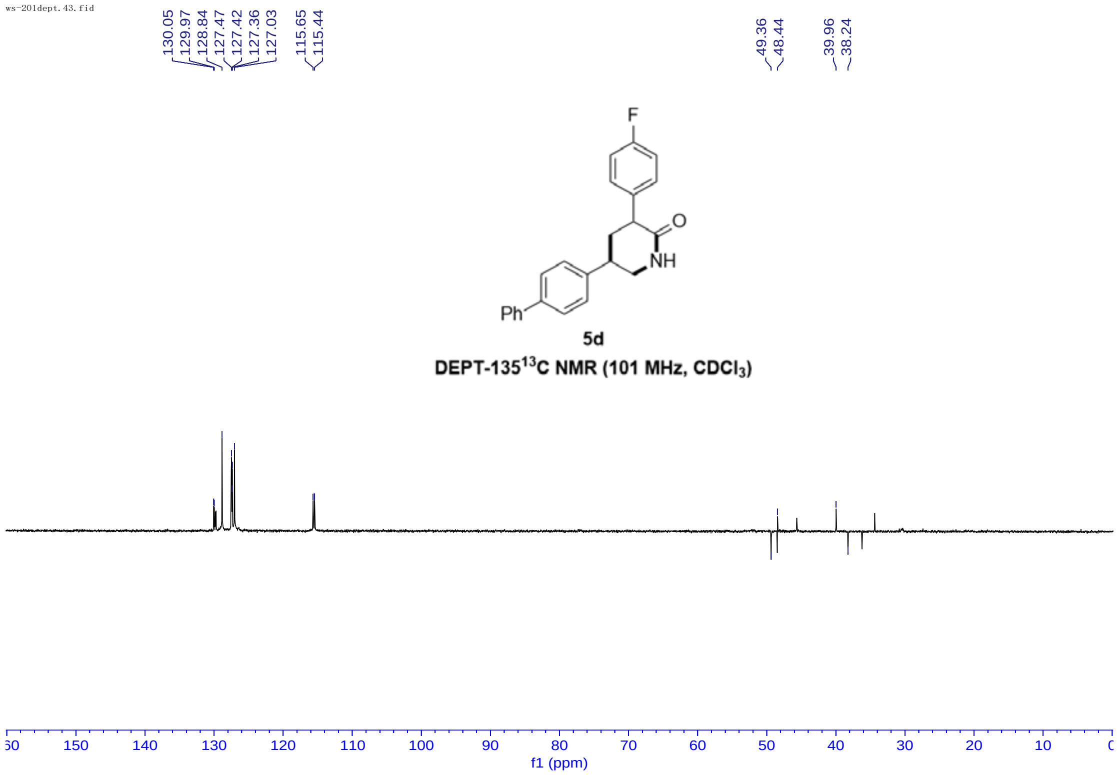

Supplementary Figure 156 DEPT-135 ^{13}C NMR (101 MHz, 298K, $CDCl_3$) of **5d**

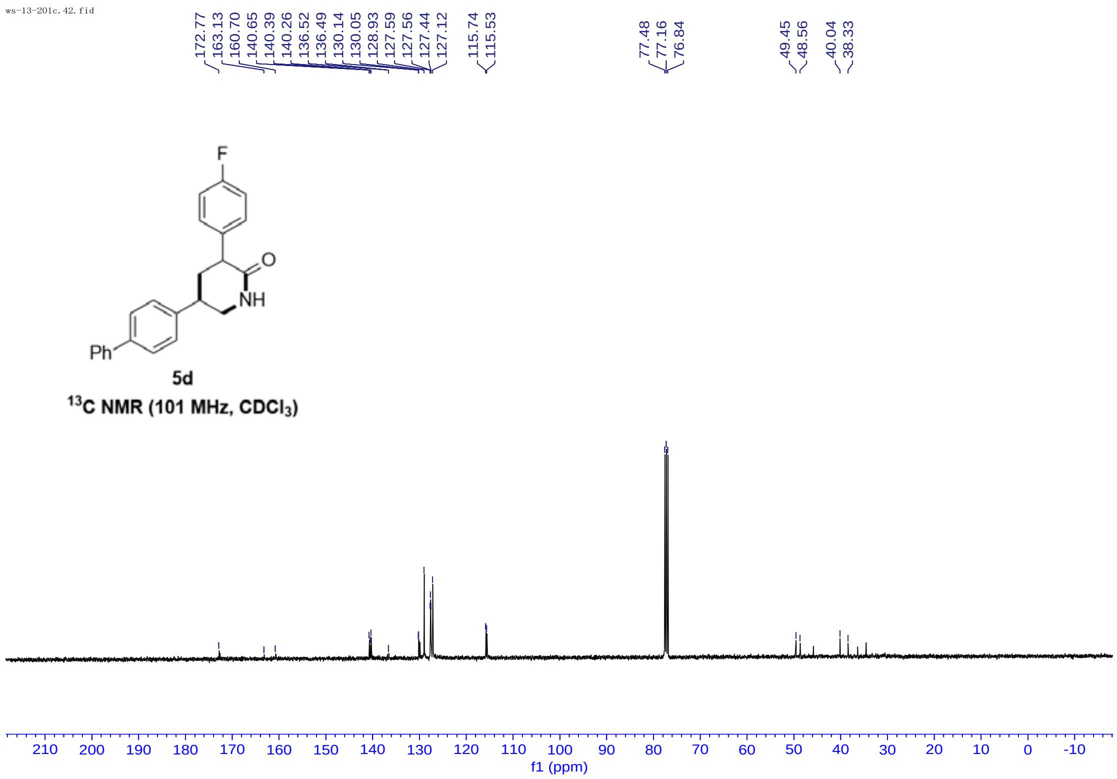

Supplementary Figure 157 ^{13}C NMR (101 MHz, 298K, $CDCl_3$) of **5d**

5-(4-bromophenyl)-3-(4-fluorophenyl)piperidin-2-one

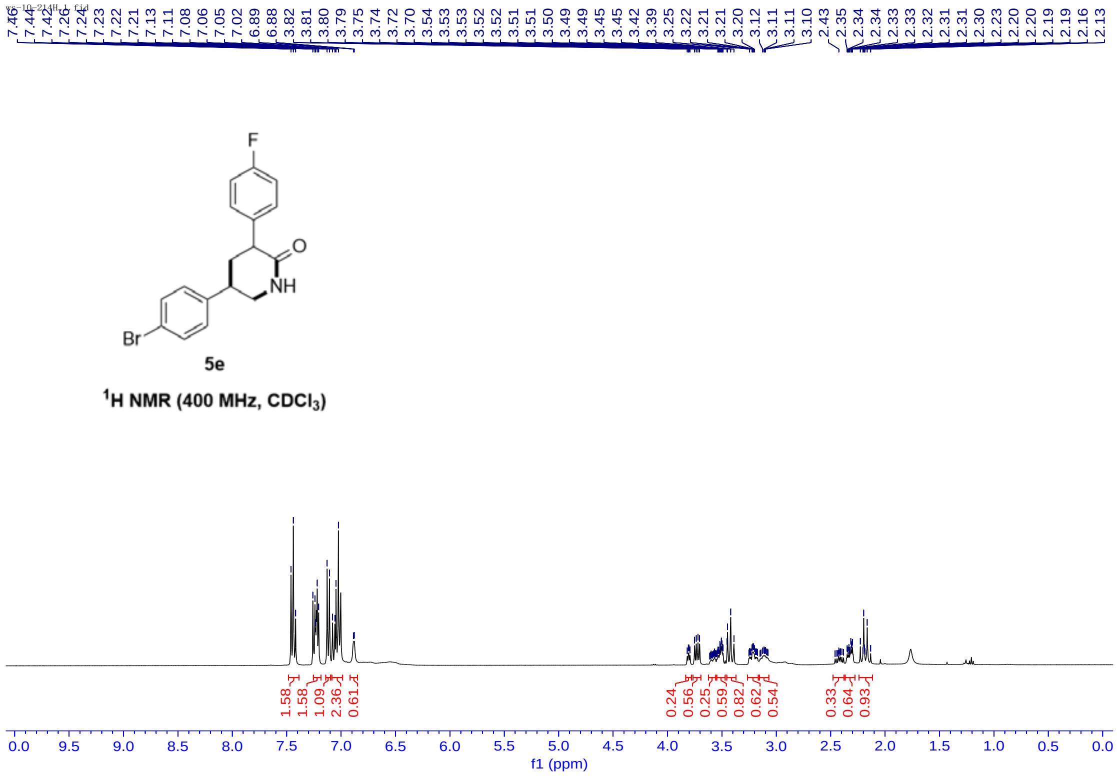

Supplementary Figure 158 ^{1}H NMR (400 MHz, 298K, $CDCl_3$) of **5e**

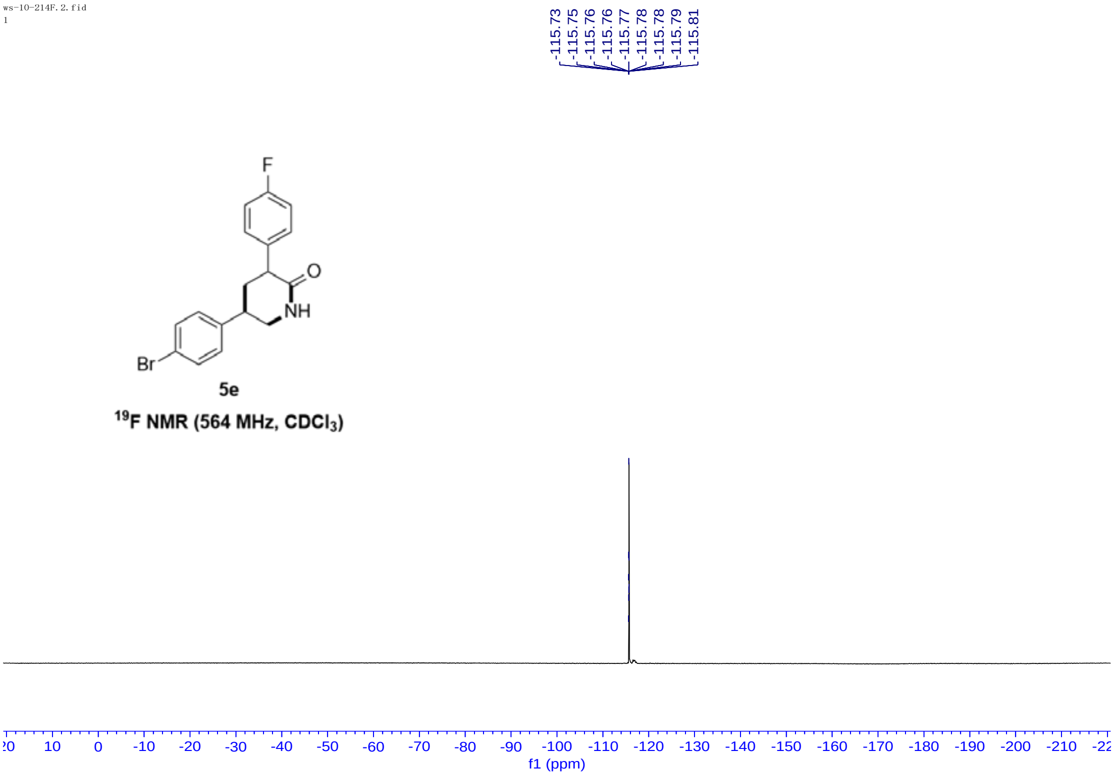

Supplementary Figure 159 ^{19}F NMR (564 MHz, 298K, $CDCl_3$) of **5e**

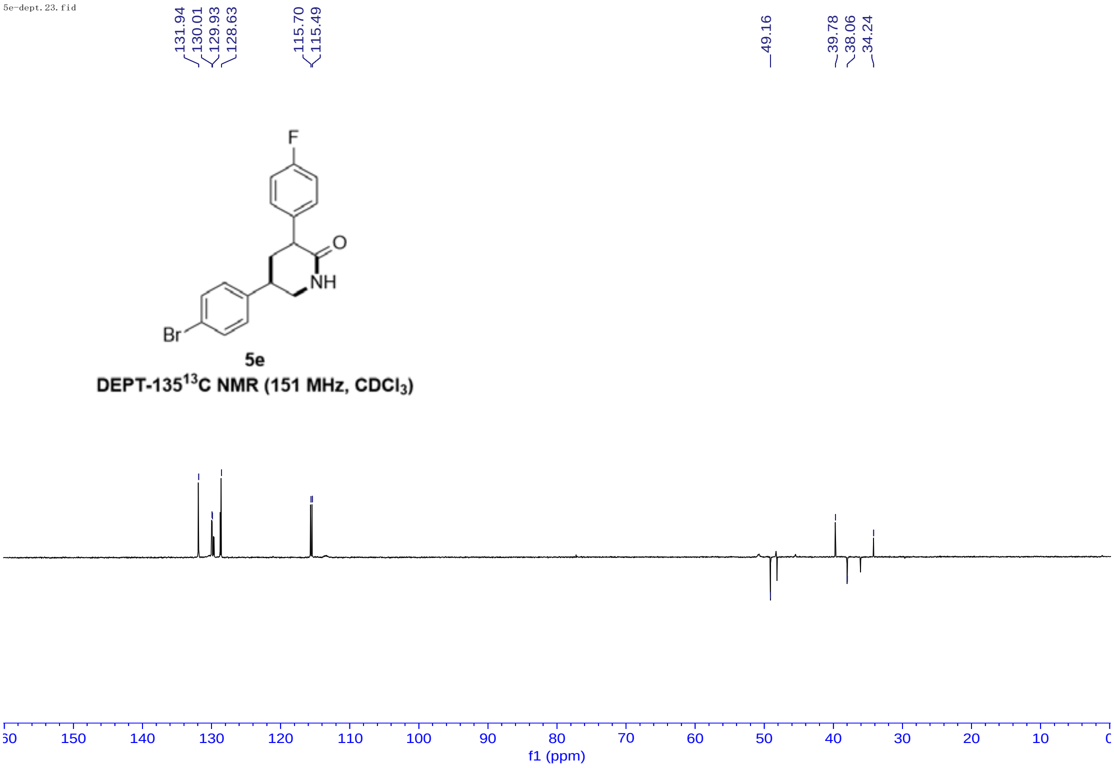

Supplementary Figure 160 DEPT-135 ^{13}C NMR (151 MHz, 298K, $CDCl_3$) of **5e**

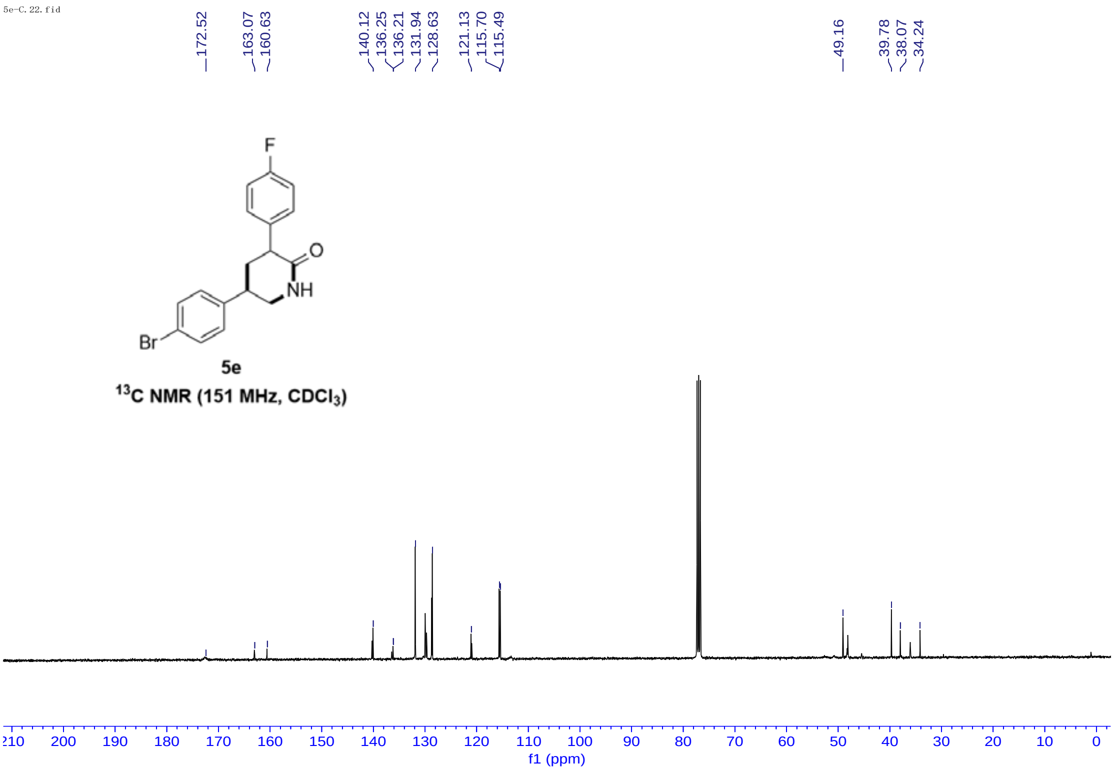

Supplementary Figure 161 ^{13}C NMR (151 MHz, 298K, $CDCl_3$) of **5e**

5-(4-chlorophenyl)-3-(4-fluorophenyl)piperidin-2-one

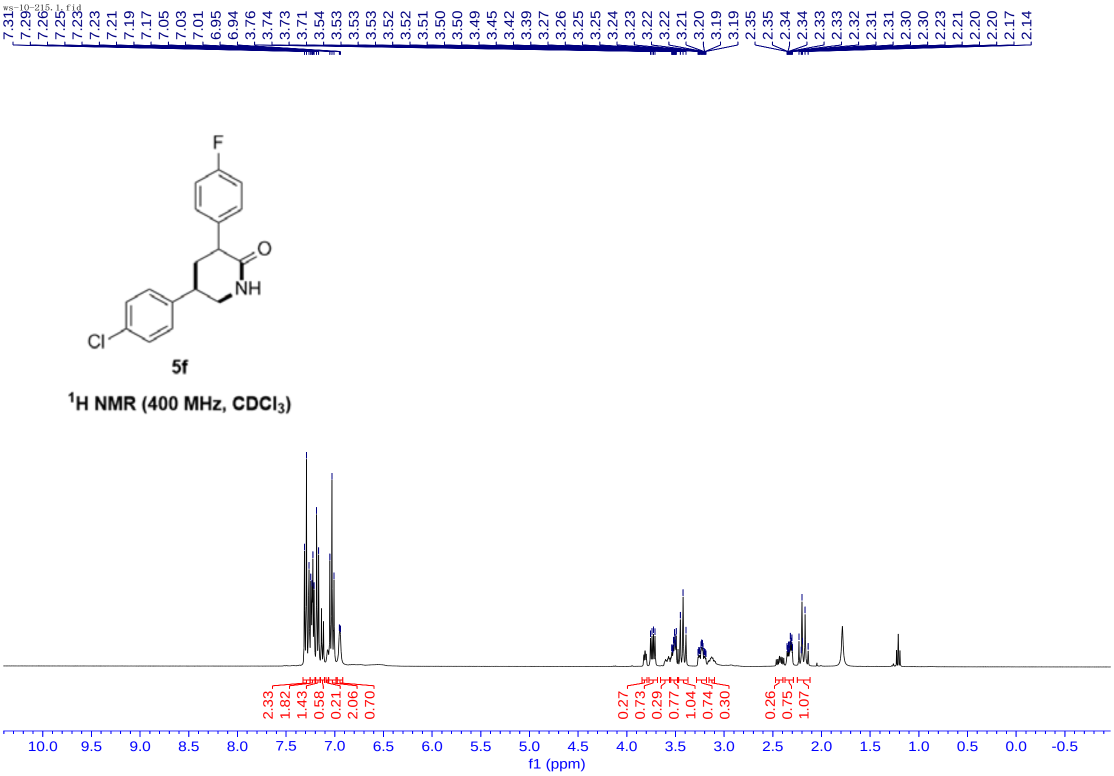

Supplementary Figure 162 ^{1}H NMR (400 MHz, 298K, $CDCl_3$) of **5f**

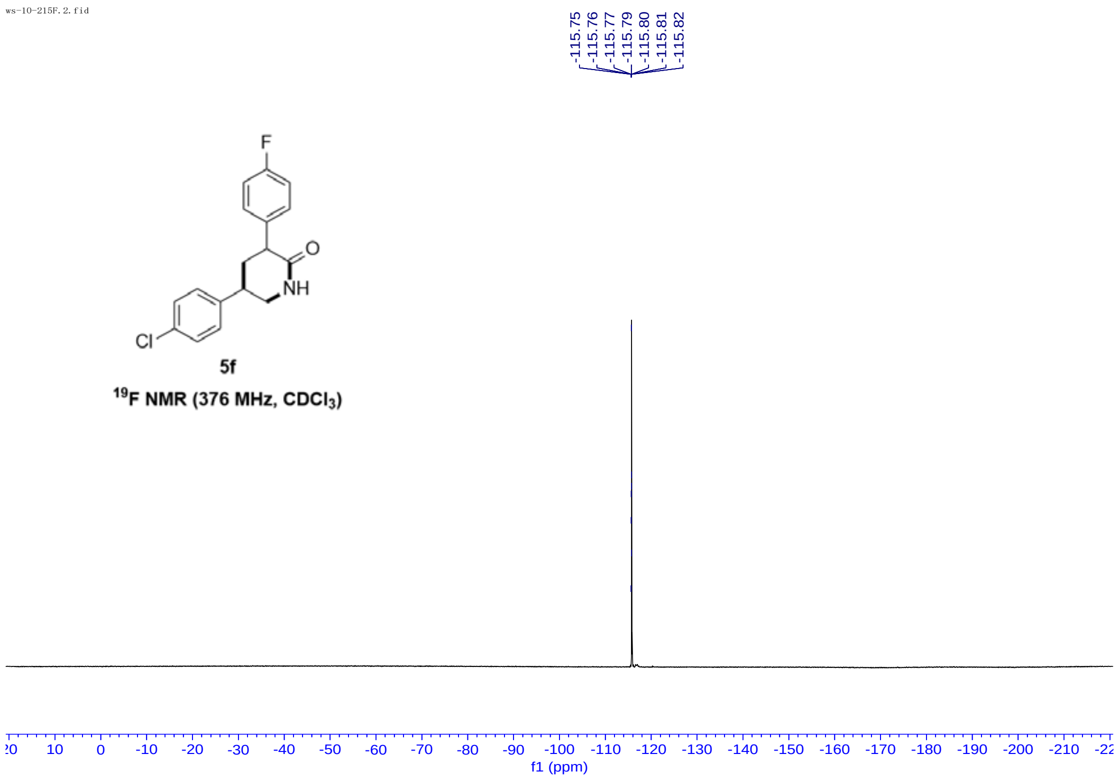

Supplementary Figure 163 ^{19}F NMR (376 MHz, 298K, $CDCl_3$) of **5f**

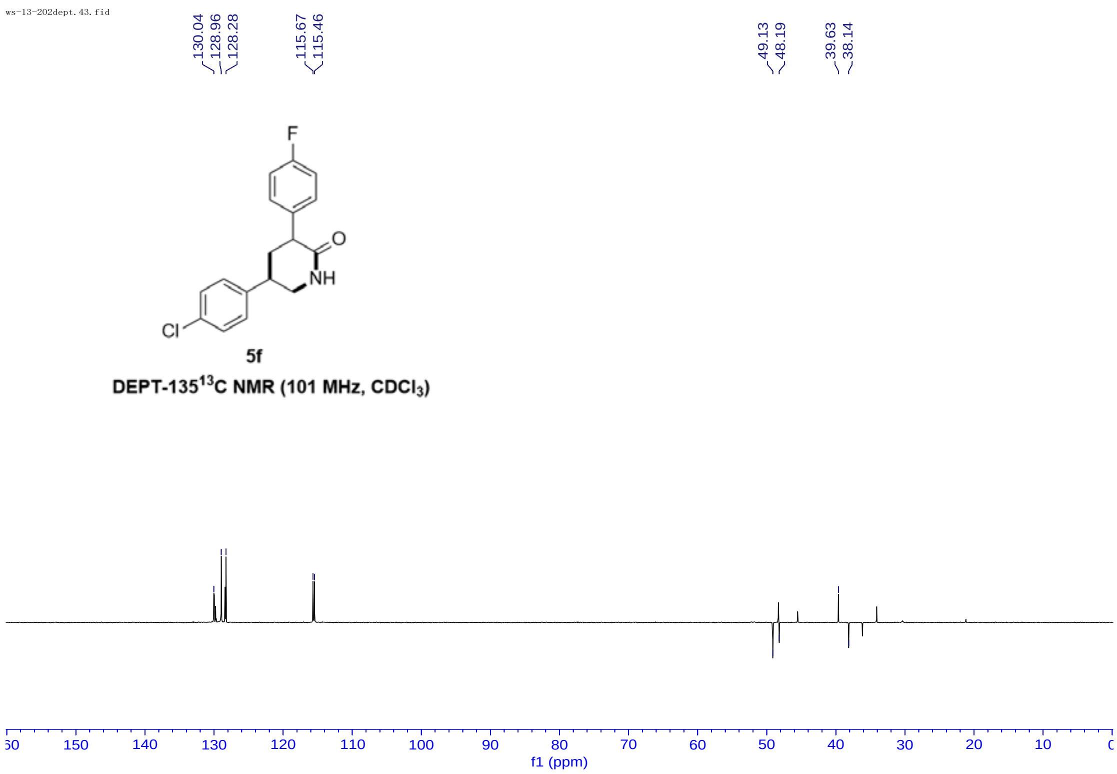

Supplementary Figure 164 DEPT-135 ^{13}C NMR (101 MHz, 298K, $CDCl_3$) of **5f**

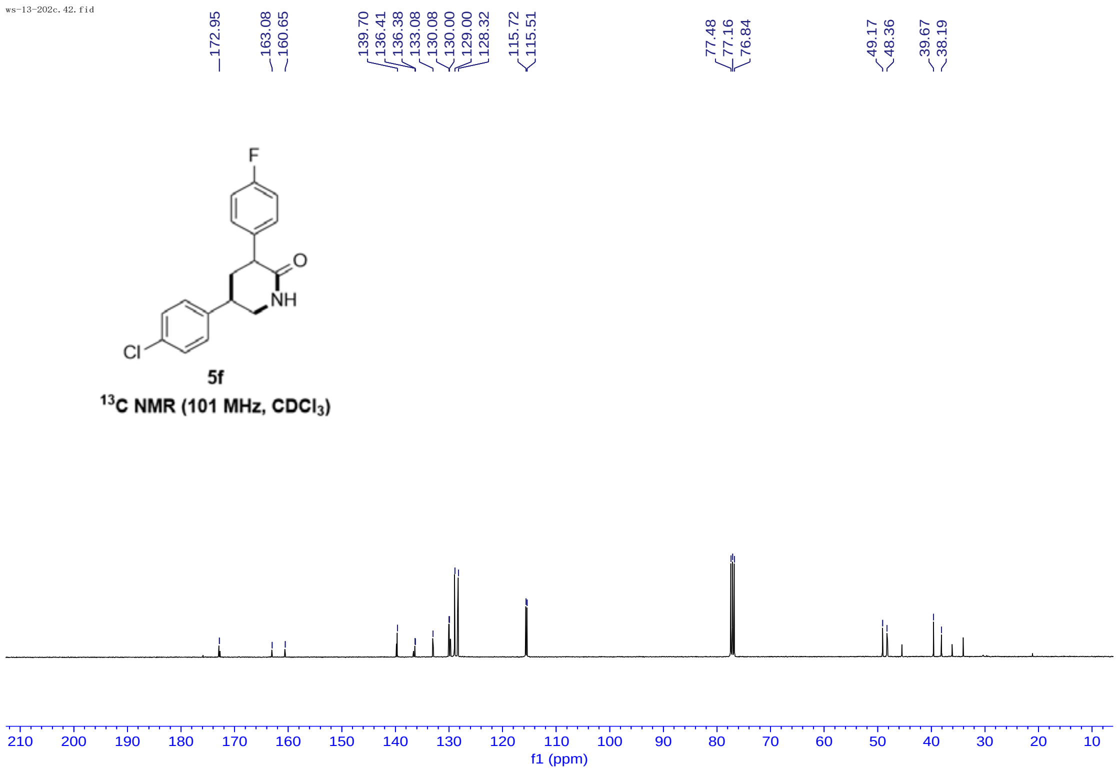

Supplementary Figure 165 ^{13}C NMR (101 MHz, 298K, $CDCl_3$) of **5f**

3,5-bis(4-fluorophenyl)piperidin-2-one

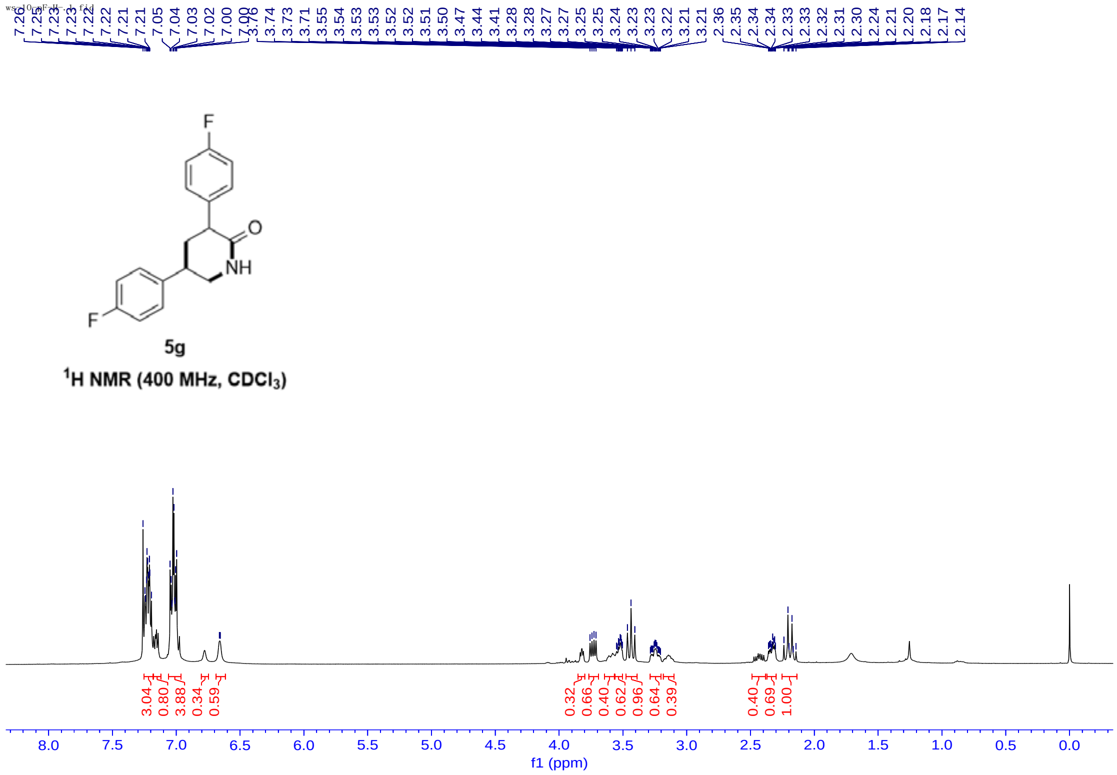

Supplementary Figure 166 ^{1}H NMR (400 MHz, 298K, $CDCl_3$) of **5g**

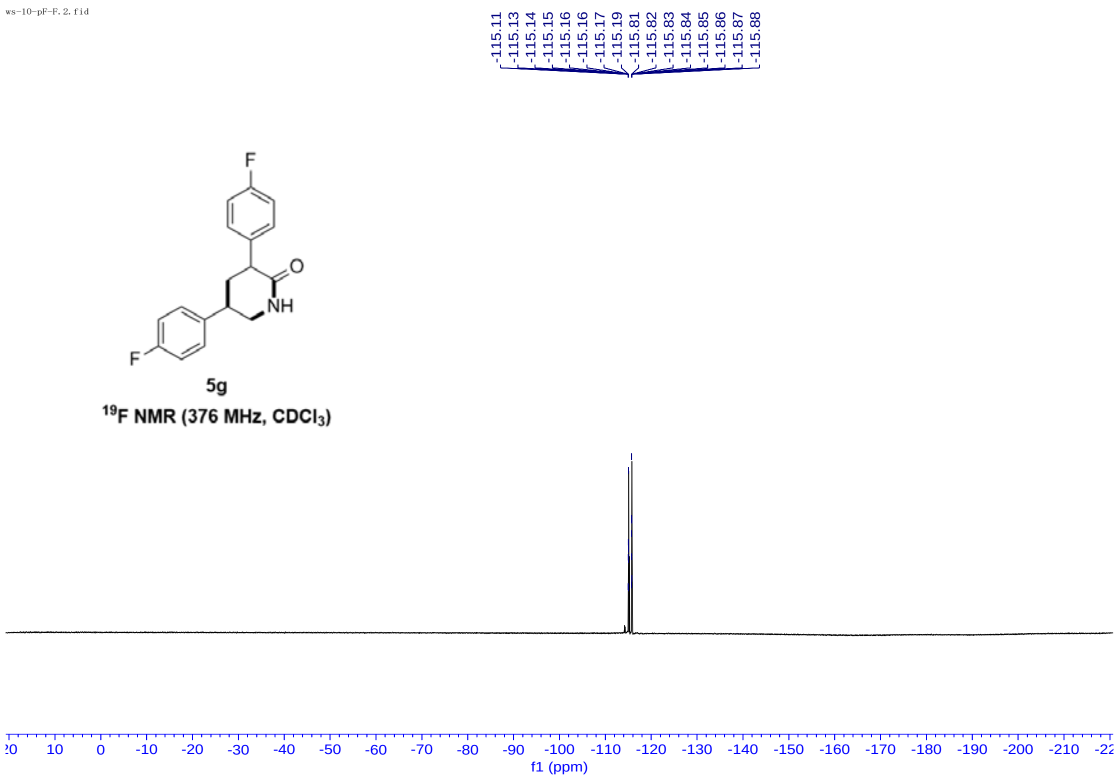

Supplementary Figure 167 ^{19}F NMR (376 MHz, 298K, $CDCl_3$) of **5g**

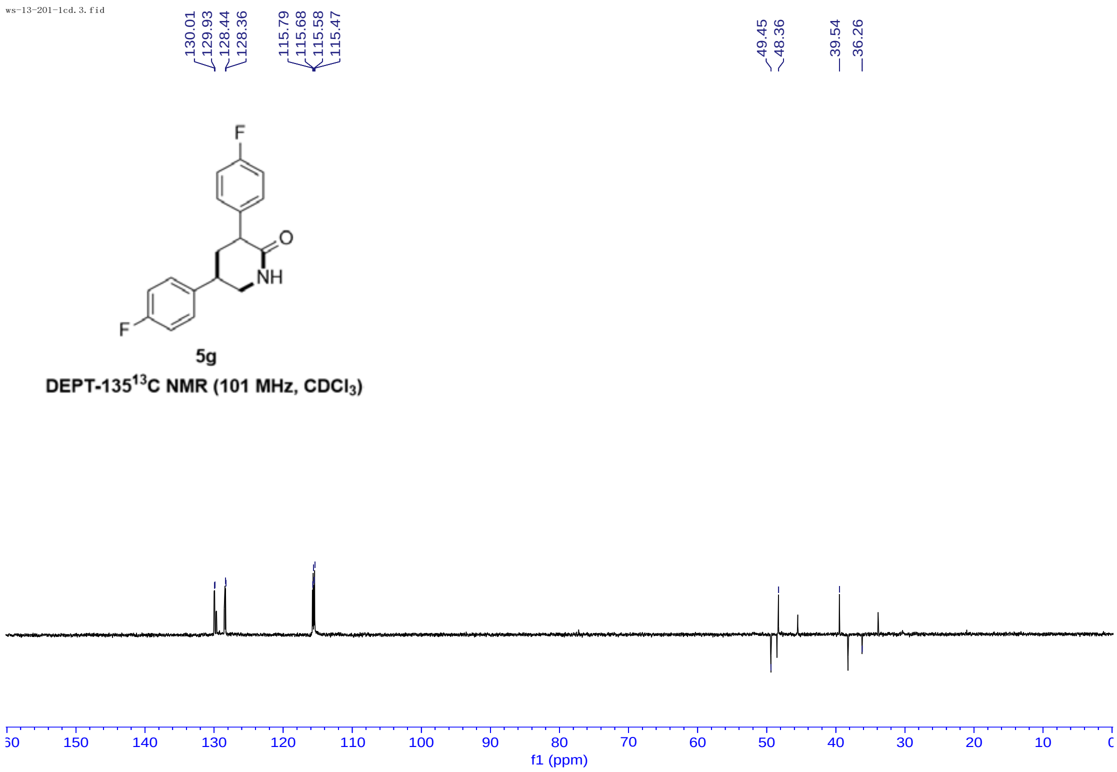

Supplementary Figure 168 DEPT-135 ^{13}C NMR (101 MHz, 298K, $CDCl_3$) of **5g**

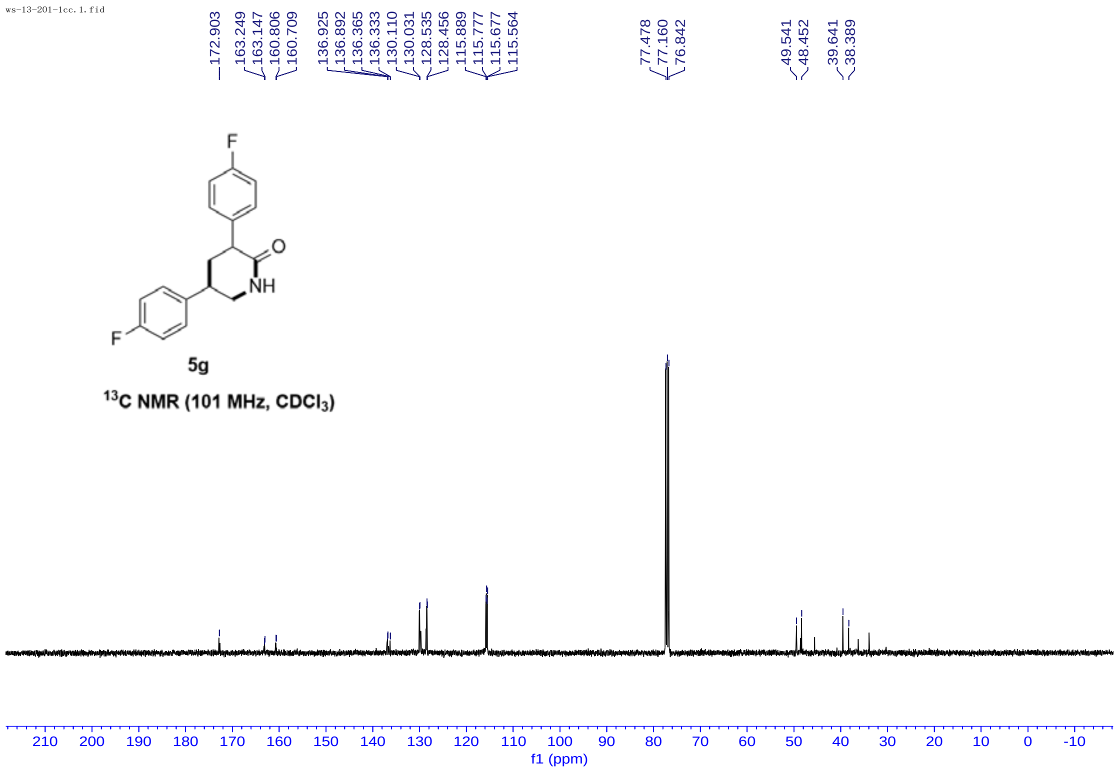

Supplementary Figure 169 ^{13}C NMR (101 MHz, 298K, $CDCl_3$) of **5g**

3-(4-fluorophenyl)-5-(*p*-tolyl)piperidin-2-one

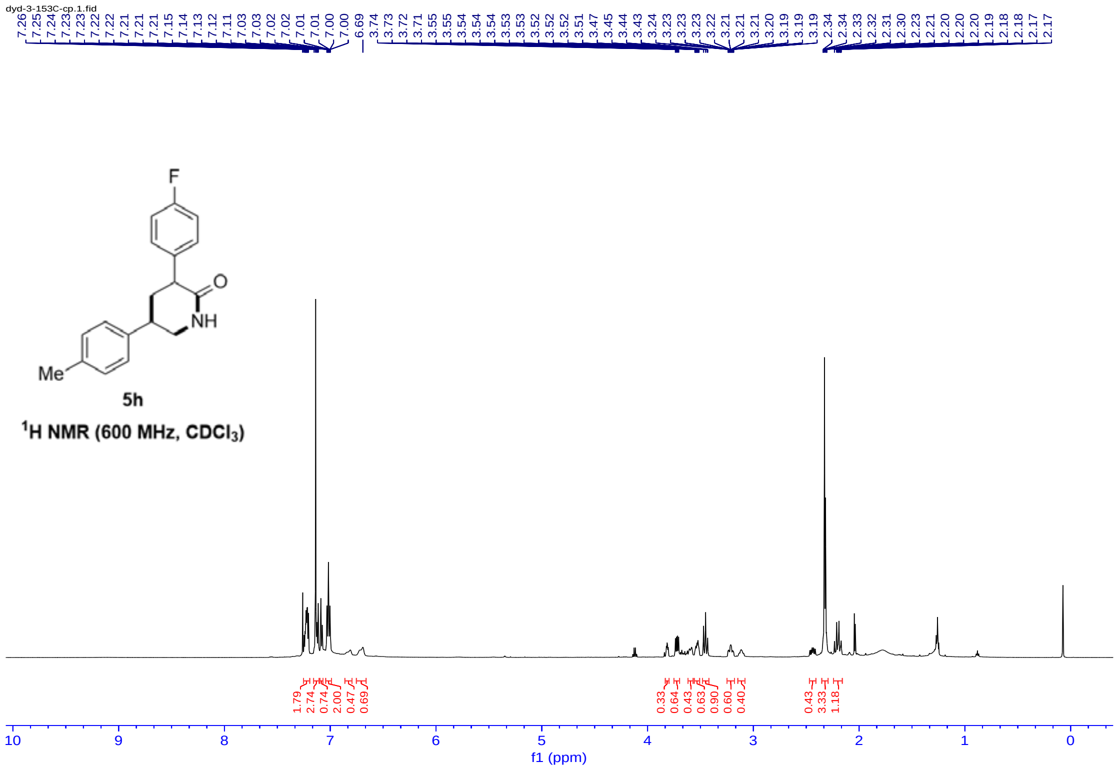

Supplementary Figure 170 ^{1}H NMR (600 MHz, 298K, $CDCl_3$) of **5h**

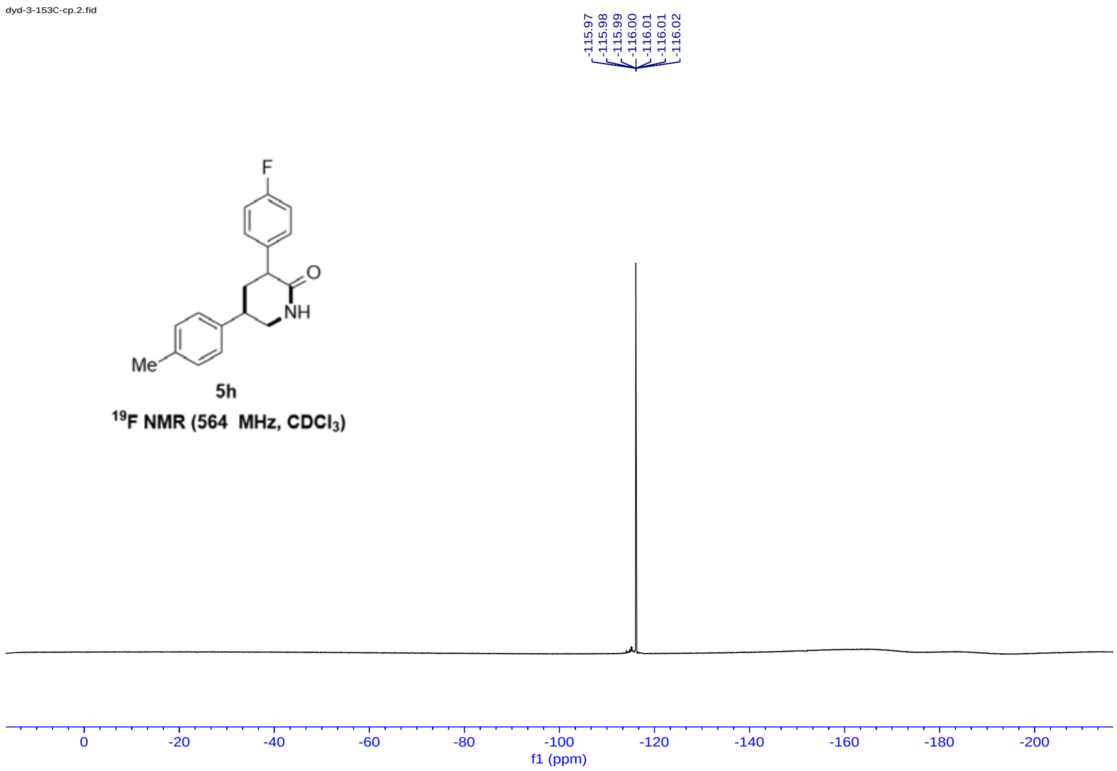

Supplementary Figure 171 ^{19}F NMR (564 MHz, 298K, $CDCl_3$) of **5h**

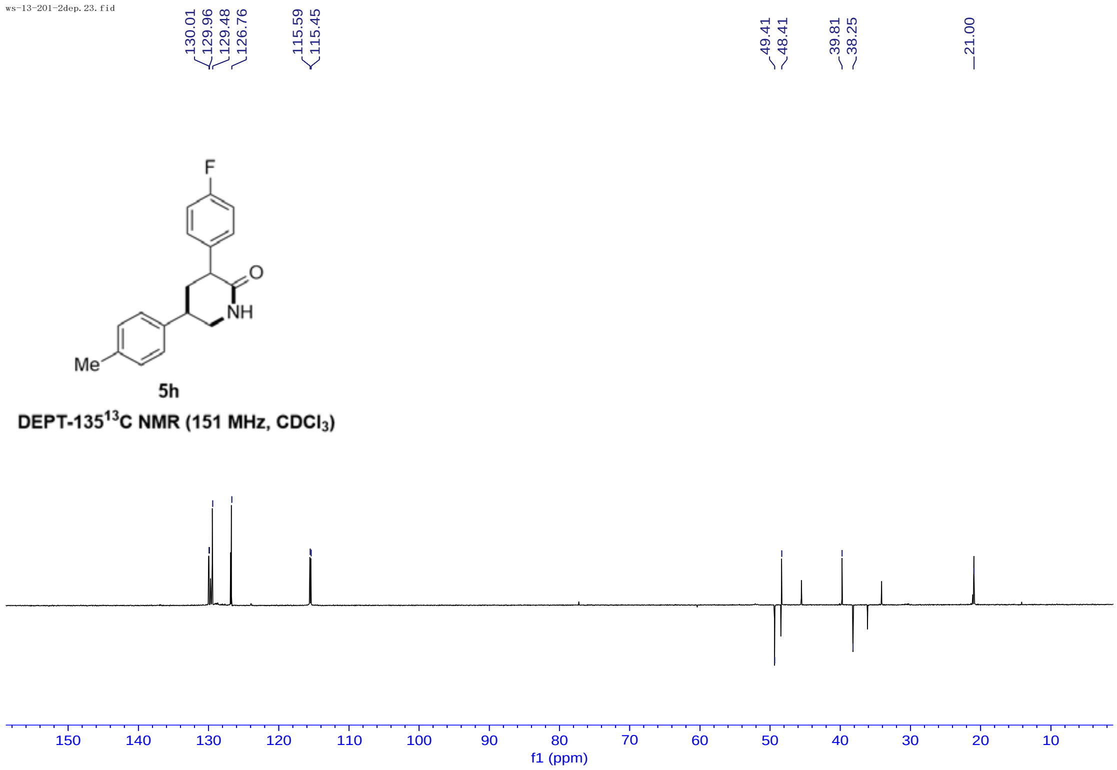

Supplementary Figure 172 DEPT-135 ^{13}C NMR (151 MHz, 298K, $CDCl_3$) of **5h**

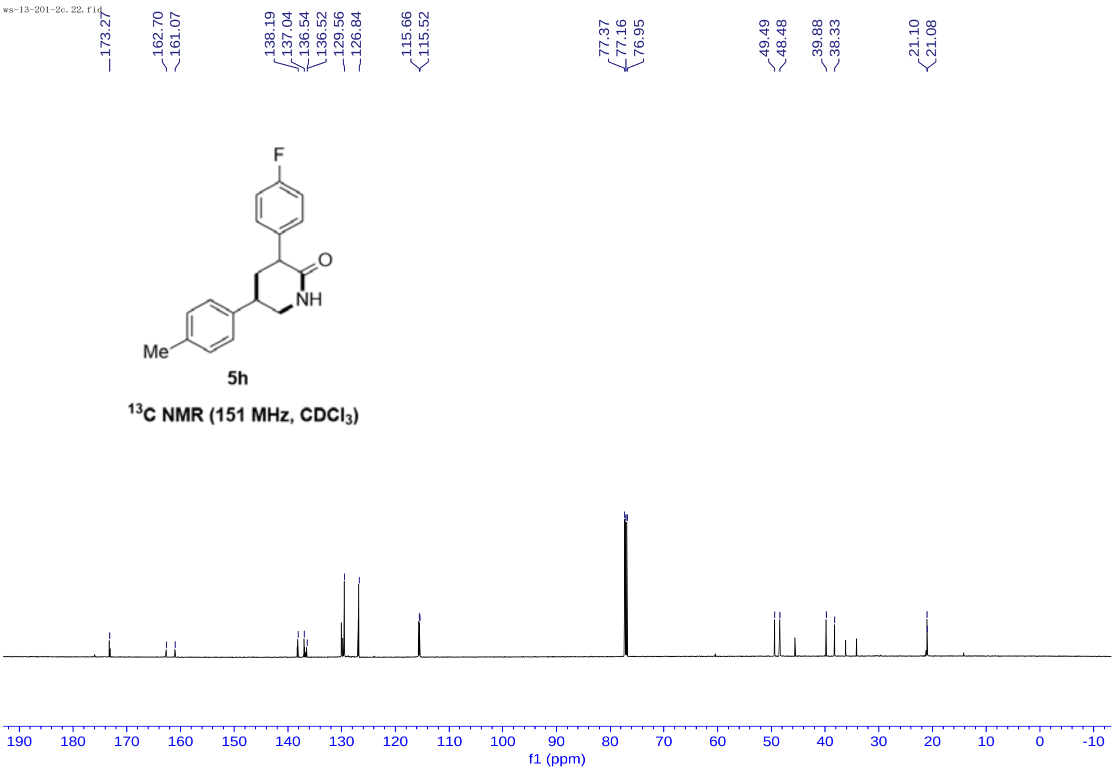

Supplementary Figure 173 ^{13}C NMR (151 MHz, 298K, $CDCl_3$) of **5h**

3-(4-fluorophenyl)-5-(4-isopropylphenyl)piperidin-2-one

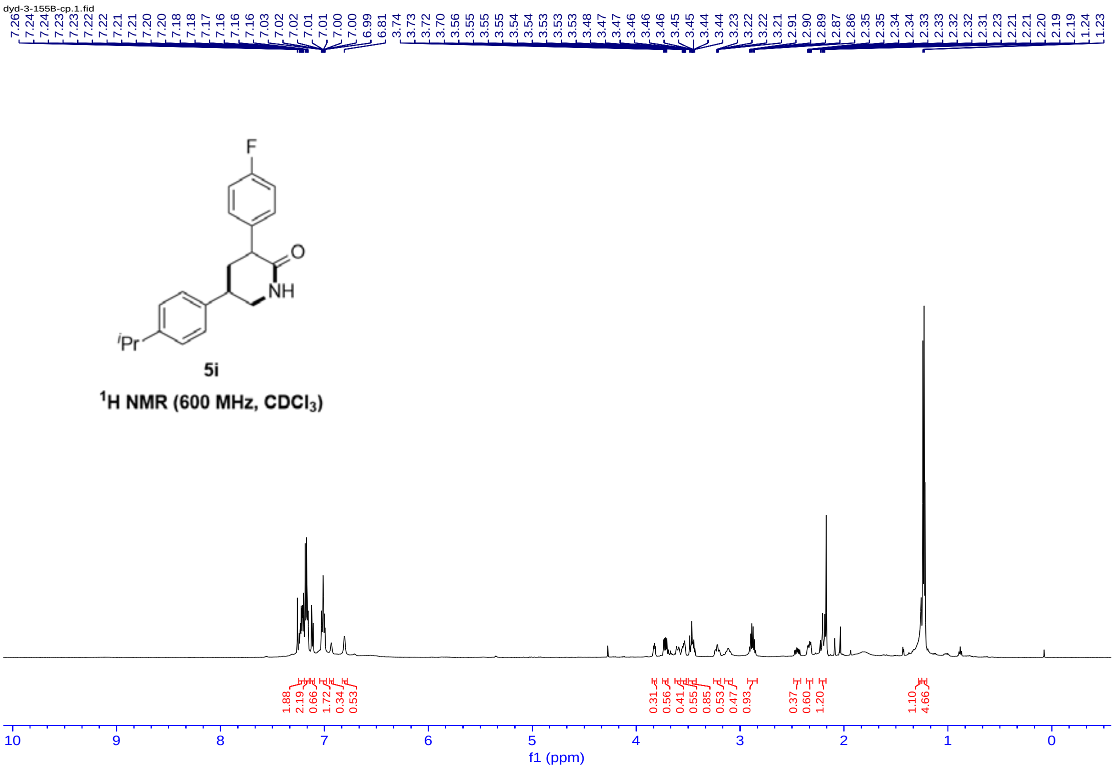

Supplementary Figure 174 ^{1}H NMR (600 MHz, 298K, $CDCl_3$) of **5i**

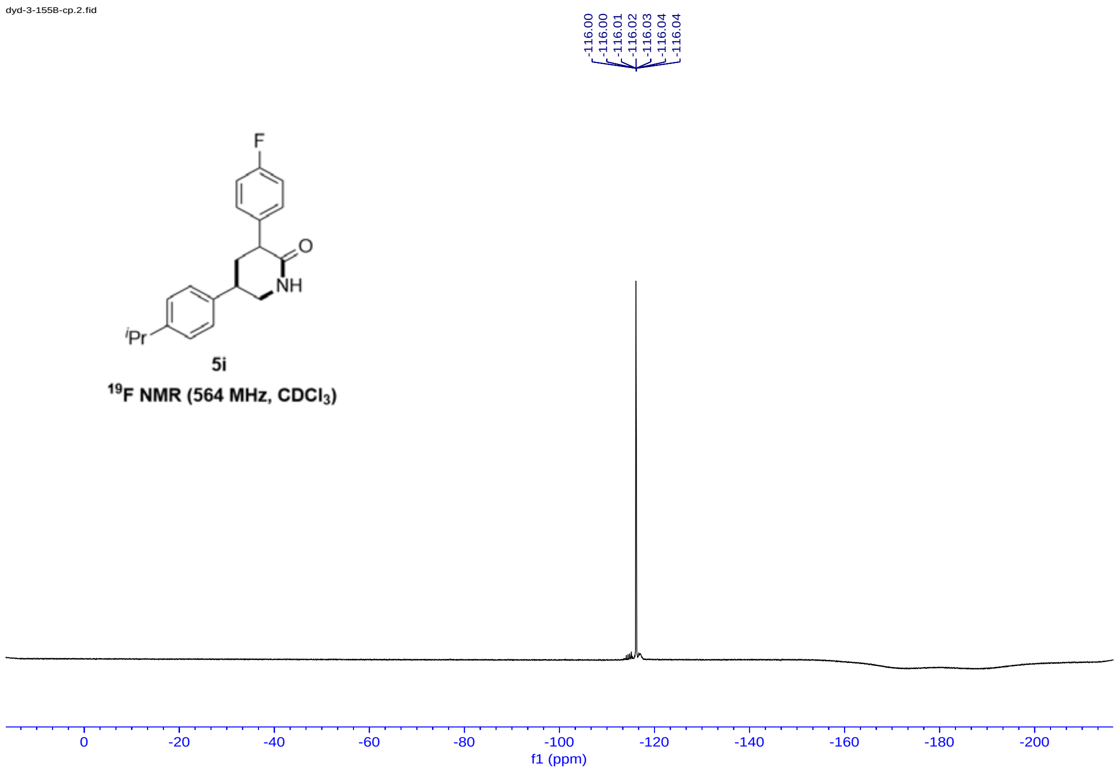

Supplementary Figure 175 ^{19}F NMR (564 MHz, 298K, $CDCl_3$) of **5i**

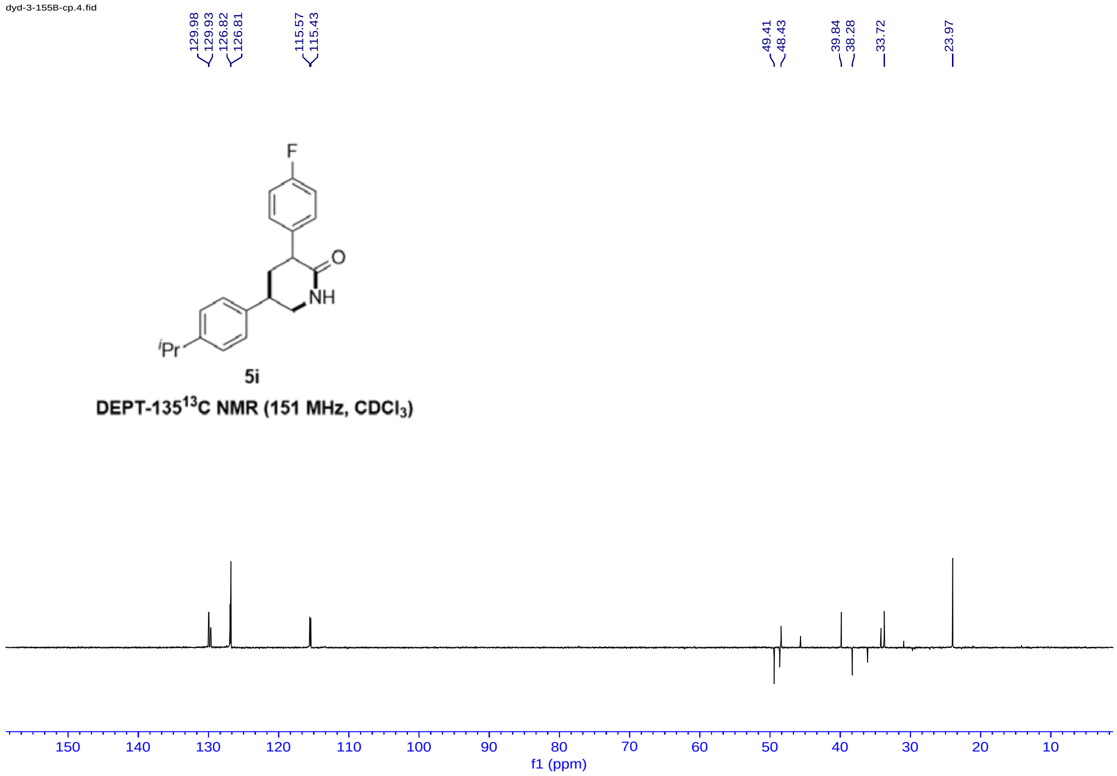

Supplementary Figure 176 DEPT-135 ^{13}C NMR (151 MHz, 298K, $CDCl_3$) of **5i**

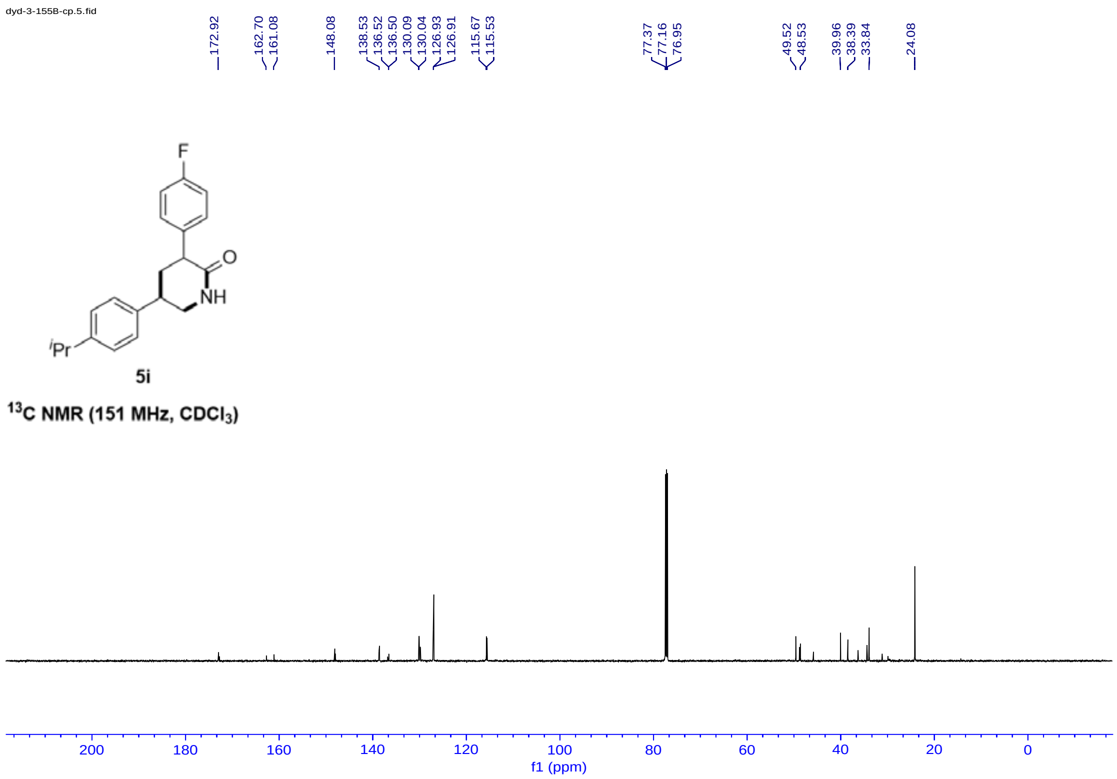

Supplementary Figure 177 ^{13}C NMR (151 MHz, 298K, $CDCl_3$) of **5i**

5-(4-(*tert*-butyl)phenyl)-3-(4-fluorophenyl)piperidin-2-one

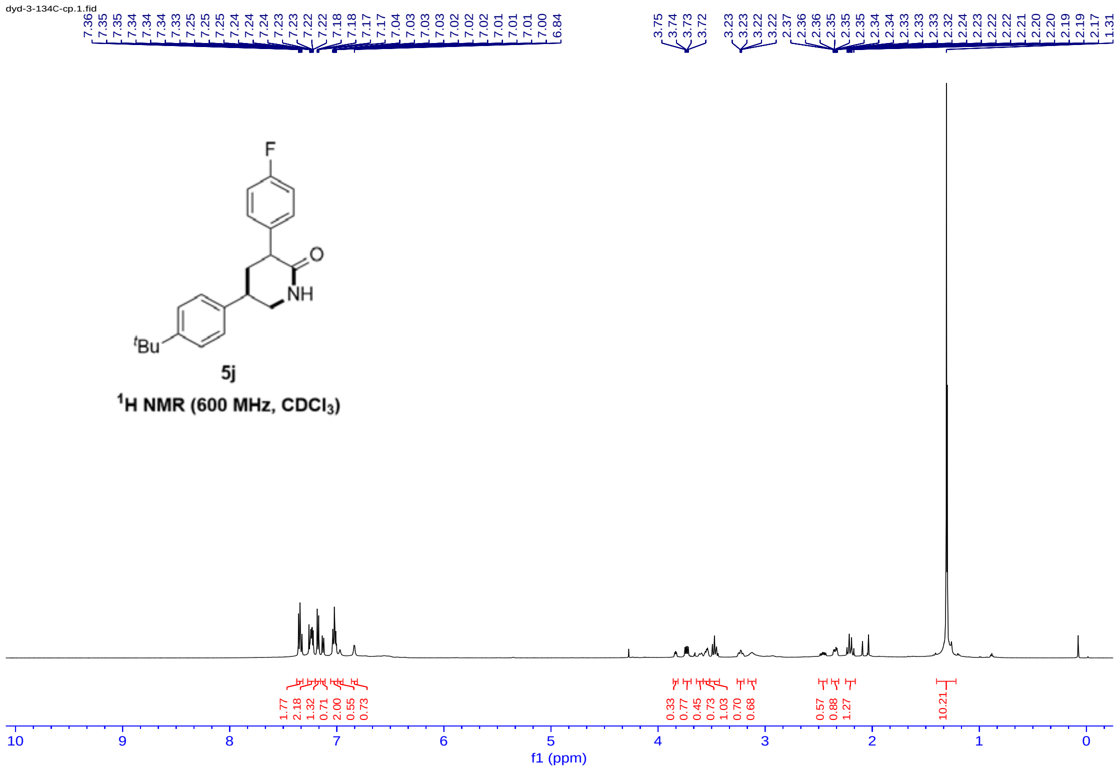

Supplementary Figure 178 ^{1}H NMR (600 MHz, 298K, $CDCl_3$) of **5j**

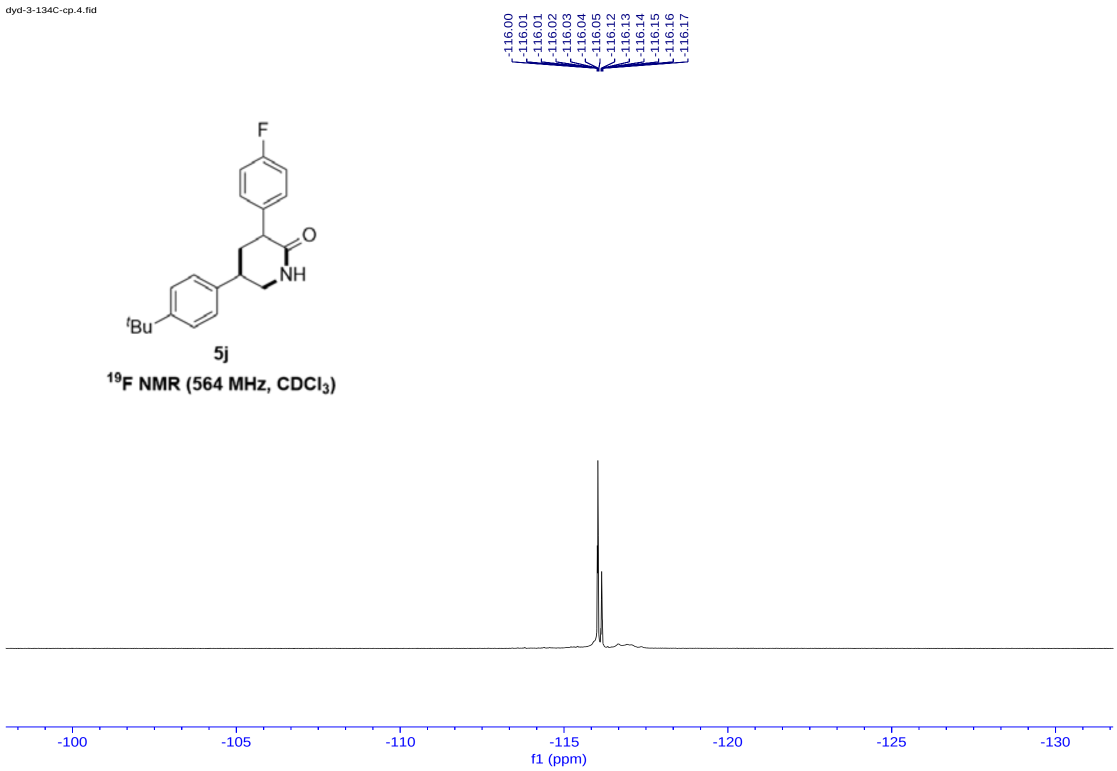

Supplementary Figure 179 ^{19}F NMR (564 MHz, 298K, $CDCl_3$) of **5j**

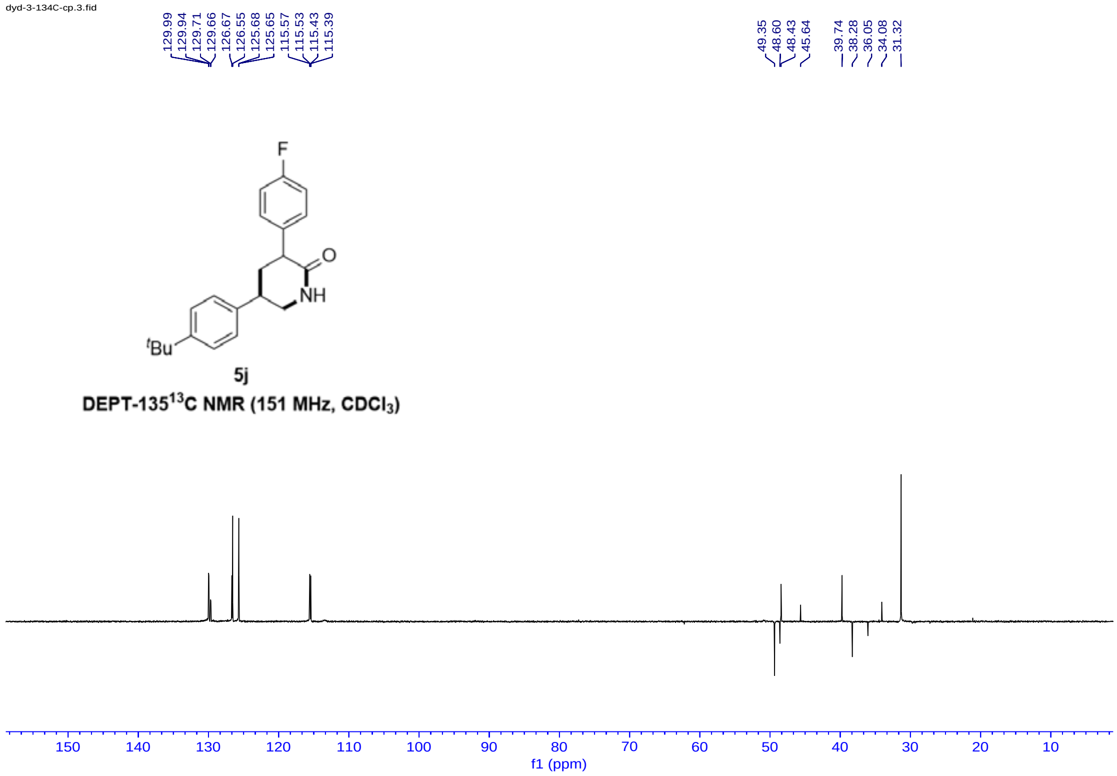

Supplementary Figure 180 DEPT-135 ^{13}C NMR (151 MHz, 298K, $CDCl_3$) of **5j**

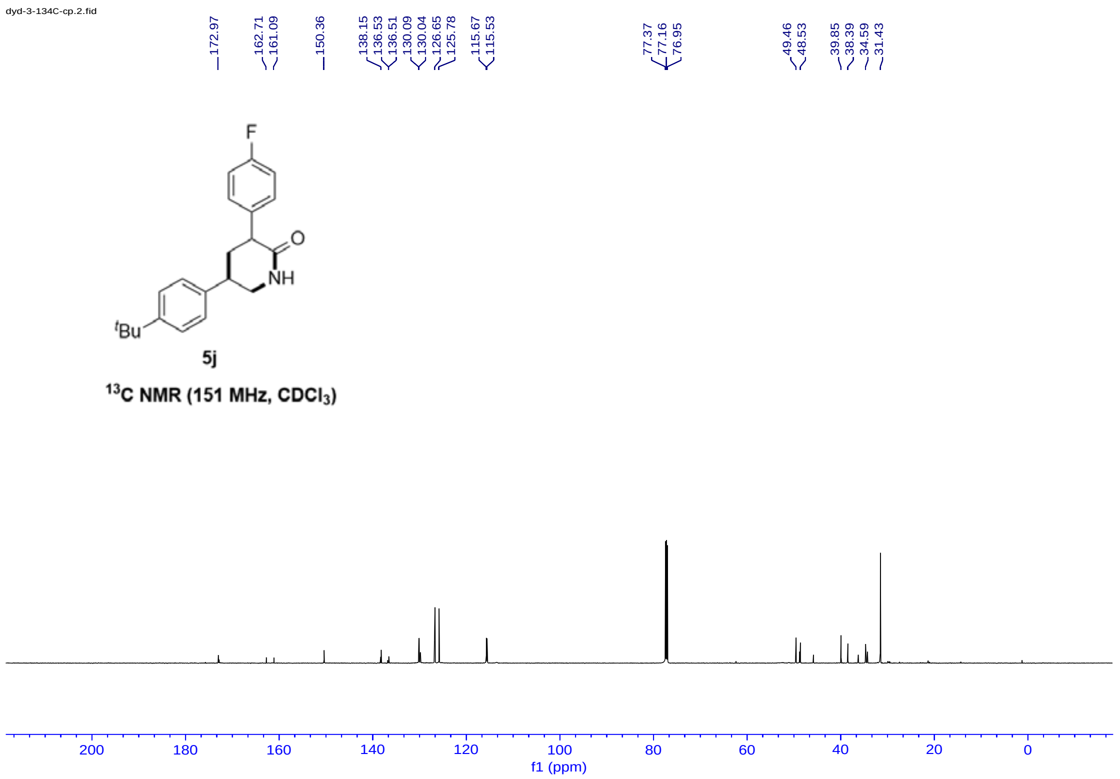

Supplementary Figure 181 ^{13}C NMR (151 MHz, 298K, $CDCl_3$) of **5j**

3-(4-fluorophenyl)-5-(4-methoxyphenyl)piperidin-2-one

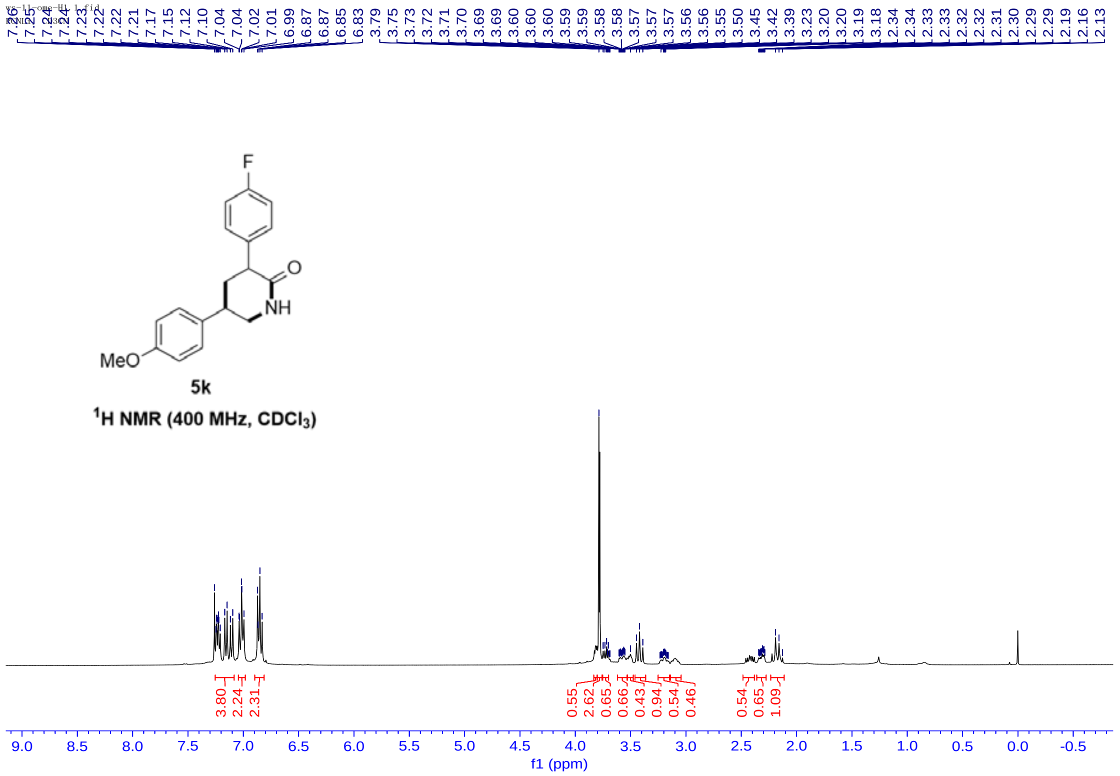

Supplementary Figure 182 ^{1}H NMR (400 MHz, 298K, $CDCl_3$) of **5k**

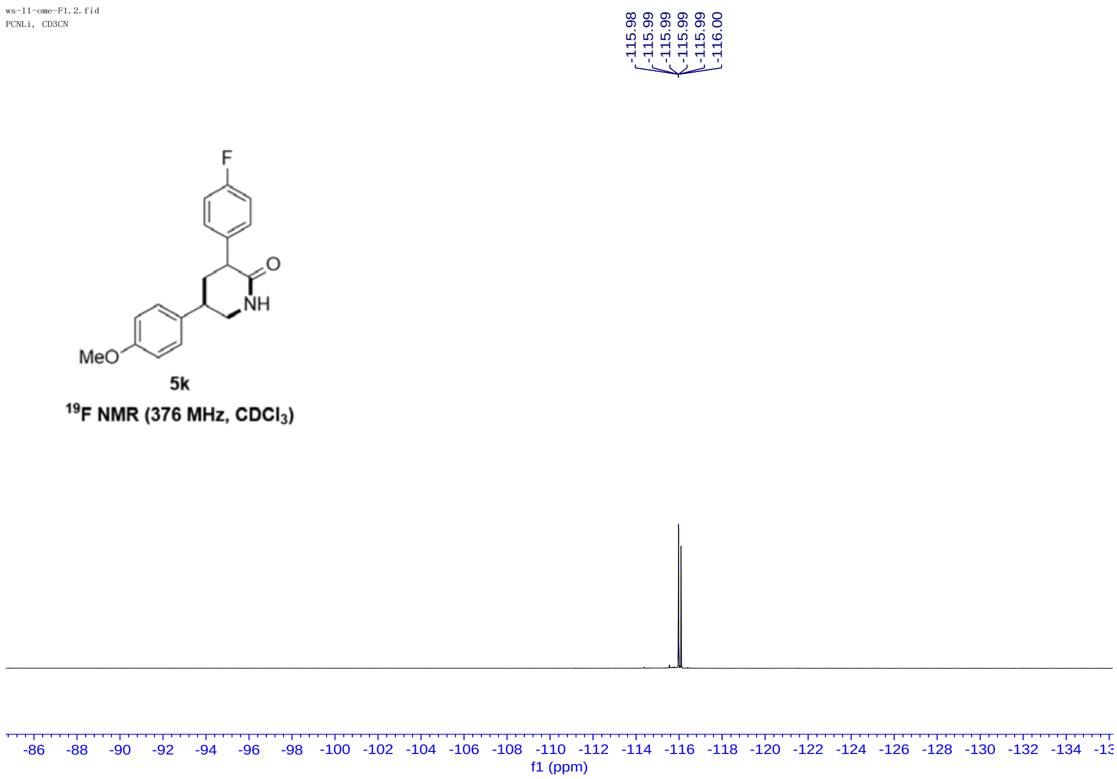

Supplementary Figure 183 ^{19}F NMR (376 MHz, 298K, $CDCl_3$) of **5k**

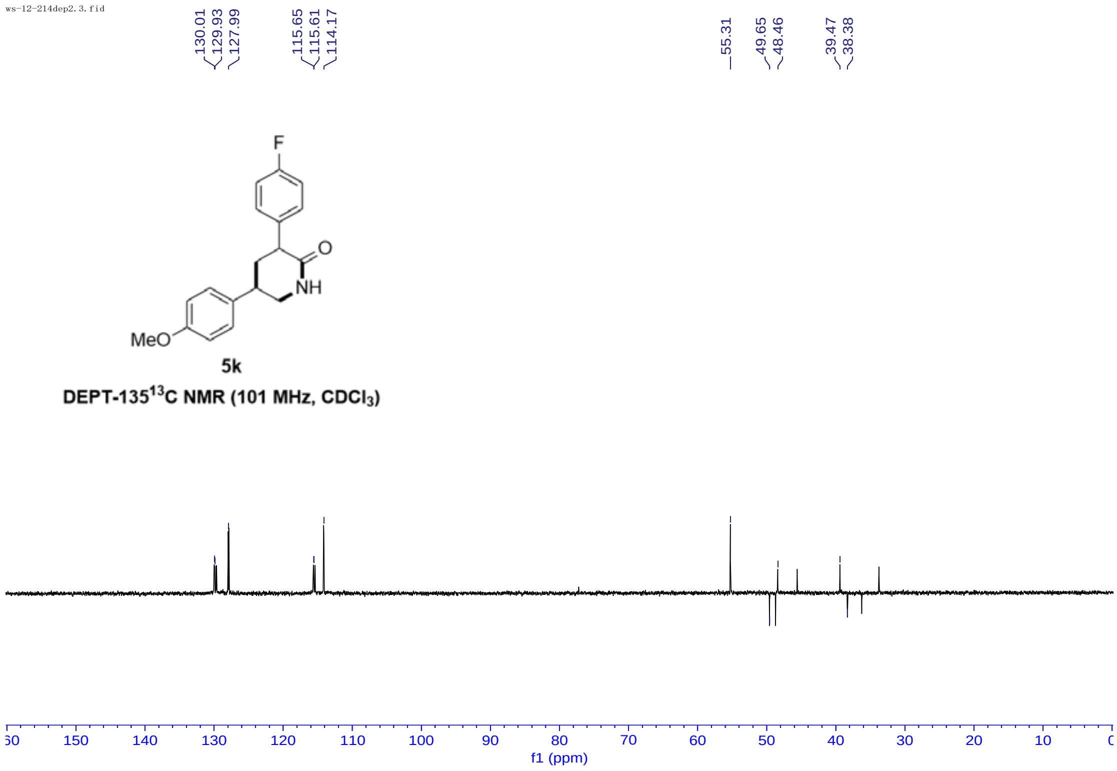

Supplementary Figure 184 DEPT-135 ^{13}C NMR (101 MHz, 298K, $CDCl_3$) of **5k**

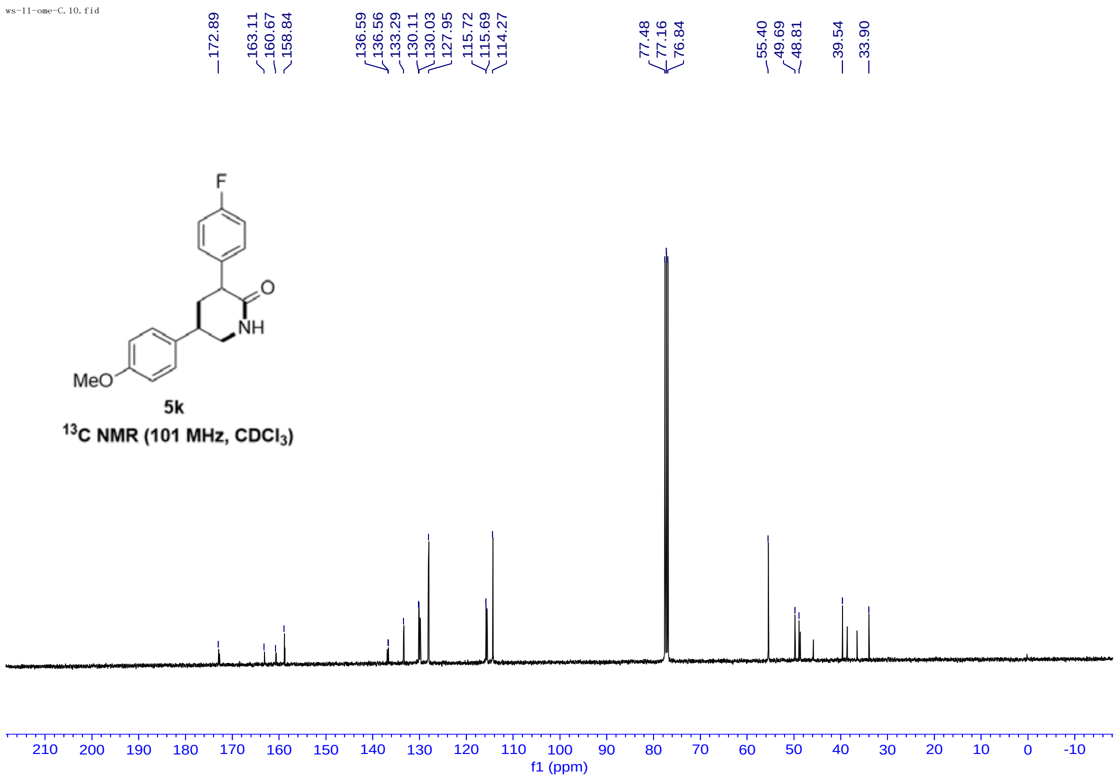

Supplementary Figure 185 ^{13}C NMR (101 MHz, 298K, $CDCl_3$) of **5k**

3-(4-fluorophenyl)-5-(*o*-tolyl)piperidin-2-one

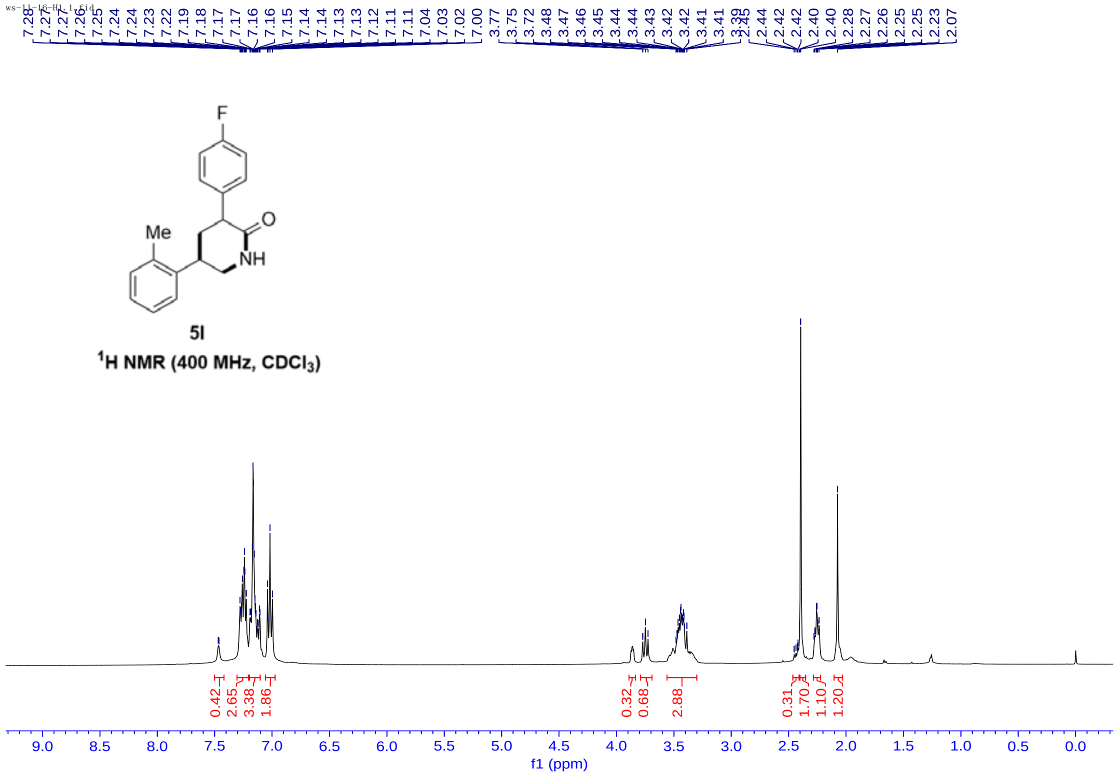

Supplementary Figure 186 ^{1}H NMR (400 MHz, 298K, $CDCl_3$) of **5l**

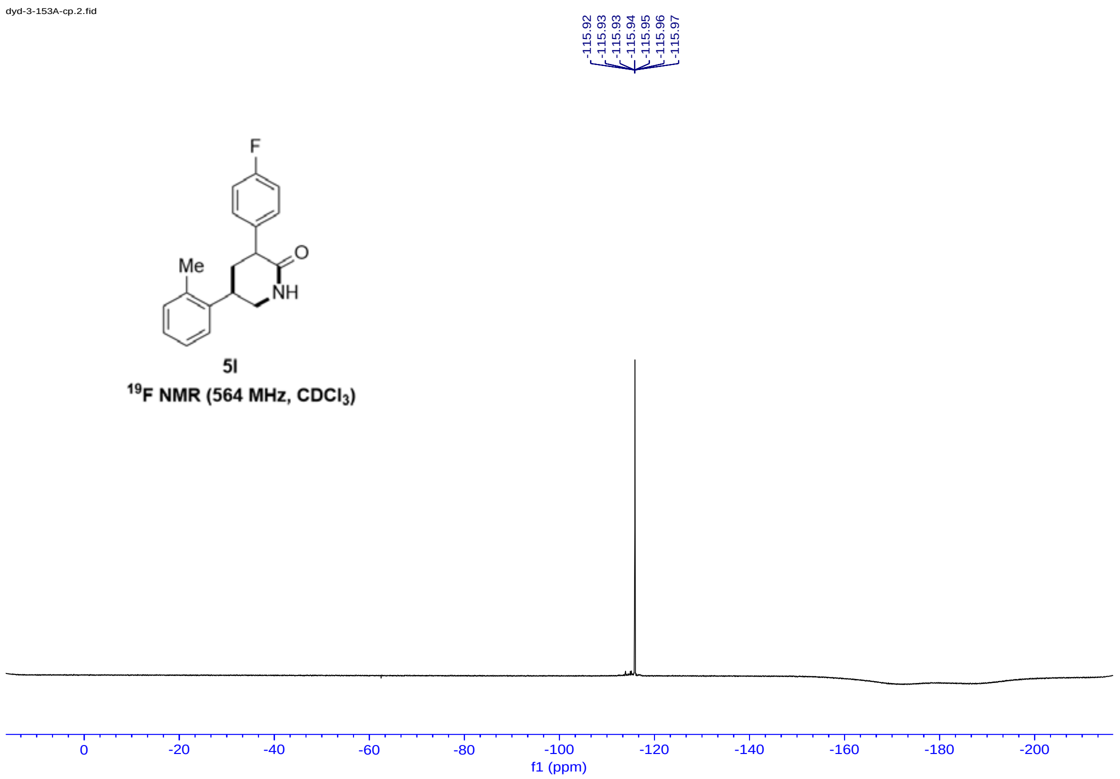

Supplementary Figure 187 ^{19}F NMR (564 MHz, 298K, $CDCl_3$) of **5l**

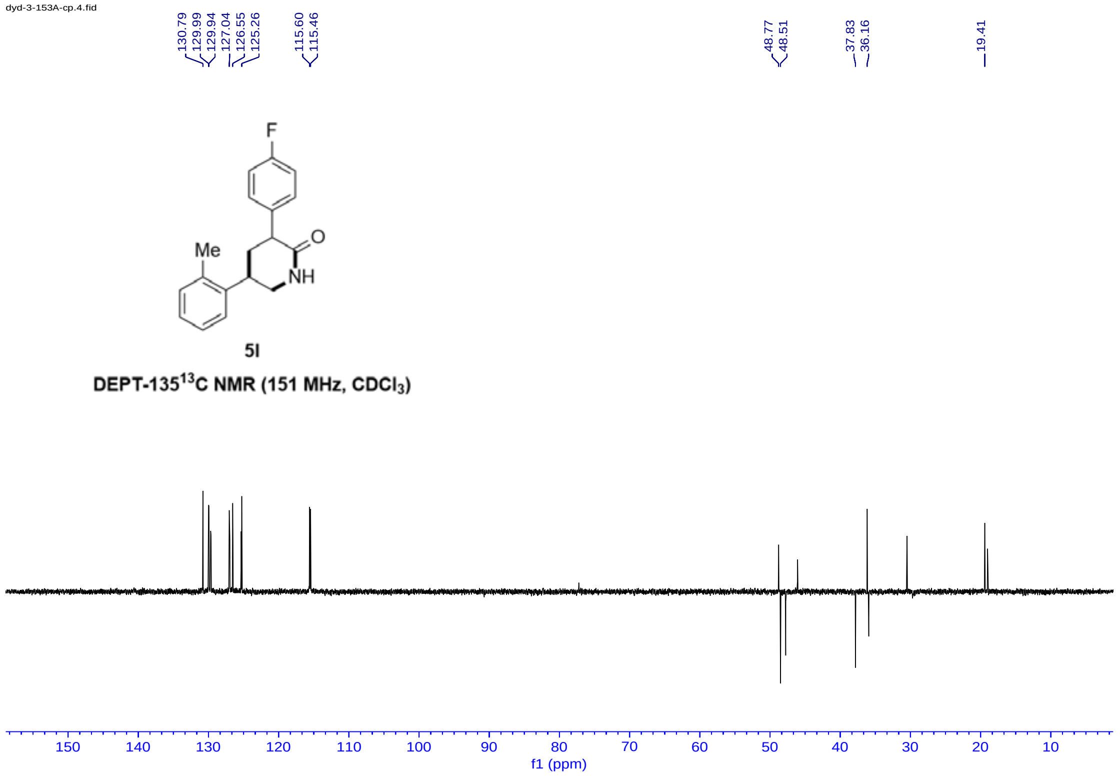

Supplementary Figure 188 DEPT-135 ^{13}C NMR (151 MHz, 298K, $CDCl_3$) of **5l**

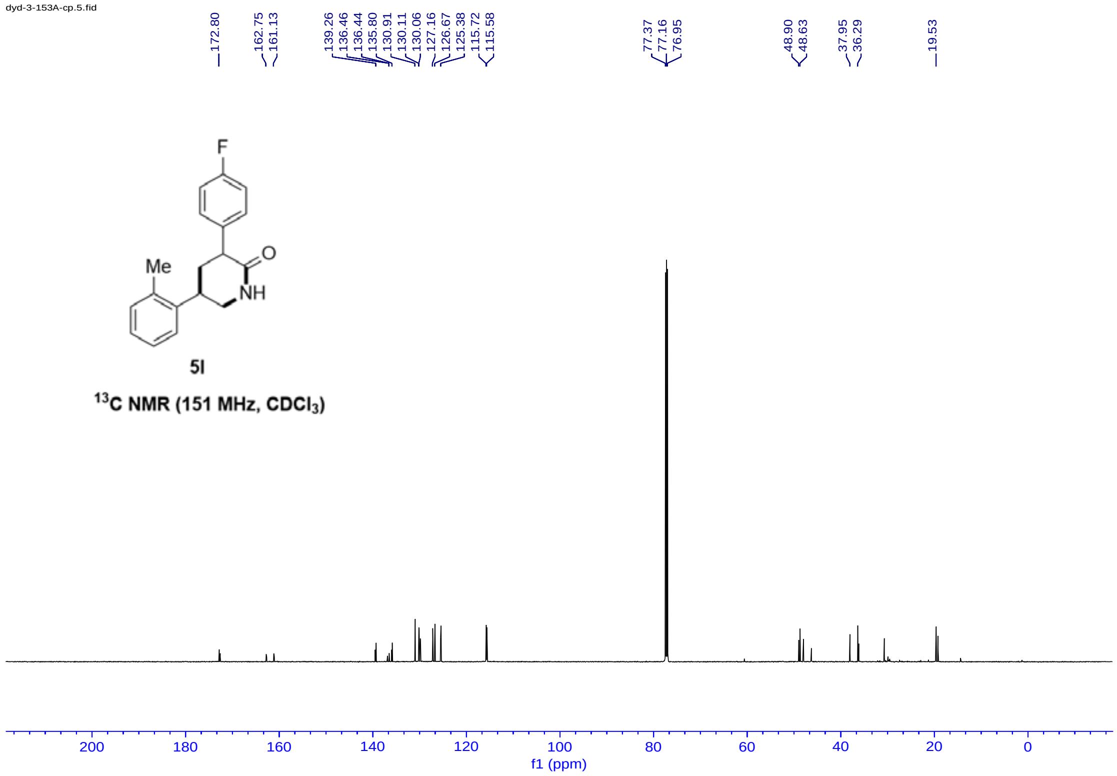

Supplementary Figure 189 ^{13}C NMR (151 MHz, 298K, $CDCl_3$) of **5l**

3-(4-fluorophenyl)-5-(*m*-tolyl)piperidin-2-one

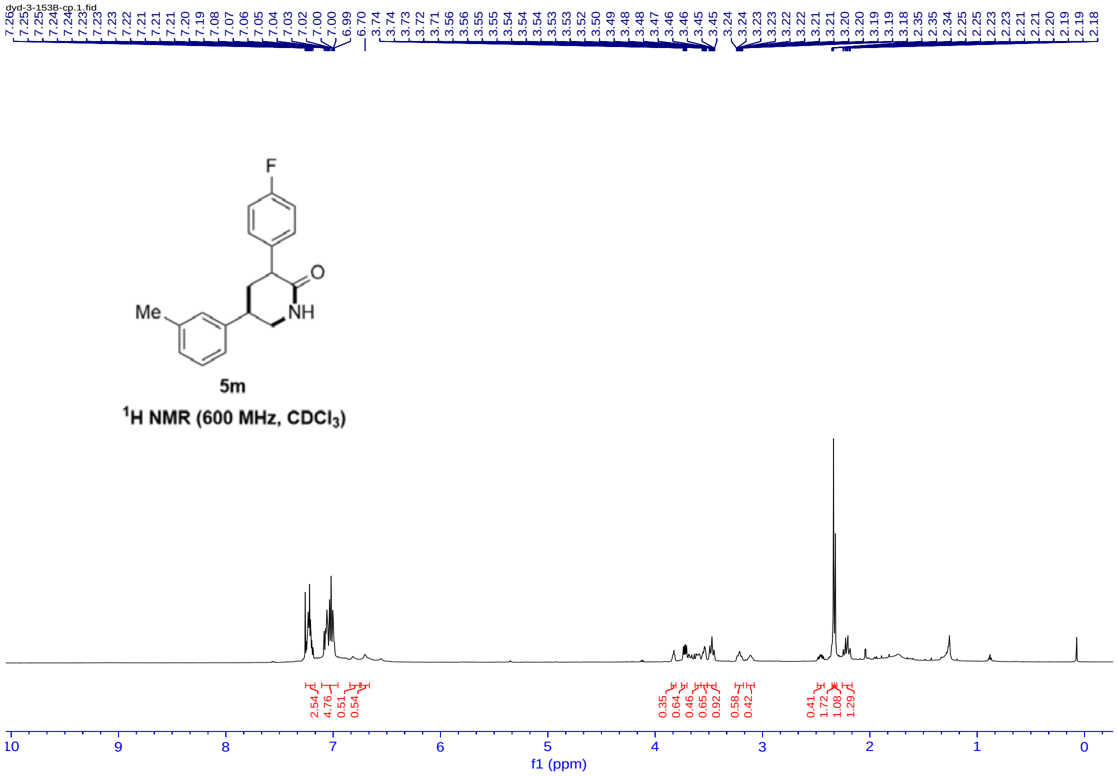

Supplementary Figure 190 ^{1}H NMR (600 MHz, 298K, $CDCl_3$) of **5m**

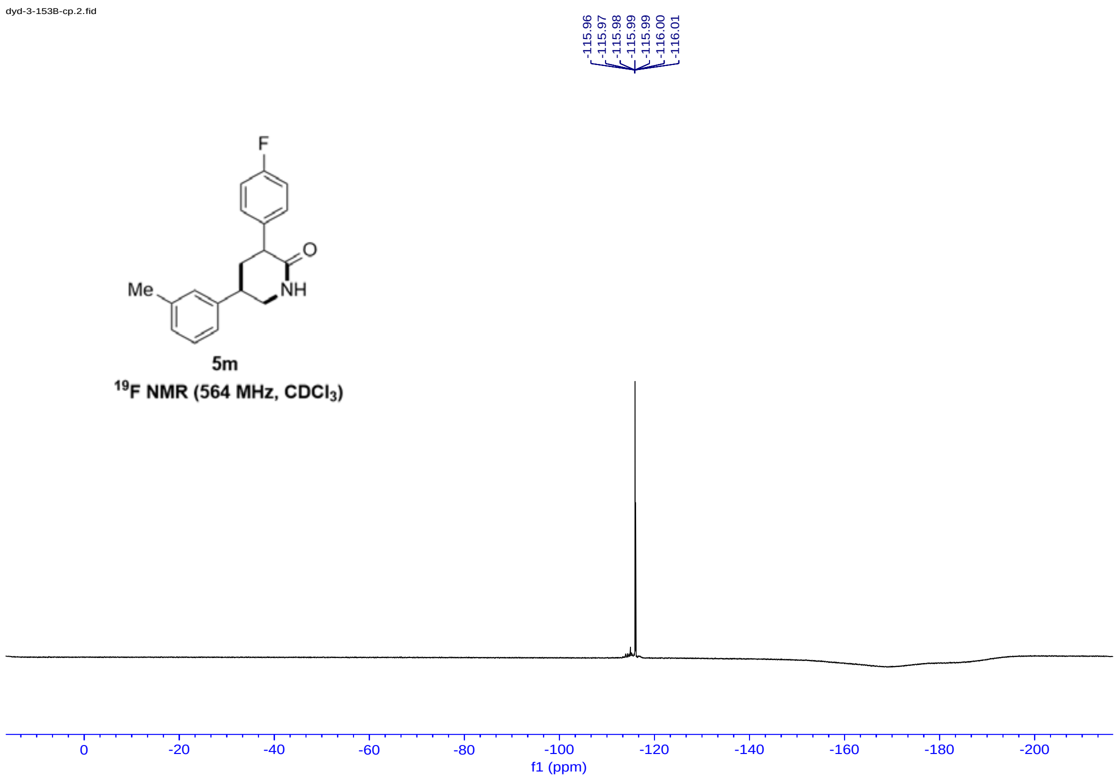

Supplementary Figure 191 ^{19}F NMR (564 MHz, 298K, $CDCl_3$) of **5m**

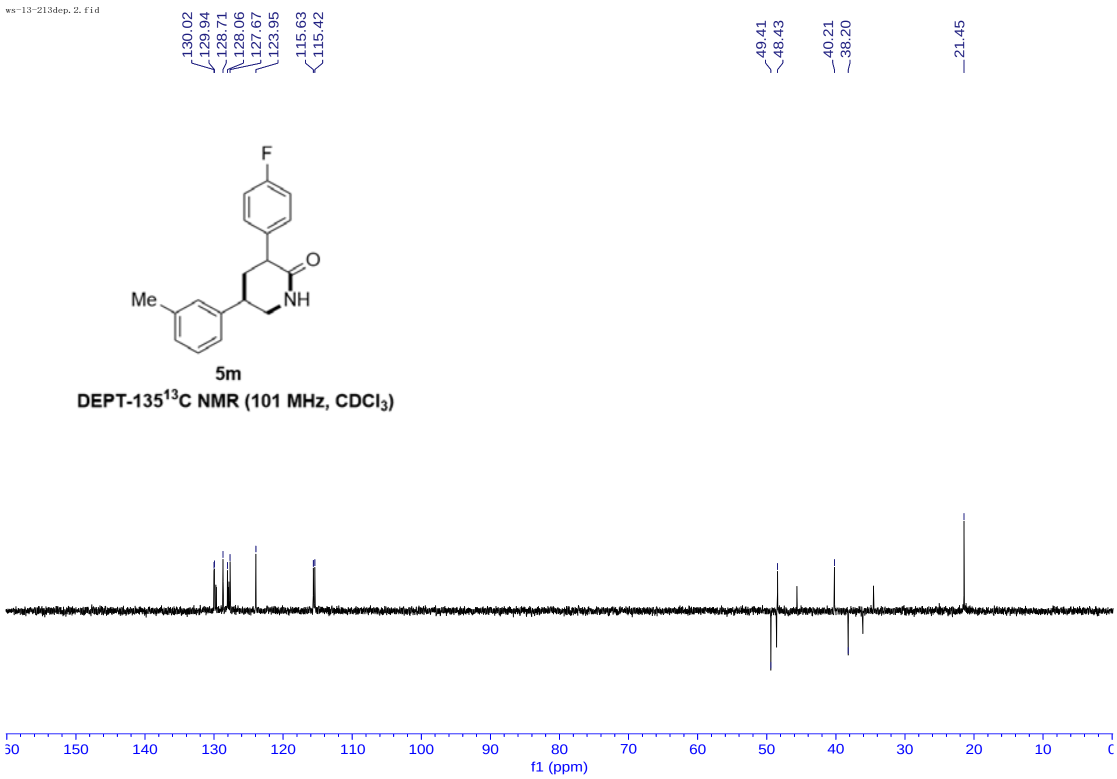

Supplementary Figure 192 DEPT-135 ^{13}C NMR (101 MHz, 298K, $CDCl_3$) of **5m**

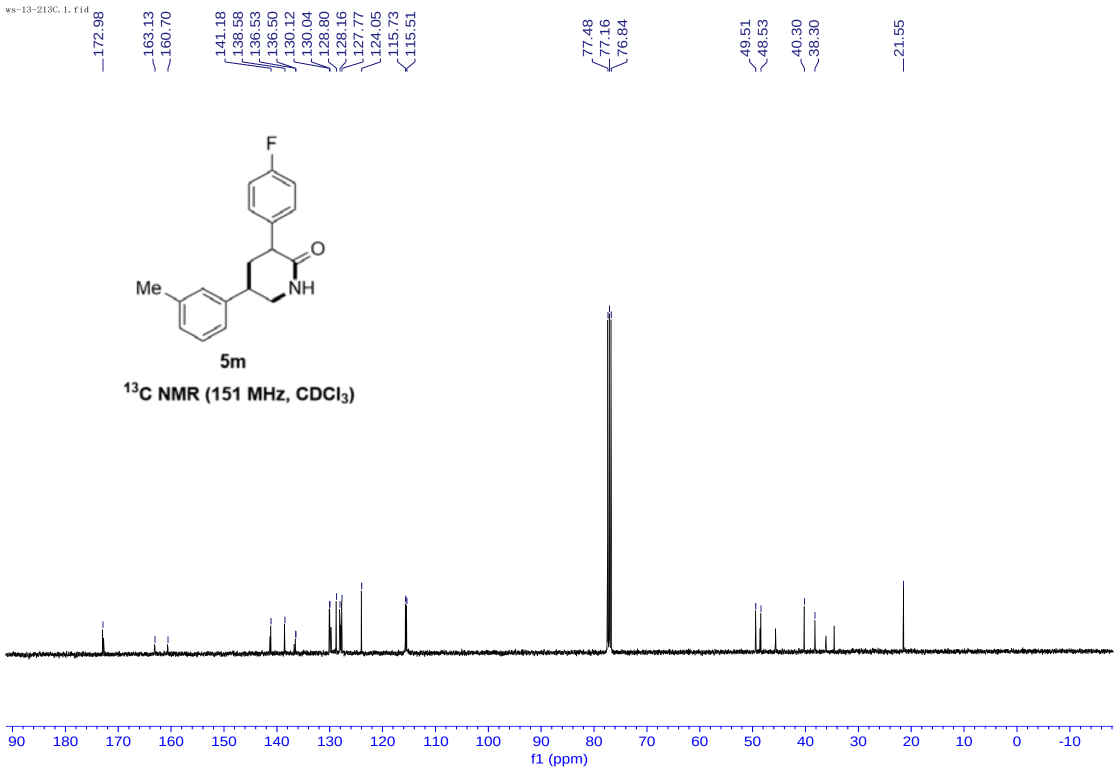

Supplementary Figure 193 ^{13}C NMR (101 MHz, 298K, $CDCl_3$) of **5m**

5-(2,5-dimethylphenyl)-3-(4-fluorophenyl)piperidin-2-one

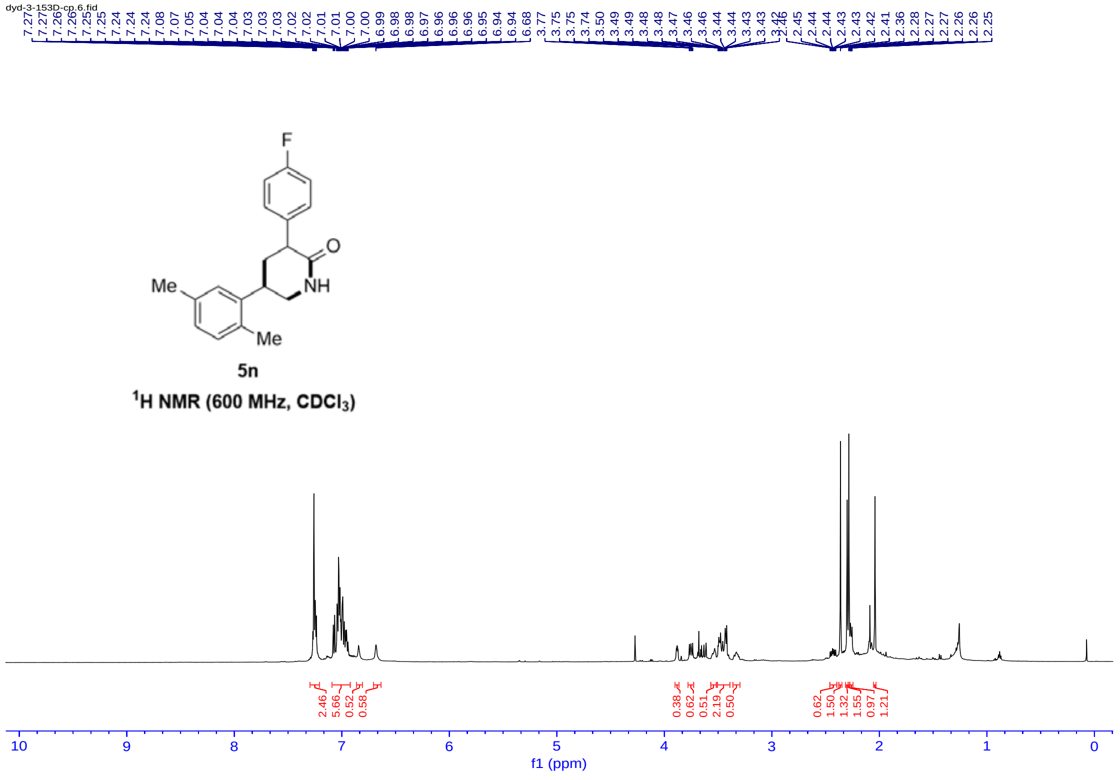

Supplementary Figure 194 ^{1}H NMR (600 MHz, 298K, $CDCl_3$) of **5n**

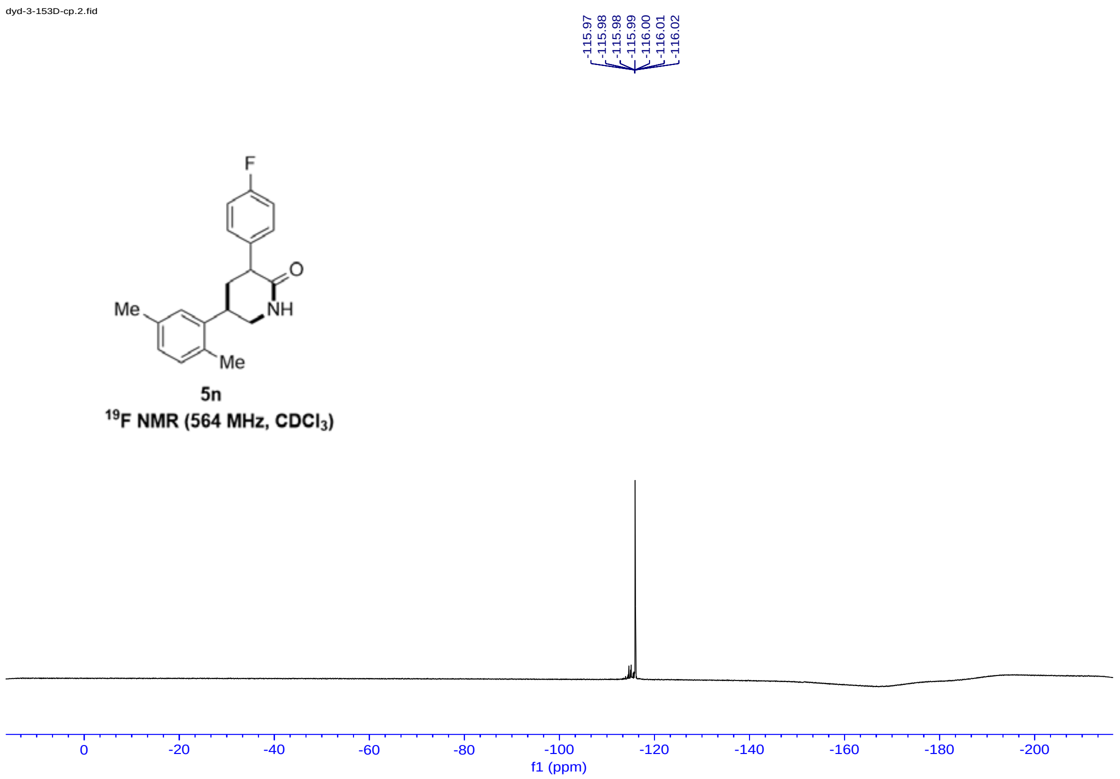

Supplementary Figure 195 ^{19}F NMR (564 MHz, 298K, $CDCl_3$) of **5n**

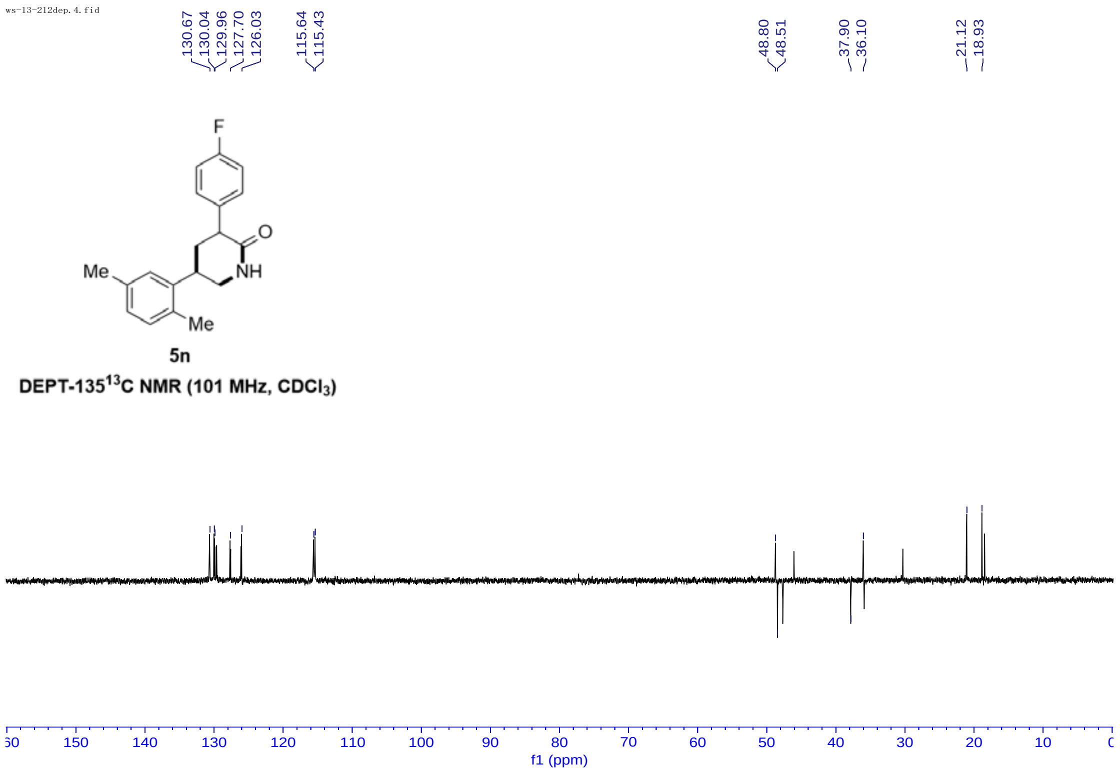

Supplementary Figure 196 DEPT-135 ^{13}C NMR (101 MHz, 298K, $CDCl_3$) of **5n**

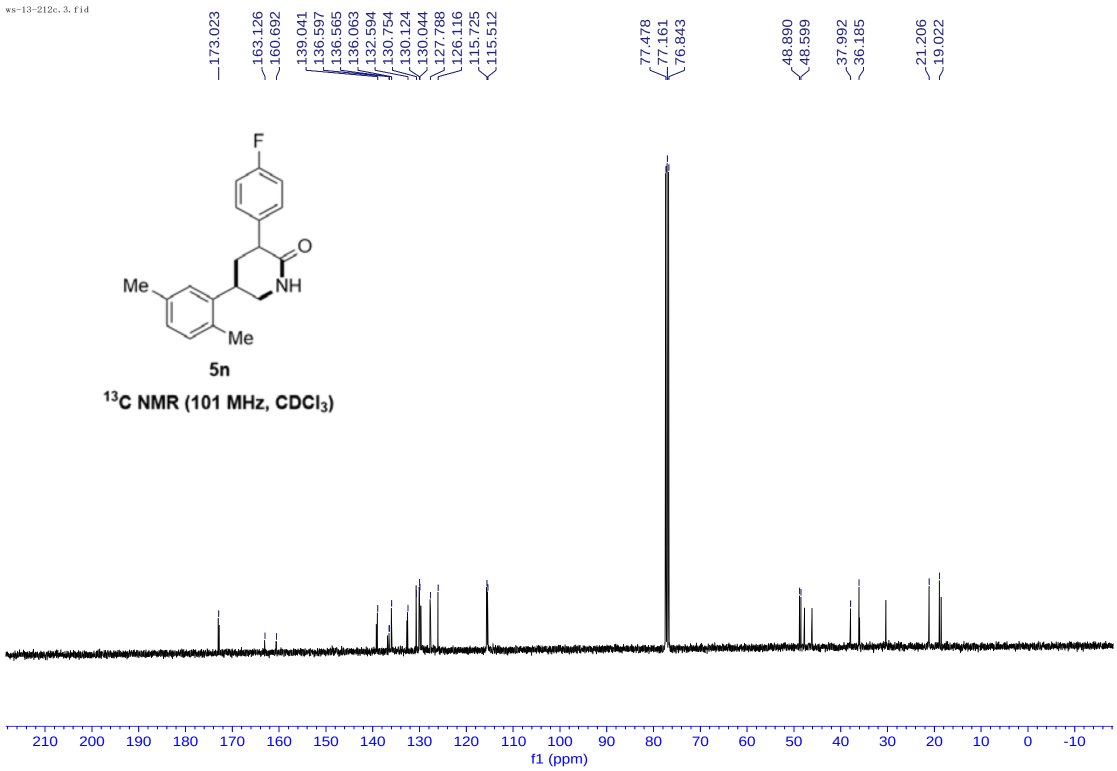

Supplementary Figure 197 ^{13}C NMR (101 MHz, 298K, $CDCl_3$) of **5n**

3-(4-fluorophenyl)-5-methyl-5-phenylpiperidin-2-one

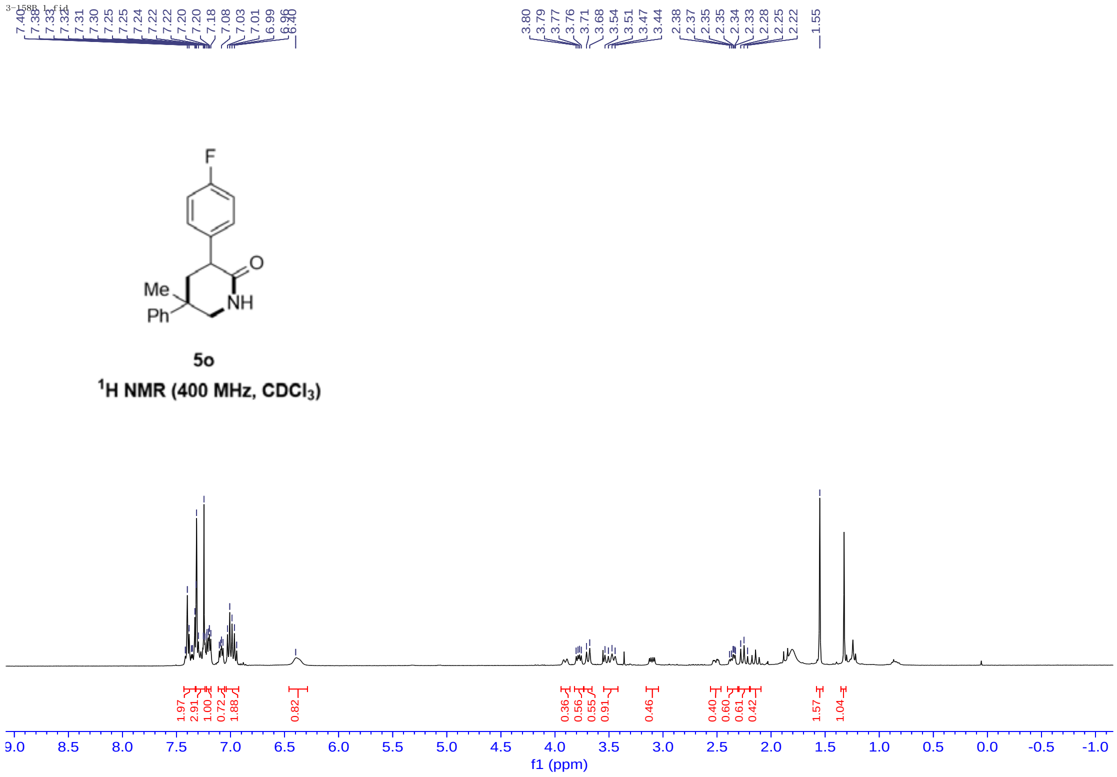

Supplementary Figure 198 ^{1}H NMR (400 MHz, 298K, $CDCl_3$) of **5o**

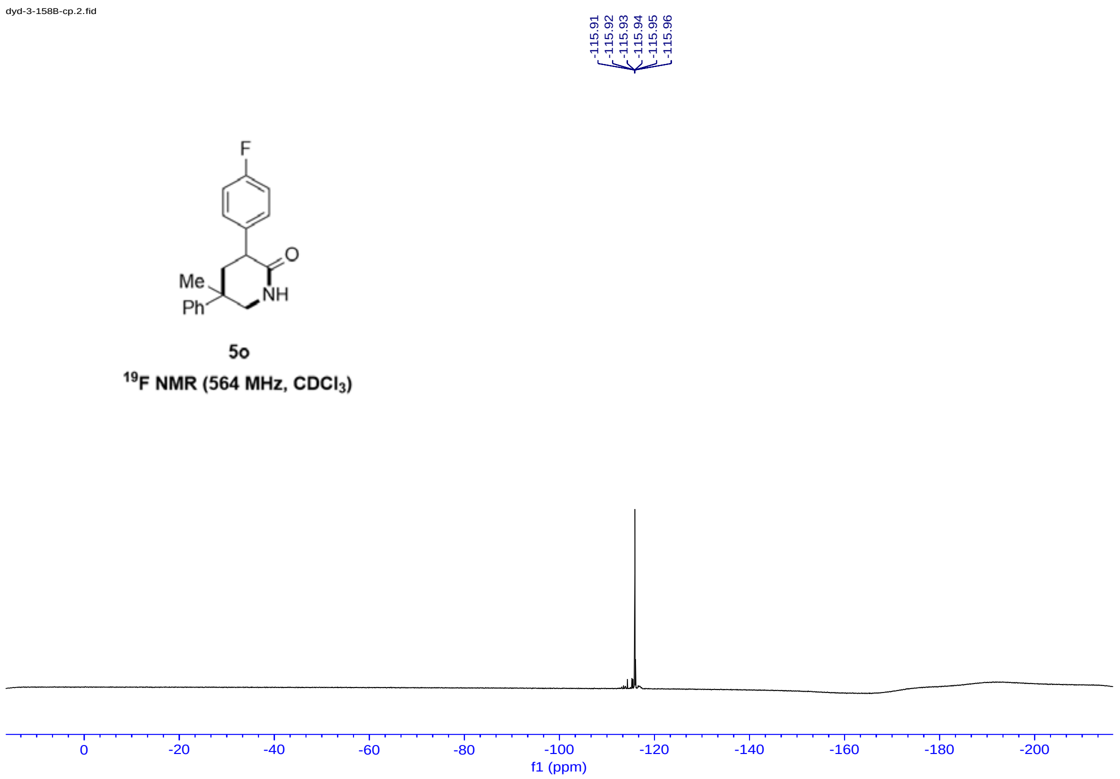

Supplementary Figure 199 ^{19}F NMR (564 MHz, 298K, $CDCl_3$) of **5o**

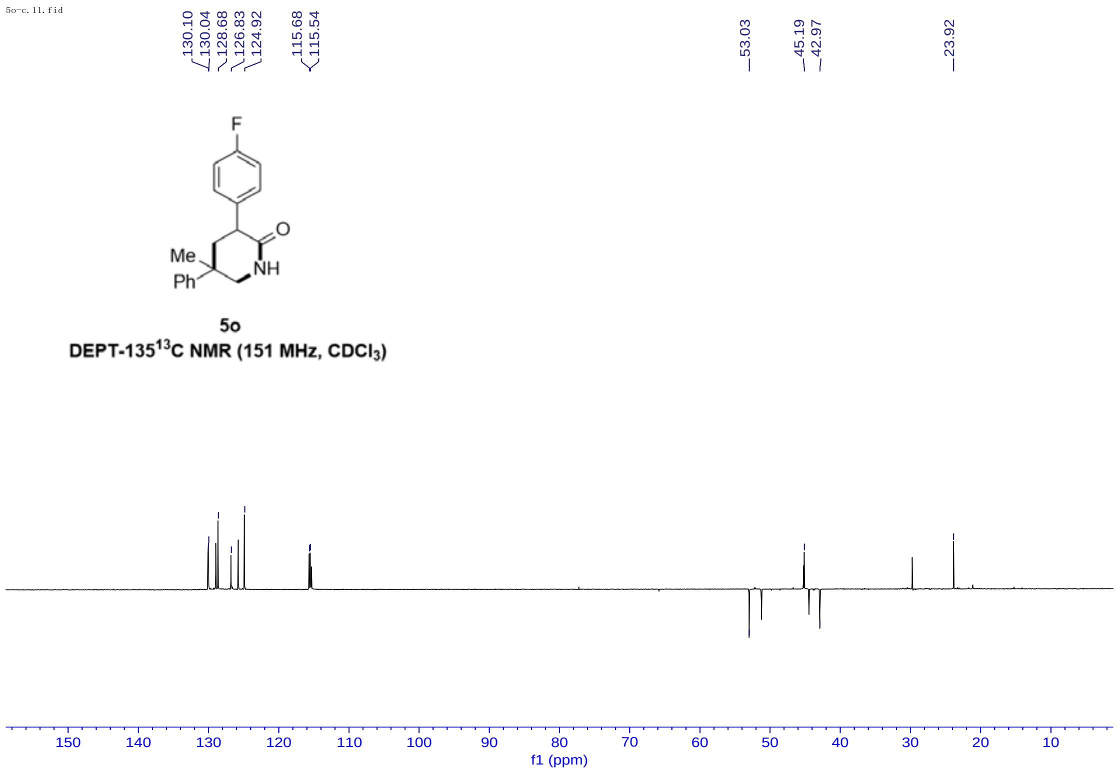

Supplementary Figure 200 DEPT-135 ^{13}C NMR (151 MHz, 298K, $CDCl_3$) of **5o**

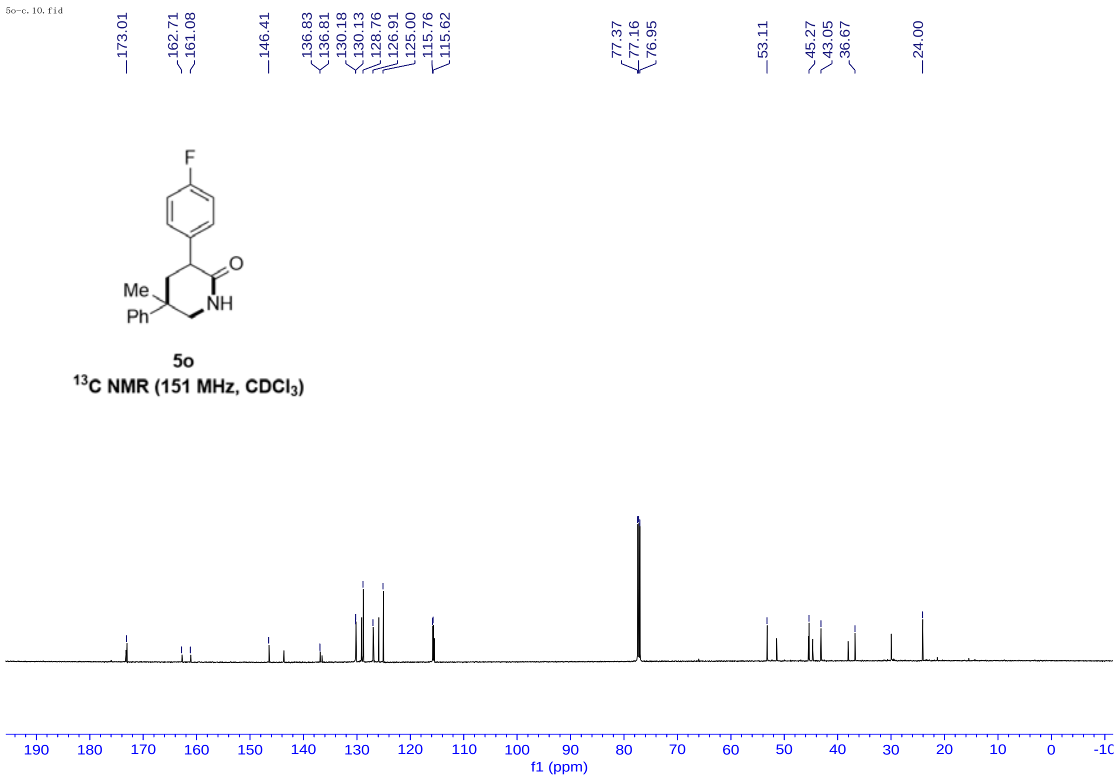

Supplementary Figure 201 ^{13}C NMR (151 MHz, 298K, $CDCl_3$) of **5o**

3-(4-fluorophenyl)-5-methyl-5-(*p*-tolyl)piperidin-2-one

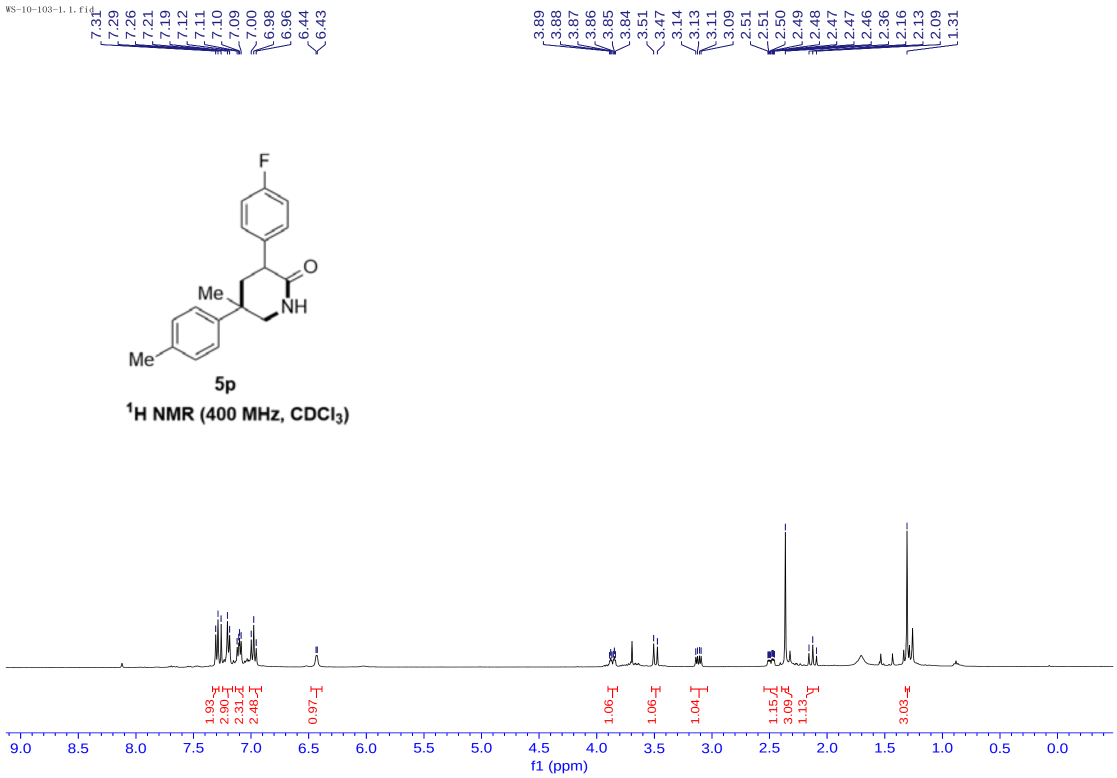

Supplementary Figure 202 ^{1}H NMR (400 MHz, 298K, $CDCl_3$) of **5p**

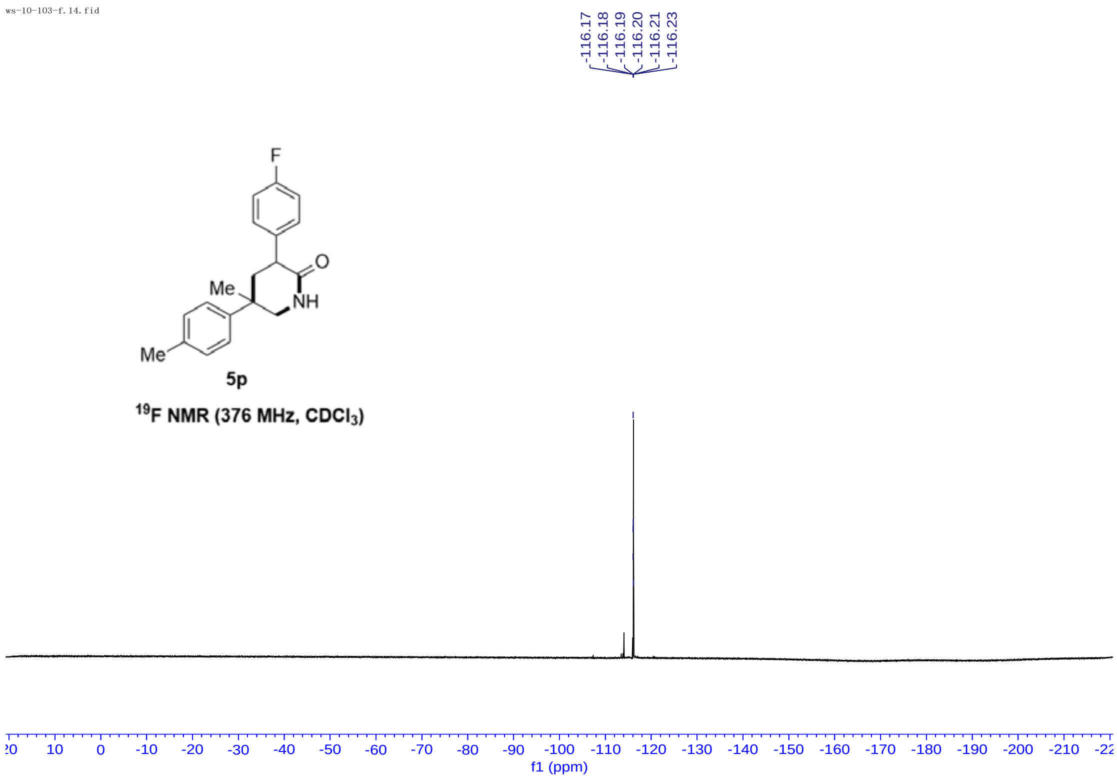

Supplementary Figure 203 ^{19}F NMR (376 MHz, 298K, $CDCl_3$) of **5p**

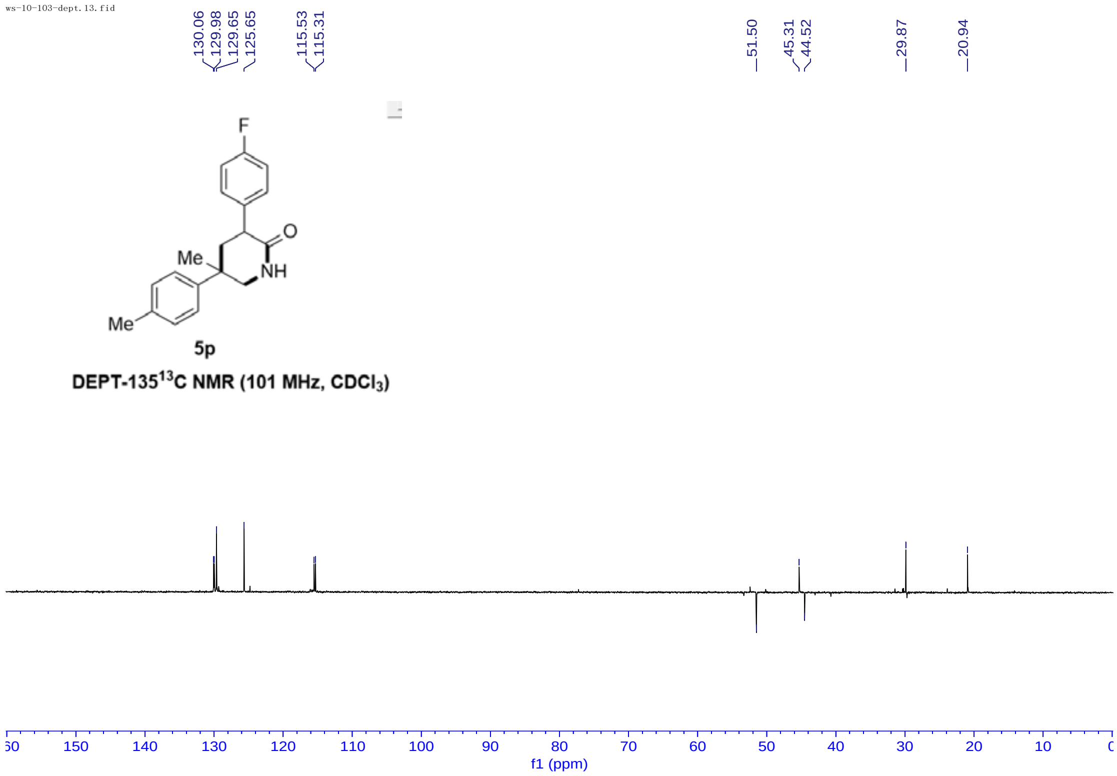

Supplementary Figure 204 DEPT-135 ^{13}C NMR (101 MHz, 298K, $CDCl_3$) of **5p**

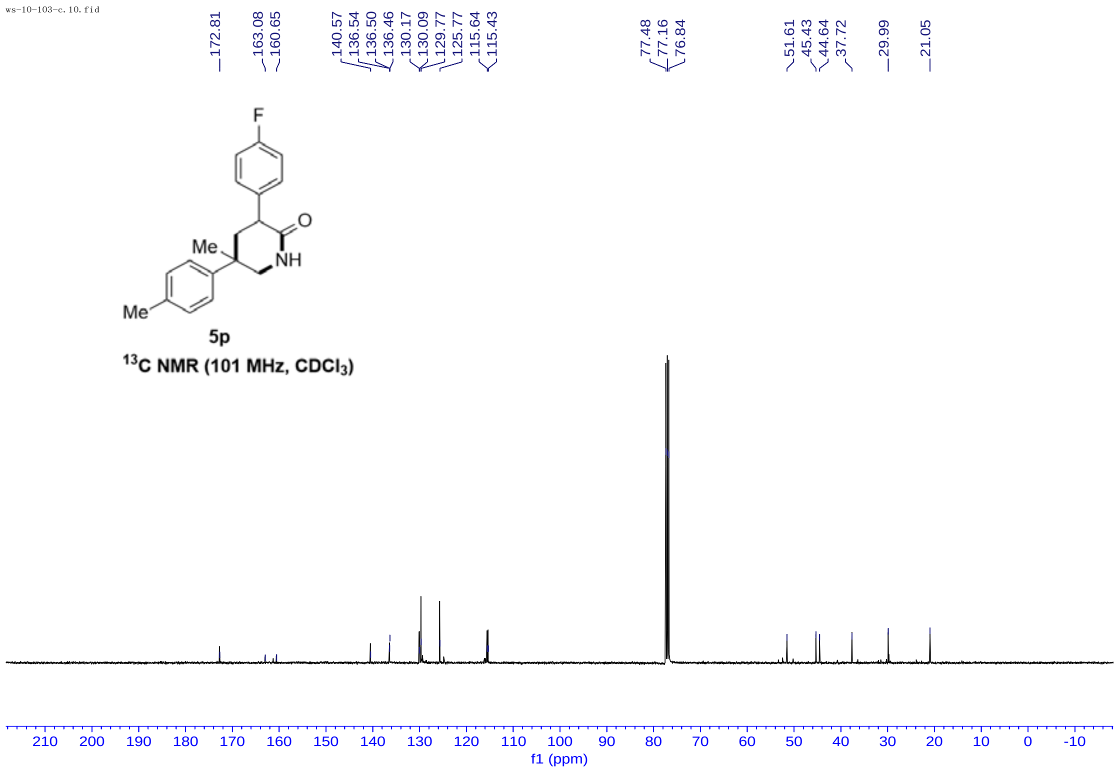

Supplementary Figure 205 ^{13}C NMR (101 MHz, 298K, $CDCl_3$) of **5p**

5-(4-(allyloxy)phenyl)-3-(4-fluorophenyl)-5-methylpiperidin-2-one

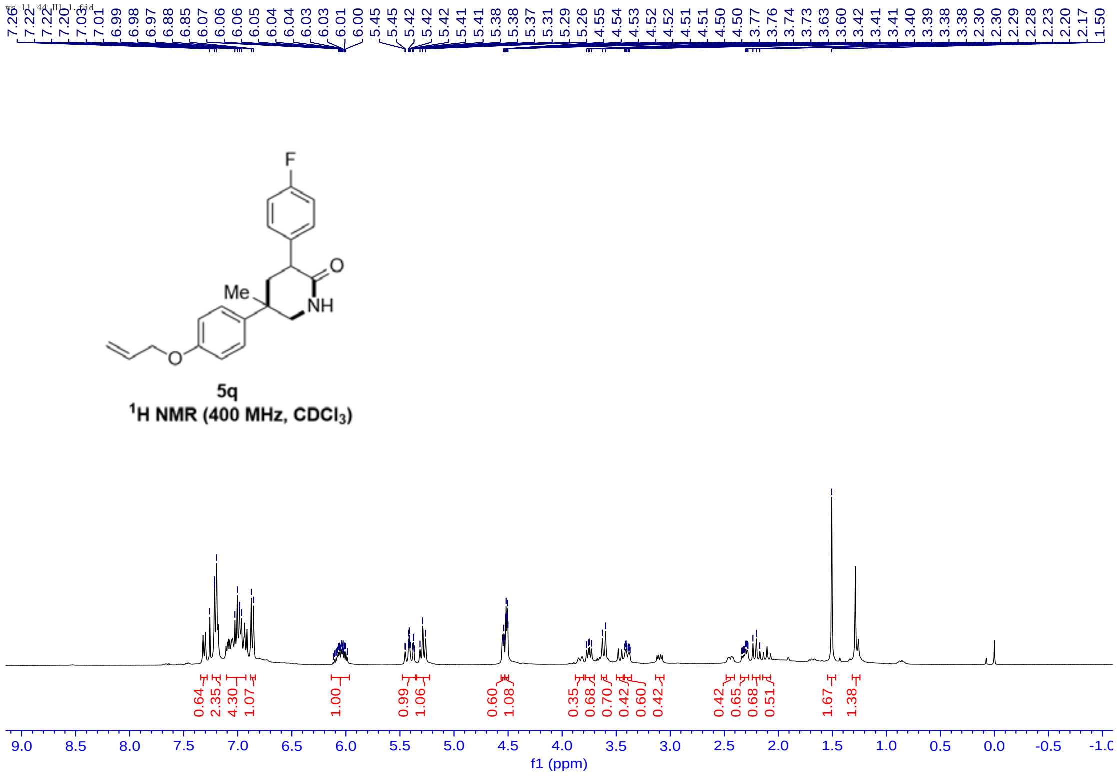

Supplementary Figure 206 ^{1}H NMR (400 MHz, 298K, $CDCl_3$) of **5q**

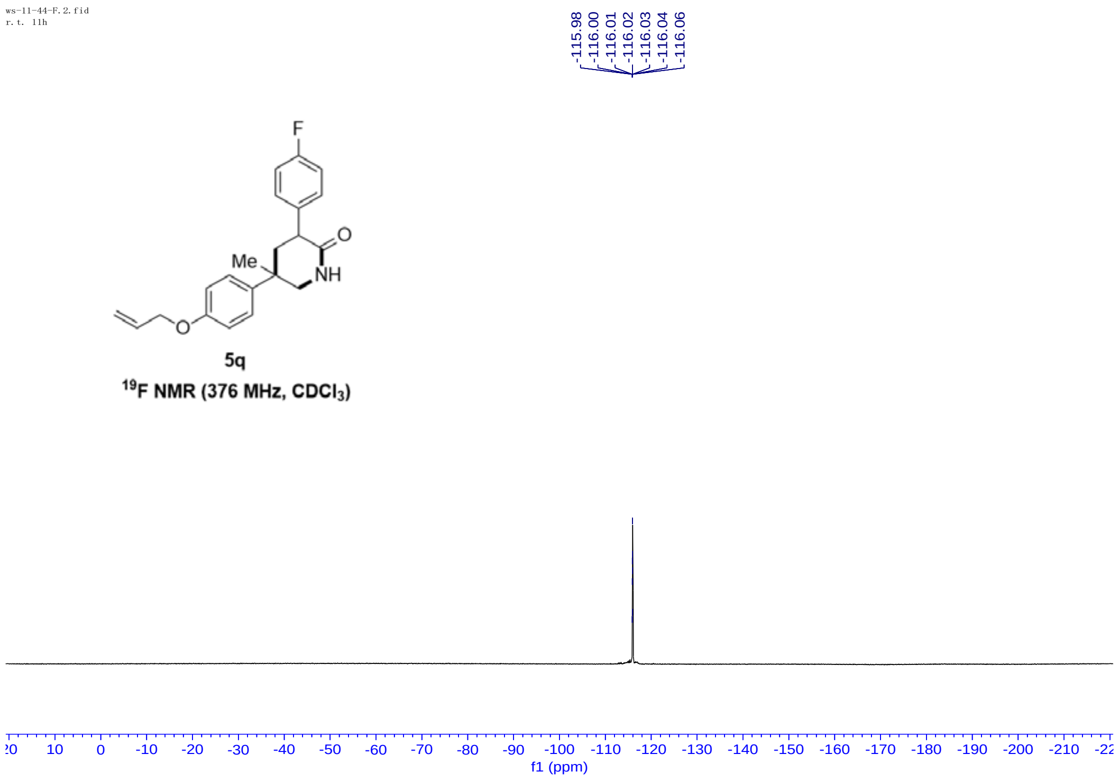

Supplementary Figure 207 ^{19}F NMR (376 MHz, 298K, $CDCl_3$) of **5q**

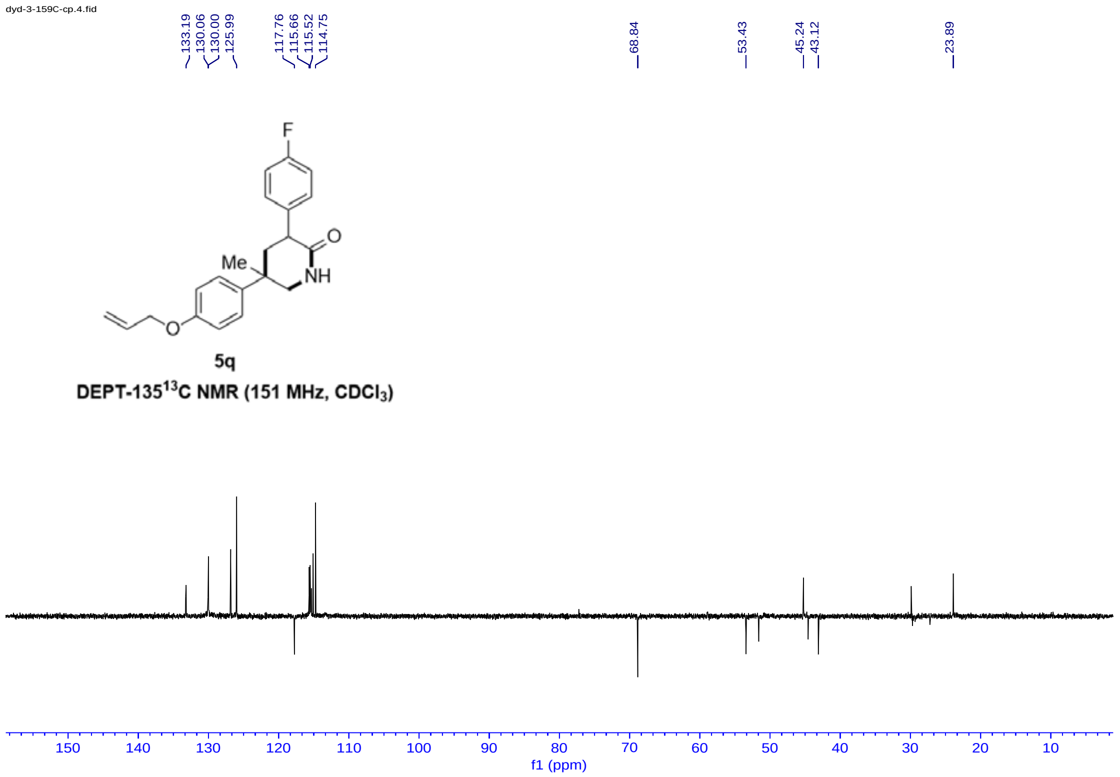

Supplementary Figure 208 DEPT-135 ^{13}C NMR (151 MHz, 298K, $CDCl_3$) of **5q**

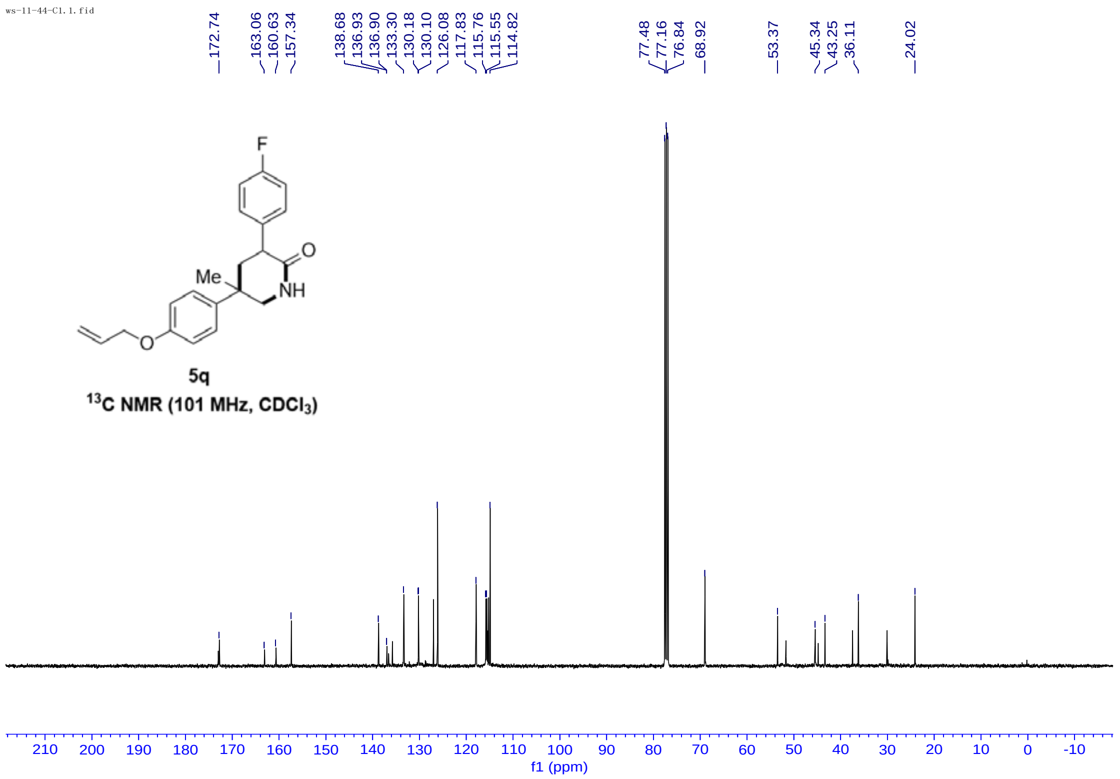

Supplementary Figure 209 ^{13}C NMR (101 MHz, 298K, $CDCl_3$) of **5q**

3-(4-fluorophenyl)-5-methyl-5-(4-((3-methylbut-2-en-1-yl)oxy)phenyl)piperidin-2-one

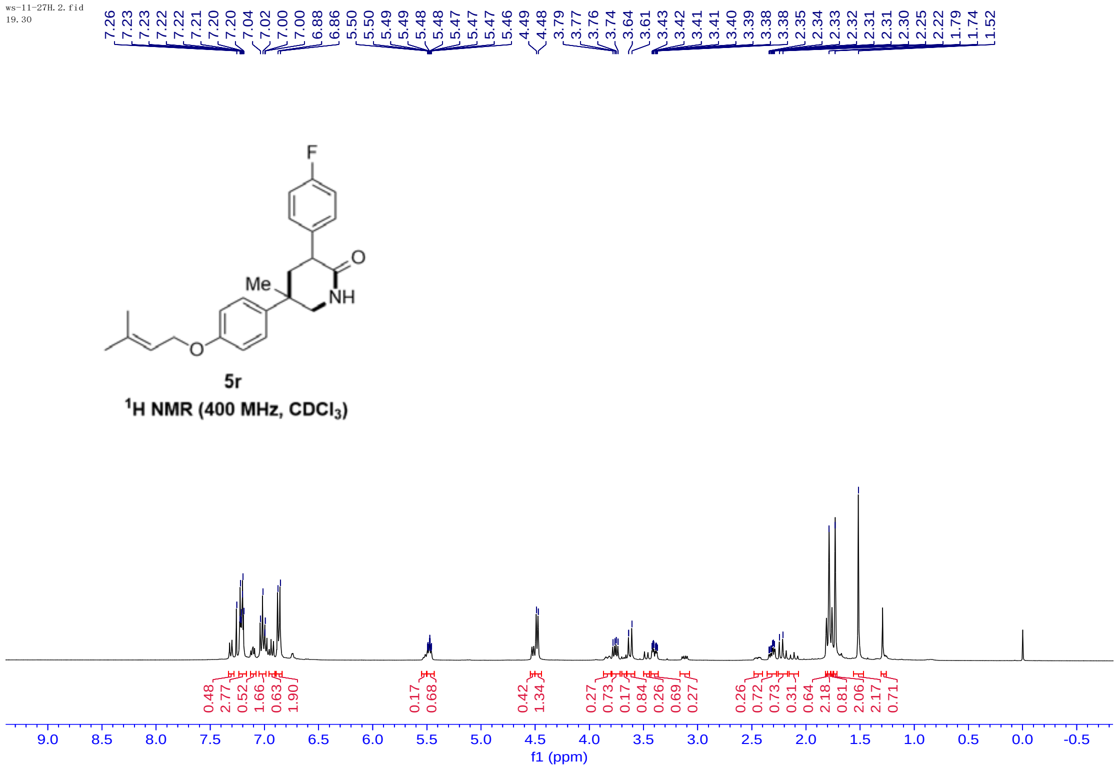

Supplementary Figure 210 ^{1}H NMR (400 MHz, 298K, $CDCl_3$) of **5r**

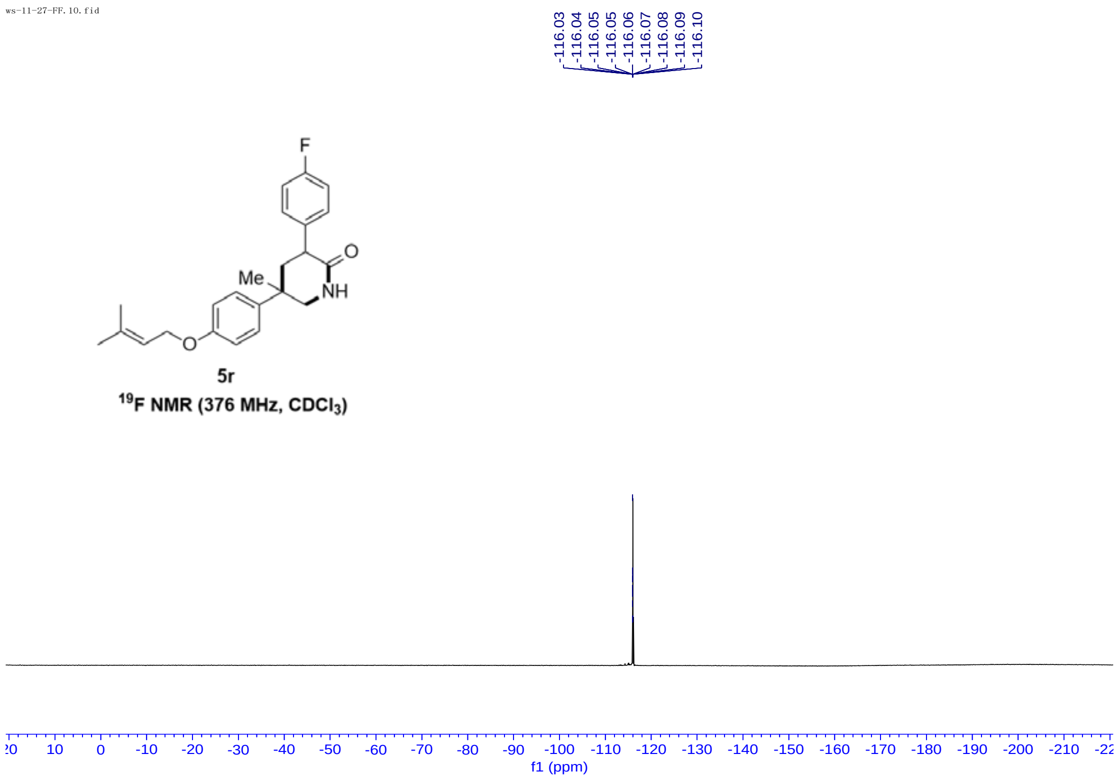

Supplementary Figure 211 ^{19}F NMR (376 MHz, 298K, $CDCl_3$) of **5r**

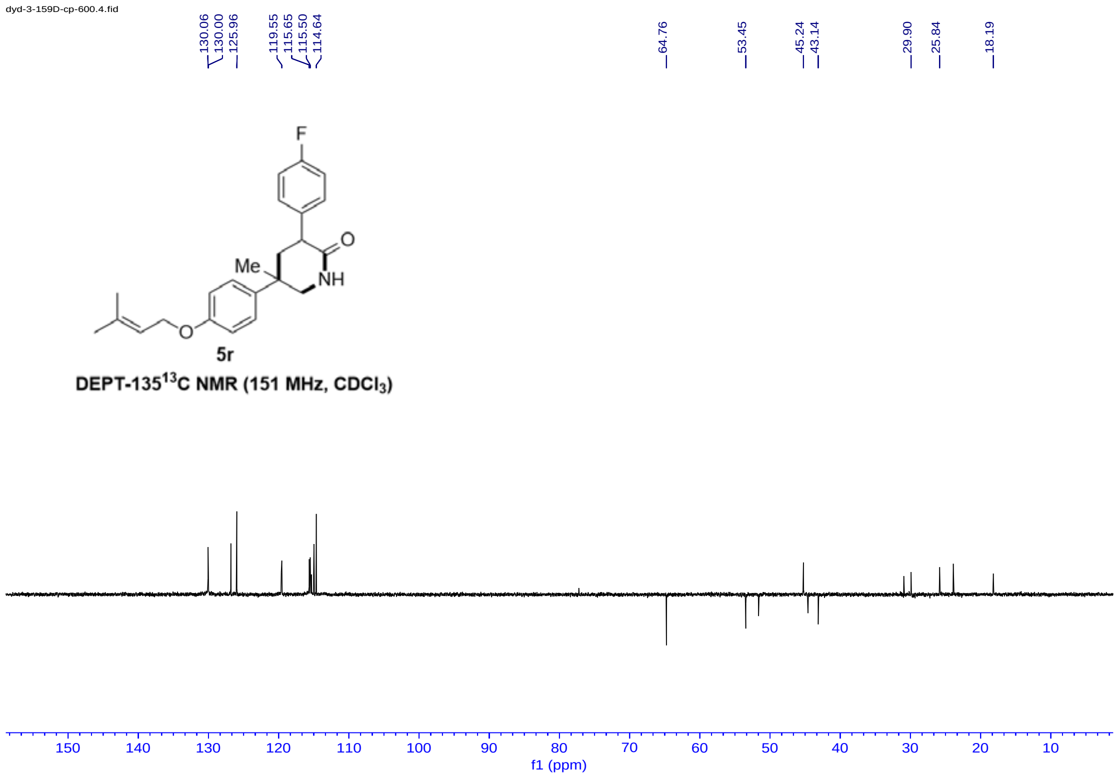

Supplementary Figure 212 DEPT-135 ^{13}C NMR (151 MHz, 298K, $CDCl_3$) of **5r**

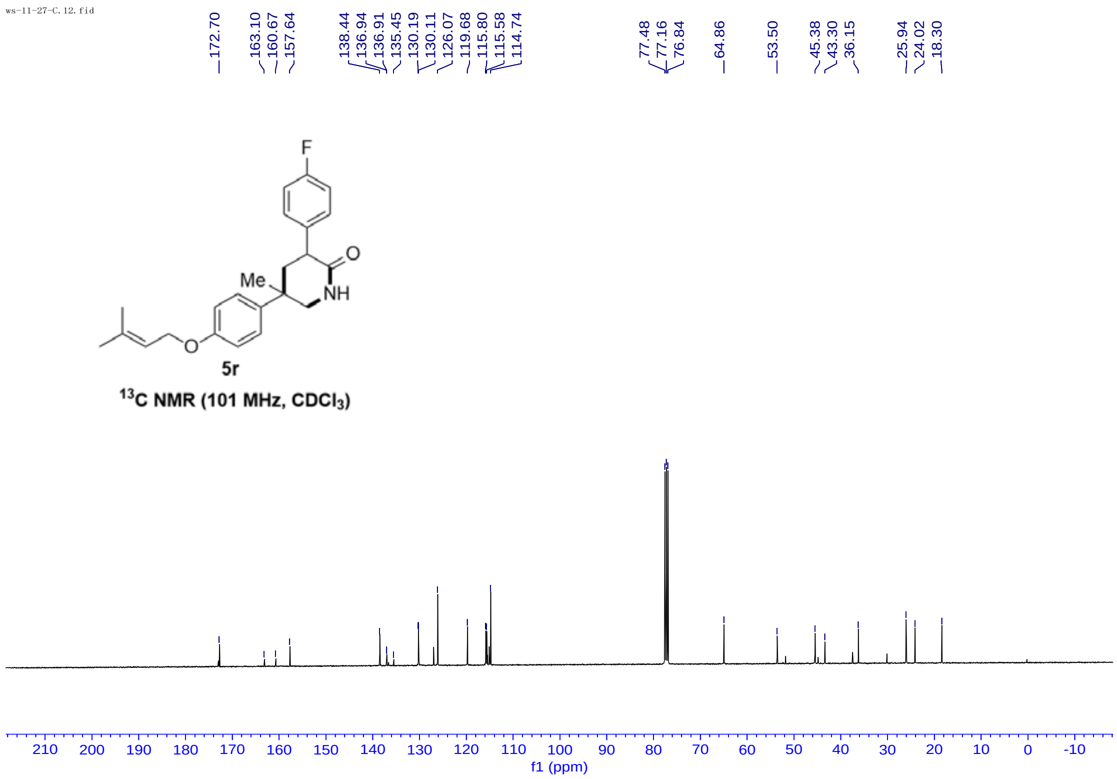

Supplementary Figure 213 ^{13}C NMR (101 MHz, 298K, $CDCl_3$) of **5r**

***tert*-butylallyl(4-(5-(4-fluorophenyl)-3-methyl-6-oxopiperidin-3-yl)phenyl) carbamate**

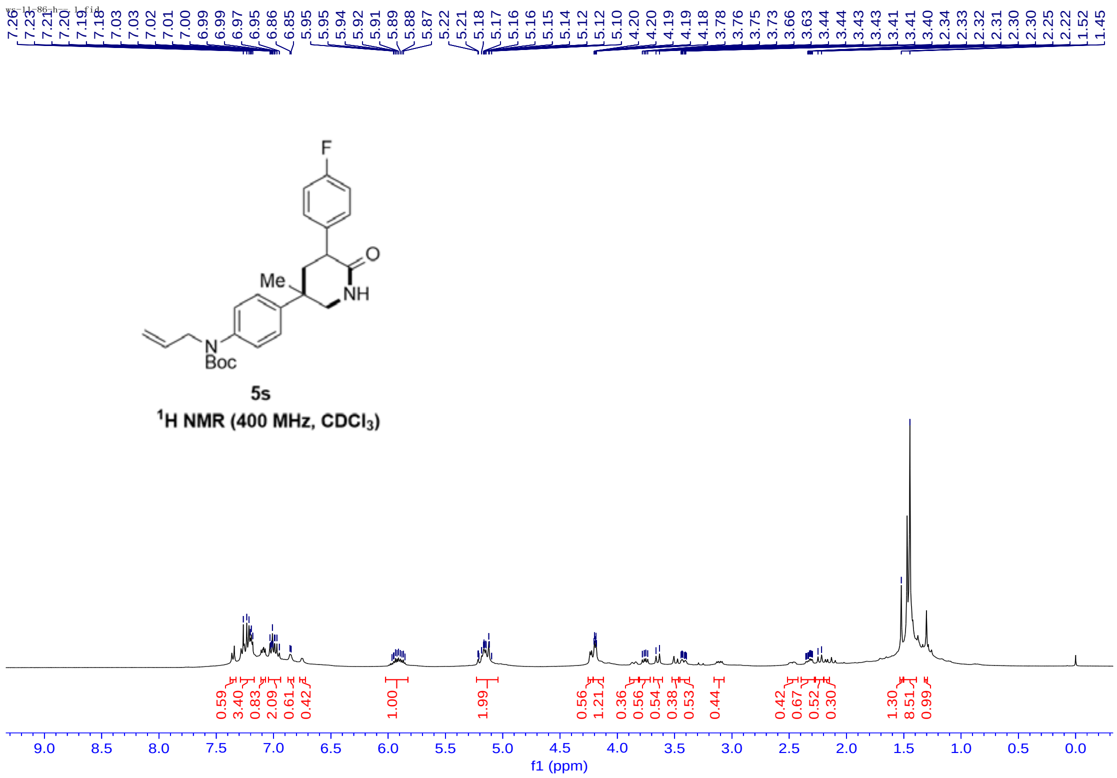

Supplementary Figure 214 ^{1}H NMR (400 MHz, 298K, $CDCl_3$) of **5s**

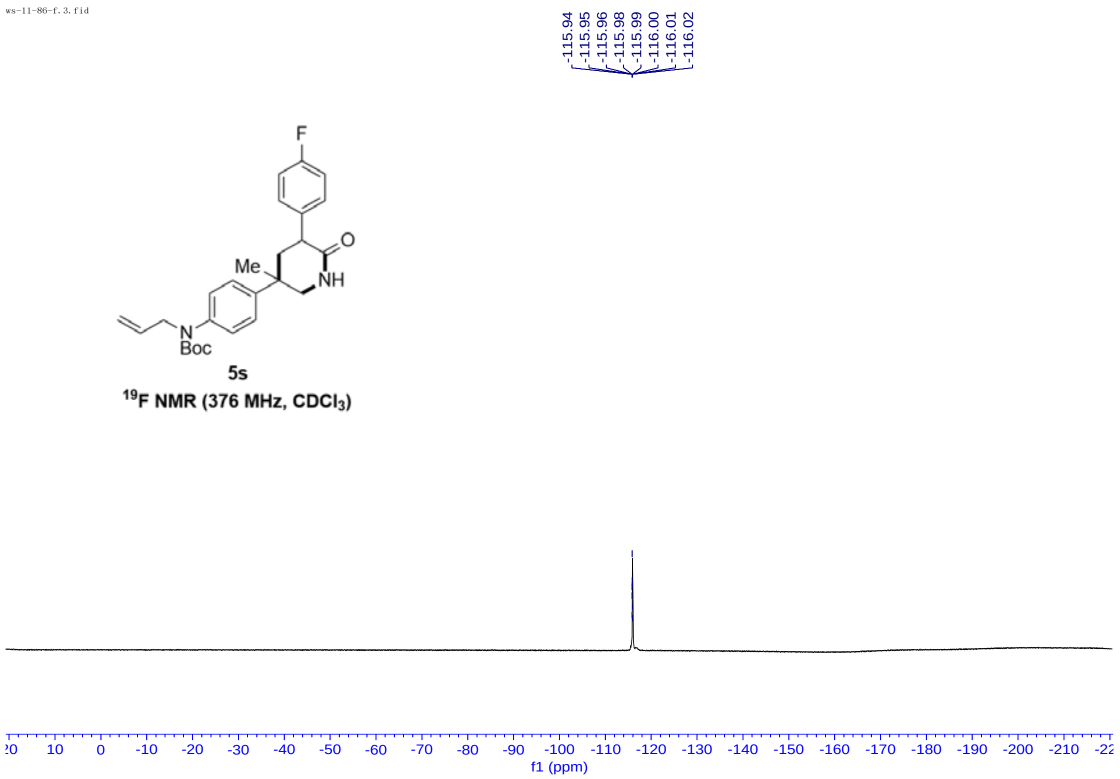

Supplementary Figure 215 ^{19}F NMR (376 MHz, 298K, $CDCl_3$) of **5s**

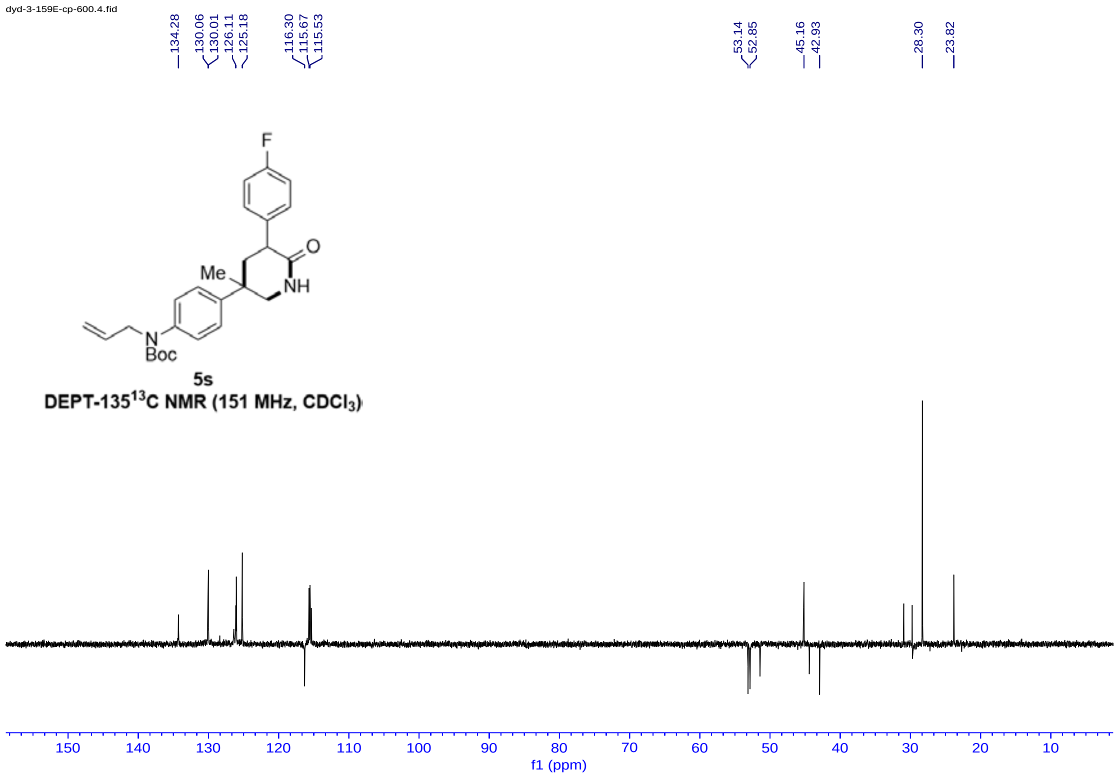

Supplementary Figure 216 DEPT-135 ^{13}C NMR (151 MHz, 298K, $CDCl_3$) of **5s**

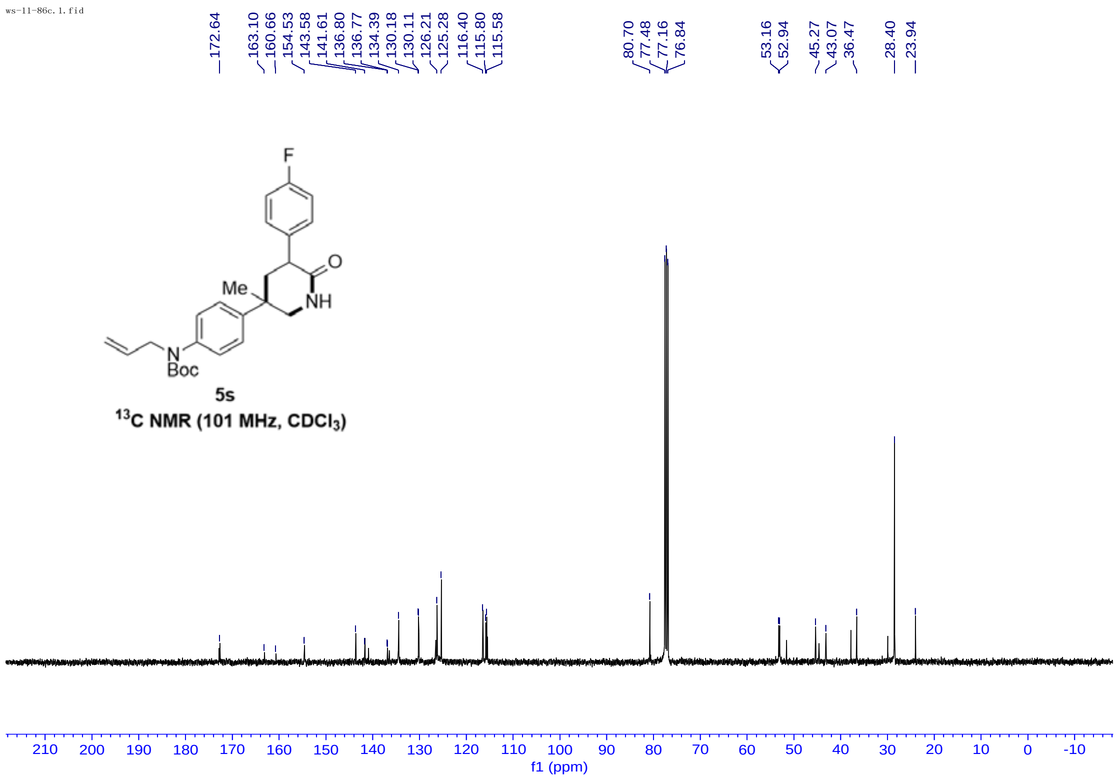

Supplementary Figure 217 ^{13}C NMR (101 MHz, 298K, $CDCl_3$) of **5s**

***cis*-3-(4-fluorophenyl)-5-(4-(2-hydroxypropan-2-yl)phenyl)piperidin-2-one**

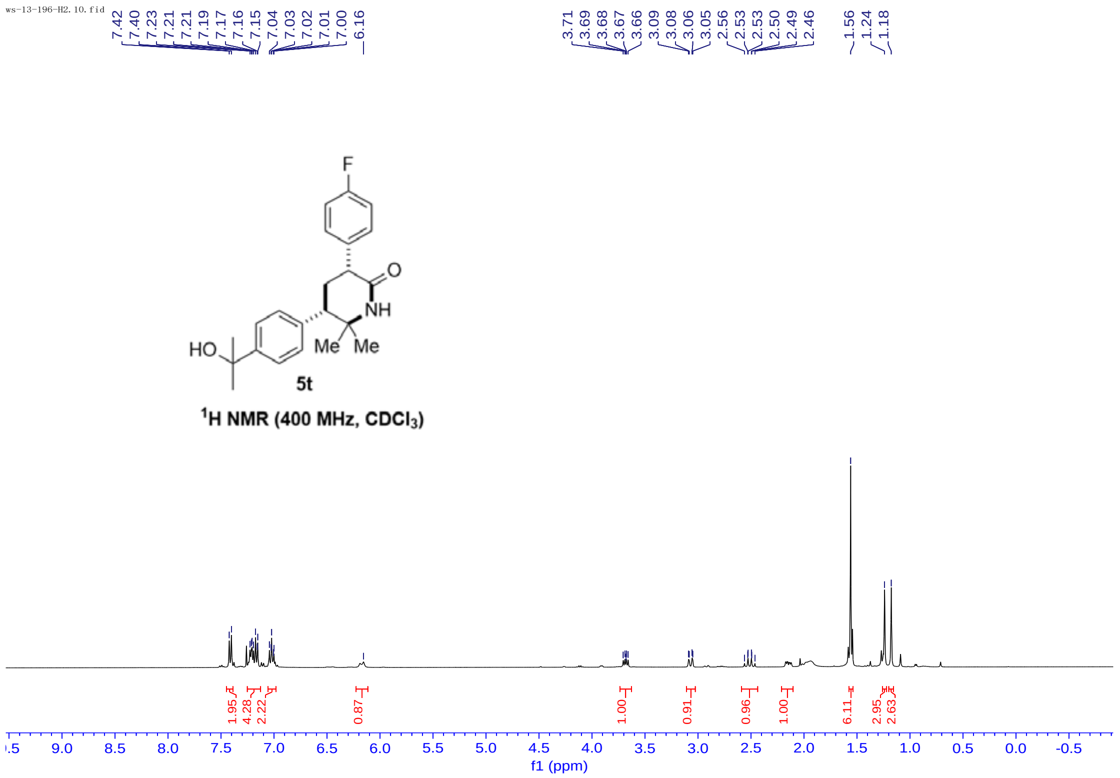

Supplementary Figure 218 ^{1}H NMR (400 MHz, 298K, $CDCl_3$) of **5t**

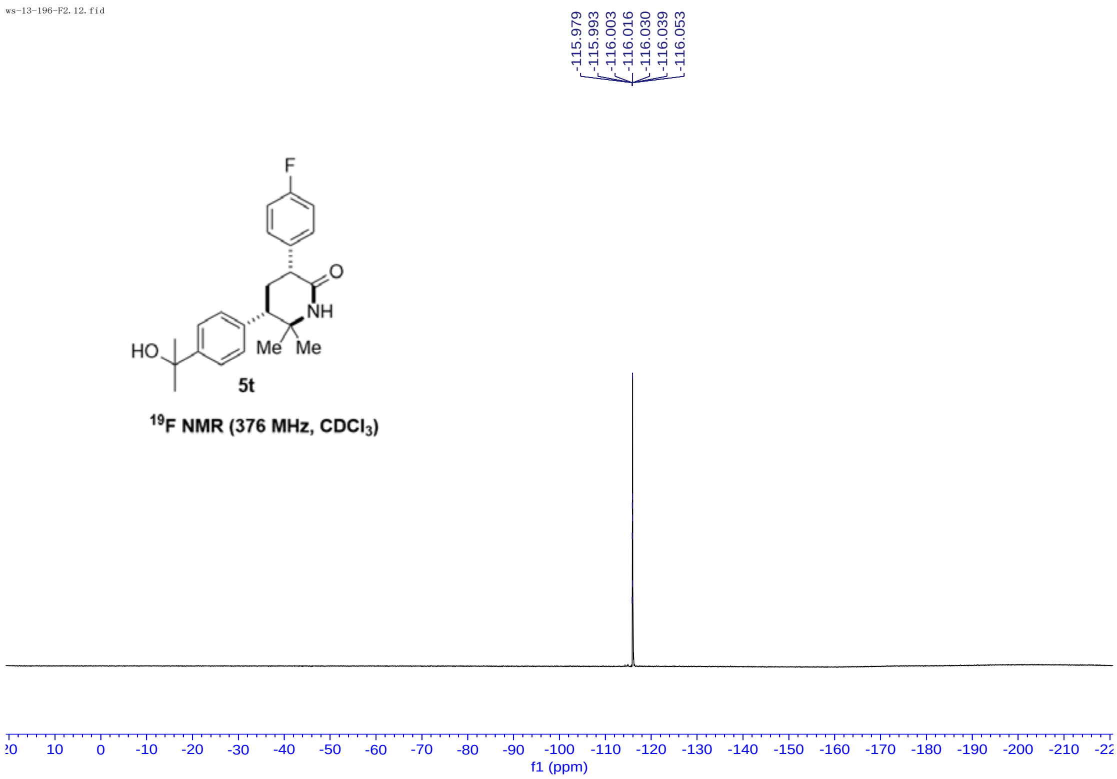

Supplementary Figure 219 ^{19}F NMR (376 MHz, 298K, $CDCl_3$) of **5t**

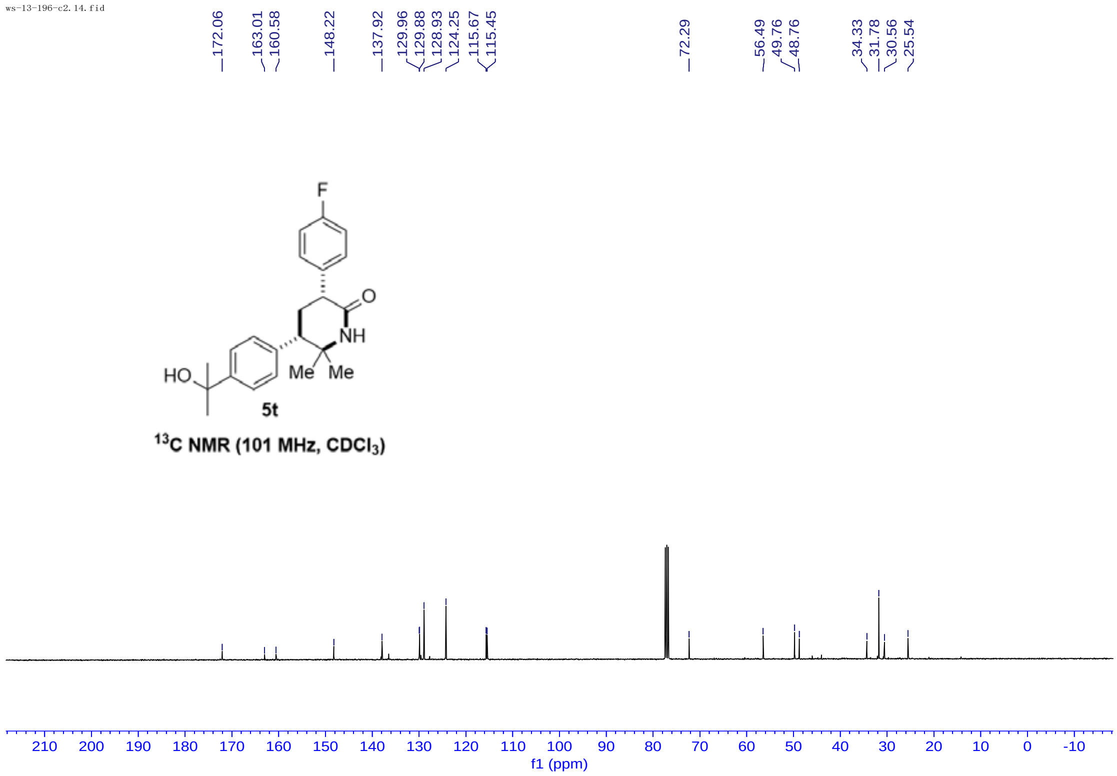

Supplementary Figure 220 ^{13}C NMR (101 MHz, 298K, $CDCl_3$) of **5t**

4a-((trimethylsilyl)oxy)octahydroquinolin-2(1*H*)-one

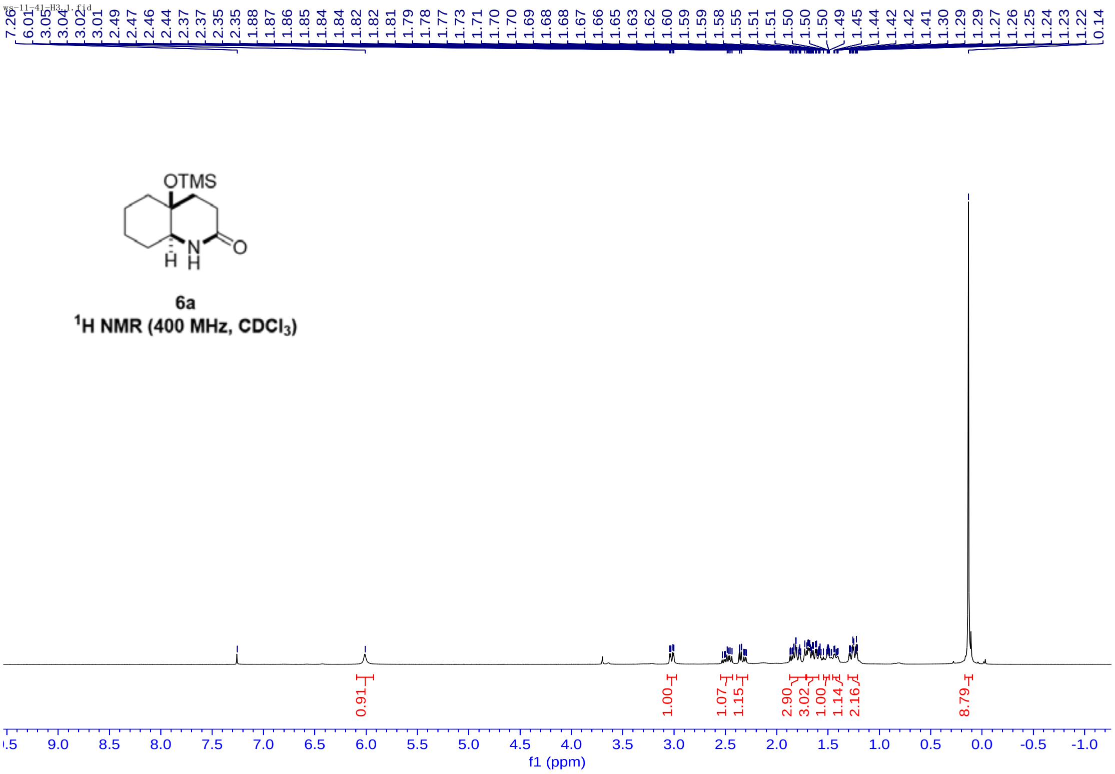

Supplementary Figure 221 ^{1}H NMR (400 MHz, 298K, $CDCl_3$) of **6a**

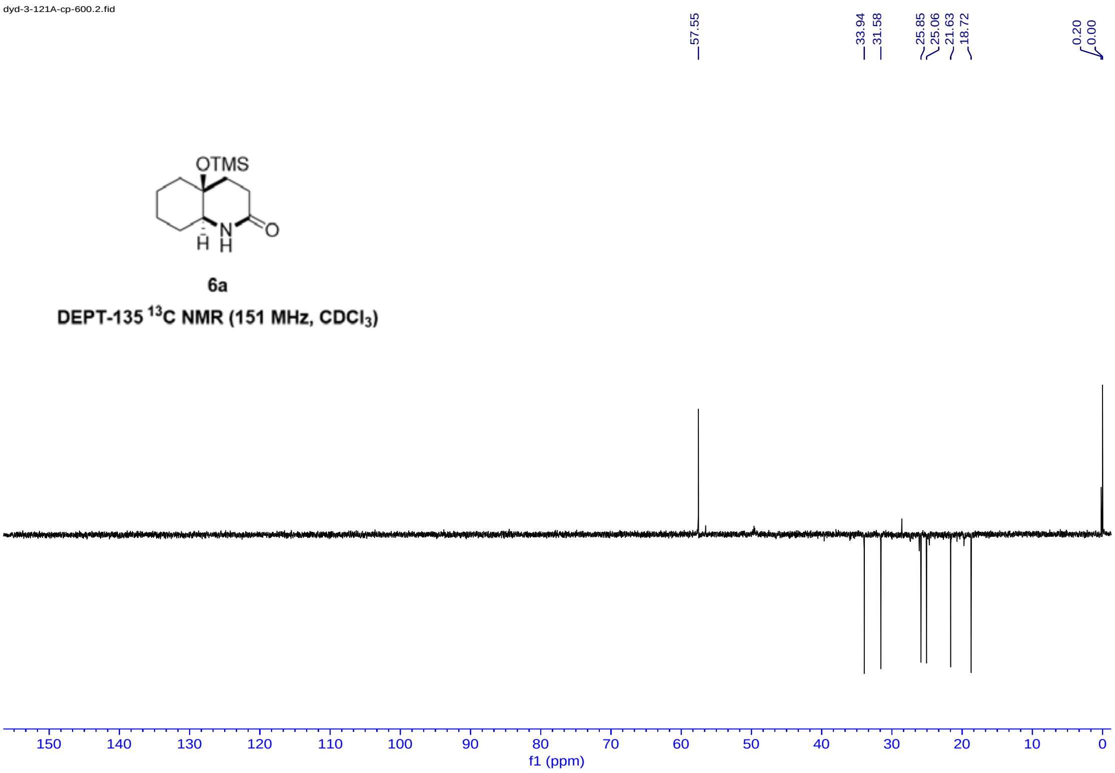

Supplementary Figure 222 DEPT-135 ^{13}C NMR (151 MHz, 298K, $CDCl_3$) of **6a**

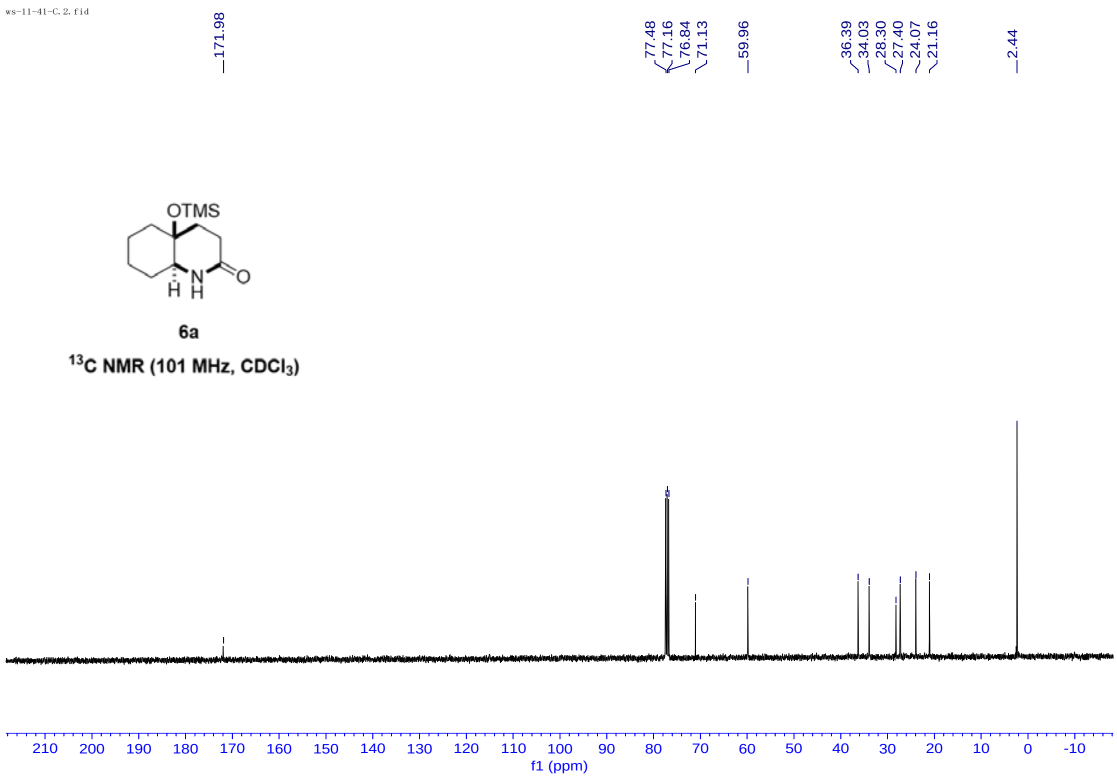

Supplementary Figure 223 ^{13}C NMR (101 MHz, 298K, CDCl$_3$) of **6a**

*(*3a*S*,5*S*,7a*R*)-5-(4-methoxyphenyl)hexahydrofuro[2,3-*b*]pyridin-6(2*H*)-one

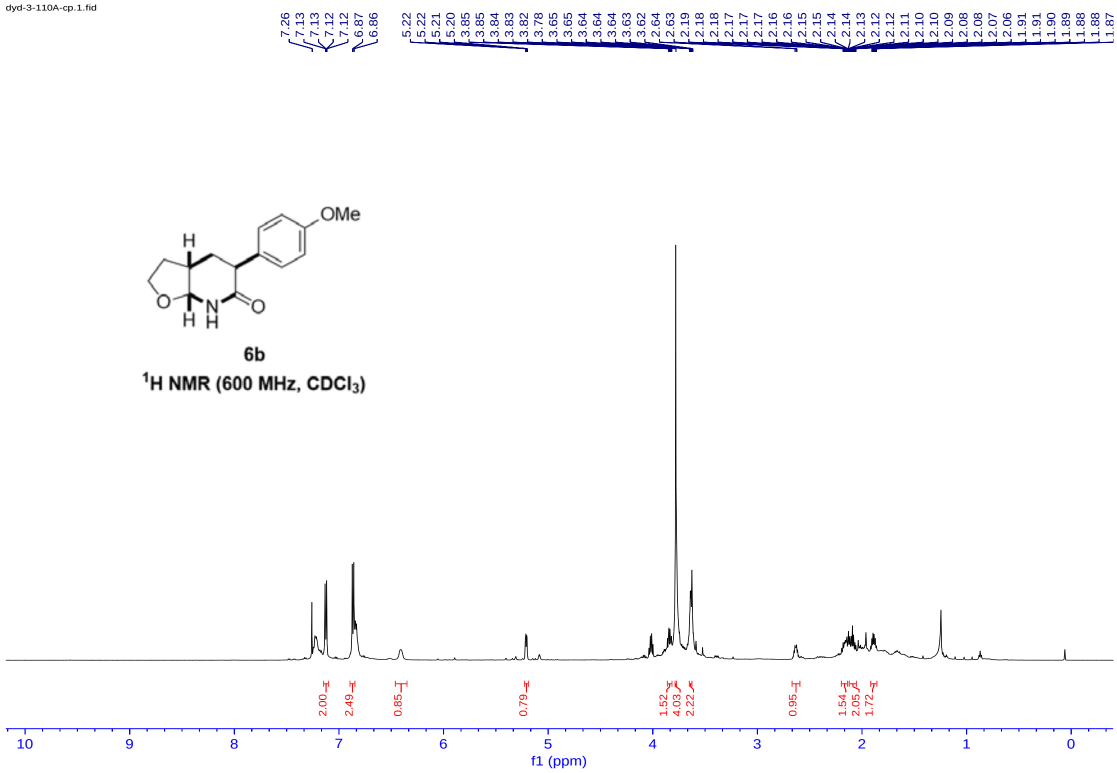

Supplementary Figure 224 ^{1}H NMR (600 MHz, 298K, CDCl$_3$) of **6b**

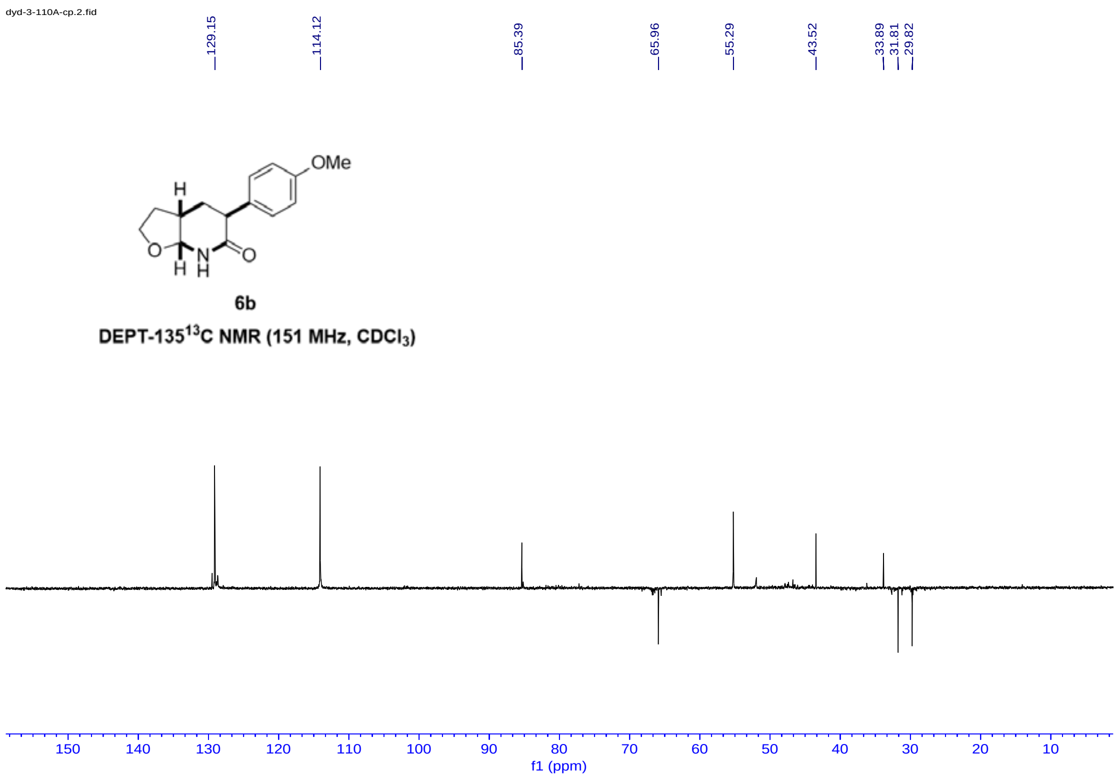

Supplementary Figure 225 DEPT-135 ^{13}C NMR (151 MHz, 298K, $CDCl_3$) of **6b**

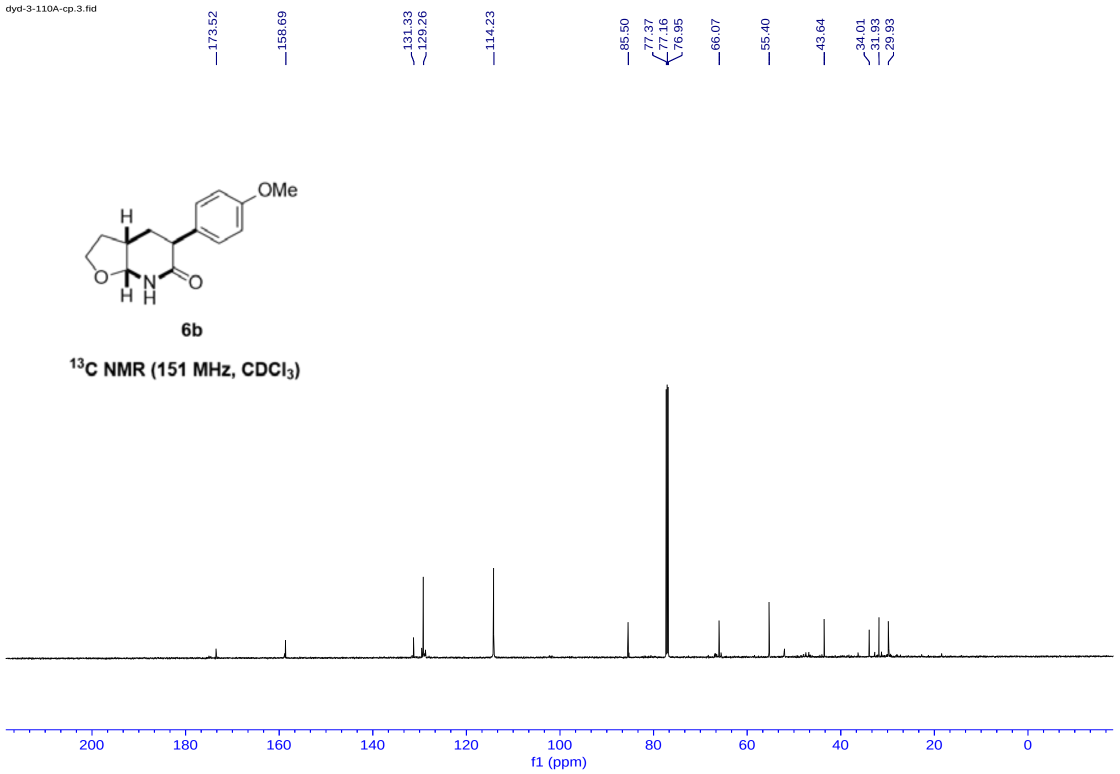

Supplementary Figure 226 ^{13}C NMR (151 MHz, 298K, $CDCl_3$) of **6b**

cis-5-(*tert*-butoxy)-3-(4-fluorophenyl)piperidin-2-one

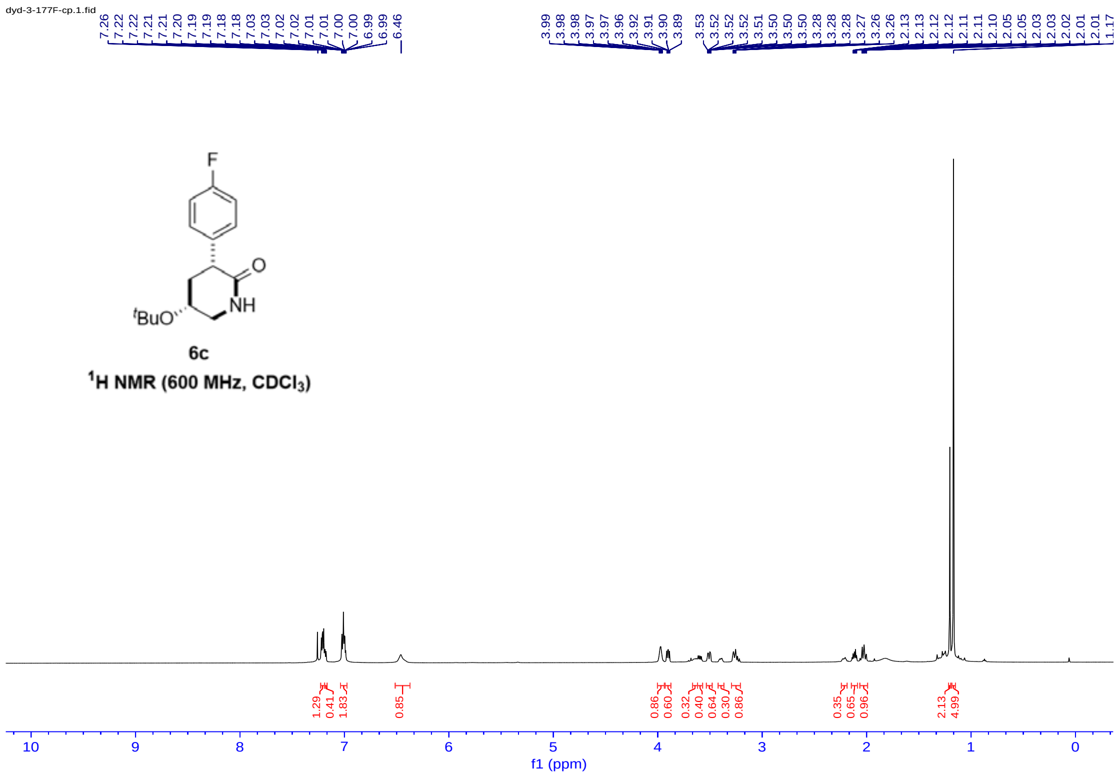

Supplementary Figure 227 ^{1}H NMR (600 MHz, 298K, $CDCl_3$) of **6c**

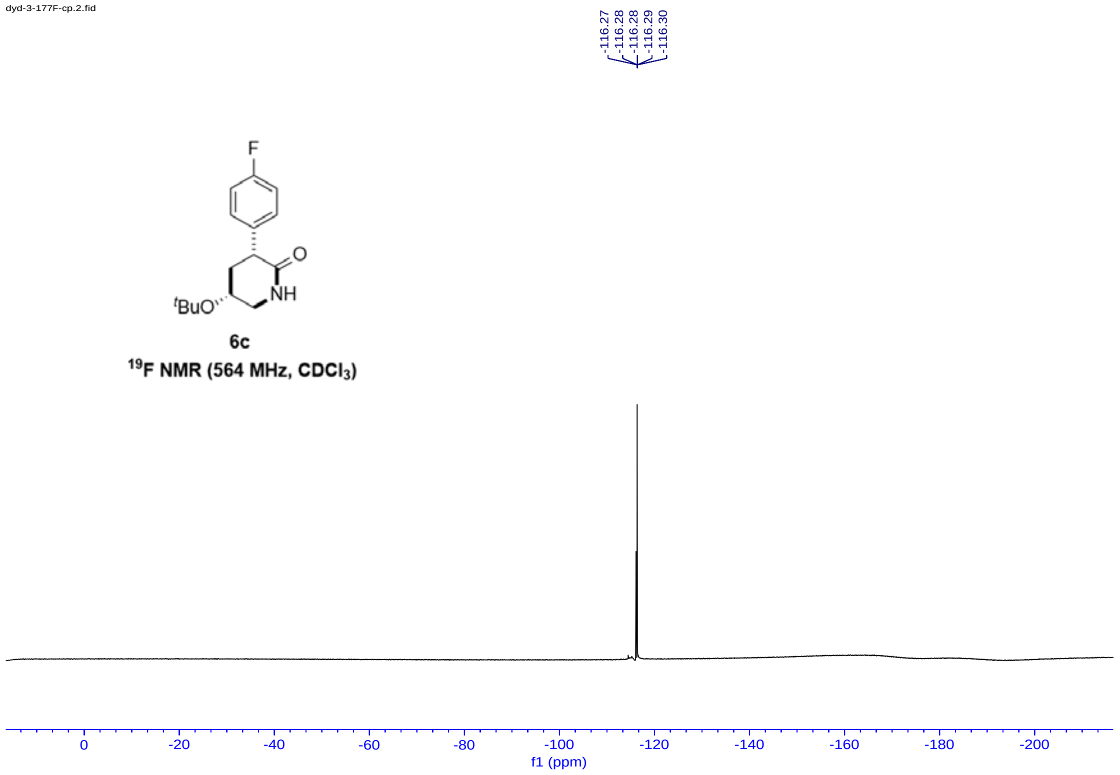

Supplementary Figure 228 ^{19}F NMR (564 MHz, 298K, $CDCl_3$) of **6c**

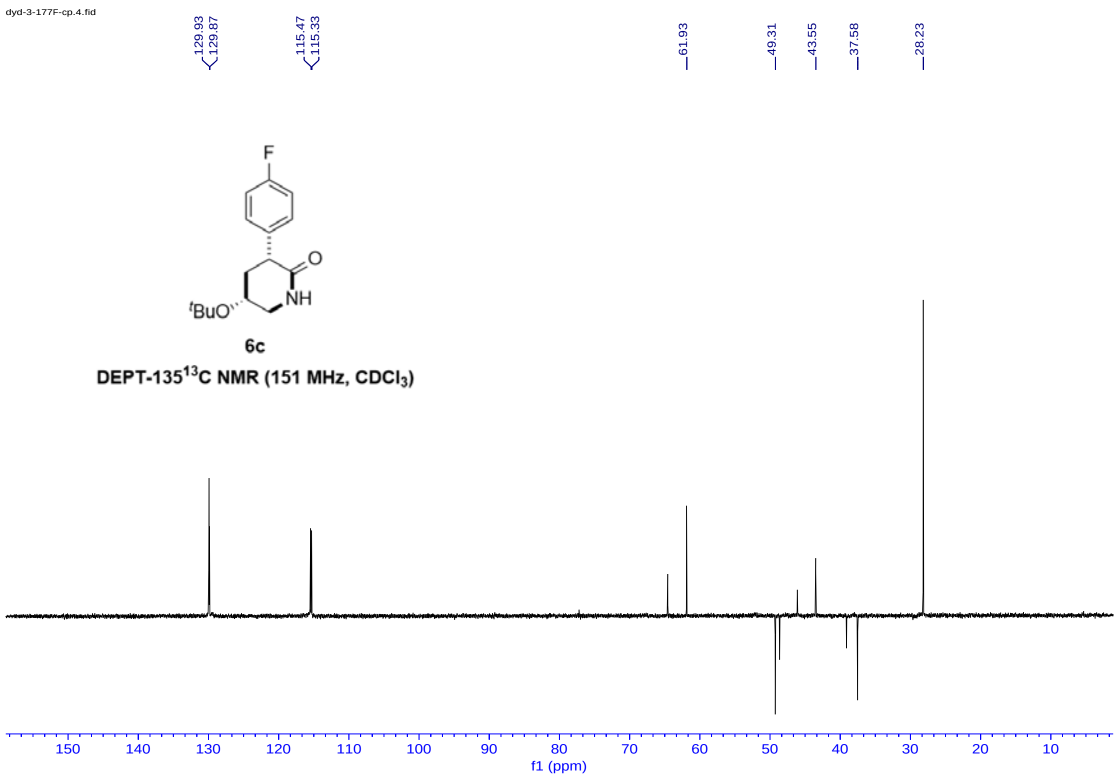

Supplementary Figure 229 DEPT-135 ^{13}C NMR (151 MHz, 298K, $CDCl_3$) of **6c**

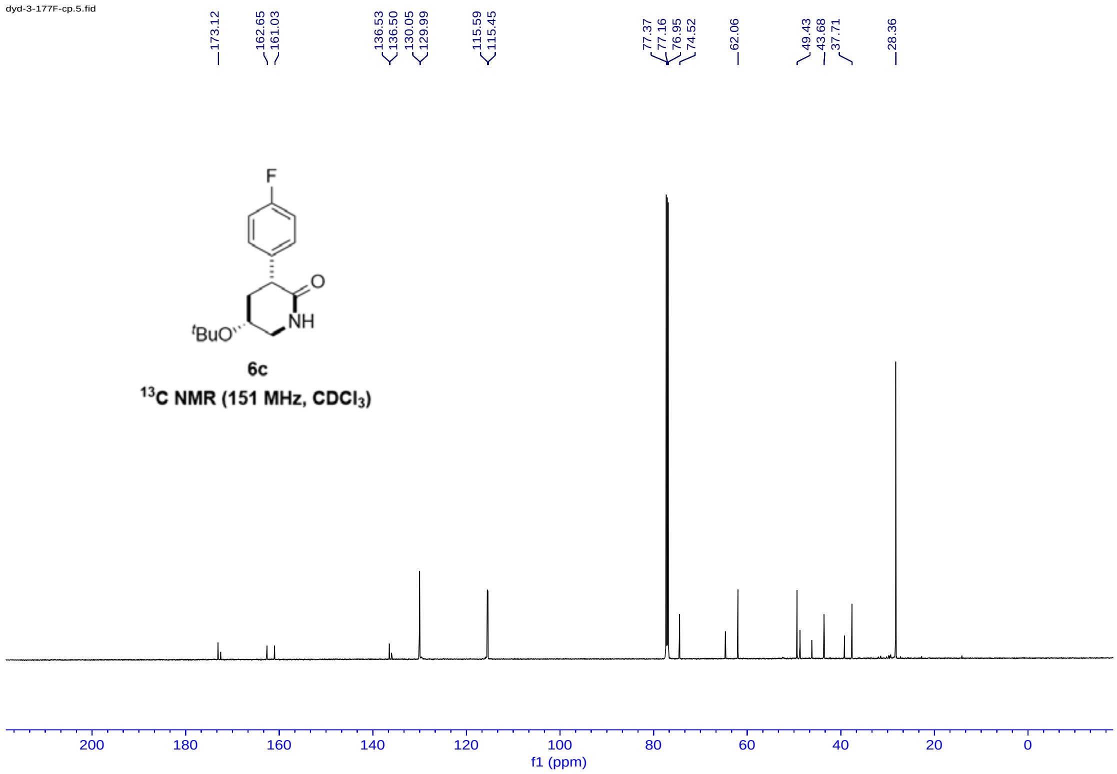

Supplementary Figure 230 ^{13}C NMR (151 MHz, 298K, $CDCl_3$) of **6c**

(4a*S*,7a*S*)-3-(4-fluorophenyl)-4a-methyloctahydro-2*H*-cyclopenta[*b*]pyridin-2-one

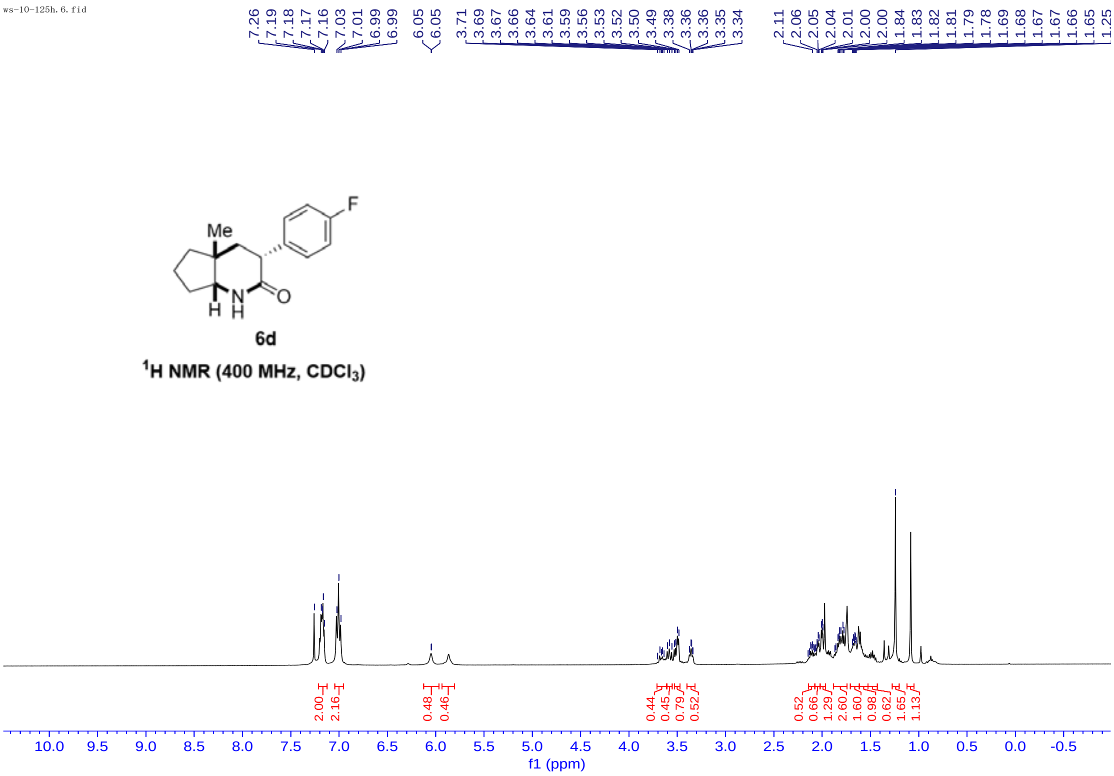

Supplementary Figure 231 ^{1}H NMR (400 MHz, 298K, $CDCl_3$) of **6d**

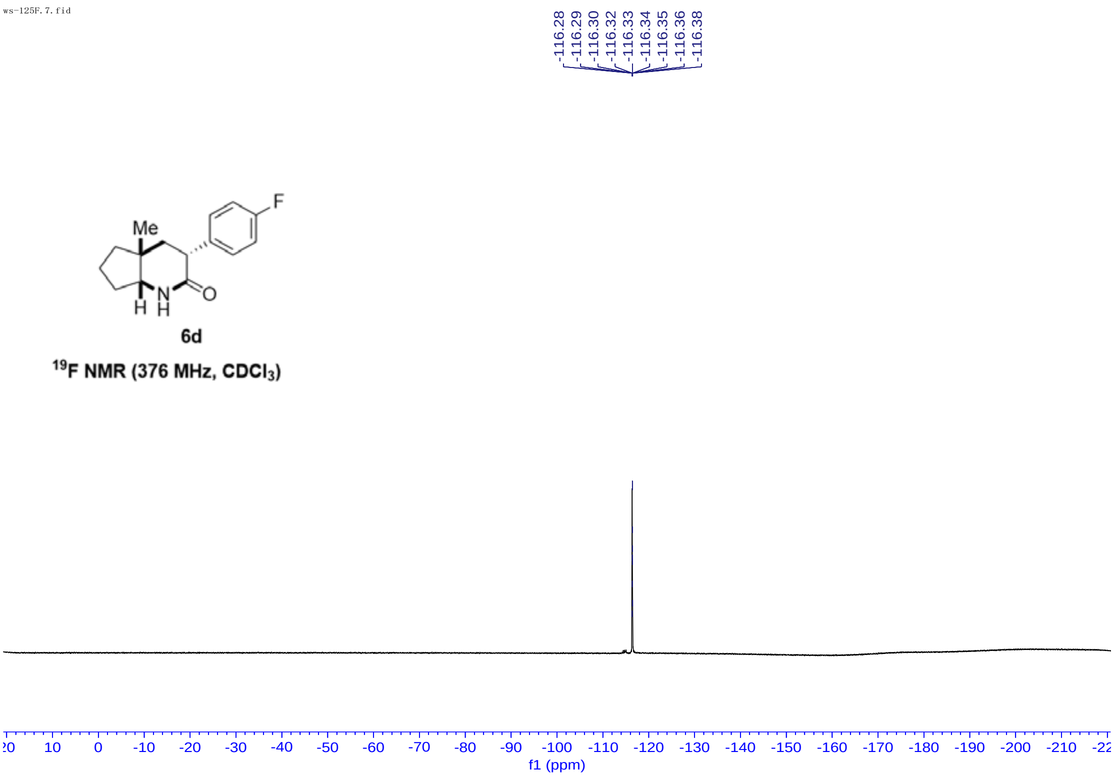

Supplementary Figure 232 ^{19}F NMR (376 MHz, 298K, $CDCl_3$) of **6d**

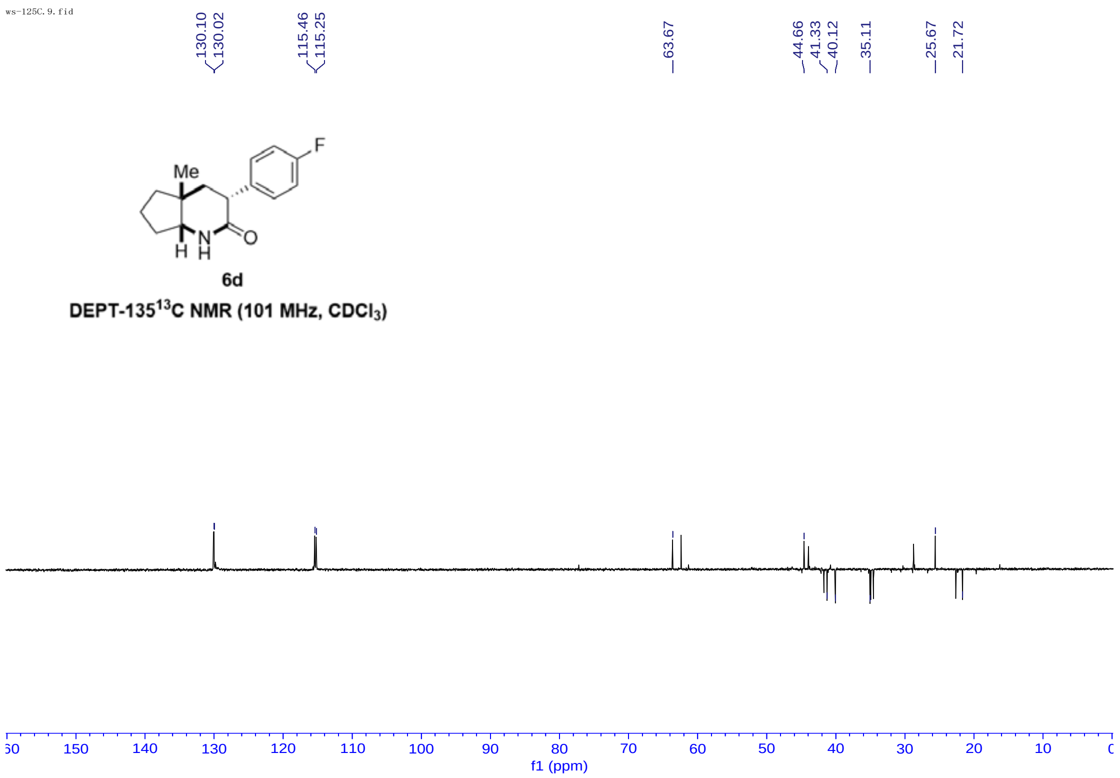

Supplementary Figure 233 DEPT-135 ^{13}C NMR (101 MHz, 298K, $CDCl_3$) of **6d**

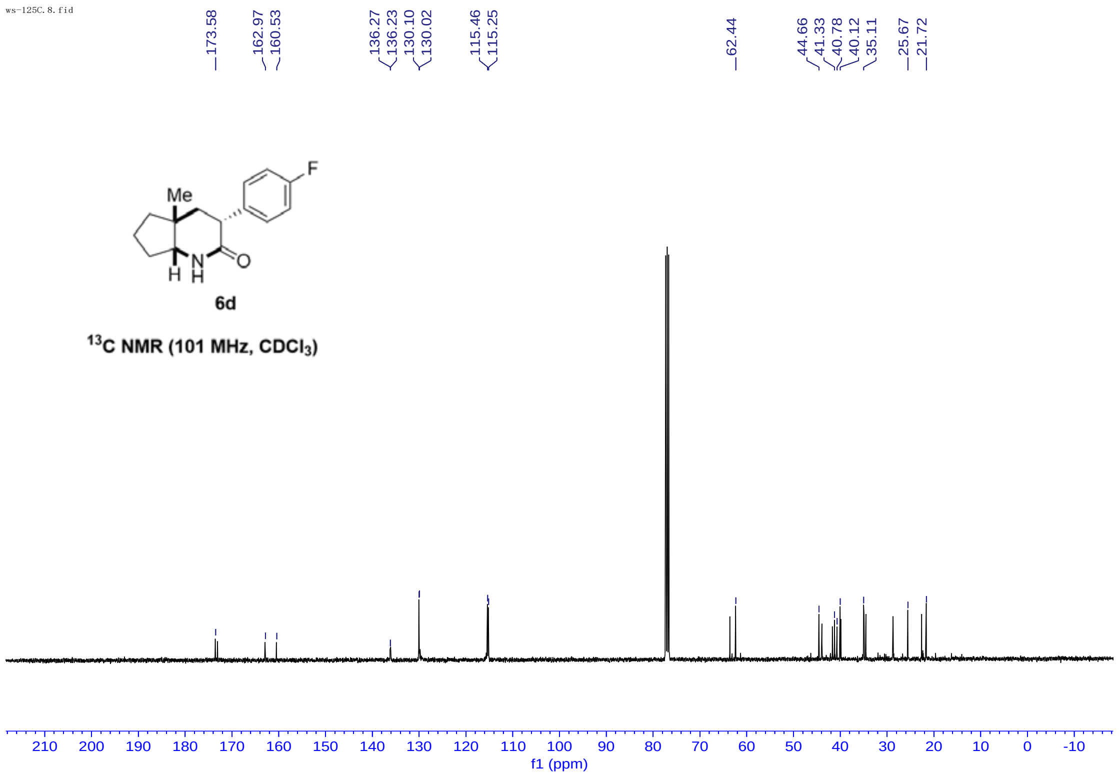

Supplementary Figure 234 ^{13}C NMR (101 MHz, 298K, $CDCl_3$) of **6d**

5,5-dibutyl-3-(4-fluorophenyl)-6-methylpiperidin-2-one

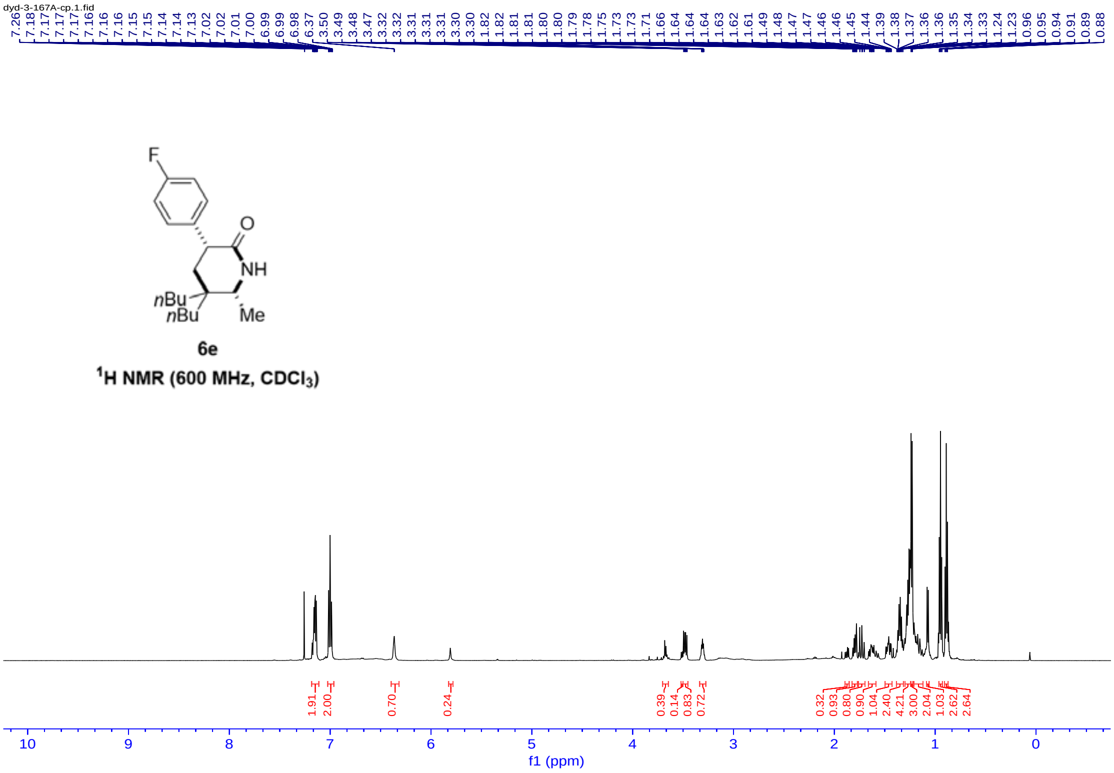

Supplementary Figure 235 ^{1}H NMR (600 MHz, 298K, $CDCl_3$) of **6e**

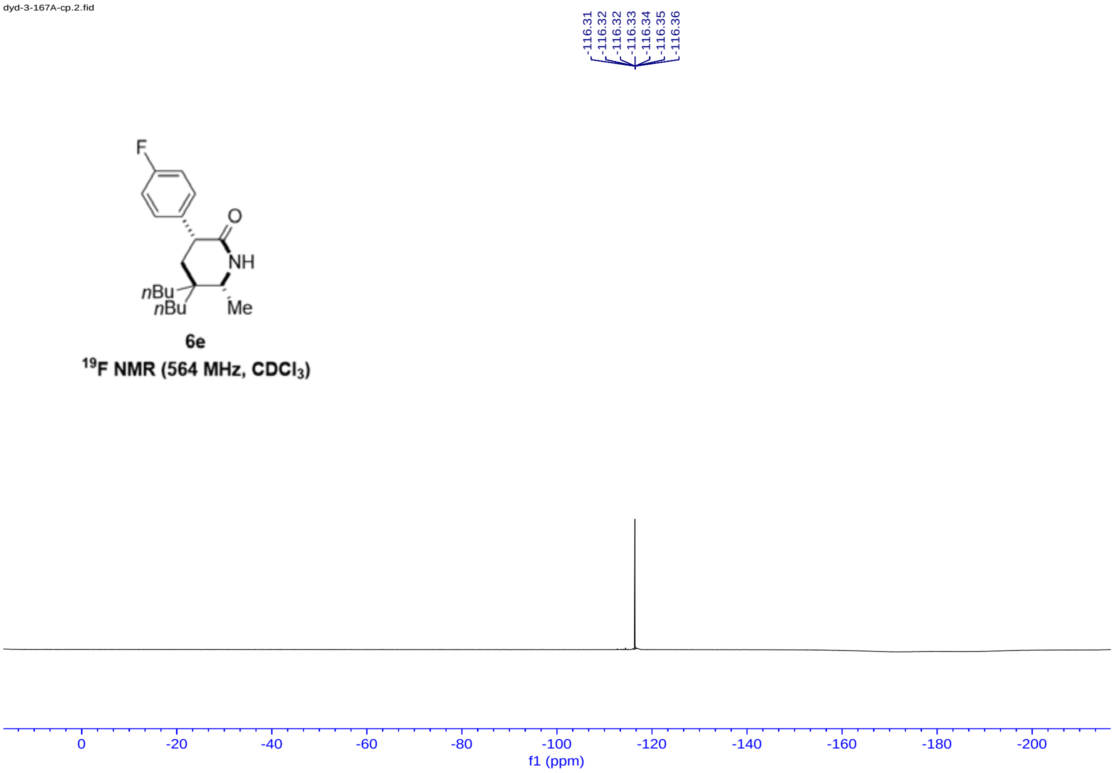

Supplementary Figure 236 ^{19}F NMR (564 MHz, 298K, $CDCl_3$) of **6e**

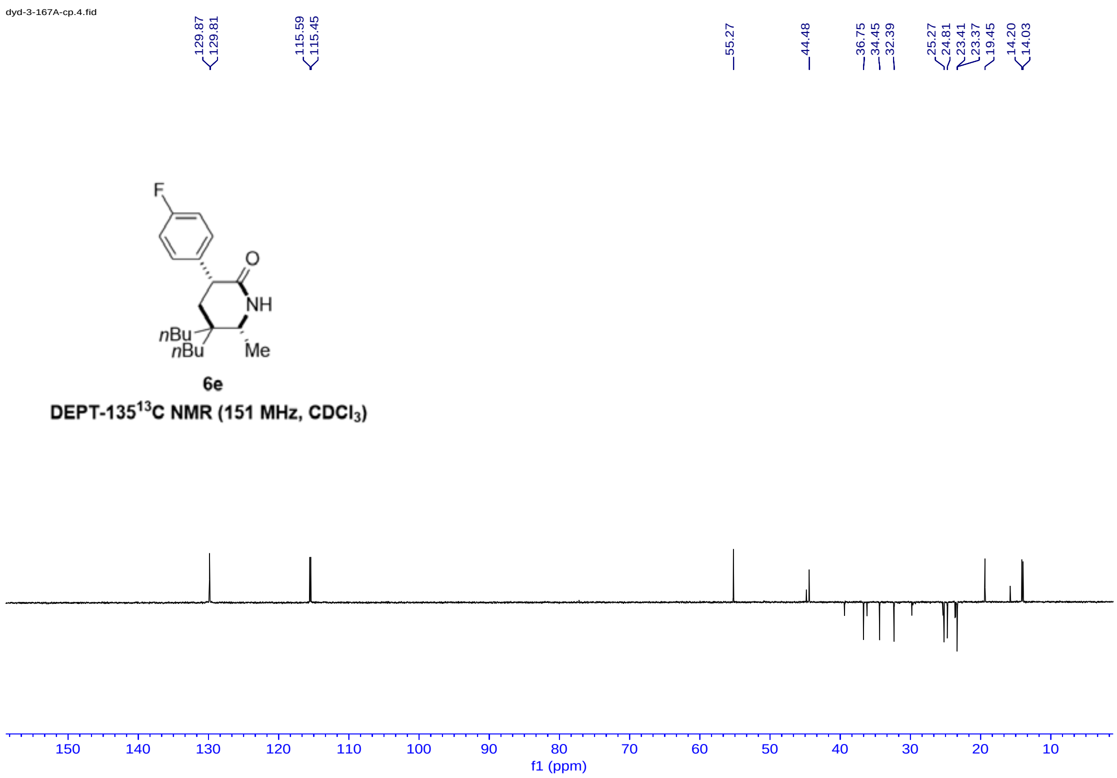

Supplementary Figure 237 DEPT-135 ^{13}C NMR (151 MHz, 298K, $CDCl_3$) of **6e**

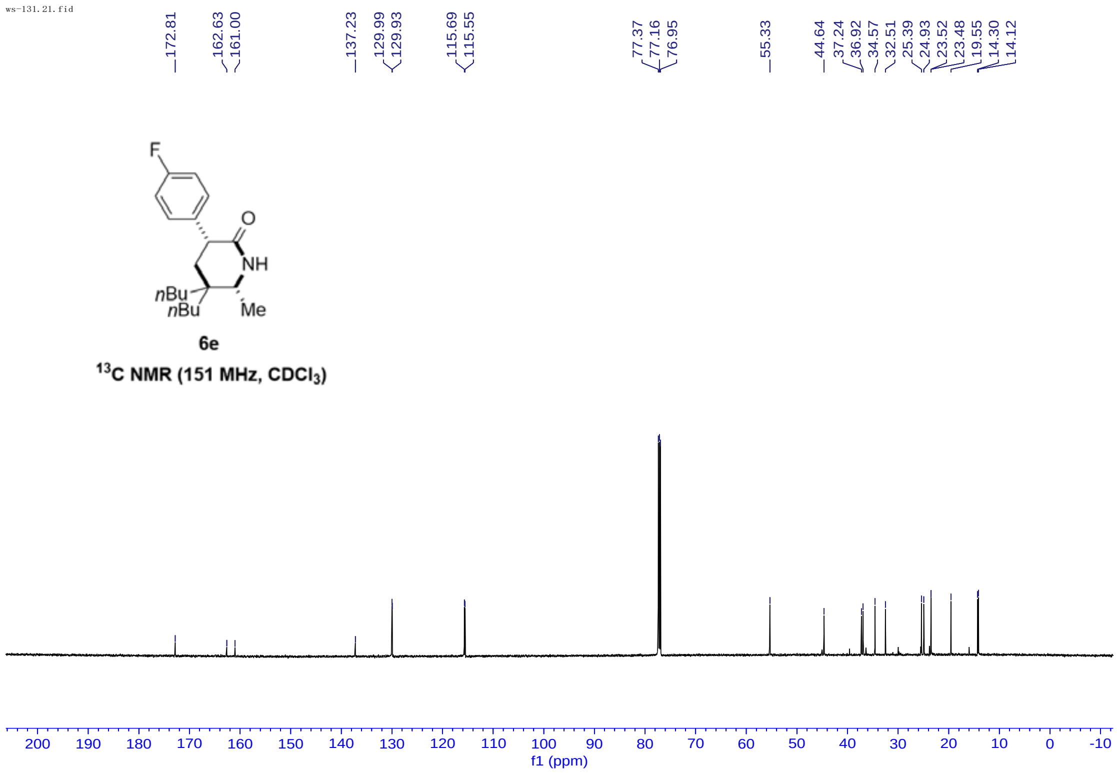

Supplementary Figure 238 ^{13}C NMR (151 MHz, 298K, $CDCl_3$) of **6e**

3-(4-fluorophenyl)-5,5-dimethyl-6-propylpiperidin-2-one

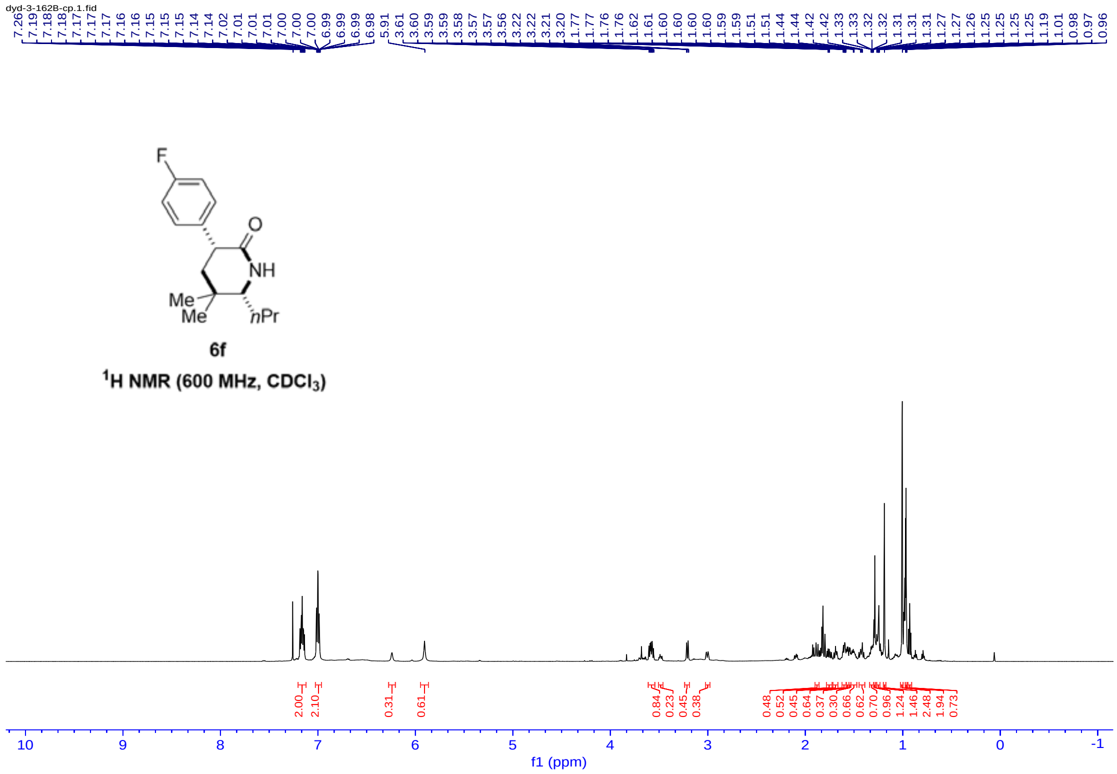

Supplementary Figure 239 ^{1}H NMR (600 MHz, 298K, $CDCl_3$) of **6f**

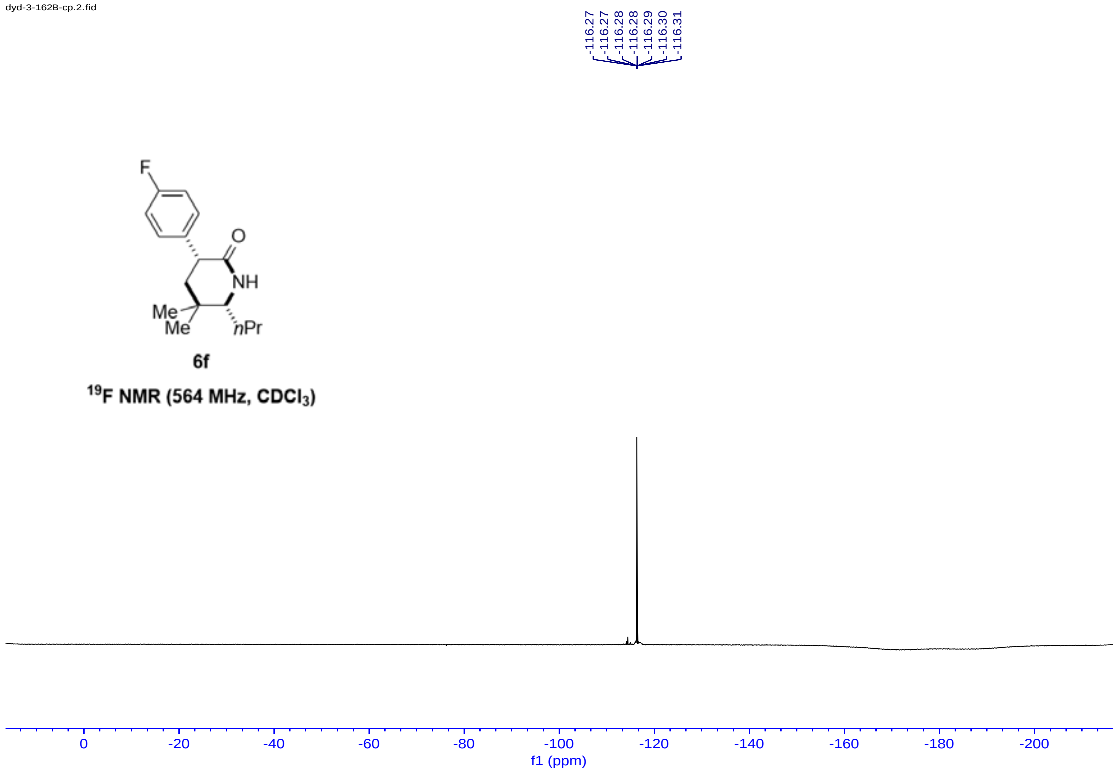

Supplementary Figure 240 ^{19}F NMR (564 MHz, 298K, $CDCl_3$) of **6f**

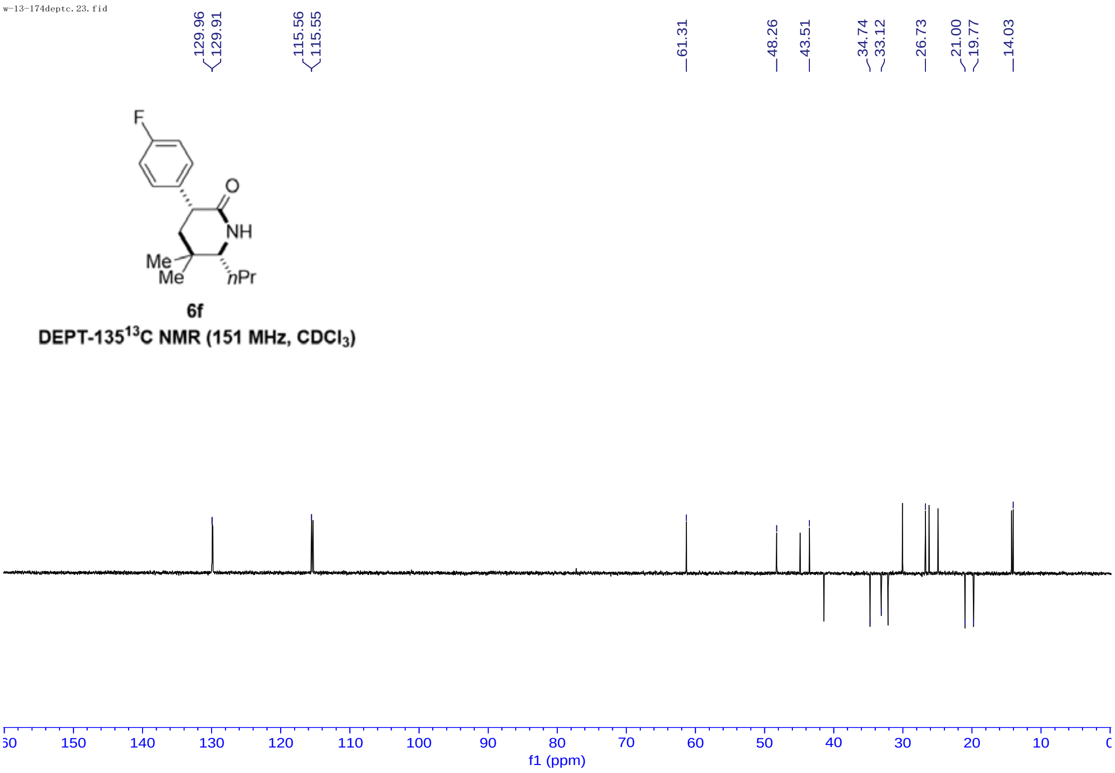

Supplementary Figure 241 DEPT-135 ^{13}C NMR (151 MHz, 298K, $CDCl_3$) of **6f**

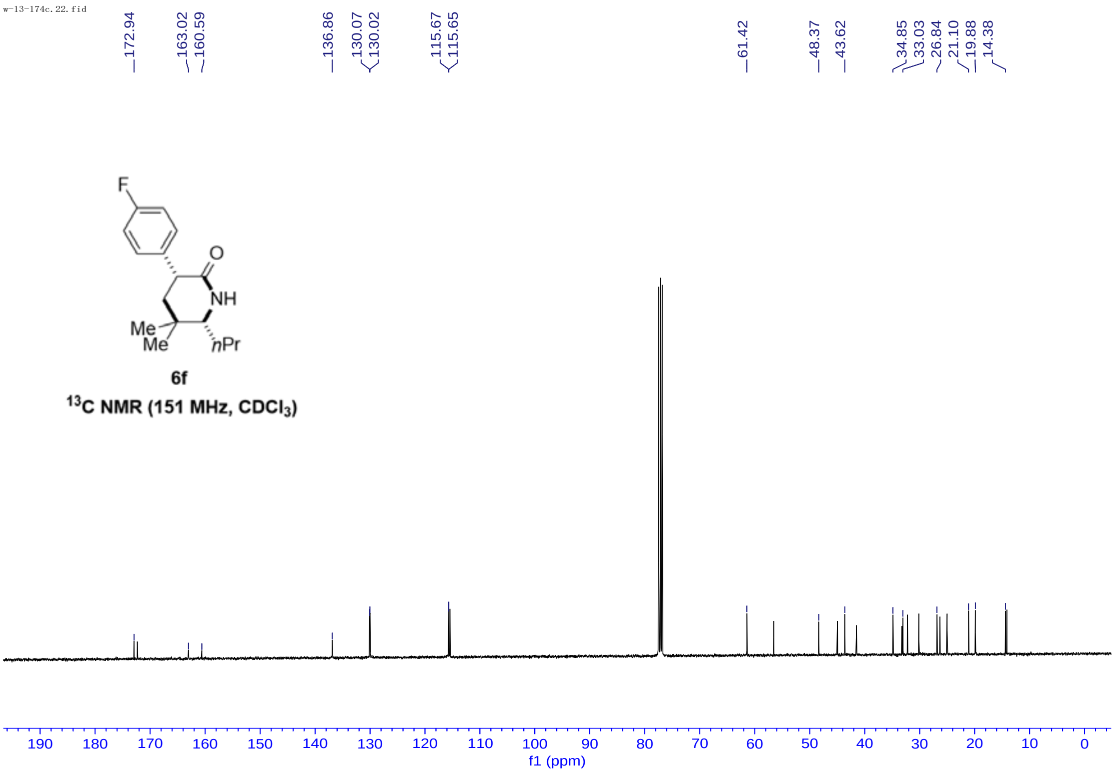

Supplementary Figure 242 ^{13}C NMR (151 MHz, 298K, $CDCl_3$) of **6f**

3-(4-fluorophenyl)-5-((8*R*,9*S*,13*S*,14*S*)-13-methyl-17-oxo-7,8,9,11,12,13,14,15,16,17-decahydro-6*H*-cyclopenta[*a*]phenanthren-3-yl)piperidin-2-one

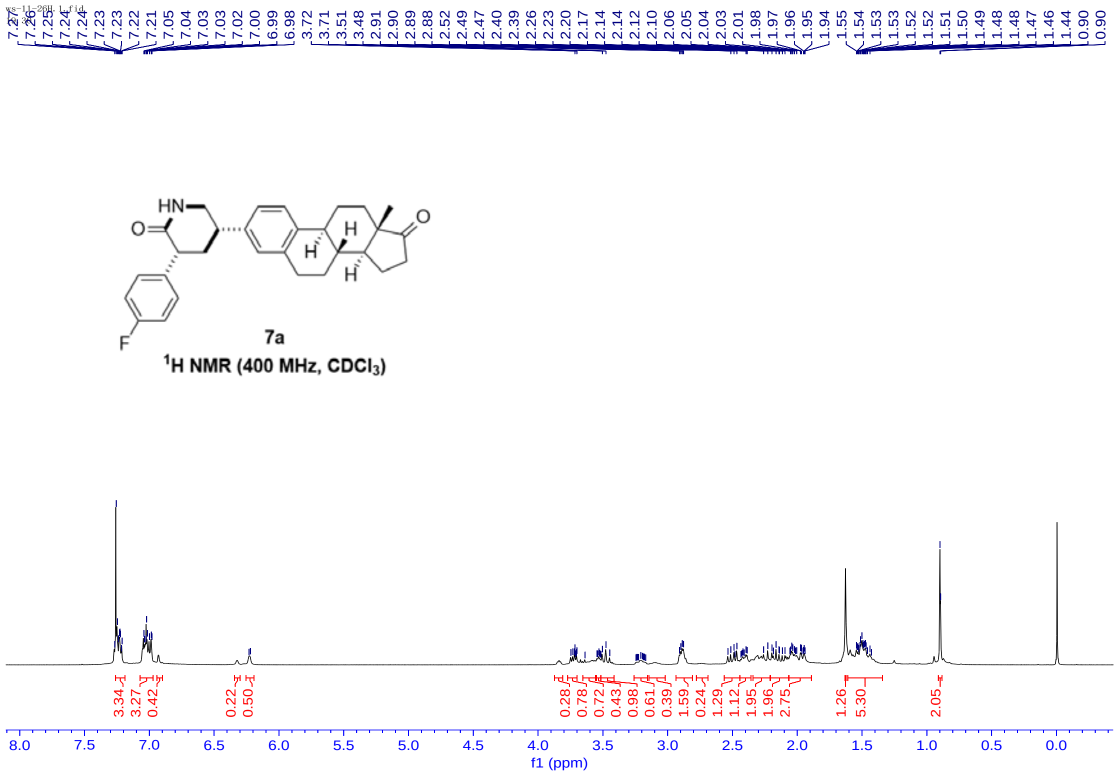

Supplementary Figure 243 ^{1}H NMR (400 MHz, 298K, $CDCl_3$) of **7a**

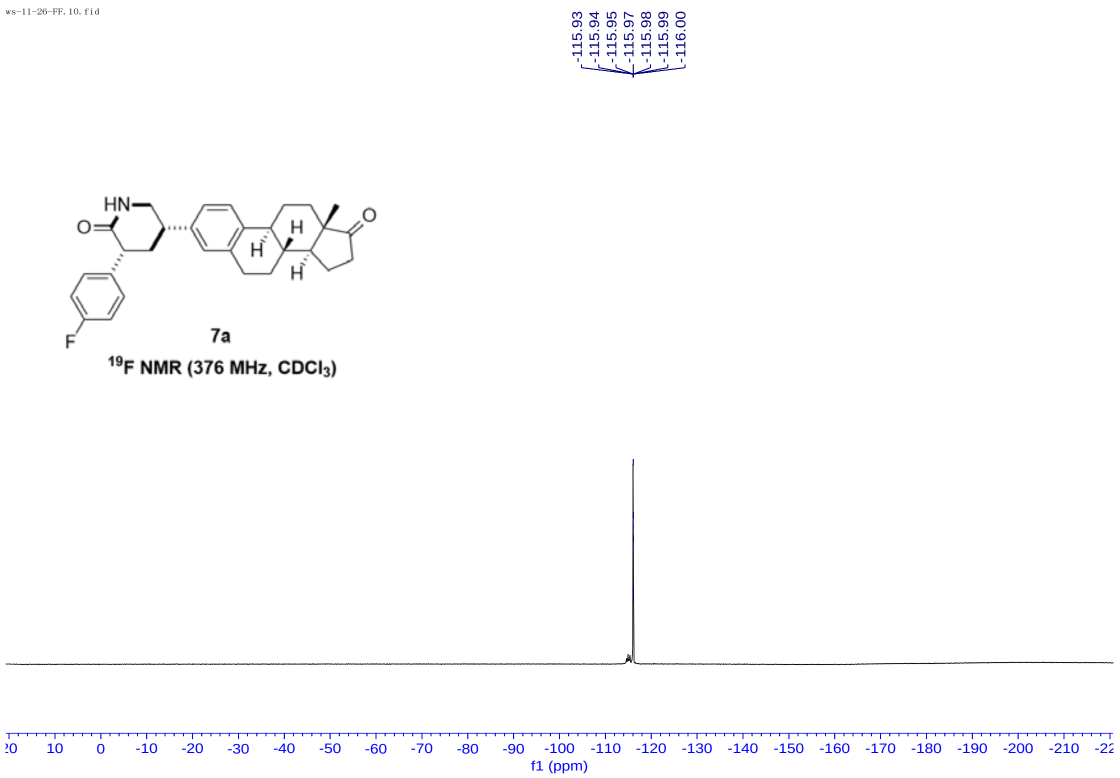

Supplementary Figure 244 ^{19}F NMR (376 MHz, 298K, $CDCl_3$) of **7a**

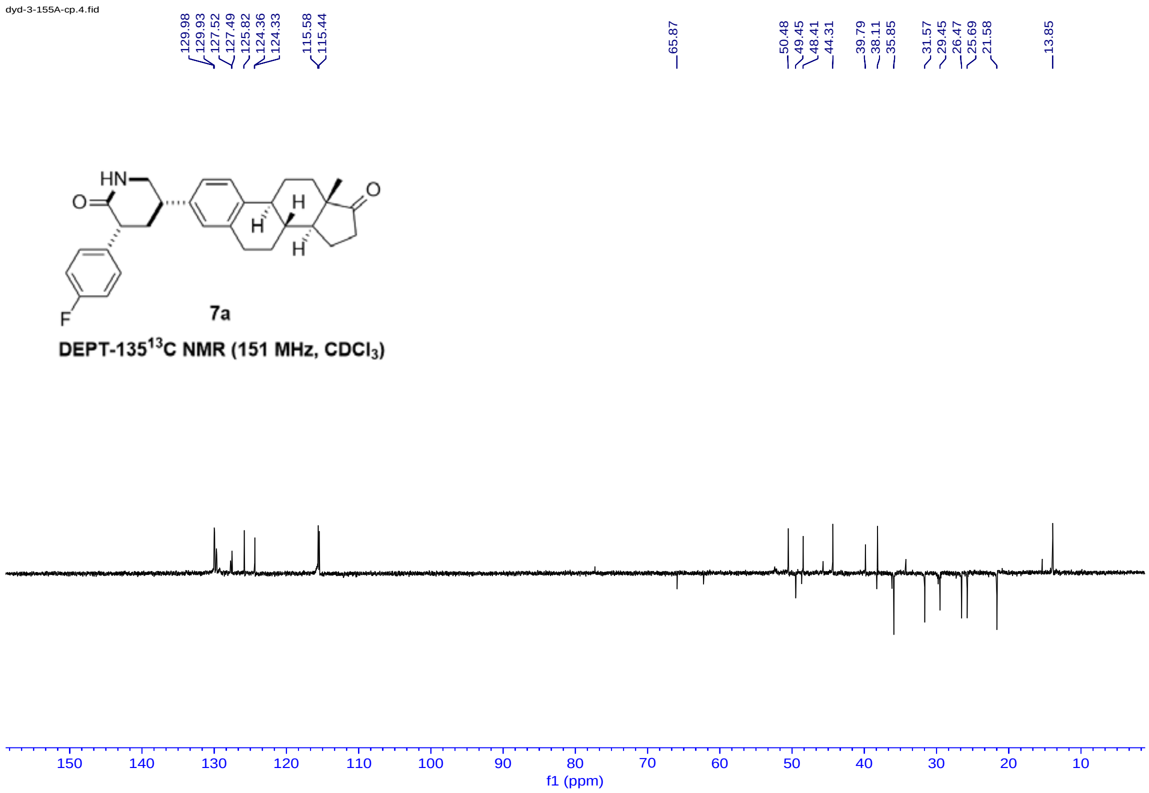

Supplementary Figure 245 DEPT-135 ^{13}C NMR (151 MHz, 298K, $CDCl_3$) of **7a**

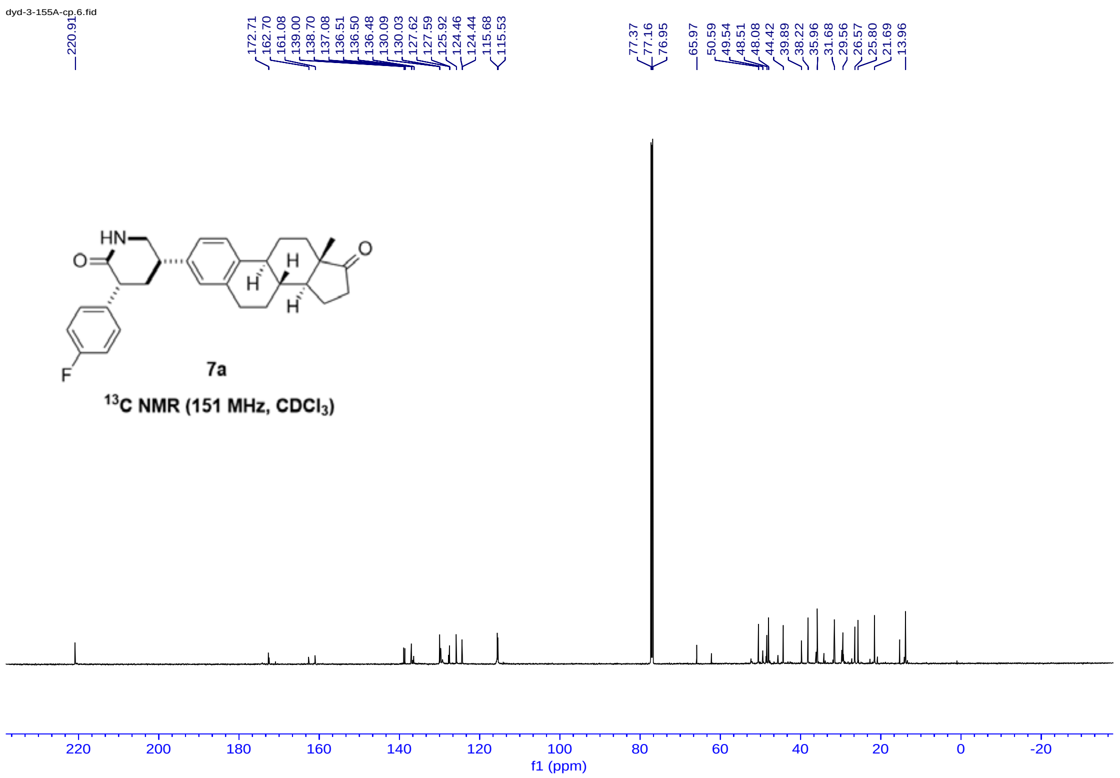

Supplementary Figure 246 ^{13}C NMR (151 MHz, 298K, $CDCl_3$) of **7a**

3-(4-fluorophenyl)-5-(((1*R*,2*S*,5*R*)-2-isopropyl-5-methylcyclohexyl)oxy)piperidin-2-one

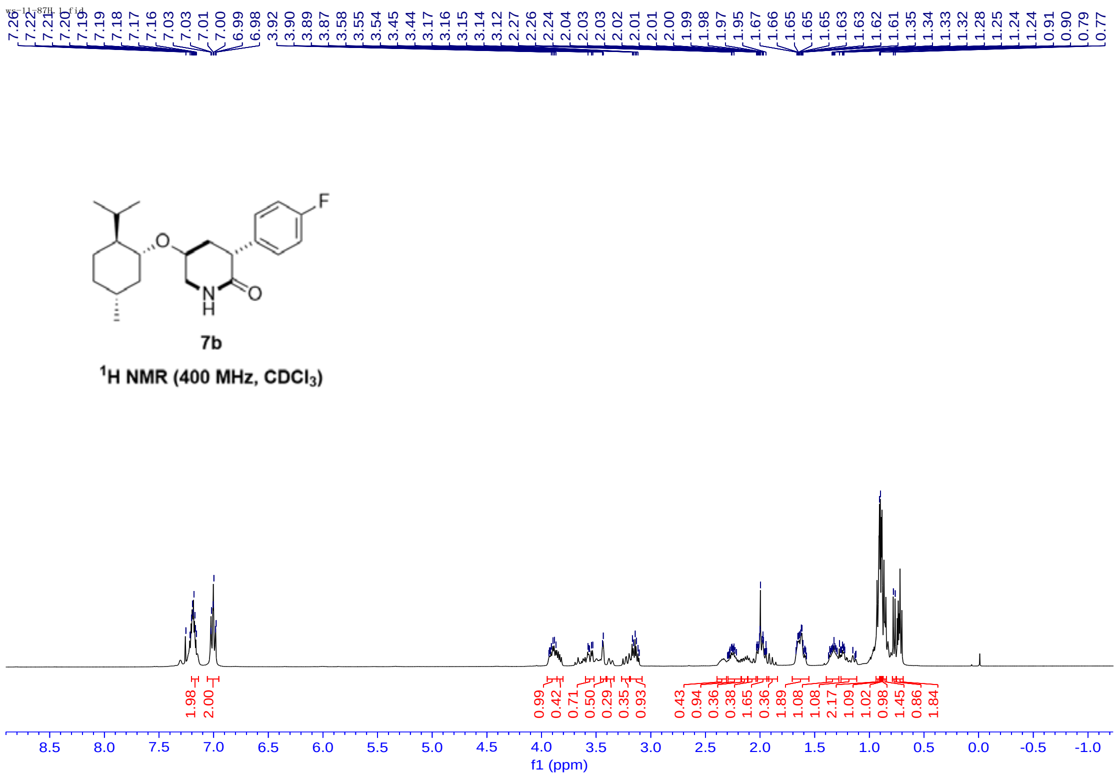

Supplementary Figure 247 ^{1}H NMR (400 MHz, 298K, $CDCl_3$) of **7b**

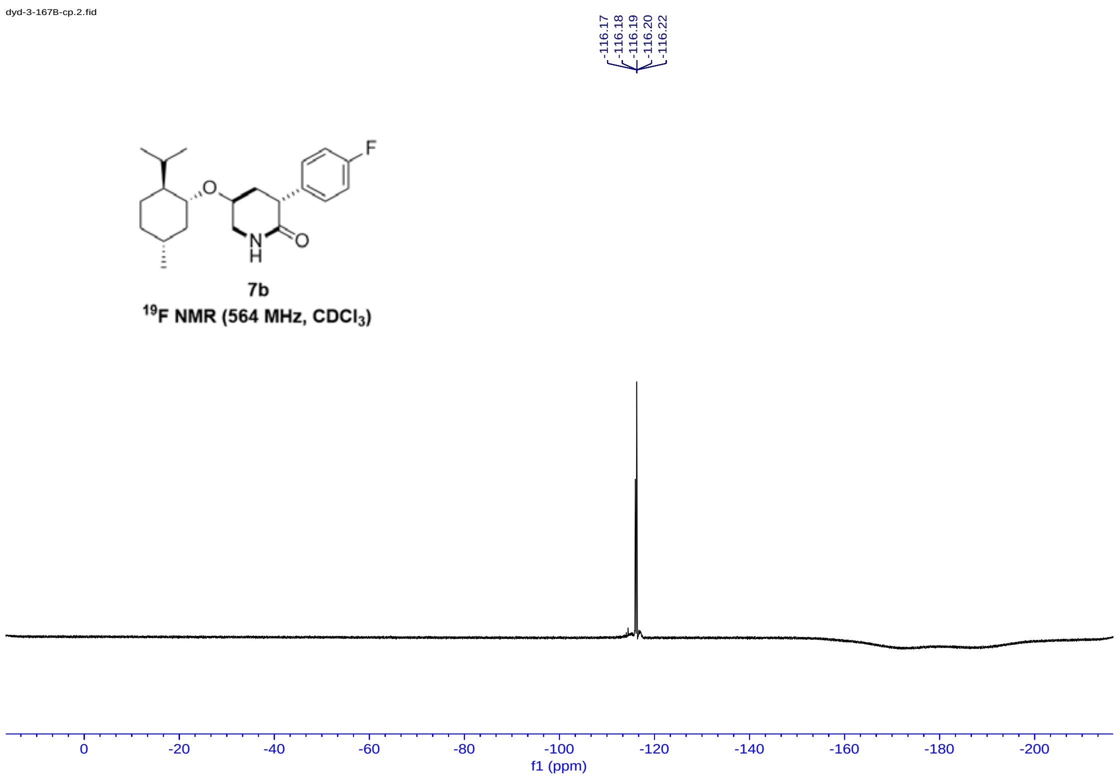

Supplementary Figure 248 ^{19}F NMR (564 MHz, 298K, $CDCl_3$) of **7b**

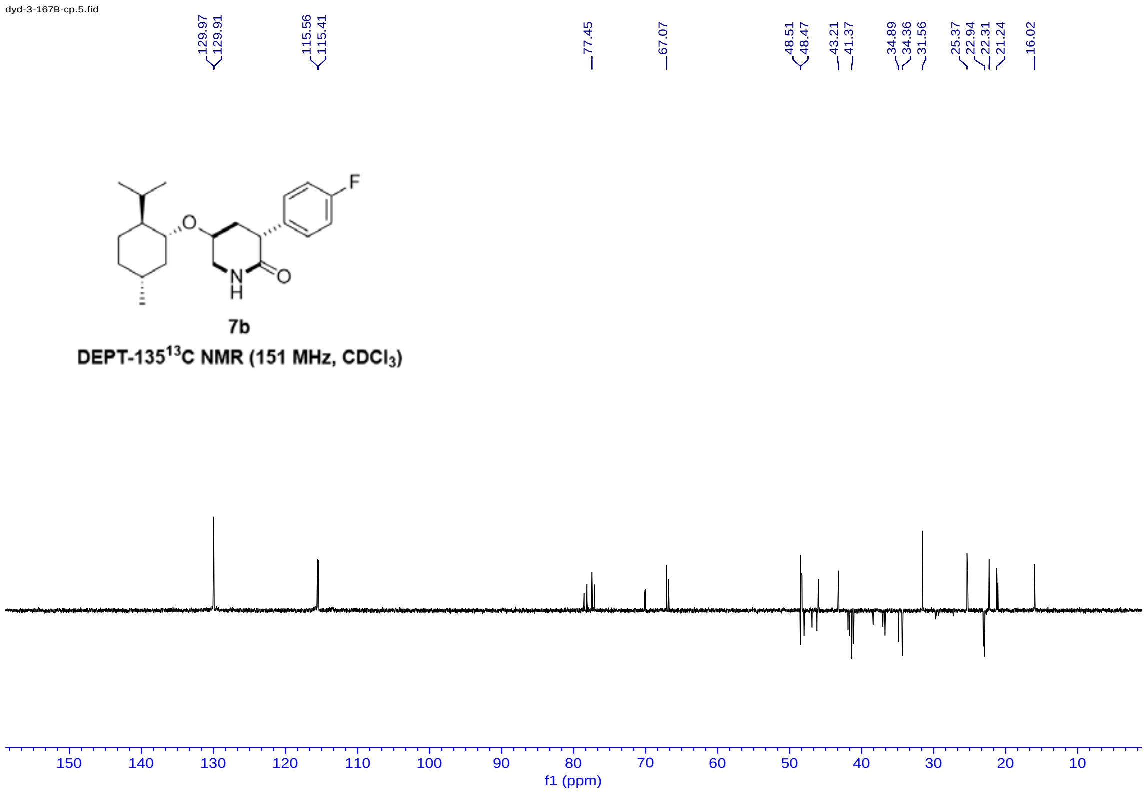

Supplementary Figure 249 DEPT-135 ^{13}C NMR (151 MHz, 298K, $CDCl_3$) of **7b**

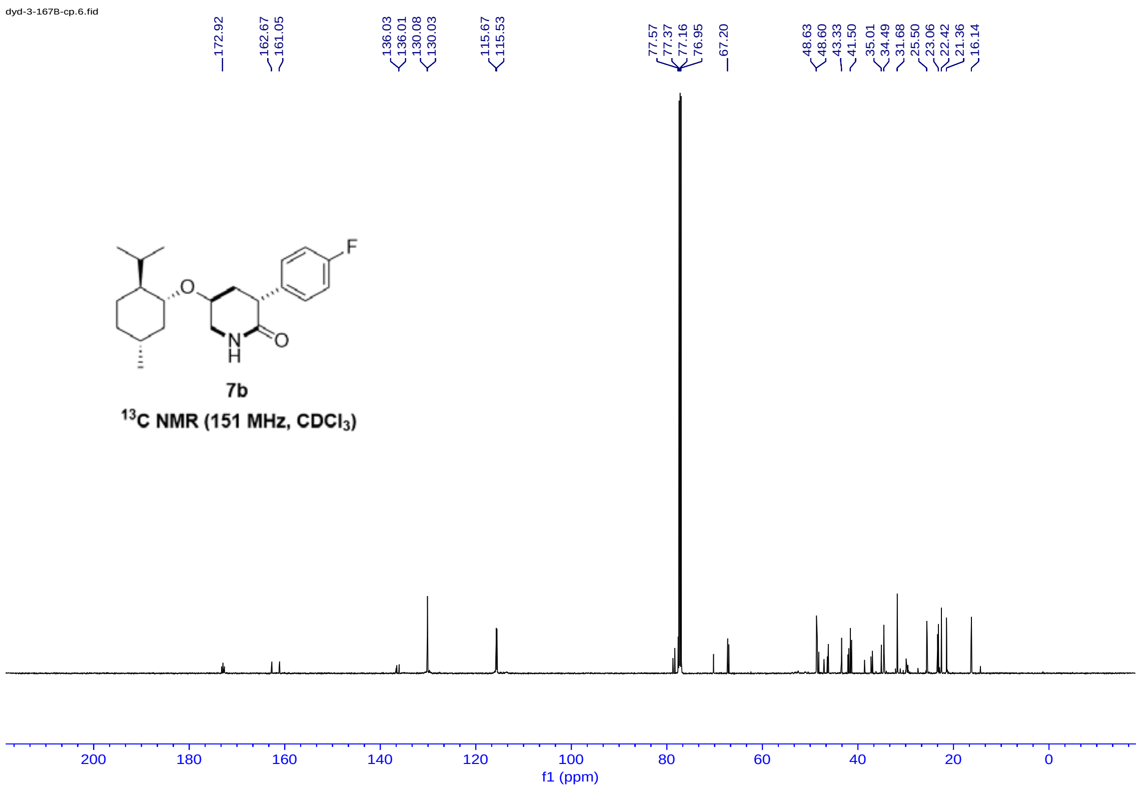

Supplementary Figure 250 ^{13}C NMR (151 MHz, 298K, $CDCl_3$) of **7b**

(4a*R*,5*R*,6a*S*,9*R*,9a*R*)-3-(4-fluorophenyl)-4a,6,6,9-tetramethyldecahydro-1*H*-5,9a-methanoazuleno[5,6-*b*]pyridin-2(3*H*)-one

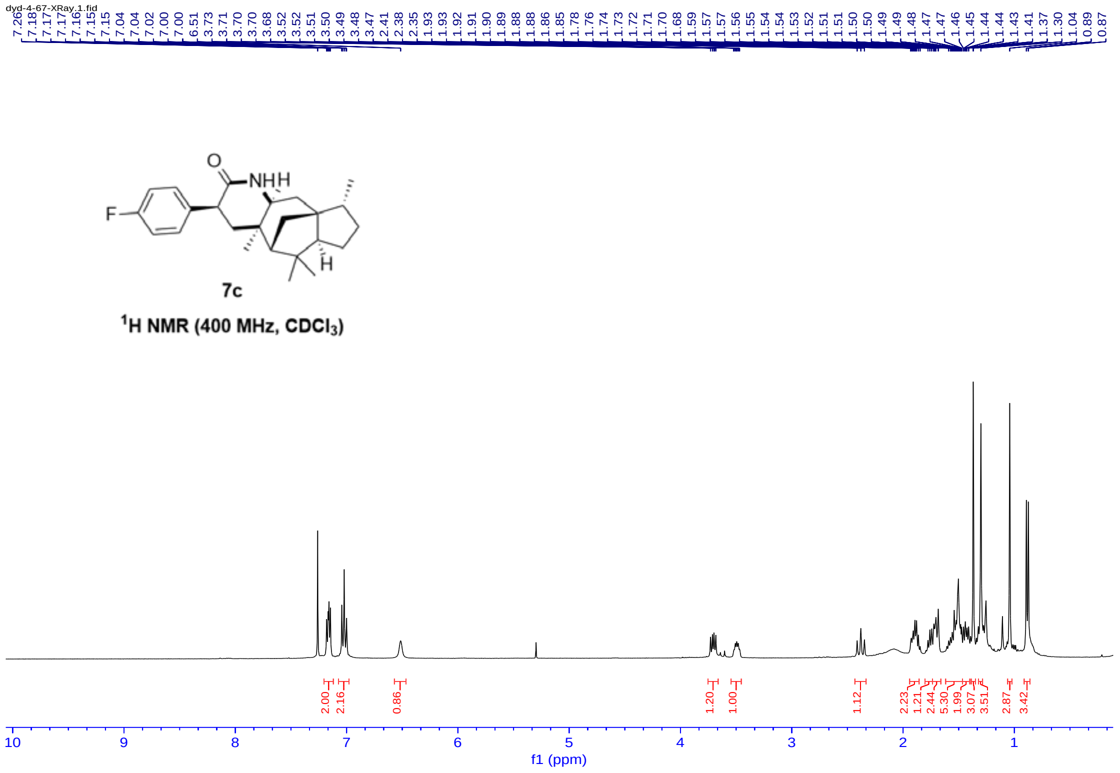

Supplementary Figure 251 ^{1}H NMR (400 MHz, 298K, $CDCl_3$) of **7c**

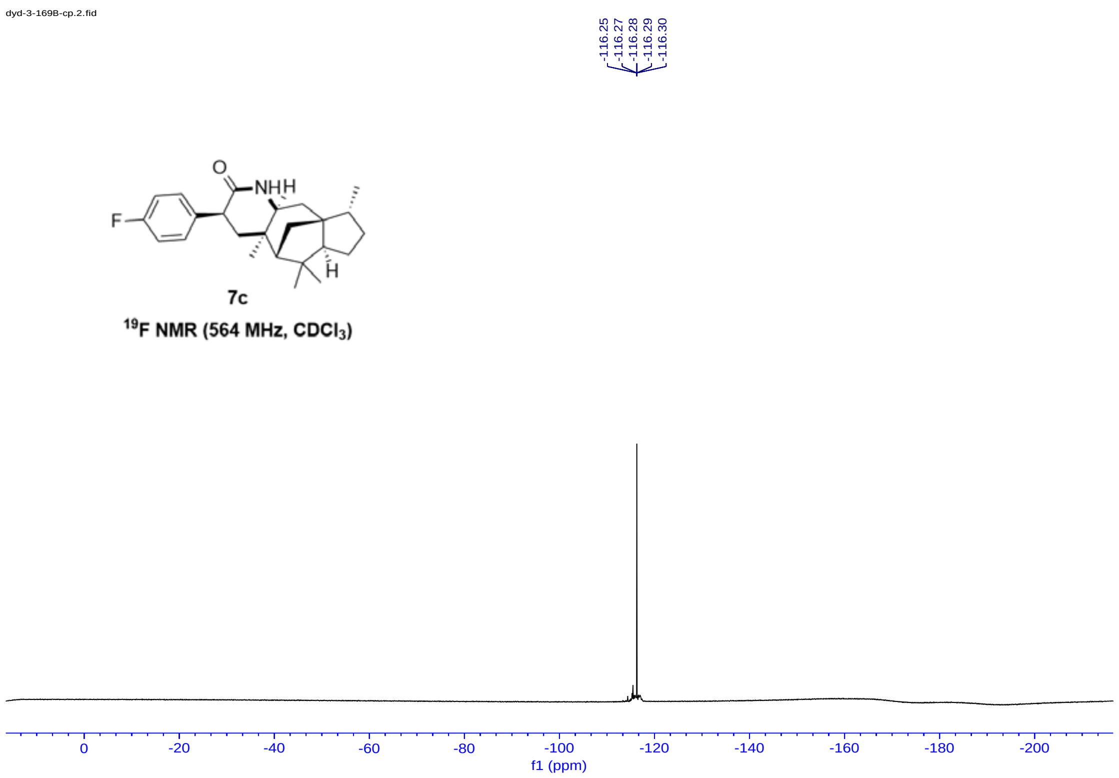

Supplementary Figure 252 ^{19}F NMR (564 MHz, 298K, $CDCl_3$) of **7c**

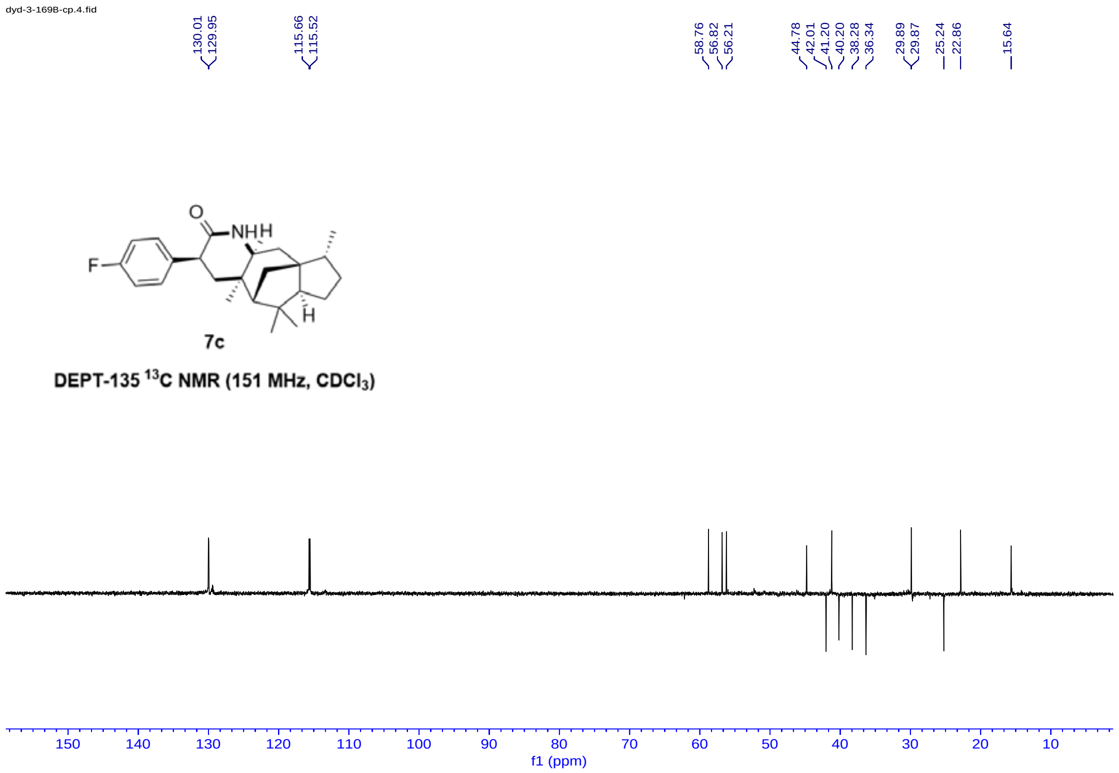

Supplementary Figure 253 DEPT-135 ^{13}C NMR (151 MHz, 298K, $CDCl_3$) of **7c**

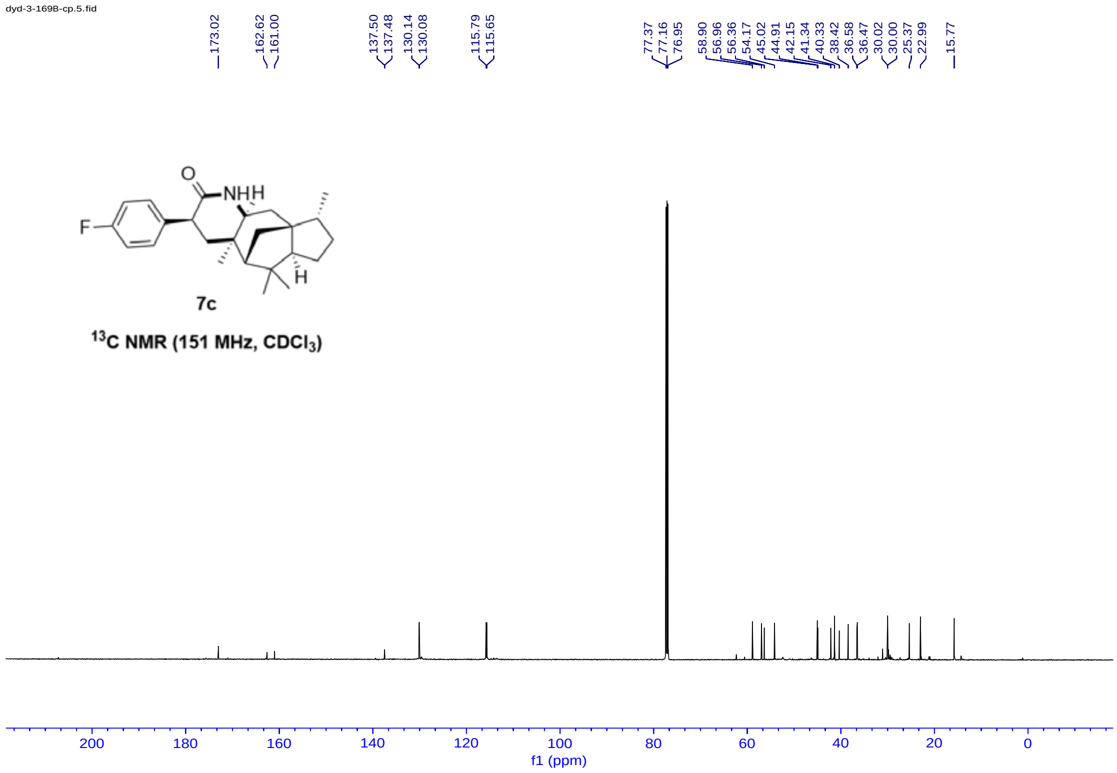

Supplementary Figure 254 ^{13}C NMR (151 MHz, 298K, $CDCl_3$) of **7c**

Supplementary References

1. Tabele, C.; Cohen, A.; Curti, C.; Bouhlel, A.; Hutter, S.; Remusat, V.; Primas, N.; Terme, T.; Azas, N.; Vanelle, P. *Eur. J. Med. Chem.* **2014**, *87*, 440.
2. Occhialini, G.; Palani, V.; Wendlandt, A. E. *J. Am. Chem. Soc.* **2022**, *144*, 145.
3. Kolotuchin, S. V.; Meyers, A. I. *J. Org. Chem*. **1999**, *64*, 7921.
4. Aoyama, H.; Tokunaga, M.; Hiraiwa, S.; Shirogane, Y.; Obora, Y.; Tsuji, Y. *Org. Lett.* **2004**, *6*, 509.
5. Baraki, H.; Habaue, S.; Okamoto, Y. *Polym. J.* **2001**, *33*, 450.
6. Chavan, S. P.; Pathak, A. B.; Pandey, A.; Kalkote, U. R. *Synth. Commun*. **2007**, *37*, 4253.
7. Shu, C.; Mega, R. S.; Andreassen, B. J.; Noble, A.; Aggarwal, V. K. *Angew. Chem. Int. Ed.* **2018**, *57*, 15430.
8. Zhuang, J.; Zhao, B.; Meng, X.; Schiffman, J. D.; Perry, S. L.; Vachet, R. W.; Thayumanavan, S. *Chem. Sci.* **2020**, *11*, 2103.
9. Farley, A. J. M.; Sandford, C.; Dixon, D. J. *J. Am. Chem. Soc.* **2015**, *137*, 15992.
10. Wang, S.; Otani, Y.; Liu, X.; Kawahata, M.; Yamaguchi K.; Ohwada, T. *J. Org. Chem.* **2014**, *79*, 5287.
11. Zhu, Q.; Nocera, D. G. *J. Am. Chem. Soc*. **2020**, *142*, 17913.
12. Jiang, B.; Zhao, M.; Li, S.-S.; Hu, Y.-H.; Loh, T.-P. *Angew. Chem. Int. Ed*. **2018**, *57*, 555.
13. Arias-Rotondo, D. M.; McCusker, J. K. *Chem. Soc. Rev*. **2016**, *45*, 5803.
14. Romero, N. A.; Nicewicz, D. A. *J. Am. Chem. Soc*. **2014**, *136*, 17024.
15. Qin, Y.; Zhu, Q.; Sun, R.; Ganley, J. M.; Knowles, R. R.; Nocera, D. G. *J. Am. Chem. Soc.* **2021**, *143*, 10232.

16. Hanchard, C. G.; Parker, C. A. *Proc. Roy. Soc. (London)*, **1956**, *A235*, 518.

17. Kuhn, H. J.; Braslavsky, S. E.; Schmidt, R. Chemical actinometry (IUPAC technical report). *Pure Appl. Chem.* **2004**, *76*, 2105.

18. Monalti, M.; Credi, A.; Prodi, L.; Gandolfi, M. T. Chemical actinometry. Handbook of photochemistry, 3re ed, Taylor & Francis group, LLC. Boca Raton, FL, **2006**, 601.
